# Transcriptional Regulation of Carbohydrate Metabolism in the Human Pathogen *Candida albicans*

**DOI:** 10.1371/journal.ppat.1000612

**Published:** 2009-10-09

**Authors:** Christopher Askew, Adnane Sellam, Elias Epp, Hervé Hogues, Alaka Mullick, André Nantel, Malcolm Whiteway

**Affiliations:** 1 Biotechnology Research Institute, National Research Council of Canada, Montréal, Québec, Canada; 2 Department of Biology, McGill University, Montréal, Québec, Canada; 3 Department of Anatomy and Cell Biology, McGill University, Montréal, Québec, Canada; 4 Départment de Microbiologie et Immunologie, l'Université de Montréal, Montréal, Québec, Canada; University of Melbourne, Australia

## Abstract

Glycolysis is a metabolic pathway that is central to the assimilation of carbon for either respiration or fermentation and therefore is critical for the growth of all organisms. Consequently, glycolytic transcriptional regulation is important for the metabolic flexibility of pathogens in their attempts to colonize diverse niches. We investigated the transcriptional control of carbohydrate metabolism in the human fungal pathogen *Candida albicans* and identified two factors, Tye7p and Gal4p, as key regulators of glycolysis. When respiration was inhibited or oxygen was limited, a *gal4tye7 C. albicans* strain showed a severe growth defect when cultured on glucose, fructose or mannose as carbon sources. The *gal4tye7* strain displayed attenuated virulence in both *Galleria* and mouse models as well, supporting the connection between pathogenicity and metabolism. Chromatin immunoprecipitation coupled with microarray analysis (ChIP-CHIP) and transcription profiling revealed that Tye7p bound the promoter sequences of the glycolytic genes and activated their expression during growth on either fermentable or non-fermentable carbon sources. Gal4p also bound the glycolytic promoter sequences and activated the genes although to a lesser extent than Tye7p. Intriguingly, binding and activation by Gal4p was carbon source-dependent and much stronger during growth on media containing fermentable sugars than on glycerol. Furthermore, Tye7p and Gal4p were responsible for the complete induction of the glycolytic genes under hypoxic growth conditions. Tye7p and Gal4p also regulated unique sets of carbohydrate metabolic genes; Tye7p bound and activated genes involved in trehalose, glycogen, and glycerol metabolism, while Gal4p regulated the pyruvate dehydrogenase complex. This suggests that Tye7p represents the key transcriptional regulator of carbohydrate metabolism in *C. albicans* and Gal4p provides a carbon source-dependent fine-tuning of gene expression while regulating the metabolic flux between respiration and fermentation pathways.

## Author Summary

Pathogens must be able to assimilate the carbon sources in their environment to generate sufficient energy and metabolites to survive. Since glycolysis is a central metabolic pathway, it is important for this metabolic flexibility. The most commonly isolated agent in human fungal infections, *Candida albicans*, depends upon glycolysis for the progression of systemic disease. We investigated glycolytic transcriptional regulation in *C. albicans* and defined two key regulators of the pathway, Tye7p and Gal4p. We demonstrated that these factors are important for the fermentative growth of *C. albicans* both *in vitro* and *in vivo* and also regulate the input and output fluxes of glycolysis. The *gal4tye7* strain showed attenuated virulence in a *Galleria* and two mouse models, potentially due to the severe growth defect in oxygen-limiting environments. Gal4p and Tye7p represent fungal specific regulators involved in the pathogenicity of the organism that may be exploited in the development of antifungal treatments. Our study describes a fungal glycolytic transcriptional circuit that is fundamentally different from that of the model yeast *Saccharomyces cerevisiae*, providing further evidence that the transcriptional networks in *S. cerevisiae* need not be generally representative of the fungal kingdom.

## Introduction

**Figure 1:**
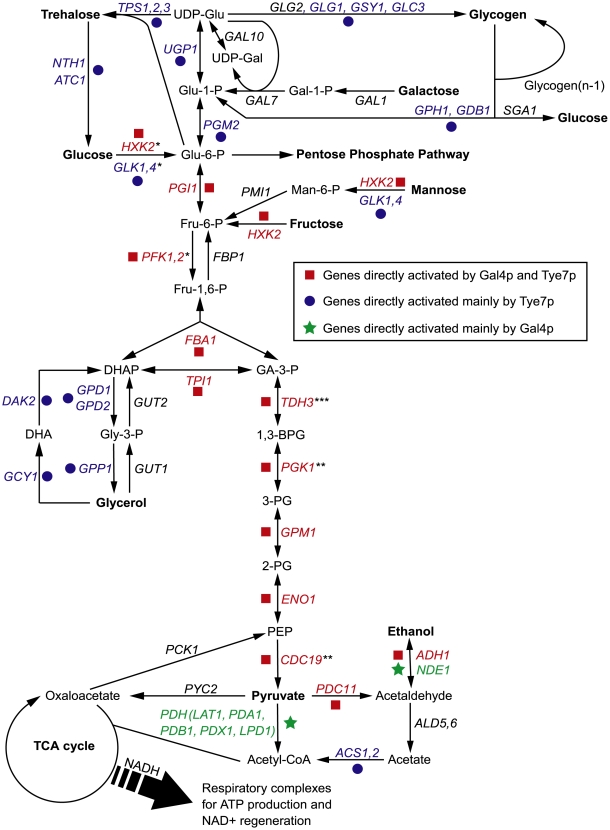
An overview of the central metabolic pathways in yeast. The enzyme names are for *C. albicans* but some have not been directly characterized and are annotated based on *S. cerevisiae* homology. Genes bound and activated by Gal4p and Tye7p are in red, genes bound and activated mainly by Tye7p are in blue, and genes bound and activated mainly by Gal4p are in green. Genes in black are not bound and activated by either factor. For simplicity, “*” represents reactions requiring ATP, “**” represents reactions producing ATP, and “***” represents reactions generating NADH. These symbols are given for reactions of the glycolytic pathway only.

In order to grow and thrive in a wide range of hosts, pathogens not only depend on certain virulence factors but also metabolic flexibility; therefore, they must be able to assimilate various carbon sources. Carbohydrates are the primary and preferred source of metabolic carbon for most organisms, and are used for generating energy and producing biomolecules. Most sugars are converted to glucose 6-phosphate or fructose 6-phosphate before entering the glycolytic pathway. Glycolysis is then responsible for converting these hexose phosphates into the key metabolite pyruvate while producing ATP and NADH (Figure 1). From there, cells carry out two major strategies of energy production: fermentation and respiration. Although both processes regenerate NAD+, respiration is significantly more energetically efficient than fermentation as it produces additional ATP through the tricarboxylic acid (TCA) cycle and oxidative phosphorylation. However, regardless of the mode of energy production, glycolysis is the central, common pathway for both processes. As glycolysis is critical for carbon assimilation, the pathway has been shown to be up-regulated during infections and important to the virulence in pathogenic bacteria, parasites, and fungi [1]–[6].

Since glycolysis is a central metabolic pathway, it is strictly regulated. While there are different levels of regulation of the process, transcriptional control is common to bacteria, fungi, plants, and animals. The glycolytic enzymes are transcriptionally regulated in response to environmental conditions such as oxygen levels, carbon source and availability, and to cellular demands such as metabolite concentrations and energy needs. However, in most species the regulators of glycolytic gene expression have not been identified, so our understanding of transcriptional control of glycolysis in eukaryotes is mainly based on the non-pathogenic yeast *Saccharomyces cerevisiae* (for review see [7]). In *S. cerevisiae*, the transcription regulators Gcr1p and Gcr2p are primarily responsible for activating the expression of the glycolytic genes [8],[9]. Gcr1p binds to CT boxes (5′-CTTCC-3′) upstream of the glycolytic genes and Gcr2p acts as a co-activator by forming a complex with Gcr1p [10],[11]. Deleting either gene decreases the expression levels of the glycolytic genes resulting in growth defects during culture on glucose [9],[12]. However, the mutant strains display wild type growth rates on non-fermentable carbon sources [9],[12]. The factor Tye7p (also referred to as Sgc1p) is another glycolysis-specific regulator in *S. cerevisiae*. Tye7p has been shown to be involved in the activation of several glycolytic genes, although not to the same extent as Gcr1p and Gcr2p [13]. This activation is independent of *GCR1* and the *tye7* strain displays no growth defects under any carbon source regime [13],[14]. The transcription factors Rap1p, Abf1p, and Reb1p also have roles in activating the glycolytic genes, but these are global factors involved in many cellular processes [15]–[18].

Although the glycolytic circuit is well characterized in *S. cerevisiae*, most organisms to do not have *GCR1* or *GCR2* homologs [19]. Furthermore, it is well established that the *Saccharomyces*-lineage exhibits a unique dependence on the fermentation pathway: these yeasts mainly ferment sugars to ethanol instead of using respiration, even under aerobic conditions [20]. *S. cerevisiae* up-regulates glycolysis and represses the TCA cycle in the presence of glucose allowing this aerobic fermentation behavior to occur [21]. Only when no fermentable carbon sources are present, after the post-diauxic shift, will *S. cerevisiae* switch to the respiratory mode. This phenomenon is known as the Crabtree effect and is due to a glucose repression circuit that is largely regulated by the transcriptional repressors Mig1p and Rgt1p, the protein kinase Snf1p, and the protein complex SCF^Grr1^ [20]. This regulatory circuit is proposed to have developed from the adaptive potential derived from the whole-genome duplication that occurred after the divergence of *Saccharomyces* from *Kluyveromyces* [22],[23]; the repression circuit is common to the *Saccharomyces*-lineage and many of the genes retained from the whole-genome duplication are involved in the lifestyle adaptation to aerobic ethanol production [24]–[26].

The facultative anaerobic lifestyle of Crabtree-positive *Saccharomyces* yeasts is in contrast to that of most other eukaryotes, which are either facultative or obligate aerobes and lack the glucose repression circuit. Under aerobic conditions, Crabtree-negative cells predominately oxidize pyruvate to carbon dioxide through the TCA cycle. In the absence of oxygen, most aerobic organisms are able to utilize the fermentation pathway to some extent to continue regenerating NAD+. This difference in metabolic flux is highlighted by transcription profiles of the aerobic fungi *Trichoderma reesei*, *Neurospora crassa*, and *Aspergillus oryzae*, which show little or no repression of the TCA cycle in glucose rich compared to glucose poor growth conditions, and therefore do not rely as heavily on the fermentation pathway as does *S. cerevisiae* [27]–[29].

The opportunistic human fungal pathogen *Candida albicans* is a facultative aerobe and thus metabolizes carbon sources in response to oxygen availability similar to that of a typical eukaryotic cell. *C. albicans* is responsible for various non life-threatening infections such as oral thrush and vaginitis but in extreme cases, especially in immunosuppressed individuals, it can cause potentially lethal systemic infections. In fact, *Candida* species are the most common isolated agent in fungal infections and the fourth leading cause of nosocomial bloodstream infections in the United States, with an attributable mortality rate of approximately 38–49% and treatment costs estimated to be $1.7 billion annually [30]–[33]. *C. albicans* accounts for more than half of all *Candida* infections [30],[33], highlighting the importance of understanding the metabolism of this pathogen for the development of effective antifungal treatments.

Crabtree-negative organisms that lack *GCR1/2* homologs, such as *C. albicans*, must control transcription of glycolytic genes differently than does *S. cerevisiae*. In this study, we characterized two fungal-specific activators of the glycolytic pathway in *C. albicans*, Tye7p and Gal4p. Deleting both genes resulted in severe growth defects when the mutant cells were cultured on fermentable carbon sources when respiration was inhibited or oxygen was limited, and chromatin immunoprecipitation coupled with microarray analysis (ChIP-CHIP) and transcription profiling showed these factors bind to and regulate expression of the glycolytic pathway genes. Tye7p and Gal4p are also required for complete pathogenicity as the mutant strains showed attenuated virulence. This work therefore defines the key regulatory elements controlling glycolytic gene expression in a facultative aerobic pathogen.

## Results

### Deletion of *CaGAL4* and *CaTYE7* results in a severe growth defect on fermentable carbon sources when the respiration pathway is disrupted

We investigated possible transcriptional regulators of glycolytic gene expression in *C. albicans*. In the well-studied yeast *S. cerevisiae*, Gcr1p, Gcr2p, and Tye7p are key glycolysis-specific activators. Unlike Gcr1p and Gcr2p, which are limited to *Saccharomyces* and closely related yeasts, Tye7-like transcription factors can be found throughout the Saccharomycotina subphylum. Therefore, while there are no homologs of Gcr1p or Gcr2p in *C. albicans*, there is a CaTye7p. ScTye7p and CaTye7p share 87% amino acid similarity in the DNA binding basic-helix-loop-helix (bHLH) domain but only 33% in the activation domain. A recent investigation also implicated the CaGal4p transcription regulator in the expression of genes involved in glycolysis [34]; while Gal4p in *S. cerevisiae* is a well-characterized zinc cluster transcription factor that regulates galactose catabolism, it does not fulfill this role in *C. albicans*. ScGal4p and CaGal4p also share homology strictly in the DNA-binding domain. Due to the potential or observed involvement of these factors in aspects of carbohydrate metabolism, we tested the role of CaTye7p and CaGal4p in the control of glycolytic gene expression in *C. albicans*.

**Figure 2:**
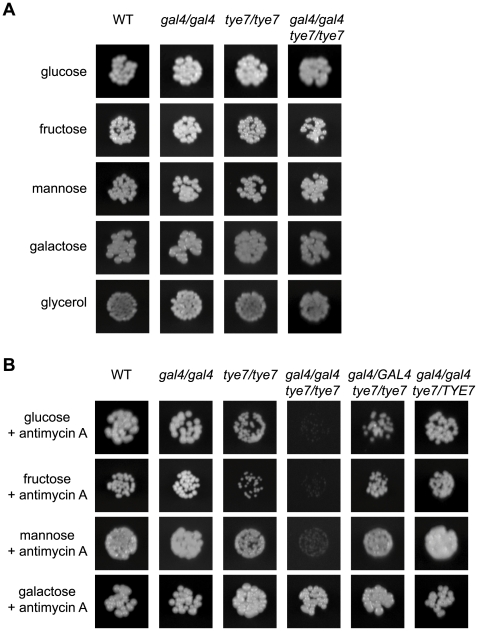
*GAL4* and *TYE7* are involved in fermentative growth with glucose, fructose or mannose as the sole carbon source. WT refers to strain CMM1. Cells were serially diluted and a representative dilution is displayed. Pictures were taken from plates where the carbon source was at 2% although the 0.2% plates gave similar results. (A) Solid media without antimycin A. Pictures were taken after 2–3 days of growth. (B) Solid media with antimycin A (2 µg/ml). Pictures were taken after 2–3 days of growth except for galactose (6 days).

We first constructed *tye7* and *gal4tye7* deletion strains to use in conjunction with our previously generated *gal4* strain [34]. We tested the ability of all strains to grow on the fermentable carbon sources glucose, fructose, mannose, and galactose, and the non-fermentable carbon source glycerol. On solid media, no growth defect was evident for any deletion strain regardless of the carbon source or concentration tested (Figure 2A). However, when the more sensitive liquid assay was used with glucose, galactose, and glycerol carbon sources, it was able to identify a minor growth defect for the *gal4tye7* strain with glucose media, as the doubling time was 147 min compared to that of the wild type of 123 min (Table 1 and Figure S1A). As *C. albicans* lacks the glucose repression mechanism that exists in *S. cerevisiae*, its respiration pathway is active under glucose growth conditions so it is not critically dependent on the glycolytic pathway. Although glycolysis is central to both the respiration and fermentation pathways, it is more important for fermentative metabolism since under these conditions the cell must rely exclusively on the ATP generated by glycolysis. As a result, glycolysis proceeds at a higher rate in fermenting cells [20].

**Table 1:** Doubling times under glucose growth conditions.

Strain	Aeration (min)	Static (min)
WT	123	139
*tye7/tye7*	121	155
*gal4/gal4*	116	132
*gal4/gal4/tye7/tye7*	147	210
*gal4/GAL4/tye7/tye7*	126	163
*gal4/gal4/tye7/TYE7*	129	138

Doubling times of the strains for the glucose growth curves in Figure S1. The natural log of the OD_600_ was plotted versus time and the best-fit line of the exponential phase was determined. The doubling time was equal to ln2/slope.

To mimic the fermentative metabolism of *S. cerevisiae*, the mitochondrial inhibitor antimycin A was added to the solid media, and cells in liquid culture were grown without aeration. These changes disrupt the proton gradient and ultimately prevent the production of ATP by oxidative phosphorylation via the respiration pathway. When *C. albicans* was forced to use fermentation, a severe growth defect during culture on glucose, fructose or mannose media was evident for the double mutant strain (Figures 2B and S1B and Table 1). Therefore, it appears that both Gal4p and Tye7p are involved in fermentative growth with most fermentable carbon sources. The *tye7* strain showed a minor growth defect under these fermentative conditions while the *gal4* strain still grew at wild type levels, suggesting that Tye7p is a more important regulator of fermentative growth. This prediction was supported by the complemented strains, as reintroducing one copy of *TYE7* was sufficient to restore wild type growth rates to the double mutant strain, while one copy of *GAL4* resulted in only partial restoration (Figure S1B). Therefore, although both factors appear to be involved in regulating the fermentative growth pathway, Tye7p plays a more central role.

Galactose was unique among the fermentable carbon sources tested as no distinct phenotype was observed for the *gal4tye7* strain compared to the wild type under fermentative growth conditions (Figures 2B and S1B). This is likely due to the Kluyver effect, which is thought to be a result of insufficient sugar uptake, and prevents the growth on certain sugars in the absence of respiration [35]. The fermentative growth conditions used (growth without aeration and antimycin A at 2 µg/ml) do not completely inhibit respiration, which allowed the strains to grow, although very slowly, in galactose media. Most yeast hexose transporters are able to take up glucose, fructose, and mannose, while galactose uptake requires separate transporters. If galactose uptake is the limiting step, then any effect of *GAL4* or *TYE7* on the fermentation pathway will be minimized. Therefore, the lack of observed difference between the mutant strain and the wild type in Figures 2B and S1B is not because the *gal4tye7* strain grows well on galactose media when respiration is disrupted, but instead is a result of the comparably poor growth of the wild type strain (Table S1).

### Location profiling reveals that Gal4p and Tye7p bind many carbohydrate metabolic promoter targets

**Figure 3:**
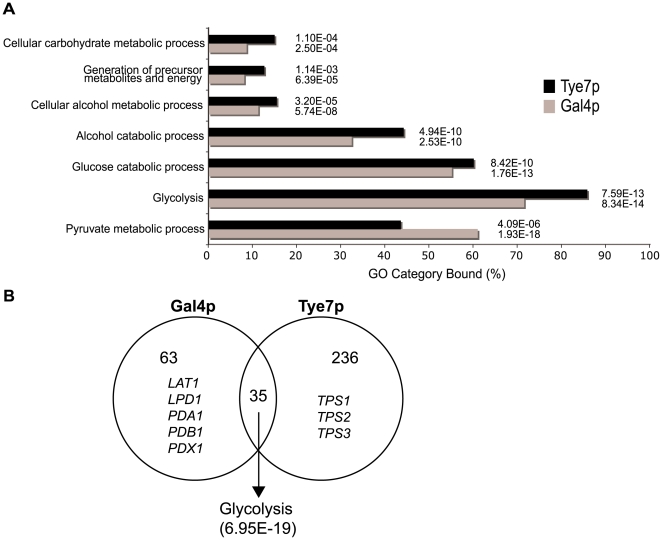
Gal4p and Tye7p bind many carbohydrate metabolic promoter targets under glucose growth conditions. (A) GO enrichment of YPD binding targets for Gal4p and Tye7p. Targets with a normalized fold enrichment >1.5 and a *P*-value<0.1 with the single spot full-genome microarray (98 and 271 genes for Gal4p and Tye7p, respectively) were analyzed with the CGD GO Term Finder (http://www.candidagenome.org/cgi-bin/GO/goTermFinder). The *P*-value of enrichment for each GO category is indicated. (B) Overlap of YPD binding targets from (A). The common targets are highly enriched for glycolysis genes. The Gal4p independent targets include the five components of the PDH while the Tye7p independent targets contain the three components of the trehalose synthase complex.

To gain insight into why Gal4p and Tye7p are required for fermentative growth, we performed ChIP-CHIP to determine their binding targets. ChIP-CHIP of chromosomally TAP-tagged Gal4p and Tye7p was first performed during growth in glucose media since glucose is the primary carbon source that stimulates the glycolytic pathway. Two different microarray formats, single spot full-genome arrays and whole-genome tiling arrays, were used to provide different strategies for data analysis and for validation purposes. Gal4p bound 98 targets and Tye7p bound 271 targets with the single probe full-genome array (Tables S2 and S3), so Tye7p appears to function as a more global regulator. However, both gene sets were significantly enriched for glucose/carbohydrate metabolic processes and both factors bound essentially all the glycolytic genes. The Gene Ontology (GO) biological processes that were enriched in the bound-gene sets are displayed in Figure 3A. The results are similar between the two factors except, as is discussed below, Gal4p bound more targets involved in pyruvate metabolism. Although Tye7p bound a large number of targets, no GO process is enriched other than carbon metabolism.

The tiling array showed highly similar results but was able to identify a few additional targets including three glycolytic genes, *PFK26-2*, *GLK1*, and *GLK4*. Smoothed peak intensity curves of the tiling array binding events were created to estimate the largest fold enrichments and thus the most significant targets. For Gal4p, the 13 *bona fide* glycolytic pathway promoters are in the top 68 smoothed peak intensities while for Tye7p they are in the top 52 peaks (Tables S4 and S5). Therefore, the glycolytic promoters are among the most significant targets for both factors. As well, several genes involved in ethanol fermentation (*PDC11*, *ADH1*, and *NDE1*) are included in this group of targets. Therefore, the ChIP-CHIP data suggests that Gal4p and Tye7p are involved in the entire fermentation pathway from glucose to ethanol.

**Figure 4:**
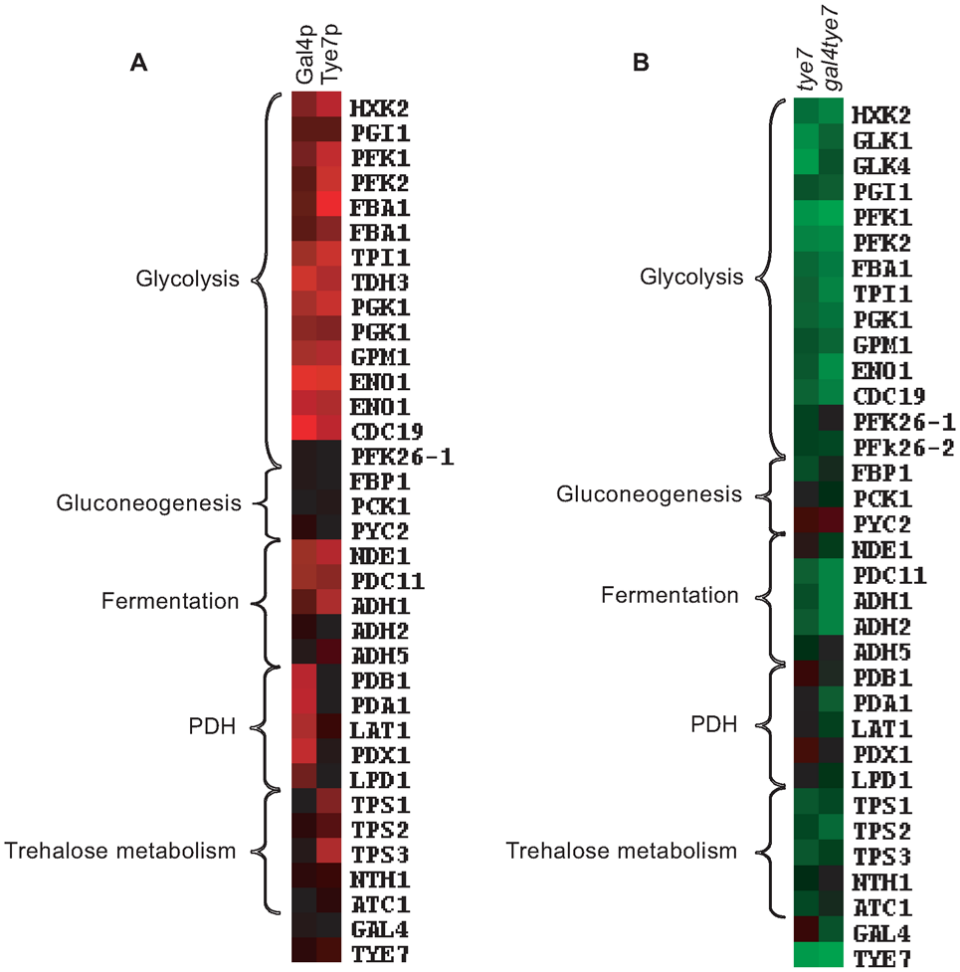
Gal4p and Tye7p bind to and regulate the expression of genes involved in the fermentation pathway under glucose growth conditions. Heat map displays of the ChIP-CHIP and expression profiles were created with the Cluster and TreeView programs (http://rana.lbl.gov/EisenSoftware.htm). (A) Tye7p and Gal4p ChIP-CHIP binding profiles of selected metabolic targets with the single spot full-genome microarray. Fold enrichments displayed represent the intergenic probe for the respective gene and enrichments <1.0 were adjusted to 1.0 for graphical purposes. Black represents no binding while red represents binding with the color brightness indicating the degree of enrichment. Binding to the glycolytic genes was so strong that binding to two neighboring probes was sometimes observed. In such cases, both values were included as these occurred in situations where a shared promoter region contained two probes but the tiling array confirmed the presence of a single binding site. The fold enrichments of these and other metabolic targets are given in Table S6. (B) Transcription profile of selected metabolic genes for the *tye7* and *gal4tye7* strains. Black represents no change in expression, green is down-regulated, and red is up-regulated with the color brightness indicating the degree of expression change. The expression levels of these and other metabolic genes are given in Table S7.

Although Gal4p and Tye7p bound many common targets, there were a significant number of individual binding events, some of which are related to carbohydrate metabolism (Figure 3B). The clearest example is that Gal4p bound the promoter sequences of the five genes encoding the pyruvate dehydrogenase complex (PDH) while Tye7p bound the promoter sequences of the three genes for the trehalose synthase complex. Tye7p also bound several genes involved in glycogen and glycerol metabolism. A summary of selected metabolic binding targets under glucose growth conditions is shown in Figure 4A and Table S6. Many of the Gal4p and Tye7p targets are linked to the glycolytic pathway suggesting that these factors also regulate the input and output fluxes of the pathway.

### Both Gal4p and Tye7p activate genes required for fermentative growth

ChIP-CHIP is a whole-genome approach for determining binding locations of a transcription factor; however, it is insufficient to give a complete picture of a factor's biological function. As was observed with *GAL4*, the binding and transcription profiles can provide different insights. Therefore, to complement the ChIP-CHIP analysis, transcription profiling comparing wild type and mutant strains was performed under glucose growth conditions. The expression levels of selected carbohydrate metabolic genes is illustrated in Figure 4B and Table S7. As expected, not all targets bound by ChIP-CHIP showed differential expression and not all differentially regulated genes showed direct binding of the transcription factors; however, in general the most significantly bound targets were down-regulated in the absence of the factor. The glycolytic and fermentation genes were down-regulated in the *tye7* strain confirming Tye7p's role as an activator of fermentative metabolism. Gal4p is also involved in the activation of the glycolytic/fermentation genes because the expression levels of these genes were lower in the *gal4tye7* strain compared to the *tye7* strain. The involvement of Gal4p in the activation of glycolytic gene expression was masked in the *gal4* expression profiles, most likely because the absence of *GAL4* caused an up-regulation of *TYE7* [34]. Therefore, Tye7p appears able to significantly compensate for the loss of Gal4p, further supporting the idea that it plays a more central role in glycolytic gene regulation than does Gal4p.

The ChIP-CHIP data showed that only Tye7p bound the genes involved in trehalose metabolism. Expression of the trehalose metabolic genes was down-regulated in the *tye7* strain but not further reduced in the *gal4tye7* strain indicating that Gal4p is not involved in their activation. Trehalose is a glucose disaccharide that has a role as a storage carbohydrate in yeast. Another important storage molecule in yeast is glycogen. Tye7p also bound several glycogen metabolic targets (*GPH1*, *GDB1*, *GLG1*, *GSY1*, and *GLC3*) that were subsequently down-regulated in the *tye7* strain and showed no influence of Gal4p in the *gal4tye7* strain. As well, Tye7p was the sole regulator of several glycerol metabolic targets (*DAK2*, *GCY1*, *GPP1*, *GPD1*, and *GPD2*). On the other hand, Gal4p activated the PDH genes with no influence from Tye7p. These genes were not down-regulated in the *tye7* strain (some were slightly up-regulated along with *GAL4*) but were reduced in the *gal4tye7* strain.

The down-regulated genes were analyzed for GO enrichment. As expected, all the categories were related to carbon metabolism. Generally, the two deletion strains had similar results with glycolysis (*tye7 P*-value: 1.34E-18; *gal4tye7*: 1.44E-15) and cellular alcohol metabolic process (*tye7*: 4.48E-17; *gal4tye7*: 8.33E-16) being the most significantly enriched categories.

### Tye7p regulates the cell's commitment to glycolysis

**Figure 5:**
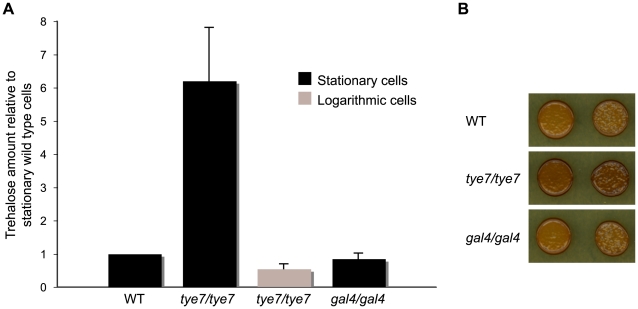
Deletion of *TYE7* results in higher trehalose and glycogen levels. (A) The trehalose content of wild type (BWP17), *tye7*, and *gal4* cells at both stationary and logarithmic phases were measured. Trehalose levels (nmol trehalose per mg cell protein) were reported relative to the wild type in stationary phase. Both wild type and *gal4* cells had no detectable trehalose levels in the logarithmic phase and were not included in the graph. (B) Wild type (BWP17), *tye7*, and *gal4* cells were exposed to iodine vapor to indicate the glycogen content as iodine vapor stains cells brown upon reacting with glycogen. The *tye7* cells stained a darker brown than the wild type indicating that there is more glycogen present.

Tye7p directly activated many genes involved in trehalose and glycogen metabolism independently of Gal4p. As well, the genes encoding the glycolytic-committing enzyme phosphofructokinase (*PFK1* and *PFK2*) were among the top six most down-regulated genes in the *tye7* strain while the gluconeogenesis-specific gene *FBP1* was moderately down-regulated. Therefore, it appears that Tye7p regulates the flux between energy storage and energy production at the glucose-6-phosphate branch point (Figure 1). To support this claim, we investigated whether the levels of trehalose and glycogen were different in the *tye7* strain compared to the wild type. Since exponentially growing cells have low trehalose levels that rapidly accumulate during stationary phase [36], trehalose amounts were determined from cells at both phases. We observed significantly increased levels of trehalose in the *tye7* strain for both stationary and logarithmic phase cells (Figure 5A). Additionally, iodine staining showed that the glycogen content in the *tye7* cells was higher than that of the wild type (Figure 5B). The higher storage carbohydrate levels correlate with the expression profile as *FBP1* and the majority of genes involved in trehalose and glycogen metabolism were down-regulated 2–3 fold while *PFK1* and *PFK2* were down-regulated approximately 6 and 9 fold, respectively. Therefore, the glucose-6-phosphate flux in the *tye7* strain would favor trehalose and glycogen synthesis. In contrast, the *gal4* strain showed wild type levels of both storage carbohydrates, suggesting that Tye7p alone regulates the cell's decision to commit to glycolysis or energy storage.

### Tye7p binds its targets independently of the carbon source while Gal4p displays differential binding

**Figure 6:**
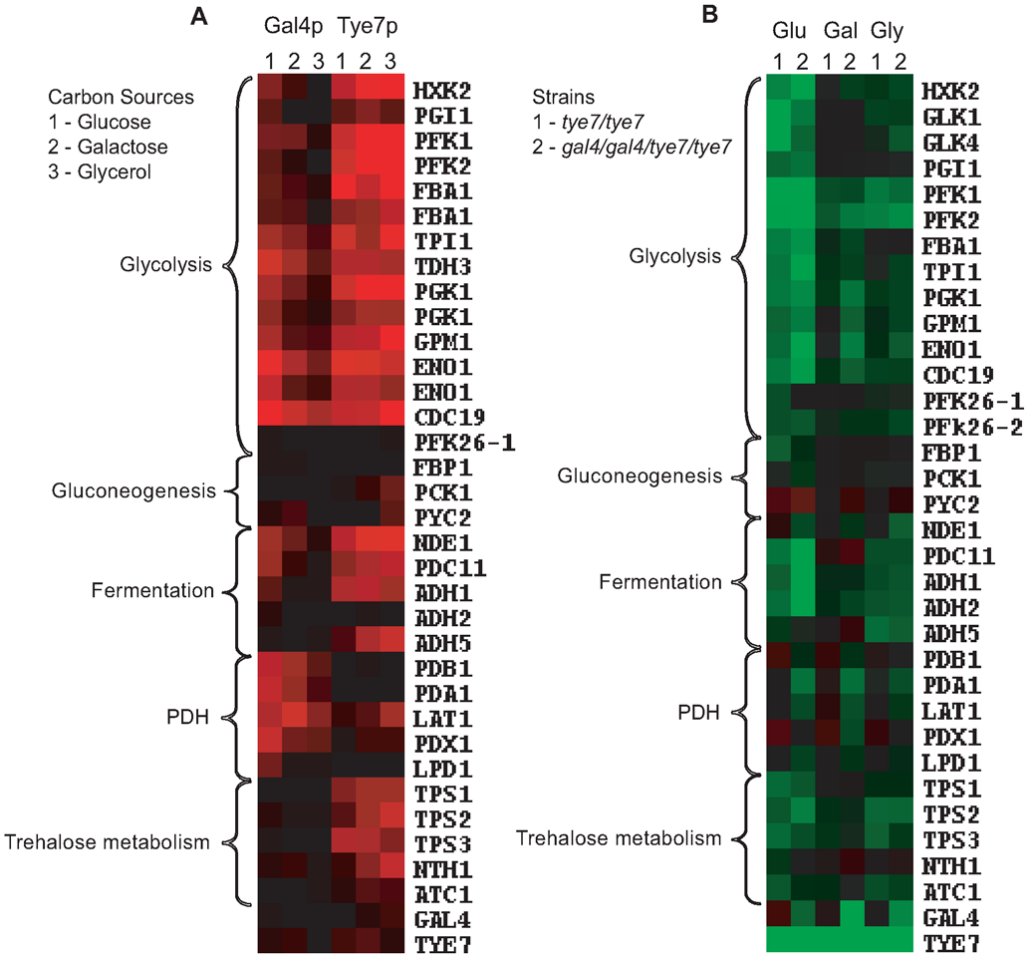
Comparison of ChIP-CHIP and expression profiles with glucose, galactose, and glycerol media. Heat map displays were created as described in Figure 4. (A) Tye7p and Gal4p ChIP-CHIP binding profiles of selected metabolic targets with the single probe full-genome microarray. The fold enrichments of these and other metabolic targets are given in Table S6. (B) Transcription profiles of selected metabolic genes for *tye7* (1) and *gal4tye7* (2) strains under glucose (Glu), galactose (Gal), and glycerol (Gly) carbon sources. The expression levels of these and other metabolic genes are given in Table S7.

It is clear that Gal4p and Tye7p are important for fermentative growth when glucose, fructose or mannose is the carbon source. Although there was no phenotype during growth on galactose or glycerol, we investigated the effect of these carbon sources on binding to identify any differences (Dataset S1). Figure 6A illustrates the ChIP-CHIP results of selected carbohydrate metabolic targets during growth on galactose and glycerol media with the behavior during growth on glucose included as a comparison.

**Figure 7:**
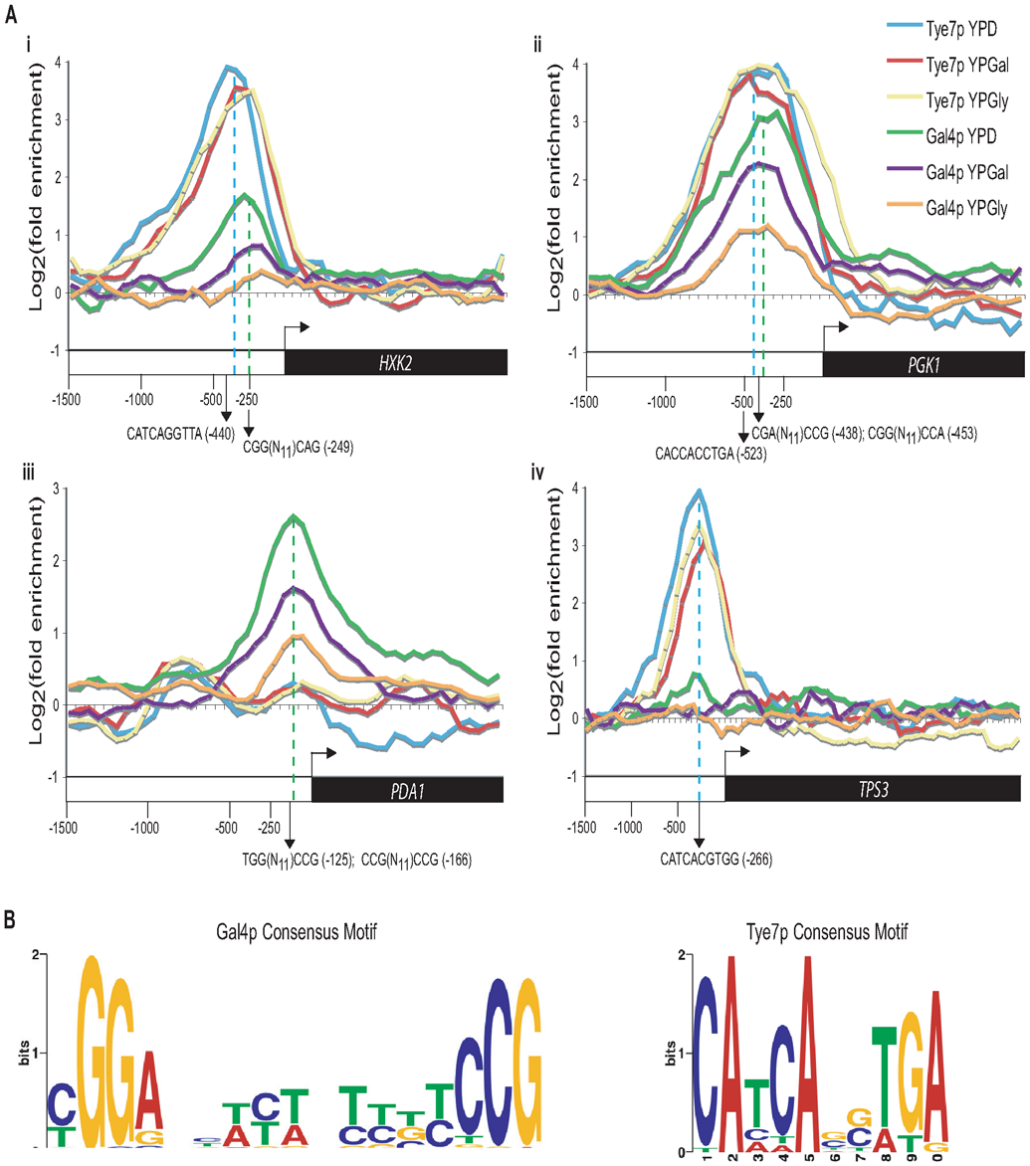
Gal4p displays carbon source-dependent binding while Tye7p binds its targets constitutively. (A) Binding of Gal4p and Tye7p to *HXK2* (i), *PGK1* (ii), *PDA1* (iii), and *TPS3* (iv) under glucose, galactose, and glycerol growth conditions with the tiling array. *HXK2* and *PGK1* represent common glycolytic targets while *PDA1* and *TPS3* represent Gal4p and Tye7p specific targets, respectively. The ORF and 1500 bp upstream region is shown. Values for each data point were determined by taking the mean fold enrichment of each probe and the surrounding four probes. The dashed blue and green lines represent the Tye7p and Gal4p binding sites, respectively (approximated over the three carbon sources). The consensus motifs and their positions are indicated for each factor. (B) Consensus motifs based on the top 30 peak intensities from the tiling array for Gal4p and Tye7p on glucose media. The sequence surrounding the peak point (covering 5 probes, 300 bp) for each target was sent to MEME (http://meme.sdsc.edu/meme4/) and the most significant motif (Gal4p: *E* = 9.3E-24; Tye7p: *E* = 1.8E-15) is reported.

A striking trend was the difference in binding between Gal4p and Tye7p under the various carbon sources. Whereas the peak intensity of Gal4p binding changed based on the carbon source, the peak sizes of Tye7p binding were largely unaffected (Figure 7A). This pattern was consistent for the majority of targets resulting in a decrease in the overall number of Gal4p binding targets from glucose to galactose to glycerol and a similar overall number of binding sites for Tye7p with the different carbon sources (Figure S2). Therefore, Gal4p binds its few targets in a carbon source-dependent manner while Tye7p appears to be a more global regulator that binds its targets independently of the carbon source the cells are growing on. This difference in target binding dependent on the carbon source could be a direct result of the protein levels of the transcription factors. We compared protein levels during growth in YPD, YPGal, and YPGly media relative to YP media and observed that Gal4p is significantly induced by glucose while Tye7p has a more constitutive expression (Figure S3). These results further support the inference that Tye7p is the more central regulator of carbohydrate metabolism.

Another trend was that Gal4p showed stronger binding to the promoters of genes encoding the glycolytic pathway enzymes that acted in the later part of the pathway (from *TPI1* on) compared to the early part of the pathway, regardless of the carbon source of the growth medium (Figure 6A). In Figure 7A, *HXK2* binding is representative of early pathway genes and *PGK1* binding is representative of later pathway genes. Interestingly, this difference in binding within the pathway corresponds at the point where the six carbon glucose molecule has been converted to two three carbon products and also represents the separation between the initial ATP consuming steps and the later energy producing steps (Figure 1). Therefore, while Tye7p appears to be involved in committing the cell to glycolysis, Gal4p appears to focus on the later part of the pathway to promote energy production once the commitment is made.

### CaTye7p and CaGal4p bind to distinct motifs

The binding distribution curves created with the tiling array were also used to predict the motif that CaGal4p and CaTye7p recognize by looking for sequences enriched around the binding sites of the top peak intensity targets. ScGal4p has a well established 5′-CGG(N_11_)CCG-3′ motif [37]. Analysis of the top CaGal4p binding targets revealed enrichment for this motif (Figure 7B). Since ScGal4p and CaGal4p have 86% sequence similarity in the DNA binding domain, it is reasonable to expect they would recognize a similar sequence. The binding distribution curves of *HXK2*, *PGK1*, and *PDA1* showed Gal4p motifs near the binding sites of CaGal4p (Figure 7A).

As previously mentioned, CaTye7p is a bHLH transcription factor. These type of factors are known to recognize the E-box sequence 5′-CANNTG-3′ [38]. The motif enriched among the top CaTye7p binding targets contains this bHLH signature (Figure 7B). The binding distribution curves of *HXK2*, *PGK1*, and *TPS3* showed Tye7p motifs near the binding sites of CaTye7p (Figure 7A).

### Tye7p and Gal4p are carbon-source dependent activators

To gain further insight into how Gal4p and Tye7p regulate their targets in response to different carbon sources, transcription profiles comparing wild type and deletion strains with galactose and glycerol as the sole carbon source were performed. Figure 6B illustrates the expression profiles of selected carbohydrate metabolic targets during growth on galactose and glycerol media with the behavior during growth on glucose included as a comparison (complete lists of down-regulated genes in Tables S8, S9, S10, S11, S12 and S13). The glycolytic genes were down-regulated in the *tye7* strain under galactose and glycerol growth conditions but not as significantly as with glucose-containing media. Gal4p strongly activated the glycolytic genes on glucose and galactose media but had only minimal effect when glycerol was the carbon source. This result correlates with the location profiling data as Gal4p displayed reduced binding under glycerol growth conditions. Tye7p and Gal4p's carbon-source dependent roles in glycolytic gene expression were validated by quantitative real-time PCR (qPCR) (Figure S4).

### Gal4p and Tye7p are involved in glycolytic gene induction in hypoxic growth conditions

**Figure 8:**
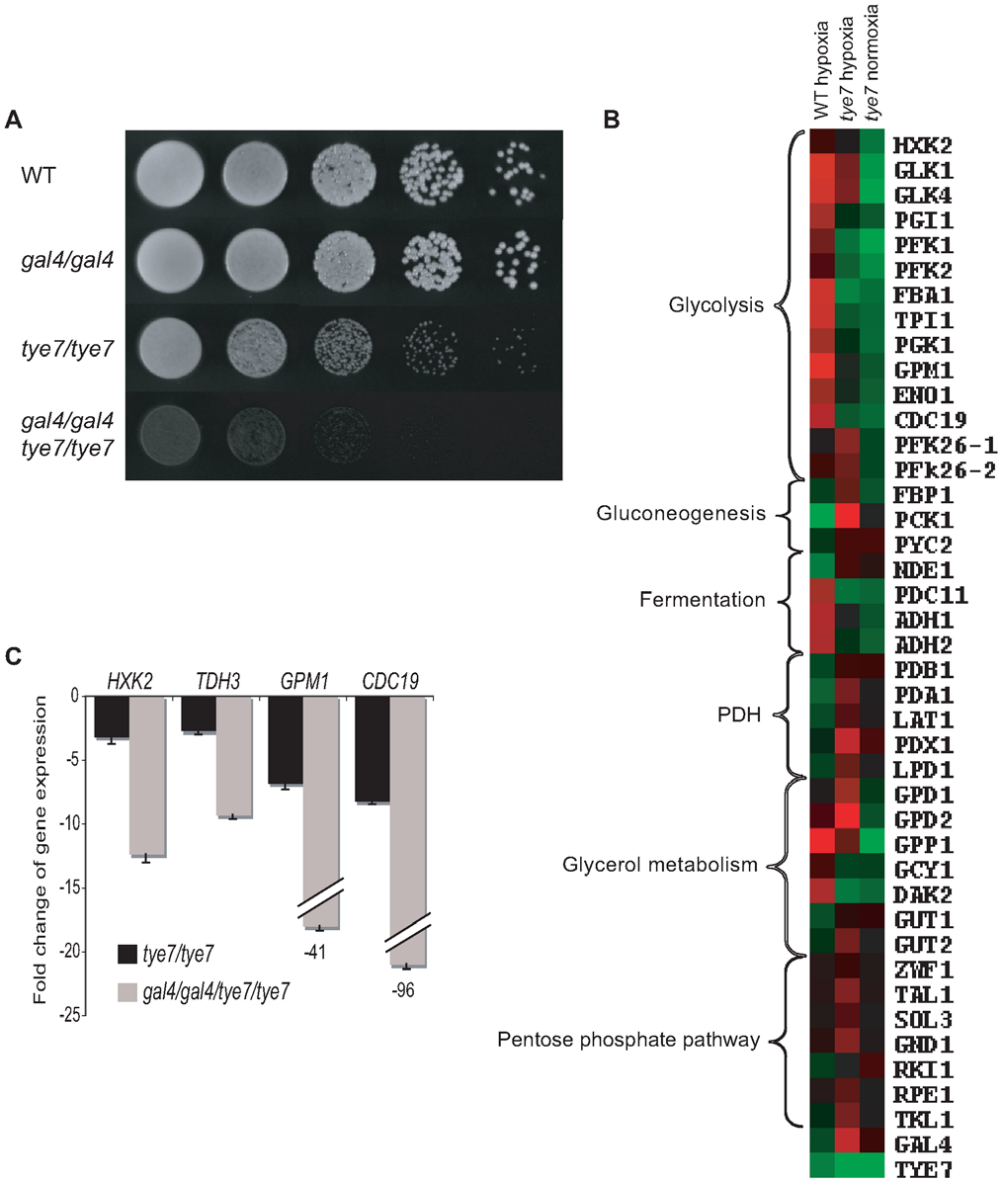
*TYE7* and *GAL4* are important for hypoxic growth and the induction of glycolytic genes in a low oxygen environment. (A) Cells were serially diluted, plated on YPD, and incubated in an anaerobic chamber at 30°C for 4 days. The chamber was flushed daily with nitrogen. Due to the space limitation of the chamber, the revertant strains were unable to be spotted on the same plate. A second experiment including the revertant strains was done on two separate plates to verify the results (Figure S5B). (B) Heat map displays were created as described in Figure 4. Transcription profiles of selected metabolic genes for (from left to right) BWP17 hypoxia relative to BWP17 normoxia, *tye7* hypoxia relative to BWP17 hypoxia, and *tye7* normoxia relative to BWP17 normoxia. The expression levels of these and other metabolic genes are given in Table S7. (C) The expression levels of several glycolytic genes were measured by qPCR in the *tye7* and *gal4tye7* strains relative to the wild type (BWP17) after 30 min of growth under hypoxic conditions. *ACT1* was used as the reference.

Pathogens must not only be adept at utilizing different carbon sources but must also be able to handle changes in oxygen levels. For *C. albicans*, this flexibility involves growth in oxygen rich environments such as the skin and oral mucosal layers and oxygen poor niches such as inner organs. Since serious systemic infections are associated with these oxygen poor conditions and the glycolytic genes are known to be up-regulated in response to hypoxia in *C. albicans* [39], we tested the ability of our deletion strains to grow in oxygen-limiting environments. The *gal4* strain was unaffected but the *tye7* and *gal4tye7* strains displayed a severe growth defect (Figure 8A). This defect was observed not only at 30°C but also at 37°C, the human physiological temperature (Figure S5A). This is further validation for Gal4p and Tye7p's role in fermentative metabolism.

To confirm that Tye7p is responsible for the induction of the glycolytic genes under hypoxic conditions we repeated the expression profile under glucose growth conditions in the presence of nitrogen instead of oxygen (for complete lists see Tables S14 and S15). As observed under oxygen rich (normoxic) growth conditions, the glycolytic genes were down-regulated (Figure 8B and Table S7). However, there were some differences compared to the normoxia profile. First, some metabolic genes altered their expression to adjust to the low oxygen environment in the absence of the key glycolytic activator. These changes include the up-regulation of gluconeogenesis-specific genes, glycerol synthesis genes, pentose phosphate pathway genes, and the PDH genes, all of which were either down-regulated or not significantly regulated in the normoxia profile. The lack of a fully functional glycolytic pathway would result in gluconeogenesis stimulation, glycerol synthesis is an alternative to ethanol fermentation to regenerate NAD+, the pentose phosphate pathway is an alternative to glycolysis to generate reducing equivalents, and increasing PDH expression would promote respiration for any of the available pyruvate. Second, *GAL4* expression was significantly up-regulated (5.5 fold compared to 1.4 fold in normoxic conditions). This up-regulation also explains the increase in PDH expression and is likely why some glycolytic genes were not as significantly down-regulated. Third, the expression of many more genes was altered in the hypoxia profile, but many of these could be attributed to the significant growth defect of the *tye7* strain (doubling time was approximately 220 min compared to the wild type at around 110 min). We chose to just focus on the effect on metabolic gene expression as we had already established Tye7p as a metabolic regulator under normoxic conditions.

Based on the reduced growth rate of the *gal4tye7* strain compared to the *tye7* strain in low oxygen growth conditions, we assumed that Gal4p was also involved in the induction of the glycolytic genes. The expression profile with the *gal4tye7* strain was not done due to the severe growth defect. However, to confirm our hypothesis we transferred normoxia grown cultures to low oxygen conditions for 30 min and analyzed glycolytic gene expression by qPCR. The *gal4tye7* strain showed a significant further down-regulation compared to the *tye7* strain (Figure 8C).

To ensure our hypoxia experimental set-up was accurate and that the glycolytic genes were induced, we compared the wild type strain under hypoxic and normoxic conditions (Figure 8B and Table S7). Our results agreed well with previously published data [39] as glycolytic, fermentation, stress response, cell wall, fatty acid, iron metabolism, and hyphae-specific genes were up-regulated while TCA cycle, respiration, and ATP-synthesis genes were down-regulated (for complete lists see Tables S16 and S17).

Surprisingly, *TYE7* and *GAL4* expression appeared down-regulated in the wild type under hypoxia. Recently, it has been shown that the glycolytic genes can be rapidly induced under hypoxia before subsequently declining [40]. This dynamic expression is especially true for transcription factors that must be quickly up-regulated to activate their target genes but may become down-regulated once their target genes have reached their needed expression levels. Therefore, we measured the levels of *TYE7*, *GAL4*, and the glycolytic gene *CDC19* at four different time points following a shift to hypoxic growth conditions (Figure S6). We observed a rapid increase in *TYE7* and *GAL4* expression in the first 15 minutes followed by a sharp decline. In contrast, *CDC19* induction was longer and the decline less drastic. Thus, *TYE7* and *GAL4* are initially induced by hypoxia to activate the glycolytic genes before they are subsequently down-regulated.

### Complete glycolytic activation by Gal4p and Tye7p is required for full virulence

**Figure 9:**
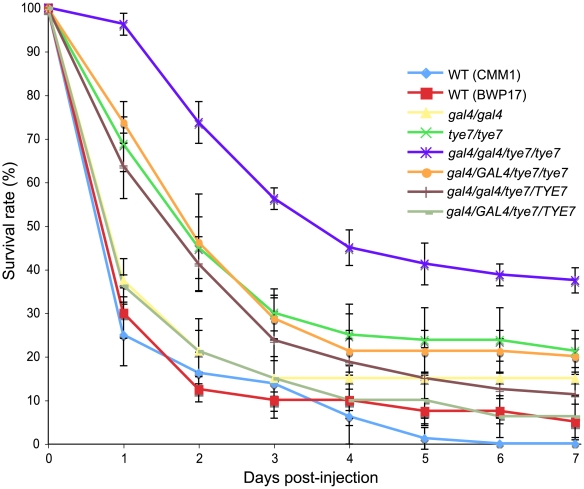
*GAL4* and *TYE7* are required for full virulence in a *Galleria* model. The survival curves for the indicated strains are shown. Each point represents the average daily survival rate based on four replicates of 20 insects. The BWP17 and CMM1 survival curves were not significantly different (*P*>0.05, log-rank test), demonstrating the limited effect of histidine and arginine auxotrophies.

Since metabolic flexibility and growth under low oxygen conditions are important for pathogens, we investigated the effect of deleting *GAL4* and *TYE7* on the virulence of *C. albicans*. We chose to first test all of our strains using the greater wax moth *Galleria mellonella* as a host model. Screening the virulence of *C. albicans* strains in *Galleria* has been shown to produce similar results to those measured through systemic infections with mice [41]. As controls, injections of PBS or UV/heat-killed BWP17 did not kill any *Galleria* over seven days, demonstrating that any death was attributable to viable *C. albicans* cells. The *gal4* strain showed a minor, but significant (*P* = 0.008, log-rank test) difference compared to the wild type, while both the *tye7* and *gal4tye7* strains showed very significant (*P*<0.0001, log-rank test) attenuated virulence (Figure 9). These results correlate with the observed growth defects under hypoxic conditions. If the mutant strains grow slower than the wild type due to a lower oxygen environment inside the insect, the insect is able to survive for a longer period of time.

**Figure 10:**
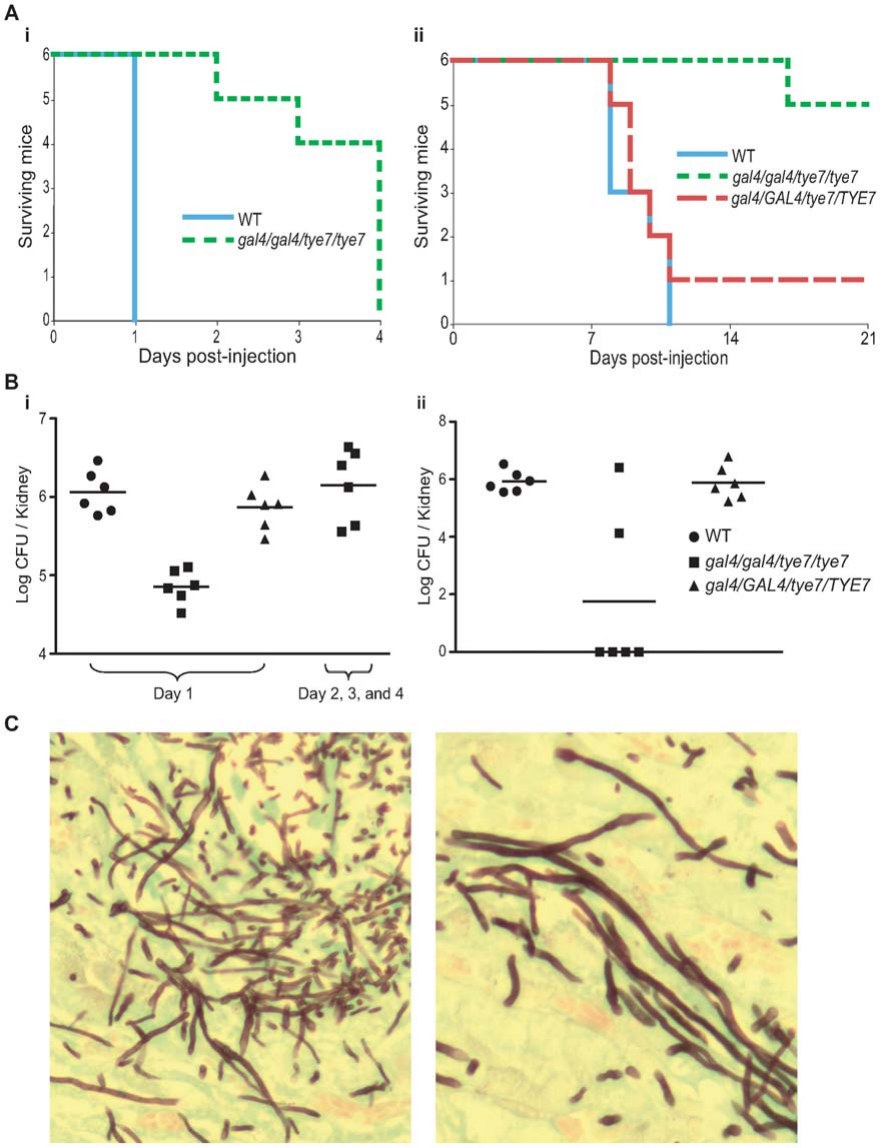
The *gal4tye7* strain shows attenuated virulence in both A/J and C57BL/6J mouse models. (A) A/J (i) and C57BL/6J (ii) mice were injected with either wild type (CAS8), *gal4/gal4/tye7/tye7* (CAS9), or *gal4/GAL4/tye7/TYE7* (CAS10) strains. A/J mice were monitored until considered moribund while C57BL/6J were monitored over a 21 day period. The revertant strain was omitted from the A/J graph since all six mice were euthanized on day 1 and would be masked by the wild type line. (B) (i) The kidney fungal load was determined 24 hours after injection for the A/J mice. Mice infected with the *gal4tye7* strain had approximately ten times less *C. albicans* cells than the mice challenged with either the wild type or revertant strains. Another fungal load of *gal4tye7* infected mice was determined upon moribundity and showed comparable levels to the mice injected with the wild type strain after 24 hours. (ii) The kidney fungal load was determined upon euthanization of C57BL/6J mice when moribundity was determined or mice survived until day 21. (C) Kidney sections of A/J infected mice were taken after euthanization and *C. albicans* cells were revealed with the Grocott-Gomori methenamine-silver stain. Pictures shown are from tissue infected with the *gal4tye7* strain indicating hyphal formation. There was no obvious difference compared to sections taken from kidneys infected with wild type or revertant strains (data not shown).

The double mutant strain was also tested in two mouse models, A/J and C57BL/6J, to support the result from the *Galleria* model and allow for further analysis. The A/J strain is C5 deficient and is highly sensitive to systemic infection with *C. albicans* while the C57BL/6J strain is C5 sufficient and therefore is less sensitive [42]. As with the *Galleria* model, the *gal4tye7* strain displayed significant attenuated virulence in both A/J (*P* = 0.0009, log-rank test) and C57BL/6J (*P* = 0.0007) mouse models (Figure 10A).

Fungal loads from different tissues were examined. For the A/J mice, two sets of six mice were injected with the *gal4tye7* strain. Fungal loads for the first set were determined 24 hours after injection, when the mice challenged with the wild type and revertant strains were euthanized due to moribundity. Fungal loads from the kidney, liver, and heart were significantly lower (*P* = 0.002, 0.002, and 0.009, respectively, Mann-Whitney test) in the *gal4tye7* infected mice compared to mice challenged with the wild type strain (Figure 10B and Table 2). The fungal load of the second set was determined when the *gal4tye7* infected mice became moribund. The fungal burden of this set showed comparable levels to the mice injected with the wild type strain (Figure 10B and Table 2). For the C57BL/6J mice, kidney fungal loads were determined following euthanization due to moribundity or survival until day 21. Five of the six C57BL/6J mice challenged with the *gal4tye7* strain survived until day 21 and four of them completely cleared the infection (Figure 10B).

**Table 2:** Fungal loads of selected tissues from A/J infected mice.

Strain	Mean burden (log CFU/organ)					
	Day 1			Day 2, 3, and 4		
	Kidney	Liver	Heart	Kidney	Liver	Heart
WT	6.1±0.3	4.2±0.2	4.0±0.5	N/A	N/A	N/A
*gal4/gal4/tye7/tye7*	4.9±0.2	3.5±0.3	2.7±0.6	6.1±0.5	4.0±0.3	4.0±0.4
*gal4/GAL4/tye7/TYE7*	5.9±0.3	3.9±0.3	3.7±0.5	N/A	N/A	N/A

Histological examination showed *in vivo* hyphae formation of the mutant strain in the kidney of A/J mice (Figure 10C) and confirmed the observations that the *gal4tye7* strain is able form hyphae in the presence of serum, N-acetylglucosamine, and spider media *in vitro* (data not shown). These results further support the hypothesis that the reduced virulence of the double mutant strain is attributed to a growth defect due to low oxygen environments in the host, which allows the organism to survive with the infection for a longer time and increases the chance the host's immune system is able to clear the infection.

## Discussion

Although fungi generally have similarly designed metabolic pathways, their transcriptional regulation of these pathways can be quite different, as illustrated by the human fungal pathogen *C. albicans* and the non-pathogenic yeast *S. cerevisiae*. Crabtree-positive yeasts, such as *S. cerevisiae*, have developed a circuit that represses the respiration pathway and up-regulates the glycolytic/fermentation pathway in the presence of excess glucose even under aerobic conditions. In contrast, *C. albicans* is Crabtree-negative and prefers to completely oxidize carbohydrates through the respiration pathway in aerobic conditions, only relying on the fermentation pathway in the absence of oxygen. Furthermore, *C. albicans* up-regulates the glycolytic pathway in low oxygen conditions while *S. cerevisiae* does not [39]. These metabolic responses of *C. albicans* are similar to the majority of fungi and other eukaryotes. Therefore, it is important to extend our understanding of transcriptional control of carbohydrate metabolism beyond *S. cerevisiae* to other organisms.

Although the glycolytic transcriptional circuit has been studied in the yeast *Kluyveromyces lactis* [19],[43], which lacks the glucose repression circuit, this organism is closely related to *S. cerevisiae* and its glycolytic genes are mainly regulated by orthologs of Gcr1p and Gcr2p. It appears that *K. lactis* is an intermediate between *S. cerevisiae* and the majority of aerobic fungi that do not contain *GCR1/2* homologs and therefore it is not a representative model of Crabtree-negative fungi. We characterized Tye7p and Gal4p, two transcriptional activators of the glycolytic pathway in *C. albicans*, which lacks *GCR1/2* homologs. Deleting both factors resulted in a severe growth defect during culture on several fermentable carbon sources (glucose, fructose, and mannose) when respiration was inhibited or oxygen was limited; the single mutant *gal4* strain showed no growth defects while the *tye7* strain displayed a slight growth defect under these conditions. All deletion strains grew at near wild type levels on a non-fermentable carbon source or with fermentable sources when the respiration pathway was not disrupted.

ChIP-CHIP and transcription profiling revealed that both Tye7p and Gal4p are directly involved in the activation of the entire glycolytic pathway. Tye7p bound all the glycolytic promoters in a carbon source-independent manner and the *tye7* strain showed a down-regulation of these genes that was most significant in glucose growth conditions. Gal4p binding to the promoter sequences of the glycolytic genes was affected by the carbon source with a decrease in binding from glucose to galactose to glycerol. Gal4p's role in the activation of these genes was also carbon source-dependent with the strongest effect during growth on glucose and galactose. Furthermore, transcription profiling and qPCR confirmed that both Tye7p and Gal4p are involved in the induction of the glycolytic genes under hypoxic growth conditions.

Gal4p and Tye7p also regulated metabolic processes linked to the glycolytic pathway. Some of these roles are common while some are independent of one another. Tye7p bound to and activated many genes involved in trehalose and glycogen metabolism without Gal4p's involvement. As well, Tye7p strongly activated the genes encoding phosphofructokinase, which catalyzes an irreversible glycolytic-committing reaction. We showed that deleting *TYE7* increases the levels of trehalose and glycogen, likely a result of the severely reduced glycolytic flux due to the extremely low expression of *PFK1* and *PFK2*. Deleting *GAL4* had no effect on the storage carbohydrate levels. Therefore, Tye7p has a role in determining whether glucose is stored or utilized for energy derivation, similar to Gcr1p in *S. cerevisiae* [44],[45]. Coordinately regulating this flux ensures that the cell is committed either to storing energy or producing energy as to avoid futile cycling; therefore, it is logical that the key glycolytic activator also regulates trehalose and glycogen metabolism.

Another equilibrium that requires regulation is the flux between fermentation and respiration. This flux depends on the competition for pyruvate between pyruvate decarboxylase (PDC) and the pyruvate dehydrogenase complex (PDH) [46]. In *S. cerevisiae*, glucose induces PDC expression to promote fermentation while the PDH genes are unaffected and the TCA cycle is repressed [21]. In *A. oryzae* and *N. crassa*, both the PDC and PDH are induced by glucose and the TCA cycle is not repressed [28],[29]. Increasing the transcription level of the PDH may be a mechanism employed by Crabtree-negative cells to allow the PDH to out-compete PDC, thereby increasing the respiratory capacity of the cell. Both Gal4p and Tye7p bound to and activated the genes involved in fermenting pyruvate to ethanol indicating that these factors regulate the entire fermentation process from glucose to ethanol. However, only Gal4p bound and activated the PDH genes suggesting that it plays an important role in the metabolic flux of the cell in directing respiration vs. fermentation modes. Consequently, Gal4p has an indirect effect on the TCA cycle. Although Gal4p did not bind the promoter sequences of the TCA cycle genes in the ChIP-CHIP profile (except *LSC1/2*), deleting *GAL4* subsequently results in the down-regulation of the PDH genes and ultimately several TCA cycle genes [34].

Therefore, although both Gal4p and Tye7p are key regulators of the glycolytic pathway, each has its own distinct role. Tye7p is the central transcriptional regulator of carbohydrate metabolism that provides a strong basal level of glycolytic expression while controlling the flux into the pathway and committing the cell to glycolysis. Gal4p is a carbon source-dependent regulator that fine-tunes gene expression based on the cells' need for the fermentation pathway. It assists Tye7p by increasing glycolytic gene expression during growth on fermentable carbon sources. In the presence of fermentable carbon sources Gal4p is able to significantly enhance the energy producing part of the glycolytic pathway and promote respiration by activating the PDH to meet the increased energy needs of the cell and minimize the dependence on fermentation. This assistance is not required with non-fermentable carbon sources as the glycolytic flux is lower since gluconeogenesis is stimulated and the cells grow at a slower rate with reduced energy needs.

The most common types of antifungal drugs for *C. albicans* infections, azoles, polyenes, and echinocandins, target cell membrane and cell wall integrity; however, targeting fungal metabolism could provide for future drug development. The importance of glycolysis to *C. albicans'* pathogenicity has been shown as deleting *CDC19* causes avirulence while a conditional mutant of *FBA1* results in attenuated virulence [3],[4]. Since the components of metabolic pathways are generally highly conserved, orthologs exist in humans reducing interest in these functions as potential drug targets (i.e. *C. albicans CDC19* has approximately 50% amino acid identity with human pyruvate kinases). However, Gal4p and Tye7p are fungal-specific regulators and therefore represent potential antifungal targets. *GAL4* and *TYE7* were shown to be important for the virulence in a *Galleria* and two mouse models. This virulence effect is likely a result of a growth defect due to the low oxygen conditions that are present in invasive infections. The ability of two-thirds of the *gal4tye7* injected C57BL/6J mice to clear the infection highlights the real potential of Gal4p and Tye7p as drug targets. Although the double mutant strain did show significant attenuated virulence in all three host models, it was not avirulent. This is likely attributed to the metabolic flexibility of *C. albicans* and its ability to use alternative carbon sources. The importance of alternative carbon metabolism to *C. albicans'* pathogenicity has been previously demonstrated as deleting key enzymes of the glyoxylate cycle, gluconeogenesis, and ß-oxidation pathways reduces virulence [3],[47],[48].

It appears that Gal4p and Tye7p have altered their function throughout the evolution of fungi. We analyzed other genomes in the Saccharomycotina subphylum and observed a pattern relating the presence of *GCR1/2* homologs and the clustering of the Gal4p motif. Species that possess *GCR1/2* homologs have an enrichment of the Gal4p motif upstream of the *GAL* regulon genes while species lacking *GCR1/2* homologs have an enrichment of the Gal4p motif in the promoter regions of the glycolytic genes. Therefore, it appears that the rewiring of Gal4p coincides with the loss/gain of *GCR1/2*, and that Gal4p and Tye7p likely regulate glycolysis in the Saccharomycotina species lacking *GCR1/2* homologs. Intriguingly, this rewiring event also appears to coincide with changes in the galactose sensory network [49].

Along with the ribosomal transcriptional network [50],[51], the transcriptional regulatory control of glycolysis represents an example of the plasticity of circuits throughout the evolution of fungi. The Rap1p ribosomal circuit and the Gcr1p/Gcr2p glycolytic circuit are unique to *S. cerevisiae* and closely related species, suggesting that the transcriptional networks of *S. cerevisiae* are not representative models of the fungal kingdom. However, there is a key distinction between these two rewiring events. While the regulatory components of the ribosomal network are different, the ultimate outcome is identical. On the other hand, carbon metabolism regulation is fundamentally different between *Saccharomyces* and other eukaryotes suggesting that the entire carbohydrate transcriptional network has undergone rewiring, not just the regulators. Thus, investigating the regulation of carbon metabolism in Crabtree-negative organisms is important as little can be extrapolated based on *S. cerevisiae*. This study thus defines the key regulatory elements of glycolytic gene expression and provides insights into the mode of transcriptional regulation of carbohydrate metabolism in a typical eukaryotic cell and a human pathogen.

## Materials and Methods

### *C. albicans* strains and media

The *C. albicans* strains used in this study are listed in Table S18. Cells were generally grown at 30°C in media containing 1% yeast extract, 2% peptone, with either 2% dextrose (YPD), 2% galactose (YPGal), or 2% glycerol (YPGly). All media was supplemented with uridine (50 µg/ml).

### Strain constructions

Plasmids and oligonucleotides used in this study are listed in Tables S19 and S20, respectively. Gal4p and Tye7p were tagged chromosomally with a TAP-*URA3* PCR product [52]. Transformations were carried out using standard procedures [53]. Correct integration of the TAP-tag was confirmed by PCR, and western blots were used to verify protein expression.

BWP17 was used for generating the *tye7* strain. CMM3 (*gal4*) [34] was used to generate the *gal4tye7* strain. The deletion and complementation strains were created using the *SAT1*-flipper cassette as described [54] with some modifications. The *tye7* disruption construct was created by cloning 500 bp flanking sequences of *TYE7* into the plasmid pSFS2A. The plasmid was linearized prior to transformation. Transformants were selected on YPD plates containing 200 µg/ml of nourseothricin and confirmed by PCR. Excision was performed by incubation at 30°C for 5 hours in YP media with 2% maltose before plating on YPD plates. Confirmation of excision events was done by PCR. The process was repeated for disruption of the second allele. Revertants of *tye7* and *gal4* were created using the *gal4tye7* strain. Complementation of *tye7* was carried out by reintroducing the ORF at its native locus by replacing the upstream flanking sequence in the *tye7* disruption cassette with the complete *TYE7* ORF. A complementation cassette that consisted of the complete *GAL4* ORF and a 500 bp downstream flanking sequence was constructed for reintroduction of *GAL4* at its native locus. We selected clones that replaced the *HIS1* deletion cassette and then restored histidine prototrophy by transformation with *Nru*I-digested pGEM-HIS1 [55].

CMM3 and CMM1 (wild type prototrophic equivalent of CMM3) [34] were also used in the phenotypic assays. In all assays BWP17 and CMM1 were used as control strains but they usually gave identical results so only one was generally shown. The exception was for the liquid growth curves as BWP17 plateaued at a lower OD_600_ than CMM1. In this case, the *tye7* strain was compared to BWP17 and normalized to CMM1 for graphical purposes.

As uridine auxotrophy affects virulence but can be restored by integration of *URA3* at the *RPS10* locus [56], for the mouse studies the CMM1, *gal4/gal4/tye7/tye7*, and *gal4/GAL4/tye7/TYE7* strains were made prototrophic by targeting the *URA3* marker to the *RPS10* locus through *Stu*I digestion of CIp10. Correct integration was confirmed by PCR and qPCR verified that only one copy of *URA3* was integrated.

### Phenotypic assays

Serial spotting plate assays were carried out as described [57] except cells were washed twice with sterile water before plating. Cells were plated on synthetic complete media containing the carbon source at 0.2% or 2% and agarose at 2% to minimize carbon source impurities. Antimycin A (2 µg/ml) was added to inhibit respiration. For liquid assays, cells were grown to log phase in synthetic medium with 5% glycerol, washed twice with sterile water, and resuspended at an OD_600_ = 0.1 in synthetic media containing the carbon source at 2%. Cells were either grown at 30°C in flasks with shaking (aerobic conditions) or in microtiter plates without shaking (static conditions). Samples were done in triplicate and the average was used for analysis. For growth under hypoxic conditions, cells were spotted on YPD plates and incubated in an anaerobic chamber. The chamber was flushed daily with nitrogen to remove oxygen and any by-products.

### ChIP-CHIP analysis

ChIP experiments were performed as previously described [52] with some modifications. Briefly, cells were grown to OD_600_ = 2 in 40 ml of YPD, YPGal, or YPGly. Tagged ChIPs were labeled with Cy5 dye and untagged (mock) ChIPs were labeled with Cy3 dye. Microarray hybridization and washing were performed as described [58]. Scanning was done with a ScanArray Lite microarray scanner (Perkin Elmer). QuantArray was used to quantify fluorescence intensities. Data handling and analysis were carried out using Genespring v.7.3 (Agilent Technologies). The significance cut-off was determined using the distribution of log-ratios for each factor. A minimum of three biological replicates were analyzed for each carbon source condition with hybridization to single spot full-genome (ORF and intergenic) microarrays containing 11,817 70-mer oligonucleotide probes [52]. For determining the number of targets and GO analysis with the single probe microarrays, the cut-off was a fold enrichment >1.5 and a t-test *P*-value<0.1.

One replicate of the ChIP-CHIP experiments for each carbon source condition was hybridized to a custom designed whole-genome tiling array for further analysis. Using the *C. albicans* Genome Assembly 21 [59] and the *MTL* alpha locus [60], we extracted a continuous series of 242,860 60 bp oligonucleotides each overlapping by 1 bp. We eliminated 2062 probes containing stretches of at least 13 A/T nucleotides. The remaining 240,798 probes were used to produce a whole-genome tiling array using the Agilent Technologies eArray service (https://earray.chem.agilent.com/erray/).

### Tiling array data processing and peak detection

Lowess normalization of the intensity ratio of each of the 240,798 probes was done using an in-house software implementation. The signal along each chromosome was smoothed using a median filter (n = 3) followed by a Gaussian low-pass filter (σ = 150 bp). For peak localization, the smoothed signal was interpolated at 10 bp intervals using cubic-spline resampling. Peaks were reported in decreasing order of the smoothed intensities.

### RNA extraction and transcription profiles

RNA was extracted with the RNeasy Kit (Qiagen) as per manufacturer's instructions. Briefly, cells were grown to OD_600_ = 0.8 in YPD, YPGal, or YPGly in aerated flasks (normoxia) and disrupted using acid washed glass beads. Transcriptional profiling was carried out as described [58] with 20 µg of RNA used for cDNA synthesis. A minimum of three biological replicates on double spotted ORF microarrays (6,394 intragenic 70-mer oligonucleotide probes) were used for analysis [58]. Scanning and analysis were carried out as described for ChIP-CHIP except scanning was performed at two different laser PMTs to avoid saturated signals for a few highly expressed transcripts including several glycolytic genes. For GO analysis and the supplementary complete lists the cut-off was a difference in expression >1.5 or <0.67 and a t-test *P*-value<0.05.

Expression profiles under hypoxic conditions were performed as described above except bottles containing YPD media were flushed with nitrogen to remove oxygen. Two biological replicates were performed and statistical analysis was done as with normoxic conditions.

For qPCR analysis of glycolytic gene expression under hypoxic conditions, BWP17, *tye7*, and *gal4tye7* cultures were grown to OD_600_ = 0.7 in YPD in an aerated flask. The cultures were then transferred to bottles flushed with nitrogen and grown for an additional 30 min before the RNA was extracted as above and analyzed by qPCR.

For hypoxic induction kinetic analysis, BWP17 was grown to OD_600_ = 0.8 in YPD in an aerated flask. Half the culture was transferred to bottles flushed with nitrogen while the other half was left to grow in the aerated flask. At different time points the RNA was extracted and compared by qPCR.

### qPCR analysis

For qPCR, cDNA was synthesized from 5 µg of total RNA using the reverse-transcription system (50 mM Tris-HCl, 75 mM KCl, 5 mM DTT, 3 mM MgCl_2_, 400 nM oligo(dT)_15_, 1 µM random octamers, 0.5 mM dNTPs, 200 units Superscript III reverse transcriptase; Invitrogen). The mixture was incubated for 60 min at 50°C. Aliquots were used for qPCR, which was performed using the Corbett Rotor-Gene RG3000A (Corbett Research) with SYBR Green fluorescence (Qiagen). Cycling was 10 min at 95°C followed by 40 cycles (95°C, 10 s; 58°C, 15 s; 72°C, 20 s). Samples were done in triplicate and means were used for calculations. Fold changes were estimated using the comparative ΔΔCt method as described [61] with the coding sequence of the *C. albicans ACT1* ORF as a reference.

### Measuring trehalose and glycogen levels

Trehalose levels were measured as based on previous studies [62]. Briefly, cells were either grown to log phase (OD_600_ = 2) or grown for 40 hours to reach stationary phase, washed twice with cold water, and resuspended in water. Cells were lysed by incubating at 95°C for 30 min and the supernatant was used for enzymatic analysis. Reactions (50 µl of sample, 100 µl 270 mM citric acid buffer pH 5.7, and 0.15 U trehalase (Sigma)) were incubated at 37°C for 5 hours. Glucose amounts were assayed with the hexokinase glucose kit (Sigma) with endogenous glucose levels determined based on reactions without trehalase. A BCA protein assay (Pierce) was performed as per manufacturer's instructions. Relative trehalose levels were based on nmol trehalose per mg of cell protein. Three biological replicates were performed for each strain and condition. Glycogen levels were estimated using the iodine vapor method [63]. Cells were serially diluted, spotted on YPD plates, and incubated for 24 hours at 30°C. The cells were then exposed to iodine vapor for 5 minutes.

### Measuring protein expression levels

To determine the protein expression of Gal4p and Tye7p under different carbon sources, overnight cultures in YPD were diluted to OD_600_ = 0.4 and grown for 2 hours in fresh YPD. Cells were washed twice with sterile water and resuspended in YP, YPD, YPGal, or YPGly media and grown for an additional 3 hours. The cells were then washed with TBS buffer and lysed using acid washed glass beads (same lysis buffer as ChIP-CHIP except for addition of a phosphatase inhibitor cocktail (Roche)). The protein extract was clarified by centrifugation and a BCA protein assay (Pierce) was performed as per manufacturer's instructions. Gal4p and Tye7p were separated on a 10% SDS gel and transferred to a PVDF membrane. A rabbit polyclonal antibody directed against the TAP-tag (Open Biosystems) was used (1∶2000 dilution). A mouse anti-actin monoclonal antibody (1∶500 dilution, Chemicon) was used to probe actin as a loading control. HRP-conjugated anti-rabbit and anti-mouse secondary antibodies (Santa Cruz) were used (1∶10,000 dilution). The HRP signal was revealed using the Lumi-Light Western Blotting Substrate (Roche).

### Virulence studies

For *Galleria mellonella* studies, overnight cultures were washed twice with PBS and resuspended in PBS at OD_600_ = 8. Larvae, in the final instar, weighing 180±10 mg were injected between the third pair prothoracic legs with 10 µl of suspension (8×10^5^ cells). Infected larvae were incubated at 37°C in the dark with excess of a multigrain diet supplemented with glycerol and vitamins [64]. Four replicates, each consisting of 20 insects, were carried out with survival rates measured daily for a period of 7 days. Death was determined based on the lack of response to touch and the inability to right themselves. A BWP17 culture was irradiated with UV for 2 hours and incubated at 95°C for 1 hour before injection to confirm that viable *C. albicans* cells were responsible for death. Kaplan-Meier survival curves were created and compared with the log-rank test (GraphPad Prism 5).

Mouse studies were carried out as previously described [65]. Briefly, 8–12 week-old A/J and C57BL/6J mice (Jackson Laboratories, Bar Harbor, ME) were inoculated via the tail vein with 200 µl of a suspension containing 3×10^5^ *C. albicans* in PBS. Six mice, three female and three male, were used for each experimental group except for the *gal4tye7* injected A/J set where six females and six males were used. Mice were closely monitored and those showing extreme lethargy were considered moribund and were euthanized. Target organs were removed aseptically and homogenized in PBS before plating on YPD plates containing chloramphenicol (34 µg/ml). The number of yeast colonies per organ was determined, log-transformed, and compared using the Mann-Whitney test (GraphPad Prism 5). Comparative genomic hybridization (CGH) analysis was performed on all strains prior to injection to verify that no aneuploidy arose as a result of any of the genetic manipulations. All experimental procedures involving mice were approved by the Biotechnology Research Institute Animal Care Committee, which operated under the guidelines of the Canadian Council of Animal Care.

## Supporting Information

Figure S1 Liquid assays verify that *GAL4* and *TYE7* are involved in fermentative growth with glucose, fructose or mannose as the carbon source. WT refers to strain CMM1. (A) Growth curves where strains were grown with aeration. (B) Growth curves where strains were grown without aeration.
(1.30 MB PDF)

Figure S2 Overlap of binding targets for Gal4p and Tye7p under glucose, galactose, and glycerol growth conditions. Peaks common to all three carbon sources had peak intensities >2 fold in all three conditions with the tiling array data. A peak was considered to be unique to a carbon source (or sources) if the peak intensity in the condition (or conditions) was >2 fold and the peak intensities in the remaining carbon sources were <1.4 fold. This result confirms that for most targets Gal4p displays carbon-source dependent binding while Tye7p binding is more constitutive.
(0.53 MB PDF)

Figure S3 Both Gal4p and Tye7p are induced by glucose but Tye7p has a higher constitutive expression. Protein expression levels of Gal4p and Tye7p under different carbon sources are presented. YP media with no additional carbon source was included to establish the basal level of expression. Actin was used as the loading control.
(0.24 MB PDF)

Figure S4 qPCR validation of transcription profile results under oxygen rich growth conditions. Expression levels for *HXK2* (A), *CDC19* (B), and *TDH3* (C) were determined in *tye7* and *gal4tye7* strains relative to the wild type (BWP17) under normoxic growth conditions. *ACT1* was used as the reference. *TDH3* was included since its expression was unchanged according to the transcription profiles despite being bound by Gal4p and Tye7p (Figure 6A); however, qPCR showed that *TDH3* is indeed activated by Gal4p and Tye7p. We later discovered that there was a spotting problem with the *TDH3* probe for the particular set of microarrays used and therefore we omitted the gene from the expression profile heat map displays in Figures 4B, 6B, and 8B.
(0.30 MB PDF)

Figure S5 *GAL4* and *TYE7* are important for hypoxic growth at both 30°C and 37°C. (A) Strains were serially diluted on YPD plates and incubated in an anaerobic jar at 37°C for 2 days. WT refers to strain CMM1. (B) Strains were serially diluted on two separate YPD plates and incubated in an anaerobic jar at 30°C for 4 days. One representative dilution is shown. The WT for the top row is CMM1 and the WT for the bottom row is BWP17. One copy of either *GAL4* or *TYE7* is able to restore the growth defect of the double mutant strain although the *GAL4* revertant does not grow at wild type levels since *TYE7* is still deleted.
(1.67 MB PDF)

Figure S6 *GAL4* and *TYE7* are initially induced by hypoxia. The expression levels of *GAL4*, *TYE7*, and *CDC19* were measured in BWP17 by qPCR at different time points following a shift from normoxic to hypoxic growth conditions. *ACT1* was used as the reference.
(0.57 MB PDF)

Table S1 Doubling times under fermentative growth conditions.
(0.02 MB XLS)

Table S2 Gal4p ChIP-CHIP targets with glucose as the carbon source.
(0.03 MB XLS)

Table S3 Tye7p ChIP-CHIP targets with glucose as the carbon source.
(0.05 MB XLS)

Table S4 Top Gal4p YPD binding peaks with tiling array.
(0.02 MB XLS)

Table S5 Top Tye7p YPD binding peaks with tiling array.
(0.02 MB XLS)

Table S6 ChIP-CHIP binding enrichments of selected metabolic genes under different carbon sources.
(0.03 MB XLS)

Table S7 Expression levels of selected metabolic genes for *tye7* and *gal4tye7* strains under different carbon sources and oxygen levels.
(0.03 MB XLS)

Table S8 Genes down-regulated in *tye7* strain in glucose growth conditions.
(0.03 MB XLS)

Table S9 Genes down-regulated in *gal4tye7* strain in glucose growth conditions.
(0.03 MB XLS)

Table S10 Genes down-regulated in *tye7* strain in galactose growth conditions.
(0.02 MB XLS)

Table S11 Genes down-regulated in *gal4tye7* strain in galactose growth conditions.
(0.04 MB XLS)

Table S12 Genes down-regulated in *tye7* strain in glycerol growth conditions.
(0.03 MB XLS)

Table S13 Genes down-regulated in *gal4tye7* strain in glycerol growth conditions.
(0.03 MB XLS)

Table S14 Genes down-regulated in *tye7* strain in glucose hypoxic growth conditions.
(0.07 MB XLS)

Table S15 Genes up-regulated in *tye7* strain in glucose hypoxic growth conditions.
(0.05 MB XLS)

Table S16 Genes down-regulated in BWP17 strain in glucose hypoxic growth conditions.
(0.04 MB XLS)

Table S17 Genes up-regulated in BWP17 strain in glucose hypoxic growth conditions.
(0.05 MB XLS)

Table S18 Strains used in this study.
(0.03 MB XLS)

Table S19 Plasmids used in this study.
(0.02 MB XLS)

Table S20 Primers used in this study.
(0.02 MB XLS)

Dataset S1 ChIP-CHIP normalized tiling array data. Columns correspond to (L to R) Tye7p YPD, YPGal, YPGly; Gal4p YPD, YPGal, YPGly. The start (>) and end (|) of each ORF is indicated. Values given for each probe are log_2_ (fold enrichment) with negative values indicating enrichment for Gal4p or Tye7p binding. Significant binding events are highlighted in green.
(3.52 MB ZIP)
