# Supplementary figures and images for: Transcriptional Regulation of Carbohydrate Metabolism in the Human Pathogen Candida albicans
Source: PLoS Pathog. 2009 Oct 9;5(10):e1000612. doi: 10.1371/journal.ppat.1000612 (PMC2749448; doi:10.1371/journal.ppat.1000612)

**A**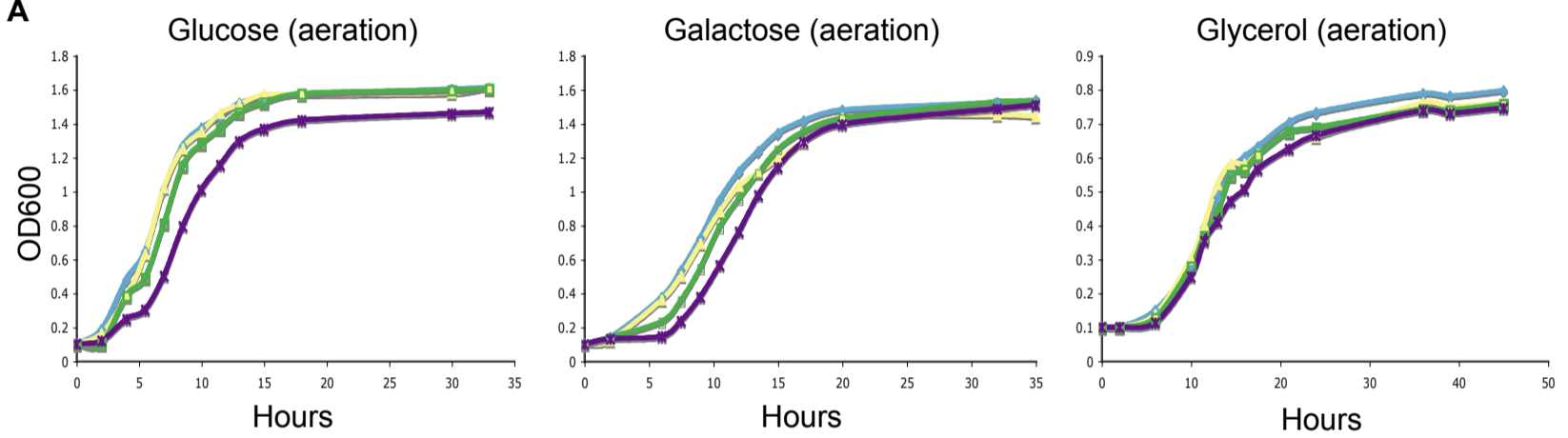**B**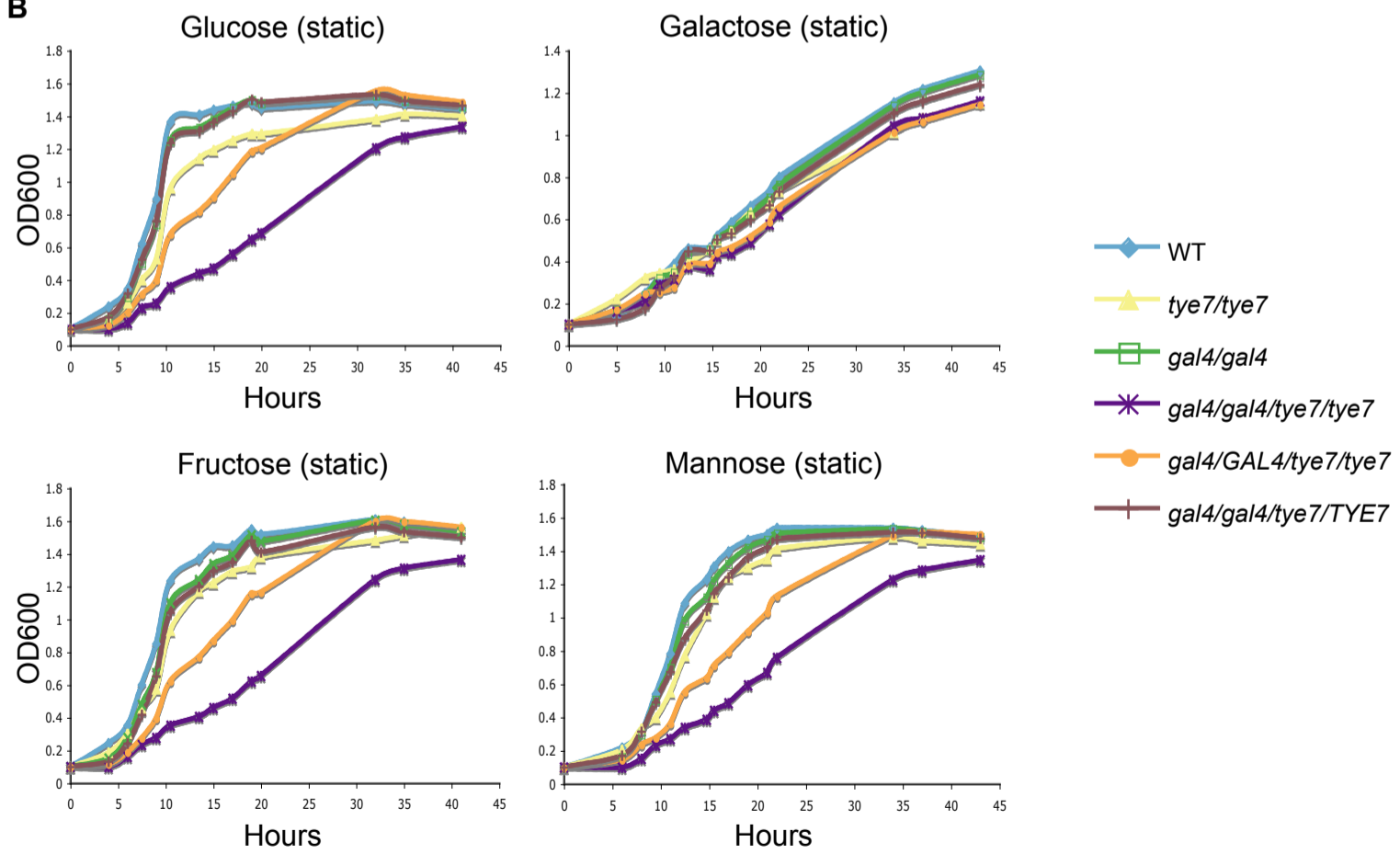

Supplement: Figure S1 — Liquid assays verify that GAL4 and TYE7 are involved in fermentative growth with glucose, fructose or mannose as the carbon source. WT refers to strain CMM1. (A) Growth curves where strains were grown with aeration. (B) Growth curves where strains were grown without aeration. (1.30 MB PDF) [file ppat.1000612.s001.pdf]

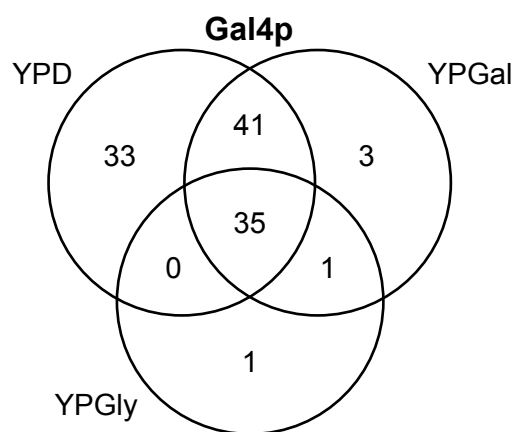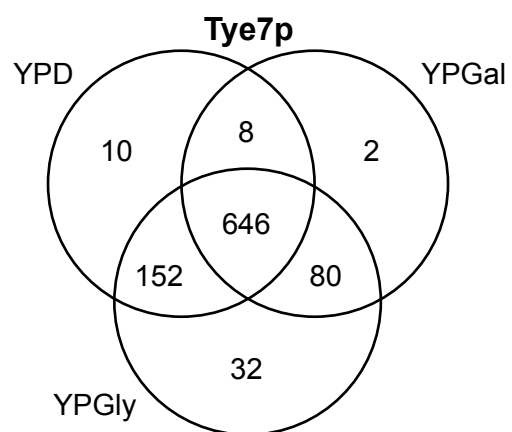

Supplement: Figure S2 — Overlap of binding targets for Gal4p and Tye7p under glucose, galactose, and glycerol growth conditions. Peaks common to all three carbon sources had peak intensities >2 fold in all three conditions with the tiling array data. A peak was considered to be unique to a carbon source (or sources) if the peak intensity in the condition (or conditions) was >2 fold and the peak intensities in the remaining carbon sources were <1.4 fold. This result confirms that for most targets Gal4p displays carbon-source dependent binding while Tye7p binding is more constitutive. (0.53 MB PDF) [file ppat.1000612.s002.pdf]

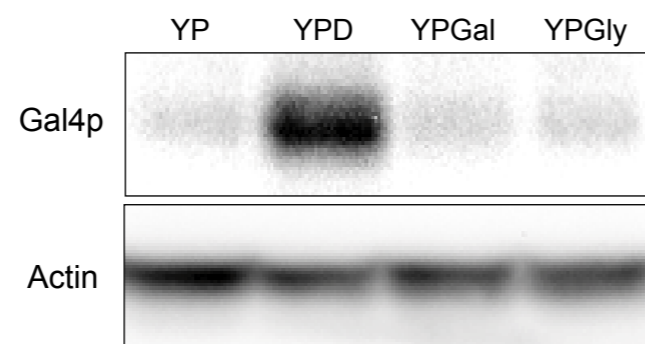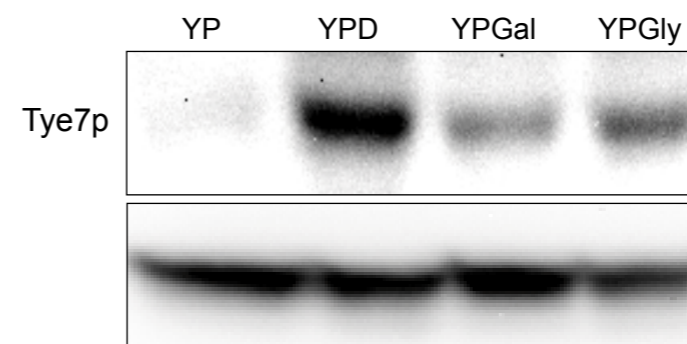

Supplement: Figure S3 — Both Gal4p and Tye7p are induced by glucose but Tye7p has a higher constitutive expression. Protein expression levels of Gal4p and Tye7p under different carbon sources are presented. YP media with no additional carbon source was included to establish the basal level of expression. Actin was used as the loading control. (0.24 MB PDF) [file ppat.1000612.s003.pdf]

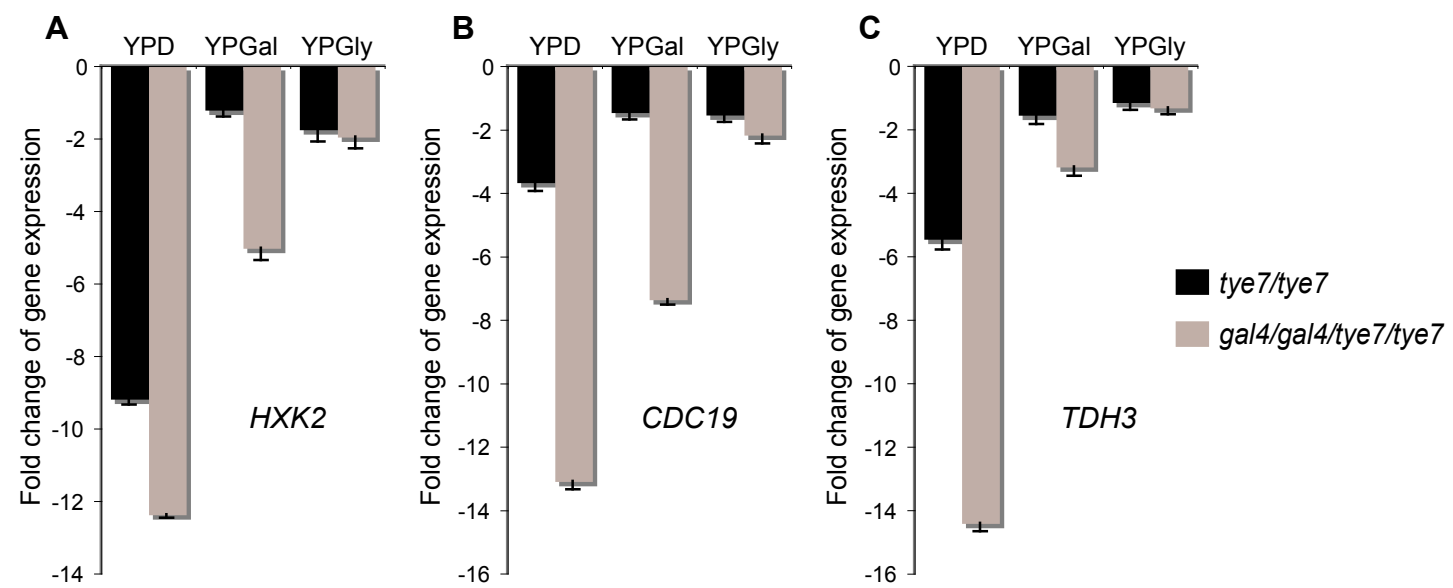

Supplement: Figure S4 — qPCR validation of transcription profile results under oxygen rich growth conditions. Expression levels for HXK2 (A), CDC19 (B), and TDH3 (C) were determined in tye7 and gal4tye7 strains relative to the wild type (BWP17) under normoxic growth conditions. ACT1 was used as the reference. TDH3 was included since its expression was unchanged according to the transcription profiles despite being bound by Gal4p and Tye7p (Figure 6A); however, qPCR showed that TDH3 is indeed activated by Gal4p and Tye7p. We later discovered that there was a spotting problem with the TDH3 probe for the particular set of microarrays used and therefore we omitted the gene from the expression profile heat map displays in Figures 4B, 6B, and 8B. (0.30 MB PDF) [file ppat.1000612.s004.pdf]

**A**

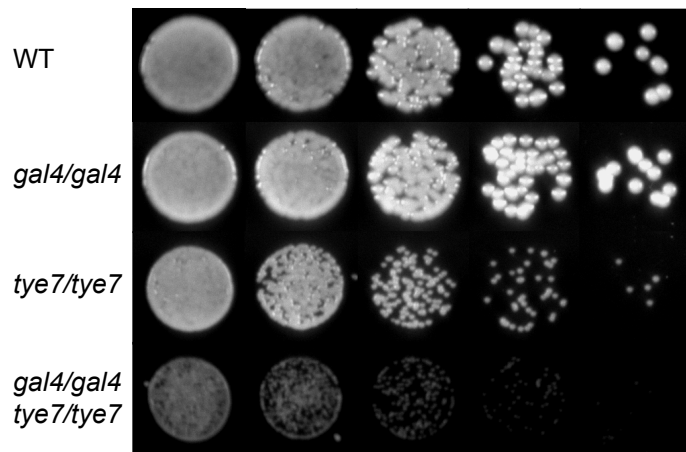

**B**

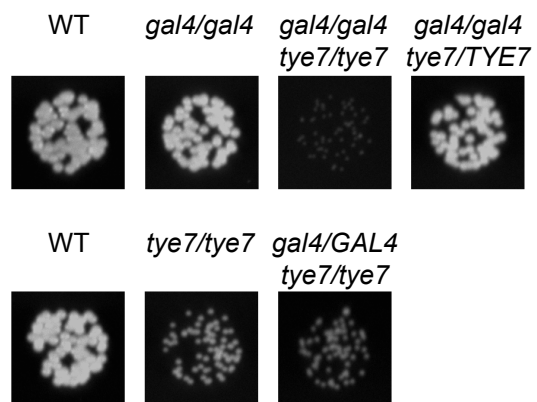

Supplement: Figure S5 — GAL4 and TYE7 are important for hypoxic growth at both 30°C and 37°C. (A) Strains were serially diluted on YPD plates and incubated in an anaerobic jar at 37°C for 2 days. WT refers to strain CMM1. (B) Strains were serially diluted on two separate YPD plates and incubated in an anaerobic jar at 30°C for 4 days. One representative dilution is shown. The WT for the top row is CMM1 and the WT for the bottom row is BWP17. One copy of either GAL4 or TYE7 is able to restore the growth defect of the double mutant strain although the GAL4 revertant does not grow at wild type levels since TYE7 is still deleted. (1.67 MB PDF) [file ppat.1000612.s005.pdf]

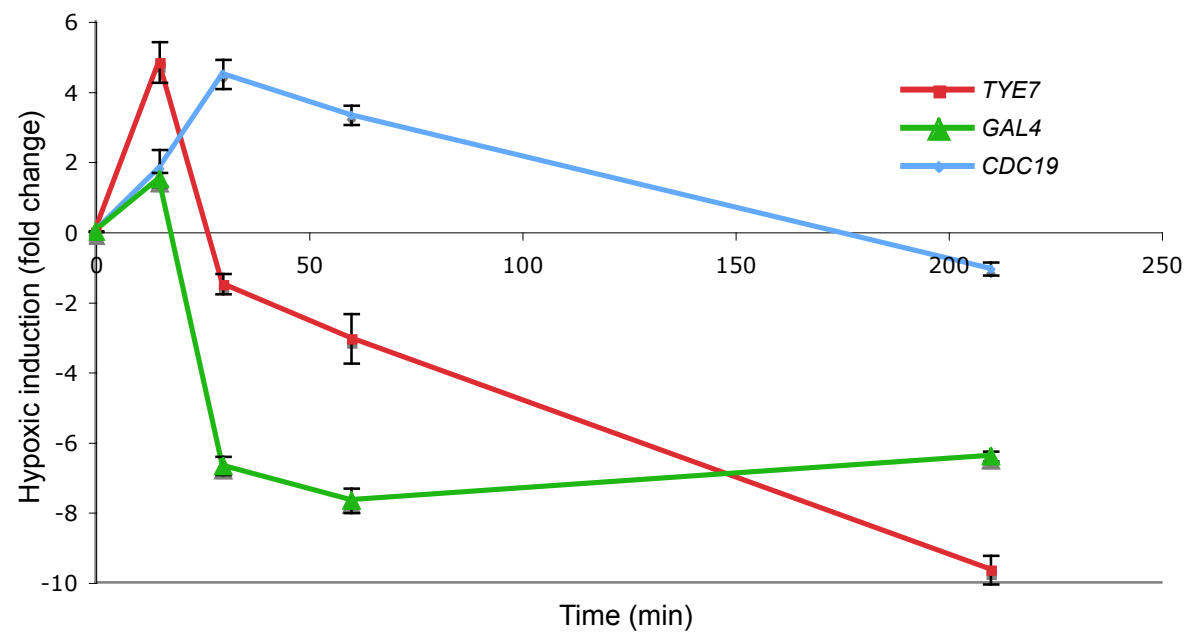

Supplement: Figure S6 — GAL4 and TYE7 are initially induced by hypoxia. The expression levels of GAL4, TYE7, and CDC19 were measured in BWP17 by qPCR at different time points following a shift from normoxic to hypoxic growth conditions. ACT1 was used as the reference. (0.57 MB PDF) [file ppat.1000612.s006.pdf]
