# Supplementary material for: Transcriptional Regulation of Carbohydrate Metabolism in the Human Pathogen Candida albicans
Source: PLoS Pathog. 2009 Oct 9;5(10):e1000612. doi: 10.1371/journal.ppat.1000612 (PMC2749448; doi:10.1371/journal.ppat.1000612)
Supplement: Dataset S1 — ChIP-CHIP normalized tiling array data. Columns correspond to (L to R) Tye7p YPD, YPGal, YPGly; Gal4p YPD, YPGal, YPGly. The start (>) and end (|) of each ORF is indicated. Values given for each probe are log2 (fold enrichment) with negative values indicating enrichment for Gal4p or Tye7p binding. Significant binding events are highlighted in green. (3.52 MB ZIP) [file ppat.1000612.s027.zip › Dataset S1.html]

```
Ca21Chr1_0000001  0.37  0.80  1.12  0.12 -0.06 -0.68 
Ca21Chr1_0000060  0.42 -0.15  0.95 -0.20  0.23 -0.50 
Ca21Chr1_0000119  0.40  0.61  0.78 -0.25  0.24 -0.23 
Ca21Chr1_0000178  0.23  0.45  1.04  0.08 -0.08  0.41 
Ca21Chr1_0000237  0.22 -0.01  0.50 -0.40 -0.21 -0.41 
Ca21Chr1_0000296 -0.03 -0.18  0.94  0.16 -0.36 -0.52 
Ca21Chr1_0000355  0.32  0.04  0.53 -0.27  0.06 -0.40 
Ca21Chr1_0000414 -0.14 -0.31  0.40 -0.11 -0.01 -0.31 
Ca21Chr1_0000473  0.29 -0.12  0.94  0.24  0.16 -0.06 
Ca21Chr1_0000532  1.61  0.31  1.02 -0.06 -0.24 -0.09 
Ca21Chr1_0000591  0.46 -0.30  0.60 -0.19  0.10 -0.40 
Ca21Chr1_0000650  0.70  0.31  0.96 -0.48  0.50 -0.23 
Ca21Chr1_0000709 -0.17  1.05  0.22  0.81  0.83  0.07 
Ca21Chr1_0000768 -0.66  0.70  0.77  0.07 -0.00 -0.20 
Ca21Chr1_0000827  0.56  0.76  1.23 -0.51  0.42  0.11 
Ca21Chr1_0000886  0.46 -0.22  0.56 -0.22  0.60 -0.42 
Ca21Chr1_0000945  0.31  0.14  1.06 -0.12 -0.15  0.54 
Ca21Chr1_0001004    NA    NA    NA    NA    NA    NA
Ca21Chr1_0001063  0.41 -0.06  0.77  0.03  0.33 -0.27 
Ca21Chr1_0001122  0.59  0.25  0.91 -0.40  0.38 -0.30 
Ca21Chr1_0001181  0.81 -0.05  0.92 -0.12 -0.05 -0.15 
Ca21Chr1_0001240  0.84  0.59  0.91 -0.16  0.61  0.37 
Ca21Chr1_0001299 -0.18  0.43  1.20  0.05 -0.17  0.36 
Ca21Chr1_0001358  0.84  0.25  1.21  0.12  0.09 -0.24 
Ca21Chr1_0001417  0.22  0.68  0.58  0.04  0.03 -0.13 
Ca21Chr1_0001476  0.45  0.05  1.08  0.06  0.04 -0.11 
Ca21Chr1_0001535  0.61  0.41  0.90 -0.03  0.17 -0.08 
Ca21Chr1_0001594  0.58  0.12  0.90 -0.21  0.24 -0.41 
Ca21Chr1_0001653  0.66  0.48  1.13  0.34  0.25 -0.02 
Ca21Chr1_0001712  0.59  0.40  1.21 -0.04  0.26 -0.85 
Ca21Chr1_0001771  0.92  0.52  0.91  0.19  0.01 -0.14 
Ca21Chr1_0001830  0.46  0.66  0.39  0.12 -0.18  0.46 
Ca21Chr1_0001889  0.58  0.45  0.69  0.02  0.40 -0.22 
Ca21Chr1_0001948  0.41  0.49  0.75  0.05  0.46 -0.57 
Ca21Chr1_0002007  0.65  0.26  1.02  0.26  1.45  0.39 
Ca21Chr1_0002066  0.73  0.48  1.14 -0.01  0.57 -0.76 
Ca21Chr1_0002125 -0.18  0.28  0.46  0.06  0.42  0.18 
Ca21Chr1_0002184  0.29  0.37  1.15  0.42  0.43 -0.45 
Ca21Chr1_0002243  0.15  0.73  0.36  0.08  0.13 -0.47 
Ca21Chr1_0002302  0.51  0.28  0.84  0.04  0.24 -0.35 
Ca21Chr1_0002361  0.39  0.16  0.69 -0.23  0.45 -0.36 
Ca21Chr1_0002420  0.58 -0.51  1.02 -0.24 -0.03 -0.11 
Ca21Chr1_0002479  0.07  0.32  0.75 -0.11 -0.06 -0.08 
Ca21Chr1_0002538  1.17  0.20  0.29  0.30  0.55 -0.50 
Ca21Chr1_0002597 -0.25 -0.14  0.55  0.27  0.29 -0.29 
Ca21Chr1_0002656  0.09  0.28  0.85 -0.10 -0.15 -0.75 
Ca21Chr1_0002715  0.33  0.22  1.25 -0.23 -0.16 -0.26 
Ca21Chr1_0002774  0.35  0.06  0.53 -0.17  0.52 -0.09 
Ca21Chr1_0002833  0.61  0.18  1.13  0.26  0.55 -0.03 
Ca21Chr1_0002892  0.66  0.59  1.14 -0.10  0.32 -0.07 
Ca21Chr1_0002951  0.40  0.52  1.11 -0.14  0.19 -0.14 
Ca21Chr1_0003010  0.53  0.31  0.92 -0.02  0.14  0.28 
Ca21Chr1_0003069 -0.02  0.56  1.16  0.24  0.46  0.23 
Ca21Chr1_0003128  0.43  0.20  0.93  0.27  0.43  0.14 
Ca21Chr1_0003187  0.70 -0.39  1.03 -0.37  0.20  0.05 
Ca21Chr1_0003246  0.90  1.17  1.02 -0.02  0.08  0.17 
Ca21Chr1_0003305  0.50  0.73  1.06 -0.24  0.10 -0.04 
Ca21Chr1_0003364  0.76  0.70  1.19  0.11  0.20  0.18 
Ca21Chr1_0003423  1.06  0.53  1.59  0.31  0.24 -0.08 
Ca21Chr1_0003482  0.65  0.12  1.07  0.23  0.16  0.01 
Ca21Chr1_0003541  0.23  0.27  0.87 -0.02 -0.00 -0.55 
Ca21Chr1_0003600  0.41 -0.28  0.58  0.03 -0.34 -0.25 
Ca21Chr1_0003659 -0.03  0.32  0.65  0.23  0.27 -0.24 
Ca21Chr1_0003718  0.09  0.07  0.62 -0.33  0.20 -0.37 
Ca21Chr1_0003777  0.27  0.22  0.36  0.03  0.36 -0.22 
Ca21Chr1_0003836 -0.09 -0.05  0.29 -0.18  0.05 -0.11 
Ca21Chr1_0003895 -0.18  0.16  0.08 -0.55  0.47 -0.24 
Ca21Chr1_0003954  0.16  0.09  0.79 -0.03  0.06 -0.01 
Ca21Chr1_0004013 -0.24 -0.38 -0.04 -0.47 -0.21 -0.69  orf19.6115>
Ca21Chr1_0004072 -0.34  0.32 -0.04 -0.32  0.02 -0.56 
Ca21Chr1_0004131  0.40  0.07  0.84 -0.01  0.16 -0.01 
Ca21Chr1_0004190  0.57  0.16  0.79 -0.48  0.05 -0.11 
Ca21Chr1_0004249  0.20  0.48  0.89  0.23  0.58 -0.26 
Ca21Chr1_0004308  0.40  0.55  1.30 -0.26  0.13 -0.46 
Ca21Chr1_0004367 -0.37  0.41  0.01 -0.08  0.63  0.29  orf19.6115| |orf19.6114
Ca21Chr1_0004426 -0.32  0.30  0.83 -0.04  0.46  0.12 
Ca21Chr1_0004485  0.42  0.58  1.12 -0.02  0.52  0.12 
Ca21Chr1_0004544  0.67  0.44  0.77  0.23  0.41  0.04 
Ca21Chr1_0004603  0.65  0.36  0.82 -0.38  0.42 -0.28 
Ca21Chr1_0004662  0.86 -0.17  0.80 -0.24  0.29  0.58  <orf19.6114
Ca21Chr1_0004721  0.56  0.58  0.54 -0.54  0.34  0.50 
Ca21Chr1_0004780  0.67  0.37  0.90 -0.32  0.02 -0.55 
Ca21Chr1_0004839  0.55  0.02  0.56 -0.38 -0.12 -0.47 
Ca21Chr1_0004898  0.28  0.72  0.99 -0.13 -0.32 -0.14 
Ca21Chr1_0004957 -0.10  0.62  0.95 -0.19  0.15 -0.63 
Ca21Chr1_0005016  0.38  0.27  1.29 -0.18 -0.08 -0.34 
Ca21Chr1_0005075  0.74  0.44  1.29 -0.47 -0.19 -0.48 
Ca21Chr1_0005134  0.60 -0.08  0.82 -0.96 -0.36 -0.54 
Ca21Chr1_0005193  0.22 -0.06  0.43 -0.96 -0.39 -0.55 
Ca21Chr1_0005252  0.06  0.41  0.85 -0.86 -0.56 -0.55 
Ca21Chr1_0005311 -0.32  0.11  0.54 -1.34 -0.44 -0.53 
Ca21Chr1_0005370 -0.04 -0.38  0.18 -0.11 -0.69 -0.93 
Ca21Chr1_0005429 -0.25  0.01  0.42 -0.62 -0.69 -0.13 
Ca21Chr1_0005488 -0.11  0.16  0.37 -0.59 -0.98 -0.29 
Ca21Chr1_0005547  0.00  0.96  0.64 -0.52 -0.91 -0.75 
Ca21Chr1_0005606 -0.05  0.19  0.27 -0.67 -0.66 -0.46 
Ca21Chr1_0005665 -0.32  0.28  0.60 -0.95 -0.87  0.15 
Ca21Chr1_0005724 -0.37 -0.05  0.14 -1.83 -1.67 -0.61 
Ca21Chr1_0005783 -0.20 -0.09  0.45 -0.48 -1.01 -0.89 
Ca21Chr1_0005842 -0.01  0.13  0.35 -0.50 -1.05 -0.84 
Ca21Chr1_0005901 -0.12  0.43  0.63 -0.56 -1.75 -1.06 
Ca21Chr1_0005960  0.03  0.51  0.48 -0.57 -1.66 -1.48 
Ca21Chr1_0006019 -0.17  0.11  0.27 -0.62 -1.27 -1.57 
Ca21Chr1_0006078  0.05  0.68  0.56 -2.78 -2.03 -1.60 
Ca21Chr1_0006137 -0.06  1.10  1.02 -0.46 -1.06 -1.02 
Ca21Chr1_0006196  0.42  0.38  0.52 -2.56 -1.54 -1.18 
Ca21Chr1_0006255  0.06  0.39 -0.18 -2.28 -1.35 -1.17 
Ca21Chr1_0006314 -0.06  0.12 -0.08 -1.28 -0.98 -0.71 
Ca21Chr1_0006373 -0.73  0.00 -0.56 -1.50 -0.77 -0.89 
Ca21Chr1_0006432 -1.10 -0.23 -0.82 -1.81 -1.01 -0.66 
Ca21Chr1_0006491 -0.86 -0.29 -0.62 -0.92 -0.96 -0.92 
Ca21Chr1_0006550 -0.20 -0.69 -0.01 -1.34 -0.43 -0.54 
Ca21Chr1_0006609 -0.25  0.04 -0.53 -0.24 -0.26 -0.72 
Ca21Chr1_0006668 -0.37  0.12 -0.29 -0.95 -0.33 -0.59 
Ca21Chr1_0006727  0.35  0.04  0.37 -0.69 -0.66 -0.00 
Ca21Chr1_0006786  0.45  0.54  0.27 -0.91 -0.08 -0.47 
Ca21Chr1_0006845  0.33  0.61  0.48 -0.81 -0.35  0.46 
Ca21Chr1_0006904  0.37  0.40  0.75 -0.42 -0.45 -0.46 
Ca21Chr1_0006963  0.14  0.89  0.37 -0.25 -0.23 -0.16 
Ca21Chr1_0007022  0.18  0.80  0.35  0.43 -0.16 -0.06 
Ca21Chr1_0007081 -0.41  0.36 -0.15 -0.48 -0.35 -0.93 
Ca21Chr1_0007140  0.66  0.88  0.49 -0.59 -0.05 -0.14 
Ca21Chr1_0007199  0.56  0.87  0.77 -0.30  0.42 -0.66 
Ca21Chr1_0007258  0.28  0.64  0.70 -0.46 -0.15 -0.58 
Ca21Chr1_0007317  0.07  0.75  0.59 -0.43 -0.14 -0.56 
Ca21Chr1_0007376  0.30  0.82  0.45 -0.26 -0.26 -0.34 
Ca21Chr1_0007435  0.47  0.42  0.77 -0.40  0.25 -0.05 
Ca21Chr1_0007494  0.24  0.27  0.44 -0.26  0.02 -0.59 
Ca21Chr1_0007553  0.92  0.29  0.25 -0.21 -0.45 -0.44 
Ca21Chr1_0007612  0.24 -0.44  0.26 -0.14 -0.41 -0.06 
Ca21Chr1_0007671  0.13 -0.14 -0.20 -0.05 -0.15  0.06 
Ca21Chr1_0007730  0.25 -0.09  0.12 -0.19 -0.11  0.04 
Ca21Chr1_0007789 -0.12  0.44  0.31  0.20 -0.07 -0.62 
Ca21Chr1_0007848  0.45  0.17  0.57 -0.23 -0.21 -0.64 
Ca21Chr1_0007907  0.32  0.42  0.05 -0.25  0.77 -0.66 
Ca21Chr1_0007966  0.13  0.04  0.47 -0.26  0.11 -0.20 
Ca21Chr1_0008025  0.54  0.50  0.58 -0.14  0.11 -0.55 
Ca21Chr1_0008084 -0.32  0.83  0.28 -0.77 -0.33  0.10 
Ca21Chr1_0008143  0.07  0.19  0.47 -0.50 -0.71 -0.18 
Ca21Chr1_0008202  0.53  0.35  0.60 -0.68 -0.62 -0.33 
Ca21Chr1_0008261  0.61  0.41  0.39 -1.28 -1.04 -0.58 
Ca21Chr1_0008320  0.46  0.04  0.81 -1.29 -1.00 -0.32 
Ca21Chr1_0008379  0.73  0.13  0.89 -0.26 -0.43 -0.30 
Ca21Chr1_0008438 -0.09  1.01  0.73 -0.31  0.31 -0.54 
Ca21Chr1_0008497  0.18  0.61  0.29 -0.00  0.10 -0.22 
Ca21Chr1_0008556  0.26  0.46  0.92 -0.55  0.03 -0.04  |orf19.6113
Ca21Chr1_0008615 -0.34  0.67  1.18 -0.13  0.20 -0.16 
Ca21Chr1_0008674  0.32  0.69  0.70 -0.17  0.04 -0.22 
Ca21Chr1_0008733  0.40  0.68  0.91 -0.36  0.36 -0.26 
Ca21Chr1_0008792  0.31  0.40  0.94  0.14 -0.03 -0.43 
Ca21Chr1_0008851  0.40  0.35  1.13  0.00  0.27 -0.52  <orf19.6113
Ca21Chr1_0008910  0.89  0.49  0.53 -0.39 -0.60 -0.16 
Ca21Chr1_0008969  0.40  0.53  0.65 -0.38 -0.30 -0.35 
Ca21Chr1_0009028  0.16 -0.05  0.55 -0.90 -0.04 -0.38 
Ca21Chr1_0009087  0.45  0.58  0.77 -0.48 -0.43 -0.79 
Ca21Chr1_0009146  0.05 -0.29  0.21 -0.86 -0.04 -0.32 
Ca21Chr1_0009205 -0.00  0.05 -0.01 -0.71 -0.79 -0.74 
Ca21Chr1_0009264 -0.24 -0.19 -0.03 -0.35 -0.53 -0.56 
Ca21Chr1_0009323  0.20 -0.19  0.33 -0.83 -0.43 -0.54 
Ca21Chr1_0009382 -0.08  0.20  0.24 -1.42 -0.57  0.24 
Ca21Chr1_0009441  0.01  0.14  0.75 -0.71 -1.00  0.22 
Ca21Chr1_0009500  0.61  0.46  0.86 -0.83 -0.10 -0.24 
Ca21Chr1_0009559  0.24 -0.06  0.71 -0.13 -0.22 -0.33 
Ca21Chr1_0009618  0.08  0.22  0.65 -0.63 -0.45 -0.84 
Ca21Chr1_0009677  0.28  0.58  0.93 -0.66 -0.47 -0.66 
Ca21Chr1_0009736  0.87  0.92  0.69 -0.80 -0.01 -0.56 
Ca21Chr1_0009795  0.74  0.82  0.61 -0.64 -0.47 -0.29 
Ca21Chr1_0009854  0.06  1.05  0.09 -0.88 -0.87 -0.95 
Ca21Chr1_0009913  0.26  0.77  0.64 -0.37 -0.84 -0.57 
Ca21Chr1_0009972  0.33  0.50  0.55 -0.58 -0.26 -0.46 
Ca21Chr1_0010031 -0.07  0.55  0.32 -1.03 -1.02 -1.03 
Ca21Chr1_0010090 -0.24  0.23  0.10 -0.55 -0.79 -0.85 
Ca21Chr1_0010149 -0.51  0.37  0.30 -0.84 -0.29 -0.48 
Ca21Chr1_0010208 -0.79  0.46  0.12 -1.30 -1.01 -0.94 
Ca21Chr1_0010267  0.34  0.54  0.04 -1.04 -1.44 -0.96 
Ca21Chr1_0010326 -0.16  0.14  0.28 -2.30 -2.62 -1.37 
Ca21Chr1_0010385 -0.09  0.02 -0.14 -2.47 -2.45 -1.99 
Ca21Chr1_0010444  0.11  0.06 -0.54 -0.40 -2.87 -2.19 
Ca21Chr1_0010503 -0.92  0.24  0.11 -1.67 -3.07 -2.51 
Ca21Chr1_0010562 -0.00  0.64  0.03 -1.79 -2.62 -1.87 
Ca21Chr1_0010621  0.03  0.62 -0.24 -2.35 -2.08 -1.86 
Ca21Chr1_0010680 -0.10  0.36  0.10 -2.81 -1.32 -1.25  orf19.6112>
Ca21Chr1_0010739  0.26  1.11  0.54 -1.38 -1.51 -0.70 
Ca21Chr1_0010798  0.17  0.35  0.01 -0.40 -1.72 -0.78 
Ca21Chr1_0010857  0.05  0.03  0.15 -0.40 -1.30 -1.06 
Ca21Chr1_0010916  0.03  0.35 -0.03 -0.33 -1.64 -0.82 
Ca21Chr1_0010975 -0.16 -0.24 -0.15 -0.70 -1.22 -1.22 
Ca21Chr1_0011034  0.31  0.34 -0.30 -0.73 -0.95 -0.54 
Ca21Chr1_0011093 -0.40  0.23 -0.24 -0.23 -0.90 -0.53 
Ca21Chr1_0011152  0.04  0.23 -0.04 -0.29 -0.01 -0.66 
Ca21Chr1_0011211  0.52 -0.01  0.56 -0.43  0.24 -0.04 
Ca21Chr1_0011270 -0.48  0.46  0.45  0.68 -0.19 -0.47 
Ca21Chr1_0011329 -0.41  0.24  0.37 -0.25 -0.61 -0.23 
Ca21Chr1_0011388  0.45  0.40  0.34  0.23 -0.44 -0.24 
Ca21Chr1_0011447  0.28  0.70  0.59 -0.15  0.06 -0.21  orf19.6112|
Ca21Chr1_0011506  0.12  0.18  0.10 -0.58 -0.38 -0.54 
Ca21Chr1_0011565  0.49  0.59  0.87 -0.65  0.02 -0.16 
Ca21Chr1_0011624  0.22  0.80  0.60 -0.32 -0.15 -0.25  |orf19.6110
Ca21Chr1_0011683  0.24  0.23  0.59 -0.92 -0.07 -0.49 
Ca21Chr1_0011742 -0.11  0.90  0.34 -0.76 -0.14 -0.62 
Ca21Chr1_0011801  0.02  0.18  0.10 -1.13 -0.47 -0.44 
Ca21Chr1_0011860 -0.13  0.05 -0.16 -0.59 -0.36 -0.42 
Ca21Chr1_0011919 -0.35 -0.29 -0.73 -0.43 -0.79 -0.67 
Ca21Chr1_0011978  0.24 -0.27 -0.32 -1.21 -0.62 -0.27  <orf19.6110
Ca21Chr1_0012037 -0.55 -0.73 -0.59 -0.87 -0.67 -0.23 
Ca21Chr1_0012096 -0.03 -0.30  0.14  1.31 -0.11 -0.32 
Ca21Chr1_0012155  0.14 -0.16 -0.35 -0.36 -0.28 -0.23  orf19.6109>
Ca21Chr1_0012214 -0.78  0.00 -0.12 -0.22 -1.01 -0.21 
Ca21Chr1_0012273 -0.07 -0.01 -0.09 -0.59 -0.28 -0.55 
Ca21Chr1_0012332 -0.19 -0.45 -0.53 -0.76  0.26 -0.63 
Ca21Chr1_0012391 -0.20 -0.19  0.30 -0.50  0.16 -0.49 
Ca21Chr1_0012450  0.27  0.17  0.22  0.46  0.19  0.22 
Ca21Chr1_0012509 -0.51 -0.82 -0.07 -0.25  0.25  0.17 
Ca21Chr1_0012568 -0.01  0.24  0.86  0.03 -0.09 -0.09 
Ca21Chr1_0012627  0.13 -0.13  0.36 -0.60 -0.07 -0.26 
Ca21Chr1_0012686  0.07  0.08  0.28  0.22  0.05 -1.19 
Ca21Chr1_0012745  0.22 -0.25  0.34 -0.51  0.09 -0.69 
Ca21Chr1_0012804  0.22 -0.05  0.17 -0.41 -0.00 -0.55 
Ca21Chr1_0012863 -0.00  0.17  0.14  0.26 -0.02 -0.99 
Ca21Chr1_0012922 -0.22  0.08  0.04 -0.56 -0.16 -0.26 
Ca21Chr1_0012981  0.14  0.11  0.36 -0.24 -0.08 -0.58 
Ca21Chr1_0013040  0.12  0.65  0.53 -0.51 -0.03 -0.15 
Ca21Chr1_0013099 -0.24  0.66  0.42 -0.88 -0.02 -0.34 
Ca21Chr1_0013158 -0.01  0.25  0.54 -1.04  0.02 -0.60 
Ca21Chr1_0013217 -0.02  0.13  0.23 -0.96 -0.23 -0.49 
Ca21Chr1_0013276  0.08  0.33  0.29 -0.92  0.30 -0.71 
Ca21Chr1_0013335 -0.17  0.05  0.18 -0.89 -0.01 -0.60 
Ca21Chr1_0013394  0.01  0.19  0.21 -0.71 -0.09 -0.77 
Ca21Chr1_0013453  0.05  0.91  0.05 -0.76  0.16 -0.67 
Ca21Chr1_0013512 -0.01  0.38  0.32 -1.01  0.11 -0.03 
Ca21Chr1_0013571  0.27 -0.14  0.04 -0.68 -0.01  0.13 
Ca21Chr1_0013630 -0.24 -0.16 -0.35 -0.48 -0.39 -0.38 
Ca21Chr1_0013689 -0.60 -0.40 -0.82 -0.37  0.44 -0.18  orf19.6109|
Ca21Chr1_0013748 -0.37 -0.25 -0.59 -0.42 -0.33 -0.47  orf19.6105>
Ca21Chr1_0013807 -0.38 -0.53 -0.39 -0.34 -0.04 -0.29 
Ca21Chr1_0013866  0.03 -0.04 -0.24 -0.82 -0.11 -0.28 
Ca21Chr1_0013925 -0.10 -0.16 -0.68 -0.78 -0.26 -0.20 
Ca21Chr1_0013984 -0.30 -0.39 -0.35 -0.66 -0.20 -0.97 
Ca21Chr1_0014043  0.13 -0.20 -0.08 -1.03  0.09 -0.28 
Ca21Chr1_0014102 -0.53 -0.05  0.17 -0.78 -0.10 -0.69 
Ca21Chr1_0014161  0.20 -0.17  0.11 -0.78 -0.24 -0.21 
Ca21Chr1_0014220  0.10 -0.01  0.06 -0.30 -0.40 -0.56 
Ca21Chr1_0014279  0.00  0.11  0.44 -0.66  0.04 -0.42 
Ca21Chr1_0014338  0.07 -0.12  0.19 -0.21  0.10 -0.54 
Ca21Chr1_0014397  0.06  0.27  0.22 -0.13 -0.09 -0.51 
Ca21Chr1_0014456 -0.07  0.35  0.81 -0.20  0.27 -0.52 
Ca21Chr1_0014515  0.12  0.45  0.42 -0.24  0.20 -0.38 
Ca21Chr1_0014574  0.24  0.33  0.51 -0.48 -0.13 -0.44 
Ca21Chr1_0014633 -0.16 -0.18  0.14 -0.17 -0.92 -0.51 
Ca21Chr1_0014692  0.63  0.79  0.12 -0.27 -0.42 -0.51 
Ca21Chr1_0014751 -0.18 -0.03  0.37 -0.03  0.20 -0.44 
Ca21Chr1_0014810  0.14  0.44  0.56 -0.07 -0.05 -0.01 
Ca21Chr1_0014869 -0.15  0.25  0.24 -0.17  0.05 -0.26  |orf19.6102 orf19.6105|
Ca21Chr1_0014928  0.31  0.08  0.20 -0.38 -0.32 -0.21 
Ca21Chr1_0014987  0.35  0.44 -0.08 -0.40 -0.19 -0.22 
Ca21Chr1_0015046  0.17  0.01  0.42 -0.59  0.18  0.08 
Ca21Chr1_0015105  0.48  0.12  1.01 -0.80  0.16  0.21 
Ca21Chr1_0015164 -0.60  0.04  0.01 -0.29  0.24 -0.32 
Ca21Chr1_0015223 -0.24  0.62 -0.27  0.36  0.30 -0.16 
Ca21Chr1_0015282  0.17 -0.22  0.65 -0.44 -0.15 -0.32 
Ca21Chr1_0015341 -0.11 -0.26 -0.25 -0.08  0.23 -0.65 
Ca21Chr1_0015400 -0.08  0.03 -0.41 -0.28 -0.05 -0.64 
Ca21Chr1_0015459  0.14 -0.04 -0.56 -0.05 -0.47 -0.35 
Ca21Chr1_0015518 -0.14  0.05 -0.31 -0.44 -0.23 -0.50 
Ca21Chr1_0015577 -0.63 -0.16 -0.95 -0.18 -0.19 -0.25  orf19.6103>
Ca21Chr1_0015636 -0.68 -0.77 -0.94 -0.31 -0.38 -0.12 
Ca21Chr1_0015695 -0.28 -0.61 -0.35  0.07  0.27 -0.25 
Ca21Chr1_0015754 -0.21 -0.47 -0.67 -0.40  0.39 -0.52  <orf19.6102
Ca21Chr1_0015813  0.09 -0.49 -0.42 -0.74  0.06 -0.56 
Ca21Chr1_0015872  0.01 -0.22 -0.00 -0.01 -0.33 -0.39 
Ca21Chr1_0015931  0.06 -0.13 -0.23 -0.45 -0.14  0.06 
Ca21Chr1_0015990  0.07 -0.22 -0.17 -0.34 -0.09 -0.32 
Ca21Chr1_0016049 -0.21 -0.05  0.14  0.04 -0.11 -0.19 
Ca21Chr1_0016108  0.05  0.50  0.05 -0.15 -0.06 -0.58  orf19.6103|
Ca21Chr1_0016167  0.50  0.36  0.38 -0.06 -0.54 -0.40 
Ca21Chr1_0016226  0.10  0.40  0.18  0.03  0.05 -0.68 
Ca21Chr1_0016285  0.06  0.22  0.60  0.48  0.18 -0.31 
Ca21Chr1_0016344  0.15  0.14  0.27  0.14  0.23 -0.33 
Ca21Chr1_0016403  0.19  0.10  0.17 -0.35  0.31 -0.22 
Ca21Chr1_0016462  1.05 -0.01  0.59  0.11 -0.35  0.49 
Ca21Chr1_0016521  0.38  0.17  0.61 -0.04  0.09 -0.06 
Ca21Chr1_0016580  0.21  0.49  0.60  0.12 -0.01 -0.05  |orf19.6100
Ca21Chr1_0016639  1.49 -0.07  0.38  0.23  0.24  0.35 
Ca21Chr1_0016698  0.23  0.26  0.33  0.11  0.14 -0.52 
Ca21Chr1_0016757  0.17  0.57  0.39  0.06  0.25 -0.75 
Ca21Chr1_0016816  0.04  0.19  0.29 -0.01  0.16 -0.69 
Ca21Chr1_0016875  0.04  0.36  0.24 -0.13  0.01 -0.54 
Ca21Chr1_0016934  0.21  0.54  0.65 -0.05  0.19 -0.52 
Ca21Chr1_0016993 -0.58  0.29  0.22 -0.01  0.24 -0.48 
Ca21Chr1_0017052  0.12 -0.07  0.31 -0.07 -0.11 -0.43 
Ca21Chr1_0017111 -0.01  0.15  0.15  0.47  0.34 -0.58 
Ca21Chr1_0017170 -0.06  0.25  0.48 -0.03 -0.15 -0.26 
Ca21Chr1_0017229  0.02  0.12  0.38  0.09  0.01 -0.45 
Ca21Chr1_0017288  0.05 -0.17  0.38  0.16 -0.38  0.02  <orf19.6100 orf19.6099>
Ca21Chr1_0017347  0.32  0.07 -0.12 -0.08  0.02 -0.31 
Ca21Chr1_0017406  0.20  0.43  0.28 -0.02  0.20 -0.31 
Ca21Chr1_0017465  0.22 -0.20  0.18 -0.11 -0.04 -0.42 
Ca21Chr1_0017524  0.95  0.05  0.62  0.78  0.29  0.19 
Ca21Chr1_0017583  0.14  0.12  0.35  0.01  0.06 -0.32 
Ca21Chr1_0017642  0.55 -0.02  0.17  0.08  0.04  0.32 
Ca21Chr1_0017701 -0.09 -0.04 -0.09 -0.29  0.06 -0.49 
Ca21Chr1_0017760  0.15 -0.11  0.53 -0.37  0.06 -0.35 
Ca21Chr1_0017819  0.27  0.07  0.43  0.12  0.18 -0.39 
Ca21Chr1_0017878  0.21  0.30  0.54 -0.46  0.19 -0.68 
Ca21Chr1_0017937  0.25  0.17  0.57 -0.27  0.05 -0.32 
Ca21Chr1_0017996 -0.05  0.29  0.26  0.19  0.33 -0.52 
Ca21Chr1_0018055 -0.36 -0.11 -0.13 -0.37  0.11 -0.30 
Ca21Chr1_0018114  0.11  0.02 -0.02 -0.25  0.49 -0.16 
Ca21Chr1_0018173 -1.07 -0.38  0.13 -0.25  0.17 -0.24 
Ca21Chr1_0018232 -0.63 -0.57  0.08 -0.06 -0.05 -0.27 
Ca21Chr1_0018291 -0.78 -0.94 -0.47 -0.16  0.23 -0.03 
Ca21Chr1_0018350 -0.79 -0.51 -0.39  0.08  0.26 -0.13 
Ca21Chr1_0018409 -0.17 -0.47  0.23  0.07  0.32 -0.32 
Ca21Chr1_0018468 -0.16 -0.12  0.27  0.01  0.48 -0.41 
Ca21Chr1_0018527 -0.21  0.27  0.34 -0.29  0.26 -0.10 
Ca21Chr1_0018586  0.03  0.11  0.54 -0.27 -0.06 -0.27 
Ca21Chr1_0018645  0.21 -0.12  0.26 -0.81  0.12 -0.48 
Ca21Chr1_0018704 -0.26 -0.05 -0.24  0.01 -0.17  0.25 
Ca21Chr1_0018763 -0.15  0.06  0.31 -0.13  0.50 -0.20 
Ca21Chr1_0018822  0.06 -0.07  0.16  0.14 -0.07 -0.22 
Ca21Chr1_0018881  0.22  0.11  0.02 -0.19 -0.37 -0.15 
Ca21Chr1_0018940 -0.07  0.21  0.44 -0.25  0.54 -0.04  |orf19.6096 orf19.6099|
Ca21Chr1_0018999  0.21  0.06  0.33 -0.38  0.13  0.09 
Ca21Chr1_0019058  0.23  0.28  0.47  0.09  0.14  0.03 
Ca21Chr1_0019117  0.04 -0.03  0.72  0.14  0.08  0.10 
Ca21Chr1_0019176  0.20  0.09  0.55 -0.23  0.11 -0.29 
Ca21Chr1_0019235 -0.20  0.33  0.42  0.42  0.10 -0.24 
Ca21Chr1_0019294  0.22  0.06  0.25 -0.11  0.11 -0.40 
Ca21Chr1_0019353  0.17  0.26  0.29 -0.62  0.05 -0.61 
Ca21Chr1_0019412  0.04 -0.07  0.22 -0.23 -0.05 -0.61 
Ca21Chr1_0019471 -0.02  0.21  0.23 -0.38 -0.08 -0.15 
Ca21Chr1_0019530  0.20 -0.08 -0.08 -0.18 -0.04  0.20 
Ca21Chr1_0019589 -0.80  0.06 -0.97  0.29  0.12 -0.20  |orf19.6094 <orf19.6096
Ca21Chr1_0019648 -1.02 -1.05 -0.81 -0.35 -0.04 -0.17 
Ca21Chr1_0019707 -0.76 -0.80 -1.02 -0.59  0.05 -0.24 
Ca21Chr1_0019766 -1.04 -0.52 -0.76  0.18 -0.54 -0.19 
Ca21Chr1_0019825 -0.47 -0.41 -0.12 -0.16 -0.26 -0.32 
Ca21Chr1_0019884 -0.28 -0.34 -0.37 -0.34 -0.01 -0.52 
Ca21Chr1_0019943 -0.32  0.13  0.07 -0.06  0.14 -0.43 
Ca21Chr1_0020002  0.26 -0.18  0.36 -0.19  0.35 -0.67 
Ca21Chr1_0020061  0.16  0.33  0.36  0.24  0.52 -0.66 
Ca21Chr1_0020120 -0.16  0.11  0.50 -0.25 -0.36 -0.66 
Ca21Chr1_0020179  0.18  0.23  0.70 -0.06 -0.43 -0.07 
Ca21Chr1_0020238  0.63  0.15  0.31 -0.32  0.20 -0.49 
Ca21Chr1_0020297  0.33  0.26  0.13 -0.06  0.29 -0.41 
Ca21Chr1_0020356  0.02  0.35  0.16 -0.18  0.04 -0.74 
Ca21Chr1_0020415  1.49  0.27  0.50 -0.05  0.32 -0.28 
Ca21Chr1_0020474  0.23 -0.03  0.39  0.13 -0.09 -0.21 
Ca21Chr1_0020533  0.24  0.25  0.25 -0.10  0.23  0.13 
Ca21Chr1_0020592  0.39  0.84  0.57 -0.14  0.20 -0.24 
Ca21Chr1_0020651 -0.11  0.30  0.44 -0.06  0.14 -0.29 
Ca21Chr1_0020710 -0.20  0.26  0.56  0.22  0.21 -0.21 
Ca21Chr1_0020769  0.45 -0.21  0.63  0.32  0.03 -0.22 
Ca21Chr1_0020828  0.39  0.14 -0.06  0.24  0.30 -0.64 
Ca21Chr1_0020887  0.22  0.06  0.37  0.03  0.08 -0.13 
Ca21Chr1_0020946  0.48  0.26  0.87  0.29  0.20 -0.10 
Ca21Chr1_0021005  0.03  0.18  0.55  0.10  0.11 -0.25 
Ca21Chr1_0021064  0.14  0.14  0.84 -0.05 -0.08 -0.58 
Ca21Chr1_0021123  0.08  0.24  0.42  0.04 -0.10  0.32 
Ca21Chr1_0021182  0.21 -0.04  0.18  0.06 -0.39 -0.56 
Ca21Chr1_0021241  0.42  0.08  0.32 -0.15  0.04 -0.55 
Ca21Chr1_0021300 -0.00  0.40  0.46  0.48 -0.04 -0.45 
Ca21Chr1_0021359 -0.59  0.15  0.21 -0.11 -0.07 -0.80 
Ca21Chr1_0021418 -0.54  0.12  0.18  0.24  0.21 -0.30 
Ca21Chr1_0021477  0.41  0.11  0.50 -0.24 -0.09 -0.44 
Ca21Chr1_0021536 -0.10  0.00  0.43 -0.35  0.09 -0.31 
Ca21Chr1_0021595  0.13 -0.02  0.12 -0.29  0.03 -0.86 
Ca21Chr1_0021654  0.18  0.25  0.24  0.18  0.23  0.21 
Ca21Chr1_0021713    NA    NA    NA    NA    NA    NA <orf19.6094
Ca21Chr1_0021772  0.16  0.17  0.19  0.14  0.05  0.28 
Ca21Chr1_0021831  0.34 -0.08  0.18 -0.05 -0.06  0.08 
Ca21Chr1_0021890  0.52  0.18  0.43 -0.06  0.16 -0.19 
Ca21Chr1_0021949  0.15  0.28  0.20  0.32  0.12  0.43 
Ca21Chr1_0022008  0.38 -0.41 -0.39  0.27 -0.23  0.18 
Ca21Chr1_0022067  0.16 -0.23  0.14 -0.11 -0.06 -0.00 
Ca21Chr1_0022126  0.74 -0.07  0.28 -0.15  0.28  0.66 
Ca21Chr1_0022185 -0.06 -0.72 -0.06 -0.07  0.01 -0.23 
Ca21Chr1_0022244  0.87 -0.20  0.30  0.00 -0.11  0.11  orf19.6092>
Ca21Chr1_0022303  0.14  0.25  0.34 -0.06  0.10 -0.55 
Ca21Chr1_0022362 -0.12  0.27  0.25 -0.06  0.02 -0.40 
Ca21Chr1_0022421  0.26  0.42  0.34 -0.22  0.22 -0.31 
Ca21Chr1_0022480  0.14  0.25  0.23 -0.12  0.27 -0.37 
Ca21Chr1_0022539  1.20 -0.10 -0.15  0.11  0.04 -0.66 
Ca21Chr1_0022598  0.01  0.82  0.87 -0.01 -0.28 -0.51 
Ca21Chr1_0022657  0.66  0.14  0.68  0.54 -0.28  0.03 
Ca21Chr1_0022716  0.23 -0.05  0.34 -0.05  0.03 -0.53 
Ca21Chr1_0022775  0.05  0.16  0.50 -0.12 -0.14 -0.28 
Ca21Chr1_0022834  0.18  0.09  0.32  0.13 -0.22 -0.31 
Ca21Chr1_0022893  0.08  0.09  0.30  0.10  0.12 -0.31 
Ca21Chr1_0022952  0.04  0.05 -0.25 -0.00 -0.14 -0.26 
Ca21Chr1_0023011  0.00 -0.08 -0.09  0.11  0.51 -0.27 
Ca21Chr1_0023070  0.03 -0.12  0.16 -0.17 -0.03 -0.65 
Ca21Chr1_0023129  0.30 -0.02  0.43  0.33  0.30 -0.42 
Ca21Chr1_0023188 -0.03  1.11  0.55  0.30  0.22 -0.45 
Ca21Chr1_0023247  0.05  0.16  0.09 -0.07  0.19 -0.34 
Ca21Chr1_0023306  0.34  0.21  0.38 -0.11 -0.22 -0.30 
Ca21Chr1_0023365  0.18  0.04 -0.04 -0.28  0.55 -0.03 
Ca21Chr1_0023424  0.15  0.73  0.22  0.06  0.27 -0.23 
Ca21Chr1_0023483  0.02  0.67  0.18 -0.02  0.21 -0.35 
Ca21Chr1_0023542  0.18  0.41  0.36  0.04  0.33 -0.16 
Ca21Chr1_0023601 -0.49  0.27  0.13 -0.11  0.31 -0.32 
Ca21Chr1_0023660  0.70  0.09  0.53 -0.23  0.22 -0.08 
Ca21Chr1_0023719 -0.02  0.17  0.34  0.33  0.55 -0.37 
Ca21Chr1_0023778 -0.51  0.03 -0.06 -0.00  0.25 -0.55 
Ca21Chr1_0023837 -0.05  0.13  0.13  0.12 -0.20 -0.39 
Ca21Chr1_0023896 -0.20  0.54 -0.26  0.13  0.72 -0.56 
Ca21Chr1_0023955 -0.13  0.37  0.06  0.25  0.16 -0.22 
Ca21Chr1_0024014 -0.53 -0.52 -0.41 -0.10  0.22 -0.19 
Ca21Chr1_0024073 -0.34 -0.38 -0.38  0.15  0.43 -0.21 
Ca21Chr1_0024132 -0.23 -0.21 -0.12  0.38  0.10 -0.06 
Ca21Chr1_0024191 -0.75 -0.40 -0.18  0.23  0.12 -0.28 
Ca21Chr1_0024250  0.12  0.06  0.20  0.25  0.14 -0.19 
Ca21Chr1_0024309 -0.07  0.03  0.11 -0.29  0.47 -0.65 
Ca21Chr1_0024368  0.04  0.01  0.32  0.18 -0.61 -0.43 
Ca21Chr1_0024427 -0.08 -0.09  0.04  0.28 -0.21 -0.73 
Ca21Chr1_0024486  0.44  0.12  0.07  0.40  0.06 -0.44 
Ca21Chr1_0024545 -0.40  0.01 -0.10  0.19  0.13 -0.80 
Ca21Chr1_0024604 -0.72  0.19 -0.37 -0.09 -0.27 -0.67 
Ca21Chr1_0024663 -0.41 -0.38 -0.20 -0.11  0.09 -0.31 
Ca21Chr1_0024722 -0.39 -0.32  0.17 -0.43  0.10 -0.28 
Ca21Chr1_0024781 -0.61 -0.30 -0.46  0.29  0.16 -0.24 
Ca21Chr1_0024840 -0.70 -0.74 -1.01 -0.36  0.32 -0.97 
Ca21Chr1_0024899 -0.32 -0.65 -0.67  0.25 -0.00 -0.21 
Ca21Chr1_0024958 -1.32 -0.90 -0.65  0.04 -0.37 -0.34 
Ca21Chr1_0025017 -1.75 -1.03 -1.12  0.03  0.04 -0.37 
Ca21Chr1_0025076 -0.76 -0.64 -0.41 -0.15  0.24 -0.68 
Ca21Chr1_0025135  0.26  0.20  0.40  0.27  0.44 -0.22 
Ca21Chr1_0025194 -1.10 -0.93 -0.89  0.33  0.14 -0.63 
Ca21Chr1_0025253  0.15 -0.79 -0.98  0.63  0.37 -0.33 
Ca21Chr1_0025312 -0.54 -0.44 -0.33  0.22  0.25 -0.47  orf19.6092|
Ca21Chr1_0025371 -0.53 -0.29 -0.89 -0.08 -0.43 -0.62  |orf19.6091
Ca21Chr1_0025430 -0.53 -0.50 -0.84  0.38  0.11 -0.29 
Ca21Chr1_0025489 -0.63  0.13 -0.47  0.19  0.20 -0.23 
Ca21Chr1_0025548 -1.13 -0.57 -0.96 -0.07 -0.19 -0.35 
Ca21Chr1_0025607 -0.80 -0.64 -0.68 -0.94  0.18 -1.01 
Ca21Chr1_0025666 -0.39 -0.08 -0.47 -0.15  0.14 -0.30 
Ca21Chr1_0025725 -0.12 -0.57 -0.19 -0.11 -0.11 -0.51 
Ca21Chr1_0025784  0.17  0.10  0.55 -0.37 -0.07 -0.26 
Ca21Chr1_0025843  0.33  0.42  0.25 -0.01  0.29 -0.18 
Ca21Chr1_0025902 -0.16  0.21 -0.02 -0.15  0.06 -0.28 
Ca21Chr1_0025961 -0.45 -0.36  0.00 -0.52 -0.16 -0.20 
Ca21Chr1_0026020 -0.32  0.21  0.04 -0.19 -0.22 -0.30 
Ca21Chr1_0026079  0.20  0.21  0.21 -0.19  0.13  0.05 
Ca21Chr1_0026138  0.36  0.30  0.41  0.18  0.65 -0.07 
Ca21Chr1_0026197 -0.15  0.19  0.12 -0.24  0.06 -0.57 
Ca21Chr1_0026256  0.09  0.13  0.40 -0.12  0.23 -0.40 
Ca21Chr1_0026315 -0.08  0.45  0.47  0.18 -0.18 -0.43 
Ca21Chr1_0026374  0.03  0.14  0.49  0.12  0.07 -0.48 
Ca21Chr1_0026433  0.12  0.32  0.78 -0.32  0.30 -0.16 
Ca21Chr1_0026492  1.34  0.15  0.37 -0.09  0.43 -0.50 
Ca21Chr1_0026551  0.21  0.18  0.23 -0.38  0.45 -0.21 
Ca21Chr1_0026610  0.06  0.26  0.50 -0.02 -0.02 -0.57 
Ca21Chr1_0026669  0.15  0.30  0.33 -0.25  0.35 -0.43 
Ca21Chr1_0026728  0.25  0.37  0.39 -0.39  0.17 -0.17 
Ca21Chr1_0026787  0.02  0.29  0.43 -0.14  0.28 -0.09 
Ca21Chr1_0026846  0.01  0.47  0.23 -0.01  0.03 -0.04 
Ca21Chr1_0026905 -0.00  0.06  0.31  0.10 -0.00 -0.12 
Ca21Chr1_0026964  0.06  0.24  0.11 -0.12 -0.19 -0.29 
Ca21Chr1_0027023 -0.11  0.42  0.11 -0.14 -0.23 -0.22 
Ca21Chr1_0027082  0.01  0.08 -0.01 -0.28 -0.06 -0.34 
Ca21Chr1_0027141 -0.18 -0.16  0.08  0.16 -0.06 -0.21 
Ca21Chr1_0027200 -0.29  0.00  0.03  0.02 -0.09 -0.16  <orf19.6091
Ca21Chr1_0027259 -1.27 -0.28 -0.31  0.25  0.20 -0.05 
Ca21Chr1_0027318    NA    NA    NA    NA    NA    NA
Ca21Chr1_0027377 -1.59 -1.35 -0.79 -0.04  0.13 -0.21 
Ca21Chr1_0027436 -1.94 -1.94 -1.23 -0.00 -0.25 -0.16 
Ca21Chr1_0027495 -2.20 -1.65 -1.28  0.13  0.12 -0.07 
Ca21Chr1_0027554 -1.55 -1.86 -1.04 -0.39 -0.02 -0.61 
Ca21Chr1_0027613 -0.90 -1.52 -0.68 -0.22 -0.04 -0.31 
Ca21Chr1_0027672 -0.52 -1.30 -0.59 -0.22  0.03 -0.27 
Ca21Chr1_0027731 -1.18 -0.84  0.03  0.06 -0.25 -0.03 
Ca21Chr1_0027790    NA    NA    NA    NA    NA    NA
Ca21Chr1_0027849 -1.27 -0.47 -0.80 -0.27 -0.06  0.05 
Ca21Chr1_0027908 -1.35 -1.08 -0.83 -0.18 -0.34  0.03 
Ca21Chr1_0027967 -1.24 -1.03 -0.66 -0.05  0.01 -0.33 
Ca21Chr1_0028026 -0.80 -0.46 -0.67  0.08 -0.05  0.35 
Ca21Chr1_0028085 -0.74 -1.19 -0.18  0.01  0.03 -0.31 
Ca21Chr1_0028144 -0.56 -1.28 -0.46 -0.27 -0.17 -0.22 
Ca21Chr1_0028203  0.90 -0.52 -0.28  0.14 -0.06 -0.11 
Ca21Chr1_0028262  0.54 -0.16  0.25 -0.02  0.30 -0.87  |orf19.6090
Ca21Chr1_0028321  0.85  0.07  0.03 -0.21  0.66 -0.13 
Ca21Chr1_0028380  0.72  0.14  0.61 -0.37  0.05  0.45 
Ca21Chr1_0028439  0.45  0.73  0.38  0.07 -0.08 -0.30 
Ca21Chr1_0028498  0.66 -0.19  0.25 -0.36 -0.02 -0.39 
Ca21Chr1_0028557  0.70 -0.09  0.07 -0.12 -0.01 -0.63 
Ca21Chr1_0028616  0.64  0.05  0.22 -0.84  0.06  0.61 
Ca21Chr1_0028675  0.49  0.38  0.23  0.16  0.15 -0.25 
Ca21Chr1_0028734  0.57  0.17  0.38  0.17  0.38 -0.10 
Ca21Chr1_0028793  0.80 -0.12  0.18 -0.02  0.05 -0.16 
Ca21Chr1_0028852  0.69  0.23  0.68  0.15  0.45 -0.05 
Ca21Chr1_0028911  0.57  0.14  0.18 -0.11  0.41 -0.31 
Ca21Chr1_0028970  1.00  0.33  0.44  0.18 -0.21  0.10 
Ca21Chr1_0029029  0.63  0.44  0.26 -0.32  0.45  0.05 
Ca21Chr1_0029088  0.94  1.16  0.56  0.34  0.49  0.08 
Ca21Chr1_0029147  0.90  0.21  0.46 -0.17  0.44  0.22 
Ca21Chr1_0029206  1.10  0.03  0.48 -0.07  0.44  0.25 
Ca21Chr1_0029265  1.07  0.06  0.20 -0.00  0.03  0.00 
Ca21Chr1_0029324  0.91  0.39  0.41  0.56  0.31  0.01 
Ca21Chr1_0029383  0.03  0.36 -0.24 -0.38  0.10 -0.08 
Ca21Chr1_0029442  0.15 -0.16 -1.22 -0.25 -0.09 -0.10  <orf19.6090
Ca21Chr1_0029501 -1.00 -0.86 -1.39  0.71 -0.07 -0.44 
Ca21Chr1_0029560 -2.59 -1.43 -1.16 -0.21 -0.21  0.12 
Ca21Chr1_0029619 -2.07 -0.63 -0.98 -0.69  0.30  0.30 
Ca21Chr1_0029678 -1.60 -0.99 -0.62 -0.30 -0.20 -0.14 
Ca21Chr1_0029737 -1.41 -1.39 -1.47 -0.47 -0.21  0.04 
Ca21Chr1_0029796 -0.61 -1.17 -0.45 -0.02 -0.20  0.16  orf19.6086>
Ca21Chr1_0029855 -0.21 -0.90 -0.79 -0.41 -0.25 -0.18 
Ca21Chr1_0029914 -0.24 -0.27 -0.16 -0.07 -0.20 -0.34 
Ca21Chr1_0029973 -0.15 -0.52 -0.12  0.11  0.04 -0.01 
Ca21Chr1_0030032  0.10 -0.12 -0.05 -0.17  0.23  0.22 
Ca21Chr1_0030091 -0.11 -0.19  0.28 -0.30 -0.12  0.07 
Ca21Chr1_0030150 -0.02 -0.12  0.27  0.02  0.15 -0.33 
Ca21Chr1_0030209 -0.35 -0.11  0.13 -0.06  0.08 -0.44 
Ca21Chr1_0030268 -0.04 -0.02  0.11 -0.12 -0.10 -0.30 
Ca21Chr1_0030327 -0.03  0.03  0.03 -0.08  0.19  0.18 
Ca21Chr1_0030386  0.30  0.05  0.26  0.19  0.16 -0.15 
Ca21Chr1_0030445  0.20  0.06  0.68  0.12  0.41 -0.50 
Ca21Chr1_0030504 -0.10  0.26  0.16 -0.13 -0.02 -0.72 
Ca21Chr1_0030563  0.14  0.18  0.02  0.12 -0.06 -0.80 
Ca21Chr1_0030622  0.24  0.29  0.37 -0.42  0.11 -0.48 
Ca21Chr1_0030681  0.09  0.28  0.32  0.12  0.20 -0.16 
Ca21Chr1_0030740  0.05  0.00  0.39 -0.12  0.31 -0.24 
Ca21Chr1_0030799 -0.42  0.25  0.05  0.26 -0.16 -0.38 
Ca21Chr1_0030858 -0.23  0.01  0.61 -0.04  0.26 -0.19 
Ca21Chr1_0030917  0.05 -0.31  0.07 -0.07  0.06 -0.48 
Ca21Chr1_0030976 -0.36  0.11  0.40  0.57  0.31 -0.68 
Ca21Chr1_0031035  0.00  0.00  0.37  0.08 -0.01 -0.58 
Ca21Chr1_0031094 -0.20  0.03  0.25  0.01  0.14 -0.46 
Ca21Chr1_0031153 -0.10 -0.07  0.10  0.20  0.16 -0.18 
Ca21Chr1_0031212 -0.26  0.13 -0.03 -0.22 -0.45 -0.16 
Ca21Chr1_0031271 -0.23 -0.11 -0.02 -0.08 -0.14 -0.31 
Ca21Chr1_0031330 -0.81  0.09 -0.38 -0.02 -0.25 -0.31 
Ca21Chr1_0031389 -0.37 -0.62 -0.76  0.73 -0.00 -0.37 
Ca21Chr1_0031448 -0.91 -0.20 -0.47 -0.19 -0.12  0.07 
Ca21Chr1_0031507 -1.94 -1.01 -1.15 -0.81 -0.63 -0.18  orf19.6086|
Ca21Chr1_0031566 -2.11 -1.27 -2.94 -1.33 -0.11 -0.46 
Ca21Chr1_0031625 -2.18 -2.09 -1.96 -1.38 -0.37 -0.23 
Ca21Chr1_0031684 -3.06 -1.55 -2.18 -1.34 -0.63 -0.64 
Ca21Chr1_0031743    NA    NA    NA    NA    NA    NA
Ca21Chr1_0031802 -0.19 -1.46 -1.40 -0.40 -0.23  0.30  orf19.6085>
Ca21Chr1_0031861 -0.30 -0.87 -0.13 -0.69 -0.62  0.21 
Ca21Chr1_0031920 -0.26 -0.47 -0.61 -0.31  0.27  0.39 
Ca21Chr1_0031979 -0.30 -0.28 -0.16 -0.12  0.14 -0.11 
Ca21Chr1_0032038 -0.14 -0.14  0.00 -0.15  0.07  0.07 
Ca21Chr1_0032097  0.33 -0.27  0.03 -0.20  0.33 -0.40 
Ca21Chr1_0032156  0.30 -0.39  0.06  0.02  0.22 -0.18 
Ca21Chr1_0032215 -0.79 -0.16  0.09  0.28 -0.02 -0.64 
Ca21Chr1_0032274 -0.33 -0.39  0.03 -0.17 -0.09 -0.79 
Ca21Chr1_0032333  0.11 -0.08  0.18  0.73  0.07 -0.23 
Ca21Chr1_0032392  0.08 -0.11  0.16  0.31  0.24 -0.14  orf19.6085|
Ca21Chr1_0032451 -0.44 -0.03 -0.33  0.23 -0.00 -0.77 
Ca21Chr1_0032510 -0.91 -0.26 -0.72  0.12  0.16 -0.14 
Ca21Chr1_0032569 -1.81 -0.21 -0.70 -0.20 -0.23 -0.05 
Ca21Chr1_0032628 -1.29  0.03 -0.98 -0.16  0.19  0.01  |orf19.6084
Ca21Chr1_0032687 -1.39 -0.31 -0.94 -0.15 -0.45  0.08 
Ca21Chr1_0032746 -0.46 -0.58 -0.97 -0.09 -0.14 -0.07 
Ca21Chr1_0032805 -0.93 -0.17 -0.75 -0.06 -0.12 -0.07 
Ca21Chr1_0032864 -1.02 -0.26 -0.69 -0.09  0.10 -0.41 
Ca21Chr1_0032923 -1.01 -0.56 -0.37 -0.01 -0.17  0.15 
Ca21Chr1_0032982 -0.18 -0.40 -0.32  0.22 -0.28  0.01 
Ca21Chr1_0033041 -0.08 -0.35  0.19  0.00 -0.16  0.08 
Ca21Chr1_0033100 -0.08  0.01 -0.12  0.23  0.13 -0.13 
Ca21Chr1_0033159  1.23  0.27 -0.04 -0.01 -0.07 -0.56 
Ca21Chr1_0033218 -0.02  0.23 -0.13 -0.10 -0.11 -0.17 
Ca21Chr1_0033277  0.18  0.04  0.25  0.25  0.05 -0.17  <orf19.6084
Ca21Chr1_0033336 -0.05 -0.29 -0.07  0.21 -0.14 -0.36 
Ca21Chr1_0033395 -0.09 -0.39 -0.17  0.10  0.24 -0.02 
Ca21Chr1_0033454  0.21 -0.19 -0.35  0.11  0.29 -0.11 
Ca21Chr1_0033513  0.21 -0.21 -0.28  0.11  0.03 -0.03 
Ca21Chr1_0033572  0.15 -0.07  0.16 -0.07 -0.04  0.14 
Ca21Chr1_0033631  0.82  0.04 -0.00 -0.17 -0.10  0.52 
Ca21Chr1_0033690  0.36 -0.18  0.05 -0.63 -0.22  0.03 
Ca21Chr1_0033749  0.53 -0.03 -0.09 -0.15 -0.36 -0.15 
Ca21Chr1_0033808 -0.16 -0.43 -0.18 -0.27  0.06  0.13 
Ca21Chr1_0033867 -0.35 -0.12 -0.45 -0.18 -0.26 -0.33 
Ca21Chr1_0033926 -0.16  0.27 -0.33 -0.49  0.03  0.09 
Ca21Chr1_0033985 -0.34 -0.36 -0.80 -0.32 -0.06 -0.32 
Ca21Chr1_0034044 -0.37 -0.49 -0.78 -0.16 -0.27 -0.04  |orf19.6083
Ca21Chr1_0034103 -0.40 -0.90 -0.68 -0.12 -0.46 -0.49 
Ca21Chr1_0034162 -0.46 -0.77 -0.36 -0.07 -0.32 -0.25 
Ca21Chr1_0034221 -0.13 -0.24 -0.87 -0.33 -0.64 -0.54 
Ca21Chr1_0034280 -0.11 -0.54 -0.45 -0.11 -0.47 -0.09 
Ca21Chr1_0034339 -0.01 -0.60 -0.84  0.20 -0.35 -0.41 
Ca21Chr1_0034398 -0.32 -0.28 -0.07 -0.03  0.03 -0.83  <orf19.6083
Ca21Chr1_0034457  0.38  0.22  0.23  0.04  0.45  0.22 
Ca21Chr1_0034516  0.32  0.02  0.08 -0.05  0.27  0.47  |orf19.6082
Ca21Chr1_0034575  0.23 -0.41 -0.15 -0.18  0.12  0.03 
Ca21Chr1_0034634  0.16 -0.29  0.15 -0.22 -0.48 -0.25 
Ca21Chr1_0034693 -0.06 -0.10  0.28 -0.13 -0.28  0.15 
Ca21Chr1_0034752  0.29 -0.30 -0.20 -0.58  0.11 -0.06 
Ca21Chr1_0034811  0.48 -0.14 -0.37 -0.66 -0.54  0.57 
Ca21Chr1_0034870  0.01 -0.36  0.09 -0.38 -0.00 -0.08 
Ca21Chr1_0034929  0.15 -0.22  0.18 -0.50 -0.10 -0.10 
Ca21Chr1_0034988  0.08 -0.30 -0.52 -0.61 -0.23  0.06 
Ca21Chr1_0035047  0.07 -0.33 -0.12 -0.09  0.10 -0.34 
Ca21Chr1_0035106  0.05  0.05  0.06 -0.16 -0.18  0.07 
Ca21Chr1_0035165  0.23  0.23  0.38 -0.24 -0.11 -0.33 
Ca21Chr1_0035224  0.01  0.14  0.09 -0.24 -0.31 -0.29 
Ca21Chr1_0035283  0.09  0.26  0.42 -0.19 -0.34 -0.07 
Ca21Chr1_0035342  0.29  0.15  0.33 -0.18  0.11  0.14 
Ca21Chr1_0035401  0.39 -0.02 -0.17 -0.56 -0.23 -0.63 
Ca21Chr1_0035460 -0.28 -0.44 -0.43 -0.05 -0.22 -0.39 
Ca21Chr1_0035519 -0.89 -0.03 -0.24 -0.14  0.01 -0.07 
Ca21Chr1_0035578 -0.55 -0.22 -0.69 -0.07  0.24  0.05 
Ca21Chr1_0035637 -0.79 -0.43 -0.91 -0.82 -0.21  0.26  <orf19.6082
Ca21Chr1_0035696 -1.13 -0.76 -2.02 -0.39  0.03 -0.48 
Ca21Chr1_0035755 -2.89 -1.77 -2.58 -0.18  0.34  0.01 
Ca21Chr1_0035814 -2.69 -1.48 -2.24 -0.13 -0.10 -0.21 
Ca21Chr1_0035873 -2.44 -2.66 -2.60 -0.35 -0.13 -0.13 
Ca21Chr1_0035932 -2.19 -2.77 -2.95 -0.43  0.03 -0.20 
Ca21Chr1_0035991 -2.30 -1.85 -2.12  0.63  0.01 -0.15 
Ca21Chr1_0036050 -2.68 -2.44 -1.93 -0.35  0.32  0.09 
Ca21Chr1_0036109 -1.57 -2.25 -1.62 -0.04  0.03 -0.19 
Ca21Chr1_0036168 -2.12 -1.77 -1.74 -0.11  0.22  0.05 
Ca21Chr1_0036227 -1.56 -1.14 -1.22 -0.24  0.37  0.19 
Ca21Chr1_0036286 -1.21 -0.33 -0.90 -0.37  0.21  0.01 
Ca21Chr1_0036345 -1.05 -1.31 -1.24 -0.57  0.03 -0.21 
Ca21Chr1_0036404 -1.08 -0.76 -0.53 -0.34  0.02 -0.33 
Ca21Chr1_0036463 -0.42  0.10  0.21  0.04 -0.27 -0.27 
Ca21Chr1_0036522 -0.93  0.21  0.92 -0.64 -0.12 -0.33 
Ca21Chr1_0036581 -0.64  0.19  0.50  0.07  0.11 -0.17 
Ca21Chr1_0036640 -0.15  0.55 -0.46 -0.09  0.28 -0.35 
Ca21Chr1_0036699  0.21  0.40  0.35 -0.02  0.29 -0.00 
Ca21Chr1_0036758 -0.65 -0.22  0.05 -0.02 -0.35 -0.13 
Ca21Chr1_0036817 -0.41  0.43  0.05  0.20  0.42  0.36 
Ca21Chr1_0036876 -0.86  0.04  0.35 -0.62  0.03 -0.19 
Ca21Chr1_0036935  0.17  0.16 -0.18 -0.57 -0.06 -0.03 
Ca21Chr1_0036994 -0.99 -0.38 -0.15 -0.53 -0.21 -0.25 
Ca21Chr1_0037053 -0.63 -0.11 -0.33 -0.89 -0.18 -0.72 
Ca21Chr1_0037112 -0.40 -0.04  0.17 -0.32  0.04 -0.15 
Ca21Chr1_0037171 -0.60 -0.34  0.05 -0.38  0.03 -0.39 
Ca21Chr1_0037230 -0.17 -0.51 -0.52 -0.03  0.09 -0.11 
Ca21Chr1_0037289 -0.22 -0.22  0.13 -0.26  0.01 -0.14 
Ca21Chr1_0037348 -0.75 -0.26 -0.61 -0.30 -0.73 -0.06 
Ca21Chr1_0037407 -0.36 -0.50 -0.96 -0.26 -0.22  0.30 
Ca21Chr1_0037466 -0.70 -0.78 -0.78 -0.15 -0.71  0.52 
Ca21Chr1_0037525 -1.17 -0.74 -1.01 -0.19  0.01  0.53 
Ca21Chr1_0037584 -0.85 -0.23 -0.55 -0.33 -0.08  0.79 
Ca21Chr1_0037643 -0.41 -0.32 -0.35 -0.10 -0.06  0.49 
Ca21Chr1_0037702 -0.10 -0.48 -0.37 -0.54 -0.01  0.34 
Ca21Chr1_0037761  0.25 -0.14 -0.27 -0.47  0.31  0.27 
Ca21Chr1_0037820 -0.63  0.28  0.16 -0.10  0.16  0.29  orf19.6081>
Ca21Chr1_0037879  0.34  0.05  0.38 -0.21  0.20  0.74 
Ca21Chr1_0037938 -0.05 -0.05  0.27  0.13 -0.26 -0.18 
Ca21Chr1_0037997  0.12  0.08  0.14  0.06  0.59 -0.42 
Ca21Chr1_0038056 -0.16  0.27  0.37  0.02  0.79 -0.28 
Ca21Chr1_0038115  0.89  0.20  0.43  0.13 -0.14 -0.27 
Ca21Chr1_0038174  1.84  0.26  0.50 -0.02  0.20  0.47 
Ca21Chr1_0038233  0.22  0.02  0.31  0.01 -0.30 -0.19 
Ca21Chr1_0038292  0.16 -0.01  0.23  0.09  0.15 -0.17 
Ca21Chr1_0038351 -0.32  0.06  0.54 -0.04  0.66 -0.51 
Ca21Chr1_0038410  0.21  0.22  0.74  0.08  0.23 -0.37 
Ca21Chr1_0038469  0.22  0.10  0.55  0.14 -0.40 -0.16 
Ca21Chr1_0038528  0.14  0.04  0.22  0.08 -0.13 -0.24 
Ca21Chr1_0038587 -0.09  0.12  0.50  0.04  0.13 -0.36 
Ca21Chr1_0038646 -0.41  0.13  0.44 -0.01  0.44 -0.29 
Ca21Chr1_0038705 -0.28 -0.07  0.27 -0.05  0.23 -0.29 
Ca21Chr1_0038764  0.32  0.25  0.25  0.16  0.26 -0.14 
Ca21Chr1_0038823 -0.05  0.26  0.57 -0.60  0.38 -0.39 
Ca21Chr1_0038882  0.13  0.16  0.71  0.60 -0.49  0.20 
Ca21Chr1_0038941  0.64  0.29  0.58 -0.32  0.27 -0.30 
Ca21Chr1_0039000  0.02 -0.07  0.37  0.02  0.16 -0.29 
Ca21Chr1_0039059  0.29  0.18  0.60 -0.21  0.23 -0.58 
Ca21Chr1_0039118 -0.06  0.21  0.33 -0.00 -0.03 -0.50 
Ca21Chr1_0039177  0.00  0.07  0.63 -0.11  0.23 -0.69 
Ca21Chr1_0039236  0.48  0.40  0.46 -0.56  0.42 -0.52 
Ca21Chr1_0039295  0.25  0.48  0.52  0.03  0.32 -0.42 
Ca21Chr1_0039354 -0.38  0.23  0.44 -0.17 -0.13 -0.35 
Ca21Chr1_0039413 -0.03  0.13  0.21 -0.12  0.18 -0.31 
Ca21Chr1_0039472  0.02 -0.05  0.31  0.31  0.11 -0.42  orf19.6081|
Ca21Chr1_0039531 -0.23 -0.12  0.17  0.19  0.08 -0.12 
Ca21Chr1_0039590 -0.09  0.21  0.45  0.01  0.19  0.25 
Ca21Chr1_0039649  0.34  0.42  0.54  0.03  0.24 -0.04 
Ca21Chr1_0039708 -0.25  0.17  0.36  0.06  0.09  0.27 
Ca21Chr1_0039767  0.30  0.23  0.23  0.25 -0.02 -0.23 
Ca21Chr1_0039826  0.37 -0.07  0.33  0.88 -0.20  0.00 
Ca21Chr1_0039885  0.14  0.08  0.46  0.17 -0.04 -0.27 
Ca21Chr1_0039944  0.29  0.15  0.01 -0.08  0.25 -0.13 
Ca21Chr1_0040003 -0.32  0.34  0.33 -0.28  0.15 -0.20 
Ca21Chr1_0040062 -0.12 -0.03  0.21  0.03  0.02  0.01 
Ca21Chr1_0040121 -0.01  0.21  0.43  0.16 -0.20 -0.23 
Ca21Chr1_0040180  0.07 -0.32  0.12 -0.15  0.31  0.25 
Ca21Chr1_0040239 -0.29  0.07  0.04  0.27  0.31  0.11 
Ca21Chr1_0040298 -0.43  0.30  0.13  0.04  0.01 -0.02 
Ca21Chr1_0040357  0.27  1.02  0.36 -0.17  0.45 -0.21 
Ca21Chr1_0040416  0.96  0.36  0.44 -0.17  0.03 -0.44 
Ca21Chr1_0040475  0.14  0.24  0.25 -0.22 -0.25 -0.54  |orf19.6080
Ca21Chr1_0040534  0.34  0.24 -0.13  0.01 -0.06 -0.41 
Ca21Chr1_0040593  0.25  0.04  0.32 -0.02 -0.54 -0.20 
Ca21Chr1_0040652  0.42  0.26  0.66 -0.06  0.42 -0.37 
Ca21Chr1_0040711  0.07 -0.03  0.48 -0.04  0.63 -0.37 
Ca21Chr1_0040770 -0.17 -0.23  0.23 -0.14  0.44  0.08 
Ca21Chr1_0040829  0.27 -0.03  0.04 -0.10  0.27 -0.23 
Ca21Chr1_0040888  0.25  0.02  0.41  0.30 -0.00  0.08 
Ca21Chr1_0040947  0.24  0.20  0.48  0.04  0.11 -0.00 
Ca21Chr1_0041006  0.17 -0.10  0.14 -0.07  0.17 -0.11 
Ca21Chr1_0041065  0.36  0.10  0.33  0.01  0.57 -0.31 
Ca21Chr1_0041124  0.35  0.10  0.32 -0.33  0.09  0.13 
Ca21Chr1_0041183  0.23  0.09  0.48  0.28 -0.05 -0.42 
Ca21Chr1_0041242  1.35  0.26  0.13 -0.76  0.03  0.60 
Ca21Chr1_0041301  0.11  0.20  0.28  0.29  0.12 -0.26 
Ca21Chr1_0041360  0.38  0.36  0.36 -0.15  0.38 -0.55 
Ca21Chr1_0041419  0.27  0.06  0.51 -0.04  0.27 -0.18 
Ca21Chr1_0041478  0.18  0.16  0.56 -0.01  0.33 -0.07 
Ca21Chr1_0041537  0.18  0.18  0.36 -0.32  0.11 -0.30 
Ca21Chr1_0041596  0.02  0.27 -0.01 -0.05  0.04 -0.01 
Ca21Chr1_0041655  0.76  0.38  0.39 -0.09  0.25  0.10 
Ca21Chr1_0041714 -0.12  0.24 -0.45 -0.45  0.11 -0.29  <orf19.6080
Ca21Chr1_0041773 -0.41 -0.13  0.09 -0.18  0.54 -0.19 
Ca21Chr1_0041832  0.18  0.10 -0.07  0.63 -0.21  0.10 
Ca21Chr1_0041891 -0.74 -0.31 -0.82 -0.70 -0.41 -0.03 
Ca21Chr1_0041950 -0.80 -0.60 -0.55 -0.57 -0.09 -0.14 
Ca21Chr1_0042009 -0.30 -0.36  0.46 -0.06 -0.13  0.13 
Ca21Chr1_0042068  0.06 -0.02 -0.10 -0.12 -0.25  0.20  |tT(AGU)6
Ca21Chr1_0042127  0.22  0.28 -0.11 -0.24  0.78  0.10  <tT(AGU)6
Ca21Chr1_0042186 -1.38  0.25 -0.86 -0.40 -0.40  0.29 
Ca21Chr1_0042245 -0.58 -0.70 -1.16 -0.66 -0.03  0.26 
Ca21Chr1_0042304 -1.68 -0.98 -2.12 -0.52 -0.03  0.63 
Ca21Chr1_0042363 -1.89 -2.10 -2.10  0.09  0.01  0.58 
Ca21Chr1_0042422 -0.35 -1.00 -1.01 -0.47  0.29 -0.55 
Ca21Chr1_0042481 -0.79 -0.93 -1.05 -0.36 -0.60  0.06 
Ca21Chr1_0042540 -0.33 -0.11 -0.57 -0.36 -0.03 -0.38 
Ca21Chr1_0042599  0.27  0.10  0.46 -0.03  0.02  0.09  orf19.6079>
Ca21Chr1_0042658 -0.09  0.63  0.21 -0.14  0.74 -0.12 
Ca21Chr1_0042717  0.09  0.11  0.33  0.26 -0.23 -0.30 
Ca21Chr1_0042776  0.52  0.10  0.21  0.19  0.27  0.04 
Ca21Chr1_0042835  0.09  0.36  0.19 -0.06  0.23 -0.01 
Ca21Chr1_0042894  0.24 -0.10  0.06  0.08  0.11  0.13 
Ca21Chr1_0042953 -0.24  0.54  0.32  0.29  0.04  0.19 
Ca21Chr1_0043012 -1.52 -0.03  0.44  0.10 -0.65  0.49 
Ca21Chr1_0043071  0.21 -0.06  0.38 -0.07 -0.09  0.21 
Ca21Chr1_0043130  0.23 -0.07  0.15 -0.05 -0.14 -0.09 
Ca21Chr1_0043189  0.25 -0.28  0.27  0.09 -0.04 -0.34 
Ca21Chr1_0043248  0.05  0.06  0.30  0.45  0.09 -0.18 
Ca21Chr1_0043307  0.12  0.40  0.22  0.00  0.14 -0.02 
Ca21Chr1_0043366  0.50  0.21  0.41  0.09  0.03  0.20 
Ca21Chr1_0043425 -0.26 -0.10  0.03  0.68  0.12 -0.64  |orf19.6078.1
Ca21Chr1_0043484  0.28  0.05  0.41  0.37 -0.08  0.79  orf19.6079|
Ca21Chr1_0043543  0.42  0.29  0.61 -0.28 -0.19 -0.06 
Ca21Chr1_0043602  0.34  0.22  0.32  0.08 -0.43  0.27 
Ca21Chr1_0043661    NA    NA    NA    NA    NA    NA <orf19.6078.1
Ca21Chr1_0043720    NA    NA    NA    NA    NA    NA
Ca21Chr1_0043779    NA    NA    NA    NA    NA    NA orf19.6078>
Ca21Chr1_0043838  0.33  0.12  0.30  0.01  0.21 -0.04 
Ca21Chr1_0043897 -0.13 -0.20  0.16  0.09  0.53  0.04 
Ca21Chr1_0043956 -0.61  0.11  0.07 -0.09  0.17 -0.77 
Ca21Chr1_0044015  0.91  0.08  0.23  0.08 -0.04 -0.18 
Ca21Chr1_0044074  0.37 -0.19  0.25 -0.49  0.30 -0.41 
Ca21Chr1_0044133 -0.10 -0.16  0.35 -0.04  0.06 -0.23 
Ca21Chr1_0044192  0.05  0.24 -0.19 -0.40 -0.52 -0.46 
Ca21Chr1_0044251  0.28 -0.03  0.44 -0.21  0.16  0.47 
Ca21Chr1_0044310  0.47  0.31  0.20 -0.04 -0.19  0.60 
Ca21Chr1_0044369  0.44  0.20  0.40 -0.23  0.26 -0.24 
Ca21Chr1_0044428  0.23  0.26 -0.06  0.05  0.12 -0.33 
Ca21Chr1_0044487  0.29  0.32  0.07  0.20 -0.07 -0.29 
Ca21Chr1_0044546 -0.04  0.21  0.24  0.01 -0.02 -0.13 
Ca21Chr1_0044605 -0.03 -0.11  0.21 -0.02 -0.29 -0.43 
Ca21Chr1_0044664  0.84  0.26  0.37  0.25 -0.30 -0.32 
Ca21Chr1_0044723 -0.25  0.60  0.14  0.21  0.01 -0.57 
Ca21Chr1_0044782 -0.21  0.27 -0.24  0.33  0.17 -0.16 
Ca21Chr1_0044841 -0.28 -0.22 -0.51 -0.03 -0.33 -0.24 
Ca21Chr1_0044900 -0.19 -0.33 -0.23 -0.11  0.07 -0.91 
Ca21Chr1_0044959  0.20  0.18  0.24 -0.26  0.17 -0.84 
Ca21Chr1_0045018 -0.11  0.04  0.14 -0.13  0.30 -0.47 
Ca21Chr1_0045077  0.21  0.25 -0.01 -0.04 -0.00 -0.22 
Ca21Chr1_0045136  0.12 -0.02 -0.08 -0.21  0.00 -0.18 
Ca21Chr1_0045195  0.53 -0.25 -0.37 -0.29 -0.05 -0.01 
Ca21Chr1_0045254  0.00 -0.21 -0.16  0.62 -0.05  0.02 
Ca21Chr1_0045313 -0.33 -0.03 -0.17  0.15 -0.24  0.10 
Ca21Chr1_0045372  0.11  0.03 -0.09  0.50  0.26  0.00 
Ca21Chr1_0045431  0.04 -0.13 -0.09 -0.21  0.10 -0.05 
Ca21Chr1_0045490 -0.32 -0.32 -0.18  0.10 -0.29 -0.30 
Ca21Chr1_0045549 -0.01 -0.11 -0.53 -0.25  0.24 -0.45 
Ca21Chr1_0045608 -0.33 -0.63 -0.80 -0.31 -0.08 -0.62 
Ca21Chr1_0045667 -0.66 -0.33 -0.57 -0.09  0.09 -0.59 
Ca21Chr1_0045726  0.01 -0.44 -1.09  0.08 -0.93 -0.73  orf19.6078|
Ca21Chr1_0045785 -0.30 -0.25 -1.04 -0.47  0.04  0.04 
Ca21Chr1_0045844 -1.14 -0.73 -1.54 -0.30  0.08  0.03 
Ca21Chr1_0045903 -1.83 -0.94 -1.57 -0.28  0.05  0.06 
Ca21Chr1_0045962 -1.82 -1.58 -2.24 -0.38  0.32  0.27 
Ca21Chr1_0046021 -0.21 -1.04 -1.27  0.18  0.03 -0.62 
Ca21Chr1_0046080 -0.36 -0.36 -0.24 -0.24  0.14 -0.19 
Ca21Chr1_0046139 -0.39 -0.29 -0.67 -0.23  0.93 -0.15 
Ca21Chr1_0046198 -0.43 -0.36 -0.58 -0.47 -0.17 -0.31 
Ca21Chr1_0046257 -0.33 -0.05 -0.12 -0.15 -0.08 -0.02 
Ca21Chr1_0046316 -0.03 -0.41 -0.52 -0.29  0.07  0.02 
Ca21Chr1_0046375 -0.19 -0.44 -0.54 -0.24  0.01  0.13 
Ca21Chr1_0046434 -0.27 -0.32 -0.71  0.32  0.04 -0.14 
Ca21Chr1_0046493 -0.18 -0.47 -0.31 -0.22  0.11 -0.07 
Ca21Chr1_0046552  0.32 -0.47 -0.23 -0.39  0.34  0.13 
Ca21Chr1_0046611 -0.17 -0.36 -0.52 -0.19 -0.90  0.07 
Ca21Chr1_0046670 -0.36 -0.36 -0.34 -0.44  0.27 -0.28 
Ca21Chr1_0046729  0.19  0.04 -0.14 -0.14  0.18 -0.28 
Ca21Chr1_0046788  0.13  0.15  0.55  0.71 -0.10  0.11  orf19.6077>
Ca21Chr1_0046847 -0.05  0.32  0.36  0.36 -0.09  0.26 
Ca21Chr1_0046906  0.14  0.27  0.52  0.14 -0.24 -0.36 
Ca21Chr1_0046965  0.36  0.29  0.49  0.43  0.40 -0.05 
Ca21Chr1_0047024  0.19  0.76  0.40 -0.04  0.05 -0.08 
Ca21Chr1_0047083  0.05  0.51  0.43 -0.01  0.17 -0.03 
Ca21Chr1_0047142  0.41  0.22  0.62  0.06 -0.02 -0.08 
Ca21Chr1_0047201  0.29 -0.03  0.63  0.03 -0.25 -0.16 
Ca21Chr1_0047260  0.04  0.29  0.27 -0.19 -0.00 -0.16 
Ca21Chr1_0047319  0.29  0.15  0.23 -0.17 -0.07 -0.17 
Ca21Chr1_0047378  0.18  0.21  0.36 -0.02  0.24 -1.49 
Ca21Chr1_0047437  0.84  0.35  0.08 -0.18  0.10 -0.32 
Ca21Chr1_0047496  0.09  0.02  0.31  0.27 -0.40 -0.40 
Ca21Chr1_0047555  0.53  0.26  0.44 -0.06 -0.27 -0.07 
Ca21Chr1_0047614  0.52  0.20  0.33 -0.09  0.29  0.11 
Ca21Chr1_0047673  0.16  0.29  0.39 -0.15  0.14 -0.08 
Ca21Chr1_0047732  0.03  0.47  0.29 -0.02  0.02 -0.04 
Ca21Chr1_0047791  0.15  0.33  0.57 -0.28 -0.06  0.11 
Ca21Chr1_0047850  0.15  0.25  0.25 -0.03  0.05 -0.09 
Ca21Chr1_0047909  0.11  0.43  0.31 -0.44 -0.03  0.12 
Ca21Chr1_0047968  0.17  0.38  0.36  0.18 -0.10  0.08 
Ca21Chr1_0048027  0.23 -0.49  0.44 -0.07  0.07  0.16 
Ca21Chr1_0048086  1.06  0.22  0.41  0.05 -0.28 -0.22 
Ca21Chr1_0048145  0.19  0.42  0.31 -0.16  0.28  0.12 
Ca21Chr1_0048204  0.36  0.14  0.58  0.06 -0.01  0.05 
Ca21Chr1_0048263  0.63 -0.01  0.50  0.27  0.88  0.23 
Ca21Chr1_0048322 -0.16 -0.08  0.05  0.61 -0.41  0.15  orf19.6077|
Ca21Chr1_0048381  0.14 -0.26  0.20 -0.20  0.29  0.00 
Ca21Chr1_0048440  0.00  0.02 -0.06  0.42  0.02 -0.24 
Ca21Chr1_0048499  0.20  0.43  0.02  0.07 -0.37 -0.53  orf19.6076>
Ca21Chr1_0048558  0.48  0.14  0.15  0.00  0.15 -0.72 
Ca21Chr1_0048617  0.25  0.37  0.35  0.12 -0.29 -0.35 
Ca21Chr1_0048676  0.26  0.02  0.26 -0.17 -0.03 -0.34 
Ca21Chr1_0048735 -0.01  0.27  0.46  0.47 -0.01  0.21 
Ca21Chr1_0048794 -0.12  0.03  0.31  0.14 -0.04 -0.16 
Ca21Chr1_0048853  0.13  1.16  0.20  0.09  0.27 -0.15 
Ca21Chr1_0048912  0.23  0.46  0.39  0.13  0.24  0.06 
Ca21Chr1_0048971  0.21 -0.23  0.10 -0.06  0.18 -0.05 
Ca21Chr1_0049030  0.16 -0.01  0.07  0.34  0.18  0.08 
Ca21Chr1_0049089 -0.20  0.17  0.26  0.54  0.27 -0.24 
Ca21Chr1_0049148 -0.34  0.13  0.29  0.28  0.21 -0.16 
Ca21Chr1_0049207  0.13  0.18  0.43  0.21  0.16 -0.18 
Ca21Chr1_0049266  0.00  0.03 -0.09  0.24  0.14 -0.42  |orf19.6075 orf19.6076|
Ca21Chr1_0049325  0.22  0.08  0.56 -0.08 -0.70 -0.20 
Ca21Chr1_0049384  0.60  0.26  0.33 -0.06  0.08 -0.15 
Ca21Chr1_0049443  0.26  0.21  0.29 -0.48 -0.15 -0.19 
Ca21Chr1_0049502  0.47  0.10  0.36 -0.50  0.14 -0.08 
Ca21Chr1_0049561  0.29  0.35  0.42 -0.45 -0.11 -0.08 
Ca21Chr1_0049620  0.30  0.27  0.40 -0.17 -0.07  0.09 
Ca21Chr1_0049679 -0.13 -0.03  0.27 -0.05 -0.35 -0.05 
Ca21Chr1_0049738  0.31  0.15  0.20 -0.05  0.11 -0.01 
Ca21Chr1_0049797  0.29  0.27  0.22 -0.01  0.05  0.08  <orf19.6075
Ca21Chr1_0049856  0.02 -0.56  0.10 -0.07  0.25 -0.10 
Ca21Chr1_0049915  0.05 -0.26  0.17  0.42 -0.27 -0.43 
Ca21Chr1_0049974  0.09  0.00  0.19 -0.11 -0.16 -0.13  orf19.6074>
Ca21Chr1_0050033  0.11  0.03 -0.06 -0.29  0.05 -0.10 
Ca21Chr1_0050092  0.11  0.28  0.12  0.21  0.14 -0.11 
Ca21Chr1_0050151  0.23  0.16  0.02  0.28  0.08  0.06 
Ca21Chr1_0050210  0.72  0.16  0.24  0.10  0.16 -0.34 
Ca21Chr1_0050269 -0.00  0.22  0.06  0.20 -0.06 -0.60 
Ca21Chr1_0050328  0.21 -0.14  0.27  0.29 -0.02 -0.53 
Ca21Chr1_0050387  0.14  0.79  0.05  0.17  0.00  0.03 
Ca21Chr1_0050446  0.17  0.22  0.22  0.15  0.26  0.18 
Ca21Chr1_0050505 -0.17  0.10  0.15  0.29 -0.40 -0.09 
Ca21Chr1_0050564  0.11  0.19  0.18  0.34  0.01 -0.10 
Ca21Chr1_0050623 -0.05  0.00 -0.08  0.03  0.29 -0.44 
Ca21Chr1_0050682 -0.14  0.21 -0.19  0.35  0.44 -0.33 
Ca21Chr1_0050741  0.22  0.20 -0.12  0.77  0.11  0.08  orf19.6074|
Ca21Chr1_0050800 -0.08  0.01 -0.50  0.03 -0.25 -0.38  |orf19.6073
Ca21Chr1_0050859 -0.39  0.14 -0.17  0.49 -0.23 -0.01 
Ca21Chr1_0050918 -0.99 -0.22 -0.61  0.13  0.14 -0.24 
Ca21Chr1_0050977 -0.56 -0.40 -0.80 -0.15 -0.14 -0.31 
Ca21Chr1_0051036 -1.09 -0.97 -0.82 -0.15 -0.24 -0.02 
Ca21Chr1_0051095 -1.40 -0.90 -0.69 -0.02  0.30 -0.00 
Ca21Chr1_0051154 -0.18 -0.95 -0.39 -0.16  0.22 -0.38 
Ca21Chr1_0051213 -0.33 -0.23 -0.28  0.27  0.04 -0.11 
Ca21Chr1_0051272 -0.30 -0.50 -0.28  0.27  0.25 -0.26 
Ca21Chr1_0051331 -0.89 -0.17 -0.12  0.25  0.40  0.52 
Ca21Chr1_0051390  0.27 -0.10  0.17  0.28 -0.02 -0.07 
Ca21Chr1_0051449 -0.02  0.12 -0.15 -0.14  0.16 -0.48 
Ca21Chr1_0051508  0.17  0.01 -0.03  0.11  0.01 -0.30 
Ca21Chr1_0051567  0.35 -0.00 -0.36  0.21 -0.45  0.26 
Ca21Chr1_0051626  0.91  0.25 -0.15  0.51 -0.23 -0.80  <orf19.6073
Ca21Chr1_0051685  0.02 -0.25 -0.11  0.06  0.02  0.77 
Ca21Chr1_0051744  0.01  0.01 -0.54 -0.44  0.19 -0.34 
Ca21Chr1_0051803 -0.35  0.06 -0.46  0.12 -0.04  0.24 
Ca21Chr1_0051862 -0.29 -0.07 -0.78 -0.66 -0.14 -0.11 
Ca21Chr1_0051921 -0.26 -0.33 -0.72 -0.05 -0.35 -0.12 
Ca21Chr1_0051980 -0.25 -0.24 -0.25 -0.06 -0.19 -0.11 
Ca21Chr1_0052039 -0.43 -0.15 -0.57 -0.33 -0.15 -0.08 
Ca21Chr1_0052098 -0.40 -0.31 -0.73  0.22 -0.06 -0.64 
Ca21Chr1_0052157 -0.57 -0.62 -1.07  0.08 -0.42 -0.42 
Ca21Chr1_0052216 -0.30  0.04 -0.93  0.07  0.25 -0.39 
Ca21Chr1_0052275 -0.21 -0.67 -0.25  0.16  0.15 -0.33 
Ca21Chr1_0052334 -0.79 -0.98 -1.07  0.08  0.08 -0.32 
Ca21Chr1_0052393 -0.84 -0.66 -0.67  0.40 -0.22 -0.38 
Ca21Chr1_0052452 -0.87 -0.84 -0.98 -0.08  0.22 -0.19 
Ca21Chr1_0052511    NA    NA    NA    NA    NA    NA
Ca21Chr1_0052570 -0.66 -0.86 -0.62 -0.03 -0.18 -0.08 
Ca21Chr1_0052629 -0.97 -0.82 -1.37 -0.04 -0.27  0.04 
Ca21Chr1_0052688 -1.12 -0.75 -1.07  0.09 -0.17 -0.20 
Ca21Chr1_0052747 -0.85 -1.06 -0.97  0.32  0.17 -0.30 
Ca21Chr1_0052806 -0.54 -0.70 -0.96 -0.30 -0.09 -0.11 
Ca21Chr1_0052865  0.58 -0.73 -0.66 -0.06  0.05 -0.33 
Ca21Chr1_0052924  0.19 -0.90 -0.07 -0.05  0.27 -0.28 
Ca21Chr1_0052983 -0.27 -0.27 -0.21  0.16  0.09 -0.27 
Ca21Chr1_0053042  0.14 -0.40 -0.01 -0.16  0.10 -0.21  orf19.6072>
Ca21Chr1_0053101 -0.23 -0.20 -0.11 -0.11 -0.01 -0.36 
Ca21Chr1_0053160  0.09 -0.21  0.10 -0.33  0.20 -0.17 
Ca21Chr1_0053219  0.01  0.07  0.28  0.19 -0.24 -0.08 
Ca21Chr1_0053278  0.36  0.02  0.30 -0.00  0.09 -0.01 
Ca21Chr1_0053337  0.03 -0.04 -0.05 -0.15 -0.11 -0.25 
Ca21Chr1_0053396  0.09 -0.06  0.03 -0.39 -0.13 -0.16 
Ca21Chr1_0053455  0.13  0.17  0.06  0.52 -0.53 -0.31 
Ca21Chr1_0053514  0.21  0.13  0.16 -0.02 -0.03 -0.27 
Ca21Chr1_0053573 -0.29  0.26  0.28 -0.22 -0.42  0.25 
Ca21Chr1_0053632 -0.12 -0.04  0.28 -0.11  0.05 -0.32 
Ca21Chr1_0053691  0.19 -0.04  0.08 -0.25  0.20 -0.56 
Ca21Chr1_0053750  0.09  0.28  0.22 -0.30  0.00 -0.10 
Ca21Chr1_0053809  1.09  0.16  0.24 -0.47  0.25 -0.27 
Ca21Chr1_0053868  0.51  0.19  0.51 -0.54  0.71  0.04 
Ca21Chr1_0053927  0.22  0.05  0.38 -0.34 -0.20  0.03 
Ca21Chr1_0053986 -0.25  0.30  0.15 -0.28  0.03  0.35 
Ca21Chr1_0054045  0.17  0.14  0.14  0.18 -0.35 -0.01 
Ca21Chr1_0054104  0.04 -0.21  0.57 -0.27 -0.15 -0.06 
Ca21Chr1_0054163 -0.05 -0.29  0.16  0.04 -0.34 -0.20 
Ca21Chr1_0054222  0.09 -0.06 -0.10 -0.02  0.17 -0.09 
Ca21Chr1_0054281  0.06 -0.32 -0.07  0.25 -0.19  0.37 
Ca21Chr1_0054340 -0.05 -0.31  0.05  0.02 -0.01 -0.27 
Ca21Chr1_0054399  0.10 -0.32 -0.08 -0.12  0.19  0.12  orf19.6072|
Ca21Chr1_0054458 -0.58 -0.08  0.06  0.15 -0.02 -0.30 
Ca21Chr1_0054517  0.26 -0.27  0.22 -0.49  0.33 -0.20 
Ca21Chr1_0054576 -0.07 -0.11 -0.04  0.30 -0.01 -0.32 
Ca21Chr1_0054635 -0.01  0.00 -0.18  0.13 -0.01 -0.33 
Ca21Chr1_0054694 -0.07 -0.06  0.06  0.06  0.14 -0.18 
Ca21Chr1_0054753  0.19 -0.21 -0.13  0.15  0.19  0.43 
Ca21Chr1_0054812 -0.07  0.16 -0.27  0.28  0.08  0.07  |orf19.6071
Ca21Chr1_0054871 -0.18  0.39 -0.15  0.23 -0.18  0.25 
Ca21Chr1_0054930  0.17 -0.05 -0.13 -0.05 -0.11  0.10 
Ca21Chr1_0054989 -0.02  0.19  0.03  0.19 -0.12  0.87 
Ca21Chr1_0055048  0.43 -0.11 -0.08  0.35  0.04  0.44 
Ca21Chr1_0055107  0.89  0.01  0.30  0.11 -0.03  0.05 
Ca21Chr1_0055166  0.25  0.15  0.00  0.13 -0.29  0.05 
Ca21Chr1_0055225 -0.00  0.02  0.08 -0.12 -0.17 -0.19 
Ca21Chr1_0055284  0.49  0.19  0.31  0.07  0.08 -0.06 
Ca21Chr1_0055343  0.14  0.18  0.20  0.27  0.40 -0.11 
Ca21Chr1_0055402  0.25  0.14  0.64  0.15  0.33  0.55 
Ca21Chr1_0055461  0.26  0.09  0.24  0.04  0.22 -0.02 
Ca21Chr1_0055520  0.59  0.07  0.24 -0.17  0.08 -0.00 
Ca21Chr1_0055579  0.11  0.14  0.18  0.04 -0.09  0.16 
Ca21Chr1_0055638  1.06  0.25  0.27 -0.26 -0.69  0.45 
Ca21Chr1_0055697  0.55  0.22  0.26  0.21 -0.17  0.10 
Ca21Chr1_0055756  0.46  0.17  0.40  0.16  0.13  0.25 
Ca21Chr1_0055815  0.05  0.06  0.16 -0.36 -0.40  0.15 
Ca21Chr1_0055874  0.33  0.34  0.19 -0.17 -0.07 -0.13 
Ca21Chr1_0055933  0.24  0.18  0.42 -0.02  0.08 -0.05 
Ca21Chr1_0055992  0.29  0.05  0.24 -0.09  0.31  0.45 
Ca21Chr1_0056051  0.21  0.15  0.17  0.13  0.05 -0.00 
Ca21Chr1_0056110  0.23  0.21  0.47  0.04 -0.03 -0.27 
Ca21Chr1_0056169  0.45  0.29  0.26 -0.24  0.28 -0.05 
Ca21Chr1_0056228  0.58 -0.28  0.21  0.17 -0.13 -0.17 
Ca21Chr1_0056287  0.32 -0.49  0.48  0.29  0.05  0.10 
Ca21Chr1_0056346  0.64  0.31  0.34  0.26  0.53 -0.32 
Ca21Chr1_0056405  0.26  0.20  0.17  0.37  0.07  0.13 
Ca21Chr1_0056464  0.28  0.92  1.83  0.13  0.19  0.18 
Ca21Chr1_0056523  0.19  0.24  0.36 -0.15  0.02 -0.34 
Ca21Chr1_0056582  0.41  0.08  0.19  0.06  0.10 -0.01 
Ca21Chr1_0056641  0.37  0.28  0.42  0.62  0.30 -0.34 
Ca21Chr1_0056700  0.20  0.11  0.53 -0.15 -0.33 -0.95 
Ca21Chr1_0056759  0.41  0.09  0.28 -0.14  0.18 -0.73 
Ca21Chr1_0056818  0.32  0.06  0.05 -0.06 -0.31 -0.34 
Ca21Chr1_0056877  0.01 -0.07  0.22  0.25 -0.05 -0.15 
Ca21Chr1_0056936  0.32 -0.13  0.03 -0.15  0.26 -0.38 
Ca21Chr1_0056995  1.48  0.12  0.28  0.07  0.31 -0.03 
Ca21Chr1_0057054  0.72  0.09  0.08  0.11 -0.01 -0.29  <orf19.6071
Ca21Chr1_0057113  0.08 -0.07  0.04 -0.13  0.20 -0.34 
Ca21Chr1_0057172  0.40  0.18  0.16 -0.05 -0.12 -0.40 
Ca21Chr1_0057231 -0.01  0.09  0.17 -0.10 -0.27 -0.46 
Ca21Chr1_0057290  0.11  0.04  0.12 -0.12 -0.18 -0.27 
Ca21Chr1_0057349  0.15  0.13  0.24 -0.07 -0.15  0.06 
Ca21Chr1_0057408  0.17  0.04 -0.06 -0.02 -0.03  0.02 
Ca21Chr1_0057467  0.29  0.14  0.34 -0.15 -0.23  0.15 
Ca21Chr1_0057526 -0.10  0.11  0.22 -0.15 -0.15  0.24 
Ca21Chr1_0057585 -0.11  0.09 -0.30  0.12  0.04 -0.09 
Ca21Chr1_0057644  0.03 -0.11  0.04 -0.07 -0.12 -0.23 
Ca21Chr1_0057703  0.40  0.16 -0.00  0.27 -0.32 -0.28 
Ca21Chr1_0057762 -0.53  0.02 -0.51 -0.18 -0.14 -0.76 
Ca21Chr1_0057821 -0.44  0.07  0.06  0.04 -0.14 -0.35 
Ca21Chr1_0057880  0.17  0.14  0.08 -0.01  0.38 -0.03 
Ca21Chr1_0057939  0.03  0.21  0.29 -0.43 -0.06  0.02 
Ca21Chr1_0057998  0.12  0.23  0.10  0.07 -0.35  0.27 
Ca21Chr1_0058057 -0.03 -0.12 -0.19  0.28 -0.31  0.32 
Ca21Chr1_0058116  0.35  0.01  0.17  0.01  0.44 -0.31 
Ca21Chr1_0058175  0.21 -0.05  0.02  0.07 -0.16  0.03 
Ca21Chr1_0058234  0.22  0.19  0.13 -0.18 -0.12  0.33 
Ca21Chr1_0058293  0.18  0.14  0.21 -0.02 -0.20  0.18 
Ca21Chr1_0058352  0.11  0.09 -0.04 -0.10 -0.23  0.37 
Ca21Chr1_0058411 -0.09  0.07  0.24  0.20  0.02  0.08 
Ca21Chr1_0058470  0.28  0.10  0.29  0.26 -0.21  0.11 
Ca21Chr1_0058529 -0.09  0.17  0.08  0.30  0.35 -0.03 
Ca21Chr1_0058588  0.45  0.18 -0.16  0.03  0.25 -0.11 
Ca21Chr1_0058647  0.29 -0.05 -0.24  0.34  0.03 -0.02 
Ca21Chr1_0058706 -0.09 -0.58 -0.03 -0.06  0.32 -0.13 
Ca21Chr1_0058765 -0.19 -0.07 -0.43 -0.08  0.13  0.18 
Ca21Chr1_0058824  0.20 -0.42 -0.48  0.06 -1.14  0.17 
Ca21Chr1_0058883  0.62 -0.01 -0.16  0.82 -0.10  1.89 
Ca21Chr1_0058942  0.22 -0.03 -0.42  0.02 -0.67 -0.41 
Ca21Chr1_0059001 -0.41  0.06  0.21 -0.01  0.99 -0.23 
Ca21Chr1_0059060  0.21  0.08 -0.48  0.16  0.32 -0.40 
Ca21Chr1_0059119  1.96  0.48 -0.34  0.24  0.16 -0.17 
Ca21Chr1_0059178  1.19  0.01 -0.16 -0.31  0.21  0.43 
Ca21Chr1_0059237  0.15  0.24  0.05  0.08  0.62 -0.06 
Ca21Chr1_0059296 -0.21 -0.14  0.19  0.40  0.27  0.22  orf19.6070>
Ca21Chr1_0059355  0.43  0.13  0.49  0.33  0.04  0.05 
Ca21Chr1_0059414  0.22  0.29  0.31  0.09  0.15 -0.23 
Ca21Chr1_0059473 -0.06  0.15  0.53  0.00  0.18 -0.23 
Ca21Chr1_0059532  0.13  0.01  0.67  0.53  0.21 -0.01 
Ca21Chr1_0059591  0.13  0.40  0.88  0.07 -0.34 -0.10 
Ca21Chr1_0059650  0.14 -0.08  0.30  0.18  0.16 -0.09 
Ca21Chr1_0059709  0.37 -0.16  0.16 -0.25  0.34 -0.37 
Ca21Chr1_0059768 -0.03  0.29  0.05  0.51  0.19 -0.35 
Ca21Chr1_0059827  0.56  0.40  0.76 -0.20  0.42 -0.10 
Ca21Chr1_0059886  0.05  0.02  0.35 -0.01  0.05  0.15 
Ca21Chr1_0059945 -0.00  0.01  0.32 -0.15  0.09 -0.49 
Ca21Chr1_0060004  0.46  0.51  0.57 -0.24  0.44 -0.23 
Ca21Chr1_0060063  0.63 -0.04  0.43  0.16  0.15 -0.31 
Ca21Chr1_0060122  0.37  0.18  0.37  0.33  0.22  0.06 
Ca21Chr1_0060181  0.52  0.34  0.63  0.38  0.10 -0.31 
Ca21Chr1_0060240  0.27  1.08  0.58 -0.06  0.07 -0.49 
Ca21Chr1_0060299  0.24  0.29  0.90 -0.12  0.18 -0.64 
Ca21Chr1_0060358  0.15  0.11  0.21 -0.20 -0.06 -0.37 
Ca21Chr1_0060417  0.55 -0.03  0.49 -0.61  0.02 -0.05 
Ca21Chr1_0060476  0.17 -0.02  0.33 -0.02  0.78 -0.00 
Ca21Chr1_0060535  0.30  0.13  0.22  0.08 -0.00 -0.16 
Ca21Chr1_0060594  0.17 -0.03  0.33 -0.33  0.08 -0.12 
Ca21Chr1_0060653  0.22  0.14  0.41  0.01  0.18 -0.23 
Ca21Chr1_0060712  0.54  0.14  0.80  0.16  0.36 -0.06 
Ca21Chr1_0060771  0.22  0.13  1.76  0.06  0.01 -0.21 
Ca21Chr1_0060830  0.23  0.16  0.49 -0.28  0.20 -0.20 
Ca21Chr1_0060889  0.38  0.36  0.52 -0.11 -0.12 -0.44 
Ca21Chr1_0060948  0.32  0.18  0.39  0.12 -0.14 -0.57 
Ca21Chr1_0061007  0.48 -0.02  0.72 -0.20  0.22 -0.35 
Ca21Chr1_0061066  0.00  0.36  0.49  0.27  0.28  0.06 
Ca21Chr1_0061125 -0.12  0.39  0.75  0.11  0.25 -0.12 
Ca21Chr1_0061184  0.24 -0.04  0.36 -0.02  0.11 -0.35 
Ca21Chr1_0061243 -0.05  0.37  0.78  0.24 -0.03 -0.15 
Ca21Chr1_0061302  0.18  0.63  1.04  0.19  0.16  0.03 
Ca21Chr1_0061361  0.04  0.77  0.62  0.31  0.40 -0.17 
Ca21Chr1_0061420 -0.25  0.41  0.20  0.03  0.11 -0.53 
Ca21Chr1_0061479  0.21  0.34  0.87  0.29  0.06 -0.07 
Ca21Chr1_0061538  0.06 -0.10  0.58 -0.37  0.31 -0.10 
Ca21Chr1_0061597  0.27 -0.26  0.60 -0.05  0.28  0.03 
Ca21Chr1_0061656  0.61  0.37  0.06 -0.15  0.00 -0.14 
Ca21Chr1_0061715 -0.23 -0.20  0.06 -0.10 -0.34 -0.40 
Ca21Chr1_0061774  0.36  0.36  0.55 -0.01  0.18 -0.45 
Ca21Chr1_0061833  0.18  0.18  0.12 -0.01  0.28 -0.21 
Ca21Chr1_0061892  0.11  0.10  0.96  0.10  0.11 -0.03 
Ca21Chr1_0061951 -0.07 -0.04  0.48  0.13 -0.54 -0.22 
Ca21Chr1_0062010  0.64  0.20  0.66  0.10  0.11 -0.28 
Ca21Chr1_0062069  0.43  0.23  0.55 -0.20  0.81 -0.48 
Ca21Chr1_0062128  0.08 -0.01  0.66  0.11  0.23 -0.21 
Ca21Chr1_0062187  0.16  0.18  0.51  0.31 -0.02 -0.00 
Ca21Chr1_0062246  0.16  0.35  0.30 -0.02 -0.86  0.12 
Ca21Chr1_0062305  0.62 -0.15  0.44 -0.27 -0.05  0.01 
Ca21Chr1_0062364  0.02  0.35  0.32 -0.06  0.10 -0.18 
Ca21Chr1_0062423  0.31 -0.10  0.51  0.15  0.28 -0.16 
Ca21Chr1_0062482  0.12  0.30  0.27  0.01  0.23 -0.06 
Ca21Chr1_0062541 -0.23  0.03  0.08  0.01 -0.37 -0.53  orf19.6070|
Ca21Chr1_0062600 -0.04 -0.29  0.37  0.08 -0.06 -0.07 
Ca21Chr1_0062659  0.50  0.25  0.45  0.10  0.46 -0.34 
Ca21Chr1_0062718 -0.10  0.20  0.15  0.06  0.15 -0.10 
Ca21Chr1_0062777  0.23 -0.01  0.13 -0.38 -0.00 -0.11 
Ca21Chr1_0062836 -0.26  0.16 -0.23 -0.50 -0.05 -0.19 
Ca21Chr1_0062895 -0.15 -0.06 -0.18  0.72 -0.31  0.01 
Ca21Chr1_0062954 -0.12 -0.23  0.26 -0.10  0.09  0.51 
Ca21Chr1_0063013  0.08 -0.13 -0.35 -0.37 -0.48 -0.03 
Ca21Chr1_0063072  0.53 -0.06  0.16  0.14  0.67 -0.03 
Ca21Chr1_0063131  0.28 -0.14  0.11  0.40 -0.15 -0.49 
Ca21Chr1_0063190 -0.31  0.12  0.15 -0.01  0.19 -0.17  orf19.6068>
Ca21Chr1_0063249  0.14  0.12  0.26  0.25  0.06 -0.38 
Ca21Chr1_0063308  0.30  0.08  0.22  0.00 -0.15  0.03 
Ca21Chr1_0063367 -0.03  0.10  0.32  0.16 -0.23  0.06 
Ca21Chr1_0063426  0.03  0.08  0.56 -0.05 -0.10  0.18 
Ca21Chr1_0063485  0.37 -0.22  0.02  0.07  0.05  0.30 
Ca21Chr1_0063544  0.98 -0.01 -0.06  0.20  0.18 -0.19 
Ca21Chr1_0063603 -0.21  0.16  0.38  0.12  0.19 -0.03 
Ca21Chr1_0063662  0.03  0.32  0.22  0.23 -0.23 -0.08 
Ca21Chr1_0063721  0.10  0.28  0.51  0.32  0.09 -0.09 
Ca21Chr1_0063780 -0.02  0.13 -0.03  0.09  0.12 -0.05 
Ca21Chr1_0063839  0.18  0.15 -0.10 -0.09  0.25 -0.32 
Ca21Chr1_0063898  0.26 -0.01  0.21  0.18  0.30 -0.11 
Ca21Chr1_0063957  0.36 -0.10  0.10  0.03 -0.48 -0.02 
Ca21Chr1_0064016 -0.03 -0.15  0.02 -0.08 -0.04 -0.27 
Ca21Chr1_0064075  0.19 -0.04  0.07 -0.13  0.01  0.05 
Ca21Chr1_0064134  0.08 -0.07  0.27 -0.25 -0.17  0.38 
Ca21Chr1_0064193  0.12  0.25  0.12  0.05  0.01  0.36 
Ca21Chr1_0064252  0.03  0.74  0.44  0.24  0.13 -0.21 
Ca21Chr1_0064311 -0.61  0.46  0.14 -0.56 -0.24  0.33  orf19.6068|
Ca21Chr1_0064370 -0.19  0.00  0.80 -0.02  0.01 -0.94 
Ca21Chr1_0064429  0.64  0.09  0.06  0.14  0.85 -0.06 
Ca21Chr1_0064488 -0.26  0.31  0.50 -0.19  0.07 -0.33 
Ca21Chr1_0064547  0.55 -0.31 -0.72 -0.05  0.46 -0.40 
Ca21Chr1_0064606 -0.07 -0.21 -0.77 -0.11 -0.25 -0.08 
Ca21Chr1_0064665  0.04 -0.11 -1.11  0.38  0.17 -0.27  |orf19.6066
Ca21Chr1_0064724 -0.72 -0.80 -0.26  0.33  0.19  0.10 
Ca21Chr1_0064783 -0.43 -0.65 -0.48 -0.34  0.44 -0.07 
Ca21Chr1_0064842 -0.30 -0.65 -0.85 -0.07  0.26 -0.01 
Ca21Chr1_0064901 -0.01 -0.95 -0.97  0.01  0.11  0.13 
Ca21Chr1_0064960 -0.11 -0.24 -0.51  0.26  0.27 -0.01 
Ca21Chr1_0065019  0.67  0.14 -0.54 -0.25 -0.04 -0.45 
Ca21Chr1_0065078 -0.20  0.03 -0.05  0.04  0.47 -0.38 
Ca21Chr1_0065137 -0.10 -0.10  0.07  0.10  0.38 -0.24 
Ca21Chr1_0065196 -0.02  0.19  0.22  0.16 -0.22 -0.21 
Ca21Chr1_0065255  0.34  0.22  0.27  0.33  0.25 -0.19 
Ca21Chr1_0065314 -0.02  0.05 -0.02 -0.04 -0.36 -0.26 
Ca21Chr1_0065373  0.31  0.11 -0.13  0.16  0.39 -0.25 
Ca21Chr1_0065432  0.43  0.16  0.24  0.03  0.01 -0.01 
Ca21Chr1_0065491  0.46 -0.09  0.00  0.35  0.29 -0.19 
Ca21Chr1_0065550  0.54 -0.03  0.31  0.02  0.16  0.12 
Ca21Chr1_0065609  0.48  0.09  0.48  0.11  0.21  0.20 
Ca21Chr1_0065668  0.69  0.17  0.35  0.42  0.04  0.20 
Ca21Chr1_0065727  0.42  0.12  0.38  0.22  0.12  0.10 
Ca21Chr1_0065786  0.14 -0.00  0.01  0.24  0.13  0.08 
Ca21Chr1_0065845  0.56 -0.09 -0.03  0.14  0.06 -0.25 
Ca21Chr1_0065904  0.41  0.00  0.53  0.49  0.08 -0.53 
Ca21Chr1_0065963  0.48  0.01  0.17  0.14 -0.01 -0.32 
Ca21Chr1_0066022  0.35  0.10  0.20  0.25 -0.05 -0.20 
Ca21Chr1_0066081  0.32  0.14  0.32  0.18 -0.09 -0.01 
Ca21Chr1_0066140  0.15  0.39  0.25 -0.03  0.08  0.15 
Ca21Chr1_0066199  0.52  0.13 -0.05  0.42 -0.07  0.08 
Ca21Chr1_0066258  0.73 -0.01 -0.02  0.27  0.55 -0.12 
Ca21Chr1_0066317 -0.03 -0.15  0.07  0.20 -0.14  0.54  <orf19.6066
Ca21Chr1_0066376  0.37  0.24  0.48  0.60 -0.46  0.23 
Ca21Chr1_0066435 -0.49  0.40  0.13  0.18 -0.20 -0.57 
Ca21Chr1_0066494 -0.22  0.03  0.24 -0.01 -0.11 -0.42 
Ca21Chr1_0066553  0.11 -0.02  0.13 -0.11  0.02  0.01 
Ca21Chr1_0066612  0.15  0.04 -0.10  0.13 -0.03  0.38 
Ca21Chr1_0066671 -0.28  0.38 -0.26  0.22  0.07 -0.24 
Ca21Chr1_0066730 -0.14 -0.05 -0.24  0.02 -0.09 -0.27 
Ca21Chr1_0066789  0.14  0.05 -0.38 -0.17 -0.18 -0.27 
Ca21Chr1_0066848 -0.16  0.58 -0.25  0.00 -0.29 -0.24 
Ca21Chr1_0066907 -0.10 -0.10  0.11 -0.45 -0.10 -0.37 
Ca21Chr1_0066966 -0.02 -0.44 -0.54 -0.09 -0.63  0.01 
Ca21Chr1_0067025 -0.11 -0.49 -0.26  0.06  0.50  0.09 
Ca21Chr1_0067084 -0.24 -0.54 -0.31  0.67  0.02 -0.07 
Ca21Chr1_0067143 -0.10 -0.08 -0.37  0.15 -0.36 -0.09 
Ca21Chr1_0067202 -0.07 -0.29 -0.35  0.21  0.06 -0.08 
Ca21Chr1_0067261  0.00 -0.40 -0.22  0.08 -0.15 -0.03 
Ca21Chr1_0067320  0.01 -0.08 -0.01 -0.12 -0.10 -0.27 
Ca21Chr1_0067379  0.07 -0.15 -0.03 -0.35 -0.14  0.20 
Ca21Chr1_0067438 -0.27 -0.13  0.21  0.04 -0.00 -0.14 
Ca21Chr1_0067497 -0.89  0.25  0.14  0.11 -0.03 -0.21 
Ca21Chr1_0067556 -0.45  0.50 -0.32 -0.05 -0.23 -0.38 
Ca21Chr1_0067615 -0.62  0.06 -0.24 -0.17 -0.07 -0.01 
Ca21Chr1_0067674 -0.61 -0.77 -0.54 -0.29  0.17 -0.01 
Ca21Chr1_0067733 -0.81 -1.45 -0.77  0.17 -0.20 -0.11 
Ca21Chr1_0067792 -0.62 -0.63 -0.42 -0.06  0.04 -0.43  orf19.6065>
Ca21Chr1_0067851  0.57 -0.18 -0.23 -0.30  0.21 -0.18 
Ca21Chr1_0067910  0.29  0.18 -0.20 -0.15 -0.21 -0.17 
Ca21Chr1_0067969  0.12 -0.14  0.44  0.13 -0.15 -0.14 
Ca21Chr1_0068028  0.60  0.08  0.27  0.63  0.03 -0.07 
Ca21Chr1_0068087  0.28  0.06  0.01  0.11 -0.18  0.38 
Ca21Chr1_0068146 -0.25 -0.23  0.33  0.10 -0.04 -0.33 
Ca21Chr1_0068205  0.00 -0.07  0.33 -0.17 -0.07 -0.69 
Ca21Chr1_0068264  0.06  0.13  0.06 -0.35 -0.27 -0.16 
Ca21Chr1_0068323  0.12  0.25  0.35  0.21 -0.19  0.21 
Ca21Chr1_0068382  0.10  0.14  0.04 -0.20  0.00 -0.21 
Ca21Chr1_0068441 -0.02 -0.09  0.32  0.17 -0.13 -0.17 
Ca21Chr1_0068500 -0.04 -0.04 -0.83 -0.06 -0.76 -0.69 
Ca21Chr1_0068559 -0.21  0.12 -0.05  0.33 -0.21 -0.10 
Ca21Chr1_0068618  1.03  0.35  0.19  0.20 -0.76  0.09 
Ca21Chr1_0068677  0.20  0.36  0.45  0.18  0.08 -0.13 
Ca21Chr1_0068736  0.08  0.52  0.17 -0.16 -0.03 -0.01 
Ca21Chr1_0068795 -0.21  0.37  0.20 -0.00  0.01  0.20  orf19.6065|
Ca21Chr1_0068854 -0.00  0.13  0.08  0.12 -0.04 -0.10 
Ca21Chr1_0068913  0.04 -0.02 -0.19  0.07 -0.23 -0.07 
Ca21Chr1_0068972 -0.06 -0.31 -0.53  0.13  0.22 -0.16 
Ca21Chr1_0069031 -0.40 -0.32  0.04  0.80 -0.22 -0.22 
Ca21Chr1_0069090    NA    NA    NA    NA    NA    NA
Ca21Chr1_0069149 -0.23 -0.16 -0.21  1.12 -0.01  0.35 
Ca21Chr1_0069208 -0.04 -0.01 -0.02  0.10 -0.20  0.56  orf19.6064>
Ca21Chr1_0069267 -0.04 -0.41 -0.14 -0.22  0.13  0.17 
Ca21Chr1_0069326  0.23  0.04  0.14  0.33 -0.08  0.11 
Ca21Chr1_0069385 -0.43 -0.16 -0.06  0.41 -0.13 -0.06 
Ca21Chr1_0069444  0.33  0.10  0.08 -0.11  0.04 -0.08 
Ca21Chr1_0069503  0.01 -0.18  0.25  0.23 -0.50  0.04 
Ca21Chr1_0069562  0.23  0.20  0.21 -0.09  0.32  0.19 
Ca21Chr1_0069621  0.31  0.44  0.19  0.23  0.41 -0.02 
Ca21Chr1_0069680  0.23 -0.06  0.15  0.01 -0.50 -0.39 
Ca21Chr1_0069739  0.79  0.16  0.30  0.13  0.09  0.47 
Ca21Chr1_0069798  0.28  0.05  0.78  0.26 -0.01 -0.17 
Ca21Chr1_0069857  0.14  0.12  0.17 -0.16 -0.23 -0.49 
Ca21Chr1_0069916  0.42  0.04  0.64  0.07  0.14 -0.40 
Ca21Chr1_0069975 -0.13 -0.20  0.37  0.05  0.22 -0.26 
Ca21Chr1_0070034 -0.13  0.04  0.34  0.19  0.12 -0.26 
Ca21Chr1_0070093  0.34 -0.12  0.07 -0.05  0.30 -0.66 
Ca21Chr1_0070152  0.03  0.22  0.44  0.02 -0.37 -0.42 
Ca21Chr1_0070211  0.03 -0.11  0.47  0.25  0.22 -0.19 
Ca21Chr1_0070270  0.40  0.17  0.19  0.00  0.27 -0.19 
Ca21Chr1_0070329  0.18  0.23  0.37  0.17  0.17 -0.35 
Ca21Chr1_0070388  0.12 -0.01  0.80 -0.02 -0.02 -0.41 
Ca21Chr1_0070447  0.29  0.13  0.19 -0.00  0.35 -0.12 
Ca21Chr1_0070506  0.17  0.29  0.28 -0.09  0.09 -0.40 
Ca21Chr1_0070565  0.45  0.28  0.50  0.14  0.32 -0.13 
Ca21Chr1_0070624  0.08  0.74  0.24  0.03 -0.27 -0.07 
Ca21Chr1_0070683  0.06  0.28  0.17 -0.29  0.53  0.19 
Ca21Chr1_0070742  0.06  0.37  0.02  0.13 -0.13 -0.03 
Ca21Chr1_0070801 -0.22  0.51  0.93 -0.16 -0.06  0.04 
Ca21Chr1_0070860 -0.09  0.23  0.37  0.08  0.25  0.02  orf19.6064|
Ca21Chr1_0070919  0.07  0.19  0.30 -0.01 -0.14  0.14 
Ca21Chr1_0070978  0.19 -0.02  0.49 -0.14  0.21  0.16 
Ca21Chr1_0071037  0.28 -0.12  0.13 -0.10  0.71 -0.10  orf19.6063>
Ca21Chr1_0071096 -0.25 -0.01 -0.10  0.44  0.02  0.09 
Ca21Chr1_0071155  0.27  0.08  0.06  0.37 -0.00 -0.11 
Ca21Chr1_0071214 -0.11 -0.36 -0.00  0.11  0.31 -0.06 
Ca21Chr1_0071273  0.21  0.11 -0.10  0.24  0.14 -0.65 
Ca21Chr1_0071332  0.16 -0.03 -0.11  0.34  0.10 -0.27 
Ca21Chr1_0071391 -0.06  0.06 -0.00  0.47  0.39 -0.47 
Ca21Chr1_0071450  0.13  0.13  0.28 -0.02  0.08 -0.48 
Ca21Chr1_0071509 -0.07  0.31  0.26  0.07  0.04 -0.16 
Ca21Chr1_0071568  0.10  0.24  0.60  0.19  0.08  0.28 
Ca21Chr1_0071627 -0.13  0.18  0.69  0.28 -0.09 -0.09 
Ca21Chr1_0071686  0.33 -0.02  0.35  0.01  0.36  0.09 
Ca21Chr1_0071745  0.00 -0.02  0.36  0.05  0.08 -0.34 
Ca21Chr1_0071804  0.43 -0.09  0.39 -0.14  0.05 -0.05 
Ca21Chr1_0071863  0.44  0.11  0.19  0.12 -0.08  0.22 
Ca21Chr1_0071922 -0.17  0.36  0.30  0.14  0.11 -0.03 
Ca21Chr1_0071981  0.01  0.27  0.22 -0.08  0.20 -0.05 
Ca21Chr1_0072040 -0.30  0.16  0.27 -0.00  0.53  0.14 
Ca21Chr1_0072099 -0.14  0.04  0.16 -0.05  0.16  0.17 
Ca21Chr1_0072158  0.14  0.12 -0.12  0.20 -0.15  0.11 
Ca21Chr1_0072217 -0.01  0.43  0.19  0.01 -0.25 -0.22 
Ca21Chr1_0072276 -0.07  0.35  0.42 -0.08 -0.15 -0.17 
Ca21Chr1_0072335  0.01  0.77  0.17  0.05  0.05  0.21 
Ca21Chr1_0072394  0.10  0.05  0.10 -0.56  0.04 -0.28 
Ca21Chr1_0072453 -0.05  0.13  0.09 -0.19 -0.17 -0.39 
Ca21Chr1_0072512  0.28  0.22 -0.06 -0.02  0.17 -0.14 
Ca21Chr1_0072571  0.28 -0.38  0.62  0.21  0.31 -0.45  orf19.6063|
Ca21Chr1_0072630  0.52 -0.06  0.00 -0.06  0.05 -0.12 
Ca21Chr1_0072689  0.39  0.39  0.47  0.29  0.61  0.34  |orf19.6062.3
Ca21Chr1_0072748  0.27  0.33  0.07 -0.20 -0.01 -0.01 
Ca21Chr1_0072807  0.11  0.28  0.43 -0.19 -0.01  0.22 
Ca21Chr1_0072866 -0.00 -0.23  0.46  0.15 -0.34 -0.05 
Ca21Chr1_0072925  0.38  0.56  0.03 -0.03  0.10 -0.17 
Ca21Chr1_0072984  0.66 -0.21  0.63  0.04 -0.26  0.14 
Ca21Chr1_0073043  0.00 -0.20  0.14 -0.08 -0.33  0.18 
Ca21Chr1_0073102  0.28  0.24  0.16 -0.19 -0.31 -0.52 
Ca21Chr1_0073161  0.37  0.15 -0.02 -0.16 -0.19 -0.04 
Ca21Chr1_0073220  0.25  0.20  0.22  0.01 -0.02 -0.06 
Ca21Chr1_0073279  0.17  0.18  0.34  0.40  0.17 -0.21  <orf19.6062.3
Ca21Chr1_0073338 -0.22 -0.38  0.47  0.32 -0.19 -0.07 
Ca21Chr1_0073397  0.09 -0.20  0.01 -0.19  0.21 -0.01 
Ca21Chr1_0073456  0.33  0.06  0.03  0.24  0.37  0.07 
Ca21Chr1_0073515  0.43 -0.12  0.29 -0.03 -0.09  0.11 
Ca21Chr1_0073574  0.28  0.00  0.49 -0.10 -0.15  0.01  orf19.6062>
Ca21Chr1_0073633  0.03  0.02  0.25 -0.01  0.25 -0.06 
Ca21Chr1_0073692  0.32 -0.08  0.24  0.34 -0.04 -0.13 
Ca21Chr1_0073751  0.19  0.18  0.15  0.00 -0.27 -0.04 
Ca21Chr1_0073810 -0.12  0.12  0.36  0.04  0.12  0.19 
Ca21Chr1_0073869  0.15  0.21  0.44  0.52  0.15 -0.05  orf19.6062|
Ca21Chr1_0073928  0.78 -0.32 -0.01 -0.08 -0.11 -0.55 
Ca21Chr1_0073987  0.22 -0.13  0.31  0.29 -0.28 -0.20 
Ca21Chr1_0074046 -0.05  0.22  0.79  0.36 -0.10 -0.27  |orf19.6061
Ca21Chr1_0074105  0.03  0.17  0.57  0.17  0.20  0.02 
Ca21Chr1_0074164  0.25  0.16  0.43  0.11  0.20  0.05 
Ca21Chr1_0074223  0.08  0.34  0.45  0.09  0.23  0.27 
Ca21Chr1_0074282 -0.17  0.28  0.59  0.21 -0.03  0.33 
Ca21Chr1_0074341  0.11  0.09  0.45  0.31  0.23 -0.39 
Ca21Chr1_0074400 -0.05  0.35  0.44  0.02  0.16 -0.23 
Ca21Chr1_0074459  0.42  0.04  0.41 -0.04  0.03 -0.30 
Ca21Chr1_0074518 -0.28  0.36  0.31 -0.00  0.25  0.06 
Ca21Chr1_0074577  0.29  0.26  0.33 -0.24 -0.35 -0.09 
Ca21Chr1_0074636  0.45  0.20  0.25  0.17  0.31 -0.16 
Ca21Chr1_0074695  0.30  0.14  0.25 -0.16  0.31 -0.28 
Ca21Chr1_0074754  0.29  0.28 -0.02 -0.13 -0.06  0.47 
Ca21Chr1_0074813  0.18 -0.20  0.23 -0.26  0.21 -0.47 
Ca21Chr1_0074872  0.09  0.09  0.24  0.27 -0.02 -0.14 
Ca21Chr1_0074931  0.07  0.02  0.11 -0.00 -0.35 -0.28 
Ca21Chr1_0074990  0.13  0.09  0.02 -0.01  0.06 -0.27 
Ca21Chr1_0075049  0.39  0.20  0.25 -0.08  0.08 -0.06 
Ca21Chr1_0075108  0.21  0.23  0.37 -0.06 -0.10  0.16 
Ca21Chr1_0075167 -0.10  0.28  0.18 -0.05 -0.21  0.12 
Ca21Chr1_0075226  0.19  0.22 -0.32 -0.30 -0.02  0.15 
Ca21Chr1_0075285 -0.26 -0.00 -0.35  0.03 -0.46  0.16 
Ca21Chr1_0075344  0.46  0.32 -0.41  0.02  0.02  0.38  <orf19.6061
Ca21Chr1_0075403  0.97 -0.32 -0.45  0.38  0.17  0.46 
Ca21Chr1_0075462  0.01 -0.43 -1.17  0.56  0.42 -0.03 
Ca21Chr1_0075521  0.17 -0.68 -1.03 -0.13  0.16  0.23  |orf19.6060
Ca21Chr1_0075580  0.13 -0.78 -1.00  0.21 -0.08 -0.08 
Ca21Chr1_0075639 -0.20 -0.39 -1.00  0.28 -0.11 -0.08 
Ca21Chr1_0075698 -0.16 -0.57 -0.32  0.55  0.09 -0.10 
Ca21Chr1_0075757  0.20 -0.10 -0.12  0.36  0.20 -0.21 
Ca21Chr1_0075816  0.90 -0.15  0.67  0.07  0.37  0.18 
Ca21Chr1_0075875  0.17  0.03  0.02  0.67 -0.17  0.03 
Ca21Chr1_0075934  0.37  0.01  0.01 -0.14  0.13 -0.12 
Ca21Chr1_0075993  0.34  0.37  0.13  0.12  0.65  0.14 
Ca21Chr1_0076052  0.22  0.14  0.18 -0.03  0.27  0.06 
Ca21Chr1_0076111 -0.14  0.18  0.15  0.40  0.18  0.46 
Ca21Chr1_0076170  0.67  0.07  0.20 -0.18  0.97  0.21 
Ca21Chr1_0076229  0.52  0.12  0.28  0.02  0.28 -0.09 
Ca21Chr1_0076288  0.10  0.97  0.24 -0.21  0.35  0.43 
Ca21Chr1_0076347  1.40  0.44  0.25 -0.09  0.16 -0.26 
Ca21Chr1_0076406  0.35  0.24  0.35 -0.15  0.07 -0.25 
Ca21Chr1_0076465  0.55  0.13  0.58  0.11  0.41  0.07 
Ca21Chr1_0076524  0.14  0.21  0.38 -0.19  0.00 -0.36 
Ca21Chr1_0076583  0.23 -0.11  0.26 -0.12  0.20 -0.29 
Ca21Chr1_0076642  0.14  0.12  0.49 -0.12  0.04 -0.76 
Ca21Chr1_0076701  0.28  0.40  0.53  0.08  0.00  0.04 
Ca21Chr1_0076760  0.54  0.04  0.46 -0.06  0.06  0.07 
Ca21Chr1_0076819  0.38  0.41  0.43 -0.09  0.05  0.19 
Ca21Chr1_0076878  0.33  0.06  0.63  0.00  0.17  0.02 
Ca21Chr1_0076937  1.36  0.14  0.58  0.36 -0.13  0.08 
Ca21Chr1_0076996  0.46  0.01  0.35  0.26  0.29  0.02 
Ca21Chr1_0077055  0.33  0.43  0.34  0.12 -0.13 -0.23 
Ca21Chr1_0077114  0.03 -0.15  0.33 -0.15  0.22 -0.08 
Ca21Chr1_0077173  0.43  0.27  0.32  0.38  0.05 -0.12 
Ca21Chr1_0077232  0.12  0.34  0.13  0.01 -0.53 -0.45 
Ca21Chr1_0077291  0.19  0.04  0.26  0.16  0.30 -0.26 
Ca21Chr1_0077350  0.41  0.24  0.15  0.11  0.03 -0.21 
Ca21Chr1_0077409  0.38 -0.03  0.15 -0.01 -0.04  0.31 
Ca21Chr1_0077468  0.03 -0.01  0.40  0.22 -0.10 -0.05 
Ca21Chr1_0077527  0.33 -0.46  0.12  0.12  0.58  0.04 
Ca21Chr1_0077586  0.58  0.11  0.14 -0.08  0.08 -0.05 
Ca21Chr1_0077645  0.38  0.21  0.23 -0.04  0.00 -0.20 
Ca21Chr1_0077704  0.71  0.14  0.37 -0.01 -0.21 -0.26 
Ca21Chr1_0077763  0.42  0.39  0.21  0.06  0.16  0.12 
Ca21Chr1_0077822 -0.17  0.08  0.21 -0.23  0.07  0.08  <orf19.6060
Ca21Chr1_0077881 -0.16 -0.17  0.02  0.24  0.13 -0.07 
Ca21Chr1_0077940  0.77  0.16  0.25 -0.13  0.88  0.09 
Ca21Chr1_0077999 -0.24  0.18  0.41  0.04 -0.40 -0.16  |orf19.6059
Ca21Chr1_0078058 -0.35  0.09  0.32 -0.06 -0.20  0.28 
Ca21Chr1_0078117  0.41  0.27  0.10 -0.49  0.18 -0.19 
Ca21Chr1_0078176  0.29 -0.01  0.11  0.04 -0.77 -0.75 
Ca21Chr1_0078235  0.30 -0.29  0.03 -0.25 -0.19 -0.32 
Ca21Chr1_0078294  1.10 -0.01 -0.18 -0.21 -0.40 -0.11 
Ca21Chr1_0078353 -0.35 -0.43 -0.44  0.08 -0.47  0.11  <orf19.6059
Ca21Chr1_0078412 -0.23 -0.24 -0.52 -0.06  0.05 -0.01 
Ca21Chr1_0078471 -0.15 -0.19 -0.40 -0.09 -0.12  0.02 
Ca21Chr1_0078530 -0.00  0.03 -0.61 -0.32 -0.09 -0.06 
Ca21Chr1_0078589  0.70 -0.29 -0.35  0.10  0.01 -0.14  |orf19.6058
Ca21Chr1_0078648  0.09 -0.43 -0.30  0.14 -0.01 -0.35 
Ca21Chr1_0078707  0.97  0.01 -0.12 -0.26  0.91 -0.13 
Ca21Chr1_0078766  0.26  0.07  0.12  0.16 -0.18 -0.44 
Ca21Chr1_0078825  0.63  0.22  0.24 -0.20  0.22 -0.32 
Ca21Chr1_0078884  0.38  0.47  0.49 -0.09  0.16  0.02 
Ca21Chr1_0078943  0.41  0.13  0.44 -0.37  0.32 -0.30 
Ca21Chr1_0079002 -0.08  0.05  1.30 -0.12 -0.68  0.32 
Ca21Chr1_0079061  0.26  0.29  0.09 -0.18 -0.04 -0.16 
Ca21Chr1_0079120  0.73  0.14  0.12 -0.05  0.15 -0.11 
Ca21Chr1_0079179 -0.26 -0.16  0.17 -0.26  0.21  0.01 
Ca21Chr1_0079238  0.55 -0.10 -0.09 -0.13  0.03 -0.02 
Ca21Chr1_0079297  0.27  0.07  0.17 -0.03 -0.02  0.04 
Ca21Chr1_0079356  0.77 -0.21 -0.18 -0.25  0.26  0.18 
Ca21Chr1_0079415  0.12  0.08 -0.15 -0.03 -0.09  0.30 
Ca21Chr1_0079474  0.22 -0.10  0.09 -0.21 -0.21  0.20 
Ca21Chr1_0079533  1.38 -0.14  0.13 -0.33  0.72  0.43 
Ca21Chr1_0079592  0.20  0.25  0.40 -0.02  0.12  0.09 
Ca21Chr1_0079651  0.08  0.08  0.12 -0.41 -0.22 -0.17  <orf19.6058
Ca21Chr1_0079710  0.57  0.21 -0.13 -0.47 -0.57 -0.48 
Ca21Chr1_0079769  0.18 -0.23 -0.33 -0.51  0.07 -0.28 
Ca21Chr1_0079828  0.05 -0.11 -0.59 -0.39 -0.34 -0.59 
Ca21Chr1_0079887  0.37 -0.11 -0.34 -0.23 -0.16 -0.31 
Ca21Chr1_0079946  0.17 -0.17 -0.34 -0.30 -0.06 -0.23 
Ca21Chr1_0080005 -0.38 -0.12 -0.23 -0.33  0.73 -0.14 
Ca21Chr1_0080064  0.41  0.00 -0.26 -0.33 -0.16 -0.12 
Ca21Chr1_0080123  0.09  0.05  0.16 -0.58  0.07 -0.16  orf19.6057>
Ca21Chr1_0080182  0.08 -0.02  0.24 -0.49 -0.14  0.03 
Ca21Chr1_0080241  0.05  0.37  0.14 -0.27 -0.17  0.09 
Ca21Chr1_0080300 -0.03  0.28 -0.07 -0.27 -0.17 -0.04 
Ca21Chr1_0080359  0.10  0.14  0.32 -0.01  0.27 -0.15 
Ca21Chr1_0080418  0.80  0.38  0.09  0.15 -0.38 -0.23 
Ca21Chr1_0080477  0.05  0.15  0.00  0.06  0.04 -0.20 
Ca21Chr1_0080536  0.17  0.26  0.14  0.22  0.37 -0.09 
Ca21Chr1_0080595  0.18  0.16  0.22  0.21  0.39  0.11 
Ca21Chr1_0080654  0.24  0.15  0.11  0.09  0.08 -0.33 
Ca21Chr1_0080713  0.49  0.76  0.14  0.05  0.20 -0.01 
Ca21Chr1_0080772  0.29  0.07 -0.12 -0.23  0.18 -0.22 
Ca21Chr1_0080831  0.84  0.17 -0.27  0.02 -0.17 -0.14 
Ca21Chr1_0080890 -0.15 -0.11  0.04  0.03  0.13 -0.20 
Ca21Chr1_0080949 -0.14 -0.17 -0.32  0.14  0.06 -0.11 
Ca21Chr1_0081008 -0.10 -0.04 -0.50  0.20  0.37 -0.32  orf19.6057|
Ca21Chr1_0081067 -0.03  0.44 -0.38  0.12  0.07  0.33 
Ca21Chr1_0081126  0.81  0.31 -0.02  0.09 -0.32  0.32 
Ca21Chr1_0081185  0.12 -0.08  0.00 -0.15  0.11 -0.37  orf19.6056>
Ca21Chr1_0081244  0.01  0.52 -0.02  0.24 -0.07  0.24 
Ca21Chr1_0081303 -0.06  0.07 -0.10  0.10 -0.12 -0.10 
Ca21Chr1_0081362  0.14  0.09  0.09  0.06  0.36  0.32 
Ca21Chr1_0081421 -0.15  0.21 -0.31 -0.21  0.32 -0.02 
Ca21Chr1_0081480 -0.15  0.01  0.01  0.03  0.37  0.10 
Ca21Chr1_0081539 -0.41  0.00  0.02  0.29 -0.16 -0.27 
Ca21Chr1_0081598 -0.64 -0.02 -0.57  0.09 -0.15 -0.36 
Ca21Chr1_0081657 -0.60 -0.12 -0.86 -0.34  0.24 -0.34 
Ca21Chr1_0081716 -1.45 -0.34 -1.44  0.07  0.07  0.44 
Ca21Chr1_0081775 -1.76 -0.74 -1.55  0.25 -0.15 -0.01 
Ca21Chr1_0081834 -2.02 -1.27 -2.16  0.21  0.07  0.56 
Ca21Chr1_0081893 -2.12 -0.41 -1.93  0.22  0.08 -0.20  orf19.6056|
Ca21Chr1_0081952 -3.36 -0.70 -1.25  0.09 -0.78  0.52 
Ca21Chr1_0082011 -1.74 -0.68 -1.82 -0.13  0.07  0.43 
Ca21Chr1_0082070 -0.62 -1.10 -1.28 -0.29  0.03 -0.17  |orf19.6055
Ca21Chr1_0082129 -0.97 -1.01 -0.69 -0.06  0.08 -0.01 
Ca21Chr1_0082188 -0.57 -0.21 -0.23 -0.02  0.07 -0.09 
Ca21Chr1_0082247  0.49  0.10 -0.21 -0.03 -0.16 -0.33 
Ca21Chr1_0082306 -0.08 -0.08  0.17  0.10 -0.15  0.06 
Ca21Chr1_0082365 -0.61 -0.13  0.09  0.32  0.17 -0.29 
Ca21Chr1_0082424  0.04  0.13  0.16  0.11  0.14 -0.50 
Ca21Chr1_0082483  0.00 -0.22  0.48  0.10  0.10 -0.24 
Ca21Chr1_0082542  0.11  0.25  0.15  0.10  0.08 -0.16 
Ca21Chr1_0082601  0.15  0.21  0.29  0.25  0.03 -0.07 
Ca21Chr1_0082660  0.36  0.18  0.35  0.12 -0.15  0.26 
Ca21Chr1_0082719  0.34  0.24  0.45  0.20  0.17  0.17 
Ca21Chr1_0082778  0.17  0.43  0.31  0.09  0.23  0.02 
Ca21Chr1_0082837 -0.14  0.43  0.53  0.25 -0.28 -0.09 
Ca21Chr1_0082896  0.19  0.06  0.25  0.10  0.31 -0.21 
Ca21Chr1_0082955  0.12 -0.00  0.36  0.27  0.07  0.11 
Ca21Chr1_0083014  0.24  0.15  0.44 -0.03  0.18 -0.05 
Ca21Chr1_0083073  1.25  0.44  0.71  0.34  0.26 -0.07 
Ca21Chr1_0083132  0.19  0.46  0.45  0.16  0.40 -0.05 
Ca21Chr1_0083191  0.55  0.66  0.60  0.27  0.80 -0.15 
Ca21Chr1_0083250  0.75  0.53  0.65  0.09  0.02 -0.29 
Ca21Chr1_0083309  0.19  0.35  0.33  0.14 -0.34 -0.01 
Ca21Chr1_0083368 -0.04  0.21  0.57  0.45 -0.21 -0.03 
Ca21Chr1_0083427  0.13  0.44  0.26  0.18  0.12 -0.15 
Ca21Chr1_0083486  0.19  0.06  0.36  0.23  0.16 -0.02 
Ca21Chr1_0083545  0.02  0.09  0.19  0.22  0.01  0.30 
Ca21Chr1_0083604  0.27  0.08 -0.08  0.54  0.13  0.03 
Ca21Chr1_0083663  0.12  0.30 -0.03  0.01 -0.05 -0.13 
Ca21Chr1_0083722  0.18  0.16  0.41  0.08 -0.36 -0.35 
Ca21Chr1_0083781  0.27  0.41  0.12 -0.06 -0.02  0.04 
Ca21Chr1_0083840  0.19  0.29 -0.02  0.02 -0.06 -0.07 
Ca21Chr1_0083899 -0.08 -0.05  0.25  0.34 -0.13 -0.40 
Ca21Chr1_0083958 -0.18 -0.09  0.22 -0.23  0.64 -0.05 
Ca21Chr1_0084017  0.01  0.08  0.35  0.05 -0.07 -0.24 
Ca21Chr1_0084076 -0.10  0.23  0.26  0.18  0.29 -0.04 
Ca21Chr1_0084135  0.24  0.06 -0.00 -0.12 -0.01 -0.04 
Ca21Chr1_0084194  0.34  0.27  0.05 -0.02 -0.08 -0.49 
Ca21Chr1_0084253  0.35  0.37  0.14 -0.52 -0.00 -0.41 
Ca21Chr1_0084312 -0.16  0.33  0.14 -0.40 -0.12 -0.09 
Ca21Chr1_0084371  0.24  0.24 -0.27  0.22  0.27 -0.23 
Ca21Chr1_0084430  0.05  0.13 -0.23  0.04 -0.12 -0.05 
Ca21Chr1_0084489  0.13  0.01 -0.64  0.08 -0.52 -0.15 
Ca21Chr1_0084548 -0.12 -0.13 -1.14  0.12  0.03 -0.18  <orf19.6055
Ca21Chr1_0084607 -0.42 -0.46 -1.23  0.17  0.47 -0.69 
Ca21Chr1_0084666 -0.66  0.00 -1.85 -0.14  0.08  1.35 
Ca21Chr1_0084725 -1.04 -1.06 -1.05  0.17 -0.15 -0.14 
Ca21Chr1_0084784 -0.78 -0.98 -1.89  0.52 -0.19 -0.05 
Ca21Chr1_0084843  0.15 -1.25 -1.32  0.34 -0.42  0.07 
Ca21Chr1_0084902  0.09 -0.11 -0.69 -0.16  0.04  0.11 
Ca21Chr1_0084961 -0.27 -0.32 -0.45  0.27  0.07  0.46 
Ca21Chr1_0085020  0.29  0.03 -0.32 -0.01  0.02  0.21 
Ca21Chr1_0085079 -0.05 -0.16  0.00  0.02  0.45 -0.00  |orf19.6054
Ca21Chr1_0085138 -0.00  0.28  0.01  0.29  0.54  0.30 
Ca21Chr1_0085197 -0.16  0.17 -0.11 -0.04 -0.09  0.17 
Ca21Chr1_0085256  1.04  0.43  0.26 -0.11  0.43 -0.01 
Ca21Chr1_0085315  0.51  0.15  0.36 -0.11  0.16  0.02 
Ca21Chr1_0085374  0.22 -0.08  0.26  0.15 -0.09  0.16 
Ca21Chr1_0085433  0.07 -0.52  0.05  0.27  0.02  0.14 
Ca21Chr1_0085492  0.13  0.03  0.33  0.48  0.15  0.24 
Ca21Chr1_0085551  0.37  0.33  0.35  0.67  0.04  0.27 
Ca21Chr1_0085610  0.07  0.44  0.48  0.73  0.03  0.33 
Ca21Chr1_0085669  0.18  0.38  0.38 -0.03  0.15 -0.13 
Ca21Chr1_0085728 -0.00  0.30  0.01 -0.00  0.08 -0.04 
Ca21Chr1_0085787  0.02  0.26  0.21 -0.02 -0.10  0.11 
Ca21Chr1_0085846 -0.00  0.24 -0.01 -0.25 -0.16  0.10 
Ca21Chr1_0085905 -0.38  0.13  0.27  0.21 -0.39  0.53 
Ca21Chr1_0085964  0.09  0.30  0.36  0.38 -0.04  0.02 
Ca21Chr1_0086023  0.90  0.16  0.16  0.04  0.29  0.00 
Ca21Chr1_0086082  0.14  0.08  0.53  0.33  0.06  0.13 
Ca21Chr1_0086141  0.20 -0.46  0.20 -0.05  0.24 -0.24 
Ca21Chr1_0086200  0.48  0.32  0.30 -0.06  0.52 -0.07 
Ca21Chr1_0086259  0.08  0.05  0.16 -0.04  0.07 -0.06 
Ca21Chr1_0086318  0.20 -0.03  0.39  1.81 -0.05 -0.12 
Ca21Chr1_0086377  0.18  0.22  0.16  0.15  0.27 -0.16 
Ca21Chr1_0086436 -0.00 -0.04 -0.20 -0.30  0.24 -0.24 
Ca21Chr1_0086495  0.39 -0.15  0.05  0.04  0.16 -0.05 
Ca21Chr1_0086554  0.11 -0.06  0.40 -0.19  0.25  0.08 
Ca21Chr1_0086613  0.09 -0.24  0.20  0.10 -0.51 -0.25 
Ca21Chr1_0086672  0.14 -0.08  0.27  0.02  0.05  0.08 
Ca21Chr1_0086731  0.52  0.09  0.43 -0.04 -0.28 -0.03 
Ca21Chr1_0086790 -0.00  0.22  0.25 -0.19 -0.04 -0.44 
Ca21Chr1_0086849 -0.11  0.13  0.14 -0.27  0.20 -0.60 
Ca21Chr1_0086908  0.23 -0.13  0.39 -0.01  0.19 -0.14 
Ca21Chr1_0086967  0.24  0.04  0.34 -0.24  0.18 -0.12 
Ca21Chr1_0087026  0.41 -0.05  0.66 -0.18  0.08 -0.12 
Ca21Chr1_0087085  0.24  0.12  0.60  0.04 -0.20 -0.13 
Ca21Chr1_0087144  0.04  0.22  0.11 -0.05 -0.01 -0.20 
Ca21Chr1_0087203 -0.02  0.33  0.35 -0.16 -0.75 -0.14  <orf19.6054
Ca21Chr1_0087262  0.16  0.36 -0.02 -0.05 -0.40 -0.22 
Ca21Chr1_0087321  0.16  0.23  0.22  0.05 -0.06 -0.09 
Ca21Chr1_0087380  0.04 -0.20 -0.13 -0.20 -0.30  0.21 
Ca21Chr1_0087439  0.03  0.03 -0.21  0.31 -0.06  0.22 
Ca21Chr1_0087498 -1.04 -0.43 -0.22 -0.15 -0.36 -0.09 
Ca21Chr1_0087557 -0.04  0.44 -0.33 -0.09 -0.30 -0.09 
Ca21Chr1_0087616 -0.09 -0.32 -0.55  0.48 -0.07  0.41 
Ca21Chr1_0087675 -0.10 -0.11 -0.51  0.16  0.33 -0.10 
Ca21Chr1_0087734  0.13 -0.09 -0.34  0.19 -0.39  0.14 
Ca21Chr1_0087793 -0.10  0.19 -0.02  0.05 -0.26  0.10 
Ca21Chr1_0087852 -0.04 -0.03  0.61 -0.05 -0.11  0.08  orf19.6053>
Ca21Chr1_0087911  0.10  0.18  0.12  0.01  0.03 -0.03 
Ca21Chr1_0087970  0.43  0.13  0.40  0.02 -0.17  0.11 
Ca21Chr1_0088029  0.19  0.27  0.39 -0.10 -0.24 -0.15 
Ca21Chr1_0088088  0.14  0.10  0.17  0.06 -0.09 -0.39 
Ca21Chr1_0088147  0.30  0.19  0.51  0.21  0.26 -0.20 
Ca21Chr1_0088206  0.38  0.11  0.00  0.16  0.24 -0.22 
Ca21Chr1_0088265  0.28  0.27  0.23  0.48 -0.16 -0.56 
Ca21Chr1_0088324 -0.08  0.14  0.29 -0.05  0.12 -0.39 
Ca21Chr1_0088383  0.27  0.33  0.22  0.05  0.31 -0.36 
Ca21Chr1_0088442  0.30  0.12  0.16 -0.23  0.36 -0.22 
Ca21Chr1_0088501  0.40 -0.01  0.22 -0.23  0.05  0.87 
Ca21Chr1_0088560  0.28  0.22 -0.19  0.21  0.06  0.35 
Ca21Chr1_0088619  0.26  0.33  0.03  0.01  0.13 -0.05 
Ca21Chr1_0088678 -0.25 -0.05  0.01 -0.17  0.82  0.23 
Ca21Chr1_0088737 -0.02 -0.08  0.31 -0.18  0.16 -0.19 
Ca21Chr1_0088796  0.25  0.12  0.29 -0.53 -0.05  0.20 
Ca21Chr1_0088855  0.82  0.12  0.73 -0.02  0.04  0.45 
Ca21Chr1_0088914  0.41  0.25  0.11 -0.38  0.14  0.10 
Ca21Chr1_0088973  0.44 -0.04  0.26  0.17  0.25 -0.05 
Ca21Chr1_0089032 -0.10  0.24  0.12 -0.03  0.08  0.08 
Ca21Chr1_0089091  0.20  0.13 -0.20  0.05 -0.00 -0.12 
Ca21Chr1_0089150  0.19  0.10  0.34 -0.02 -0.04  0.26 
Ca21Chr1_0089209 -0.10  0.22  0.42  0.80  0.27  0.28 
Ca21Chr1_0089268  0.31  0.27  0.15 -0.10  0.12  0.12 
Ca21Chr1_0089327  0.86  0.33  0.31  0.26  0.05 -0.15 
Ca21Chr1_0089386  0.22  0.26  0.38  0.10  0.04 -0.51 
Ca21Chr1_0089445  0.09  0.14  0.26 -0.22  0.31  0.01 
Ca21Chr1_0089504  0.47  0.05  0.38  0.09 -0.04  0.28 
Ca21Chr1_0089563  0.24  0.16  0.21 -0.01 -0.09 -0.09 
Ca21Chr1_0089622 -3.29  0.81  0.36  0.04 -0.15  0.06 
Ca21Chr1_0089681  0.10 -0.00  0.12  0.24  0.02 -0.01 
Ca21Chr1_0089740 -0.17  0.30  0.23 -0.06 -0.06 -0.21 
Ca21Chr1_0089799 -0.15  0.07  0.26 -0.15  0.01 -0.15  orf19.6053|
Ca21Chr1_0089858  0.48 -0.19  0.13  0.49  0.04 -0.32 
Ca21Chr1_0089917  0.43 -0.33  0.00  0.16  0.26 -0.44 
Ca21Chr1_0089976  0.23  0.04  0.23  0.14  0.03  0.10 
Ca21Chr1_0090035 -0.02 -0.21  0.02  0.14  0.17  0.10 
Ca21Chr1_0090094  0.10  0.00  0.13  0.08  0.01 -0.14  orf19.6052>
Ca21Chr1_0090153  0.18  0.08  0.15 -0.18  0.16 -0.69 
Ca21Chr1_0090212  0.18 -0.02  0.45  0.20  0.16 -0.25 
Ca21Chr1_0090271  0.18 -0.09  0.08  0.19  0.17 -0.22 
Ca21Chr1_0090330  0.29 -0.05  0.25  0.17  0.40 -0.18 
Ca21Chr1_0090389  0.28  0.10  0.46  0.49  0.74 -0.32 
Ca21Chr1_0090448  0.33  0.07  0.11  0.20  0.34 -0.09 
Ca21Chr1_0090507  0.54  0.23  0.18 -0.07  0.11 -0.20 
Ca21Chr1_0090566  0.13 -0.01  0.41  0.16  0.13  0.04 
Ca21Chr1_0090625  0.02  0.03 -0.03 -0.00  0.20 -0.07 
Ca21Chr1_0090684 -0.03 -0.12  0.33  0.15  0.22 -0.27 
Ca21Chr1_0090743 -0.08  0.06  0.29 -0.05 -0.57 -0.54 
Ca21Chr1_0090802 -0.48  0.30  0.33  0.16 -0.02 -0.79 
Ca21Chr1_0090861 -0.28  0.21  0.32  0.67  0.06 -0.21 
Ca21Chr1_0090920  0.04  0.53 -0.07  0.11 -0.22 -0.22 
Ca21Chr1_0090979  0.07  0.24  0.33  0.15  0.22 -0.59 
Ca21Chr1_0091038 -0.05 -0.06  0.13  0.09  0.38  0.08 
Ca21Chr1_0091097  0.06 -0.01  0.03  0.22  0.25 -0.04 
Ca21Chr1_0091156  0.28  0.07  0.32  0.16  0.34 -0.18 
Ca21Chr1_0091215 -0.06  0.24  0.20  0.45  0.28 -0.15  orf19.6052|
Ca21Chr1_0091274    NA    NA    NA    NA    NA    NA |orf19.6049
Ca21Chr1_0091333  0.58  0.00  0.44 -0.25  0.40  0.17 
Ca21Chr1_0091392  0.59  0.19  0.24 -0.08  0.25  0.35 
Ca21Chr1_0091451  0.43 -0.17  0.34 -0.40  0.04  0.13 
Ca21Chr1_0091510  0.18  0.31  0.47 -0.11  0.09  0.20 
Ca21Chr1_0091569  0.57  0.32  0.00 -0.02 -0.26 -0.02 
Ca21Chr1_0091628 -0.75  0.03  0.34 -0.15 -0.08 -0.57 
Ca21Chr1_0091687  0.18  0.22  0.58 -0.38  0.05 -0.21 
Ca21Chr1_0091746  0.36  0.36  0.66  0.41 -0.05 -0.42 
Ca21Chr1_0091805  0.26  0.29  0.47 -0.24  0.10  0.06 
Ca21Chr1_0091864  0.20  0.30  0.60 -0.31  0.23 -0.08 
Ca21Chr1_0091923  0.29  0.12  0.35  0.19  0.22  0.00 
Ca21Chr1_0091982  0.08 -0.05  0.21  0.11  0.14 -0.10 
Ca21Chr1_0092041  0.34  0.23  0.26  0.05  0.08 -0.02 
Ca21Chr1_0092100  0.26  0.20  0.48  0.20 -0.06 -0.18 
Ca21Chr1_0092159  0.51  0.19  0.20 -0.20  0.25 -0.02 
Ca21Chr1_0092218 -0.50  0.16 -0.10 -0.08 -0.24 -0.16 
Ca21Chr1_0092277  0.86  0.27  0.41 -0.18  0.28 -0.15 
Ca21Chr1_0092336  0.25  0.32  0.54  0.28  0.29 -0.24 
Ca21Chr1_0092395  0.18  0.17  0.70  0.15  0.16 -0.53 
Ca21Chr1_0092454  0.26  0.05  0.30  0.29  0.07 -0.07 
Ca21Chr1_0092513  0.22 -0.06  0.73  0.34 -0.10 -0.20 
Ca21Chr1_0092572  0.19  0.22  0.50  0.11  0.02 -0.23 
Ca21Chr1_0092631 -0.34  0.21  0.26  0.82 -0.19  0.05 
Ca21Chr1_0092690  0.19 -0.08  0.13 -0.07  0.05  0.09 
Ca21Chr1_0092749  0.33  0.16  0.20 -0.32  0.04  0.30 
Ca21Chr1_0092808 -0.03  0.16  0.59 -0.05  0.04  0.23 
Ca21Chr1_0092867  0.39  0.17  0.45 -0.19  0.62 -0.13 
Ca21Chr1_0092926  0.25  0.21  0.46 -0.02  0.07  0.16 
Ca21Chr1_0092985  0.21  0.09  0.28  0.41  0.10 -0.01 
Ca21Chr1_0093044  0.24  0.40  0.58 -0.08  0.53 -0.32 
Ca21Chr1_0093103  0.29  0.39  0.27 -0.49  0.32 -0.83 
Ca21Chr1_0093162  0.31  0.04  0.51 -0.07  0.12  0.18 
Ca21Chr1_0093221  0.18  0.11  0.13  0.24 -0.11 -0.15 
Ca21Chr1_0093280  0.16  0.34  0.58  0.11  0.08  0.06 
Ca21Chr1_0093339  0.02  0.20  0.31  0.31 -0.23 -0.33 
Ca21Chr1_0093398  0.39  0.10  0.24  1.11  0.14 -0.09 
Ca21Chr1_0093457  0.04  0.23 -0.03  0.05  0.18  0.21 
Ca21Chr1_0093516 -0.09  0.00 -0.07 -0.11  0.04  0.41 
Ca21Chr1_0093575 -0.03  0.02 -0.13  0.30 -0.22  0.44 
Ca21Chr1_0093634  0.18  0.18 -0.09 -0.19  0.41 -0.05 
Ca21Chr1_0093693 -0.05  0.22 -0.53  0.00 -0.43  0.02  <orf19.6049
Ca21Chr1_0093752 -0.11 -0.18 -0.18  0.03 -0.01 -0.19 
Ca21Chr1_0093811  0.01 -0.50 -0.30 -0.06  0.06 -0.15 
Ca21Chr1_0093870 -0.80 -0.14 -0.14  0.01 -0.47 -0.20 
Ca21Chr1_0093929 -0.55  0.14 -0.18  0.08  0.14 -0.45  orf19.6048>
Ca21Chr1_0093988  0.20  0.05  0.15  0.29  0.02 -0.16 
Ca21Chr1_0094047  1.11  0.11  0.33  0.81 -0.21  0.18 
Ca21Chr1_0094106 -0.33  0.24  0.26 -0.25 -0.13  0.20 
Ca21Chr1_0094165  0.46  0.04  0.20  0.32  0.05  0.01 
Ca21Chr1_0094224  0.30  0.22  0.39  0.07  0.17  0.52 
Ca21Chr1_0094283  0.41  0.34  0.24 -0.14 -0.43 -0.07 
Ca21Chr1_0094342  0.13 -0.06  0.32 -0.11 -0.20 -0.19 
Ca21Chr1_0094401  0.44  0.57  0.31 -0.02  0.52  0.68 
Ca21Chr1_0094460  0.32  0.08  0.49  0.22  0.11 -0.46 
Ca21Chr1_0094519  0.17  0.26  0.74  0.08  0.08 -0.04 
Ca21Chr1_0094578  0.46  0.52  0.54  0.27  0.20 -0.54 
Ca21Chr1_0094637  0.08  0.10  0.03 -0.31  0.25 -0.45 
Ca21Chr1_0094696  0.23  0.05  0.30 -0.11 -0.13 -0.54 
Ca21Chr1_0094755  0.13  0.08  0.31  0.31  0.06 -0.24 
Ca21Chr1_0094814  1.29  0.22  0.57 -0.03 -0.19 -0.21 
Ca21Chr1_0094873  0.15  0.03  0.44  0.01  0.15 -0.84  orf19.6048|
Ca21Chr1_0094932  0.65  0.12 -0.17 -0.35  0.35 -0.66 
Ca21Chr1_0094991  0.13  0.35  0.05 -0.04  0.11 -0.26 
Ca21Chr1_0095050  0.03 -0.19  0.13 -0.19  0.20  0.14 
Ca21Chr1_0095109 -0.36  0.23 -0.26  0.16 -0.38  0.10 
Ca21Chr1_0095168  0.62  0.03  0.13 -0.17  0.13 -0.12  orf19.6047>
Ca21Chr1_0095227  0.35  0.45  0.39 -0.04  0.15 -0.20 
Ca21Chr1_0095286  0.28  0.29  0.32 -0.18  0.32 -0.23 
Ca21Chr1_0095345  0.14 -0.42  0.11 -0.13  0.18 -0.01 
Ca21Chr1_0095404 -0.31  0.20 -0.05 -0.20  0.15  0.02 
Ca21Chr1_0095463  0.01 -0.27  0.05 -0.17  0.14  0.02 
Ca21Chr1_0095522 -0.31 -0.01 -0.12 -0.36 -0.35  0.10 
Ca21Chr1_0095581  0.03  0.79  0.14 -0.44  0.26 -0.32 
Ca21Chr1_0095640  0.15  0.17 -0.08 -0.17 -0.23 -0.25 
Ca21Chr1_0095699 -0.50  0.14  0.32 -0.48  0.25 -0.13 
Ca21Chr1_0095758  0.22  0.24  0.50  0.02  0.16  0.09 
Ca21Chr1_0095817  0.07 -0.42 -0.24  0.04  0.10 -0.07 
Ca21Chr1_0095876  0.05  0.11  0.37  0.37  0.46 -0.18 
Ca21Chr1_0095935  0.51  0.29 -0.07  0.23  0.41 -0.01 
Ca21Chr1_0095994 -0.05  0.08  0.24  0.16  0.26 -0.16 
Ca21Chr1_0096053  0.08  0.12  0.05 -0.13  0.26  0.17 
Ca21Chr1_0096112 -0.13  0.35  0.20 -0.15 -0.15  0.11 
Ca21Chr1_0096171  0.06  0.05  0.20  0.27  0.48 -0.02 
Ca21Chr1_0096230 -0.30  0.01  0.01  0.96 -0.29  0.56 
Ca21Chr1_0096289  0.11 -0.01  0.05  0.37  0.58 -0.04 
Ca21Chr1_0096348 -0.12  0.02 -0.04 -0.01  0.32  0.13 
Ca21Chr1_0096407 -0.92  0.08 -0.14 -0.04  0.43  0.04 
Ca21Chr1_0096466 -0.05 -0.01 -0.08 -0.00  0.05 -0.05  orf19.6047|
Ca21Chr1_0096525 -0.13  0.10  0.02  0.03 -0.17 -0.27 
Ca21Chr1_0096584  0.03  0.19  0.04 -0.14 -0.10  0.24 
Ca21Chr1_0096643 -0.17  0.47  0.11  0.26  0.14  0.40 
Ca21Chr1_0096702 -0.03 -0.16 -0.18  0.34  0.23  0.04 
Ca21Chr1_0096761  0.06  0.09  0.21 -0.14 -0.07 -0.00  orf19.6046>
Ca21Chr1_0096820 -0.89  0.12  0.30 -0.33  0.27 -0.12 
Ca21Chr1_0096879  0.31  0.20  0.25 -0.02  0.13 -0.13 
Ca21Chr1_0096938  0.15  0.05  0.55 -0.06  0.05 -0.11 
Ca21Chr1_0096997  0.68  0.29  0.10 -0.60  0.22 -0.70 
Ca21Chr1_0097056  0.09  0.20  0.31 -0.13 -0.03 -0.28 
Ca21Chr1_0097115  0.19 -0.02  0.49  0.29  0.14 -0.24 
Ca21Chr1_0097174  0.38  0.19  0.40  0.65  0.04  0.85 
Ca21Chr1_0097233  0.58  0.15  0.10  0.08 -0.15 -0.10 
Ca21Chr1_0097292  0.34  0.04  0.15  0.11  0.43 -0.42 
Ca21Chr1_0097351  0.27  0.55  0.33  0.09  0.14 -0.25 
Ca21Chr1_0097410 -0.09 -0.04  0.06  0.29 -0.02  0.06 
Ca21Chr1_0097469  0.21  0.06  0.50  0.20  0.26 -0.18 
Ca21Chr1_0097528  0.17  0.30  0.13 -0.22  0.29  0.07 
Ca21Chr1_0097587 -0.18  0.16  0.34  0.21  0.40 -0.35 
Ca21Chr1_0097646  0.29  0.31  0.33  0.21  0.18 -0.23 
Ca21Chr1_0097705  0.08  0.14  0.17  0.22  0.27 -0.37 
Ca21Chr1_0097764 -0.01  0.20  0.02  0.38 -0.20 -0.03 
Ca21Chr1_0097823  0.26  0.27  0.28  0.28  0.22 -0.21 
Ca21Chr1_0097882 -0.04 -0.06  0.10 -0.09  0.56 -0.08 
Ca21Chr1_0097941  0.26 -0.04  0.46  0.06 -0.21 -0.06 
Ca21Chr1_0098000  0.30 -0.05  0.31  0.00  0.04 -0.08 
Ca21Chr1_0098059  0.08  0.11  0.13 -0.23  0.38 -0.39 
Ca21Chr1_0098118 -0.43  0.09  0.05  0.74 -0.47 -0.16 
Ca21Chr1_0098177  0.55  0.18  0.25  0.07  0.06 -0.05 
Ca21Chr1_0098236  0.35  0.19  0.47  0.10 -0.34 -0.70 
Ca21Chr1_0098295  0.47  0.57  0.54  0.24 -0.27 -0.01 
Ca21Chr1_0098354  0.02 -0.01  0.46  0.07 -0.17 -0.32 
Ca21Chr1_0098413  0.14  0.25  0.02 -0.03  0.37 -0.09 
Ca21Chr1_0098472  0.14  0.24  0.41 -0.13  0.06 -0.15 
Ca21Chr1_0098531  0.31  0.19  0.25  0.17  0.25 -0.22 
Ca21Chr1_0098590 -0.07  0.53  0.40  0.31  0.33 -0.22 
Ca21Chr1_0098649 -0.14  0.19  0.37 -0.01  0.11 -0.32 
Ca21Chr1_0098708  0.37  0.11  0.56  0.17  0.09 -0.19 
Ca21Chr1_0098767  0.12  1.12  0.47 -0.10  0.57 -0.20 
Ca21Chr1_0098826  0.31  0.31  0.49  0.10  0.31 -0.02 
Ca21Chr1_0098885  0.56 -0.05  0.28  0.26  0.13 -0.13 
Ca21Chr1_0098944  0.28  0.04  0.40  0.28  0.49  0.21 
Ca21Chr1_0099003  0.15 -0.21  0.29  0.06  0.34 -0.64 
Ca21Chr1_0099062  0.27  0.13  0.13 -0.16  0.30 -0.26 
Ca21Chr1_0099121  0.99  0.18  0.24 -0.07  0.28 -0.59 
Ca21Chr1_0099180  0.27  0.24  0.33  0.12  0.05 -0.11 
Ca21Chr1_0099239 -0.00  0.21  0.32  0.17  0.20 -0.15 
Ca21Chr1_0099298  0.09 -0.09  0.09  0.52  0.37 -0.14 
Ca21Chr1_0099357  0.33  0.27  0.33  0.04 -0.04 -0.08 
Ca21Chr1_0099416  0.11 -0.35  0.13  0.05 -0.24 -0.25 
Ca21Chr1_0099475  0.17  0.44  0.18  0.21  0.22 -0.04 
Ca21Chr1_0099534 -0.00  0.10  0.42  0.44 -0.03 -0.17 
Ca21Chr1_0099593  0.03  0.18  0.22  0.03 -0.20 -0.49 
Ca21Chr1_0099652  0.41  0.34  0.20  0.65  0.32 -0.35 
Ca21Chr1_0099711  0.29  0.33  0.04 -0.05 -0.48 -0.50 
Ca21Chr1_0099770  0.41 -0.05  0.05 -0.35 -0.12 -0.61 
Ca21Chr1_0099829 -0.05 -0.03  0.11 -0.13  0.19 -0.37 
Ca21Chr1_0099888  0.08 -0.44 -0.80 -0.17  0.26 -0.36 
Ca21Chr1_0099947 -0.10 -0.18 -0.33  0.01  0.00 -0.08 
Ca21Chr1_0100006  0.16 -0.40 -0.12 -0.10  0.10 -0.23 
Ca21Chr1_0100065 -0.20 -0.45  0.34 -0.18 -0.07 -0.32 
Ca21Chr1_0100124 -0.01  0.03 -0.26 -0.15  0.56 -0.08 
Ca21Chr1_0100183 -0.33 -0.05 -0.11 -0.27  0.52 -0.50 
Ca21Chr1_0100242 -0.13  0.16  0.18 -0.05 -0.03 -0.31 
Ca21Chr1_0100301  0.72  0.02 -0.08 -0.32  0.22 -0.30 
Ca21Chr1_0100360  0.20 -0.24 -0.02 -0.05 -0.07 -0.65 
Ca21Chr1_0100419  0.94  0.17 -0.03  0.16  0.29 -0.39 
Ca21Chr1_0100478  0.14  0.15  0.29  0.01  0.14 -0.26 
Ca21Chr1_0100537  0.17  0.13 -0.01  0.00  0.34 -0.03 
Ca21Chr1_0100596  0.12  0.39  0.22  0.03  0.23 -0.11 
Ca21Chr1_0100655  0.24  0.32  0.09  0.05  0.31 -0.15 
Ca21Chr1_0100714  0.14  0.12  0.51  0.24  0.12 -0.02 
Ca21Chr1_0100773  0.06  0.26  0.23 -0.36  0.16 -0.22 
Ca21Chr1_0100832  0.18  0.13  0.36  0.13 -0.00  0.08 
Ca21Chr1_0100891 -0.14  0.23 -0.13  0.23  0.50 -0.67 
Ca21Chr1_0100950 -0.02  0.61 -0.61 -0.01  0.08 -0.30 
Ca21Chr1_0101009 -0.27 -0.21 -0.53  0.16 -0.11 -0.21 
Ca21Chr1_0101068 -0.11 -0.21 -0.31  0.39 -0.07 -0.19 
Ca21Chr1_0101127 -0.57 -0.30 -0.51 -0.47 -0.10 -0.15 
Ca21Chr1_0101186 -0.10 -0.19 -0.12  0.10  0.03 -0.21 
Ca21Chr1_0101245 -0.19 -0.70 -0.48  0.13 -0.73 -0.16 
Ca21Chr1_0101304 -0.86 -0.08 -1.12  0.36 -0.57  0.74 
Ca21Chr1_0101363 -0.00 -0.16 -1.19 -0.26  0.17  1.00 
Ca21Chr1_0101422 -0.46 -0.14 -0.98  0.41 -0.01 -0.25  orf19.6046|
Ca21Chr1_0101481 -0.91 -0.10 -1.05  0.05  0.44 -0.00 
Ca21Chr1_0101540 -0.36 -0.82  0.23 -0.21 -0.48  0.03 
Ca21Chr1_0101599 -1.29 -0.99 -0.84  0.57  0.22 -0.04 
Ca21Chr1_0101658 -0.26 -0.88 -1.49  0.09 -0.02 -0.18 
Ca21Chr1_0101717  0.88 -1.08 -1.05 -0.20  0.32  0.06  orf19.6045>
Ca21Chr1_0101776 -0.08 -0.63 -0.75  0.08  0.14  0.10 
Ca21Chr1_0101835 -0.01 -0.51 -0.44  0.05  0.15  0.03 
Ca21Chr1_0101894  0.10 -0.25 -0.85  0.02 -0.25  0.11 
Ca21Chr1_0101953 -0.20 -0.23 -0.76 -0.18 -0.46  0.23 
Ca21Chr1_0102012  0.28 -0.24 -0.38  0.48 -0.41  0.73 
Ca21Chr1_0102071 -0.10 -0.14 -0.41 -0.01  0.17  0.14 
Ca21Chr1_0102130  0.01 -0.03 -0.01  0.90  0.11  0.55 
Ca21Chr1_0102189 -0.13  0.07  0.21 -0.23  0.01 -0.05 
Ca21Chr1_0102248  0.02  0.44  0.32 -0.07 -0.00 -0.35 
Ca21Chr1_0102307 -0.02  0.19  0.34  0.28 -0.06 -0.37 
Ca21Chr1_0102366  0.09 -0.04  0.16 -0.06 -0.00 -0.28 
Ca21Chr1_0102425  0.62  0.12 -0.06  0.22  0.21  0.14 
Ca21Chr1_0102484  0.07  0.16  0.30  0.24  1.03 -0.04 
Ca21Chr1_0102543  0.07  0.17  0.49  0.37 -0.24 -0.38 
Ca21Chr1_0102602  0.03  0.13  0.24 -0.09  0.21 -0.01 
Ca21Chr1_0102661  0.17  0.18  0.29 -0.17 -0.00 -0.18 
Ca21Chr1_0102720  0.25 -0.07  0.14 -0.25  0.15 -0.23 
Ca21Chr1_0102779  0.25  0.33  0.45 -0.18  0.33 -0.23 
Ca21Chr1_0102838 -0.07  0.11 -0.16 -0.51  0.22 -0.41 
Ca21Chr1_0102897  0.15  0.05  0.75 -0.23  0.11 -0.16 
Ca21Chr1_0102956  0.55 -0.22  0.48 -0.32 -0.28 -0.18 
Ca21Chr1_0103015  0.50  0.11  0.20 -0.25  0.01 -0.06 
Ca21Chr1_0103074  0.12  0.20 -0.04  0.00  1.06  0.17 
Ca21Chr1_0103133 -0.04  0.15  0.08 -0.24 -0.17 -0.03 
Ca21Chr1_0103192  0.28 -0.10  0.11 -0.21 -0.19  0.10 
Ca21Chr1_0103251  0.16 -0.02 -0.01 -0.30 -0.84 -0.13 
Ca21Chr1_0103310 -0.09  0.04  0.17  0.31 -0.03  0.04 
Ca21Chr1_0103369 -0.09  0.47  0.69  0.00  0.05 -0.01 
Ca21Chr1_0103428 -0.51  0.12 -0.43 -0.02  0.57  0.31 
Ca21Chr1_0103487  0.05  0.38  0.02 -0.03 -0.06 -0.23  orf19.6045|
Ca21Chr1_0103546  0.57  0.45 -0.07 -0.16 -0.06 -0.19 
Ca21Chr1_0103605  0.15  0.28 -0.44 -0.07 -0.11 -0.12 
Ca21Chr1_0103664  0.97 -0.05 -0.38  0.15  0.09  0.17 
Ca21Chr1_0103723 -0.60 -0.42 -0.24 -0.41  0.17  0.05 
Ca21Chr1_0103782 -0.29 -0.01 -0.47  0.28 -0.14 -0.02 
Ca21Chr1_0103841  0.19 -0.84 -0.18  0.21  0.20 -0.03  orf19.6044>
Ca21Chr1_0103900 -0.03  0.09 -0.03  0.17 -0.12 -0.21 
Ca21Chr1_0103959  0.44  0.19  0.90 -0.13  0.19  0.12 
Ca21Chr1_0104018  0.06  0.17  0.22  0.04  0.17 -0.45 
Ca21Chr1_0104077  0.08  0.58  0.34  0.19  0.09 -0.16 
Ca21Chr1_0104136  0.36  0.28  0.44  0.27  0.24 -0.21 
Ca21Chr1_0104195  0.29  0.95  0.79  0.34 -0.12 -0.09 
Ca21Chr1_0104254  0.38  0.14  0.39  0.31 -0.28 -0.11 
Ca21Chr1_0104313  0.47  0.19  0.46 -0.45  0.20  0.21 
Ca21Chr1_0104372  0.29  0.27  0.29  0.21 -0.09 -0.05 
Ca21Chr1_0104431 -0.02  0.40  0.48 -0.02  0.25 -0.01 
Ca21Chr1_0104490  0.45  0.14  0.05  0.10  0.24  0.16 
Ca21Chr1_0104549  0.42  0.06  0.46  0.10 -0.01 -0.17 
Ca21Chr1_0104608  0.35  0.29  0.10 -0.24 -0.16 -0.14 
Ca21Chr1_0104667  0.22  0.25  0.43  0.09  0.27 -0.13 
Ca21Chr1_0104726  0.07  0.07  0.27  0.12  0.08 -0.43 
Ca21Chr1_0104785  0.38  0.24  0.14  0.37 -0.01 -0.20  orf19.6044|
Ca21Chr1_0104844  0.12  0.40  0.41 -0.19  0.32 -0.21 
Ca21Chr1_0104903 -0.08  0.34  0.25  0.28  0.15  0.04 
Ca21Chr1_0104962  1.12  0.02  0.21 -0.05  0.05  0.15 
Ca21Chr1_0105021  0.06 -0.05  0.12  0.58  0.23  0.38 
Ca21Chr1_0105080  0.06 -0.23 -0.43  0.02  0.19 -0.26 
Ca21Chr1_0105139 -0.11  0.15 -0.01 -0.34  0.01 -0.25  orf19.6043>
Ca21Chr1_0105198  0.13 -0.35  0.18 -0.10  0.01 -0.03 
Ca21Chr1_0105257 -0.04 -0.04  0.24 -0.05  0.11 -0.38 
Ca21Chr1_0105316  0.26  0.23  0.40  0.03 -0.35 -0.14 
Ca21Chr1_0105375  0.25  0.01  0.48 -0.26 -0.31 -0.24 
Ca21Chr1_0105434  0.52  0.21  0.55  0.10 -0.10 -0.17 
Ca21Chr1_0105493  0.18  0.05  0.52  0.03  0.07 -0.25 
Ca21Chr1_0105552  0.11  0.67  0.35 -0.17 -0.10  0.02 
Ca21Chr1_0105611 -0.06 -0.04  0.41  0.07 -0.03  0.75 
Ca21Chr1_0105670  0.18 -0.05  0.34  0.20 -0.05 -0.02 
Ca21Chr1_0105729  0.43  0.19  0.67 -0.02  0.05 -0.08 
Ca21Chr1_0105788  0.22  0.10  1.04  0.29  0.15 -0.01 
Ca21Chr1_0105847  0.27  0.46  0.52 -0.26  0.24 -0.06 
Ca21Chr1_0105906  0.18  0.22  0.40  0.34  0.25 -0.05 
Ca21Chr1_0105965  1.00  0.25  0.29  0.27  0.39  0.67 
Ca21Chr1_0106024 -0.03 -0.21  0.20  0.12  0.48 -0.24 
Ca21Chr1_0106083  0.05  0.17  0.32  0.16 -0.07 -0.31 
Ca21Chr1_0106142  0.14  0.28  0.39  0.23  0.15  0.54 
Ca21Chr1_0106201  0.06 -0.04  0.31 -0.12  0.13 -0.49 
Ca21Chr1_0106260  0.62  0.01  0.30  0.18  0.04 -0.10 
Ca21Chr1_0106319  0.14 -0.07  0.02  0.35 -0.35 -0.16 
Ca21Chr1_0106378  0.09 -0.07  0.15  0.55 -0.02  0.06 
Ca21Chr1_0106437  0.16 -0.06  0.02  0.10  0.22  0.26 
Ca21Chr1_0106496 -0.20 -0.13 -0.22 -0.02 -0.01 -0.05 
Ca21Chr1_0106555 -0.31  0.03 -0.22  0.12 -0.37 -0.11 
Ca21Chr1_0106614 -0.29 -0.20 -0.10 -0.09 -0.02 -0.47 
Ca21Chr1_0106673 -0.65 -0.09 -0.27  0.76 -0.45  0.15 
Ca21Chr1_0106732 -0.64 -0.34  0.00 -0.19  0.06 -0.20 
Ca21Chr1_0106791  0.57 -0.03  0.18  0.15  0.37 -0.02 
Ca21Chr1_0106850  0.48 -0.30  0.08  0.18  0.20 -0.16 
Ca21Chr1_0106909  0.01  0.04  0.23  0.13 -0.13  0.14  orf19.6043|
Ca21Chr1_0106968  0.63  0.61  0.30 -0.09  0.54  0.12 
Ca21Chr1_0107027  0.56  0.09  0.27  0.12  0.29 -0.15 
Ca21Chr1_0107086  0.39  0.06  0.21 -0.01  0.07  0.15  |orf19.6041
Ca21Chr1_0107145  0.36  0.00  0.27 -0.13 -0.01 -0.05 
Ca21Chr1_0107204  0.28 -0.19  0.10 -0.02  0.32  0.07 
Ca21Chr1_0107263  0.21  0.20  0.00  0.01 -0.01  0.02 
Ca21Chr1_0107322  0.14  0.18  0.39  0.13 -0.28  0.05 
Ca21Chr1_0107381  0.55  0.20  0.60  0.17 -0.17  0.38 
Ca21Chr1_0107440  0.23 -0.08  0.52 -0.04 -0.12  0.32 
Ca21Chr1_0107499  0.23  0.01  0.44 -0.01 -0.04  0.75 
Ca21Chr1_0107558  0.22  0.06  0.65  0.01 -0.03  0.03 
Ca21Chr1_0107617  0.25  0.13  0.48 -0.13  0.00  0.30 
Ca21Chr1_0107676  0.67  0.72  0.47  0.54  0.06 -0.14 
Ca21Chr1_0107735  0.33  0.10  0.58 -0.00  0.02 -0.38 
Ca21Chr1_0107794  0.53  0.10  0.14  0.08  0.05 -0.45 
Ca21Chr1_0107853 -0.03  0.02  0.64  0.55  0.17 -0.20 
Ca21Chr1_0107912  0.09  0.25 -0.11  0.09  0.20 -0.56 
Ca21Chr1_0107971  0.07  0.30 -0.14 -0.04  0.20 -0.29 
Ca21Chr1_0108030  0.40  0.38  0.18 -0.16  0.27  0.16 
Ca21Chr1_0108089  0.33  0.31  0.38  0.51  0.90  0.05 
Ca21Chr1_0108148  0.19  0.10  0.61  0.16  0.41  0.10 
Ca21Chr1_0108207  0.10 -0.17  0.41  0.27  0.16 -0.16 
Ca21Chr1_0108266  0.28  0.08  0.18  0.06 -0.24  0.08 
Ca21Chr1_0108325  0.16  0.28  0.34 -0.09  0.33 -0.34 
Ca21Chr1_0108384  0.38 -0.57  0.66  0.33  0.62 -0.19 
Ca21Chr1_0108443  0.27  0.32  0.58  0.40  0.52  0.05 
Ca21Chr1_0108502  0.10  0.02  0.21  0.40  0.69 -0.06 
Ca21Chr1_0108561  0.61 -0.30  0.51 -0.14  0.47 -0.26 
Ca21Chr1_0108620  0.21 -0.10  0.32 -0.09  0.14  0.03 
Ca21Chr1_0108679  0.15  0.01  0.54  0.22  0.91 -0.04 
Ca21Chr1_0108738  0.24  0.46  0.41  0.20  0.17  0.14 
Ca21Chr1_0108797  0.23  0.36  0.32  0.21 -0.02 -0.02 
Ca21Chr1_0108856  0.24  0.25  0.47  0.04  0.20  0.07 
Ca21Chr1_0108915  0.18  0.42  0.51  0.41 -0.03 -0.10 
Ca21Chr1_0108974  0.23  0.46  0.50  0.32 -0.12  0.16 
Ca21Chr1_0109033  0.36  0.24  0.29 -0.18  0.46 -0.25 
Ca21Chr1_0109092  0.39  0.01 -0.01  0.09 -0.02  0.16 
Ca21Chr1_0109151  0.30 -0.08 -0.21  0.33  0.43 -0.16 
Ca21Chr1_0109210  0.16  0.09  0.23  0.21  0.20 -0.58 
Ca21Chr1_0109269 -0.00  0.21  0.06  0.10  0.20 -0.88 
Ca21Chr1_0109328  0.29 -0.03  0.33  0.27  0.35 -0.17 
Ca21Chr1_0109387  0.38 -0.02  0.20  0.24  0.06 -0.20 
Ca21Chr1_0109446  0.70 -0.01  0.23  0.08  0.11  0.22 
Ca21Chr1_0109505 -0.04  0.01  0.23  0.16  0.20  0.05 
Ca21Chr1_0109564  0.48  0.06  0.18  0.08  0.19 -0.04 
Ca21Chr1_0109623  0.22  0.41  0.32 -0.06  0.38  0.08 
Ca21Chr1_0109682  0.26  0.15  0.43 -0.13  0.15  0.01 
Ca21Chr1_0109741  0.42  0.31  0.32  0.24  0.35 -0.10 
Ca21Chr1_0109800  0.47  0.03  0.52  0.71  1.01  0.03 
Ca21Chr1_0109859  0.14  0.20  0.26 -0.23 -0.10  0.09 
Ca21Chr1_0109918  0.28  0.13  0.46  0.13  0.01 -0.15 
Ca21Chr1_0109977  0.31  0.21  0.17  0.23  0.17 -0.09 
Ca21Chr1_0110036  0.48  0.16  0.21  0.11  0.01  0.20 
Ca21Chr1_0110095  0.30  0.11  0.23  0.36 -0.13 -0.01 
Ca21Chr1_0110154  0.27  0.25  0.06  0.09  0.33  0.01 
Ca21Chr1_0110213 -0.44  0.11  0.10  0.15  0.23  0.19 
Ca21Chr1_0110272  0.10 -0.23  0.20  0.38 -0.26  0.08 
Ca21Chr1_0110331  0.20  0.19  0.51 -0.03 -0.34  0.15 
Ca21Chr1_0110390  0.34  0.25  0.37 -0.00 -0.46  0.13 
Ca21Chr1_0110449  0.15  0.01  0.30 -0.04 -0.12  0.29 
Ca21Chr1_0110508  0.26 -0.13  0.55  0.16 -0.02  0.11 
Ca21Chr1_0110567  0.40 -0.19  0.25 -0.16 -0.14  0.57 
Ca21Chr1_0110626  0.27  0.21  0.28  0.31  0.27  0.44 
Ca21Chr1_0110685  0.29  0.29  0.30  0.11 -0.18  0.33 
Ca21Chr1_0110744  0.19  0.15 -0.26 -0.16 -0.00 -0.05 
Ca21Chr1_0110803 -0.09 -0.08 -0.33  0.21 -0.10 -0.26 
Ca21Chr1_0110862  0.17  0.04 -0.11 -0.02  0.68 -0.38 
Ca21Chr1_0110921  1.23  0.08  0.63  0.04 -0.08 -0.46 
Ca21Chr1_0110980  0.13  0.25 -0.35 -0.15  0.22 -0.24  <orf19.6041
Ca21Chr1_0111039 -0.25  0.19 -0.17  0.30 -0.07  0.22 
Ca21Chr1_0111098  0.08  0.15 -0.31  0.29 -0.06 -0.03 
Ca21Chr1_0111157  0.03 -0.30 -0.93  0.70  0.98 -0.14 
Ca21Chr1_0111216 -0.08 -0.06  0.10  0.08 -0.06  0.21 
Ca21Chr1_0111275  0.37 -0.03 -0.12 -0.04 -0.12 -0.01 
Ca21Chr1_0111334  0.29  0.30 -0.02  0.03 -0.05 -0.05 
Ca21Chr1_0111393  0.07  0.08 -0.40  0.20 -0.18 -0.41 
Ca21Chr1_0111452  0.28 -0.00  0.16 -0.01 -0.00  0.01 
Ca21Chr1_0111511  0.22  0.23  0.54 -0.02  0.02  0.33 
Ca21Chr1_0111570  0.21  0.39  0.70  0.07  0.01  0.41  |orf19.6040
Ca21Chr1_0111629  0.12  0.41  0.91  0.26  0.25  0.36 
Ca21Chr1_0111688  0.32  0.42  0.80 -0.02 -0.12  0.31 
Ca21Chr1_0111747  0.31  0.08  0.52 -0.14  0.66 -0.05 
Ca21Chr1_0111806  0.27  0.29  0.57 -0.02 -0.06  0.30 
Ca21Chr1_0111865  0.50  0.14  0.48  0.14  0.31 -0.12 
Ca21Chr1_0111924  0.40 -0.00  0.47 -0.11 -0.18  0.11 
Ca21Chr1_0111983  0.25  0.23  0.50  0.02 -0.04  0.23 
Ca21Chr1_0112042  0.01  0.15  0.09  0.05 -0.41  0.05 
Ca21Chr1_0112101  0.36  0.40  0.44 -0.09 -0.06  0.14 
Ca21Chr1_0112160  0.33  0.29  0.58 -0.14  0.08  0.08 
Ca21Chr1_0112219  0.23  0.08 -0.15 -0.13  0.16  0.33  <orf19.6040
Ca21Chr1_0112278  0.56  0.03  0.20 -0.11 -0.27  0.26 
Ca21Chr1_0112337  0.07  0.60  0.15  0.16 -0.00  0.55 
Ca21Chr1_0112396  0.29  0.19 -0.01 -0.02 -0.03  0.70 
Ca21Chr1_0112455  0.50 -0.39  1.34  0.06 -0.50  0.22 
Ca21Chr1_0112514  0.10  0.26  0.20  0.23 -0.37 -0.15 
Ca21Chr1_0112573  1.08  0.63  0.13 -0.04 -0.08  0.25 
Ca21Chr1_0112632  0.66 -0.13 -0.56 -0.13  0.02  0.53 
Ca21Chr1_0112691  0.54  0.07 -0.03  0.25  1.40  0.51 
Ca21Chr1_0112750  0.81 -0.07 -0.04 -0.26 -0.11  0.25  |orf19.6039
Ca21Chr1_0112809 -0.09  0.11 -0.10  0.03 -0.13 -0.06 
Ca21Chr1_0112868  0.46  0.32  0.18 -0.16  0.09  0.10 
Ca21Chr1_0112927  0.29  0.91  0.20  0.10 -0.10  0.00 
Ca21Chr1_0112986  0.25 -0.06  0.30 -0.44 -0.25  0.19 
Ca21Chr1_0113045  0.42  0.65  0.13 -0.12  0.09  0.09 
Ca21Chr1_0113104  0.31 -0.10  0.26  0.22 -0.30  0.05 
Ca21Chr1_0113163  0.07  0.23  0.56 -0.01 -0.32 -0.15 
Ca21Chr1_0113222  0.30  0.28  0.27  0.00  0.08 -0.18 
Ca21Chr1_0113281  0.30  0.13  0.24 -0.01 -0.05  0.21 
Ca21Chr1_0113340  0.03  0.11  0.18  0.42 -0.13 -0.32 
Ca21Chr1_0113399 -0.88  0.19 -0.21  0.36 -0.12 -0.12 
Ca21Chr1_0113458  0.14 -0.14  0.25  0.18  0.20 -0.13 
Ca21Chr1_0113517  0.22  0.20  0.20 -0.07 -0.02 -0.49 
Ca21Chr1_0113576  0.14 -0.00  0.14 -0.13 -0.56 -0.08 
Ca21Chr1_0113635 -0.48  0.06  0.17  0.07 -0.51  0.35 
Ca21Chr1_0113694  0.11 -0.07  0.45 -0.02 -0.22  0.08 
Ca21Chr1_0113753  0.21  0.25  0.02 -0.18 -0.68 -0.26  <orf19.6039
Ca21Chr1_0113812  0.25 -0.15  0.42 -0.05 -0.75  0.04 
Ca21Chr1_0113871  0.10 -0.16  0.20  0.08  0.11 -0.48 
Ca21Chr1_0113930  0.26  0.08  0.07 -0.03 -0.07  0.07 
Ca21Chr1_0113989  0.04 -0.03  0.19 -0.22  0.07  0.01 
Ca21Chr1_0114048  0.06  0.26 -0.03 -0.12 -0.38  0.21 
Ca21Chr1_0114107 -0.05  0.24  0.01  0.15  0.00 -0.07  |orf19.6038
Ca21Chr1_0114166  0.13  0.32  0.01  0.03  0.10  0.12 
Ca21Chr1_0114225 -0.48  0.03  0.06  0.13 -0.32 -0.33 
Ca21Chr1_0114284 -0.06 -0.24  0.12 -0.59 -0.15 -0.18 
Ca21Chr1_0114343  0.62  0.82  0.81 -0.01 -0.01 -0.15 
Ca21Chr1_0114402  0.32  0.36  0.27  0.25  0.17 -0.12 
Ca21Chr1_0114461  0.65  0.16 -0.10  0.03  0.07 -0.07 
Ca21Chr1_0114520 -0.00  0.09 -0.36 -0.02 -0.03 -0.21 
Ca21Chr1_0114579  0.39 -0.15 -0.51 -0.29 -0.07 -0.23 
Ca21Chr1_0114638 -0.08 -0.33 -0.29 -0.15  0.19 -0.10 
Ca21Chr1_0114697 -0.01 -0.49 -0.54 -0.13 -0.39 -0.10 
Ca21Chr1_0114756 -0.31 -0.16  0.02 -0.32 -0.18 -0.20 
Ca21Chr1_0114815 -0.09 -0.21 -0.45 -0.16 -0.16  0.15 
Ca21Chr1_0114874 -0.04  0.00 -0.25  0.06 -0.05  0.24 
Ca21Chr1_0114933  0.06 -0.10 -0.46 -0.12  0.24  0.11 
Ca21Chr1_0114992  0.46 -0.23 -0.21 -0.09 -0.71 -0.13 
Ca21Chr1_0115051  0.23  0.04 -0.08 -0.28  0.36 -0.25 
Ca21Chr1_0115110  0.21  0.05  0.08  0.22 -0.01 -0.07 
Ca21Chr1_0115169  0.47  0.29  0.13  0.23  0.11  0.24 
Ca21Chr1_0115228  1.20 -0.15  0.21  0.28 -0.38  0.07 
Ca21Chr1_0115287  0.26 -0.03  0.13  0.19 -0.42 -0.34 
Ca21Chr1_0115346  0.18  0.22  0.30  0.17  0.00  0.00 
Ca21Chr1_0115405  0.37  0.51  2.03  0.12 -0.10 -0.16 
Ca21Chr1_0115464  0.34  0.28  0.23  0.09  0.25 -0.08 
Ca21Chr1_0115523  0.05  0.19  0.11 -0.18 -0.15 -0.33 
Ca21Chr1_0115582 -0.15  0.06  0.08  0.12 -0.51 -0.31 
Ca21Chr1_0115641  0.17  0.17 -0.09 -0.19  0.06  0.08 
Ca21Chr1_0115700 -0.03  0.16 -0.02 -0.50 -0.23 -0.06 
Ca21Chr1_0115759  0.13  0.10 -0.19 -0.01  0.00 -0.08 
Ca21Chr1_0115818  0.16  0.00 -0.12  0.05 -0.00  0.13 
Ca21Chr1_0115877 -0.11 -0.30 -0.29  0.06 -0.01  0.13  <orf19.6038
Ca21Chr1_0115936 -0.01  0.06 -0.32  0.27 -0.82  0.82 
Ca21Chr1_0115995 -0.07 -0.21  0.11  0.23 -0.30 -0.08 
Ca21Chr1_0116054  0.00 -0.06 -0.17  0.04 -0.20 -0.16 
Ca21Chr1_0116113 -0.30  0.04 -0.34 -0.12 -0.35  0.17 
Ca21Chr1_0116172 -0.21 -0.11 -0.19 -0.11 -0.13  0.28 
Ca21Chr1_0116231  0.16  0.74 -0.03 -0.32 -0.27  0.30 
Ca21Chr1_0116290 -0.09  0.03 -0.47 -0.24  0.29  0.35 
Ca21Chr1_0116349  0.16  0.65 -0.52  0.24 -0.17  0.05 
Ca21Chr1_0116408  0.04 -0.00 -0.44 -0.14  0.08  0.16 
Ca21Chr1_0116467  0.95 -0.05 -0.03 -0.21 -0.80  0.48 
Ca21Chr1_0116526 -0.49 -0.09 -0.65 -0.27 -0.35  0.56 
Ca21Chr1_0116585 -0.30 -0.19 -0.78 -0.33 -0.08  1.14 
Ca21Chr1_0116644  0.33 -0.16 -1.08 -0.16 -0.10  0.20 
Ca21Chr1_0116703 -0.60 -0.45 -1.97 -0.05 -0.28 -0.22 
Ca21Chr1_0116762 -0.52 -0.91 -2.15 -0.23 -0.12 -0.14 
Ca21Chr1_0116821 -0.35 -1.58 -2.00 -0.02 -0.16  0.77 
Ca21Chr1_0116880 -1.08 -1.59 -2.04 -0.34 -0.10 -0.36 
Ca21Chr1_0116939 -1.16 -1.39 -1.76 -0.15  0.15 -0.27 
Ca21Chr1_0116998 -1.11 -1.63 -1.64 -0.01 -0.03 -0.15 
Ca21Chr1_0117057 -0.66 -1.22 -0.59  1.26 -0.26 -0.02 
Ca21Chr1_0117116 -0.44 -1.21 -1.09  0.14  0.21 -0.65 
Ca21Chr1_0117175  0.51 -0.97 -1.13 -0.25 -0.52 -0.09 
Ca21Chr1_0117234 -0.26 -0.20 -0.41  0.09 -0.11  0.57 
Ca21Chr1_0117293 -0.04 -0.08 -0.24 -0.36 -0.06 -0.14  orf19.6037>
Ca21Chr1_0117352  0.16  0.10 -0.04 -0.40  0.09 -0.32 
Ca21Chr1_0117411  0.12  0.13 -0.00  0.07 -0.22 -0.24 
Ca21Chr1_0117470  0.16  0.31  0.04 -0.17 -0.23 -0.30 
Ca21Chr1_0117529 -0.09  0.17  0.08 -0.13  0.20 -0.42 
Ca21Chr1_0117588  0.11  0.14  0.13  0.13  0.00 -0.35 
Ca21Chr1_0117647  0.22 -0.07  0.26  0.27  0.09  0.02 
Ca21Chr1_0117706  0.18  0.11  0.12  0.10 -0.03 -0.08 
Ca21Chr1_0117765  0.41  0.21  0.50  0.05  0.18 -0.18 
Ca21Chr1_0117824  0.17 -0.12  0.21  0.09  0.33 -0.39 
Ca21Chr1_0117883  0.26  0.13  0.01 -0.21 -0.11 -0.62 
Ca21Chr1_0117942  0.22  0.06  0.17  0.29  0.12 -0.34 
Ca21Chr1_0118001  0.14  0.25  0.24  0.07  0.13 -0.28 
Ca21Chr1_0118060 -0.03 -0.12  0.15  0.01 -0.14  0.54 
Ca21Chr1_0118119  0.23  0.20  0.40  0.08 -0.16  0.16 
Ca21Chr1_0118178 -0.40  0.06  0.11 -0.03  0.27  0.22 
Ca21Chr1_0118237  0.17  0.13  0.43  0.09  0.03 -0.11 
Ca21Chr1_0118296  0.24  0.01  0.29  0.29  0.16  0.11 
Ca21Chr1_0118355  0.25 -0.05  0.53  0.10  0.01 -0.18 
Ca21Chr1_0118414  0.47  0.09  0.03  0.34  0.41 -0.35 
Ca21Chr1_0118473  0.17  0.24  0.33  0.01 -0.24 -0.28 
Ca21Chr1_0118532  0.10 -0.14  0.47  0.07  0.17 -0.34 
Ca21Chr1_0118591  0.02  0.50  0.04  0.09 -0.61 -0.18 
Ca21Chr1_0118650  0.51  0.26  0.60  0.12  0.20 -0.19 
Ca21Chr1_0118709  0.35  0.32  0.07 -0.02 -0.29 -0.71 
Ca21Chr1_0118768 -0.09  0.05  0.29 -0.02  0.12 -0.30 
Ca21Chr1_0118827  0.35  0.62  0.33  0.59  0.02  0.49 
Ca21Chr1_0118886 -0.16  0.34  0.20 -0.05  0.12 -0.24 
Ca21Chr1_0118945  0.30 -0.26  0.26 -0.18  0.17  0.24 
Ca21Chr1_0119004  0.16  0.02  0.28  0.24  0.03  0.23 
Ca21Chr1_0119063 -0.17 -0.64  0.56  0.01  0.10  0.10 
Ca21Chr1_0119122  0.12  0.27  0.48 -0.11 -0.20 -0.10 
Ca21Chr1_0119181  0.45  0.32  0.24  0.51  0.04 -0.30 
Ca21Chr1_0119240 -0.20  0.20  0.09  0.30 -0.46  0.04 
Ca21Chr1_0119299  0.26  0.23  0.29  0.21  0.25 -0.36 
Ca21Chr1_0119358  0.15  0.19  0.26  0.05 -0.09 -0.12 
Ca21Chr1_0119417  0.34  0.12  0.30 -0.05 -0.25  0.12  orf19.6037|
Ca21Chr1_0119476  0.00  0.10 -0.31  0.24  0.81  0.74 
Ca21Chr1_0119535  0.31 -0.18  0.37  0.07  0.00 -0.05 
Ca21Chr1_0119594  0.44 -0.04  0.32 -0.00 -0.32  0.41 
Ca21Chr1_0119653  0.08 -0.11  0.25  0.08  0.20  0.33 
Ca21Chr1_0119712  0.00  0.10  0.03 -0.17 -0.01 -0.04  orf19.6036>
Ca21Chr1_0119771 -0.04  0.01  0.11 -0.18 -0.14 -0.47 
Ca21Chr1_0119830 -0.26 -0.00  0.32  0.13 -0.09 -0.21 
Ca21Chr1_0119889 -0.82 -0.14  0.14 -0.46 -1.08  0.40 
Ca21Chr1_0119948 -0.27  0.06  0.03  0.23 -0.25 -0.23 
Ca21Chr1_0120007 -0.28  0.06  0.02  0.31  0.10 -1.23 
Ca21Chr1_0120066  0.18 -0.58  0.37 -0.00 -0.18  0.30 
Ca21Chr1_0120125  0.30  0.28  0.25  0.23  0.05  0.29 
Ca21Chr1_0120184  0.23 -0.03  0.13 -0.04 -0.08  0.17 
Ca21Chr1_0120243 -0.29  0.11  0.16  0.26  0.13 -0.65 
Ca21Chr1_0120302  0.14  0.33 -0.17 -0.17 -0.54 -0.94 
Ca21Chr1_0120361 -0.31  0.08  0.05 -0.21 -0.24  0.31 
Ca21Chr1_0120420  0.00 -0.23  0.22  0.23 -0.00 -0.65 
Ca21Chr1_0120479  0.14  0.18  0.04  0.07  0.36  0.13 
Ca21Chr1_0120538  0.05 -0.13 -0.51  0.23  0.37 -0.22 
Ca21Chr1_0120597  0.13  0.27 -0.22  0.20  0.32  0.38 
Ca21Chr1_0120656  0.74  0.26  0.15 -0.35  0.13  0.43 
Ca21Chr1_0120715 -0.01  0.07  1.47  0.22  0.19 -0.79 
Ca21Chr1_0120774  0.05  0.23  0.59  0.05 -0.13  0.16 
Ca21Chr1_0120833  0.06  0.31  0.24  0.07  0.17  0.05 
Ca21Chr1_0120892 -0.08  0.10  0.30 -0.00  0.45 -0.19 
Ca21Chr1_0120951  0.51  0.39  0.31  0.31  0.19  0.34 
Ca21Chr1_0121010  0.07  0.28  0.46  0.14 -0.23 -0.23 
Ca21Chr1_0121069 -0.19  0.13  0.38  0.48  0.63 -0.10 
Ca21Chr1_0121128  0.05  0.05  0.29  0.11  0.26 -0.12 
Ca21Chr1_0121187  0.16  0.14  0.39  0.12  0.14 -0.49 
Ca21Chr1_0121246 -0.18  0.43  0.61  0.17 -0.00 -0.21 
Ca21Chr1_0121305  0.69  0.15  0.40  0.09 -0.09  0.06 
Ca21Chr1_0121364  0.20 -0.04  0.29  0.10 -0.88  0.58 
Ca21Chr1_0121423  0.11  0.09  0.18  0.30  0.05 -0.22 
Ca21Chr1_0121482  0.30  0.25  0.14  0.25 -0.12 -0.41 
Ca21Chr1_0121541  0.09  0.00  0.16  0.13  0.18 -0.11 
Ca21Chr1_0121600 -0.06  0.14  0.11  0.21  0.18 -0.44 
Ca21Chr1_0121659 -0.35  0.30  0.24  0.31 -0.07  0.12 
Ca21Chr1_0121718  0.21  0.33  0.14  0.17  0.00  0.02 
Ca21Chr1_0121777 -0.17  0.25 -0.13 -0.11  0.08  0.22 
Ca21Chr1_0121836  0.02  0.14  0.21  0.06  0.13 -0.10 
Ca21Chr1_0121895  0.01  0.33  0.56  0.41 -0.38  0.02 
Ca21Chr1_0121954  0.61 -0.24  0.04  0.30 -0.50 -0.69 
Ca21Chr1_0122013  1.11 -0.37 -0.01  0.26 -0.14 -0.02 
Ca21Chr1_0122072 -0.51  0.29  0.01 -0.10  3.00 -0.02 
Ca21Chr1_0122131 -0.44  0.32 -0.05 -0.29 -0.56 -0.55 
Ca21Chr1_0122190 -0.45 -0.02  0.02  0.12 -0.13  0.96 
Ca21Chr1_0122249  0.21  0.35 -0.55 -0.77 -0.18 -1.14 
Ca21Chr1_0122308 -0.18  0.09  0.07  0.21 -0.10  0.33 
Ca21Chr1_0122367  0.72  0.11  0.32 -0.42 -0.36 -0.34 
Ca21Chr1_0122426  1.15  0.15  0.05  0.19 -0.13 -0.16 
Ca21Chr1_0122485 -0.35  0.15 -0.25 -0.20 -0.02  0.06 
Ca21Chr1_0122544 -0.41  0.26  0.02  0.17 -0.27 -0.18 
Ca21Chr1_0122603 -0.26  0.52  1.25  0.09  0.05 -1.22 
Ca21Chr1_0122662 -0.32 -0.08 -0.60  0.01 -0.29  0.33 
Ca21Chr1_0122721 -0.04  0.08 -0.18  0.10 -0.14  0.51 
Ca21Chr1_0122780 -0.14 -0.04  0.08  0.10  0.16  0.42  orf19.6036|
Ca21Chr1_0122839 -0.11 -0.13 -0.11 -0.16 -0.06  0.32 
Ca21Chr1_0122898  0.21 -0.05  0.15  0.28  0.01  0.57 
Ca21Chr1_0122957  0.20  0.09  0.27  0.30 -0.05  0.21 
Ca21Chr1_0123016  0.08  0.08 -0.14 -0.08 -0.50  0.68  orf19.6035>
Ca21Chr1_0123075 -0.24  0.24  0.17  0.69 -0.12 -0.28 
Ca21Chr1_0123134 -0.32  0.38  0.23  0.31 -0.46  0.28 
Ca21Chr1_0123193 -0.28 -0.04  0.28  0.06 -0.22 -0.54 
Ca21Chr1_0123252  0.45 -0.14  0.54 -0.38 -0.24 -0.52 
Ca21Chr1_0123311 -0.44  0.25  0.19 -0.05  0.44 -0.60 
Ca21Chr1_0123370  0.22  0.17  0.45  0.30  0.07  0.66 
Ca21Chr1_0123429 -0.37  0.48 -0.08 -0.14 -0.35 -0.47  orf19.6035|
Ca21Chr1_0123488 -0.07  0.02 -0.29  0.03  0.68  0.16 
Ca21Chr1_0123547  0.21  0.60 -0.32 -0.16  0.55 -0.71 
Ca21Chr1_0123606  0.14  0.75  0.35  0.20  0.11 -0.57 
Ca21Chr1_0123665  0.85  0.16 -0.48 -0.10  0.98  0.00 
Ca21Chr1_0123724  0.11  0.60 -0.00 -0.08 -0.36 -0.01 
Ca21Chr1_0123783  0.34  0.53 -0.19 -0.34 -0.18  0.26 
Ca21Chr1_0123842  0.87  0.14  0.40 -0.16 -0.06 -0.12  |orf19.6034
Ca21Chr1_0123901  0.53  0.28  0.51 -0.03  0.35 -0.24 
Ca21Chr1_0123960  0.19  0.07  0.44 -0.08 -0.08 -0.11 
Ca21Chr1_0124019  0.44  0.24  0.25  0.08 -0.14  0.09 
Ca21Chr1_0124078  0.39  0.33  0.14  0.23  0.18 -0.61 
Ca21Chr1_0124137  0.16  0.19 -0.11  0.12  0.20 -0.37 
Ca21Chr1_0124196  0.46  0.27  0.39  0.21 -0.16 -0.62 
Ca21Chr1_0124255  0.25  0.21  0.40 -0.16  0.13 -0.41 
Ca21Chr1_0124314  0.71  0.07  0.22 -0.04 -0.09 -0.26 
Ca21Chr1_0124373  0.23  0.02  0.21 -0.29 -0.09 -0.41 
Ca21Chr1_0124432  0.55 -0.04  0.09  0.12 -0.23  0.05 
Ca21Chr1_0124491  0.29  0.02  0.07  0.36  0.21  0.05 
Ca21Chr1_0124550  0.35  1.01  0.21 -0.00  0.11  0.21 
Ca21Chr1_0124609  0.08  0.38  0.18  0.14  0.32  0.26 
Ca21Chr1_0124668  0.80  0.02  0.99 -0.08  0.01  0.32 
Ca21Chr1_0124727  0.63 -0.06  0.43 -0.01  0.33  0.00 
Ca21Chr1_0124786  0.34  0.45  0.30  0.55  0.24  0.06 
Ca21Chr1_0124845  0.45  0.16  0.24 -0.14  0.06  0.25 
Ca21Chr1_0124904  0.51  0.11  0.23 -0.28  0.10  0.19 
Ca21Chr1_0124963  0.41  0.26  0.35 -0.10 -0.64  0.53 
Ca21Chr1_0125022  0.57  0.44  0.47  0.35 -0.08  0.04 
Ca21Chr1_0125081  0.66  0.15  0.28 -0.05  0.04  0.00 
Ca21Chr1_0125140  0.29 -0.30  0.43 -0.47  0.08  0.53 
Ca21Chr1_0125199  0.17  0.08  0.11  0.11 -0.12  0.85 
Ca21Chr1_0125258 -0.45 -0.00  0.12  0.06 -0.16  0.55 
Ca21Chr1_0125317  0.11  0.08 -0.06  0.20 -0.12 -0.05 
Ca21Chr1_0125376  0.11  0.13 -0.09  0.18 -0.08  0.21 
Ca21Chr1_0125435  0.09  0.07  0.25 -0.10 -0.24  0.20 
Ca21Chr1_0125494  0.32  0.22  0.33  0.42 -0.13 -0.13 
Ca21Chr1_0125553  0.83  0.38  0.07  0.24 -0.19 -0.03  <orf19.6034
Ca21Chr1_0125612  0.42 -0.12 -0.77  0.03  0.40  0.45 
Ca21Chr1_0125671  0.96  0.09 -0.44 -0.11 -0.12  0.31 
Ca21Chr1_0125730  0.02 -0.48 -0.46 -0.30 -0.29 -0.46 
Ca21Chr1_0125789 -0.43  0.34  0.71  0.01  0.04 -0.20 
Ca21Chr1_0125848 -0.42  0.14 -0.16 -0.11  0.48  0.52 
Ca21Chr1_0125907 -0.04 -0.16 -0.44 -0.22 -0.18  0.43 
Ca21Chr1_0125966  0.07 -0.19 -0.16 -0.07 -0.24  0.33 
Ca21Chr1_0126025  0.09 -0.43 -0.02 -0.21 -0.27  0.48 
Ca21Chr1_0126084 -0.36  0.59 -0.37 -0.11 -0.16  0.25 
Ca21Chr1_0126143 -0.09 -0.01 -0.51 -0.41  0.05 -0.06 
Ca21Chr1_0126202 -1.05 -0.32 -0.43 -0.66 -0.58  0.04 
Ca21Chr1_0126261  0.05 -0.28 -0.70 -0.14 -0.37  0.26 
Ca21Chr1_0126320 -1.67 -0.68 -1.47 -0.85 -0.41 -0.40 
Ca21Chr1_0126379 -1.92 -1.33 -1.47 -1.54 -0.61 -0.03 
Ca21Chr1_0126438 -1.58 -1.17 -1.04 -1.36 -1.04 -0.19 
Ca21Chr1_0126497 -1.36 -1.19 -2.13 -0.41 -0.75  0.05  tP(UGG)3>
Ca21Chr1_0126556 -0.77 -0.95 -1.35 -1.48 -0.68  0.00  tP(UGG)3|
Ca21Chr1_0126615 -1.00 -1.46 -1.79 -0.76 -0.57 -0.53 
Ca21Chr1_0126674 -0.11 -1.49 -0.49 -0.73 -0.16  0.12 
Ca21Chr1_0126733 -0.05 -0.21  0.35  0.01  0.18 -1.17  |orf19.6033
Ca21Chr1_0126792  0.05  0.27  0.50  0.21  0.44 -0.27 
Ca21Chr1_0126851  0.03  0.56 -0.13 -0.02  0.26  0.14 
Ca21Chr1_0126910  0.18  0.06  0.28 -0.13  0.14  0.26 
Ca21Chr1_0126969  0.12  0.12  0.33  0.08  0.36  0.58 
Ca21Chr1_0127028  0.32  0.13  0.33 -0.07  0.07  0.24 
Ca21Chr1_0127087  0.38  0.26 -0.30 -0.17  0.32 -0.05 
Ca21Chr1_0127146  0.08  0.02  0.25 -0.01  0.14 -0.25 
Ca21Chr1_0127205  0.25  0.24  0.25 -0.17 -0.17  0.10 
Ca21Chr1_0127264  0.47  0.23  0.18  0.72  0.03  0.06 
Ca21Chr1_0127323  0.46  0.05  0.10  0.04  0.41 -0.39 
Ca21Chr1_0127382  0.37 -0.07  0.13  0.07  0.03 -0.04 
Ca21Chr1_0127441  0.26  0.27  0.30  0.35  0.17 -0.19 
Ca21Chr1_0127500 -0.61  0.12  0.18 -0.09 -0.44  0.51 
Ca21Chr1_0127559  0.18  0.11  0.37  0.04  0.13 -0.32 
Ca21Chr1_0127618  0.25 -0.04  0.40 -0.01  0.14 -0.51 
Ca21Chr1_0127677  0.10  0.23  0.54  0.12  0.06 -0.35 
Ca21Chr1_0127736  0.13  0.20  0.28 -0.36  0.14 -0.47 
Ca21Chr1_0127795  0.52  0.15  0.33 -0.05  0.26 -0.23 
Ca21Chr1_0127854 -0.24 -0.24  0.04  0.20 -0.29 -0.94 
Ca21Chr1_0127913 -0.16  0.07  0.25  0.03  0.36 -0.22 
Ca21Chr1_0127972 -0.23  0.00  0.18  0.33 -0.06 -0.19 
Ca21Chr1_0128031 -0.30 -0.12  0.64  0.33  0.40 -0.35 
Ca21Chr1_0128090 -0.20 -0.27  0.16  0.40 -0.12  0.25 
Ca21Chr1_0128149  0.00  0.31 -0.06  0.15 -0.04  0.37 
Ca21Chr1_0128208 -0.25  0.18  0.23 -0.39 -0.00  0.34 
Ca21Chr1_0128267  0.09  0.13 -0.18 -0.12 -0.22  0.26 
Ca21Chr1_0128326  0.04  0.22  0.33 -0.04 -0.02  0.08 
Ca21Chr1_0128385  0.87  0.21  0.29  0.11 -0.21  0.16 
Ca21Chr1_0128444  0.38  0.08 -0.03  0.28  0.14 -0.09 
Ca21Chr1_0128503  0.22  0.20  0.09  0.00 -0.03  0.23 
Ca21Chr1_0128562  0.28 -0.06  0.31 -0.16  0.11 -0.28  <orf19.6033
Ca21Chr1_0128621 -0.05  0.10 -0.10  0.06 -0.04 -0.12 
Ca21Chr1_0128680  0.01  0.16  0.35  0.20 -0.58  0.02 
Ca21Chr1_0128739  0.11  0.36 -0.48  0.09 -0.29 -0.29 
Ca21Chr1_0128798  0.29 -0.26 -0.09  0.59  0.13  0.47 
Ca21Chr1_0128857  0.14  0.13 -0.59 -0.25  0.19 -0.13 
Ca21Chr1_0128916  0.64 -0.23 -0.40  0.18  0.12 -0.12 
Ca21Chr1_0128975 -0.63  0.01 -0.29  0.25 -0.45 -0.10 
Ca21Chr1_0129034  0.61 -0.24 -0.31 -0.09 -0.17  0.76 
Ca21Chr1_0129093  0.15 -0.10 -0.06 -0.35  0.86  0.88 
Ca21Chr1_0129152  0.02 -0.21 -0.31 -0.06 -0.15 -0.28 
Ca21Chr1_0129211  0.87 -0.31  0.36  0.37  0.56 -0.17  |orf19.6032
Ca21Chr1_0129270  1.16  0.02  0.12 -0.02 -0.06  0.03 
Ca21Chr1_0129329  0.00  0.08 -0.21  0.10 -0.25  0.56 
Ca21Chr1_0129388  0.93  0.22 -0.01  0.09 -0.20 -0.31 
Ca21Chr1_0129447  0.00 -0.21  0.21  0.18 -0.13 -0.24 
Ca21Chr1_0129506  0.50 -0.30  0.33 -0.26  0.31 -0.80 
Ca21Chr1_0129565 -0.20 -0.34  0.24 -0.06 -0.30 -0.58 
Ca21Chr1_0129624  0.27 -0.16  0.11  0.21 -0.02  0.05 
Ca21Chr1_0129683  0.20 -0.04  0.06  0.27  0.01  0.11 
Ca21Chr1_0129742  0.22 -0.17 -0.10 -0.08 -0.04  0.01 
Ca21Chr1_0129801 -0.15  0.07  0.04  0.37  0.01 -0.10 
Ca21Chr1_0129860  0.36  0.38  0.08 -0.08  0.10 -0.29 
Ca21Chr1_0129919  0.84  0.25 -0.02  0.14 -0.04 -0.35 
Ca21Chr1_0129978  0.60  0.22  0.03  0.05  0.00 -0.22 
Ca21Chr1_0130037  0.13  0.58 -0.07 -0.00  0.02 -0.25 
Ca21Chr1_0130096  0.30 -0.02  0.01  0.10 -0.03 -0.12 
Ca21Chr1_0130155  0.33  0.09  0.17  0.19 -0.23  0.19 
Ca21Chr1_0130214  0.19  0.34  0.04  0.27 -0.03  0.11 
Ca21Chr1_0130273  1.30  0.35 -0.07  0.12  0.01 -0.60 
Ca21Chr1_0130332 -0.13 -0.05 -0.25  0.24  0.07 -0.42 
Ca21Chr1_0130391  0.13  0.10  0.12  0.12 -0.09 -0.42 
Ca21Chr1_0130450  0.65  0.31  0.33  0.01  0.19  0.04 
Ca21Chr1_0130509  0.10  0.33  0.42  0.13  0.15  0.14 
Ca21Chr1_0130568  0.67  0.63  0.13 -0.17 -0.07  0.40 
Ca21Chr1_0130627  0.31  0.01 -0.00 -0.02  0.14 -0.32  <orf19.6032
Ca21Chr1_0130686 -0.35  0.15  0.57  0.11  0.04  0.34 
Ca21Chr1_0130745  0.34 -0.00  0.05  0.06 -0.05  0.00 
Ca21Chr1_0130804  0.90  0.46  0.27  0.37 -0.23  0.37 
Ca21Chr1_0130863  0.22 -0.08 -0.15 -0.12  0.19 -1.18 
Ca21Chr1_0130922  0.78 -0.02 -0.26 -0.20 -0.22  0.21 
Ca21Chr1_0130981  0.10  0.14 -0.89 -0.03 -0.08  0.10 
Ca21Chr1_0131040 -0.12  0.07 -0.15  0.15  0.11 -0.61 
Ca21Chr1_0131099 -0.05  0.02 -0.03 -0.05 -0.20  0.55 
Ca21Chr1_0131158  0.29 -0.27 -0.21 -0.59 -0.37  2.40 
Ca21Chr1_0131217 -0.44 -0.34 -0.25  0.02 -0.07  0.75 
Ca21Chr1_0131276  0.34  0.03 -0.04  0.01 -0.10  0.35 
Ca21Chr1_0131335  0.03  0.29 -0.07  0.04 -0.08  0.46 
Ca21Chr1_0131394  0.29 -0.01 -0.16 -0.10  0.01  0.09  |orf19.6031
Ca21Chr1_0131453  0.37  0.18 -0.03  0.21 -0.07  0.56 
Ca21Chr1_0131512 -0.04  0.22  0.01  0.02  0.01  0.53 
Ca21Chr1_0131571  0.23 -0.01  0.40 -0.20 -0.25  0.34 
Ca21Chr1_0131630 -0.26  0.12  0.12 -0.18 -0.04 -0.08 
Ca21Chr1_0131689  0.12  0.14 -0.31  0.07 -0.26 -0.11 
Ca21Chr1_0131748  0.03 -0.03 -0.14 -0.24  0.11  0.03 
Ca21Chr1_0131807 -0.33  0.21 -0.30  0.32  0.27 -0.09 
Ca21Chr1_0131866 -0.06  0.03  0.22 -0.03  0.12  0.19 
Ca21Chr1_0131925 -0.71  0.33 -0.18  0.13  0.15  0.00 
Ca21Chr1_0131984  0.14 -0.11 -0.26  0.15 -0.06 -0.03 
Ca21Chr1_0132043 -0.22 -0.27 -0.47 -0.02  0.06  0.00 
Ca21Chr1_0132102  0.37  0.21  0.02  0.14 -0.02 -0.65 
Ca21Chr1_0132161  0.04 -0.04 -0.02 -0.02  0.12 -0.06 
Ca21Chr1_0132220 -0.29  0.37 -0.11  0.04  0.18 -0.52 
Ca21Chr1_0132279  0.01 -0.13 -0.10 -0.14 -0.19  0.08 
Ca21Chr1_0132338  0.44  0.08  0.24 -0.19  0.40  0.19 
Ca21Chr1_0132397 -0.53 -0.59  0.32 -0.16  0.05 -0.17 
Ca21Chr1_0132456  0.29  0.06  0.13  0.31 -0.10 -0.56 
Ca21Chr1_0132515  0.50  0.42  0.15 -0.32  0.23 -0.53 
Ca21Chr1_0132574 -0.15  0.45  0.22  0.21  0.50  0.22 
Ca21Chr1_0132633  0.18  0.08  0.13 -0.39  0.25 -0.28 
Ca21Chr1_0132692  0.22  0.26  0.43  0.38 -0.08 -0.48 
Ca21Chr1_0132751  0.23  0.06  0.55  0.02 -0.17 -0.08 
Ca21Chr1_0132810  0.30 -0.10  0.46 -0.01  0.07 -0.11 
Ca21Chr1_0132869  0.31  0.13  0.48  0.10  0.26 -0.26 
Ca21Chr1_0132928  0.34  0.47  0.41  0.44 -0.04 -0.13 
Ca21Chr1_0132987  0.35  0.29  0.22  0.12  0.07  0.07 
Ca21Chr1_0133046  0.02  0.06  0.14  0.16 -0.61 -0.03 
Ca21Chr1_0133105 -0.79  0.06  0.37 -0.11 -0.38 -0.23 
Ca21Chr1_0133164  0.25  0.29  0.23  0.05 -0.16  0.21 
Ca21Chr1_0133223  0.01  0.27 -0.02  0.08  0.13 -0.19 
Ca21Chr1_0133282  0.47  1.00  0.24  0.26 -0.23  0.31 
Ca21Chr1_0133341 -0.18  0.48  0.40 -0.12 -0.55 -0.31 
Ca21Chr1_0133400 -0.17  0.46  0.17 -0.08  0.03  0.47 
Ca21Chr1_0133459  0.05 -0.23  0.44  0.22 -0.28  0.83 
Ca21Chr1_0133518  0.01 -0.25 -0.87  0.01 -0.14 -0.15 
Ca21Chr1_0133577 -0.64 -0.01 -0.81 -0.08 -0.25 -1.12 
Ca21Chr1_0133636  0.12  0.28 -1.17  0.06 -0.39 -0.42 
Ca21Chr1_0133695  0.09  0.21 -1.11  0.49 -0.10 -0.13 
Ca21Chr1_0133754 -0.39 -0.32 -1.71 -0.29 -0.11 -0.40 
Ca21Chr1_0133813  0.03 -0.29 -1.49 -0.17 -0.02 -0.38 
Ca21Chr1_0133872 -0.49 -0.54 -1.40 -0.02 -0.29  0.09 
Ca21Chr1_0133931 -0.76 -0.32 -1.60 -0.10  0.05 -0.00  <orf19.6031
Ca21Chr1_0133990 -1.12 -0.60 -1.73 -0.47 -1.15 -0.04 
Ca21Chr1_0134049 -0.86 -0.80 -1.55 -0.31  0.11 -0.12 
Ca21Chr1_0134108 -0.15 -0.24 -0.38  0.28  0.46  0.51 
Ca21Chr1_0134167 -0.12 -0.20 -0.88 -0.15  0.08 -0.77 
Ca21Chr1_0134226  0.03 -0.61 -1.35  0.09 -0.20  0.09  orf19.6030>
Ca21Chr1_0134285 -0.24  0.02 -0.40  0.25 -0.09  0.21 
Ca21Chr1_0134344 -0.08 -0.14 -0.43 -0.44 -0.20 -0.88 
Ca21Chr1_0134403  0.66  0.21 -0.08  0.27  0.26  0.57 
Ca21Chr1_0134462  0.28 -0.16  0.04 -0.33  0.17 -0.24 
Ca21Chr1_0134521  0.02  0.16 -0.17  0.20  0.01 -0.33 
Ca21Chr1_0134580  0.34  0.07  0.01 -0.09 -0.21 -0.17 
Ca21Chr1_0134639 -0.06  0.20  0.33 -0.06  0.01  0.11 
Ca21Chr1_0134698 -0.10  0.00  0.17 -0.49  0.32 -0.05 
Ca21Chr1_0134757  0.15  0.30  0.25  0.11 -0.19 -0.10 
Ca21Chr1_0134816 -0.11  0.40  0.15 -0.18 -0.19  0.05 
Ca21Chr1_0134875 -0.91  0.09 -0.53 -0.00 -0.16  0.18  orf19.6030|
Ca21Chr1_0134934 -0.17  0.16 -0.16  0.17 -0.12 -0.29 
Ca21Chr1_0134993 -0.11  0.07 -0.65  0.04 -0.23 -0.31 
Ca21Chr1_0135052 -0.62 -0.07 -0.46  0.17 -0.33 -0.19 
Ca21Chr1_0135111 -0.78 -0.21 -0.44 -0.01 -0.23 -1.01 
Ca21Chr1_0135170 -0.46  0.27  0.18 -0.03 -0.49 -0.66 
Ca21Chr1_0135229 -0.22 -0.22  0.10  0.04 -0.08 -0.46 
Ca21Chr1_0135288  1.81 -0.16  0.33  0.18  0.38 -0.56 
Ca21Chr1_0135347  0.80 -0.12  0.06  0.04 -0.57 -0.64 
Ca21Chr1_0135406    NA    NA    NA    NA    NA    NA
Ca21Chr1_0135465  1.50  0.08  0.08  0.26  0.33 -0.75 
Ca21Chr1_0135524  0.03  0.21 -0.32 -0.19  0.19 -0.53 
Ca21Chr1_0135583  0.34  0.12  0.04  0.24  0.18  0.49 
Ca21Chr1_0135642  0.12 -0.18  0.19 -0.78 -0.15 -0.08  |orf19.6029
Ca21Chr1_0135701  0.77  0.15  0.45  0.26  0.19 -0.09 
Ca21Chr1_0135760  0.28  0.12  0.51  0.64  0.07  0.16 
Ca21Chr1_0135819  0.36  0.26  0.25  0.29  0.10 -0.04 
Ca21Chr1_0135878  0.15  0.27  0.31  0.05 -0.21 -0.06 
Ca21Chr1_0135937  0.05  0.14  0.02  0.13  0.17 -0.25 
Ca21Chr1_0135996  0.11 -0.03  0.26  0.30 -0.22 -0.15 
Ca21Chr1_0136055  0.00  0.09  0.32  0.32 -0.14  0.51 
Ca21Chr1_0136114  0.30  0.18  0.38  0.86 -0.80  0.38 
Ca21Chr1_0136173  0.12  0.10  0.23 -0.19 -0.29  0.17 
Ca21Chr1_0136232  0.09  0.21  0.32  0.33  0.11  0.22 
Ca21Chr1_0136291  0.49  0.07  0.34  0.07  0.39  0.41 
Ca21Chr1_0136350 -0.93 -0.14  0.00  0.72 -0.49 -0.14 
Ca21Chr1_0136409 -0.27 -0.13  0.22  0.31 -0.14  0.08  <orf19.6029
Ca21Chr1_0136468  0.55 -0.03  0.11  0.09 -0.28  0.42 
Ca21Chr1_0136527  0.52 -0.67 -0.06  0.12  0.25 -0.36 
Ca21Chr1_0136586 -0.39  0.15 -0.06  0.35  0.13 -0.13 
Ca21Chr1_0136645  0.40  0.27  0.18 -0.33  0.24  0.48 
Ca21Chr1_0136704  0.25 -0.24 -0.33  0.31  0.26  0.44 
Ca21Chr1_0136763 -0.09  0.17 -0.20 -0.10 -0.06  0.48 
Ca21Chr1_0136822 -0.64  0.28 -0.25 -0.15  0.04  0.14 
Ca21Chr1_0136881  0.22 -0.12 -0.48  0.03  0.09  0.37 
Ca21Chr1_0136940    NA    NA    NA    NA    NA    NA
Ca21Chr1_0136999 -0.28  0.01 -0.93 -0.24 -0.11  0.78 
Ca21Chr1_0137058 -0.06 -0.65 -0.35 -0.03  0.26 -0.44 
Ca21Chr1_0137117 -0.17 -0.08 -0.63 -0.21 -0.16 -0.30 
Ca21Chr1_0137176  0.39 -0.25 -0.06 -0.10 -0.30  0.24 
Ca21Chr1_0137235  0.04 -0.32  0.14  0.10 -0.23  0.14 
Ca21Chr1_0137294 -0.22 -0.21 -0.31  0.10 -0.26 -0.20 
Ca21Chr1_0137353 -0.04 -0.21 -0.16  0.46  0.02  0.01 
Ca21Chr1_0137412 -0.23  0.28 -0.07  0.40  0.25  0.10 
Ca21Chr1_0137471 -0.01  0.07  0.19 -0.02  0.13 -0.35 
Ca21Chr1_0137530  0.10  0.27  0.02  0.09  0.00 -0.65 
Ca21Chr1_0137589  0.10  0.28 -0.35  0.31  0.29 -0.26 
Ca21Chr1_0137648 -0.16  0.33 -0.32  0.04  0.12  0.08 
Ca21Chr1_0137707 -0.09 -0.06  0.31  0.02  0.27 -1.07 
Ca21Chr1_0137766  0.35 -0.12 -0.03  0.04 -0.25  0.05 
Ca21Chr1_0137825 -0.17  0.34  0.12  0.28 -0.11 -0.60 
Ca21Chr1_0137884 -0.19  0.57 -0.06 -0.09 -0.28 -1.29 
Ca21Chr1_0137943  0.08  0.35 -0.33 -0.32 -0.07 -0.62 
Ca21Chr1_0138002  0.13  0.13  0.43  0.21  0.15 -0.80 
Ca21Chr1_0138061 -0.87  0.06 -0.11  0.04  0.00 -0.94 
Ca21Chr1_0138120    NA    NA    NA    NA    NA    NA
Ca21Chr1_0138179 -0.75 -0.11 -0.22  0.05  0.19  0.26 
Ca21Chr1_0138238 -0.89  0.01  0.04  0.17  1.33 -0.63 
Ca21Chr1_0138297    NA    NA    NA    NA    NA    NA
Ca21Chr1_0138356 -2.04 -0.42  0.46  0.11 -0.16 -0.30 
Ca21Chr1_0138415 -1.64  0.47 -0.71  0.13 -0.70  0.23 
Ca21Chr1_0138474 -0.43  0.09  0.03  0.07 -0.29 -0.34 
Ca21Chr1_0138533 -1.25  0.24  0.34  0.29 -0.29  0.09  |orf19.6028
Ca21Chr1_0138592 -1.35  0.13  0.19  0.06  0.02  0.93 
Ca21Chr1_0138651 -0.91  0.59 -0.07 -0.00 -0.03  0.82 
Ca21Chr1_0138710 -0.35 -0.08  0.22  0.05 -0.18  0.60 
Ca21Chr1_0138769 -0.10  0.35  0.65  0.26 -0.07  0.07 
Ca21Chr1_0138828 -0.13 -0.00 -0.00 -0.19 -0.33  0.24 
Ca21Chr1_0138887  0.20  0.43  0.36  0.20  0.22  0.28 
Ca21Chr1_0138946  0.63  0.19  0.32 -0.03  0.18  0.29 
Ca21Chr1_0139005 -0.76  0.65 -0.76  0.07 -0.43  0.45 
Ca21Chr1_0139064  0.45  0.02  0.16  0.15  0.03  0.17 
Ca21Chr1_0139123 -0.25 -0.06 -0.03  0.16  0.23  0.14 
Ca21Chr1_0139182 -0.57  0.42 -0.38  0.14  0.22  0.04 
Ca21Chr1_0139241 -0.53  0.30 -0.28 -0.08  0.10  0.13 
Ca21Chr1_0139300 -0.92  0.20 -0.13 -0.32  0.13 -0.09 
Ca21Chr1_0139359 -0.60 -0.31 -0.46 -0.19 -0.11  1.09 
Ca21Chr1_0139418 -1.55 -0.65 -0.73 -0.25  0.19  0.38 
Ca21Chr1_0139477 -0.70 -0.33 -0.11 -0.35  0.11 -0.13 
Ca21Chr1_0139536 -0.43 -0.22 -0.05 -0.29 -0.21 -0.56 
Ca21Chr1_0139595 -0.20  0.08  0.07 -0.44 -0.03 -0.32 
Ca21Chr1_0139654 -0.06  0.35 -0.21  0.56  0.16  0.07 
Ca21Chr1_0139713 -0.08  0.08 -0.09 -0.48 -0.17 -0.51 
Ca21Chr1_0139772 -1.07  0.22 -0.06 -0.04 -0.21 -0.49 
Ca21Chr1_0139831 -0.54  0.31  0.26  0.24  0.29  0.15 
Ca21Chr1_0139890 -0.74  0.05  0.21 -0.30 -0.28  1.61 
Ca21Chr1_0139949 -0.00  0.41  0.60  0.27 -0.38  0.25 
Ca21Chr1_0140008 -0.31  0.40  0.11 -0.04 -0.23  0.44 
Ca21Chr1_0140067  0.41  0.23  0.22 -0.02 -0.27  0.40 
Ca21Chr1_0140126 -0.28  0.32  0.29  0.11 -0.03  0.00 
Ca21Chr1_0140185 -0.19 -0.01  0.18  0.36 -0.23  0.25 
Ca21Chr1_0140244 -0.30  0.52  0.32  0.17 -0.41  0.74 
Ca21Chr1_0140303 -0.19  0.28  0.21  0.19 -0.60  0.41 
Ca21Chr1_0140362 -0.65  0.27  0.09  0.20  0.17  2.36 
Ca21Chr1_0140421  0.21  0.10  0.18 -0.07 -0.27  0.13 
Ca21Chr1_0140480 -0.67  0.00 -0.10  0.08  0.76 -0.77 
Ca21Chr1_0140539 -0.23 -0.05 -0.25  0.32  0.15  0.33 
Ca21Chr1_0140598  0.12  0.07 -0.56  0.29 -0.33  0.37 
Ca21Chr1_0140657  0.01  0.15  0.40 -0.03  0.27  0.64 
Ca21Chr1_0140716 -0.44  0.43  0.30  0.34  0.07  0.35 
Ca21Chr1_0140775 -0.27  0.26 -0.30 -0.24  0.14  0.24 
Ca21Chr1_0140834  0.49  0.35 -0.07  0.10  0.19 -0.02 
Ca21Chr1_0140893 -0.46 -0.03  0.71  0.13  0.34  0.26  <orf19.6028
Ca21Chr1_0140952 -0.26  0.13 -0.21  0.17 -0.38 -0.27 
Ca21Chr1_0141011 -0.30  0.32  0.31  0.40 -0.05  0.56 
Ca21Chr1_0141070 -0.27  0.34  0.39  0.17 -0.22 -0.08 
Ca21Chr1_0141129 -0.23  0.08  0.22 -0.02 -0.47 -0.00 
Ca21Chr1_0141188  0.08  0.11 -0.44 -0.06 -0.25  0.21 
Ca21Chr1_0141247 -0.78  0.12 -0.06 -0.20  0.02 -0.24 
Ca21Chr1_0141306 -0.40 -0.03 -0.06 -0.00 -0.10 -0.20 
Ca21Chr1_0141365 -0.63  0.50 -0.89  0.15  0.01  0.10 
Ca21Chr1_0141424 -0.60  0.10  0.07 -0.05  0.27 -0.18 
Ca21Chr1_0141483  0.17  0.37  0.37  0.19 -0.14  0.06 
Ca21Chr1_0141542 -0.31  0.17  0.32 -0.02 -0.18  0.20 
Ca21Chr1_0141601 -0.67 -0.10  0.56  0.16  0.36  0.10 
Ca21Chr1_0141660  1.03  0.36  0.12  0.19  0.19 -0.07 
Ca21Chr1_0141719  0.59  0.30 -1.08 -0.01 -0.01 -0.74 
Ca21Chr1_0141778 -0.38  0.09 -0.30  0.46  0.01 -1.34 
Ca21Chr1_0141837  0.28  0.00 -0.38  0.14  0.33 -0.46 
Ca21Chr1_0141896  0.20 -0.01 -0.29  0.07  0.45  0.26 
Ca21Chr1_0141955 -0.89  0.12 -0.02 -0.07  0.20 -0.32 
Ca21Chr1_0142014 -0.68  0.20 -0.51 -0.37  0.06 -0.85 
Ca21Chr1_0142073 -0.54  0.23 -0.08 -0.37 -0.16 -0.52 
Ca21Chr1_0142132 -0.42 -0.10 -0.01  0.16  0.48 -0.27 
Ca21Chr1_0142191  0.12 -0.09 -0.26 -0.01  0.14 -0.32 
Ca21Chr1_0142250 -0.74 -0.03 -1.07  0.20  0.25 -0.38 
Ca21Chr1_0142309  1.15 -0.25 -0.46  0.08 -0.26 -0.61 
Ca21Chr1_0142368 -0.35  0.03  0.19 -0.31  0.94 -0.23 
Ca21Chr1_0142427  0.09 -0.03 -0.14  0.18  0.33 -0.95 
Ca21Chr1_0142486    NA    NA    NA    NA    NA    NA
Ca21Chr1_0142545  0.13  0.02 -0.25 -0.01  0.38 -0.17 
Ca21Chr1_0142604 -0.23  0.05 -0.67 -0.15  1.01 -0.22 
Ca21Chr1_0142663 -0.53 -0.15 -1.37 -0.00 -0.11  0.20 
Ca21Chr1_0142722    NA    NA    NA    NA    NA    NA
Ca21Chr1_0142781 -0.61 -1.03 -0.81 -0.15 -0.06  0.50 
Ca21Chr1_0142840 -0.49 -0.76 -1.23 -0.47 -0.14  0.81 
Ca21Chr1_0142899 -0.32 -0.40 -0.43  0.24 -0.12  0.70 
Ca21Chr1_0142958 -0.23  0.02 -0.67  0.03 -0.37  1.52 
Ca21Chr1_0143017 -0.33 -0.20 -0.53 -0.36  0.12  1.38 
Ca21Chr1_0143076  0.03 -0.25 -0.78 -0.00 -0.23  1.35 
Ca21Chr1_0143135 -0.15 -0.21 -0.32  0.22  0.14  0.53 
Ca21Chr1_0143194  0.02 -0.15 -0.65 -0.32 -0.28  0.26 
Ca21Chr1_0143253 -0.79 -0.02 -0.02  0.39  0.85 -0.08 
Ca21Chr1_0143312 -0.13 -0.10 -0.15  0.09 -0.21  0.02 
Ca21Chr1_0143371  0.02  0.25  0.18  0.10  0.10  0.13 
Ca21Chr1_0143430  0.32  0.55  0.25  0.25 -0.08  0.07 
Ca21Chr1_0143489  0.21  0.57  0.37 -0.25 -0.28 -0.15 
Ca21Chr1_0143548  0.14  0.28  0.27 -0.21  0.01 -0.10 
Ca21Chr1_0143607  0.06 -0.02  0.28  0.18 -0.16 -0.52 
Ca21Chr1_0143666  0.41  0.01  0.20  0.09  0.06  0.24 
Ca21Chr1_0143725  0.03 -0.21 -0.06  0.01 -0.22  0.15 
Ca21Chr1_0143784 -0.14  0.10  0.18  0.25 -0.11  0.48 
Ca21Chr1_0143843  0.13  0.14  0.25  0.15 -0.18  0.39 
Ca21Chr1_0143902  0.02  0.26  0.03 -0.16 -0.08 -0.16 
Ca21Chr1_0143961 -0.38 -0.12  0.07 -0.09  0.14 -0.62 
Ca21Chr1_0144020 -0.08 -0.06 -0.04 -0.05 -0.27 -0.69 
Ca21Chr1_0144079  0.14  0.20  0.20  0.16 -0.14 -0.32 
Ca21Chr1_0144138  0.35  0.09  0.19  0.21  0.03  0.80 
Ca21Chr1_0144197 -0.14  0.47 -0.15  0.54  0.55  0.51 
Ca21Chr1_0144256  0.19  0.25  0.26 -0.11 -0.42 -0.49 
Ca21Chr1_0144315  0.12 -0.50  0.24  0.09 -0.21 -0.08 
Ca21Chr1_0144374  0.26  0.03 -0.11  0.54  0.01  0.01 
Ca21Chr1_0144433  0.38  0.38  0.31  0.17  0.86  0.23 
Ca21Chr1_0144492 -0.06 -0.04  0.03  0.51  0.03 -1.03 
Ca21Chr1_0144551 -0.33  0.31  0.15  0.04  0.14 -0.47 
Ca21Chr1_0144610 -0.07  0.06  0.23 -0.03  0.27 -0.06 
Ca21Chr1_0144669 -0.02  0.32  0.20  0.28 -0.32 -0.05 
Ca21Chr1_0144728 -0.24  0.53 -0.18  0.08 -0.37  0.26 
Ca21Chr1_0144787  0.28  0.26 -0.28  0.13 -0.26 -0.05 
Ca21Chr1_0144846 -0.10  0.22  0.09  0.03 -0.37 -0.20 
Ca21Chr1_0144905  0.21  0.77 -0.02  0.17 -0.42 -0.09 
Ca21Chr1_0144964  0.31  0.28  0.35 -0.14 -0.66 -0.36 
Ca21Chr1_0145023  0.27  0.09 -0.12 -0.23 -0.19 -0.58 
Ca21Chr1_0145082 -0.17  0.10 -0.43 -0.22 -0.04 -0.63 
Ca21Chr1_0145141 -0.27  0.32 -0.03  0.22 -0.34 -0.25 
Ca21Chr1_0145200  0.31  0.48  0.32  0.25  0.40 -0.68 
Ca21Chr1_0145259  2.01  0.11 -0.24 -0.12 -0.23 -0.37 
Ca21Chr1_0145318    NA    NA    NA    NA    NA    NA
Ca21Chr1_0145377 -0.16  0.52 -0.44  0.02  0.25  0.50 
Ca21Chr1_0145436  0.26 -0.09  0.85 -0.08 -0.17 -0.61 
Ca21Chr1_0145495  0.26 -0.19 -0.75 -0.03  0.88 -1.28 
Ca21Chr1_0145554    NA    NA    NA    NA    NA    NA
Ca21Chr1_0145613 -0.14 -0.04  0.05 -0.05  0.24 -0.11 
Ca21Chr1_0145672 -0.34  0.17 -0.45  0.11 -0.11  0.05 
Ca21Chr1_0145731  0.58 -0.01  0.01  0.43 -0.07  0.35 
Ca21Chr1_0145790 -0.25  0.04  0.11  0.00  0.85 -0.52 
Ca21Chr1_0145849  0.98  0.00 -0.56  0.05 -0.03 -0.32 
Ca21Chr1_0145908  0.11  0.42  0.69 -0.03  0.05 -0.19 
Ca21Chr1_0145967  1.49  0.27  0.11  0.16 -0.25 -0.59 
Ca21Chr1_0146026  0.08  0.40 -0.53  0.02 -0.13 -0.40 
Ca21Chr1_0146085  0.09  0.35  0.37  0.02 -0.36 -0.43 
Ca21Chr1_0146144 -0.40  0.53 -0.05  0.06  0.11 -0.05 
Ca21Chr1_0146203 -0.13  0.03 -0.54  0.27 -0.44 -0.90 
Ca21Chr1_0146262  0.07 -0.07 -0.40 -0.04 -0.40  1.05 
Ca21Chr1_0146321  0.32 -0.28 -0.95 -0.03  0.26 -0.36 
Ca21Chr1_0146380 -0.69  0.25  0.53  1.21 -0.31  0.65 
Ca21Chr1_0146439 -0.26  0.21 -0.02  0.37  0.31  0.33 
Ca21Chr1_0146498  0.09  0.17  0.65 -0.22  0.20  0.39 
Ca21Chr1_0146557  0.30 -0.07 -0.34 -0.09  0.02  0.67 
Ca21Chr1_0146616 -0.33 -0.03  0.05  0.20 -0.02  0.17 
Ca21Chr1_0146675  0.01  0.23 -0.29  0.24 -0.41 -0.21 
Ca21Chr1_0146734    NA    NA    NA    NA    NA    NA
Ca21Chr1_0146793 -0.09  0.16 -0.36  0.22 -0.27 -0.27 
Ca21Chr1_0146852  0.35  0.01  0.10 -0.38  0.00  0.09 
Ca21Chr1_0146911  1.55  0.38 -0.29 -0.43 -0.51 -0.44 
Ca21Chr1_0146970  0.13  0.21 -0.15  0.47 -0.10 -0.56 
Ca21Chr1_0147029  1.67  0.43  0.77  0.02  0.51 -0.45 
Ca21Chr1_0147088  0.46  0.15 -0.06  0.04  0.34  0.03 
Ca21Chr1_0147147 -0.24  0.18 -0.08 -0.18 -0.27 -0.42 
Ca21Chr1_0147206 -0.31  0.19 -0.09  0.03 -0.49 -0.13 
Ca21Chr1_0147265 -0.46  0.08 -0.07 -0.12  0.00  0.12 
Ca21Chr1_0147324 -0.25  0.11 -0.36 -0.28 -0.26 -0.08 
Ca21Chr1_0147383 -0.17 -0.07 -0.31 -0.38  0.07  0.06 
Ca21Chr1_0147442  0.09  0.02 -0.22  0.34  0.67  0.17 
Ca21Chr1_0147501 -0.17  0.31 -0.07 -0.18  0.05 -0.13 
Ca21Chr1_0147560  0.13  0.41  0.43  0.15 -0.05  0.82 
Ca21Chr1_0147619 -0.40 -0.01 -0.06  0.00 -0.54  0.01 
Ca21Chr1_0147678 -0.42  0.07  0.16  0.07 -0.77  0.62 
Ca21Chr1_0147737  0.64  0.31  0.53  0.10  0.02 -0.24 
Ca21Chr1_0147796  0.32  0.33 -0.09  0.12 -0.42 -0.16 
Ca21Chr1_0147855  0.98  0.28  0.11  0.01  0.07 -0.93 
Ca21Chr1_0147914  0.39  0.70 -0.01  0.12 -0.11 -0.58 
Ca21Chr1_0147973    NA    NA    NA    NA    NA    NA
Ca21Chr1_0148032 -0.07  0.58 -0.18  0.06  0.26 -0.73 
Ca21Chr1_0148091 -0.04 -0.32 -0.70 -0.40  0.24 -0.16 
Ca21Chr1_0148150 -0.18 -0.00 -0.63 -0.09  0.05 -0.34 
Ca21Chr1_0148209 -0.03  0.31  0.16 -0.46  0.12 -0.45 
Ca21Chr1_0148268  0.08 -0.15 -0.62 -0.28  0.60 -0.65 
Ca21Chr1_0148327 -0.21  0.43  0.01 -0.40 -0.35 -0.17 
Ca21Chr1_0148386  0.13 -0.11 -0.71  0.11 -0.49 -0.31 
Ca21Chr1_0148445  0.50 -0.16 -0.81 -0.29  0.36 -0.63 
Ca21Chr1_0148504    NA    NA    NA    NA    NA    NA
Ca21Chr1_0148563  1.80 -0.33 -0.66 -0.19  0.16  0.89 
Ca21Chr1_0148622  0.13  0.03 -0.21  0.25 -0.30 -0.87 
Ca21Chr1_0148681 -0.31  0.66  0.56  0.02  0.08 -0.12 
Ca21Chr1_0148740  0.67 -0.14  0.38 -0.19  0.08 -0.84 
Ca21Chr1_0148799  1.83  0.26 -0.32  0.08 -0.19  0.56 
Ca21Chr1_0148858 -0.35  0.10  0.12 -0.62 -0.86  0.16 
Ca21Chr1_0148917 -0.71  0.04 -0.31  0.24 -0.41 -0.11 
Ca21Chr1_0148976 -0.72  0.16  0.18  0.32 -0.17  0.24 
Ca21Chr1_0149035    NA    NA    NA    NA    NA    NA
Ca21Chr1_0149094  0.55  0.00 -0.19 -0.00  0.05  0.01 
Ca21Chr1_0149153 -0.39  0.16 -0.51  0.15  0.42  0.28 
Ca21Chr1_0149212 -0.04 -0.85  0.23 -0.60  0.16 -1.19 
Ca21Chr1_0149271  0.01  0.12  0.47 -0.10 -0.40 -0.72 
Ca21Chr1_0149330 -0.33 -0.02 -0.17 -0.00  0.01  0.49 
Ca21Chr1_0149389 -0.36 -0.15 -0.30 -0.34 -0.13  0.05 
Ca21Chr1_0149448  0.12 -0.18  0.01  0.25  0.63 -0.47 
Ca21Chr1_0149507  0.03 -0.02 -0.22  0.05  0.68 -0.72 
Ca21Chr1_0149566 -0.05 -0.19  0.97 -0.31 -0.34 -0.73 
Ca21Chr1_0149625 -0.10  0.07  0.24  0.00  0.36 -0.17 
Ca21Chr1_0149684  0.76 -0.12 -0.32  0.41 -0.46 -0.44 
Ca21Chr1_0149743 -0.32  0.31 -0.14  0.35  1.19 -1.09 
Ca21Chr1_0149802 -0.45  0.18  0.02  0.49 -0.58 -0.12 
Ca21Chr1_0149861 -0.80  0.38 -0.23  0.18 -0.12  0.40 
Ca21Chr1_0149920 -0.64  0.20 -0.06  0.04  0.02  1.72 
Ca21Chr1_0149979 -0.27  0.55 -0.04  0.13 -0.02  0.77 
Ca21Chr1_0150038 -0.03  0.30 -0.08 -0.29 -0.06  0.73 
Ca21Chr1_0150097    NA    NA    NA    NA    NA    NA
Ca21Chr1_0150156 -0.39 -3.55 -0.31 -0.16 -0.38  0.71 
Ca21Chr1_0150215 -0.22  0.09  0.06 -0.05 -0.44  0.22 
Ca21Chr1_0150274  0.04  0.27 -0.09 -0.02 -0.14 -0.19 
Ca21Chr1_0150333  0.28 -0.00 -0.04  0.05 -0.35 -0.62 
Ca21Chr1_0150392  0.34  0.10 -0.23 -0.32 -0.24 -0.50 
Ca21Chr1_0150451 -0.43 -0.30 -0.35  0.78  0.14 -0.57 
Ca21Chr1_0150510 -0.31 -0.40  0.09  0.58 -0.04 -0.11 
Ca21Chr1_0150569  0.20 -0.15 -0.04 -0.06 -0.06  0.10  orf19.6027>
Ca21Chr1_0150628  2.06  0.22  0.17 -0.14 -0.45 -0.01 
Ca21Chr1_0150687 -0.13 -0.07 -0.02  0.04 -0.01 -0.12 
Ca21Chr1_0150746  0.24  0.38  0.05  0.01 -0.11 -0.09 
Ca21Chr1_0150805 -0.12  0.34  0.47  0.28  0.01 -0.04 
Ca21Chr1_0150864  0.14  0.33  0.42  0.11 -0.24 -0.11 
Ca21Chr1_0150923  0.16  0.40  0.92  0.03 -0.12  0.30 
Ca21Chr1_0150982  0.64  0.29  0.42  0.41 -0.22  0.08 
Ca21Chr1_0151041 -0.17  0.15  0.24  0.42  0.09  0.28 
Ca21Chr1_0151100 -0.03 -0.08  0.00  0.28  0.13  1.32 
Ca21Chr1_0151159 -0.47  0.32  0.01  0.60  0.13 -0.12 
Ca21Chr1_0151218  0.27  0.31 -0.34  0.14 -0.35  0.01 
Ca21Chr1_0151277 -0.12  0.01 -0.13  0.15 -0.05 -0.64 
Ca21Chr1_0151336  0.03 -0.12 -0.10 -0.42 -0.13 -0.39 
Ca21Chr1_0151395 -0.13 -0.04  0.04 -0.10 -0.33 -0.55 
Ca21Chr1_0151454 -0.21  0.17 -0.11 -0.02  0.33 -0.42 
Ca21Chr1_0151513 -0.15  0.23 -0.15  0.07 -0.14 -0.58 
Ca21Chr1_0151572  0.28  0.35  0.03 -0.17  0.02 -0.88 
Ca21Chr1_0151631  0.97  0.57 -0.57  0.45  0.33 -0.32 
Ca21Chr1_0151690  0.64 -0.72  0.05  0.30 -0.26  0.20  orf19.6027|
Ca21Chr1_0151749 -0.18  0.34  0.11 -0.11  0.01  0.47 
Ca21Chr1_0151808 -0.11 -0.20 -0.12  0.40  0.35 -0.34 
Ca21Chr1_0151867  0.04  0.29 -0.24 -0.45  0.94 -0.36 
Ca21Chr1_0151926  0.02 -0.04 -0.14  0.12 -0.14  0.52 
Ca21Chr1_0151985  0.41 -0.10  0.18 -0.40  0.03  0.04 
Ca21Chr1_0152044  0.02  0.10  0.38  0.13 -0.33 -0.09 
Ca21Chr1_0152103 -0.04  0.09 -0.03  0.03 -0.24  0.90 
Ca21Chr1_0152162 -0.38  0.31 -0.17 -0.19 -0.34  1.06 
Ca21Chr1_0152221  0.04  0.55  0.31 -0.02 -0.62  0.79 
Ca21Chr1_0152280 -0.42 -0.09  0.21 -0.15 -0.02  0.59 
Ca21Chr1_0152339  0.04  0.15  0.44  0.06 -0.85  0.51 
Ca21Chr1_0152398 -0.34 -0.36 -0.00 -0.21 -0.01 -0.43 
Ca21Chr1_0152457 -0.51  0.08  0.43 -0.27 -0.18  0.23 
Ca21Chr1_0152516 -0.90 -0.05  0.01 -0.45 -0.07  0.51 
Ca21Chr1_0152575 -0.49 -0.11  0.12  0.23 -0.26 -0.72 
Ca21Chr1_0152634 -0.17 -0.22 -0.10  0.30 -0.07 -0.14 
Ca21Chr1_0152693  0.84  0.16  0.19  0.04 -0.08  0.21 
Ca21Chr1_0152752 -0.17  0.10 -0.18 -0.02 -0.16  0.94 
Ca21Chr1_0152811 -0.32 -0.15 -0.17  0.46 -0.20  0.18 
Ca21Chr1_0152870 -0.35  0.30  0.19 -0.13  0.12  1.63 
Ca21Chr1_0152929 -0.24  0.48 -0.02  0.06  0.18  1.17 
Ca21Chr1_0152988 -0.07 -0.48  0.16 -0.21  0.06  0.24 
Ca21Chr1_0153047    NA    NA    NA    NA    NA    NA
Ca21Chr1_0153106 -0.52  0.27 -0.61 -0.19 -0.21 -0.25 
Ca21Chr1_0153165 -0.21  0.26 -0.39  0.34 -0.16 -0.28 
Ca21Chr1_0153224  0.07 -0.06 -0.46 -0.38 -0.13  0.31 
Ca21Chr1_0153283 -0.68 -0.39 -0.29  0.31  0.46 -0.43 
Ca21Chr1_0153342 -0.37  0.02 -0.59  0.05 -0.05 -0.38 
Ca21Chr1_0153401 -0.38 -0.04 -0.33  0.12  0.13 -0.55 
Ca21Chr1_0153460 -0.69  0.20 -0.66  0.10 -0.24 -0.62 
Ca21Chr1_0153519 -0.86  0.15 -0.55 -0.00  0.34 -0.38 
Ca21Chr1_0153578 -0.30 -0.07 -0.43  0.02 -0.03  0.03 
Ca21Chr1_0153637 -0.11 -0.40 -0.71 -0.14  0.20 -0.31 
Ca21Chr1_0153696 -0.30 -0.31 -0.99 -0.14 -0.01 -0.18 
Ca21Chr1_0153755 -0.18 -0.30 -0.70  0.18 -0.30 -0.26 
Ca21Chr1_0153814 -0.16 -0.20 -1.14  0.22 -0.41 -0.07 
Ca21Chr1_0153873 -0.39  0.76 -0.38  0.01 -0.22 -0.11 
Ca21Chr1_0153932 -0.23  0.40 -0.68  0.05 -0.18 -0.09 
Ca21Chr1_0153991 -0.06  0.02 -0.15  0.16 -0.06  0.55 
Ca21Chr1_0154050 -0.83  0.01 -0.16  0.40  0.12  0.72 
Ca21Chr1_0154109 -0.64  0.05 -0.49 -0.41 -0.13  0.55 
Ca21Chr1_0154168 -0.67 -0.05 -0.49 -0.26 -0.21  0.48 
Ca21Chr1_0154227 -0.79  0.06 -0.78  0.08 -0.71  0.92 
Ca21Chr1_0154286 -0.36  0.26 -0.96 -0.36 -0.24  0.18 
Ca21Chr1_0154345 -0.46 -0.19 -1.00 -0.44 -0.15 -0.07 
Ca21Chr1_0154404 -0.16  0.00 -0.83  0.12 -0.21 -0.24 
Ca21Chr1_0154463 -0.09 -0.34 -0.85 -0.53 -0.44  0.23 
Ca21Chr1_0154522 -0.01 -0.04 -0.44 -0.69 -0.04  0.23 
Ca21Chr1_0154581 -0.15 -0.05 -0.47 -0.26 -0.30  0.29  |orf19.6026
Ca21Chr1_0154640  0.32 -0.09 -0.31 -0.46 -0.54 -0.27 
Ca21Chr1_0154699 -0.28 -0.09  0.08 -0.28 -0.06 -0.01 
Ca21Chr1_0154758  0.24  0.27  0.09 -0.55  0.25 -0.21 
Ca21Chr1_0154817  1.09 -0.15  1.31 -0.47 -0.85 -0.06 
Ca21Chr1_0154876  0.22 -0.11  0.04 -0.25 -0.42 -0.56 
Ca21Chr1_0154935 -0.01 -0.16 -0.12 -0.52 -0.15  0.06 
Ca21Chr1_0154994 -0.07  0.11 -0.27 -0.12 -0.29 -0.01 
Ca21Chr1_0155053  0.22  0.08  0.18  0.16  0.12  1.58 
Ca21Chr1_0155112  0.01  0.23  0.30  0.26  0.81  0.12 
Ca21Chr1_0155171 -0.25 -0.05  0.17 -0.21 -0.19 -1.15 
Ca21Chr1_0155230  0.27  0.07  0.29  0.21 -0.13  0.02 
Ca21Chr1_0155289 -0.52  0.28  0.31  0.05  0.68 -0.15  <orf19.6026
Ca21Chr1_0155348 -0.19  0.03 -0.57  0.39  0.41  0.16 
Ca21Chr1_0155407    NA    NA    NA    NA    NA    NA orf19.6025>
Ca21Chr1_0155466  0.17 -0.35  0.39 -0.21 -0.29  0.54 
Ca21Chr1_0155525 -0.16  0.12 -0.02  0.24 -0.18  0.34 
Ca21Chr1_0155584 -0.41  0.02 -0.43  1.08 -0.34  0.10 
Ca21Chr1_0155643 -0.05  0.02  0.06 -0.21 -0.08  0.10 
Ca21Chr1_0155702  0.39  0.32 -0.01  0.18  0.25  0.27 
Ca21Chr1_0155761 -0.39 -0.44  0.85  0.16 -0.66  0.35 
Ca21Chr1_0155820 -0.40 -0.11  0.08  0.12 -0.43 -0.43 
Ca21Chr1_0155879  0.07  0.30 -0.32  0.69 -0.34  0.24 
Ca21Chr1_0155938  0.23 -0.11 -0.14 -0.15 -0.35 -0.44 
Ca21Chr1_0155997 -0.05  0.23 -0.18  0.19 -0.35  0.32 
Ca21Chr1_0156056  0.37  0.11  0.09  0.12 -0.32 -0.27 
Ca21Chr1_0156115 -0.15  0.04  0.02  0.13 -0.10 -0.09 
Ca21Chr1_0156174 -1.65  0.47 -0.47  0.18  0.15 -0.06 
Ca21Chr1_0156233 -0.88 -0.46 -1.12 -0.19 -0.38 -0.46 
Ca21Chr1_0156292 -1.39 -0.39 -0.84 -0.10 -0.07 -0.03  orf19.6025|
Ca21Chr1_0156351 -1.44 -0.23 -1.38  0.08  0.01 -0.14 
Ca21Chr1_0156410 -1.70 -0.54 -1.39  0.09 -0.30 -0.11 
Ca21Chr1_0156469 -1.62  0.00 -1.44 -0.25 -0.06  0.12 
Ca21Chr1_0156528 -1.21 -0.55 -1.23  0.56 -0.37  0.49 
Ca21Chr1_0156587 -0.68 -0.77 -0.48  0.11  0.21  0.13 
Ca21Chr1_0156646 -0.49 -0.34 -1.03 -0.27 -0.01  0.02  orf19.6024>
Ca21Chr1_0156705 -0.62  0.02 -0.55  0.17  0.18 -0.46 
Ca21Chr1_0156764 -0.68  0.09  0.29 -0.00 -0.31 -1.04 
Ca21Chr1_0156823 -0.47  0.03 -0.37  0.28  0.32  0.24 
Ca21Chr1_0156882 -0.40 -0.11 -0.18  0.37  0.19 -0.09 
Ca21Chr1_0156941 -0.12  0.49  0.11  0.36  0.36 -0.65 
Ca21Chr1_0157000 -0.17  0.36 -0.09  0.25  0.08 -0.79 
Ca21Chr1_0157059  0.02  0.37 -1.26 -0.53  0.15 -0.82 
Ca21Chr1_0157118 -0.26  0.15 -0.43 -0.08 -0.36 -0.61 
Ca21Chr1_0157177  0.39 -0.20  1.42  0.31 -0.94  0.38 
Ca21Chr1_0157236  0.68  0.36 -0.18  0.33  0.09 -0.38 
Ca21Chr1_0157295  0.53  0.12 -0.75 -0.07  0.19 -1.03 
Ca21Chr1_0157354 -0.68 -0.33 -0.50  0.19  0.10 -0.90 
Ca21Chr1_0157413 -0.71 -0.30 -1.50 -0.10 -0.52 -0.38 
Ca21Chr1_0157472 -0.84 -0.18 -1.35  0.46 -0.61 -0.25 
Ca21Chr1_0157531 -0.47  0.00 -1.59 -0.76 -0.24 -2.14 
Ca21Chr1_0157590 -1.63 -1.16 -2.63 -0.09 -0.12 -0.55 
Ca21Chr1_0157649 -1.03 -0.19 -3.24  0.05 -0.59  0.34  orf19.6024|
Ca21Chr1_0157708 -2.18 -0.52 -3.38  0.01 -0.12  1.04 
Ca21Chr1_0157767 -2.10 -1.23 -3.68  0.25 -0.07  0.16 
Ca21Chr1_0157826 -2.74 -2.19 -3.03  0.35 -0.07  1.70 
Ca21Chr1_0157885 -2.42 -2.15 -2.78 -0.07 -0.73  0.68 
Ca21Chr1_0157944 -1.55 -2.85 -2.98  0.68 -0.23  0.42 
Ca21Chr1_0158003 -1.46 -2.26 -3.28  0.30  0.51  0.31 
Ca21Chr1_0158062 -1.74 -1.56 -2.81  0.30 -0.20 -0.14 
Ca21Chr1_0158121 -1.12 -1.79 -1.99 -0.07 -0.47  0.62 
Ca21Chr1_0158180 -0.84 -1.17 -1.86 -0.01  0.07  0.58 
Ca21Chr1_0158239 -0.26 -0.89 -0.44  0.19  0.10  0.77 
Ca21Chr1_0158298 -0.58 -0.09 -0.75  0.07 -0.37  1.07 
Ca21Chr1_0158357 -0.30  0.04 -0.63 -0.20  0.72  0.94 
Ca21Chr1_0158416 -0.57 -0.01 -0.59 -0.47  0.27 -0.82 
Ca21Chr1_0158475  1.05  0.00 -0.27  0.17 -0.05  0.28 
Ca21Chr1_0158534  0.31 -0.29 -0.53  0.24 -0.23  0.44 
Ca21Chr1_0158593 -0.00  0.05  0.05  0.08 -0.33 -0.53 
Ca21Chr1_0158652 -0.11 -0.08 -0.16  0.20 -0.17  0.07 
Ca21Chr1_0158711  0.70 -0.31 -0.35  0.21 -0.28 -0.08  orf19.6023>
Ca21Chr1_0158770 -0.35 -0.30 -0.29  0.17 -0.30  0.10 
Ca21Chr1_0158829 -0.61 -0.14 -0.65 -0.13 -0.23 -0.39 
Ca21Chr1_0158888 -0.61 -0.11 -0.06 -0.03  0.12 -0.28 
Ca21Chr1_0158947 -0.20 -0.44 -0.45  0.41  0.02  0.25 
Ca21Chr1_0159006 -0.44 -0.91 -0.71  0.69 -0.08 -0.27 
Ca21Chr1_0159065 -0.14  0.04 -0.33 -0.03  0.18 -0.31 
Ca21Chr1_0159124  0.12  0.20 -0.49 -0.12  0.39 -0.13 
Ca21Chr1_0159183  0.59  0.37  0.09  0.23  0.39 -0.12 
Ca21Chr1_0159242  0.34  0.03  0.40  0.11 -0.18  0.09 
Ca21Chr1_0159301  0.22  0.16  0.03 -0.52 -0.29 -0.10 
Ca21Chr1_0159360  0.36  0.39  0.01 -0.13 -0.17  0.10 
Ca21Chr1_0159419  0.03  0.22  0.19  0.20 -0.45 -0.07 
Ca21Chr1_0159478  0.17  0.14  0.06  0.18 -0.52  0.09 
Ca21Chr1_0159537  0.04  0.07  0.28  0.05  0.16  0.21 
Ca21Chr1_0159596  0.34  0.19 -0.13 -0.21  0.50  0.31 
Ca21Chr1_0159655  0.12  0.00  0.17 -0.53 -0.47 -0.15 
Ca21Chr1_0159714 -0.36 -0.01  0.24 -0.13 -0.30  0.10 
Ca21Chr1_0159773 -0.10 -0.09  0.22 -0.19  0.05 -0.20 
Ca21Chr1_0159832  0.11  0.19  0.15  0.00 -0.09  0.04 
Ca21Chr1_0159891  0.35  0.34  0.35 -0.47  0.18 -0.02 
Ca21Chr1_0159950  0.52  0.39  0.24 -0.09  0.02 -0.32 
Ca21Chr1_0160009  0.18  0.28  0.49  0.01 -0.04 -0.15 
Ca21Chr1_0160068  0.45  0.22  0.21 -0.58  0.49 -0.23 
Ca21Chr1_0160127  0.13  0.18 -0.03 -0.15 -0.07 -0.32 
Ca21Chr1_0160186  0.20  0.41  0.21 -0.05 -0.37 -0.11 
Ca21Chr1_0160245 -0.11  0.42  0.46  1.35  0.18  0.01 
Ca21Chr1_0160304 -0.13  0.10  0.35  0.15  0.14 -0.19 
Ca21Chr1_0160363 -0.14  0.17  0.28  0.12  0.31 -0.02 
Ca21Chr1_0160422 -0.12  0.27 -0.12 -0.11 -0.13  0.13 
Ca21Chr1_0160481 -0.05  0.14  0.04 -0.29 -0.07 -0.32  orf19.6023|
Ca21Chr1_0160540 -0.27 -0.17 -0.81  0.32  0.15 -0.03 
Ca21Chr1_0160599  1.35 -0.26  0.14  0.10 -0.07 -0.14 
Ca21Chr1_0160658  0.27 -0.01 -0.10  0.07 -0.05 -0.12 
Ca21Chr1_0160717  1.13  0.41 -0.14  0.67  0.37 -0.14 
Ca21Chr1_0160776 -0.19  0.39  0.19  0.11 -0.25  0.39 
Ca21Chr1_0160835  0.08  0.22  0.30  0.10 -0.49 -0.69 
Ca21Chr1_0160894  0.14  0.31  0.35 -0.04 -0.06  0.39  |orf19.6022
Ca21Chr1_0160953 -0.20 -0.29 -0.05  0.09  0.20  0.11 
Ca21Chr1_0161012 -0.21  0.96  0.07 -0.34 -0.05  0.42 
Ca21Chr1_0161071 -0.62  0.39  0.16 -0.12 -0.74  0.51 
Ca21Chr1_0161130  0.03  0.31  0.16  0.10  0.08  0.13 
Ca21Chr1_0161189  0.19  0.13 -0.31  0.06  0.07  0.38 
Ca21Chr1_0161248 -0.53 -0.11  0.02  0.00 -0.09  0.25 
Ca21Chr1_0161307 -0.22 -0.20 -0.24 -0.15  0.16  0.02 
Ca21Chr1_0161366  0.13  0.16  0.14 -0.01  0.08 -0.09 
Ca21Chr1_0161425  0.22  0.40  0.34  0.22 -0.11 -0.10 
Ca21Chr1_0161484  0.40  0.23 -0.08 -0.38  1.02  0.62 
Ca21Chr1_0161543  0.21  0.11  0.05 -0.22 -0.10  0.38 
Ca21Chr1_0161602 -0.82 -0.04  0.29  0.19  0.40  0.20 
Ca21Chr1_0161661  0.37  0.19  0.26 -0.23  0.21  0.62 
Ca21Chr1_0161720  0.26  0.15 -0.17  0.07  0.36  0.92 
Ca21Chr1_0161779 -0.16  0.12 -0.56  0.11  0.24 -0.43 
Ca21Chr1_0161838  0.14 -0.33  0.28 -0.28  0.03  0.44 
Ca21Chr1_0161897  1.15  0.22 -0.01 -0.09 -0.12  0.45 
Ca21Chr1_0161956  0.52  0.10  0.31  0.19  0.08  0.10 
Ca21Chr1_0162015  0.65  0.12  0.32 -0.20  0.10  0.12 
Ca21Chr1_0162074  0.16  0.31  0.27  0.25 -0.21  0.27 
Ca21Chr1_0162133  0.09  0.11  0.19  0.04 -0.13  0.15 
Ca21Chr1_0162192  0.16  0.12  0.09 -0.15 -0.06  0.75 
Ca21Chr1_0162251  0.17 -0.05 -0.16  0.12 -0.06 -0.19 
Ca21Chr1_0162310  0.07 -0.07  0.01 -0.06  0.03 -0.26 
Ca21Chr1_0162369 -0.08  0.47 -0.07  0.31  0.29 -0.33 
Ca21Chr1_0162428 -0.15  0.18  0.30  0.25  0.55 -0.35 
Ca21Chr1_0162487  0.05  0.13 -0.04  0.20  0.10 -0.35 
Ca21Chr1_0162546  0.04  0.06  0.00  0.24 -0.46 -0.06 
Ca21Chr1_0162605 -0.19  0.27 -0.08  0.00 -0.16  0.22 
Ca21Chr1_0162664  0.04 -0.00 -0.35  0.06 -0.25 -0.08  <orf19.6022
Ca21Chr1_0162723  0.21 -0.19 -0.17 -0.11 -0.00 -0.00 
Ca21Chr1_0162782 -0.52  0.59 -0.33  0.11 -0.02  0.26 
Ca21Chr1_0162841  0.27  0.18 -0.31  0.09  1.04 -0.25 
Ca21Chr1_0162900  0.25  0.31  0.07 -0.13  0.28 -1.27 
Ca21Chr1_0162959 -0.04  0.00 -0.04 -0.05  0.25  1.00 
Ca21Chr1_0163018 -0.57 -0.23 -0.48 -0.09 -0.18  0.33 
Ca21Chr1_0163077  0.33 -0.20 -0.05 -0.66 -0.09  0.87 
Ca21Chr1_0163136  0.93 -0.20 -0.25 -0.23 -0.10  0.37 
Ca21Chr1_0163195 -0.30 -0.06 -0.20 -0.27 -0.06  0.73 
Ca21Chr1_0163254 -0.56  0.25 -0.18  0.05 -0.18  0.00 
Ca21Chr1_0163313 -0.65 -0.30 -0.16 -0.05 -0.11 -0.31 
Ca21Chr1_0163372  0.17 -0.15  0.10  0.35 -0.11  0.14 
Ca21Chr1_0163431 -0.04  0.21 -0.15  0.35 -0.29 -0.05 
Ca21Chr1_0163490 -0.21  0.28  0.24  0.18 -0.40  0.00 
Ca21Chr1_0163549 -0.33  0.42  0.03  0.53 -0.14  0.08 
Ca21Chr1_0163608 -0.17  0.25 -0.40 -0.06 -0.19  0.32 
Ca21Chr1_0163667 -0.24  0.21 -0.91  0.22 -0.33  0.04 
Ca21Chr1_0163726 -0.33 -0.07 -1.43 -0.18  0.03  0.14 
Ca21Chr1_0163785 -0.64 -0.23 -1.38 -0.20  0.05  0.38 
Ca21Chr1_0163844 -0.95 -1.00 -1.69 -0.12 -0.08 -0.24 
Ca21Chr1_0163903 -1.38 -1.15 -2.09 -0.17 -0.37  0.19 
Ca21Chr1_0163962 -1.25 -1.12 -2.17  0.13  0.05  0.45 
Ca21Chr1_0164021 -0.63 -1.77 -1.76 -0.45 -0.29  0.19 
Ca21Chr1_0164080 -1.26 -1.14 -1.65 -0.17 -0.04  0.17 
Ca21Chr1_0164139 -0.80 -1.08 -1.02  0.12 -0.37  0.54 
Ca21Chr1_0164198 -0.33 -0.38 -0.81 -0.06 -0.06  0.16 
Ca21Chr1_0164257 -0.11 -0.34 -0.62 -0.25 -0.49 -0.67 
Ca21Chr1_0164316  0.01 -0.34 -0.06  0.43 -0.00  0.15 
Ca21Chr1_0164375 -0.43  0.13  0.53  0.13 -0.11  0.09 
Ca21Chr1_0164434  0.09  0.43 -0.02 -0.06  0.01  0.26 
Ca21Chr1_0164493  0.42 -0.00  0.57  0.08  0.23  0.02 
Ca21Chr1_0164552  0.03  0.21 -0.39 -0.06  0.20  0.18 
Ca21Chr1_0164611 -0.25  0.23 -0.43 -0.21 -0.18  0.06 
Ca21Chr1_0164670 -0.19 -0.16 -0.75 -0.19 -0.63  0.09 
Ca21Chr1_0164729 -0.55 -0.36 -0.56  0.16  0.25 -0.66 
Ca21Chr1_0164788 -0.07 -0.60 -1.57  0.29  0.02  0.60 
Ca21Chr1_0164847 -0.48 -0.61 -1.21  0.06  0.05  0.36 
Ca21Chr1_0164906 -0.14 -0.75 -0.69 -0.20 -0.65 -0.60 
Ca21Chr1_0164965 -0.34 -1.05 -1.01 -0.05 -0.26 -0.07 
Ca21Chr1_0165024 -1.15 -0.60 -1.18 -0.10 -0.68  0.04 
Ca21Chr1_0165083 -0.94 -0.55 -0.70 -0.11  0.00  0.05 
Ca21Chr1_0165142    NA    NA    NA    NA    NA    NA
Ca21Chr1_0165201 -0.85 -1.11 -1.04  0.13 -0.08  0.35 
Ca21Chr1_0165260 -0.72 -1.13 -0.57 -0.33  0.05 -0.09 
Ca21Chr1_0165319 -0.51 -0.63 -0.57 -0.31  0.00  0.27 
Ca21Chr1_0165378 -0.48 -0.58 -0.40 -0.05 -0.07  0.63 
Ca21Chr1_0165437  0.41 -0.23 -0.08 -0.64  0.08  0.55 
Ca21Chr1_0165496 -0.40  0.24  0.02  0.25 -0.19  0.12 
Ca21Chr1_0165555 -0.20  0.42 -0.28 -0.17  0.48  0.18 
Ca21Chr1_0165614  0.06  0.03 -0.15 -0.35 -0.06  0.25 
Ca21Chr1_0165673 -0.43  1.05 -0.14  0.01 -0.14  0.10 
Ca21Chr1_0165732 -0.02  0.24 -0.14 -0.08  0.03  0.26 
Ca21Chr1_0165791 -0.03  0.11  0.03 -0.13 -0.17  0.35 
Ca21Chr1_0165850  0.48  0.38  0.34 -0.02 -0.14  0.44 
Ca21Chr1_0165909 -0.20 -0.02  0.02 -0.26 -0.43  0.65 
Ca21Chr1_0165968 -0.24 -0.38 -0.18 -0.14 -0.27  0.38 
Ca21Chr1_0166027 -0.39  0.10 -0.20 -0.66 -0.63 -0.08 
Ca21Chr1_0166086 -0.74  0.29  0.03 -0.02 -0.24  0.10 
Ca21Chr1_0166145 -0.44  0.05 -0.16  0.11 -0.32  0.12 
Ca21Chr1_0166204 -0.19 -0.27 -0.20 -0.15  0.00  0.20 
Ca21Chr1_0166263 -0.26  0.01  0.28  0.22 -0.24  0.55 
Ca21Chr1_0166322  0.19 -0.04  0.04 -0.21  0.28 -0.13 
Ca21Chr1_0166381  0.02 -0.19  0.93  0.34  0.03  0.35 
Ca21Chr1_0166440 -0.32 -0.13  0.04 -0.46  0.11 -0.32 
Ca21Chr1_0166499 -0.92  0.01 -0.11  0.23  0.29 -0.24 
Ca21Chr1_0166558 -0.34  0.72  0.40 -0.09 -0.05 -0.13 
Ca21Chr1_0166617 -0.35  0.46  0.13  0.15 -0.03 -0.41 
Ca21Chr1_0166676  0.08  0.15  0.30 -0.06  0.02  0.01 
Ca21Chr1_0166735 -0.10  0.13  0.23  0.14 -0.15  0.40 
Ca21Chr1_0166794  0.01  0.35  0.01 -0.08  0.06  0.05 
Ca21Chr1_0166853  0.33  0.11 -0.15 -0.31  0.07  0.20 
Ca21Chr1_0166912  0.43 -0.03  0.24 -0.11 -0.17  0.27 
Ca21Chr1_0166971  0.15  0.19  0.10 -0.13 -0.23 -0.38 
Ca21Chr1_0167030 -0.16  0.12  0.08  0.32  0.42 -0.32  orf19.6021>
Ca21Chr1_0167089  0.20  0.54  0.63  0.93  0.05  0.30 
Ca21Chr1_0167148  0.58 -0.08 -0.19 -0.14  0.05 -0.53 
Ca21Chr1_0167207 -0.05  0.26  0.29  0.57  0.38 -0.46 
Ca21Chr1_0167266  0.03  0.35  0.09  0.12  0.12  0.28 
Ca21Chr1_0167325  0.23  0.43  1.19 -0.08  0.08 -0.02 
Ca21Chr1_0167384  0.04  0.28  0.40  0.12  0.15 -0.22 
Ca21Chr1_0167443  0.48 -0.35 -0.04  0.16 -0.04  0.05 
Ca21Chr1_0167502  0.16 -0.10  0.01 -0.08  0.02 -0.23 
Ca21Chr1_0167561  0.31  0.41  0.18 -0.12  0.15  0.39 
Ca21Chr1_0167620  0.09  0.38 -0.07  0.17  0.69  0.44 
Ca21Chr1_0167679 -0.36  0.33  0.17  0.15 -0.38  0.24 
Ca21Chr1_0167738  0.21  0.27 -0.30  0.18  0.13 -0.52 
Ca21Chr1_0167797 -0.04  0.55 -0.96  0.42 -0.15 -0.84  orf19.6021|
Ca21Chr1_0167856 -0.42 -0.12  0.01  0.21  0.20  0.58 
Ca21Chr1_0167915  0.23  0.09 -0.34  0.09 -0.58  0.34 
Ca21Chr1_0167974  0.09  0.02 -0.03 -0.20 -0.04 -0.18 
Ca21Chr1_0168033 -0.22 -0.39 -0.85 -0.29 -0.17 -0.48 
Ca21Chr1_0168092 -0.26 -0.34 -0.62 -0.70 -0.04  0.02 
Ca21Chr1_0168151 -0.10 -0.44 -1.06 -0.25  0.02  0.05 
Ca21Chr1_0168210 -0.40 -0.58 -0.75 -0.22 -0.04 -0.04 
Ca21Chr1_0168269  1.69 -0.39 -0.79  0.17 -0.19  0.57 
Ca21Chr1_0168328 -0.42 -0.37 -0.74 -0.07 -0.32 -0.05 
Ca21Chr1_0168387  0.16 -0.03 -0.35  0.36 -0.45 -0.12 
Ca21Chr1_0168446 -0.04 -0.23  0.31 -0.33  0.22 -0.11 
Ca21Chr1_0168505 -0.58 -0.06 -0.17  0.13  0.29 -0.85  orf19.6020>
Ca21Chr1_0168564 -0.03 -0.33  0.26  0.02 -0.19 -0.08 
Ca21Chr1_0168623  1.05  0.12 -0.23  0.07 -0.12  0.31 
Ca21Chr1_0168682 -0.12 -0.22  0.07  0.15  0.02 -0.17 
Ca21Chr1_0168741 -0.02  0.79  0.23  0.19 -0.02 -0.39 
Ca21Chr1_0168800 -0.08  0.09  0.01  0.34 -0.12  0.01 
Ca21Chr1_0168859 -0.20  0.05 -0.16  0.18 -0.20  0.03 
Ca21Chr1_0168918 -0.21  0.22 -0.01 -0.02 -0.00  0.17 
Ca21Chr1_0168977 -0.11  0.19  0.11  0.28 -0.12  0.22 
Ca21Chr1_0169036 -0.57  0.34  0.18  0.15 -0.36 -0.14 
Ca21Chr1_0169095 -0.33  0.14  0.37  0.01  0.26  0.11 
Ca21Chr1_0169154 -0.35  0.10  0.05 -0.35 -0.26  0.31 
Ca21Chr1_0169213 -0.46  0.18 -0.19 -0.11 -0.00  0.97 
Ca21Chr1_0169272  0.02 -0.19  0.15  0.04  0.44  0.15 
Ca21Chr1_0169331 -0.31 -0.06  0.92  0.26  0.20  0.80 
Ca21Chr1_0169390 -0.26  0.18 -0.17  0.34  0.12  0.38 
Ca21Chr1_0169449 -0.87  0.03 -0.22 -0.16  0.41  0.75 
Ca21Chr1_0169508 -0.40  0.29 -0.38  0.17  0.14  0.07  orf19.6020|
Ca21Chr1_0169567  0.27  0.04  0.13 -0.21 -0.11  3.16 
Ca21Chr1_0169626  0.31 -0.03  0.05  0.21 -0.34 -0.16 
Ca21Chr1_0169685 -0.64  0.07 -0.83  0.08 -0.27  1.25 
Ca21Chr1_0169744 -0.59  0.10 -0.74 -0.02 -0.28  0.94 
Ca21Chr1_0169803 -0.22 -0.08 -0.20  0.19 -0.27  1.06 
Ca21Chr1_0169862  0.38 -0.27 -1.01  0.16 -0.05 -0.66 
Ca21Chr1_0169921  0.13 -0.03 -0.01 -0.05  0.30 -0.15 
Ca21Chr1_0169980 -0.14 -0.10 -0.44 -0.10  0.34  0.97 
Ca21Chr1_0170039  0.68  0.12  0.17  0.17 -0.20 -0.05 
Ca21Chr1_0170098  0.20  0.23  0.82 -0.01 -0.28  0.41 
Ca21Chr1_0170157  0.01 -0.09  0.26 -0.37 -0.36  0.42  orf19.6018>
Ca21Chr1_0170216  0.15  0.18  0.33 -0.37 -0.13  0.33 
Ca21Chr1_0170275 -0.10 -0.11  0.02  0.01 -0.31 -0.10 
Ca21Chr1_0170334  0.14  0.96  0.29  0.26 -0.14 -0.01 
Ca21Chr1_0170393 -0.06 -0.02  0.08  0.23 -0.44 -0.50 
Ca21Chr1_0170452 -0.17  0.21  0.32  0.72 -0.09  0.13 
Ca21Chr1_0170511  0.35  0.15  0.10  0.48 -0.15  0.09 
Ca21Chr1_0170570  0.06  0.22 -0.01 -0.03  0.10 -0.04 
Ca21Chr1_0170629  0.14 -0.16 -0.01 -0.11  0.17 -0.20 
Ca21Chr1_0170688 -0.03  0.07  0.27  0.14 -0.24 -0.09 
Ca21Chr1_0170747  0.33  0.13  0.45  0.28  0.18 -0.14 
Ca21Chr1_0170806  0.28  0.25  0.19 -0.02  0.16 -0.07 
Ca21Chr1_0170865    NA    NA    NA    NA    NA    NA
Ca21Chr1_0170924  0.11 -0.13  0.44  0.20 -0.17 -0.53 
Ca21Chr1_0170983  0.15  0.16  0.26  0.00 -0.07 -0.19 
Ca21Chr1_0171042 -0.01  0.06  0.21 -0.19  0.04  0.19 
Ca21Chr1_0171101  0.29 -0.04  0.11  0.18  0.13 -0.32 
Ca21Chr1_0171160  0.02 -0.12 -0.09  0.21  0.37 -0.10 
Ca21Chr1_0171219 -0.04  0.10  0.13  0.16 -0.00  0.05 
Ca21Chr1_0171278  0.78  0.21  0.43  0.22  0.63  0.17 
Ca21Chr1_0171337  0.16 -0.49  0.29  0.26 -0.11  0.14 
Ca21Chr1_0171396  0.24  0.65  0.26  0.30  0.28  0.50 
Ca21Chr1_0171455  0.10 -0.04  0.25  0.25  0.49 -0.09 
Ca21Chr1_0171514  0.25 -0.08 -0.05  0.06  0.21 -0.41 
Ca21Chr1_0171573 -0.61  0.02  0.11  0.42 -0.45 -0.03 
Ca21Chr1_0171632  0.00  0.04  0.21  0.50  0.01 -0.50 
Ca21Chr1_0171691  0.29  0.17  0.39 -0.04  0.09 -0.16 
Ca21Chr1_0171750  0.14  0.14  0.38  0.17  0.09  0.50 
Ca21Chr1_0171809  0.09 -0.06  0.25  0.57  0.10 -0.12 
Ca21Chr1_0171868 -0.13  0.07  0.23  0.12 -0.16 -0.26 
Ca21Chr1_0171927  0.07 -0.04  0.22 -0.47 -0.34 -0.22 
Ca21Chr1_0171986  0.24 -0.08 -0.23 -0.13 -0.28 -0.45 
Ca21Chr1_0172045 -0.05 -0.11  0.30 -0.34  0.07 -0.40 
Ca21Chr1_0172104 -0.38  0.27  0.14 -0.00  0.01 -0.13 
Ca21Chr1_0172163 -0.13  0.11  0.27  0.04 -0.17 -0.32 
Ca21Chr1_0172222 -0.01 -0.23  0.01  0.03 -0.24 -0.48  orf19.6018|
Ca21Chr1_0172281 -0.12  0.33  0.23  0.07 -0.13  0.27 
Ca21Chr1_0172340 -0.05 -2.05  0.05  0.16 -0.06 -0.44 
Ca21Chr1_0172399  0.00  0.28  0.43  0.61 -0.34  0.00 
Ca21Chr1_0172458 -0.01  0.23  0.21  0.37 -0.47 -0.57 
Ca21Chr1_0172517  0.11  0.15 -0.02  0.08  0.27 -0.34 
Ca21Chr1_0172576  0.15  0.08 -0.12 -0.17  0.07 -0.36 
Ca21Chr1_0172635  0.03  0.19 -0.24 -0.06 -0.19  0.01 
Ca21Chr1_0172694  0.13  0.11  0.01 -0.25 -0.23  0.08 
Ca21Chr1_0172753  0.14  0.29  0.04 -0.18 -0.21  0.04 
Ca21Chr1_0172812  0.34  0.29  0.04 -0.25 -0.21 -0.03 
Ca21Chr1_0172871  0.47  0.14  0.14 -0.27  0.04 -0.32 
Ca21Chr1_0172930 -0.11  0.07  0.16  0.58  0.04 -0.12 
Ca21Chr1_0172989 -0.34  0.22  0.32  0.28 -0.15  0.00 
Ca21Chr1_0173048 -0.15  0.16  0.15 -0.26  0.08  0.10 
Ca21Chr1_0173107 -0.26  0.17  0.15 -0.13 -0.09 -0.04 
Ca21Chr1_0173166  0.08 -0.05  0.25 -0.69 -0.21  0.07 
Ca21Chr1_0173225 -0.32 -0.04 -0.11 -0.02 -0.23  0.08 
Ca21Chr1_0173284 -0.40  0.04 -0.11 -0.28 -0.20  0.22 
Ca21Chr1_0173343 -0.51 -0.16 -0.00 -0.30 -0.00  0.31 
Ca21Chr1_0173402 -0.36  0.03 -0.05 -0.08  0.01  0.50 
Ca21Chr1_0173461 -0.18 -0.19 -0.34 -0.42 -0.29  0.71 
Ca21Chr1_0173520 -0.49  0.26 -0.21 -0.15 -0.12  0.17 
Ca21Chr1_0173579 -0.68 -0.07 -0.18  0.04 -0.28  0.40 
Ca21Chr1_0173638 -0.95 -0.07 -0.39  0.10 -0.16  0.11 
Ca21Chr1_0173697 -0.72  0.05 -0.51  0.09 -0.10  0.15 
Ca21Chr1_0173756  0.50 -0.41 -0.26 -0.73 -0.14  0.03 
Ca21Chr1_0173815 -0.53 -0.17 -0.37  0.15 -0.16  0.21 
Ca21Chr1_0173874  0.48 -0.10  0.18  0.03 -0.27  0.40 
Ca21Chr1_0173933 -0.15  0.07 -0.12 -0.06  0.09 -0.87 
Ca21Chr1_0173992 -0.33  0.01 -1.27  0.53 -0.36  0.68 
Ca21Chr1_0174051    NA    NA    NA    NA    NA    NA
Ca21Chr1_0174110 -0.66  0.02 -0.45 -0.04 -0.35  0.83 
Ca21Chr1_0174169 -0.67 -0.14  0.24  0.10 -0.37 -0.25 
Ca21Chr1_0174228 -0.38  0.06 -0.60  0.02 -0.04 -0.39 
Ca21Chr1_0174287 -0.06  0.06 -0.01 -0.32  0.04 -1.49 
Ca21Chr1_0174346 -0.57 -0.38  0.89 -0.10 -0.23  1.02 
Ca21Chr1_0174405 -0.02 -0.04 -0.10 -0.00 -0.18 -0.32  orf19.6017>
Ca21Chr1_0174464  0.05 -0.00 -0.10  0.16  0.22  0.66 
Ca21Chr1_0174523  0.12 -0.05 -0.03  0.20  0.17 -0.03 
Ca21Chr1_0174582  0.06  0.26  0.16  0.37 -0.08 -0.03 
Ca21Chr1_0174641  0.41  0.16  0.53  0.03 -0.23  0.47 
Ca21Chr1_0174700 -0.19  0.02  0.36  0.01 -0.17 -0.25 
Ca21Chr1_0174759  0.41  0.02  0.45  0.37  0.09  0.36 
Ca21Chr1_0174818  0.18  0.30 -0.12  0.52  0.27 -0.19 
Ca21Chr1_0174877 -0.10 -0.02 -0.45  0.31  0.22 -0.27 
Ca21Chr1_0174936  0.03 -0.27  0.11  0.41 -0.05  0.03 
Ca21Chr1_0174995  0.19 -0.18 -0.30 -0.18  0.40 -0.09 
Ca21Chr1_0175054  0.02 -0.39 -0.30 -0.31 -0.71  0.86 
Ca21Chr1_0175113 -0.25 -0.27 -0.48 -0.19 -0.38  0.24 
Ca21Chr1_0175172 -0.19 -0.70 -0.51 -0.04 -0.59  0.03 
Ca21Chr1_0175231 -0.05 -0.71 -0.26  0.21 -0.05  0.09 
Ca21Chr1_0175290 -0.17 -0.52 -0.46  0.05 -0.09  0.15 
Ca21Chr1_0175349 -0.28 -0.26  0.56  0.05 -0.01 -0.21 
Ca21Chr1_0175408  0.05  0.11  0.23  0.32 -0.14  0.34 
Ca21Chr1_0175467  0.06  0.04  0.93  0.28 -0.04 -0.10 
Ca21Chr1_0175526 -0.17 -0.37  0.09  0.04  0.36  0.33 
Ca21Chr1_0175585  0.18 -0.32 -0.02  0.22  0.06 -0.08 
Ca21Chr1_0175644  0.22  0.05 -0.05  0.06  0.22  0.60 
Ca21Chr1_0175703  0.07 -0.03 -0.01  0.01 -0.05 -0.16 
Ca21Chr1_0175762  0.52 -0.15  0.00 -0.00 -0.24 -0.27 
Ca21Chr1_0175821  0.01  0.15  0.92  0.33  0.10 -0.12 
Ca21Chr1_0175880 -0.11  0.30  0.02  0.31  0.12 -0.36 
Ca21Chr1_0175939  0.07  0.31  0.22  0.11 -0.49 -0.13 
Ca21Chr1_0175998  0.12 -0.01 -0.17 -0.04  0.04 -0.18 
Ca21Chr1_0176057  0.25 -0.44 -1.36 -0.28  0.27  0.40  orf19.6017|
Ca21Chr1_0176116 -0.30 -0.27 -0.65  0.12 -0.30  0.14 
Ca21Chr1_0176175 -0.42 -0.30 -0.53  0.17 -0.32  0.42 
Ca21Chr1_0176234 -1.06 -0.36 -0.78  0.08 -0.21 -0.66 
Ca21Chr1_0176293 -0.33 -0.71 -0.44  0.13 -0.04 -0.12  orf19.6016>
Ca21Chr1_0176352 -0.77 -0.21 -0.49  0.13 -0.25 -0.06 
Ca21Chr1_0176411 -0.58 -0.10 -0.31 -0.03 -0.33  0.35 
Ca21Chr1_0176470 -0.02  0.12 -0.09  0.06  0.82 -0.34 
Ca21Chr1_0176529  0.04 -0.01 -0.03  0.20 -0.26  0.28 
Ca21Chr1_0176588 -0.18 -0.18  0.04 -0.35 -0.27  0.03 
Ca21Chr1_0176647 -0.18  0.06 -0.25 -0.26 -0.13  0.43 
Ca21Chr1_0176706 -0.18  0.24 -0.19  0.19 -0.03  0.24 
Ca21Chr1_0176765 -0.13  0.31 -0.39  0.02 -0.32  0.17 
Ca21Chr1_0176824  0.18 -0.15 -0.11  0.05 -0.15 -0.12 
Ca21Chr1_0176883  0.08 -0.01 -0.01 -0.09 -0.06  0.09 
Ca21Chr1_0176942  0.28  0.17 -0.21  0.07 -0.23 -0.29 
Ca21Chr1_0177001  0.17  0.07  0.29  0.14  0.01 -0.26 
Ca21Chr1_0177060  0.11 -0.06  0.07  0.20 -0.22 -1.13 
Ca21Chr1_0177119  0.11 -0.13  0.06  0.37 -0.22 -0.32 
Ca21Chr1_0177178  0.19 -0.16  0.29 -0.09 -0.01 -0.23 
Ca21Chr1_0177237  0.31  0.28  0.09 -0.00 -0.05  0.16 
Ca21Chr1_0177296  0.35  0.07  0.61  0.10  0.20 -0.16 
Ca21Chr1_0177355 -0.14  0.52  0.37 -0.22  0.22 -0.35 
Ca21Chr1_0177414  0.51  0.39  0.36  0.40  0.48 -0.22 
Ca21Chr1_0177473 -0.24  0.10  0.54 -0.14  0.02 -0.33 
Ca21Chr1_0177532  0.13  0.20  0.12  0.03 -0.30  0.66 
Ca21Chr1_0177591  0.36  0.64  0.55 -0.27 -0.19  0.51 
Ca21Chr1_0177650  0.37  0.48  0.04 -0.21 -0.19  0.01 
Ca21Chr1_0177709  0.06 -0.10 -0.23  0.16  0.43  0.42 
Ca21Chr1_0177768 -0.37  0.01  0.20  0.07 -0.30 -0.04 
Ca21Chr1_0177827 -0.24 -0.32  0.08  0.05 -0.08  0.20 
Ca21Chr1_0177886  0.42  0.11  0.26  0.11  0.55 -0.01 
Ca21Chr1_0177945 -0.41 -0.22  0.21  0.18 -0.23  0.04 
Ca21Chr1_0178004 -0.23 -0.09 -0.04  0.10 -0.21  0.13 
Ca21Chr1_0178063  0.06 -0.05 -0.32 -0.49 -0.14  0.06 
Ca21Chr1_0178122 -0.04 -0.07 -0.07  0.02 -0.41  0.23 
Ca21Chr1_0178181 -0.11 -0.15 -0.07 -0.02 -0.75 -0.09 
Ca21Chr1_0178240  0.15  0.06 -0.10 -0.16 -0.22  0.06 
Ca21Chr1_0178299  0.09 -0.19 -0.06 -0.11 -0.41  0.32 
Ca21Chr1_0178358  0.23 -0.13  0.07 -0.05  0.21 -0.22 
Ca21Chr1_0178417  0.17 -0.10  0.01  0.02 -0.23  0.27 
Ca21Chr1_0178476 -0.29 -0.14 -0.14  0.03 -0.58  1.23  orf19.6016|
Ca21Chr1_0178535 -0.04  0.19 -0.38  0.24  0.02  0.51 
Ca21Chr1_0178594 -0.12  0.23 -0.37  0.11  0.34 -0.36 
Ca21Chr1_0178653 -0.07 -0.37  0.24 -0.07 -0.16  0.53  orf19.6014>
Ca21Chr1_0178712 -0.25  0.14 -0.31  0.04 -0.42  0.48 
Ca21Chr1_0178771  0.12  0.16 -0.12 -0.11 -0.07 -0.15 
Ca21Chr1_0178830  0.18 -0.00  0.10  0.02  0.15 -0.06 
Ca21Chr1_0178889 -0.13  0.15  0.36 -0.06 -0.13  0.04 
Ca21Chr1_0178948  0.02  0.18  0.09 -0.24 -0.64 -0.35 
Ca21Chr1_0179007  0.08  0.04  0.14  0.04  0.07  0.05 
Ca21Chr1_0179066 -0.09  0.24  0.13 -0.27  0.12 -0.19 
Ca21Chr1_0179125 -0.17  0.26 -0.01  0.27 -0.03 -0.35 
Ca21Chr1_0179184 -0.03 -0.35  0.27  0.05 -0.26  0.09 
Ca21Chr1_0179243  0.09 -0.01 -0.52  0.04  0.06 -0.07 
Ca21Chr1_0179302 -0.09  0.48  0.28  0.07 -0.88 -0.14  orf19.6014|
Ca21Chr1_0179361  0.66  0.38 -0.45 -0.55 -0.60 -0.14 
Ca21Chr1_0179420  0.34  0.25 -0.17  0.01  0.15 -0.10 
Ca21Chr1_0179479  0.35 -0.22 -0.29 -0.18  0.16 -0.01 
Ca21Chr1_0179538 -0.06 -0.01 -0.33  0.03 -0.07  0.09 
Ca21Chr1_0179597 -0.40 -0.03  0.00  0.63  0.13 -0.72 
Ca21Chr1_0179656 -0.11 -0.08 -0.37 -0.21  0.07 -0.15 
Ca21Chr1_0179715 -0.22 -0.43 -0.15  0.17 -0.32 -0.29 
Ca21Chr1_0179774 -0.30  0.08 -0.53 -0.42 -0.30 -0.22 
Ca21Chr1_0179833 -0.70 -0.31  0.09  0.02 -0.59 -0.57 
Ca21Chr1_0179892  0.02  0.13 -0.29  0.25  0.06 -0.36 
Ca21Chr1_0179951 -0.49 -0.08  0.03  0.02 -0.01 -0.24 
Ca21Chr1_0180010 -0.23  0.23  0.18  0.51 -0.04 -0.01 
Ca21Chr1_0180069  0.45  0.01  0.36  0.04  0.12  0.28 
Ca21Chr1_0180128  0.60  0.03  0.17  0.12  0.09 -0.19 
Ca21Chr1_0180187  0.15 -0.01  0.42  0.02  0.58 -0.38  orf19.6013>
Ca21Chr1_0180246  0.33  0.10  0.27 -0.57  0.25 -0.37 
Ca21Chr1_0180305 -0.12  0.17 -0.04  0.21  0.07 -0.26 
Ca21Chr1_0180364  0.29  0.22  0.40 -0.62  0.10 -0.25 
Ca21Chr1_0180423  0.19  0.23 -0.02 -0.08  0.07  0.24 
Ca21Chr1_0180482  0.37  0.35  0.03 -0.08  0.56  0.16 
Ca21Chr1_0180541 -0.29  0.34  0.44  0.11 -0.25  1.93 
Ca21Chr1_0180600  0.07  0.22  0.05 -0.18 -0.23 -0.04 
Ca21Chr1_0180659 -0.03  0.00  0.19  0.17 -0.24 -0.33 
Ca21Chr1_0180718  0.28 -0.09 -0.42 -0.07 -0.14  0.23 
Ca21Chr1_0180777 -0.58  0.21 -0.06  0.09  0.07 -0.11  orf19.6013|
Ca21Chr1_0180836 -0.57  0.20 -0.06  0.20  0.10 -0.07 
Ca21Chr1_0180895 -0.07 -0.12 -0.39  0.07 -0.47 -0.24 
Ca21Chr1_0180954  0.31 -0.19 -0.97  0.17  0.31 -0.47 
Ca21Chr1_0181013  0.07 -0.07 -0.74  0.18 -0.07 -0.52 
Ca21Chr1_0181072 -0.47 -0.22 -0.96  0.18 -0.08 -0.28 
Ca21Chr1_0181131 -0.29 -0.40 -0.98 -0.07  0.15 -0.11 
Ca21Chr1_0181190    NA    NA    NA    NA    NA    NA
Ca21Chr1_0181249  0.05 -0.19 -0.95 -0.18 -0.33 -0.10 
Ca21Chr1_0181308  0.27 -0.10  0.11  0.03  0.43  0.41 
Ca21Chr1_0181367  0.05 -0.18  0.74 -0.38 -0.06 -0.18  orf19.6012>
Ca21Chr1_0181426  0.32  0.24  0.37 -0.11 -0.37 -0.01 
Ca21Chr1_0181485  0.47 -0.11  0.28  0.16 -0.29 -0.56 
Ca21Chr1_0181544  0.14 -0.23  0.11 -0.37 -0.45 -0.49 
Ca21Chr1_0181603 -0.51 -0.14  0.27  0.33 -0.00 -0.45 
Ca21Chr1_0181662 -0.04 -0.17  0.21  0.06 -0.35 -0.86 
Ca21Chr1_0181721 -0.22  0.24  0.15  0.42 -0.23 -0.38 
Ca21Chr1_0181780 -0.34  0.13  0.39 -0.03  0.07 -0.13 
Ca21Chr1_0181839  0.02  0.36  0.11 -0.08  0.21  0.00 
Ca21Chr1_0181898  0.36 -0.44  0.17 -0.21  0.27 -0.23 
Ca21Chr1_0181957  0.16  0.40  0.24 -0.28  0.23 -0.40 
Ca21Chr1_0182016 -0.13  0.39  0.14  0.28  0.18 -0.12 
Ca21Chr1_0182075 -0.27  0.13  0.24  0.38 -0.15 -0.20 
Ca21Chr1_0182134 -0.06  0.58  0.37  0.14 -0.05 -0.30 
Ca21Chr1_0182193  0.04  0.05  0.21  0.04  0.04 -0.02 
Ca21Chr1_0182252  0.07  0.21  0.29  0.05  0.07 -0.39 
Ca21Chr1_0182311 -0.18  0.30  0.34 -0.11 -0.16  0.13 
Ca21Chr1_0182370  0.24 -0.06  0.33 -0.23 -0.32 -0.15 
Ca21Chr1_0182429  0.09  0.10  0.37 -0.02 -0.20 -0.17 
Ca21Chr1_0182488  0.44  0.30  0.46  0.28 -0.27  0.15 
Ca21Chr1_0182547  0.18  0.03  0.38  0.14 -0.09  0.02 
Ca21Chr1_0182606  0.33  0.10  0.32 -0.04  0.02 -0.05 
Ca21Chr1_0182665 -0.04  0.16  0.41 -0.17  0.18 -0.16 
Ca21Chr1_0182724  0.26  0.23  0.32  0.25 -0.22  0.00 
Ca21Chr1_0182783  0.13  0.34  0.34  0.08 -0.05 -0.24 
Ca21Chr1_0182842  0.37  0.06  0.28 -0.26  0.15  0.05 
Ca21Chr1_0182901 -0.39  0.15 -0.42  0.20 -0.18  0.00 
Ca21Chr1_0182960  0.08  0.36  0.38  0.20 -0.76 -0.21 
Ca21Chr1_0183019  0.28  0.22  0.40 -0.08 -0.08 -0.05 
Ca21Chr1_0183078  0.31  0.52  0.17  0.10 -0.02  0.07 
Ca21Chr1_0183137  0.16  0.06  0.20  0.05 -0.44 -0.17 
Ca21Chr1_0183196  0.08  0.02  0.32  0.01  0.00  0.12 
Ca21Chr1_0183255  0.49  0.09  0.29  0.15 -0.36 -0.44 
Ca21Chr1_0183314  0.34 -0.18  0.41  0.17 -0.19  0.26 
Ca21Chr1_0183373 -0.34  0.01  0.10  0.20 -0.03  0.21 
Ca21Chr1_0183432 -0.18  0.22  0.20  0.57 -0.14  0.40 
Ca21Chr1_0183491 -0.45  0.11  0.39  0.16 -0.18  1.06 
Ca21Chr1_0183550  0.17 -0.05  0.50  0.25 -0.09 -0.10 
Ca21Chr1_0183609  0.21  0.18  0.17  0.61  0.03  0.11 
Ca21Chr1_0183668  0.14  0.17  0.27  0.55 -0.16 -0.40 
Ca21Chr1_0183727 -0.02  0.05  0.30  0.36  0.11 -0.23 
Ca21Chr1_0183786 -0.08  0.14  0.42  0.21  0.05 -0.31 
Ca21Chr1_0183845  0.23  0.17  0.34 -0.24  0.05  0.02 
Ca21Chr1_0183904 -0.15  0.29  0.65 -0.10 -0.54 -0.07 
Ca21Chr1_0183963  0.17 -0.11  0.23 -0.02 -0.71 -0.20 
Ca21Chr1_0184022  0.03 -0.23  0.27  0.26 -0.07 -0.48 
Ca21Chr1_0184081  0.11  0.35  0.37  0.24 -0.40 -0.31 
Ca21Chr1_0184140  0.13 -0.02  0.38  0.03  0.08  0.23 
Ca21Chr1_0184199  0.08  0.17  0.22 -0.14  0.09  0.06 
Ca21Chr1_0184258  0.29  0.40  0.18  0.18  0.22 -0.15 
Ca21Chr1_0184317  0.46 -0.03  0.62  0.58  0.06 -0.19 
Ca21Chr1_0184376 -0.04 -0.13  0.21  0.13  0.19  0.12 
Ca21Chr1_0184435 -0.19 -0.02  0.02  0.54  0.08 -0.22 
Ca21Chr1_0184494  0.43  0.05 -0.08  0.22 -0.16 -0.16 
Ca21Chr1_0184553  0.43  0.08  0.07  0.29  0.02 -0.38 
Ca21Chr1_0184612 -0.08 -0.28  0.24  0.03  0.87  0.00 
Ca21Chr1_0184671 -0.00  0.21  0.32  0.06 -0.31  0.24 
Ca21Chr1_0184730 -0.04  0.01  0.40 -0.45  0.09  0.19 
Ca21Chr1_0184789  0.43  0.97  0.51 -0.18  0.12  0.24 
Ca21Chr1_0184848  0.12  0.26  0.18 -0.19 -0.10  0.14 
Ca21Chr1_0184907  0.04 -0.13  0.44  0.11  0.00  0.29 
Ca21Chr1_0184966  0.12 -0.14  0.11 -0.04  0.21  0.03 
Ca21Chr1_0185025 -0.34 -0.13  0.05  0.99 -0.37  0.17 
Ca21Chr1_0185084  0.70  0.22 -0.33  0.03 -0.10  0.52 
Ca21Chr1_0185143  0.35  0.15  0.28  0.01 -0.05  0.14 
Ca21Chr1_0185202  0.44  0.35  0.28  0.82 -0.31  0.04  orf19.6012|
Ca21Chr1_0185261 -0.19  0.04  1.42  0.15  0.37 -0.24 
Ca21Chr1_0185320  0.11  0.20  0.24  0.34  0.82 -0.12 
Ca21Chr1_0185379 -0.04 -0.00  0.47  0.11  0.27 -0.91 
Ca21Chr1_0185438  0.62  0.08  0.31  0.42 -0.18 -1.04 
Ca21Chr1_0185497  0.20  0.35  0.18  0.20 -0.04 -0.07  |orf19.6011
Ca21Chr1_0185556 -0.02  0.46  0.15 -0.31  0.07 -0.19 
Ca21Chr1_0185615  0.22  0.23  0.25 -0.34  0.07 -0.14 
Ca21Chr1_0185674  0.58  0.27  0.23 -0.21  0.16  0.15 
Ca21Chr1_0185733  0.10  0.43  0.48  0.05  0.07  0.18 
Ca21Chr1_0185792  0.44  0.19  0.21  0.26 -0.03  0.13 
Ca21Chr1_0185851  0.24  0.19  0.41 -0.15  0.98  0.11 
Ca21Chr1_0185910  0.63 -0.08  0.12  0.25  0.21 -0.35 
Ca21Chr1_0185969  1.13 -0.20  0.26  0.10 -0.06 -0.18 
Ca21Chr1_0186028  0.09  0.30 -0.24 -0.08 -0.09 -0.06 
Ca21Chr1_0186087 -0.00  0.21  0.50  0.01  0.08 -0.02 
Ca21Chr1_0186146  0.19 -0.33  0.26  0.03  0.44 -0.02 
Ca21Chr1_0186205 -0.03 -0.02 -0.05 -0.18  0.13  0.42 
Ca21Chr1_0186264  0.06  0.13  0.15  0.20 -0.15  0.34 
Ca21Chr1_0186323  0.28  0.17  0.29  0.12 -0.08  0.47 
Ca21Chr1_0186382  0.32  0.25  0.16 -0.00 -0.04  0.60 
Ca21Chr1_0186441  0.25  0.26  0.28 -0.11 -0.14  0.38 
Ca21Chr1_0186500  0.29  0.18  0.32 -0.03 -0.08  0.26 
Ca21Chr1_0186559  0.41 -0.16  0.06 -0.08 -0.39  0.69 
Ca21Chr1_0186618  0.50  0.15  0.27  0.08  0.24  0.12 
Ca21Chr1_0186677  0.39 -0.00  0.06 -0.26  0.02  0.10 
Ca21Chr1_0186736  0.41  0.19  0.41 -0.04 -0.25  0.29 
Ca21Chr1_0186795  0.81  0.25  0.09  0.08 -0.28  0.28 
Ca21Chr1_0186854  0.50  0.12  0.29 -0.45 -0.35  0.66 
Ca21Chr1_0186913  0.41 -0.07  0.20 -0.03 -0.04 -0.27 
Ca21Chr1_0186972  0.14  0.32  0.06  0.12 -0.10  0.10 
Ca21Chr1_0187031  0.40 -0.01  0.15 -0.05 -0.02  0.01 
Ca21Chr1_0187090  0.14 -0.22  0.09  0.25 -0.33 -0.35 
Ca21Chr1_0187149  0.31 -0.05  0.31  0.09 -0.17 -0.07 
Ca21Chr1_0187208  0.44  0.01  0.33  0.05  0.22 -0.71 
Ca21Chr1_0187267  0.31  0.17  0.01 -0.14  0.15 -0.06 
Ca21Chr1_0187326  0.13  0.20  0.49  0.51  0.26 -0.11 
Ca21Chr1_0187385  0.38  0.33  0.57  0.24  0.38 -0.15 
Ca21Chr1_0187444  0.36  0.22  0.16 -0.09 -0.04 -0.36 
Ca21Chr1_0187503  0.16  0.07  0.32  0.11 -0.03 -0.22 
Ca21Chr1_0187562  0.61  0.17  0.52 -0.00  0.25 -0.13 
Ca21Chr1_0187621  0.58  0.11  0.48  0.01  0.14  0.08 
Ca21Chr1_0187680  0.40  0.09  0.32 -0.04  0.32 -0.25 
Ca21Chr1_0187739  0.18 -0.09  0.20 -0.21  0.71 -0.31 
Ca21Chr1_0187798 -0.45  0.38  0.60 -0.02  0.22 -0.58 
Ca21Chr1_0187857  0.61  0.33  0.63 -0.10  0.11 -0.32 
Ca21Chr1_0187916  0.05  0.26  0.59  0.11 -0.12 -0.10 
Ca21Chr1_0187975  0.25  0.03  0.36 -0.37 -0.10  0.13 
Ca21Chr1_0188034 -0.06  0.09  0.22  0.11  0.21 -0.06 
Ca21Chr1_0188093 -0.05  0.24  0.10  0.01 -0.02 -0.28 
Ca21Chr1_0188152  0.19  0.06  0.25 -0.11 -0.27 -0.28 
Ca21Chr1_0188211  0.27  0.47 -0.05  0.05 -0.19 -0.26 
Ca21Chr1_0188270  0.55  0.91  0.97  0.21  0.05 -0.04 
Ca21Chr1_0188329  0.50  0.29  0.39 -0.13  0.43 -0.01 
Ca21Chr1_0188388  0.19  0.23  0.61  0.22  0.07 -0.08 
Ca21Chr1_0188447  0.58  0.21  0.74 -0.07  0.17 -0.00 
Ca21Chr1_0188506  0.39  0.23  0.45 -0.07  0.25  0.08 
Ca21Chr1_0188565  0.44  0.15  0.89 -0.22 -0.02 -0.20 
Ca21Chr1_0188624  0.32  0.94  0.09 -0.13 -0.02 -0.11 
Ca21Chr1_0188683  0.01  0.05  0.31 -0.17 -0.14 -0.33 
Ca21Chr1_0188742 -0.10  0.05  0.31  0.05 -0.28 -0.29 
Ca21Chr1_0188801  0.29 -0.23  0.24 -0.14  0.10 -0.33 
Ca21Chr1_0188860  0.09  0.15  0.51 -0.15  0.01 -0.04 
Ca21Chr1_0188919  0.48  0.30  0.66 -0.02  0.14 -0.12 
Ca21Chr1_0188978 -0.08  0.07  0.27 -0.08 -0.03 -0.20 
Ca21Chr1_0189037  0.15 -0.05 -0.24 -0.02  0.01 -0.37 
Ca21Chr1_0189096  0.29 -0.21 -0.20  0.10 -0.11 -0.06 
Ca21Chr1_0189155 -0.99 -0.56 -1.10  0.02  0.10 -0.23 
Ca21Chr1_0189214 -0.39 -0.65 -1.17 -0.50  0.16 -0.09 
Ca21Chr1_0189273 -0.42 -0.67 -0.52 -0.29  0.17  0.17 
Ca21Chr1_0189332  0.02 -0.64 -0.66  0.06  0.26 -0.62 
Ca21Chr1_0189391  0.56 -0.40 -0.45 -0.04  0.15 -0.32 
Ca21Chr1_0189450  0.07 -0.18  0.23 -0.07  0.14 -0.18 
Ca21Chr1_0189509 -0.04  0.01  0.45  0.16 -0.03  0.34 
Ca21Chr1_0189568  0.22  0.68  0.39 -0.14  0.03  0.06 
Ca21Chr1_0189627 -0.06 -0.03  0.11  0.14  0.13 -0.07 
Ca21Chr1_0189686 -0.31  0.13  0.20  0.17 -0.05 -0.12 
Ca21Chr1_0189745 -0.29  0.15  0.30  0.25 -0.01  0.03 
Ca21Chr1_0189804 -0.23  0.20  0.28  0.13  0.23  0.02 
Ca21Chr1_0189863  0.24 -0.20  0.47  0.35 -0.34  0.10 
Ca21Chr1_0189922  0.34 -0.13  0.00  0.16 -0.11 -0.02 
Ca21Chr1_0189981  0.17 -0.27  0.39 -0.35  0.01 -0.07 
Ca21Chr1_0190040  0.01  0.05  0.02  0.26  0.10 -0.32 
Ca21Chr1_0190099  0.33  0.39  0.16  0.24 -0.53  0.51  <orf19.6011
Ca21Chr1_0190158  0.45  0.13  0.21 -0.05 -0.33 -0.31 
Ca21Chr1_0190217  1.26 -0.24 -0.56  0.29  0.32 -1.25 
Ca21Chr1_0190276  0.37  0.48  0.19 -0.01  0.07  0.44 
Ca21Chr1_0190335  0.79 -0.01  0.29 -0.01 -0.52 -0.12 
Ca21Chr1_0190394 -0.23 -0.11 -0.18  0.12 -0.20  0.44 
Ca21Chr1_0190453  0.05 -0.38 -0.17  0.03 -0.27 -0.19 
Ca21Chr1_0190512 -0.15 -0.27  0.11  0.14 -0.16  0.43 
Ca21Chr1_0190571 -0.30 -0.64  0.07 -0.18 -0.31  0.22 
Ca21Chr1_0190630  0.25  0.09 -0.07  0.29  0.11 -0.11 
Ca21Chr1_0190689 -0.30  0.02 -0.07 -0.28 -0.59  0.75  orf19.6010.1>
Ca21Chr1_0190748 -0.46  0.08  0.04  0.18 -0.31  0.65 
Ca21Chr1_0190807 -0.05 -0.34 -0.20 -0.11 -0.17 -0.06 
Ca21Chr1_0190866 -0.19  0.33 -0.04 -0.09 -0.11 -0.44 
Ca21Chr1_0190925 -0.08  0.15  0.12  0.05  0.13 -0.21 
Ca21Chr1_0190984  0.12  0.31 -0.07  0.12 -0.20 -0.22 
Ca21Chr1_0191043  0.58  0.25  0.01 -0.19  0.04 -0.36 
Ca21Chr1_0191102  0.09  0.24  0.25  0.10  0.12 -0.08  orf19.6010.1|
Ca21Chr1_0191161 -0.21  0.23  0.32  0.03 -0.05  0.95 
Ca21Chr1_0191220 -1.01  0.65 -0.06 -0.27 -0.03 -1.51 
Ca21Chr1_0191279  0.27 -0.12  0.07  0.12  0.11  0.20 
Ca21Chr1_0191338  0.62 -0.47 -0.10  0.14  0.06  0.28 
Ca21Chr1_0191397  0.22  0.04 -0.07  0.19 -0.45 -0.04  |orf19.6010
Ca21Chr1_0191456  0.21  0.07  0.17  0.05 -0.21  0.11 
Ca21Chr1_0191515  0.32 -0.17  0.32 -0.09 -0.23 -0.21 
Ca21Chr1_0191574  0.40  0.30  0.11  0.03  0.19 -0.12 
Ca21Chr1_0191633  0.86  0.08  0.13  0.14 -0.11 -0.05 
Ca21Chr1_0191692  0.28  0.15  0.04  0.20  0.45  0.17 
Ca21Chr1_0191751  0.11 -0.07  0.19  0.15 -0.12  0.17 
Ca21Chr1_0191810  0.04  0.41  0.30  0.20 -0.20  0.29 
Ca21Chr1_0191869  0.07  0.07  0.22 -0.07 -0.19 -0.31 
Ca21Chr1_0191928  0.31  0.10  0.24  0.02 -0.01 -0.29 
Ca21Chr1_0191987  0.21  0.53  0.23  0.03 -0.03 -0.24 
Ca21Chr1_0192046  0.02  0.02  0.42  0.12 -0.25 -0.40 
Ca21Chr1_0192105 -0.25  0.13 -0.01  0.12 -0.06 -0.19 
Ca21Chr1_0192164  0.15  0.16  0.28 -0.45 -0.25 -0.71 
Ca21Chr1_0192223  0.32  0.36  0.40  0.12 -0.28 -0.02 
Ca21Chr1_0192282  0.35  0.42  0.52  0.02 -0.08  0.31 
Ca21Chr1_0192341  0.51 -0.01  0.44 -0.18 -0.11  0.16 
Ca21Chr1_0192400  0.45  0.11  0.44  0.29 -0.29  0.02 
Ca21Chr1_0192459  0.02  0.05  0.18  0.00 -0.28  0.08 
Ca21Chr1_0192518  0.42  0.16  0.22 -0.73 -0.04 -0.23 
Ca21Chr1_0192577  0.30 -0.00  0.37 -0.08 -0.10 -0.16 
Ca21Chr1_0192636  0.30  0.33  0.10 -0.12 -0.29 -0.00 
Ca21Chr1_0192695  0.13  0.03  0.16 -0.20 -0.18  0.08 
Ca21Chr1_0192754  0.32  0.03  0.29  0.23  0.21 -0.01 
Ca21Chr1_0192813  0.05 -0.13  0.03  0.11  0.04 -0.13 
Ca21Chr1_0192872  0.46  0.62  0.28  0.19  0.05 -0.19 
Ca21Chr1_0192931  0.04  0.24  0.35  0.49  0.15 -0.13 
Ca21Chr1_0192990  0.51  0.08  0.00 -0.02 -0.06 -0.11 
Ca21Chr1_0193049  0.37  0.16  0.46 -0.32 -0.13  0.18 
Ca21Chr1_0193108  0.38  0.11  0.51  0.09 -0.08  0.10 
Ca21Chr1_0193167  0.43  0.24  0.20 -0.04  0.24  0.05 
Ca21Chr1_0193226  0.37  0.32  0.37 -0.00  0.26  0.08 
Ca21Chr1_0193285  0.41  0.04 -0.08 -0.02 -0.18  0.15 
Ca21Chr1_0193344  0.44  0.24  0.12  0.04  0.05  0.35 
Ca21Chr1_0193403 -0.03  0.14  0.15  0.02  0.06 -0.78  <orf19.6010
Ca21Chr1_0193462 -0.09 -0.10  0.40  0.06  0.08  0.22 
Ca21Chr1_0193521  0.41  0.59  0.06 -0.30  0.16  0.78 
Ca21Chr1_0193580  0.08 -0.07  0.47  0.01  0.02  0.08 
Ca21Chr1_0193639  0.16 -0.08 -0.25 -0.50  0.37 -0.29 
Ca21Chr1_0193698 -0.08  0.12  0.59  0.06  0.16  0.13 
Ca21Chr1_0193757  2.34  0.47 -0.14  0.12  0.38 -0.04 
Ca21Chr1_0193816 -0.37 -0.66 -1.53 -0.21  0.27 -0.05 
Ca21Chr1_0193875 -0.90 -0.81 -1.82  0.16  0.46  0.27 
Ca21Chr1_0193934 -1.58 -0.92 -1.76 -0.52 -0.49  0.09 
Ca21Chr1_0193993 -0.48 -0.74 -1.34 -0.23  0.10  0.14 
Ca21Chr1_0194052 -0.50 -0.39 -0.79  0.10 -0.09  0.52 
Ca21Chr1_0194111 -0.10 -0.19 -0.31 -0.04 -0.36  0.37 
Ca21Chr1_0194170 -0.27  0.09 -0.07  0.05 -0.33  0.02 
Ca21Chr1_0194229  0.73  0.25 -0.21  1.05  0.25  0.18 
Ca21Chr1_0194288 -0.49  0.30  0.17 -0.08 -0.22 -0.09 
Ca21Chr1_0194347  0.37  0.20  0.44  0.07  0.13  0.22 
Ca21Chr1_0194406 -0.18  0.20 -0.41 -0.53 -0.15 -0.41 
Ca21Chr1_0194465 -0.04 -0.19 -0.31 -0.45 -0.09  0.13 
Ca21Chr1_0194524  0.17  0.20 -0.57 -0.08 -0.16 -0.26 
Ca21Chr1_0194583 -0.04 -0.01 -0.47  0.57  0.35  0.01 
Ca21Chr1_0194642  0.33  0.31 -0.14  0.02  0.52  0.13  |orf19.6009
Ca21Chr1_0194701  0.22 -0.34  0.05  0.26 -0.22  0.13 
Ca21Chr1_0194760  0.10  0.20  0.30  0.26  0.01  0.27 
Ca21Chr1_0194819  0.52 -0.08 -0.23  0.07 -0.13  0.00 
Ca21Chr1_0194878  0.72  0.24  1.26  0.14 -0.30  0.43 
Ca21Chr1_0194937  0.13  0.31  0.13  0.30  0.19  0.34 
Ca21Chr1_0194996  0.21  0.06  0.26  0.31 -0.09  0.12 
Ca21Chr1_0195055  0.24 -0.03  0.08  0.13 -0.17  0.08 
Ca21Chr1_0195114  0.19 -0.06  0.04  0.44  0.13  0.13 
Ca21Chr1_0195173  0.28  0.05 -0.04  0.21  0.21 -0.19 
Ca21Chr1_0195232  0.19  0.18 -0.12 -0.16  0.06 -0.20 
Ca21Chr1_0195291  0.25  0.16  0.01 -0.45  0.31  0.04 
Ca21Chr1_0195350  0.28  0.01  0.26  0.09 -0.71  0.25 
Ca21Chr1_0195409  0.22  0.11  0.06 -0.13 -0.02  0.27 
Ca21Chr1_0195468  0.49  0.09  0.69 -0.07 -0.11  0.11 
Ca21Chr1_0195527  0.05  0.18  0.73  0.03 -0.00 -0.26 
Ca21Chr1_0195586  0.25  0.08  0.50  0.13  0.16  0.14 
Ca21Chr1_0195645  0.40  0.20  0.53  0.18 -0.12  0.30 
Ca21Chr1_0195704  0.08  0.36  0.47 -0.06 -0.12  0.14 
Ca21Chr1_0195763  0.39  0.12  0.59 -0.22  0.12  0.02 
Ca21Chr1_0195822  0.31  0.25  0.45  0.16  0.33  0.13 
Ca21Chr1_0195881  0.04  0.18  0.33  0.15  0.21 -0.11 
Ca21Chr1_0195940  0.42 -0.08  0.40  0.20  0.19 -0.06 
Ca21Chr1_0195999  0.21  0.18  0.86  0.34 -0.11  0.14 
Ca21Chr1_0196058  0.50 -0.42  0.19  0.15  0.40 -0.30 
Ca21Chr1_0196117  0.41  0.25  0.45  0.12  0.08  0.06 
Ca21Chr1_0196176  0.41  0.16  0.02  0.12  0.13  0.05 
Ca21Chr1_0196235  0.10 -0.08  0.05  0.09 -0.09 -0.59 
Ca21Chr1_0196294  0.21 -0.07  0.23 -0.12  0.20 -0.32 
Ca21Chr1_0196353 -0.04 -0.21  0.38  0.23  0.20 -0.25 
Ca21Chr1_0196412 -0.20 -0.14 -0.23  0.25 -0.01  0.19 
Ca21Chr1_0196471  0.08 -0.24 -0.15 -0.15  0.04  0.24 
Ca21Chr1_0196530  0.52 -0.18  0.17 -0.16  0.02 -0.38 
Ca21Chr1_0196589  0.11  0.29  0.29  0.18 -0.10 -0.89 
Ca21Chr1_0196648  0.34  0.42  0.66  0.11 -0.08 -0.09 
Ca21Chr1_0196707  0.51 -0.06  0.54  0.18  0.32  0.56 
Ca21Chr1_0196766  0.25  0.08 -0.24  0.00  0.16 -0.18 
Ca21Chr1_0196825  0.10  0.20  0.44 -0.47  0.18  0.28 
Ca21Chr1_0196884  0.46 -0.01  0.11 -0.11  0.18 -0.19 
Ca21Chr1_0196943  0.14 -0.03  1.04 -0.32  0.04 -0.23 
Ca21Chr1_0197002  0.03  0.16  0.38 -0.06  0.09  0.13 
Ca21Chr1_0197061 -0.07  0.27  0.21  0.16  0.38 -0.02 
Ca21Chr1_0197120  0.35  0.40  0.35  0.09  0.29  0.17 
Ca21Chr1_0197179  0.55  0.56  0.19 -0.10  0.24  0.19 
Ca21Chr1_0197238  0.42  0.63  0.38  0.02  0.29  0.37 
Ca21Chr1_0197297  0.43  0.13  0.47 -0.26  0.09 -0.14 
Ca21Chr1_0197356  0.34 -0.02  0.42 -0.18 -0.07 -0.30 
Ca21Chr1_0197415  0.16  0.33  0.25  0.02 -0.22  0.14 
Ca21Chr1_0197474  0.08  0.35  0.30  0.11  0.02 -0.10 
Ca21Chr1_0197533  0.00  0.03  0.58  0.35  0.27 -0.24 
Ca21Chr1_0197592 -0.30 -0.03  0.35 -0.25 -0.63 -0.61 
Ca21Chr1_0197651  0.36  0.24  0.48 -0.22  0.53 -0.43 
Ca21Chr1_0197710  0.02  0.41  0.58 -0.69  0.24 -0.01 
Ca21Chr1_0197769  0.12  1.04  0.26 -0.25  0.39 -0.01  <orf19.6009
Ca21Chr1_0197828  0.26  0.20  0.68 -0.24  0.31  0.00 
Ca21Chr1_0197887  0.57 -0.11  0.22 -0.35  0.21 -0.03 
Ca21Chr1_0197946  0.72  0.20  0.28 -0.06 -0.09  0.44 
Ca21Chr1_0198005 -0.13  0.21  0.07  0.26  0.13  0.25 
Ca21Chr1_0198064 -0.37  0.24  0.12  0.50  0.33  0.84 
Ca21Chr1_0198123 -0.03  0.15  0.10  0.54 -0.18  0.03 
Ca21Chr1_0198182  0.11  0.12 -0.09 -0.21 -0.18  0.10  orf19.6008.3>
Ca21Chr1_0198241 -0.25  0.23  0.08  0.39 -0.19  0.41 
Ca21Chr1_0198300 -0.11 -0.24  0.16  0.25 -0.13  0.32 
Ca21Chr1_0198359  0.26 -0.03  0.07  0.22  0.12  0.22 
Ca21Chr1_0198418  0.10 -0.16  0.17  0.15  0.10  0.27 
Ca21Chr1_0198477  0.07  0.16  0.43  0.21  0.21  0.13 
Ca21Chr1_0198536  0.35  0.14  0.38  0.69  0.21  0.53 
Ca21Chr1_0198595  0.15  0.26  0.19  0.30  0.11 -0.58 
Ca21Chr1_0198654  0.36  0.38  0.80  0.05  0.15  0.17 
Ca21Chr1_0198713 -0.03  0.21  0.36  0.06 -0.61 -0.29  orf19.6008.3|
Ca21Chr1_0198772 -0.03  0.44  0.32 -0.04  0.26 -0.35 
Ca21Chr1_0198831  0.20  0.17 -0.07 -0.15  0.08 -0.25 
Ca21Chr1_0198890  0.20 -0.05  0.11 -0.22  0.10 -0.21 
Ca21Chr1_0198949 -0.35 -0.03  0.40  0.15  0.13 -0.19  orf19.6008.4>
Ca21Chr1_0199008 -0.07  0.34  0.33  0.23  0.20  0.15 
Ca21Chr1_0199067 -0.07 -0.15  0.32 -0.12  0.01 -0.05 
Ca21Chr1_0199126  0.45  0.12  0.36  0.13  0.12 -0.08 
Ca21Chr1_0199185 -0.13  0.05  0.65 -0.31 -0.03 -0.21  orf19.6008.4|
Ca21Chr1_0199244  0.76  0.29 -0.93  0.36 -0.48 -0.45 
Ca21Chr1_0199303  0.39  0.13  0.38  0.23 -0.23  0.80  |orf19.3288 |orf19.3288.1
Ca21Chr1_0199362 -0.17 -0.54  0.48  0.08  0.41  0.40 
Ca21Chr1_0199421  0.05  0.02  0.29 -0.00  0.06 -0.13 
Ca21Chr1_0199480  0.07  0.10  0.25  0.19  0.36 -0.01 
Ca21Chr1_0199539  0.08  0.71  0.15 -0.37  0.11 -0.18 
Ca21Chr1_0199598 -0.07  0.04  0.01 -0.27 -0.14 -0.29 
Ca21Chr1_0199657  0.13 -0.03  0.20  0.35 -0.11 -0.02 
Ca21Chr1_0199716  0.21 -0.50  0.37  0.02  0.28 -0.30 
Ca21Chr1_0199775  0.18 -0.09  0.34 -0.17  0.41 -0.17 
Ca21Chr1_0199834  0.21 -0.05  0.56  0.03 -0.11  0.15 
Ca21Chr1_0199893  0.23  0.04  0.44 -0.15  0.44  0.18 
Ca21Chr1_0199952  0.08  0.02 -0.07  0.12  0.02 -0.07 
Ca21Chr1_0200011  0.04  0.30  0.41 -0.22 -0.03  0.18 
Ca21Chr1_0200070  0.03  0.18  0.27  0.06 -0.08 -0.39 
Ca21Chr1_0200129 -0.02  0.65  0.57  0.30  0.07 -0.11 
Ca21Chr1_0200188  0.33 -0.09  0.38 -0.22  0.01 -0.21 
Ca21Chr1_0200247  0.27 -0.02  0.19 -0.05  0.45  0.04 
Ca21Chr1_0200306    NA    NA    NA    NA    NA    NA
Ca21Chr1_0200365    NA    NA    NA    NA    NA    NA
Ca21Chr1_0200424 -0.06 -0.25  0.17  0.12  0.28  0.10 
Ca21Chr1_0200483  0.11 -0.33 -0.03  0.10  0.31  0.15 
Ca21Chr1_0200542 -0.00 -0.02  0.33  0.28  0.01  0.05 
Ca21Chr1_0200601  0.13  0.16  0.46  0.23  0.72 -0.02 
Ca21Chr1_0200660  0.23 -0.01  0.18  0.18  0.26 -0.15 
Ca21Chr1_0200719  0.26 -0.16  0.24  0.58  0.36  0.25 
Ca21Chr1_0200778  0.27  0.33  0.36  0.17  0.09  0.49 
Ca21Chr1_0200837  0.17  0.06  0.39 -0.41  0.66  0.18 
Ca21Chr1_0200896  0.09  0.03  0.42 -0.42 -0.14  0.02 
Ca21Chr1_0200955  0.42  0.10  0.37 -0.47  0.37 -0.08  <orf19.3288
Ca21Chr1_0201014  0.12  0.16  0.39  0.13  0.40  0.23 
Ca21Chr1_0201073  0.32 -0.13  0.24  0.19  0.57 -0.38 
Ca21Chr1_0201132  0.06  0.14  0.23  0.08  0.39  0.79 
Ca21Chr1_0201191  0.27  0.08  0.17 -0.29  0.18 -0.19 
Ca21Chr1_0201250  0.14 -0.17  0.16  0.32  0.09 -0.21 
Ca21Chr1_0201309  0.42  0.04  0.34 -0.09  0.40  0.04 
Ca21Chr1_0201368  0.21 -0.17  0.39 -0.15  0.41  0.14 
Ca21Chr1_0201427  0.07 -0.01  0.25  0.04  0.45 -0.11 
Ca21Chr1_0201486  0.29  0.04  0.35  0.07  0.04 -0.28 
Ca21Chr1_0201545  0.29  0.39  0.54 -0.52  0.28  0.02 
Ca21Chr1_0201604  0.26  0.09  0.34  0.50  0.91  0.39 
Ca21Chr1_0201663  0.05  0.12  0.29 -0.19  0.28  0.27 
Ca21Chr1_0201722  0.13  0.37  0.44 -0.07 -0.06  0.08 
Ca21Chr1_0201781  0.25 -0.07  0.65  0.09  0.38  0.04 
Ca21Chr1_0201840  0.45  0.08  0.32 -0.47  0.17  0.05 
Ca21Chr1_0201899  0.17  0.24  0.21  0.23  0.25  0.91 
Ca21Chr1_0201958  0.15  0.22  0.23 -0.13  0.24 -0.01 
Ca21Chr1_0202017 -0.16  0.51  0.30 -0.25  0.12 -0.02 
Ca21Chr1_0202076 -0.80  0.34 -0.12  0.72  0.33  0.02 
Ca21Chr1_0202135  0.34 -0.05  0.29 -0.18  0.04  0.13 
Ca21Chr1_0202194  0.00  0.13  0.38  0.20  0.31  0.47 
Ca21Chr1_0202253  0.23 -0.23  0.07 -0.38  0.60 -0.02  <orf19.3288.1
Ca21Chr1_0202312  0.24 -0.20 -0.43  0.12 -0.39 -0.05 
Ca21Chr1_0202371 -0.13 -0.46 -0.11  0.47  0.11  0.31 
Ca21Chr1_0202430 -0.05 -0.02  0.03  0.14  0.13  0.24 
Ca21Chr1_0202489  0.20  0.14  0.09  0.17 -0.21  0.24  |orf19.3289
Ca21Chr1_0202548 -0.03  0.15  0.24  0.40 -0.23  0.07 
Ca21Chr1_0202607  0.61  0.18  0.18  0.18 -0.20 -0.40 
Ca21Chr1_0202666  0.31  0.23  0.47  0.11  0.31 -0.19 
Ca21Chr1_0202725  0.34  0.16  0.09 -0.02  0.43 -0.32 
Ca21Chr1_0202784  0.23  0.05  0.35 -0.21  0.34 -0.08 
Ca21Chr1_0202843  0.11 -0.03 -0.05 -0.13  0.05 -0.04 
Ca21Chr1_0202902 -0.06 -0.14 -0.26  0.11  0.10  0.57 
Ca21Chr1_0202961 -0.06  0.14 -0.12 -0.10  0.12 -0.12 
Ca21Chr1_0203020 -0.20 -0.04 -0.20 -0.69  0.06 -0.09 
Ca21Chr1_0203079  0.07 -0.11 -0.24 -0.26  0.08 -0.01 
Ca21Chr1_0203138  0.11 -0.11  0.00 -0.29  0.25 -0.00 
Ca21Chr1_0203197  0.47  0.14  0.14 -0.38 -0.29 -0.00 
Ca21Chr1_0203256  0.16  0.15  0.04 -0.32  0.07 -0.01 
Ca21Chr1_0203315  0.00  0.08  0.23  0.40  0.22  0.40 
Ca21Chr1_0203374 -0.11 -0.38  0.20 -0.08  0.17  0.58 
Ca21Chr1_0203433  0.08  0.24 -0.26  0.07  0.02  0.20 
Ca21Chr1_0203492  0.29 -0.05  0.05  0.25  0.70 -0.30 
Ca21Chr1_0203551  0.15  0.33  0.04  0.11  0.03 -0.15 
Ca21Chr1_0203610  0.19  0.08 -0.15  0.17  0.19 -0.29  <orf19.3289
Ca21Chr1_0203669 -0.68  0.08 -0.15  0.04 -0.70 -0.22 
Ca21Chr1_0203728  0.11  0.31 -0.03 -0.02  0.00 -0.06 
Ca21Chr1_0203787    NA    NA    NA    NA    NA    NA
Ca21Chr1_0203846  0.12 -0.29  0.24  0.35 -0.01 -0.24 
Ca21Chr1_0203905  0.23 -0.07  0.19 -0.25  0.35 -0.09  orf19.3290>
Ca21Chr1_0203964  0.39 -0.08  0.33  0.20  0.19 -0.08 
Ca21Chr1_0204023  0.63  0.10  0.36  0.12  0.20 -0.23 
Ca21Chr1_0204082  0.16  0.21  0.17  0.19  0.39 -0.39 
Ca21Chr1_0204141  0.83  0.58  0.11 -0.14  0.15 -0.21 
Ca21Chr1_0204200  0.22  0.27  0.41  0.17  0.84  0.03 
Ca21Chr1_0204259  0.31  0.33  0.16 -0.47  0.36  0.28 
Ca21Chr1_0204318  0.29  0.17  0.24  0.07  0.04  0.24 
Ca21Chr1_0204377  0.31  0.10 -0.14  0.06  0.04  0.54  orf19.3290|
Ca21Chr1_0204436 -0.42  0.18  0.37 -0.10 -0.08  0.05 
Ca21Chr1_0204495 -0.70  0.26  0.03  0.29  0.07 -0.08 
Ca21Chr1_0204554  0.09  0.24  0.11  0.23  0.41 -0.45 
Ca21Chr1_0204613  0.03  0.10  0.25 -0.25 -0.14  0.49 
Ca21Chr1_0204672 -0.53 -0.29 -0.79 -0.34  0.41  0.08 
Ca21Chr1_0204731 -0.29 -0.22 -0.14 -0.34  0.12  0.11 
Ca21Chr1_0204790 -0.56 -0.21 -0.66 -0.46 -0.03  0.62 
Ca21Chr1_0204849 -0.91 -0.14 -0.47 -0.84 -0.18  0.35 
Ca21Chr1_0204908 -0.79  0.18 -0.49 -0.60 -0.13 -0.22 
Ca21Chr1_0204967  0.26  0.46 -0.31 -0.01  0.25  0.25  tA(AGC)4>
Ca21Chr1_0205026 -0.16  0.54 -0.77 -0.56  0.19  0.03  tA(AGC)4|
Ca21Chr1_0205085 -0.50 -0.21 -0.27 -0.29  0.16  0.43 
Ca21Chr1_0205144  0.46  0.03  0.03 -0.26  0.04  0.31 
Ca21Chr1_0205203  0.12 -0.26  0.15 -0.23  0.77  0.40 
Ca21Chr1_0205262 -0.09  0.03 -0.14 -0.07  0.26  0.89  orf19.3291>
Ca21Chr1_0205321 -0.08 -0.01  0.30 -0.22 -0.19 -0.34 
Ca21Chr1_0205380 -0.14  0.09  0.34  0.10 -0.24  0.33 
Ca21Chr1_0205439  0.39  0.37  0.33 -0.22  0.04  0.08 
Ca21Chr1_0205498  0.11  0.10  0.53 -0.29  0.23 -0.08 
Ca21Chr1_0205557  0.05 -0.27  0.02 -0.10  0.06  0.11 
Ca21Chr1_0205616 -0.00  0.19  0.29  0.09  0.12  0.17 
Ca21Chr1_0205675  0.18  0.29  0.85 -0.15  0.16  0.30 
Ca21Chr1_0205734  0.20  0.48  0.61  0.09  0.51  0.06 
Ca21Chr1_0205793 -0.16  0.59  0.72  0.26  0.18  0.04 
Ca21Chr1_0205852 -0.13  0.16  0.45  0.00  0.40 -0.25 
Ca21Chr1_0205911 -0.01  0.38  0.14  0.28  0.12 -0.39 
Ca21Chr1_0205970  0.08  0.27  0.51 -0.13  0.26 -0.20 
Ca21Chr1_0206029  0.18  0.21  0.53  0.10 -0.11 -0.16 
Ca21Chr1_0206088 -0.01  0.09  0.39  0.20  0.39 -0.07 
Ca21Chr1_0206147  0.20 -0.05 -0.06 -0.50  0.35 -0.07 
Ca21Chr1_0206206 -0.00 -0.05  0.25  0.03 -0.16 -0.30 
Ca21Chr1_0206265 -0.05  0.18  0.25  0.20  0.24  0.24  orf19.3291|
Ca21Chr1_0206324  0.14  0.11  0.68  0.40  0.49 -0.06 
Ca21Chr1_0206383 -0.10  0.08  0.29  0.19 -0.15  0.06 
Ca21Chr1_0206442  0.38  0.07  0.45 -0.01  0.15  0.43  orf19.3292>
Ca21Chr1_0206501  0.47 -0.02  0.10 -0.16 -0.07  0.14 
Ca21Chr1_0206560 -0.02  0.30  0.24 -0.15 -0.11 -0.12 
Ca21Chr1_0206619  0.15  0.11  0.23 -0.37 -0.04  0.29 
Ca21Chr1_0206678 -0.01  0.62  0.70  0.12  0.23 -0.01 
Ca21Chr1_0206737  0.09  0.07  0.01  0.15  0.05 -0.45 
Ca21Chr1_0206796  0.56  0.21  0.06 -0.25  0.12 -0.90 
Ca21Chr1_0206855  0.50  0.34  0.10  0.14  0.04  0.06 
Ca21Chr1_0206914  0.29  0.46  0.17 -0.10  0.02 -0.63  |orf19.3293 orf19.3292|
Ca21Chr1_0206973  0.95 -0.05  0.17 -0.16 -0.01  0.17 
Ca21Chr1_0207032  0.69 -0.98 -0.00 -0.24 -0.12  0.14 
Ca21Chr1_0207091  0.23 -0.17  0.12  0.39 -0.15  0.22 
Ca21Chr1_0207150  0.50 -0.11  0.12  0.31  0.25 -0.30 
Ca21Chr1_0207209  0.74 -0.10 -0.21  0.26  0.30  0.36 
Ca21Chr1_0207268  0.46 -0.05  0.13  0.13  0.04  0.05 
Ca21Chr1_0207327  0.14  0.14  0.01  0.24  0.22 -0.03 
Ca21Chr1_0207386  0.51  0.11  0.06 -0.05  0.16 -0.08 
Ca21Chr1_0207445  0.05  0.06  0.11 -0.14  0.20  0.27 
Ca21Chr1_0207504  0.05  0.08  0.10  0.01 -0.12  0.08 
Ca21Chr1_0207563  0.40  0.17  0.60 -0.01  0.38 -0.00 
Ca21Chr1_0207622  0.70  0.78  0.29  0.33  0.12 -0.06 
Ca21Chr1_0207681  0.64  0.24  0.19 -0.04  0.01 -0.10 
Ca21Chr1_0207740  0.37  0.40  0.28  0.15 -0.07 -0.13 
Ca21Chr1_0207799  0.44  0.30  0.29  0.25 -0.08  0.17 
Ca21Chr1_0207858  0.34  0.21  0.17 -0.00 -0.03 -0.08  <orf19.3293
Ca21Chr1_0207917    NA    NA    NA    NA    NA    NA
Ca21Chr1_0207976  0.15  0.03 -0.38  0.16  0.00  0.37 
Ca21Chr1_0208035 -0.52  0.16 -0.22 -0.12  1.57  0.41 
Ca21Chr1_0208094  1.29 -0.37 -0.70  0.08 -0.12  0.05 
Ca21Chr1_0208153  0.03 -0.32 -0.30 -0.05  0.81  0.15 
Ca21Chr1_0208212 -0.66 -0.33 -1.37 -0.41 -0.52 -0.10 
Ca21Chr1_0208271 -0.02 -0.42 -0.14 -0.05 -0.13  0.02  orf19.3294>
Ca21Chr1_0208330 -0.30  0.32 -0.13  0.32 -0.51  0.10 
Ca21Chr1_0208389  0.03 -0.09 -0.00  0.04 -0.14 -0.43 
Ca21Chr1_0208448 -0.15 -0.10  0.11 -0.24  0.14 -0.16 
Ca21Chr1_0208507  0.28 -0.11  0.04 -0.22  0.20 -0.28 
Ca21Chr1_0208566  0.26  0.04  0.24 -0.14  0.57  0.11 
Ca21Chr1_0208625 -0.21  0.03  0.24 -0.16  0.30 -0.22 
Ca21Chr1_0208684 -0.34  0.17  0.24 -0.40  0.50 -0.41 
Ca21Chr1_0208743 -0.22  0.44  0.24  0.14  0.27 -0.25  orf19.3294|
Ca21Chr1_0208802  0.08  0.09  0.47  0.22 -0.20  0.44 
Ca21Chr1_0208861  0.72 -0.41  1.26  0.24 -0.14 -0.09 
Ca21Chr1_0208920  0.89 -0.23  0.02 -0.09  0.36 -0.07 
Ca21Chr1_0208979  0.34  0.29  0.37  0.31 -0.39 -0.07 
Ca21Chr1_0209038  0.34  0.44  0.42  0.17 -0.04  0.00  |orf19.3295
Ca21Chr1_0209097 -0.51  0.34 -0.19 -0.08  0.20 -0.01 
Ca21Chr1_0209156  0.32  0.00  0.37 -0.06  0.37 -0.10 
Ca21Chr1_0209215  0.89  0.36  0.65  0.02  0.20  0.13 
Ca21Chr1_0209274  0.27  0.09  0.73  0.04  0.16 -0.08 
Ca21Chr1_0209333  0.10  0.15  0.14 -0.26  0.34 -0.16 
Ca21Chr1_0209392  0.36  0.36  0.47 -0.03  0.54 -0.11 
Ca21Chr1_0209451  0.94  0.38  0.53 -0.17  0.44 -0.07 
Ca21Chr1_0209510  0.22  0.41  0.64  0.06  0.15  0.35 
Ca21Chr1_0209569  0.34  0.22  0.48  0.62 -0.07 -0.61 
Ca21Chr1_0209628  0.17  0.04  0.64  0.26  0.32 -0.29 
Ca21Chr1_0209687  0.26  0.15  0.62  0.11  0.05 -0.03 
Ca21Chr1_0209746  0.31 -0.45  0.44  0.30  0.23 -0.01 
Ca21Chr1_0209805  0.53 -0.29  0.86  0.07  0.25  0.61 
Ca21Chr1_0209864  0.19 -0.09  0.36  0.23  0.06 -0.09 
Ca21Chr1_0209923  0.61  0.19  0.30  0.16  0.87 -0.09 
Ca21Chr1_0209982 -0.20  0.34  0.54  0.28  0.13  0.16 
Ca21Chr1_0210041  0.26  0.77  0.15  0.09  0.27 -0.04 
Ca21Chr1_0210100  0.50  0.17  0.61  0.54  0.01 -0.00 
Ca21Chr1_0210159 -0.01  0.26  0.31 -0.11 -0.53 -0.31 
Ca21Chr1_0210218  0.47  0.32  0.47 -0.15  0.07 -0.04 
Ca21Chr1_0210277  0.33  0.04  0.52  0.20  0.15 -0.03 
Ca21Chr1_0210336  0.49  0.46  0.63  0.14  0.66 -0.44 
Ca21Chr1_0210395  0.55  0.29  0.47  0.07  0.25 -0.17 
Ca21Chr1_0210454 -0.01  0.05  0.28 -0.08  0.01 -0.19  <orf19.3295
Ca21Chr1_0210513 -0.37  0.08  0.45 -0.02 -0.22 -0.34 
Ca21Chr1_0210572  0.01  0.07  0.13 -0.35 -0.30  0.36 
Ca21Chr1_0210631  0.48  0.01 -0.11  0.05  0.15  0.36 
Ca21Chr1_0210690  0.43 -0.18  0.29 -0.13  0.59  0.53 
Ca21Chr1_0210749  0.41 -0.19  0.32 -0.26  0.16  0.29 
Ca21Chr1_0210808  0.02 -0.17  0.26  0.45 -0.11  0.06 
Ca21Chr1_0210867  0.26  0.04  0.32  0.09 -0.28 -0.29  orf19.3296>
Ca21Chr1_0210926  0.01  0.07  0.19 -0.09 -0.56 -0.05 
Ca21Chr1_0210985  0.25  0.78  0.37  0.08  0.05  0.00 
Ca21Chr1_0211044  0.24  0.05  0.53 -0.37 -0.19 -0.20 
Ca21Chr1_0211103 -0.01 -0.06  0.33 -0.03 -0.02 -0.39 
Ca21Chr1_0211162  0.21 -0.03 -0.36  0.10 -0.26 -0.49 
Ca21Chr1_0211221  0.14  0.12  0.34  0.69  0.06 -0.43 
Ca21Chr1_0211280 -0.27  0.50  0.41 -0.07 -0.12 -0.41 
Ca21Chr1_0211339  0.03 -0.00  0.15  0.07  0.08 -0.52 
Ca21Chr1_0211398 -0.17  0.13  0.13  0.33  0.04 -0.52 
Ca21Chr1_0211457 -0.17  0.13  0.08  0.12 -0.08 -0.08 
Ca21Chr1_0211516 -0.32  0.09  0.09  0.10 -0.08 -0.13 
Ca21Chr1_0211575 -0.03 -0.20 -0.51  0.01 -0.58 -0.04 
Ca21Chr1_0211634 -0.19  0.05 -0.33 -0.00 -0.10 -0.03 
Ca21Chr1_0211693 -0.26 -0.45 -0.47  0.12 -0.08 -0.10 
Ca21Chr1_0211752  0.17 -0.51 -0.83 -0.15  0.22  0.07 
Ca21Chr1_0211811 -0.62 -0.37 -0.71  0.27 -0.15 -0.30 
Ca21Chr1_0211870  0.36 -0.18 -0.42  0.17  0.00  0.79 
Ca21Chr1_0211929  0.09 -0.14 -0.22  0.36  0.14  0.20 
Ca21Chr1_0211988  0.28  0.11  0.10 -0.16  0.18 -0.33 
Ca21Chr1_0212047 -0.06 -0.25  0.27  0.20 -0.21 -0.12 
Ca21Chr1_0212106 -0.06  0.13 -0.08 -0.25  0.23 -0.00 
Ca21Chr1_0212165  0.21  0.06  0.09 -0.28  0.62 -0.06 
Ca21Chr1_0212224  0.19 -0.35  0.09  0.07  0.29 -0.15 
Ca21Chr1_0212283  0.26  0.11  0.15  0.43  0.03  0.04 
Ca21Chr1_0212342 -0.01 -0.69 -0.05 -0.05  0.22  0.28  orf19.3296|
Ca21Chr1_0212401  0.45 -0.06 -0.16  0.04 -0.12  0.57  |orf19.3297
Ca21Chr1_0212460 -0.46  0.21 -0.40 -0.05  0.21  0.51 
Ca21Chr1_0212519 -0.25  0.34 -0.57  0.23  0.06  0.01 
Ca21Chr1_0212578 -0.48 -0.18 -0.74  0.40 -0.03  0.17 
Ca21Chr1_0212637 -0.36 -0.26 -1.30 -0.19  0.05  0.07 
Ca21Chr1_0212696 -0.76 -0.32 -1.11 -0.20  0.06  0.27  <orf19.3297
Ca21Chr1_0212755 -0.62 -0.70 -1.63  0.07 -0.00  0.19 
Ca21Chr1_0212814 -0.88 -1.47 -0.64 -0.13 -0.00  0.42 
Ca21Chr1_0212873 -2.36 -0.76 -2.01  0.40  0.20 -0.15 
Ca21Chr1_0212932 -1.53 -1.17 -2.11  0.04 -0.17  0.15 
Ca21Chr1_0212991 -1.38 -0.89 -1.53 -0.04 -0.04 -0.01 
Ca21Chr1_0213050 -0.56 -1.12 -1.32  0.21 -0.12  1.04 
Ca21Chr1_0213109 -0.36 -0.81 -1.35  0.22  0.02  0.08 
Ca21Chr1_0213168  0.71 -0.29 -0.95  0.13  0.39 -0.28 
Ca21Chr1_0213227 -0.33 -0.67 -1.45 -0.09 -0.25  0.23 
Ca21Chr1_0213286 -0.41 -0.44 -0.97  0.14 -0.20  0.19 
Ca21Chr1_0213345 -0.31 -0.83 -0.83  0.01 -0.36  0.20 
Ca21Chr1_0213404 -0.46 -0.23 -0.46 -0.00  0.09 -0.46 
Ca21Chr1_0213463 -0.35  0.00 -0.21 -0.08  0.24 -0.18 
Ca21Chr1_0213522  0.08  0.43 -0.21  0.19 -0.00  0.11 
Ca21Chr1_0213581  0.14 -0.18 -0.27 -0.03 -0.39  0.18 
Ca21Chr1_0213640  0.08 -0.13 -0.06  0.31  0.03 -0.08  orf19.3298>
Ca21Chr1_0213699  0.01 -0.19 -0.27 -0.15 -0.25  0.00 
Ca21Chr1_0213758  0.00  0.05 -0.11  0.20 -0.24  0.05 
Ca21Chr1_0213817  0.43  0.27  0.01 -0.11  0.08 -0.30 
Ca21Chr1_0213876  0.13  0.11  0.27  0.35  0.19 -0.06 
Ca21Chr1_0213935  0.10  0.13 -0.12 -0.26 -0.55 -0.30 
Ca21Chr1_0213994  0.01  0.07  0.18  0.29 -0.05 -0.19 
Ca21Chr1_0214053  0.04 -0.33  0.08  0.18  0.00 -0.10 
Ca21Chr1_0214112  0.63 -0.02  0.28  0.19 -0.11 -0.10 
Ca21Chr1_0214171  0.03 -0.21  0.03  0.05 -0.15 -0.41 
Ca21Chr1_0214230 -0.06 -0.35 -0.06  0.55  0.52  0.14 
Ca21Chr1_0214289  0.08 -0.12 -0.00 -0.03 -0.07  0.02 
Ca21Chr1_0214348 -0.20  0.34 -0.07  0.43  0.15  0.12 
Ca21Chr1_0214407  0.24 -0.09  0.38  0.35  0.20 -0.10 
Ca21Chr1_0214466  0.09 -0.11  0.11  0.28  0.13 -0.13 
Ca21Chr1_0214525 -0.30 -0.30 -0.68  0.09 -0.03 -0.30 
Ca21Chr1_0214584 -0.60 -0.74 -0.56  0.20  0.01 -0.15 
Ca21Chr1_0214643 -1.46 -0.45 -0.76  0.26 -0.77  0.10 
Ca21Chr1_0214702 -0.96 -0.70 -0.81  0.21  0.76 -0.22 
Ca21Chr1_0214761 -1.31 -0.82 -1.19 -0.56  0.01 -0.44 
Ca21Chr1_0214820 -1.42 -1.33 -0.85 -0.37 -0.07 -0.03 
Ca21Chr1_0214879 -1.49 -1.22 -1.16  0.63 -0.33  0.07 
Ca21Chr1_0214938 -0.36 -0.77 -1.12  0.16  0.32  0.24 
Ca21Chr1_0214997 -0.50 -0.70 -0.67  0.14  0.09  0.46 
Ca21Chr1_0215056 -0.23 -0.24 -0.71  0.19  0.14  0.04 
Ca21Chr1_0215115 -0.00 -0.87 -0.48  0.02  0.06  0.28 
Ca21Chr1_0215174  0.13 -0.17 -0.11 -0.13 -0.07  0.98 
Ca21Chr1_0215233  0.11 -0.05  0.11  0.04  0.09  0.06 
Ca21Chr1_0215292  0.37  0.12  0.07  0.15  0.13 -0.09 
Ca21Chr1_0215351  0.22  0.03  0.00  0.07  0.12  0.03 
Ca21Chr1_0215410  0.10  0.11  0.21 -0.13  0.07 -0.14 
Ca21Chr1_0215469  0.14  0.05  0.05 -0.20  0.02 -0.27 
Ca21Chr1_0215528  0.13  0.13  0.12  0.26 -0.05 -0.13 
Ca21Chr1_0215587 -0.44  0.21  0.43  0.20 -0.46 -0.44 
Ca21Chr1_0215646  0.03  0.20  0.39 -0.09  0.22 -0.39 
Ca21Chr1_0215705 -0.07  0.16  0.30 -0.19  0.20 -0.36 
Ca21Chr1_0215764  0.10 -0.06  0.22 -0.26  0.10  0.47 
Ca21Chr1_0215823  0.28  0.43  0.48  0.16  0.11 -0.31 
Ca21Chr1_0215882  0.11  0.35 -0.02 -0.10  0.08 -0.04 
Ca21Chr1_0215941 -0.21  0.20  0.27  0.09 -0.01 -0.24 
Ca21Chr1_0216000 -0.03  0.26 -0.01  0.06  0.15 -0.32 
Ca21Chr1_0216059 -0.26  0.00 -0.12 -0.72  0.25  0.30 
Ca21Chr1_0216118  0.65  0.10  0.19 -0.26 -0.02 -0.36 
Ca21Chr1_0216177  0.17  0.25  0.14 -0.20  0.33 -0.11 
Ca21Chr1_0216236 -0.08  0.33  0.14  0.40  0.06 -0.12 
Ca21Chr1_0216295  0.34  0.27  0.45  0.21 -0.04 -0.18 
Ca21Chr1_0216354  0.32  0.03 -0.05  0.10  0.09 -0.11 
Ca21Chr1_0216413  0.06  0.23  0.52  0.02 -0.17 -0.00 
Ca21Chr1_0216472  0.34  0.22  0.05 -0.14 -0.04 -0.15 
Ca21Chr1_0216531  0.80  0.14  0.07 -0.03 -0.25 -0.37 
Ca21Chr1_0216590  0.18  0.20  0.40  0.03 -0.18 -0.50 
Ca21Chr1_0216649  0.33  0.14  0.22 -0.10 -0.00 -0.26 
Ca21Chr1_0216708  0.09  0.46  0.33  0.16  0.08 -0.35 
Ca21Chr1_0216767  0.02  0.08  0.01 -0.07 -0.56  0.08 
Ca21Chr1_0216826  0.36 -0.01  0.38  0.10  0.22 -0.37 
Ca21Chr1_0216885  0.15  0.11  0.11  0.19  0.12 -0.05 
Ca21Chr1_0216944  0.21  0.06  0.59  0.14  0.15  0.05 
Ca21Chr1_0217003  0.19  0.13  0.48 -0.17  0.04 -0.05 
Ca21Chr1_0217062  0.21  0.19  0.41  0.18 -0.02  0.07 
Ca21Chr1_0217121  0.27 -0.07  0.35  0.35  0.16 -0.15 
Ca21Chr1_0217180  0.23  0.27  0.33  0.24  0.34  0.04 
Ca21Chr1_0217239  0.33  0.35  0.26 -0.73 -0.08  0.02 
Ca21Chr1_0217298  0.33  0.38  0.27  0.08 -0.08 -0.43 
Ca21Chr1_0217357 -0.09  0.17  0.47 -0.05  0.14  0.01 
Ca21Chr1_0217416  0.38  0.06  0.25 -0.10  0.36  0.10 
Ca21Chr1_0217475  0.31  0.06  1.30  0.18  0.37  0.48 
Ca21Chr1_0217534  0.20  0.46  0.62 -0.04  0.11 -0.06 
Ca21Chr1_0217593  0.40  0.49  0.48 -0.08  0.11 -0.22 
Ca21Chr1_0217652  0.25 -0.00  0.51 -0.32 -0.23 -0.12 
Ca21Chr1_0217711  0.63  0.20  0.68 -0.78 -0.14 -0.08 
Ca21Chr1_0217770  0.11  0.15  0.27 -0.17  0.03 -0.20 
Ca21Chr1_0217829  0.14  0.11  0.62 -0.13 -0.32 -0.68 
Ca21Chr1_0217888  0.02  0.31  0.41  0.06 -0.12  0.26 
Ca21Chr1_0217947  0.13 -0.12 -0.25 -0.11 -0.09  0.10 
Ca21Chr1_0218006  0.44  0.40  0.55  0.15  0.08  0.27 
Ca21Chr1_0218065  0.22  0.27  0.07 -0.23 -0.06  0.24 
Ca21Chr1_0218124  0.28  0.12  0.36  0.06  0.31 -0.13 
Ca21Chr1_0218183 -0.36  0.56  0.38  0.36  0.65 -0.02 
Ca21Chr1_0218242  0.81  0.10  0.25 -0.09  0.26 -0.29 
Ca21Chr1_0218301  0.33  0.22  0.62  0.12  0.00 -0.21 
Ca21Chr1_0218360  0.15  0.11  0.56 -0.02  0.01  0.23 
Ca21Chr1_0218419  0.14 -0.15 -0.31  0.37 -0.16  0.46 
Ca21Chr1_0218478 -0.08 -0.17 -0.30  0.23 -0.22  0.92 
Ca21Chr1_0218537 -0.20  0.38  0.09  0.11 -0.14  0.02 
Ca21Chr1_0218596  0.05  0.11 -0.02  0.23 -0.14  0.01 
Ca21Chr1_0218655 -0.14  0.22  0.02  0.08 -0.13 -0.17 
Ca21Chr1_0218714  0.07  0.15  0.21 -0.55 -0.76  0.36 
Ca21Chr1_0218773  0.79  0.34  0.26 -0.11  0.01  0.02 
Ca21Chr1_0218832  0.33  0.22  0.41  0.15  0.06  0.14 
Ca21Chr1_0218891  0.32  0.22  0.57 -0.05 -0.13  0.05 
Ca21Chr1_0218950  0.16  0.02  0.29  0.19  0.19  0.04 
Ca21Chr1_0219009  0.12 -0.02  0.51  0.22  0.22 -0.04 
Ca21Chr1_0219068 -0.04  0.46  0.61  0.29 -0.19 -0.10 
Ca21Chr1_0219127  0.00  0.34  0.53  0.35 -0.14  0.02 
Ca21Chr1_0219186  0.14 -0.08  0.21  0.27 -0.24 -0.33 
Ca21Chr1_0219245  0.12  0.20 -0.14 -0.16  0.34 -0.92 
Ca21Chr1_0219304 -0.47  0.19  0.62 -0.51 -0.12  0.19 
Ca21Chr1_0219363  0.05  0.17  0.25  0.29 -0.30  0.07 
Ca21Chr1_0219422  0.34  0.44  0.40  0.23 -0.01  0.16 
Ca21Chr1_0219481  0.17  0.07  0.23 -0.02 -0.20 -0.20 
Ca21Chr1_0219540  0.04  0.32  0.27 -0.36 -0.33 -0.04 
Ca21Chr1_0219599 -0.10 -0.01  0.13  0.24 -0.30 -0.05 
Ca21Chr1_0219658 -0.13  0.08  0.11 -0.38  0.39  0.40 
Ca21Chr1_0219717 -0.07 -0.10  0.40  0.04  0.08 -0.21 
Ca21Chr1_0219776 -0.25  0.16  0.25 -0.57 -0.19 -0.01 
Ca21Chr1_0219835 -0.05  0.70  0.06  0.08 -0.03  0.03 
Ca21Chr1_0219894  0.04 -0.18  0.32  0.02 -0.17 -0.06 
Ca21Chr1_0219953  0.21 -0.10  0.32 -0.03 -0.31  0.27 
Ca21Chr1_0220012 -0.17  0.16  0.37  0.04  0.08  0.09 
Ca21Chr1_0220071  0.54  0.45  0.46  0.01  0.04 -0.08 
Ca21Chr1_0220130 -0.23  0.27  0.51 -0.04  0.29 -0.35 
Ca21Chr1_0220189  0.37  0.11  0.18  0.06  0.06  0.14 
Ca21Chr1_0220248 -0.01  0.12  0.36 -0.12  0.12  0.02 
Ca21Chr1_0220307 -0.03  0.10  0.29 -0.26 -0.11 -0.17 
Ca21Chr1_0220366  0.42 -0.37 -0.07  0.37 -0.49  0.28  orf19.3298|
Ca21Chr1_0220425  0.24 -0.00 -0.11 -0.09 -0.17 -0.32 
Ca21Chr1_0220484  1.10 -0.35  0.02  0.54 -0.04  1.16 
Ca21Chr1_0220543  1.17 -0.10 -0.15 -0.34  0.03 -0.75  |orf19.3300
Ca21Chr1_0220602  0.08 -0.08  0.06 -0.14 -0.42  0.07 
Ca21Chr1_0220661  0.75 -0.11  0.34 -0.15 -0.04  0.16 
Ca21Chr1_0220720 -0.32  0.11  0.29  0.17  0.23  0.60 
Ca21Chr1_0220779 -0.55  0.33 -0.19  0.24 -0.12 -0.12 
Ca21Chr1_0220838 -0.69 -0.16 -0.93  0.85  0.05  0.24 
Ca21Chr1_0220897  0.10  0.15  0.00  0.20 -0.25  0.45 
Ca21Chr1_0220956  0.25 -0.13  0.13  0.09  0.37 -0.56 
Ca21Chr1_0221015  0.40 -0.35  0.20  0.63 -0.14 -0.36 
Ca21Chr1_0221074  0.42  0.09  0.03 -0.06  0.10 -0.07 
Ca21Chr1_0221133  0.08  0.02 -0.30  0.10  0.26  0.17 
Ca21Chr1_0221192 -0.30  0.70  0.82  0.37  0.65  0.05 
Ca21Chr1_0221251  0.37  0.10  0.44 -0.32  0.72  0.11 
Ca21Chr1_0221310  0.37 -0.16  0.33  0.13  0.24  0.10 
Ca21Chr1_0221369  0.40  0.30  0.59  0.29  0.01  0.05 
Ca21Chr1_0221428  0.48  0.46  0.46  0.04 -0.27  0.29 
Ca21Chr1_0221487  0.41 -0.24  0.25  0.41  0.13  0.25 
Ca21Chr1_0221546  0.10  0.14  0.18  0.16  0.42 -0.20 
Ca21Chr1_0221605  0.15  0.06 -0.16 -0.14  0.65  0.11 
Ca21Chr1_0221664  0.53  0.09  0.17 -0.18  0.02  0.42 
Ca21Chr1_0221723  0.42 -0.09  0.13 -0.25  0.24  0.26 
Ca21Chr1_0221782  0.35 -0.04  0.07  0.01  0.08 -0.39 
Ca21Chr1_0221841  0.54  0.16 -0.04  0.02  0.31  0.13 
Ca21Chr1_0221900 -0.36  0.20  0.58 -0.25  0.54  0.13 
Ca21Chr1_0221959  0.26  0.26 -0.22 -0.57  0.04  0.14 
Ca21Chr1_0222018 -0.17  0.17 -0.43 -0.17 -0.08  0.14  <orf19.3300
Ca21Chr1_0222077 -0.36  0.06 -0.65  0.31  0.39  0.31 
Ca21Chr1_0222136  0.27 -0.25 -1.10 -0.01  0.10 -0.01 
Ca21Chr1_0222195  0.18  0.39 -0.47  0.09  0.00  0.23 
Ca21Chr1_0222254 -0.50  0.38 -0.37 -0.36 -0.10  0.31 
Ca21Chr1_0222313 -0.95  0.31 -0.21 -0.71 -0.12  0.33 
Ca21Chr1_0222372  0.37  0.02 -1.19 -0.89 -0.28  1.07 
Ca21Chr1_0222431 -0.72 -0.03 -0.22 -0.53 -0.01  0.39 
Ca21Chr1_0222490 -0.81  0.17 -0.18 -0.62 -0.44  0.07 
Ca21Chr1_0222549 -0.60 -0.36 -0.37 -0.75 -0.19  0.29 
Ca21Chr1_0222608 -0.65 -0.40 -0.04 -0.56 -0.56  0.14 
Ca21Chr1_0222667 -1.20 -0.66  0.01 -0.91 -0.11 -0.33 
Ca21Chr1_0222726 -1.45  0.42 -0.37 -0.10 -0.24  0.19 
Ca21Chr1_0222785 -1.70 -0.81 -0.05 -0.11  0.08  0.39 
Ca21Chr1_0222844 -1.90 -0.93 -0.84 -0.15 -0.27  0.35 
Ca21Chr1_0222903 -1.92 -1.17 -0.24  0.14 -0.11  0.15 
Ca21Chr1_0222962 -1.80 -1.35 -0.93 -0.08 -0.07 -0.10 
Ca21Chr1_0223021 -1.59 -1.02 -1.05  0.16 -0.28 -0.22 
Ca21Chr1_0223080 -1.26 -1.12 -0.92 -0.03 -0.26  0.58 
Ca21Chr1_0223139  0.29 -0.78 -0.02  0.06  0.95  0.21 
Ca21Chr1_0223198 -0.69 -0.35 -0.54  0.14 -0.25  0.17 
Ca21Chr1_0223257 -0.28 -0.47 -0.63 -0.17 -0.33  0.07 
Ca21Chr1_0223316 -0.46 -0.11 -0.41  0.19 -0.24  0.40 
Ca21Chr1_0223375 -0.83 -0.20 -0.17  0.26 -0.15 -0.09 
Ca21Chr1_0223434 -0.43 -0.44 -0.68  0.16 -0.24 -0.26 
Ca21Chr1_0223493 -0.30  0.29 -0.59  0.28  0.10 -0.06 
Ca21Chr1_0223552 -0.28 -0.22  0.39 -0.06 -0.05  0.23 
Ca21Chr1_0223611 -0.64 -0.22 -0.70 -0.38 -0.32 -0.61 
Ca21Chr1_0223670 -0.67  0.04 -0.82 -0.13 -0.14 -0.27 
Ca21Chr1_0223729 -0.08 -0.43 -0.64 -0.29 -0.32  0.33 
Ca21Chr1_0223788 -0.21  0.20 -0.44 -0.16 -0.01 -0.10 
Ca21Chr1_0223847 -0.30 -0.10 -0.51 -0.65 -0.79  0.05 
Ca21Chr1_0223906 -0.12  0.13 -0.33 -0.45 -0.23  0.00 
Ca21Chr1_0223965  0.00 -0.37 -0.24 -0.16 -0.49  0.09 
Ca21Chr1_0224024 -1.18 -0.52 -1.56 -0.50 -0.59  0.11 
Ca21Chr1_0224083 -1.25 -0.37 -1.37 -0.53 -0.27 -0.12 
Ca21Chr1_0224142 -1.17 -0.81 -1.37 -0.00 -0.31  0.03 
Ca21Chr1_0224201 -1.66 -1.63 -1.46 -0.24  0.03 -0.46 
Ca21Chr1_0224260 -1.19 -1.09 -1.48 -0.29 -0.25 -0.31 
Ca21Chr1_0224319 -0.00 -1.16 -1.30 -0.02 -0.10 -0.81 
Ca21Chr1_0224378 -0.48 -0.38 -0.24  0.14  0.09 -0.45 
Ca21Chr1_0224437 -0.01 -0.19 -0.45 -0.27 -0.12  1.81 
Ca21Chr1_0224496  0.18 -0.38  0.14 -0.22  0.16 -0.36 
Ca21Chr1_0224555  0.18 -0.20  0.42 -0.17 -0.03 -0.09 
Ca21Chr1_0224614 -0.26  0.03  0.01 -0.15 -0.40  0.13 
Ca21Chr1_0224673 -0.07  0.08 -0.02 -0.24 -0.05 -0.07  orf19.3301>
Ca21Chr1_0224732  0.13  0.03 -0.18 -0.23 -0.31  0.20 
Ca21Chr1_0224791 -0.26  0.15  0.31  0.13  0.18 -0.35 
Ca21Chr1_0224850  0.16  0.02  0.08 -0.46  0.18  0.03 
Ca21Chr1_0224909  0.15 -0.02  0.17 -0.04  0.05  0.12 
Ca21Chr1_0224968  0.37  0.00  0.11 -0.12  0.21 -0.18 
Ca21Chr1_0225027  0.12 -0.07  0.02 -0.21  0.10  0.28 
Ca21Chr1_0225086 -0.13  0.17 -0.13 -0.07  0.05 -0.17 
Ca21Chr1_0225145 -0.37  0.13 -0.18  0.14  0.17  0.04 
Ca21Chr1_0225204  0.11  0.04  0.04 -0.42 -0.11 -0.23 
Ca21Chr1_0225263 -0.03  0.18  0.10 -0.44  0.03 -0.64 
Ca21Chr1_0225322  0.46  0.25  0.30 -0.36  0.31 -0.05 
Ca21Chr1_0225381  0.38  0.17  0.10 -0.03 -0.29  0.12 
Ca21Chr1_0225440  0.28 -0.06  0.13 -0.21  0.22 -0.24 
Ca21Chr1_0225499  0.21 -0.12  0.10 -0.19 -0.18 -0.42 
Ca21Chr1_0225558 -0.21  0.05  0.65 -0.28  0.66  0.34 
Ca21Chr1_0225617 -0.01  0.01  0.12 -0.05 -0.09  0.08 
Ca21Chr1_0225676 -0.10  0.05  0.08  0.15  0.94  0.12 
Ca21Chr1_0225735 -0.10  0.19  0.11 -0.18 -0.07  0.02 
Ca21Chr1_0225794  0.50  0.25 -0.00  0.19 -0.08  0.31 
Ca21Chr1_0225853 -0.34 -0.07 -0.27  0.06  0.06 -0.09 
Ca21Chr1_0225912 -0.50 -0.09 -0.52 -0.17 -0.25  0.49 
Ca21Chr1_0225971 -0.12  0.08 -0.43 -0.18  0.48 -0.47 
Ca21Chr1_0226030 -0.41  0.14 -0.46 -0.16  0.56 -0.00 
Ca21Chr1_0226089 -0.30 -0.03 -0.22 -0.19 -0.07 -0.04 
Ca21Chr1_0226148 -0.32 -0.24 -0.09 -0.09  0.05  0.13 
Ca21Chr1_0226207  1.05 -0.16  0.58 -0.17 -0.52  0.35 
Ca21Chr1_0226266 -0.23  0.23 -0.21 -0.01  0.07  0.17 
Ca21Chr1_0226325 -0.39  0.18  0.19 -0.35  0.05 -0.46 
Ca21Chr1_0226384 -0.02  0.09  0.50  0.43 -0.97 -0.04 
Ca21Chr1_0226443 -0.12  0.17  0.28  0.42  0.20 -0.30 
Ca21Chr1_0226502 -0.07  0.18 -0.13  0.01  0.01 -0.25 
Ca21Chr1_0226561 -0.04 -0.02 -0.04 -0.21 -0.20 -0.26 
Ca21Chr1_0226620 -0.10  0.01  0.04 -0.03 -0.27 -0.20 
Ca21Chr1_0226679 -0.17 -0.36 -0.16  0.07 -0.27 -0.34 
Ca21Chr1_0226738  0.46  0.27  0.33 -0.31 -0.01 -0.01 
Ca21Chr1_0226797  0.12 -0.09  0.29  0.00 -0.04  0.00 
Ca21Chr1_0226856  0.25  0.46  0.13 -0.15 -0.10  0.16  orf19.3301|
Ca21Chr1_0226915 -0.63  0.21  0.22  0.19 -0.23  0.44 
Ca21Chr1_0226974  1.16  0.06 -0.22  0.69 -0.43 -0.52 
Ca21Chr1_0227033  0.37  0.48  0.59 -0.15 -0.04 -0.63 
Ca21Chr1_0227092 -0.35  0.05  0.46 -0.15 -0.08  0.05 
Ca21Chr1_0227151  0.00  0.10  0.59 -0.15 -0.06 -0.05 
Ca21Chr1_0227210  0.13  0.29  0.21 -0.47 -0.16 -0.17 
Ca21Chr1_0227269  0.25  0.14  0.55  0.16 -0.48  0.11 
Ca21Chr1_0227328  0.09  0.19  0.20 -0.15 -0.14  0.10 
Ca21Chr1_0227387  0.53 -0.11  0.65 -0.89 -0.04  0.64 
Ca21Chr1_0227446  0.12  0.03  0.41 -0.06  0.08  0.03 
Ca21Chr1_0227505  0.44 -0.12  0.14  0.11 -0.17  0.52 
Ca21Chr1_0227564  0.03  0.17  0.37 -0.09 -0.16  0.18 
Ca21Chr1_0227623  0.06  0.03  0.50  0.04 -0.33  0.09 
Ca21Chr1_0227682  0.35  0.33  0.56 -0.40  0.28 -0.30  |orf19.3302
Ca21Chr1_0227741  0.57  0.38  0.59 -0.17 -0.13 -0.33 
Ca21Chr1_0227800  0.47  0.17  0.62 -0.31 -0.15 -0.18 
Ca21Chr1_0227859  0.11 -0.02  0.58 -0.04 -0.10 -0.24 
Ca21Chr1_0227918  0.25  0.32  0.65  0.04 -0.30 -0.27 
Ca21Chr1_0227977  0.22  0.06  0.30  0.06 -0.17  0.18 
Ca21Chr1_0228036  0.54  0.14  1.01  0.20 -0.04  0.19 
Ca21Chr1_0228095  0.37  0.10  0.58 -0.20  0.10  0.10 
Ca21Chr1_0228154  0.48  0.29  0.63  0.22 -0.33 -0.17 
Ca21Chr1_0228213  0.44  0.07  0.38 -0.41 -0.57  0.14 
Ca21Chr1_0228272  0.92  0.17  0.22 -0.10 -0.12  0.03 
Ca21Chr1_0228331 -0.12 -0.12  0.24 -0.16 -0.55 -0.21 
Ca21Chr1_0228390  0.44  0.42  0.44 -0.03  0.16 -0.34 
Ca21Chr1_0228449  0.26 -0.30  0.50 -0.02  0.06 -0.31 
Ca21Chr1_0228508  0.15  0.12  0.26 -0.27 -0.21  0.20 
Ca21Chr1_0228567  0.02  0.20  0.12  0.05 -0.21  0.17 
Ca21Chr1_0228626 -0.86 -0.08 -0.32  0.14 -0.26  0.13 
Ca21Chr1_0228685 -0.14 -0.07  0.29  0.05  0.67  0.08 
Ca21Chr1_0228744  0.24  0.04  0.27  0.17  0.00  0.06 
Ca21Chr1_0228803  0.05  0.35  0.16  0.06 -0.08 -0.23 
Ca21Chr1_0228862  1.00 -0.08  0.11  0.13 -0.11  0.03 
Ca21Chr1_0228921  0.20  0.09 -0.08  0.28  0.04 -0.01 
Ca21Chr1_0228980 -0.10 -0.08 -0.20  0.26 -0.16 -0.20 
Ca21Chr1_0229039  0.04 -0.14  0.17 -0.12 -0.06  0.08 
Ca21Chr1_0229098  0.43  0.04  0.11 -0.29  0.19 -0.29 
Ca21Chr1_0229157 -0.03 -0.07  0.24 -0.09 -0.42  0.38 
Ca21Chr1_0229216  0.16  0.01  0.38  0.19  0.24 -0.13 
Ca21Chr1_0229275  0.16  0.12  0.38 -0.07  0.06 -0.11 
Ca21Chr1_0229334  0.15  0.27 -0.19 -0.23 -0.52 -0.03 
Ca21Chr1_0229393  0.11 -0.26 -0.20 -0.07 -0.04 -0.17 
Ca21Chr1_0229452  0.02 -0.25 -0.29 -0.02 -0.16 -0.19 
Ca21Chr1_0229511 -0.04 -0.30 -0.06 -0.03 -0.22 -0.20 
Ca21Chr1_0229570  0.18  0.05  0.06 -0.02 -0.20 -0.09 
Ca21Chr1_0229629 -0.38  0.04  0.52 -0.64 -0.68  0.07 
Ca21Chr1_0229688  0.04  0.39  0.27 -0.03 -0.04  0.05 
Ca21Chr1_0229747  0.35  0.28  0.36 -0.17 -0.22 -0.19  <orf19.3302
Ca21Chr1_0229806 -0.07  0.13  0.10  0.10  0.05  0.65 
Ca21Chr1_0229865 -0.05  0.47  0.00  0.38  0.99 -0.51 
Ca21Chr1_0229924    NA    NA    NA    NA    NA    NA
Ca21Chr1_0229983  0.14  0.39 -0.93  0.07 -0.19  0.10 
Ca21Chr1_0230042  1.61 -0.15 -0.05 -0.48  0.63 -0.51 
Ca21Chr1_0230101 -0.20 -0.16 -0.71 -0.34  1.17 -0.20 
Ca21Chr1_0230160  0.11 -0.06 -0.59 -0.63 -0.38  0.34 
Ca21Chr1_0230219  0.14  0.46 -0.11 -0.06 -0.25 -0.08 
Ca21Chr1_0230278  0.21 -0.11 -0.86 -0.07 -0.05 -0.27 
Ca21Chr1_0230337  1.37 -0.52 -1.09 -0.03  0.28 -0.38 
Ca21Chr1_0230396  0.89 -0.62 -0.76 -0.43 -0.30 -0.83 
Ca21Chr1_0230455 -0.11 -0.33 -0.91 -0.38 -0.39 -0.33 
Ca21Chr1_0230514 -0.78 -0.54 -1.82 -1.25 -0.03 -0.94 
Ca21Chr1_0230573 -0.78 -1.31 -1.80 -1.29 -0.39 -0.83 
Ca21Chr1_0230632 -1.32 -0.68 -1.89 -1.32 -0.16 -0.58 
Ca21Chr1_0230691 -1.14 -1.37 -1.76 -0.98  0.28  0.18 
Ca21Chr1_0230750 -0.45 -0.95 -1.54 -1.02 -0.60  0.25 
Ca21Chr1_0230809  0.04 -1.05 -0.44 -0.20 -0.13 -0.02 
Ca21Chr1_0230868 -0.49 -0.68 -0.83 -0.48  0.19 -0.37 
Ca21Chr1_0230927 -0.84 -1.26 -1.90 -0.65 -0.09  0.07 
Ca21Chr1_0230986 -0.25 -1.39 -1.98 -0.58 -0.32  0.08 
Ca21Chr1_0231045 -1.05 -1.73 -1.17 -0.63 -0.24 -0.01 
Ca21Chr1_0231104 -1.86 -1.86 -1.86 -1.26 -0.52  0.07 
Ca21Chr1_0231163 -1.70 -1.64 -2.00 -0.95 -0.15 -0.17 
Ca21Chr1_0231222 -0.97 -2.01 -2.49 -1.28 -0.67 -0.34 
Ca21Chr1_0231281 -2.07 -2.91 -2.37 -2.13 -0.66 -0.21 
Ca21Chr1_0231340 -2.00 -2.40 -1.77 -1.87 -0.84 -0.39 
Ca21Chr1_0231399 -2.50 -1.33 -2.86 -2.15 -1.39 -0.92 
Ca21Chr1_0231458 -2.46 -2.27 -2.34 -2.61 -2.56 -1.14 
Ca21Chr1_0231517 -1.94 -1.89 -2.13 -3.08 -2.47 -1.43 
Ca21Chr1_0231576 -2.40 -2.12 -1.95 -3.95 -2.39 -1.75 
Ca21Chr1_0231635 -0.17 -2.18 -1.73 -2.05 -2.52 -1.55 
Ca21Chr1_0231694 -2.15 -1.49 -1.76 -3.16 -2.33 -1.48 
Ca21Chr1_0231753 -1.20 -1.04 -1.97 -1.16 -1.96 -1.11 
Ca21Chr1_0231812 -1.72 -1.71 -1.65 -1.65 -1.16 -0.64 
Ca21Chr1_0231871 -1.73 -1.58 -1.94 -1.11 -1.17 -0.86 
Ca21Chr1_0231930 -1.81 -1.57 -2.00 -1.34 -0.84 -0.30 
Ca21Chr1_0231989 -1.43 -1.29 -1.59 -1.54 -1.04 -0.33 
Ca21Chr1_0232048 -1.01 -1.53 -1.71 -1.40 -0.56  0.26 
Ca21Chr1_0232107 -0.77 -1.11 -0.89 -1.12 -0.80 -0.32 
Ca21Chr1_0232166 -0.53 -0.57 -0.83 -0.84 -0.42 -0.13 
Ca21Chr1_0232225 -0.47 -0.85 -0.73 -0.92 -0.27 -0.15 
Ca21Chr1_0232284  0.45 -0.47  0.05 -0.58 -0.53  0.22 
Ca21Chr1_0232343 -0.64 -0.48 -0.53  0.26 -0.30 -0.68 
Ca21Chr1_0232402 -0.50 -0.37 -0.31 -0.64 -0.10 -0.22 
Ca21Chr1_0232461 -0.42 -0.02 -0.59 -0.71 -0.03 -0.42 
Ca21Chr1_0232520 -0.22 -0.23 -0.28 -0.22 -0.75 -0.21 
Ca21Chr1_0232579 -0.34 -0.08 -0.10 -0.12 -0.08  0.08 
Ca21Chr1_0232638 -0.20 -0.42 -0.59 -0.26 -0.09  0.09 
Ca21Chr1_0232697 -0.23 -0.45 -0.59 -0.01  0.08  0.11 
Ca21Chr1_0232756 -0.11 -0.02 -0.67 -0.38 -0.21  0.19 
Ca21Chr1_0232815  0.78 -0.09 -0.39 -0.19  0.42 -0.09  |orf19.3303
Ca21Chr1_0232874 -0.39  0.25 -0.54 -0.36 -0.49 -0.14 
Ca21Chr1_0232933  0.11 -0.13  0.30 -0.09 -0.29 -0.22 
Ca21Chr1_0232992  0.14 -0.04  0.31 -0.28 -0.22  0.25 
Ca21Chr1_0233051 -0.14 -0.13  0.53 -0.29 -0.14 -0.06 
Ca21Chr1_0233110  0.48  0.20  0.28 -0.20 -1.06 -0.17 
Ca21Chr1_0233169 -0.05  0.03  0.64  0.47 -0.38  0.01 
Ca21Chr1_0233228  0.17 -0.22  0.45 -0.20 -0.16 -0.21 
Ca21Chr1_0233287  0.07 -0.05  0.33 -0.04 -0.57 -0.31 
Ca21Chr1_0233346  0.08  0.13  0.33 -0.26 -0.31 -0.27 
Ca21Chr1_0233405  0.14 -0.50  0.58 -0.19 -0.28 -0.12 
Ca21Chr1_0233464  0.14  0.16  0.47  0.23 -0.19 -0.06 
Ca21Chr1_0233523  1.24  0.12  0.66 -0.13 -0.05 -0.28 
Ca21Chr1_0233582  0.19  0.54  0.31 -0.10  0.45 -0.12 
Ca21Chr1_0233641  0.67  0.13  0.55 -0.32 -0.22  0.02 
Ca21Chr1_0233700  0.35  0.08  0.30 -0.24 -0.06 -0.30 
Ca21Chr1_0233759  0.11  0.33  0.71 -0.13 -0.05  0.01 
Ca21Chr1_0233818  0.18 -0.27  0.56 -0.06 -0.27  0.05 
Ca21Chr1_0233877  0.06  0.56  0.44 -0.09 -0.75 -0.32 
Ca21Chr1_0233936  0.18  0.04  0.30 -0.01 -0.45 -0.12 
Ca21Chr1_0233995  0.09 -0.09  0.49 -0.29 -0.82 -0.08 
Ca21Chr1_0234054  0.09 -0.18  0.26 -0.03 -0.31  0.05 
Ca21Chr1_0234113  0.13 -0.36  0.16 -0.08 -0.66  0.50 
Ca21Chr1_0234172  0.17 -0.38  0.26  0.18  0.02  0.18 
Ca21Chr1_0234231 -0.21 -0.17 -0.20  0.15  0.06  0.23 
Ca21Chr1_0234290 -0.07 -0.04  0.33  0.03 -0.58  0.23 
Ca21Chr1_0234349 -0.25 -0.37 -0.30 -0.26 -0.04 -0.22 
Ca21Chr1_0234408 -0.24 -0.09 -0.07  0.17 -0.18  0.53 
Ca21Chr1_0234467  0.03  0.77  0.24  0.28  0.01 -0.05 
Ca21Chr1_0234526  0.08  0.03 -0.00  0.06 -0.02 -0.11 
Ca21Chr1_0234585  0.04  0.01  0.09  0.31  0.24 -0.13 
Ca21Chr1_0234644 -0.45  0.24  0.22 -0.19 -0.06 -0.44 
Ca21Chr1_0234703  0.46  0.10  0.42  0.15  0.43  0.27 
Ca21Chr1_0234762  0.58  0.14  0.42 -0.13  0.20 -0.09 
Ca21Chr1_0234821  0.23  0.32  0.10  0.03  0.25  0.17 
Ca21Chr1_0234880  0.11 -0.03 -0.25 -0.36  0.08  0.39 
Ca21Chr1_0234939  0.00  0.09  0.48  0.13  0.36  0.76  <orf19.3303
Ca21Chr1_0234998 -0.22  0.56 -0.10 -0.21  0.22  0.75 
Ca21Chr1_0235057  0.66  0.30  0.23 -0.30 -0.50  0.66  |orf19.3304
Ca21Chr1_0235116  0.28 -0.21  0.04 -0.07  0.40  0.14 
Ca21Chr1_0235175 -0.11 -0.00  0.05 -0.09 -0.20  0.19 
Ca21Chr1_0235234 -0.12 -0.09  0.14  0.13 -0.09  0.67 
Ca21Chr1_0235293  0.09  0.07  0.21 -0.16  0.14  0.40 
Ca21Chr1_0235352 -0.25  0.25  0.03 -0.45 -0.10  0.65 
Ca21Chr1_0235411 -0.21  0.08  0.35  0.16 -0.20 -0.03 
Ca21Chr1_0235470  0.17  0.10  0.37  0.19  0.13 -0.08 
Ca21Chr1_0235529  0.51  0.05  0.08 -0.08 -0.15 -0.01 
Ca21Chr1_0235588 -0.30  0.11  0.15 -0.16 -0.18  0.72 
Ca21Chr1_0235647  0.14  0.16  0.14  0.20 -0.15  0.52 
Ca21Chr1_0235706  0.07  0.08  0.19 -0.02 -0.16  0.13 
Ca21Chr1_0235765  0.11  0.20 -0.07  0.13 -0.28 -0.02 
Ca21Chr1_0235824  0.06  0.17  0.13  0.41 -0.38  0.02 
Ca21Chr1_0235883  0.18  0.88  0.03 -0.32  0.12  0.01  <orf19.3304
Ca21Chr1_0235942 -0.33  0.08  0.75  0.06 -0.14  0.68 
Ca21Chr1_0236001 -0.12 -0.11  0.19  0.26 -0.23  0.11 
Ca21Chr1_0236060  0.02 -0.16  0.35 -0.18 -0.10  0.15  |orf19.3305
Ca21Chr1_0236119  0.24  0.12  0.28  0.59  0.17  0.38 
Ca21Chr1_0236178  0.06  0.18  0.24 -0.56 -0.07  0.16 
Ca21Chr1_0236237  0.08  0.59  0.42  0.16 -0.44 -0.31 
Ca21Chr1_0236296 -0.08 -0.55  0.39 -0.14 -0.36 -0.24 
Ca21Chr1_0236355  0.15  0.03  0.14 -0.17 -0.13  0.56 
Ca21Chr1_0236414  0.33 -0.02  0.22  0.40  0.18 -0.07 
Ca21Chr1_0236473  0.10 -0.06  0.28  0.55  0.28  0.17 
Ca21Chr1_0236532  0.03 -0.06 -0.12 -0.02  0.10  0.24 
Ca21Chr1_0236591  0.27  0.21  0.12  0.11  0.11 -0.22 
Ca21Chr1_0236650  0.17 -0.03 -0.16  0.07 -0.18 -0.20 
Ca21Chr1_0236709  0.27  0.14  0.10  0.05  0.10  0.11 
Ca21Chr1_0236768 -0.20  0.12  0.19 -0.01 -0.42 -0.32 
Ca21Chr1_0236827  0.14  0.08  0.01  0.02  0.05  0.04 
Ca21Chr1_0236886 -0.18  0.65 -0.09 -0.05  0.03  0.13 
Ca21Chr1_0236945 -0.02 -0.08 -0.05  0.39 -0.02 -0.09 
Ca21Chr1_0237004  0.07 -0.21  0.03  0.43 -0.00 -0.55 
Ca21Chr1_0237063  0.33 -0.11 -0.00 -0.22 -0.09  0.24 
Ca21Chr1_0237122  0.24 -0.15  0.51 -0.05  0.13 -0.52 
Ca21Chr1_0237181  0.15  0.03  0.21  0.11 -0.19 -0.28 
Ca21Chr1_0237240 -0.15  0.02  0.24  0.55 -0.01 -0.32 
Ca21Chr1_0237299  0.29  0.08  0.29  0.54  0.12 -0.25 
Ca21Chr1_0237358  0.08  0.16  0.36  0.37  0.07 -0.10 
Ca21Chr1_0237417  0.34 -0.01 -0.02  0.48 -0.18 -0.25 
Ca21Chr1_0237476  0.17 -0.20 -0.03 -0.07 -0.00 -0.02 
Ca21Chr1_0237535 -0.00  0.20  0.18  0.07  0.25  0.25 
Ca21Chr1_0237594 -0.01 -0.03  0.03 -0.05 -0.04 -0.26 
Ca21Chr1_0237653  0.19  0.15  0.40 -0.29  0.20 -0.50 
Ca21Chr1_0237712  0.29  0.16  0.30 -0.08  0.18 -0.03 
Ca21Chr1_0237771  0.14 -0.16  0.26 -0.18  0.14 -0.05 
Ca21Chr1_0237830 -0.47 -0.38 -0.25  0.09 -0.48 -0.25 
Ca21Chr1_0237889 -0.81 -0.28 -0.71  0.32 -0.09 -0.23 
Ca21Chr1_0237948 -0.68 -0.54 -0.30 -0.07  0.00 -0.14 
Ca21Chr1_0238007 -0.31 -0.36 -0.33 -0.21  0.06 -0.27 
Ca21Chr1_0238066 -0.14 -0.63  0.01  0.18 -0.07 -0.18 
Ca21Chr1_0238125 -0.14 -0.08  0.24 -0.29  0.13 -0.49 
Ca21Chr1_0238184  0.08 -0.11  0.23  0.21 -0.12 -0.37 
Ca21Chr1_0238243  0.31  0.17 -0.04  0.11  0.04 -0.15 
Ca21Chr1_0238302 -0.09  0.15  0.35 -0.03 -0.23 -0.21  <orf19.3305
Ca21Chr1_0238361 -0.06 -0.28 -0.33 -0.17  0.03  0.47 
Ca21Chr1_0238420  0.02  0.08  0.08  0.37 -0.06  0.22 
Ca21Chr1_0238479  0.13 -0.06  0.33  0.05 -0.09  1.04 
Ca21Chr1_0238538  0.37  0.06 -0.43 -0.08 -0.20  0.77 
Ca21Chr1_0238597  0.33 -0.06  0.41 -0.61  0.59  0.83 
Ca21Chr1_0238656 -0.02 -0.22  0.13  0.04 -0.04  0.53 
Ca21Chr1_0238715  0.10 -0.06 -0.42  0.49 -0.21  0.28 
Ca21Chr1_0238774  1.02  0.16  0.11 -0.34  0.18  0.40 
Ca21Chr1_0238833  0.41 -0.05 -0.01  0.31  0.10  0.35 
Ca21Chr1_0238892  0.04  0.06  0.18  0.10  0.04  0.41 
Ca21Chr1_0238951  0.29 -0.20  0.24 -0.07 -0.00  0.19 
Ca21Chr1_0239010  0.02  0.15  0.59  0.23  0.01  0.34 
Ca21Chr1_0239069  0.34  0.02 -0.01 -0.08 -0.73  0.06  |orf19.3306
Ca21Chr1_0239128  0.62  0.38  0.96 -0.02  0.18 -0.08 
Ca21Chr1_0239187 -0.18  0.18  0.15  0.24  0.28  0.01 
Ca21Chr1_0239246  0.19  0.41  0.41  0.28 -0.08 -0.10 
Ca21Chr1_0239305  0.33  0.19  0.50  0.06  0.20 -0.22 
Ca21Chr1_0239364  0.02 -0.02  0.49  0.12 -0.01 -0.51 
Ca21Chr1_0239423  0.05  0.28  0.33  0.08  0.09 -0.12 
Ca21Chr1_0239482  0.69 -0.01  0.32 -0.13  0.04 -0.12 
Ca21Chr1_0239541  0.25 -0.07  0.32  0.32  0.52 -0.42 
Ca21Chr1_0239600  0.63 -0.01  0.15  0.31 -0.36 -0.16 
Ca21Chr1_0239659  0.12  0.11 -0.03  0.23  0.11  0.01 
Ca21Chr1_0239718  0.26 -0.00  0.58 -0.05 -0.13 -0.14 
Ca21Chr1_0239777  0.33  0.53  0.14  0.04  0.56  0.40 
Ca21Chr1_0239836  0.13 -0.13  0.30 -0.02 -0.08 -0.12 
Ca21Chr1_0239895 -0.05 -0.06 -0.02 -0.38 -0.27  0.09 
Ca21Chr1_0239954  0.03 -0.08  1.16  0.21 -0.70  0.03 
Ca21Chr1_0240013  0.23  0.06  0.04 -0.25  0.01 -0.00 
Ca21Chr1_0240072  0.46  0.18  0.21  0.40 -0.18 -0.05 
Ca21Chr1_0240131  0.25 -0.10  0.00  0.03  0.13 -0.13 
Ca21Chr1_0240190  0.46 -0.04  0.25  0.01  0.08 -0.06 
Ca21Chr1_0240249 -0.21  0.09 -0.09 -0.03  0.16 -0.08 
Ca21Chr1_0240308 -0.05  0.17  0.28  0.41 -0.28 -0.20 
Ca21Chr1_0240367  0.11  0.33 -0.13  0.39  0.28 -0.19 
Ca21Chr1_0240426  0.29  0.06  0.43  0.36 -0.01 -0.23 
Ca21Chr1_0240485  0.43  0.04  0.17  0.29  0.06 -0.08 
Ca21Chr1_0240544 -0.40  0.02  0.21  0.11 -0.44 -0.12 
Ca21Chr1_0240603  0.22  0.20  0.59 -0.17 -0.16  0.11 
Ca21Chr1_0240662 -0.08  0.08  0.23 -0.11 -0.20 -0.01 
Ca21Chr1_0240721 -0.11  0.11  0.34  0.19 -0.15  0.18 
Ca21Chr1_0240780  0.15  0.14  0.31  0.75  0.33 -0.04 
Ca21Chr1_0240839  0.61  0.17  0.46  0.65  0.14  0.29 
Ca21Chr1_0240898  0.86  0.15  0.54  0.03 -0.17  0.65  <orf19.3306
Ca21Chr1_0240957  0.23 -0.08  0.00  0.12  0.33  0.26 
Ca21Chr1_0241016  0.00 -0.02  0.31  0.15 -0.37  0.06 
Ca21Chr1_0241075  0.28 -0.04  0.14 -0.17  0.50 -0.91  |orf19.3307
Ca21Chr1_0241134 -0.80  0.35 -0.52 -0.33 -0.54 -0.04 
Ca21Chr1_0241193 -0.17 -0.08  0.21 -0.19 -0.43 -0.04 
Ca21Chr1_0241252 -0.45 -0.59 -0.19  0.23 -0.53 -0.09 
Ca21Chr1_0241311 -0.11  0.07 -0.10  0.05 -0.04  0.44 
Ca21Chr1_0241370 -0.22 -0.06 -0.58 -0.22 -0.01  0.62 
Ca21Chr1_0241429 -0.66 -0.64 -0.61 -0.27 -0.11  0.46 
Ca21Chr1_0241488 -0.58 -0.58 -0.83  0.03 -0.13  0.23 
Ca21Chr1_0241547  0.06 -0.58 -0.77 -0.01  0.22 -0.05 
Ca21Chr1_0241606 -0.30 -0.21 -0.41 -0.59 -0.09 -0.08 
Ca21Chr1_0241665 -0.34 -0.30  0.15  0.32 -0.34 -0.15 
Ca21Chr1_0241724 -0.14 -0.08  0.01  0.18 -0.26 -0.06 
Ca21Chr1_0241783  0.20 -0.13  0.28  0.03  0.09 -0.08 
Ca21Chr1_0241842 -0.04  0.49 -0.21  0.08  0.74 -0.49 
Ca21Chr1_0241901 -0.01  0.07  0.02 -0.07 -0.16 -0.19 
Ca21Chr1_0241960  0.12  0.40  0.29  0.09  0.15 -0.20 
Ca21Chr1_0242019 -0.92 -0.17  0.12 -0.08 -0.18 -0.27 
Ca21Chr1_0242078  0.11  0.17  0.02 -0.01 -0.14 -0.08 
Ca21Chr1_0242137 -0.09  0.17  0.19 -0.05 -0.14  0.25 
Ca21Chr1_0242196  0.06  0.23  0.06 -0.15 -0.08 -0.25 
Ca21Chr1_0242255  0.22  0.13  0.46  0.27 -0.08 -0.02 
Ca21Chr1_0242314  0.14  0.36  0.27  0.02  0.04 -0.28 
Ca21Chr1_0242373  0.07 -0.23  0.15  0.07 -0.15 -0.11 
Ca21Chr1_0242432  0.08  0.41  0.12  0.30 -0.03  0.05 
Ca21Chr1_0242491  0.19 -0.33 -0.11 -0.09  0.08 -0.68  <orf19.3307
Ca21Chr1_0242550 -0.04 -0.00 -0.07  0.17  0.06  0.06 
Ca21Chr1_0242609  0.03 -0.29 -0.09 -0.04  0.05  0.03 
Ca21Chr1_0242668 -0.25 -0.15 -0.19  0.10 -0.01 -0.15 
Ca21Chr1_0242727  0.19  0.15  0.18  0.23  0.01 -0.08  orf19.3308>
Ca21Chr1_0242786  0.65 -0.19 -0.12  0.03  0.08 -0.24 
Ca21Chr1_0242845 -0.04 -0.24  0.05 -0.09  0.33 -0.52 
Ca21Chr1_0242904  0.30  0.17  0.30 -0.05  0.24 -0.34 
Ca21Chr1_0242963  0.43  0.36  0.44 -0.20  0.56 -0.20 
Ca21Chr1_0243022  0.13 -0.01  0.36  0.22  0.03  0.02 
Ca21Chr1_0243081  0.32  0.24  0.37  0.07  0.09 -0.14 
Ca21Chr1_0243140  0.16  0.68  0.46  0.36  0.30 -0.10 
Ca21Chr1_0243199  0.62  0.22  0.74  0.42  0.03  0.24 
Ca21Chr1_0243258  0.12  0.22  0.39  0.06  0.14 -0.06 
Ca21Chr1_0243317 -0.16  0.28  0.27  0.23 -0.25  0.39 
Ca21Chr1_0243376  0.72  0.14 -0.02  0.06  0.02  0.09 
Ca21Chr1_0243435  0.03  0.25  0.46  0.40  0.00 -0.21 
Ca21Chr1_0243494  0.22  0.05  0.22 -0.21  0.01  0.34 
Ca21Chr1_0243553  0.06  0.29  0.23 -0.10  0.05  0.58 
Ca21Chr1_0243612 -0.06  0.16  0.28  0.24 -0.32  0.00 
Ca21Chr1_0243671  0.29  0.14  0.16  0.02  0.07 -0.23 
Ca21Chr1_0243730 -0.28  0.31  0.13  0.33  0.17 -0.41 
Ca21Chr1_0243789  0.17  0.28  0.35  0.38  0.15 -0.73 
Ca21Chr1_0243848 -0.01  0.02  0.26  0.43 -0.17 -0.08 
Ca21Chr1_0243907  0.19  0.08  0.24  0.51 -0.01 -0.33 
Ca21Chr1_0243966 -0.15  0.10  0.23  0.21  0.11 -0.25 
Ca21Chr1_0244025  0.00  0.03  0.06  0.22  0.23  0.16 
Ca21Chr1_0244084 -0.11  0.36  0.15 -0.11 -0.16 -0.14 
Ca21Chr1_0244143 -0.10  0.13  0.30  0.14  0.21 -0.16 
Ca21Chr1_0244202  0.11 -0.08  0.03 -0.22  0.19 -0.04 
Ca21Chr1_0244261  0.81  0.11  0.16 -0.16  0.17 -0.54 
Ca21Chr1_0244320  0.27  0.16  0.19 -0.06  0.08 -0.22 
Ca21Chr1_0244379  0.08 -0.11  0.27 -0.33 -0.03  0.62 
Ca21Chr1_0244438  0.09 -0.14  0.17  0.08  0.30 -0.31 
Ca21Chr1_0244497 -0.08  0.14  0.33  0.18 -0.09 -0.42 
Ca21Chr1_0244556 -0.45  0.12  0.15  0.09  0.13  0.40 
Ca21Chr1_0244615  0.16  0.11  0.15 -0.02  0.28  0.02  orf19.3308|
Ca21Chr1_0244674 -0.22  0.15  0.23 -0.30 -0.30 -0.07 
Ca21Chr1_0244733 -0.10 -0.03 -0.06  0.06 -0.23 -0.07 
Ca21Chr1_0244792 -0.01  0.14 -0.00  0.04  0.22  0.24 
Ca21Chr1_0244851  0.16  0.08  0.13 -0.18 -0.21 -0.51 
Ca21Chr1_0244910 -0.14  0.01 -0.16 -0.02 -0.16 -0.05 
Ca21Chr1_0244969  0.57 -0.15 -0.23 -0.29 -0.75 -0.28 
Ca21Chr1_0245028 -0.52 -0.12 -0.01  0.33  0.03  0.03 
Ca21Chr1_0245087  0.45 -0.15  0.05 -0.02  0.16 -0.06 
Ca21Chr1_0245146  0.08 -0.44  0.11  0.47 -0.43 -0.19 
Ca21Chr1_0245205  0.36 -0.24 -0.23 -0.07  0.13 -0.43  orf19.3309>
Ca21Chr1_0245264  0.36 -0.09  0.16  0.18  0.11 -0.40 
Ca21Chr1_0245323 -0.03  0.11  0.16 -0.22  0.08 -0.15 
Ca21Chr1_0245382  0.09  0.26  0.13  0.40 -0.16 -0.22 
Ca21Chr1_0245441  0.46  0.43  0.63  0.29  0.30  0.33 
Ca21Chr1_0245500  0.17  0.17  0.67 -0.01 -0.08  0.07 
Ca21Chr1_0245559  0.02  0.23  0.38  0.11  0.16 -0.15 
Ca21Chr1_0245618  0.22  0.13  0.64  0.40  0.16 -0.25 
Ca21Chr1_0245677  0.24  0.01  0.25 -0.11  0.93  0.16 
Ca21Chr1_0245736  0.08  0.15  0.40  0.57 -0.07 -0.08 
Ca21Chr1_0245795  0.12  0.74  0.01 -0.30 -0.11  0.09 
Ca21Chr1_0245854  0.12 -0.16  0.15  0.38  0.03  0.28 
Ca21Chr1_0245913  0.04 -0.31 -0.06  0.00 -0.16 -0.02 
Ca21Chr1_0245972 -0.04 -0.27 -0.08  0.03  0.18  0.06 
Ca21Chr1_0246031 -0.25  0.32 -1.05 -0.00 -0.10 -0.37 
Ca21Chr1_0246090  0.02 -0.13 -0.03 -0.13  0.03 -0.19 
Ca21Chr1_0246149 -0.96  0.24 -0.14 -0.46  0.06 -0.19 
Ca21Chr1_0246208 -0.81 -0.11  0.26  0.39  0.42  0.19 
Ca21Chr1_0246267  0.02 -0.16 -0.03 -0.33 -0.04 -0.18 
Ca21Chr1_0246326  0.36  0.10  0.45 -0.17 -0.17 -0.12 
Ca21Chr1_0246385  0.35  0.09  0.24 -0.47  0.20 -0.15 
Ca21Chr1_0246444 -0.63  0.15 -0.06  0.20 -0.29 -0.51  orf19.3309|
Ca21Chr1_0246503 -0.02  0.16  0.01  0.04 -0.51  1.77 
Ca21Chr1_0246562  0.26  0.09  0.15  0.01 -0.13 -0.84 
Ca21Chr1_0246621  0.32 -0.08 -0.04  0.19  0.82 -0.05  |orf19.3310
Ca21Chr1_0246680  0.14  0.23  0.15 -0.04  0.20 -0.05 
Ca21Chr1_0246739  0.10  0.08  0.03  0.13 -0.02 -0.06 
Ca21Chr1_0246798  0.14 -0.08  0.33  0.11 -0.31 -0.32 
Ca21Chr1_0246857  0.43  0.08  0.48 -0.30  0.69 -0.59 
Ca21Chr1_0246916 -0.06  0.17  0.57 -0.29  0.00  0.09 
Ca21Chr1_0246975  0.09  0.13  0.20 -0.25 -0.09 -0.28 
Ca21Chr1_0247034  0.08 -0.00 -0.53 -0.17  0.11 -0.52 
Ca21Chr1_0247093 -0.19 -0.24 -0.50 -0.16  0.03 -0.21 
Ca21Chr1_0247152  0.14 -0.74 -1.03 -0.27  0.04 -0.11 
Ca21Chr1_0247211 -0.80 -0.96 -1.19 -0.03  0.04  0.29 
Ca21Chr1_0247270 -0.28 -1.43 -1.40 -0.11  0.03 -0.16 
Ca21Chr1_0247329 -1.10 -1.36 -1.55  0.02 -0.27 -0.18 
Ca21Chr1_0247388 -1.69 -1.77 -2.29  0.19 -0.19  0.14  <orf19.3310
Ca21Chr1_0247447  0.07 -1.94 -2.69 -0.12 -0.21 -0.04 
Ca21Chr1_0247506 -1.28 -1.81 -1.97 -0.15  0.20 -0.22 
Ca21Chr1_0247565  0.16 -1.39 -2.18 -0.07 -0.08  0.86 
Ca21Chr1_0247624  0.08 -0.58 -2.11  0.06  0.32  0.27 
Ca21Chr1_0247683 -0.44 -0.55 -2.00 -0.27 -0.76 -0.12 
Ca21Chr1_0247742 -0.44 -1.08 -1.54 -0.37 -0.18  0.57 
Ca21Chr1_0247801 -0.68 -0.59 -1.79 -0.66 -0.10  0.50 
Ca21Chr1_0247860 -0.47 -1.03 -0.91 -0.32  0.33  0.09 
Ca21Chr1_0247919 -0.03 -0.45 -0.76 -0.33  0.01  0.21 
Ca21Chr1_0247978  0.84 -0.04 -0.39  0.36  0.34  0.03 
Ca21Chr1_0248037  0.33  0.54 -0.09 -0.06  0.02 -0.02 
Ca21Chr1_0248096 -0.86  0.32 -0.26  0.61  0.08  0.17 
Ca21Chr1_0248155  0.24  0.06 -0.24 -0.36 -0.25  0.11 
Ca21Chr1_0248214 -1.03 -0.46 -0.95 -0.49 -1.12  0.11 
Ca21Chr1_0248273 -0.39  0.55 -0.44 -0.48 -0.06 -0.29 
Ca21Chr1_0248332 -0.35 -0.10 -0.96 -0.61 -0.38 -0.06 
Ca21Chr1_0248391  0.14  0.37  0.31 -0.69 -0.18  0.35 
Ca21Chr1_0248450 -0.25 -0.40 -0.90 -1.67 -0.44 -0.13 
Ca21Chr1_0248509 -0.38 -0.33 -0.69 -1.12 -0.20  0.26 
Ca21Chr1_0248568 -0.09 -0.63 -0.54 -0.40 -0.28 -0.06 
Ca21Chr1_0248627  0.33 -0.14 -0.13 -0.39 -0.12  0.45 
Ca21Chr1_0248686  0.18  0.12 -0.03 -0.08 -0.20  0.26 
Ca21Chr1_0248745  0.13 -0.11  0.38  0.06 -0.14  0.00 
Ca21Chr1_0248804 -0.33 -0.07  0.31 -0.37 -0.17  0.11  orf19.3311>
Ca21Chr1_0248863 -0.02  0.11  0.24 -0.41 -0.18 -0.01 
Ca21Chr1_0248922  1.02  0.21  0.05 -0.45 -0.47 -0.19 
Ca21Chr1_0248981  0.58 -0.07  0.47  0.02  0.03  0.21 
Ca21Chr1_0249040  0.26  0.11 -0.41 -0.16  0.25 -0.00 
Ca21Chr1_0249099 -0.14  0.17  0.11 -0.12  0.01  0.19 
Ca21Chr1_0249158  0.12  0.16  0.32  0.33  0.40 -0.11 
Ca21Chr1_0249217 -0.02  0.29  0.28  0.11 -0.08 -0.14 
Ca21Chr1_0249276  0.09  0.49  0.38  0.42 -0.03  0.09 
Ca21Chr1_0249335  0.25  0.36  0.36  0.10  0.02 -0.11 
Ca21Chr1_0249394  0.37  0.37  0.03  0.18  0.30 -0.04 
Ca21Chr1_0249453 -0.03  0.33  0.31 -0.35  0.16 -0.17 
Ca21Chr1_0249512  0.22  0.43 -0.30  0.17  0.28  0.21 
Ca21Chr1_0249571  0.06  0.26  0.36  0.06  0.09  0.07 
Ca21Chr1_0249630  0.11 -0.15  0.07  0.08  0.08 -0.19 
Ca21Chr1_0249689  0.42  0.44  0.07  0.14  0.05 -0.17 
Ca21Chr1_0249748 -0.56  0.15 -0.21 -0.02  0.20  0.50 
Ca21Chr1_0249807 -0.14  0.06 -0.70  0.23  0.13  0.19 
Ca21Chr1_0249866 -0.11  0.24 -0.77  0.09  0.00  0.33  orf19.3311|
Ca21Chr1_0249925  0.66  0.00 -1.08  0.05  0.02 -0.17 
Ca21Chr1_0249984 -0.28 -0.30 -1.16  0.26  0.04  0.16 
Ca21Chr1_0250043 -0.58 -1.05 -1.37  0.30  0.14  0.23 
Ca21Chr1_0250102 -0.39 -0.43 -0.95  0.31  0.31  0.05  orf19.3312>
Ca21Chr1_0250161 -0.28 -0.24 -0.94  0.25  0.08 -0.02 
Ca21Chr1_0250220 -0.10 -0.95 -0.71  0.19  0.17 -0.21 
Ca21Chr1_0250279 -0.13 -0.30 -0.35 -0.18 -0.22 -0.08 
Ca21Chr1_0250338  0.28 -0.38 -0.32  0.43  0.27  0.17 
Ca21Chr1_0250397 -0.06 -0.54  0.01 -0.02 -0.03  0.26 
Ca21Chr1_0250456 -0.06  0.02 -0.07 -0.10 -0.38  0.03 
Ca21Chr1_0250515 -0.83  0.14  0.49 -0.04 -0.65  0.82 
Ca21Chr1_0250574  0.13  0.36  0.16  0.03  0.07  0.30 
Ca21Chr1_0250633 -0.05  0.06 -0.02  0.26  0.05  0.35 
Ca21Chr1_0250692  0.04  0.17  0.25  0.22 -0.39  0.12 
Ca21Chr1_0250751 -0.29 -0.04  0.01  0.36 -0.19  0.08 
Ca21Chr1_0250810  0.02  0.19  0.29  0.34 -0.11  0.00 
Ca21Chr1_0250869 -0.02  0.06  0.31  0.40  0.10 -0.20 
Ca21Chr1_0250928  0.17  0.09  0.25  0.08 -0.05  0.09 
Ca21Chr1_0250987 -0.06  0.21  0.21  0.06  0.00 -0.10 
Ca21Chr1_0251046  0.10  0.08  0.16  0.22  0.01  0.03 
Ca21Chr1_0251105 -0.39  0.16  0.36  0.34  0.01 -0.18 
Ca21Chr1_0251164 -0.17 -0.03  0.27  0.43 -0.14 -0.04 
Ca21Chr1_0251223  0.03  0.23  0.32  0.22 -0.30  0.39 
Ca21Chr1_0251282  0.26  0.40  0.31 -0.18  0.09  0.10 
Ca21Chr1_0251341  0.05  0.30  0.14  0.03 -0.06 -0.22 
Ca21Chr1_0251400  0.25  0.03  0.13  0.18 -0.24 -0.32 
Ca21Chr1_0251459 -0.06  0.13  0.03  0.24 -0.22 -0.22 
Ca21Chr1_0251518  0.08  0.18  0.16 -0.00 -0.19  0.02 
Ca21Chr1_0251577  0.17  0.51  0.33 -0.18 -0.05  0.09 
Ca21Chr1_0251636  0.07  0.39  0.33  0.07 -0.00 -0.16 
Ca21Chr1_0251695  0.08  0.47  0.11  0.07  0.02 -0.21 
Ca21Chr1_0251754  0.07  0.08  0.19 -0.09 -0.15 -0.71 
Ca21Chr1_0251813  0.21  0.23  0.25  0.08  0.00 -0.08 
Ca21Chr1_0251872  0.17  0.02  0.18 -0.33 -0.18 -0.26 
Ca21Chr1_0251931  0.01  0.16  0.32  0.51  0.30 -0.08  orf19.3312|
Ca21Chr1_0251990  0.28 -0.08 -0.39  0.09 -0.03 -0.02  |orf19.3314
Ca21Chr1_0252049  0.02 -0.36 -0.29  0.12  0.04  0.13 
Ca21Chr1_0252108 -0.13 -0.11 -0.28  0.14  0.15  0.10 
Ca21Chr1_0252167  0.35 -0.05  0.02 -0.18 -0.27  0.05 
Ca21Chr1_0252226 -0.07  0.05 -0.21  0.18 -0.13 -0.18 
Ca21Chr1_0252285 -0.03 -0.10  0.18  0.03 -0.25  0.02 
Ca21Chr1_0252344 -0.14  0.13  0.01  0.27  0.20  0.14 
Ca21Chr1_0252403 -0.67 -0.24 -0.20  0.22  0.09 -0.37 
Ca21Chr1_0252462 -0.05 -0.39  0.12  0.16  0.13  0.04  <orf19.3314
Ca21Chr1_0252521 -0.23 -0.39 -0.77  0.46 -0.02  0.19 
Ca21Chr1_0252580 -0.91 -0.28 -0.97 -0.08  0.32 -0.17 
Ca21Chr1_0252639 -0.77 -0.68 -1.53 -0.12 -0.15 -0.14 
Ca21Chr1_0252698 -1.46 -1.25 -1.38 -0.01 -0.41 -0.05 
Ca21Chr1_0252757 -0.94 -1.26 -1.29 -0.01  0.18 -0.03 
Ca21Chr1_0252816 -1.03 -0.91 -1.43 -0.24  0.30 -0.18 
Ca21Chr1_0252875 -1.03 -0.73 -1.75 -0.17 -0.14 -0.04 
Ca21Chr1_0252934 -0.22 -0.64 -0.76  0.34  0.19 -0.56 
Ca21Chr1_0252993 -0.05 -0.21 -0.58  0.30  0.38 -0.50 
Ca21Chr1_0253052 -0.43 -0.30  0.04  0.57 -0.13 -0.27 
Ca21Chr1_0253111 -0.75 -0.00 -0.29  0.51 -0.15  0.68  orf19.3315>
Ca21Chr1_0253170 -0.01  0.19  0.17 -0.03 -0.15  0.15 
Ca21Chr1_0253229 -0.08 -0.09  0.13 -0.34  0.26  0.28 
Ca21Chr1_0253288 -0.01 -0.14  0.20  0.36  0.06  0.29 
Ca21Chr1_0253347 -0.14  0.12 -0.08  0.05 -0.36  0.70 
Ca21Chr1_0253406  0.38 -0.11  0.02  0.07  0.02  0.28 
Ca21Chr1_0253465 -0.05  0.10  0.10  0.17 -0.02  0.68 
Ca21Chr1_0253524 -0.04  0.17  0.09  0.48  0.16  0.27 
Ca21Chr1_0253583  0.14  0.11 -0.03 -0.07  0.13 -0.49 
Ca21Chr1_0253642  0.11  0.12 -0.32 -0.06 -0.78  0.21 
Ca21Chr1_0253701  0.16  0.08  0.19 -0.11 -0.10 -0.03 
Ca21Chr1_0253760  0.26 -0.15  0.41 -0.19  0.04 -0.34 
Ca21Chr1_0253819 -0.09  0.18 -0.15  0.18 -0.37 -0.06 
Ca21Chr1_0253878  0.21  0.24  0.18  0.20  0.35 -0.04 
Ca21Chr1_0253937 -0.02  0.12 -0.06  0.10  0.03 -0.06 
Ca21Chr1_0253996  0.27  0.25 -0.01 -0.16  0.15 -0.11 
Ca21Chr1_0254055  1.40  0.39  2.05  0.62  0.28 -1.01 
Ca21Chr1_0254114  0.03  0.22  0.34  0.44 -0.17 -0.06 
Ca21Chr1_0254173  0.44  0.09  0.34 -0.01  0.29  0.55 
Ca21Chr1_0254232 -0.08  0.31 -0.36 -1.04  1.26  0.03 
Ca21Chr1_0254291 -0.14  0.48  0.33  0.02 -0.07 -0.21 
Ca21Chr1_0254350 -0.03  0.37  0.36 -0.71  0.15 -0.52 
Ca21Chr1_0254409 -0.26  0.56  0.37 -0.19 -0.15  0.16  orf19.3315|
Ca21Chr1_0254468  0.15  0.16  0.31  0.05  0.08  0.40 
Ca21Chr1_0254527  0.33  0.37  0.28  0.19 -0.05 -0.18 
Ca21Chr1_0254586 -0.12 -0.11 -0.24  0.02 -0.55  0.20  |orf19.3317
Ca21Chr1_0254645 -0.10  0.11  0.24 -0.12 -0.04  0.26 
Ca21Chr1_0254704 -0.00  0.39  0.11 -0.65 -0.40 -0.19 
Ca21Chr1_0254763 -0.26  0.01  0.34  0.03 -0.24  0.58 
Ca21Chr1_0254822  0.14  0.33 -0.02  0.27 -0.14  0.00 
Ca21Chr1_0254881  0.11  0.05 -0.22  0.12  0.54 -0.43 
Ca21Chr1_0254940  0.37  0.05 -0.18 -0.17 -0.24  0.32  <orf19.3317
Ca21Chr1_0254999  0.11 -0.15  0.23 -0.21 -0.74 -0.04 
Ca21Chr1_0255058 -0.09  0.09  0.06  0.23 -0.51  0.30 
Ca21Chr1_0255117 -0.39 -0.14 -0.05  0.44 -0.36  0.41 
Ca21Chr1_0255176  0.14 -0.57  0.29  0.19  1.13 -0.04  |orf19.3318
Ca21Chr1_0255235 -0.13  0.00 -0.08 -0.28 -0.01  0.59 
Ca21Chr1_0255294  0.03  0.40  0.08  0.35  0.18  0.45 
Ca21Chr1_0255353 -0.27  0.18  0.39  0.19 -0.35  1.13 
Ca21Chr1_0255412 -0.20  0.29  0.15  0.16 -0.08  0.55 
Ca21Chr1_0255471  0.54  0.25 -0.03 -0.18 -0.19  0.45 
Ca21Chr1_0255530  0.07  0.12  0.47 -0.29 -0.49  0.93 
Ca21Chr1_0255589  0.52  0.22  0.15 -0.28 -0.17  0.16 
Ca21Chr1_0255648 -0.01  0.44  0.23  0.16 -0.18 -0.13 
Ca21Chr1_0255707  0.09  0.36  0.10 -0.02  0.09 -0.46 
Ca21Chr1_0255766  0.03  0.37  0.30 -0.38 -0.17 -0.22 
Ca21Chr1_0255825 -0.11  0.43  0.06  0.27 -0.23 -0.57 
Ca21Chr1_0255884 -0.23  0.83  0.42  0.82  0.00 -0.27 
Ca21Chr1_0255943  0.07  0.06  0.42  0.05  0.02 -0.26 
Ca21Chr1_0256002  0.04  1.21  0.48 -0.18 -0.29 -0.20 
Ca21Chr1_0256061  0.24  0.46  0.36 -0.22  0.24 -0.20 
Ca21Chr1_0256120  0.34  0.19  0.19  0.12  0.09 -0.81 
Ca21Chr1_0256179  0.23  0.29  0.37  0.27  0.05 -0.00 
Ca21Chr1_0256238 -0.08 -0.04  0.04  0.07  0.18 -0.48 
Ca21Chr1_0256297  0.62 -0.16  0.30 -0.21  0.30 -0.10 
Ca21Chr1_0256356  0.19  0.08  0.36 -0.12  0.25  0.27 
Ca21Chr1_0256415  0.22 -0.12  0.22  0.17  0.35  0.08 
Ca21Chr1_0256474  0.03 -0.07  0.20  0.02  0.25 -0.21 
Ca21Chr1_0256533  0.18  0.12  0.45  0.43  0.57 -0.07 
Ca21Chr1_0256592  0.05 -0.02  0.50  0.07 -0.12 -0.12 
Ca21Chr1_0256651 -0.02  0.18  0.26  0.15 -0.15  0.13 
Ca21Chr1_0256710  0.24  0.19  0.13 -0.32 -0.06  0.09 
Ca21Chr1_0256769 -0.21 -0.06 -0.50 -0.48  0.06  0.02 
Ca21Chr1_0256828 -0.30 -0.22 -0.40 -0.46 -0.04 -0.04 
Ca21Chr1_0256887 -0.73 -0.62 -0.09 -0.15 -0.18  0.26 
Ca21Chr1_0256946 -0.65 -0.79 -0.67  0.05 -0.01 -0.16 
Ca21Chr1_0257005 -0.68 -0.88 -1.16 -0.07  0.05 -0.39 
Ca21Chr1_0257064 -0.36 -0.59 -0.75  0.06  0.09 -0.20 
Ca21Chr1_0257123  0.11 -0.33 -0.46  0.48  0.13 -0.09 
Ca21Chr1_0257182 -0.13 -0.34 -0.26 -0.07 -0.06  0.04 
Ca21Chr1_0257241  0.15  0.01 -0.20 -0.25  0.04  0.10 
Ca21Chr1_0257300  0.05  0.22 -0.22  0.17 -0.21  0.21 
Ca21Chr1_0257359  0.37  0.09  0.04  0.10  0.03  0.08 
Ca21Chr1_0257418 -0.10  0.25  0.12  0.12 -0.29  0.47  <orf19.3318
Ca21Chr1_0257477 -0.08 -0.08  0.05  0.31  0.22  0.54 
Ca21Chr1_0257536 -0.06  0.02  0.39  0.24  0.05  0.06 
Ca21Chr1_0257595 -0.58 -0.01  0.05 -0.33  0.36 -0.10 
Ca21Chr1_0257654  0.20 -0.09 -0.06  0.19  0.22  0.24 
Ca21Chr1_0257713  0.22  0.07 -0.27 -0.46  0.02  0.10 
Ca21Chr1_0257772 -0.08 -0.23  0.09  0.05  0.21 -0.91 
Ca21Chr1_0257831  0.30  0.08 -0.11  0.01 -0.10  0.05 
Ca21Chr1_0257890  0.70  0.04  0.39 -0.29  0.07 -0.03  orf19.3319>
Ca21Chr1_0257949  0.24  0.13  0.09 -0.08 -0.39 -0.48 
Ca21Chr1_0258008  0.20  0.20  0.10 -0.41 -0.09 -0.28 
Ca21Chr1_0258067 -0.59  0.76  0.46 -0.12 -0.29 -0.14 
Ca21Chr1_0258126  0.04  0.23  0.18  0.14 -0.33 -0.01 
Ca21Chr1_0258185  0.20  0.10  0.24 -0.21 -0.03 -0.11 
Ca21Chr1_0258244 -0.14  0.27  0.18  0.37 -0.80 -0.14 
Ca21Chr1_0258303  0.22  0.05 -0.01 -0.34 -0.13  0.10 
Ca21Chr1_0258362 -0.32  0.02  0.17 -0.19 -0.20  0.65 
Ca21Chr1_0258421 -0.38  0.14  0.36 -0.18 -0.06  0.47 
Ca21Chr1_0258480  0.03  0.09  0.05  0.09 -0.44  0.46 
Ca21Chr1_0258539 -0.41 -0.27  0.02 -0.23 -0.30  0.02 
Ca21Chr1_0258598 -0.31 -0.01  0.00 -0.33 -0.18  0.38 
Ca21Chr1_0258657 -0.23 -0.04 -0.53 -0.15  0.06  0.01 
Ca21Chr1_0258716 -0.27  0.05 -0.50  0.07 -0.02  0.40 
Ca21Chr1_0258775 -0.44  0.21 -0.67 -0.14  0.39 -0.26 
Ca21Chr1_0258834 -0.88 -0.53 -1.15 -0.54 -0.23  0.19 
Ca21Chr1_0258893 -1.33 -1.09 -1.93 -0.58 -0.33 -0.06  orf19.3319|
Ca21Chr1_0258952 -1.31 -1.00 -1.99 -1.76 -1.12 -0.18  orf19.3320>
Ca21Chr1_0259011 -1.36 -1.61 -1.52 -1.10 -0.48 -0.36 
Ca21Chr1_0259070 -1.51 -1.02 -1.39 -1.25 -0.83  0.05 
Ca21Chr1_0259129 -1.33 -1.35 -1.85 -0.58 -0.26  0.16 
Ca21Chr1_0259188 -0.65 -0.93 -1.66 -0.72 -0.29 -0.22 
Ca21Chr1_0259247 -0.50 -0.77 -0.21 -0.13  0.14  0.22 
Ca21Chr1_0259306 -0.36 -0.92 -1.05 -0.24 -0.11  0.33  orf19.3320|
Ca21Chr1_0259365  1.06  0.26  0.41 -0.08  0.04  0.13 
Ca21Chr1_0259424 -0.30 -0.24 -0.29 -0.10  0.30  1.41 
Ca21Chr1_0259483 -0.11 -0.33 -0.04 -0.11 -0.32  0.10 
Ca21Chr1_0259542  0.03  0.02  0.13 -0.27 -0.36  0.12 
Ca21Chr1_0259601  0.23 -0.26  0.34  0.23 -0.10  0.29  orf19.3321>
Ca21Chr1_0259660 -0.08  0.12  0.16 -0.08 -0.20 -0.00 
Ca21Chr1_0259719  0.12  0.12  0.25 -0.19 -0.50  0.20 
Ca21Chr1_0259778  0.24  0.23  0.26  0.06  0.10  0.21 
Ca21Chr1_0259837  0.21 -0.16  0.17  0.23 -0.06  0.17 
Ca21Chr1_0259896  0.17 -0.13  0.40  0.22 -0.63  0.02 
Ca21Chr1_0259955  0.15  0.03  0.12  0.23 -0.01  0.08 
Ca21Chr1_0260014 -0.14  0.59  0.01  0.15 -0.05  0.19 
Ca21Chr1_0260073 -0.04  0.49  0.31  0.03  0.18  0.02 
Ca21Chr1_0260132 -0.15  0.19  0.21  0.06 -0.23 -0.46 
Ca21Chr1_0260191  0.29  0.15  0.02 -0.09  0.03  0.01 
Ca21Chr1_0260250  0.25  0.30  0.19  0.40  0.07  0.03 
Ca21Chr1_0260309  0.05 -0.13  0.23 -0.12 -0.04 -0.15 
Ca21Chr1_0260368 -0.02  0.13  0.22  0.31 -0.06  0.03 
Ca21Chr1_0260427  0.06  0.14  0.37  0.40 -0.20  0.14 
Ca21Chr1_0260486 -0.04 -0.10  0.31 -0.23 -0.05  0.13 
Ca21Chr1_0260545  0.27 -0.28  0.46 -0.30 -0.21  0.07 
Ca21Chr1_0260604  0.26  0.19  0.22 -0.02  0.11 -0.06 
Ca21Chr1_0260663 -0.05  0.24  0.18 -0.05  0.10 -0.12 
Ca21Chr1_0260722 -0.17  0.62  0.28 -0.14  0.05 -0.42 
Ca21Chr1_0260781  0.22  0.48  0.24  0.01 -0.01 -0.09 
Ca21Chr1_0260840  0.04  0.19  0.06 -0.22  0.22 -0.17 
Ca21Chr1_0260899  0.01  0.05  0.14  0.11  0.40 -0.62 
Ca21Chr1_0260958 -0.20  0.06  0.33  0.18  0.14 -0.62 
Ca21Chr1_0261017 -0.07 -0.02 -0.02  0.21  0.14 -0.57 
Ca21Chr1_0261076 -0.31  0.14  0.06  0.03  0.26 -0.45 
Ca21Chr1_0261135  0.44  0.15 -0.01 -0.20  1.00 -0.42  orf19.3321|
Ca21Chr1_0261194  0.34 -0.01  0.02  0.11  0.27 -0.30 
Ca21Chr1_0261253  1.55 -0.14  0.13  0.25 -0.25 -0.13 
Ca21Chr1_0261312  0.43  0.12  0.55  0.13  0.13 -0.49  |orf19.3322
Ca21Chr1_0261371  0.16  0.02  0.31  0.18  0.17 -0.32 
Ca21Chr1_0261430  0.01  0.34  0.34 -0.27 -0.66  0.01 
Ca21Chr1_0261489  0.10  0.43  0.31 -0.18 -0.08 -0.09 
Ca21Chr1_0261548  0.22 -0.01  0.16  0.02  0.18  0.08 
Ca21Chr1_0261607 -0.22  0.30  0.46  0.32  0.18  0.07 
Ca21Chr1_0261666  0.26  0.41  0.52  0.22  0.26 -0.37 
Ca21Chr1_0261725  0.09  0.27  0.13  0.35  0.36  0.51 
Ca21Chr1_0261784 -0.01  0.65  0.37  0.58  0.04  0.37 
Ca21Chr1_0261843  0.11 -0.05  0.51  0.17 -0.40  0.21  <orf19.3322
Ca21Chr1_0261902 -0.14 -0.28  0.11 -0.03 -0.28  0.27 
Ca21Chr1_0261961 -0.63  0.02  0.18 -0.22  0.46  0.30 
Ca21Chr1_0262020  0.20  0.07  0.11  0.15 -0.26  0.01 
Ca21Chr1_0262079  0.14  0.20  0.31 -0.08  0.20 -0.04 
Ca21Chr1_0262138  0.03  0.30  0.78  0.11  0.09  0.07 
Ca21Chr1_0262197  0.47  0.46  0.08  0.07 -0.04  0.05  |orf19.3323
Ca21Chr1_0262256  0.61  0.57  0.64 -0.05  0.29  0.11 
Ca21Chr1_0262315  0.34  0.30  0.39 -0.57  0.06  0.19 
Ca21Chr1_0262374  0.43  0.10  0.10  0.27  0.30  0.12 
Ca21Chr1_0262433  0.20  0.00  0.14  0.35  0.59  0.08 
Ca21Chr1_0262492 -0.03  0.09  0.05  0.28 -0.02  0.27 
Ca21Chr1_0262551 -0.06  0.14 -0.10  0.24 -0.30  0.04 
Ca21Chr1_0262610 -0.03 -0.07  0.17  0.16  0.09  0.29 
Ca21Chr1_0262669  0.48  0.14 -0.09  0.03 -0.02  0.64 
Ca21Chr1_0262728  0.13 -0.14 -0.20  0.15  0.10  0.94 
Ca21Chr1_0262787  0.20  0.32  0.14  0.06 -0.55  0.39 
Ca21Chr1_0262846  0.24 -0.18 -0.05  0.03  0.29  0.04 
Ca21Chr1_0262905  0.58 -0.07  0.29 -0.15  0.06  0.21 
Ca21Chr1_0262964  0.12  0.17  0.15 -0.08  0.23  0.12 
Ca21Chr1_0263023  0.09  0.30  0.43  0.17  0.01  0.09 
Ca21Chr1_0263082 -0.20  0.24  0.01 -0.04 -0.00  0.12  <orf19.3323
Ca21Chr1_0263141  1.16  0.02  0.29 -0.32  0.26 -0.20 
Ca21Chr1_0263200 -1.09 -0.17  0.37 -0.02  0.35  0.34 
Ca21Chr1_0263259  0.62  0.11 -0.30 -0.09 -0.05 -0.14 
Ca21Chr1_0263318  1.32 -0.27 -0.45 -0.11  0.32 -0.04 
Ca21Chr1_0263377  0.74 -0.29  0.14  0.37  0.12  0.20 
Ca21Chr1_0263436  0.91 -0.35  0.42  0.38  0.37  0.10  |orf19.3324
Ca21Chr1_0263495  0.87  0.26  0.23  0.16  0.37  0.49 
Ca21Chr1_0263554  0.78  0.12  0.42 -0.09 -0.13  0.56 
Ca21Chr1_0263613  0.66  0.04  0.26  0.15 -0.03  0.45 
Ca21Chr1_0263672  0.89  0.17  0.26  0.27  0.28  0.09 
Ca21Chr1_0263731  0.93 -0.07  0.41  0.20  0.11  0.03 
Ca21Chr1_0263790  0.26 -0.04  0.28  0.15  0.10 -0.06 
Ca21Chr1_0263849  1.34  0.18  0.11 -0.10 -0.02 -0.12 
Ca21Chr1_0263908  0.73  0.10  0.43  0.50  0.32  0.17 
Ca21Chr1_0263967  0.43 -0.03 -0.24  0.04  0.11  0.10 
Ca21Chr1_0264026  0.55 -0.06  0.26  0.26  0.18  0.12 
Ca21Chr1_0264085  0.70 -0.02  0.29  0.32  0.40  0.93 
Ca21Chr1_0264144  0.28 -0.01  0.06 -0.17  0.08 -0.12 
Ca21Chr1_0264203  0.24  0.19 -0.16 -0.06  0.23  0.05 
Ca21Chr1_0264262  0.15  0.65  0.43  0.02  0.09 -0.19 
Ca21Chr1_0264321  0.98  0.05  0.47  0.09  0.24 -0.37 
Ca21Chr1_0264380  0.86 -0.22  0.34  0.06  0.18 -0.24 
Ca21Chr1_0264439  0.52  0.05  0.28 -0.13  0.22 -0.17 
Ca21Chr1_0264498  1.33  0.30  0.84  0.03  0.04 -0.24 
Ca21Chr1_0264557  1.21  0.09  0.19  0.16 -0.02  0.09 
Ca21Chr1_0264616  0.97  0.19  0.39 -0.15 -0.03  0.78 
Ca21Chr1_0264675  0.56  0.25  0.28  0.05 -0.47  0.15  <orf19.3324
Ca21Chr1_0264734 -0.27 -0.06 -0.20  0.10 -0.20 -1.22 
Ca21Chr1_0264793  0.03 -0.35 -0.56 -0.54 -0.18  0.41 
Ca21Chr1_0264852  0.08  0.21 -0.27 -0.05  0.06 -0.10 
Ca21Chr1_0264911 -0.03 -0.18 -0.24  0.03 -0.03  0.22 
Ca21Chr1_0264970  0.19  0.26 -0.00  0.08 -0.09 -0.21 
Ca21Chr1_0265029 -0.06  0.20  0.13 -0.25 -0.08 -0.38 
Ca21Chr1_0265088  0.63 -0.19  0.11 -0.19  0.06 -0.29 
Ca21Chr1_0265147  0.05 -0.03 -0.07  0.08 -0.11 -0.33 
Ca21Chr1_0265206  0.20  0.37  0.12 -0.24  0.30 -0.55 
Ca21Chr1_0265265  0.07  0.16  0.25 -0.04 -0.03 -0.12 
Ca21Chr1_0265324  0.00  0.04 -0.06 -0.12 -0.21 -0.23 
Ca21Chr1_0265383  0.20  0.16  0.25  0.37 -0.15 -0.09 
Ca21Chr1_0265442  0.41  0.33  0.26  0.01  0.19 -0.87  |orf19.3325
Ca21Chr1_0265501  0.20  0.23  0.19 -0.40  0.04 -0.19 
Ca21Chr1_0265560  0.26  0.10  0.09 -0.66  0.00 -0.14 
Ca21Chr1_0265619  0.46  0.29  0.19 -0.38 -0.02 -0.25 
Ca21Chr1_0265678  0.35  0.37  0.00 -0.17  0.11 -0.16 
Ca21Chr1_0265737  0.42  1.12 -0.14 -0.36  0.23 -0.03 
Ca21Chr1_0265796  0.32  0.32  0.47 -0.22 -0.14  0.31 
Ca21Chr1_0265855  0.11  0.49  0.29 -0.56 -0.60 -0.13 
Ca21Chr1_0265914  0.19  0.21  0.08 -0.27  0.07 -0.14 
Ca21Chr1_0265973  0.11  0.26 -0.13  0.14  0.28  0.10 
Ca21Chr1_0266032  0.18 -0.03  0.09  0.65  0.17  0.43 
Ca21Chr1_0266091  0.49  0.05 -0.10 -0.48  0.04  0.29 
Ca21Chr1_0266150  0.04  0.21  0.17 -0.44  0.29  0.29 
Ca21Chr1_0266209  0.37  0.16  0.06 -0.47  0.24  0.00 
Ca21Chr1_0266268  0.36  0.66  0.48 -0.09  0.10  0.10 
Ca21Chr1_0266327  0.15  0.46  0.28 -0.23  1.12  0.15 
Ca21Chr1_0266386  0.22 -0.06  0.37 -0.40  0.11 -0.05 
Ca21Chr1_0266445  0.19  0.10  0.37 -0.07  0.15  0.05 
Ca21Chr1_0266504  0.26 -0.20  0.44 -0.28 -0.08  0.11 
Ca21Chr1_0266563 -0.24  0.19  1.22 -0.44  0.51 -0.44 
Ca21Chr1_0266622  0.07  0.43  0.47 -0.38  0.31  0.21 
Ca21Chr1_0266681  0.31  0.27  1.11 -0.50  0.48  0.64 
Ca21Chr1_0266740  0.06 -0.00  0.08 -0.71 -0.03  0.26 
Ca21Chr1_0266799  0.10 -0.02  0.17 -0.85  0.56  0.32 
Ca21Chr1_0266858  0.06 -0.31  0.20 -0.60  0.05  0.10 
Ca21Chr1_0266917  0.04 -1.13  0.22 -1.03 -0.44 -0.23 
Ca21Chr1_0266976 -0.40 -0.12  0.09 -1.05 -0.34  0.02 
Ca21Chr1_0267035  0.27  0.28  0.07 -1.29 -0.47  0.15 
Ca21Chr1_0267094  0.12  0.03  0.34 -1.38 -0.19 -0.06 
Ca21Chr1_0267153  0.17  0.23 -0.13 -1.48 -0.46  0.14 
Ca21Chr1_0267212  0.35  0.08  0.07 -1.93 -0.53 -0.13 
Ca21Chr1_0267271  0.30 -0.87  0.16 -2.00 -0.63 -0.28 
Ca21Chr1_0267330  0.19  0.23  0.35 -2.02 -0.68 -0.13 
Ca21Chr1_0267389 -0.07  0.23 -0.14 -1.09 -0.53 -0.67 
Ca21Chr1_0267448 -0.26 -0.09 -0.18 -0.80 -0.67 -0.01  <orf19.3325
Ca21Chr1_0267507  0.17 -0.11 -0.43 -0.95 -1.43  0.23 
Ca21Chr1_0267566 -0.44 -0.35  0.06 -0.77 -0.38  0.41 
Ca21Chr1_0267625  0.14 -0.17 -0.65 -0.89 -0.33  0.22 
Ca21Chr1_0267684 -0.54 -0.04 -0.73 -0.83 -0.41 -0.05 
Ca21Chr1_0267743 -0.68 -0.22 -1.18 -0.24 -0.44  0.31 
Ca21Chr1_0267802 -1.78 -1.05 -1.51 -0.56 -0.47 -0.18 
Ca21Chr1_0267861 -1.91 -1.34 -1.45 -0.04  0.32 -0.01 
Ca21Chr1_0267920 -2.17 -1.06 -1.99 -0.05  0.08 -0.08 
Ca21Chr1_0267979 -2.76 -1.44 -2.00  0.19  0.60  0.49 
Ca21Chr1_0268038 -2.99 -1.72 -2.44 -0.66 -0.08  0.29 
Ca21Chr1_0268097 -2.93 -1.73 -2.98 -0.10  0.44  0.16 
Ca21Chr1_0268156 -1.91 -2.41 -2.73 -0.55 -0.18  0.29 
Ca21Chr1_0268215 -2.39 -3.11 -2.20 -0.14  0.11  0.42 
Ca21Chr1_0268274 -1.65 -0.31 -2.33 -0.19  0.02  0.43 
Ca21Chr1_0268333 -1.19 -0.96 -1.75 -0.28 -0.02  0.05 
Ca21Chr1_0268392 -0.72 -0.47 -1.45 -1.01  0.05  0.31 
Ca21Chr1_0268451 -0.71 -0.24 -0.96 -0.11 -0.04  0.41 
Ca21Chr1_0268510 -0.76 -0.55 -1.70  0.03 -0.10 -0.01 
Ca21Chr1_0268569 -1.78 -0.59 -2.30 -0.57  0.09  0.09 
Ca21Chr1_0268628 -1.14 -0.93 -2.14 -0.10 -0.15 -0.63 
Ca21Chr1_0268687 -2.89 -2.40 -2.76 -0.20 -0.12 -0.16 
Ca21Chr1_0268746 -3.40 -2.44 -2.14 -0.25  0.02 -0.09 
Ca21Chr1_0268805 -2.88 -2.47 -1.98 -0.27  0.22 -0.05 
Ca21Chr1_0268864 -2.32 -2.03 -1.78 -0.45  0.25 -0.09 
Ca21Chr1_0268923 -1.10 -1.03 -1.25  0.03 -0.17 -0.05 
Ca21Chr1_0268982 -1.07 -0.95 -0.69 -0.08 -0.07 -0.32 
Ca21Chr1_0269041 -0.87 -0.69 -0.72 -0.25 -0.12 -0.39 
Ca21Chr1_0269100 -1.15 -0.34 -1.00  0.21 -0.34 -0.60 
Ca21Chr1_0269159 -0.79 -0.15 -0.24 -0.10  0.21 -0.29 
Ca21Chr1_0269218  1.05 -0.23 -0.64 -0.24  0.45 -0.07 
Ca21Chr1_0269277  1.75 -0.21 -0.55 -0.14 -0.18 -0.32 
Ca21Chr1_0269336  0.89  0.34 -0.25 -0.35  0.72  0.03  |orf19.3325.3
Ca21Chr1_0269395  0.17  0.01 -0.03 -0.07  0.10  0.14 
Ca21Chr1_0269454  0.63  2.04  0.16 -0.09  0.30  0.07 
Ca21Chr1_0269513  0.54  0.30 -0.04  0.03 -0.01  0.03 
Ca21Chr1_0269572 -0.40  0.04  0.06 -0.06  0.08  0.09 
Ca21Chr1_0269631 -0.21  0.21  0.25 -0.02  0.03 -0.16 
Ca21Chr1_0269690  0.10 -0.03 -0.45 -0.02  0.28 -0.06 
Ca21Chr1_0269749 -0.11 -0.31 -0.02 -0.75  0.02  0.11 
Ca21Chr1_0269808 -0.04 -0.30 -0.04 -0.45 -0.20 -0.01 
Ca21Chr1_0269867  0.45  0.16  0.15 -0.19 -0.11  0.35 
Ca21Chr1_0269926 -0.24  0.13  0.15 -0.43 -0.11  0.40 
Ca21Chr1_0269985  0.39 -0.03  0.19 -0.19 -0.05  0.81  <orf19.3325.3
Ca21Chr1_0270044 -0.15 -0.02  0.07 -0.43 -0.27  0.23 
Ca21Chr1_0270103  0.26  0.10 -0.14  0.04 -0.12  0.46 
Ca21Chr1_0270162  0.01  0.10  0.07  0.19 -0.20  0.54 
Ca21Chr1_0270221 -0.44  0.04  0.15 -0.36  0.14  0.35 
Ca21Chr1_0270280  0.45  0.60  0.18 -0.11  0.29 -1.01  |orf19.3327
Ca21Chr1_0270339 -0.04  0.30 -0.10 -0.00  0.20  0.03 
Ca21Chr1_0270398  0.30  0.42 -0.11 -0.08 -0.48 -0.12 
Ca21Chr1_0270457  0.40  0.30  0.27  0.10 -0.22  0.00 
Ca21Chr1_0270516  0.04  0.15  0.12  0.16  0.04  0.01 
Ca21Chr1_0270575  0.32  0.61 -0.13 -0.36 -0.11 -0.20 
Ca21Chr1_0270634  0.02 -0.09  0.04 -0.13 -0.05 -0.27 
Ca21Chr1_0270693 -0.28  0.06 -0.07  0.16 -0.33 -0.23 
Ca21Chr1_0270752 -0.01  0.04 -0.19  0.44  0.10  0.34 
Ca21Chr1_0270811  0.11  0.01  0.11  0.20  0.03  0.18 
Ca21Chr1_0270870 -0.10 -0.14  0.14  0.45  0.01  0.09 
Ca21Chr1_0270929 -0.01  0.12  0.28  0.41  0.11 -0.03 
Ca21Chr1_0270988  0.07  0.29  0.27  0.31  0.15  0.03 
Ca21Chr1_0271047 -0.38  0.20  0.14 -0.16 -0.06  0.08 
Ca21Chr1_0271106  0.22  0.24 -0.18 -0.11 -0.38  0.00 
Ca21Chr1_0271165  0.48 -0.02  0.01 -0.33  0.01  0.14 
Ca21Chr1_0271224  0.00  0.22  0.31  0.17  0.21  0.28 
Ca21Chr1_0271283 -0.01  0.15 -0.11  0.11  0.19 -0.29 
Ca21Chr1_0271342 -0.33 -0.01  0.00  0.06  0.05 -0.71 
Ca21Chr1_0271401 -0.01  0.11 -0.30  0.67 -0.12  0.47 
Ca21Chr1_0271460 -0.26 -0.08 -0.37  0.23  0.18 -0.10 
Ca21Chr1_0271519 -0.40  0.06 -0.06  0.27  0.17 -0.09 
Ca21Chr1_0271578  0.13 -0.04  0.11  0.01  0.06 -0.03 
Ca21Chr1_0271637 -0.19  0.20  0.16 -0.32 -0.10  0.13 
Ca21Chr1_0271696  0.09  0.28  0.12  0.18  0.23 -0.27 
Ca21Chr1_0271755  0.06 -0.05  0.06  0.03 -0.01  0.51 
Ca21Chr1_0271814  0.37  0.26  0.59  0.10  0.04  0.06 
Ca21Chr1_0271873 -0.00  0.09  0.20 -0.21  0.26  0.10 
Ca21Chr1_0271932  0.15  0.23  0.55  0.02  0.40  0.11  <orf19.3327
Ca21Chr1_0271991    NA    NA    NA    NA    NA    NA
Ca21Chr1_0272050 -0.41 -0.29 -1.53  0.07 -0.79  0.48 
Ca21Chr1_0272109  0.00 -0.01 -0.34 -0.17  0.02  0.36 
Ca21Chr1_0272168  0.40  0.03 -0.46 -0.24 -0.17 -0.24 
Ca21Chr1_0272227    NA    NA    NA    NA    NA    NA
Ca21Chr1_0272286 -0.21 -0.68 -0.40 -0.20  0.07  0.17 
Ca21Chr1_0272345 -0.32  0.84  0.13 -0.14 -0.13  0.67  |orf19.3328
Ca21Chr1_0272404  0.23  0.32  0.40 -0.25  0.49  0.18 
Ca21Chr1_0272463  0.07  0.40  0.24 -0.01 -0.06  0.36 
Ca21Chr1_0272522 -0.45  0.02  0.04 -0.21 -0.10  0.16 
Ca21Chr1_0272581 -0.58 -0.02 -0.07 -0.04 -0.34 -0.07 
Ca21Chr1_0272640  0.27  0.14  0.22  0.02  0.04  0.18 
Ca21Chr1_0272699  0.11  0.27  0.21 -0.19 -0.06 -0.41 
Ca21Chr1_0272758  0.23  0.14  0.42 -0.29  0.38 -0.06 
Ca21Chr1_0272817 -0.02  0.19  0.06  0.08  0.18 -0.32 
Ca21Chr1_0272876  0.14  1.45  0.41 -0.10  0.14 -0.33 
Ca21Chr1_0272935  0.23 -0.15  0.07 -0.21  0.18 -0.43 
Ca21Chr1_0272994  0.33  0.25  0.41 -0.10  0.22 -0.40 
Ca21Chr1_0273053  0.35  0.26  0.63  0.19  0.24 -0.27 
Ca21Chr1_0273112  0.39  0.53  0.86 -0.10  0.50 -0.11 
Ca21Chr1_0273171 -0.41 -0.09  0.20  0.19  0.19 -0.26 
Ca21Chr1_0273230  0.28 -0.11  0.17 -0.04  0.03  0.09 
Ca21Chr1_0273289  0.46 -0.22  0.59 -0.00  0.39 -0.24 
Ca21Chr1_0273348  0.15  0.29  0.09 -0.13 -0.05 -0.66 
Ca21Chr1_0273407 -0.00  0.46  0.13 -0.09  0.26 -0.34 
Ca21Chr1_0273466 -0.48  0.95 -0.01  0.24  0.17 -0.50 
Ca21Chr1_0273525 -0.15 -0.27  0.13 -0.34 -0.08 -0.14 
Ca21Chr1_0273584 -0.13 -0.06 -0.23 -0.14 -0.15  0.01 
Ca21Chr1_0273643 -0.23 -0.19 -0.30 -0.30  0.31  0.33 
Ca21Chr1_0273702 -0.07 -0.15 -0.18 -0.17 -0.24  0.15 
Ca21Chr1_0273761  0.17 -0.02 -0.12 -0.13  0.27 -0.22 
Ca21Chr1_0273820 -0.57 -0.28  0.50 -0.01  0.07 -0.00 
Ca21Chr1_0273879  0.12  0.91  0.58 -0.07  0.34 -0.03 
Ca21Chr1_0273938  0.06 -0.20  0.28 -0.06  0.29  0.09 
Ca21Chr1_0273997  0.36  0.16  0.21 -0.19  0.04  0.16 
Ca21Chr1_0274056  0.03 -0.11  0.35  0.01 -0.05  0.11 
Ca21Chr1_0274115  0.58  0.11  1.46  0.32  0.07  0.19 
Ca21Chr1_0274174  0.55  0.17  0.49 -0.12 -0.23 -0.12  <orf19.3328
Ca21Chr1_0274233 -0.04  0.27  0.41  0.07  0.32 -0.03 
Ca21Chr1_0274292 -0.33  0.28  0.33 -0.03  0.28  0.10 
Ca21Chr1_0274351  0.27  0.64 -0.19  0.26  0.36  0.44 
Ca21Chr1_0274410 -0.24  0.21  0.01 -0.01  0.39  0.35 
Ca21Chr1_0274469  0.00  0.22 -0.11 -0.07 -0.07  0.40 
Ca21Chr1_0274528  0.27  0.24 -0.16 -0.08  0.07  0.64 
Ca21Chr1_0274587    NA    NA    NA    NA    NA    NA
Ca21Chr1_0274646  0.20 -0.28 -0.43 -0.29 -0.28  0.36 
Ca21Chr1_0274705  0.11 -0.05 -0.19  0.03  0.33 -0.70 
Ca21Chr1_0274764  0.10 -0.05 -0.19 -0.44  0.25 -0.04 
Ca21Chr1_0274823  0.12  0.13 -0.11 -0.11 -0.09  0.36 
Ca21Chr1_0274882 -0.11  0.09 -0.10 -0.07  0.03 -0.15 
Ca21Chr1_0274941 -0.09 -0.32  0.27 -0.22 -0.26 -0.15 
Ca21Chr1_0275000 -0.01  0.06  0.24 -0.55 -0.01  0.03 
Ca21Chr1_0275059 -0.07  0.02  0.65 -0.27 -0.19  0.19 
Ca21Chr1_0275118 -0.13  0.36  0.18 -0.08  0.07  0.00 
Ca21Chr1_0275177  0.47 -0.12  0.05 -0.49  0.07  0.31 
Ca21Chr1_0275236 -0.04  0.17 -0.06 -0.20  0.16  0.09 
Ca21Chr1_0275295  0.17  0.07  0.19  0.01  0.00  0.15 
Ca21Chr1_0275354  0.23  0.08  0.15 -0.02 -0.11  0.08 
Ca21Chr1_0275413  0.09 -0.03  0.38  0.13  0.02 -0.07 
Ca21Chr1_0275472  0.85  0.14  0.53  0.11  0.08 -0.23  |orf19.3329
Ca21Chr1_0275531 -0.01  0.40  0.10 -0.13  0.06 -0.26 
Ca21Chr1_0275590  0.36  0.14  0.24 -0.38 -0.01 -0.33 
Ca21Chr1_0275649 -0.45  0.18 -0.03 -0.43  0.26  0.51 
Ca21Chr1_0275708  0.22  0.60  0.12 -0.17  0.06  0.11 
Ca21Chr1_0275767  0.15  0.10  0.22 -0.06  0.58 -0.32 
Ca21Chr1_0275826  0.28 -0.02  0.35  0.10  0.37  0.58 
Ca21Chr1_0275885  0.23  0.23 -0.14  0.41  0.06  0.07 
Ca21Chr1_0275944  0.27  0.23  0.45  0.03  0.07 -0.08 
Ca21Chr1_0276003  0.00  0.13  0.02  0.00  0.65 -0.05 
Ca21Chr1_0276062  0.23  0.12  0.23  0.39 -0.21 -0.21 
Ca21Chr1_0276121  0.38 -0.09  0.25  0.13  0.26 -0.26 
Ca21Chr1_0276180  0.07  0.03  0.38 -0.19 -0.10  0.55 
Ca21Chr1_0276239 -0.03  0.14  0.32 -0.02 -0.05 -0.11 
Ca21Chr1_0276298  0.31 -0.02  0.05 -0.49  0.34 -0.18 
Ca21Chr1_0276357  0.26  0.21  0.38  0.04 -0.05 -0.12 
Ca21Chr1_0276416  0.04 -0.04  0.32 -0.14 -0.09 -0.11 
Ca21Chr1_0276475  0.59  0.43  0.36  0.36  0.29 -0.04 
Ca21Chr1_0276534  0.12  0.07  0.27 -0.02 -0.18 -0.24 
Ca21Chr1_0276593  0.18  0.29  0.07  0.07  0.01  0.07 
Ca21Chr1_0276652 -0.22  0.65  0.33 -0.39 -0.00 -0.25 
Ca21Chr1_0276711  0.26  0.50  0.33 -0.20  0.14 -0.25 
Ca21Chr1_0276770 -0.78  0.28  0.42  0.08 -0.25 -0.43 
Ca21Chr1_0276829  0.34  0.28  0.13 -0.28  0.12 -0.48 
Ca21Chr1_0276888  1.07  0.51  0.11  0.06 -0.31 -0.06 
Ca21Chr1_0276947  0.13  0.04  0.25 -0.20  0.21 -0.08  <orf19.3329
Ca21Chr1_0277006  0.03 -0.31 -0.03 -0.33  0.19  0.14 
Ca21Chr1_0277065 -0.54 -0.44 -0.44 -0.59  0.71  0.03 
Ca21Chr1_0277124 -1.23 -0.64 -0.79 -0.34 -0.42 -0.03 
Ca21Chr1_0277183 -0.38 -0.67 -1.30 -0.84 -0.52  0.23 
Ca21Chr1_0277242 -0.46 -0.49 -1.20 -0.49  0.09 -0.01 
Ca21Chr1_0277301 -1.20 -1.02 -1.14 -0.22 -0.38  0.42 
Ca21Chr1_0277360 -0.43 -0.17 -0.77 -0.67  0.09 -0.09 
Ca21Chr1_0277419  0.31 -0.02 -0.18 -0.31  0.19  0.09 
Ca21Chr1_0277478  0.09 -0.60 -0.40 -0.04 -0.19 -0.09 
Ca21Chr1_0277537  0.29 -0.15  0.04 -0.03 -0.42 -0.10 
Ca21Chr1_0277596  1.26 -0.05  0.01  0.02 -0.19 -0.23 
Ca21Chr1_0277655 -0.75  0.10  0.27  0.21 -0.08  0.50 
Ca21Chr1_0277714  0.27  1.48  0.38  0.32 -0.01  0.06 
Ca21Chr1_0277773  0.20  0.92  0.23 -0.38 -0.41  0.06 
Ca21Chr1_0277832 -0.19  0.47 -0.12  0.01  0.04  0.07 
Ca21Chr1_0277891  0.00  0.08  0.13  0.06 -0.27  0.05 
Ca21Chr1_0277950  0.02  0.07 -0.24  0.19 -0.01  0.01 
Ca21Chr1_0278009 -0.01  0.19  0.37 -0.16  0.09  0.08 
Ca21Chr1_0278068 -0.15  0.37  0.22  0.07 -0.16  0.20 
Ca21Chr1_0278127  0.07  0.42  0.08  0.16  0.07 -0.05 
Ca21Chr1_0278186  0.51  0.10  0.00 -0.13 -0.02  0.39  |orf19.3330
Ca21Chr1_0278245  0.08  0.16 -0.10 -0.03  0.07 -0.08 
Ca21Chr1_0278304  0.10  0.37 -0.09  0.07  0.26 -0.32 
Ca21Chr1_0278363  0.37  0.45  0.50  0.07  0.31 -0.36 
Ca21Chr1_0278422  0.22  0.57  0.29  0.03  0.20 -0.10 
Ca21Chr1_0278481  0.20  0.25  0.25  0.05  0.54 -0.17 
Ca21Chr1_0278540 -0.05 -0.20  0.09 -0.29 -0.00 -0.67 
Ca21Chr1_0278599  0.26  0.13  0.39 -0.36  0.14 -0.23 
Ca21Chr1_0278658  0.20  0.12  0.36 -0.07  0.01 -0.57 
Ca21Chr1_0278717  0.18  0.25  0.54  0.44  0.04  0.19 
Ca21Chr1_0278776  0.11  0.28  0.28 -0.35  0.12 -0.00 
Ca21Chr1_0278835  0.18  0.20  0.19  0.07  0.01 -0.25 
Ca21Chr1_0278894  0.32  0.06  0.31 -0.07 -0.02 -0.04 
Ca21Chr1_0278953 -0.09  0.20  0.05 -0.02  0.06 -0.33 
Ca21Chr1_0279012  0.18  0.43  0.30  0.10  0.29 -0.10 
Ca21Chr1_0279071 -0.01  0.33 -0.14  0.27 -0.21 -0.18 
Ca21Chr1_0279130  0.28 -0.01 -0.02 -0.31 -0.05 -0.12 
Ca21Chr1_0279189  0.17  0.26 -0.17 -0.09 -0.13 -0.33 
Ca21Chr1_0279248  0.15 -0.26 -0.57 -0.22 -0.06 -0.15  <orf19.3330
Ca21Chr1_0279307 -0.43  0.03 -0.14  0.38  0.60  1.23 
Ca21Chr1_0279366 -0.69 -0.46 -0.61  0.15  0.09  0.57 
Ca21Chr1_0279425 -0.10 -0.56 -0.50 -0.27  0.00  0.04 
Ca21Chr1_0279484 -0.19 -0.26 -0.48  0.17 -0.03  0.21 
Ca21Chr1_0279543  0.07 -0.29 -0.24  0.13  0.14 -0.19 
Ca21Chr1_0279602  0.25 -0.42 -0.49  0.14 -0.35  0.34 
Ca21Chr1_0279661 -0.38 -0.05 -0.15 -0.01 -0.05  0.43 
Ca21Chr1_0279720  0.59  0.19 -0.23 -0.04 -0.23  0.56 
Ca21Chr1_0279779 -0.47  0.05 -0.24  0.09  0.15  0.32 
Ca21Chr1_0279838 -0.36 -0.06 -0.41  0.29 -0.04  0.13 
Ca21Chr1_0279897 -0.42 -0.21 -0.37 -0.10  0.42 -0.22 
Ca21Chr1_0279956 -0.67 -0.67 -0.58 -0.13 -0.00 -0.01 
Ca21Chr1_0280015 -1.03 -1.02 -0.81 -0.04 -0.32  0.06 
Ca21Chr1_0280074 -2.29 -1.16 -1.48  0.26  0.09 -0.00 
Ca21Chr1_0280133 -2.11 -0.90 -1.99  0.16 -0.27 -0.07 
Ca21Chr1_0280192 -1.14 -1.06 -1.01  0.55 -0.01  0.18 
Ca21Chr1_0280251 -0.76 -0.68 -0.52  0.42  0.17  0.37 
Ca21Chr1_0280310 -1.17 -1.50 -1.24 -0.01  0.13 -0.22 
Ca21Chr1_0280369 -0.69 -1.17 -0.75 -0.24  0.20 -0.56 
Ca21Chr1_0280428 -0.27 -0.39  0.23  0.02  0.20  0.52  |orf19.3330.3
Ca21Chr1_0280487 -0.18 -0.25 -0.18 -0.12  0.07  0.02 
Ca21Chr1_0280546  0.15 -0.27 -0.05 -0.36 -0.05  0.65  <orf19.3330.3
Ca21Chr1_0280605  0.00  0.37 -0.08 -0.23 -0.29 -0.23 
Ca21Chr1_0280664  0.49 -0.05  0.39  0.24 -0.00 -0.24 
Ca21Chr1_0280723  1.34 -0.00  0.26  0.07 -0.15  0.26 
Ca21Chr1_0280782  0.07  0.03  0.26  0.27  0.08  0.36 
Ca21Chr1_0280841 -0.33 -0.19  0.05 -0.25  0.02 -0.01 
Ca21Chr1_0280900  0.36  0.33  0.04 -0.20 -0.53  0.08 
Ca21Chr1_0280959  0.02  0.31 -0.20 -0.00  0.14  0.22 
Ca21Chr1_0281018 -0.08 -0.04  0.06  0.04 -0.24  0.34 
Ca21Chr1_0281077  0.36 -0.35  0.22 -0.17 -0.21 -0.17 
Ca21Chr1_0281136  0.24  0.31  0.08 -0.12 -0.04 -0.19 
Ca21Chr1_0281195  0.34 -0.11  0.27 -0.30 -0.02  0.07 
Ca21Chr1_0281254  0.20  0.16 -0.11 -0.35 -0.04  0.06 
Ca21Chr1_0281313  0.14  0.14 -0.16 -0.28  0.16 -0.14 
Ca21Chr1_0281372 -0.39  0.04  0.02 -0.29 -0.31  0.38 
Ca21Chr1_0281431  0.11  0.27  0.11 -0.66 -0.34  0.13 
Ca21Chr1_0281490  0.11  0.15 -0.12 -0.48 -0.12 -0.00 
Ca21Chr1_0281549  0.07  0.30  0.10 -0.54 -0.30 -0.09 
Ca21Chr1_0281608  0.04  0.04 -0.18 -0.67  0.82 -0.45 
Ca21Chr1_0281667  0.32  0.01 -0.17 -0.98 -0.49 -0.29 
Ca21Chr1_0281726 -0.12  0.00 -0.39 -0.88 -0.34 -0.06 
Ca21Chr1_0281785  0.08 -0.53 -0.27 -0.97 -0.68  0.78 
Ca21Chr1_0281844 -0.03 -0.15 -0.31 -1.37 -0.20  0.10 
Ca21Chr1_0281903 -0.40 -0.38 -0.35 -0.15 -0.41 -0.24 
Ca21Chr1_0281962 -0.34 -0.10 -0.14 -0.16 -0.23  0.03 
Ca21Chr1_0282021 -0.29 -0.18 -0.36 -0.64 -0.29  0.00 
Ca21Chr1_0282080  0.16 -0.25 -0.27  0.10 -0.19  0.00 
Ca21Chr1_0282139 -0.14 -0.10 -0.98 -0.09 -0.37  0.33 
Ca21Chr1_0282198 -0.24 -0.36 -0.72 -0.41 -0.13  0.38 
Ca21Chr1_0282257 -0.32 -0.41 -0.53 -0.01  0.22  0.22 
Ca21Chr1_0282316 -0.06  0.06 -0.28  0.31 -0.31  0.64 
Ca21Chr1_0282375 -0.06 -0.23  0.12  0.05 -0.11  0.26 
Ca21Chr1_0282434 -0.04 -0.10 -0.01 -0.04 -0.06 -0.14  orf19.3331>
Ca21Chr1_0282493  0.05 -0.01  0.30 -0.01 -0.18 -0.31 
Ca21Chr1_0282552 -0.08 -0.08  0.24 -0.02 -0.05  0.22 
Ca21Chr1_0282611  0.03 -0.04  0.02  0.17  1.00 -0.11 
Ca21Chr1_0282670 -0.01  0.12  0.24  0.02 -0.17  0.10 
Ca21Chr1_0282729  0.03  0.03  0.02  0.08  0.14  0.02 
Ca21Chr1_0282788 -0.39 -0.05  0.33  0.46  0.00 -0.28 
Ca21Chr1_0282847  0.08  0.31  0.26 -0.10  0.43 -0.41 
Ca21Chr1_0282906  0.27  0.21  0.05 -0.14  0.22 -0.36 
Ca21Chr1_0282965  0.14 -0.10  0.38 -0.53  0.27 -0.47 
Ca21Chr1_0283024  0.16  0.25  0.15  0.43  0.08  0.15 
Ca21Chr1_0283083 -0.06  0.45  0.15 -0.02  0.07 -0.22 
Ca21Chr1_0283142 -0.00  0.47  0.16  0.05 -0.02 -0.15 
Ca21Chr1_0283201  0.64  0.42  0.55 -0.29 -0.35 -0.16 
Ca21Chr1_0283260 -0.02  0.01  0.75  0.22 -0.11 -0.20 
Ca21Chr1_0283319  0.43  0.25  0.17 -0.09 -0.22  0.04 
Ca21Chr1_0283378  0.30 -0.20  0.08 -0.06 -0.48  0.71 
Ca21Chr1_0283437  0.11  0.33  0.27  0.17 -0.20  0.13 
Ca21Chr1_0283496 -0.26  0.22  0.18  0.36  0.26  0.10 
Ca21Chr1_0283555  0.05  0.15  0.58  0.18  0.02 -0.10 
Ca21Chr1_0283614  0.15  0.13  0.15  0.06  0.22 -0.27 
Ca21Chr1_0283673  0.23 -0.09 -0.13  0.17  0.33 -0.31 
Ca21Chr1_0283732  0.13 -0.10  0.08  0.03  0.32 -0.03 
Ca21Chr1_0283791  0.20  0.08  0.06  0.13 -0.04 -0.23 
Ca21Chr1_0283850  0.18  0.25  0.33  0.09  0.05 -0.12 
Ca21Chr1_0283909  1.20 -0.40  0.22  0.16  0.27 -0.18 
Ca21Chr1_0283968  0.20 -0.24  0.03  0.22  0.22 -0.21 
Ca21Chr1_0284027 -0.06  0.15  2.26  0.38 -0.13  0.21 
Ca21Chr1_0284086  0.29  0.36 -0.23  0.17  0.02  0.25 
Ca21Chr1_0284145 -0.38  0.31 -0.12  0.23 -0.27  0.29  orf19.3331|
Ca21Chr1_0284204  0.76 -0.05 -0.51 -0.05 -0.52  0.58 
Ca21Chr1_0284263 -0.29  0.05 -0.50 -0.03  0.97  0.28 
Ca21Chr1_0284322 -0.25  0.20 -0.05 -0.12  0.04  0.45 
Ca21Chr1_0284381 -0.15 -0.05 -1.11  0.01 -0.11  0.13 
Ca21Chr1_0284440  0.16 -0.10 -0.62  0.20  0.22  0.00 
Ca21Chr1_0284499  0.09 -0.56 -0.12  0.26  0.15  0.05 
Ca21Chr1_0284558 -0.07 -0.38 -0.43  0.10  0.42  0.09 
Ca21Chr1_0284617  0.23 -0.18  0.04  0.32  0.06 -0.35  orf19.3332>
Ca21Chr1_0284676 -0.11  0.56  0.19 -0.08  0.05 -0.27 
Ca21Chr1_0284735  0.25  0.16  0.45 -0.32 -0.12 -0.20 
Ca21Chr1_0284794  0.27  0.12  0.52  0.12 -0.07 -0.21 
Ca21Chr1_0284853  0.14  0.07  0.32  0.19 -0.34  0.01 
Ca21Chr1_0284912  0.29  0.08  0.27 -0.15  0.22 -0.14 
Ca21Chr1_0284971  0.20 -0.23 -0.04 -0.29 -0.20  0.08 
Ca21Chr1_0285030  0.42 -0.02  0.53 -0.15  0.07  0.01 
Ca21Chr1_0285089  0.45  0.26  0.36  0.19  0.13 -0.25 
Ca21Chr1_0285148  0.49  0.08  0.58 -0.03 -0.03  0.15 
Ca21Chr1_0285207  0.42  0.04  0.46  0.12  0.06 -0.59 
Ca21Chr1_0285266 -0.22  0.10  0.35  0.07  0.13 -0.38 
Ca21Chr1_0285325  0.20  0.38  0.18 -0.49  0.13 -0.25 
Ca21Chr1_0285384  0.40  0.42  0.05 -0.12  0.15 -0.21 
Ca21Chr1_0285443  0.34 -0.26  0.26 -0.28  0.01 -0.20 
Ca21Chr1_0285502  0.76  0.10  0.36  0.17 -0.15  0.64 
Ca21Chr1_0285561 -0.10 -0.20  0.16  0.40  0.21 -0.51 
Ca21Chr1_0285620  0.03  1.16  0.04 -0.06 -0.33 -0.40 
Ca21Chr1_0285679  0.43  0.46  0.55  0.12 -0.10 -0.14 
Ca21Chr1_0285738  0.06  0.39  0.56  0.17  0.13 -0.33 
Ca21Chr1_0285797  0.22  0.36 -0.08 -0.01  0.12 -0.49 
Ca21Chr1_0285856  0.05  0.45  0.38 -0.09  0.15 -0.40 
Ca21Chr1_0285915  0.38  0.18  0.27  0.32  0.27 -0.05 
Ca21Chr1_0285974  0.10  0.43  0.31  0.08 -0.13  0.12 
Ca21Chr1_0286033  0.11  0.06  0.29  0.20 -0.19 -0.06 
Ca21Chr1_0286092  0.12  0.36  0.30  0.44  0.44 -0.46 
Ca21Chr1_0286151  0.35  0.28  0.43  0.18  0.04  0.42 
Ca21Chr1_0286210  0.21  0.16  0.28  0.08  0.01 -0.13 
Ca21Chr1_0286269 -0.18 -0.16  0.27  0.17 -0.14 -0.38 
Ca21Chr1_0286328  0.22  0.09  0.35  0.38  0.11 -0.16 
Ca21Chr1_0286387  1.01  0.13  0.50  1.01  0.32 -0.34 
Ca21Chr1_0286446  0.15  0.23  0.53  0.53 -0.07 -0.06 
Ca21Chr1_0286505  0.15 -0.11  0.23  0.26 -0.26  0.60 
Ca21Chr1_0286564 -0.11  0.03  0.22  0.02  0.14  0.05 
Ca21Chr1_0286623  0.14  0.11  0.57  0.35  0.21 -0.02 
Ca21Chr1_0286682  0.12  0.26  0.53 -0.04  0.25 -0.11 
Ca21Chr1_0286741 -0.02  0.09  0.37  0.14  0.63 -0.23 
Ca21Chr1_0286800  0.39  0.04  0.19 -0.13  0.28 -0.17 
Ca21Chr1_0286859 -0.02  0.39  0.54 -0.07  0.18 -0.65 
Ca21Chr1_0286918  0.30  0.14  0.24  0.06  0.30 -0.40 
Ca21Chr1_0286977  0.26  0.30  0.57  0.09 -0.21 -0.24 
Ca21Chr1_0287036  0.14  0.05  0.41  0.18  0.04 -0.37 
Ca21Chr1_0287095  0.24  0.05  0.51  0.34  0.30 -0.70 
Ca21Chr1_0287154  0.37  0.26  0.55  0.19  0.25  0.15 
Ca21Chr1_0287213  0.49 -0.05  0.44  0.05  0.18 -0.10 
Ca21Chr1_0287272  0.20  0.06  0.34 -0.07  0.10 -0.04 
Ca21Chr1_0287331  0.03  0.04  0.28  0.11  0.30  0.03 
Ca21Chr1_0287390 -0.06  0.08  0.15 -0.31  0.39  0.61 
Ca21Chr1_0287449  0.15  0.21  0.56  0.19  0.26  0.27 
Ca21Chr1_0287508  0.07  0.07  0.12 -0.50  0.16 -0.09 
Ca21Chr1_0287567  0.08  0.30  0.09 -0.41  0.02  0.49 
Ca21Chr1_0287626 -0.23  0.39  0.16  0.10 -0.06  0.39  orf19.3332|
Ca21Chr1_0287685 -0.15  0.46 -0.08 -0.11 -0.17  0.19 
Ca21Chr1_0287744 -0.22  0.52 -0.01 -0.14  0.07 -0.21 
Ca21Chr1_0287803 -0.40  0.41 -0.12 -0.48  0.09 -0.05 
Ca21Chr1_0287862 -0.16 -0.11 -0.14  0.06 -0.41 -0.42 
Ca21Chr1_0287921 -0.24 -0.25 -0.36 -0.35  0.15 -0.24 
Ca21Chr1_0287980  0.52 -0.54 -1.29  0.03 -0.07 -0.34 
Ca21Chr1_0288039 -0.54 -0.71 -0.72  0.45 -0.18 -0.39  orf19.3333>
Ca21Chr1_0288098 -0.47 -0.50 -0.63 -0.20  1.07 -0.27 
Ca21Chr1_0288157 -0.35 -0.02 -0.54 -0.15  0.08 -0.49 
Ca21Chr1_0288216 -0.11 -0.23 -0.32 -0.07  0.05 -0.39 
Ca21Chr1_0288275  0.05 -0.18 -0.09  0.23  0.23 -0.12 
Ca21Chr1_0288334  0.14  0.43 -0.03  0.22 -0.08 -0.34 
Ca21Chr1_0288393  0.08 -0.43  0.22  0.02  0.30 -0.64 
Ca21Chr1_0288452  0.47  0.00  0.43  0.23 -0.25 -0.61 
Ca21Chr1_0288511  0.22 -0.01  0.21 -0.04  0.27 -0.58 
Ca21Chr1_0288570  0.12 -0.05  0.45 -0.24  0.06 -0.33 
Ca21Chr1_0288629  0.51  0.10  0.43 -0.39  0.12 -0.34 
Ca21Chr1_0288688  0.01 -0.14  0.26  0.30 -0.15 -0.24 
Ca21Chr1_0288747 -0.07 -0.10 -0.08  0.80  0.06 -0.28 
Ca21Chr1_0288806 -0.11 -0.77 -0.05 -0.14 -0.04 -0.17 
Ca21Chr1_0288865 -0.96  0.20  0.07 -0.17 -0.49  0.38 
Ca21Chr1_0288924 -0.00  0.01  0.29  0.32  0.20 -0.32 
Ca21Chr1_0288983 -0.19  0.15 -0.27 -0.11  0.44 -0.13 
Ca21Chr1_0289042  0.02 -0.07 -0.06 -0.19 -0.22 -0.33 
Ca21Chr1_0289101  0.31 -0.08 -0.01 -0.22  0.28 -0.10 
Ca21Chr1_0289160 -0.19  0.14 -0.32  0.21 -0.44 -0.48 
Ca21Chr1_0289219 -0.16 -0.04  0.07  0.29 -0.24 -0.39 
Ca21Chr1_0289278 -0.21  0.03  0.00  0.40 -0.12  0.01 
Ca21Chr1_0289337 -0.58 -0.39  0.40 -0.20 -0.14 -0.11  orf19.3333|
Ca21Chr1_0289396 -0.17  0.16  0.05  0.15  0.15 -0.00 
Ca21Chr1_0289455 -0.23  0.39 -0.05  0.03 -0.34 -0.42 
Ca21Chr1_0289514  0.63  0.04  0.58 -0.01 -0.15 -1.69 
Ca21Chr1_0289573  0.04  0.06  0.05 -0.22 -0.01 -0.37 
Ca21Chr1_0289632  2.04 -0.12 -0.36 -0.39  0.02 -0.30 
Ca21Chr1_0289691  0.63  0.04  0.17 -0.24 -0.03  0.02  |orf19.3334
Ca21Chr1_0289750  0.95 -0.08  0.25  0.10  0.20 -0.32 
Ca21Chr1_0289809  0.69  0.27  0.42  0.06  0.33 -0.26 
Ca21Chr1_0289868  0.65  0.27  0.43  0.50  0.32 -0.34 
Ca21Chr1_0289927  0.40  0.32  0.49  0.02  0.21 -0.09 
Ca21Chr1_0289986  0.62  0.14  0.61  0.19 -0.07 -0.24 
Ca21Chr1_0290045  0.92  0.26  0.32  0.10  0.04  0.07 
Ca21Chr1_0290104  0.70  0.53  0.26  0.31  0.22  0.19 
Ca21Chr1_0290163  0.82  0.22  2.71  0.01  0.31  0.15 
Ca21Chr1_0290222  0.44 -0.15  0.03  0.13  0.27 -0.21 
Ca21Chr1_0290281  0.44 -0.00  0.12  0.09  0.20  0.00 
Ca21Chr1_0290340  0.90  0.24  0.15 -0.01  0.30  0.13 
Ca21Chr1_0290399  1.15 -0.13 -0.34 -0.29  0.08  0.26 
Ca21Chr1_0290458 -0.33 -0.53 -1.44  0.16  0.23  0.15  <orf19.3334
Ca21Chr1_0290517 -0.44 -0.66 -1.18  0.06  0.02  0.42 
Ca21Chr1_0290576    NA    NA    NA    NA    NA    NA
Ca21Chr1_0290635 -0.56 -1.15 -1.99 -0.02 -0.03  0.53 
Ca21Chr1_0290694 -0.74 -0.94 -1.41 -0.01 -0.51  0.06 
Ca21Chr1_0290753 -0.46 -0.65 -1.13 -0.14  0.21  0.62 
Ca21Chr1_0290812 -0.44 -0.59 -1.17  0.02 -0.07  0.10 
Ca21Chr1_0290871 -0.32 -0.74 -0.85 -0.41 -0.17  0.28 
Ca21Chr1_0290930 -0.62 -0.10 -0.65 -0.14  0.06  0.29 
Ca21Chr1_0290989 -0.28  0.14 -1.25 -0.24 -0.28 -0.16 
Ca21Chr1_0291048 -0.10 -0.56 -1.05 -0.41 -0.13  0.23 
Ca21Chr1_0291107 -0.34 -0.73 -0.21 -0.21 -0.05 -0.14 
Ca21Chr1_0291166 -0.47 -0.68 -1.08 -0.37 -0.35 -0.38 
Ca21Chr1_0291225 -0.68 -0.72 -1.25 -0.12 -0.08 -0.11 
Ca21Chr1_0291284 -0.43 -0.57 -0.75  0.19 -0.05 -0.19 
Ca21Chr1_0291343 -0.87 -0.53 -1.12  0.17 -0.06  0.18 
Ca21Chr1_0291402 -0.35 -0.69 -0.74 -0.03 -0.24 -0.26 
Ca21Chr1_0291461 -0.11 -0.28 -0.68 -0.12 -0.09  0.01 
Ca21Chr1_0291520  0.03 -0.44 -0.88 -0.32 -0.03  0.20 
Ca21Chr1_0291579 -0.24 -0.12 -0.27  0.29  0.18 -0.29 
Ca21Chr1_0291638  0.66 -0.30 -0.15 -0.17 -0.32  0.46 
Ca21Chr1_0291697  0.54  0.06 -0.07  0.05 -0.39 -0.10 
Ca21Chr1_0291756  0.47 -0.05  0.22 -0.20 -0.25 -0.53 
Ca21Chr1_0291815  1.17 -0.23  0.43 -0.21 -0.05 -0.21  orf19.3335>
Ca21Chr1_0291874  0.06 -0.04  0.53  0.17  0.10  0.19 
Ca21Chr1_0291933  0.10 -0.32  0.21  0.20 -0.05 -0.06 
Ca21Chr1_0291992  0.20  0.06  0.19 -0.24  0.19 -0.17 
Ca21Chr1_0292051 -0.23  0.15  0.26 -0.05 -0.01  0.06 
Ca21Chr1_0292110 -0.18  0.24  0.30 -0.15  0.07 -0.12 
Ca21Chr1_0292169 -0.22  0.33  0.28  0.10  0.09 -0.24 
Ca21Chr1_0292228  0.47  0.42  0.21  0.07  0.33 -0.28 
Ca21Chr1_0292287  0.06  0.36  0.62  0.21 -0.10  0.08 
Ca21Chr1_0292346  0.13  0.28  0.15  0.04  0.28 -0.39 
Ca21Chr1_0292405 -0.20  0.07 -0.03 -0.08  0.05  0.17  orf19.3335|
Ca21Chr1_0292464 -0.28 -0.03  0.13 -0.21  0.04 -0.22 
Ca21Chr1_0292523 -0.41  0.09 -0.02 -0.18  0.05  0.31 
Ca21Chr1_0292582 -0.02  0.36 -0.32 -0.17  0.10  0.00 
Ca21Chr1_0292641  0.25  0.64  0.24 -0.01 -0.10  0.20 
Ca21Chr1_0292700 -0.06  0.21  0.30  0.19 -0.13  0.29 
Ca21Chr1_0292759  0.16  0.36  0.08 -0.01 -0.13 -0.37 
Ca21Chr1_0292818 -0.22 -0.01 -0.14 -0.17 -0.16  0.36 
Ca21Chr1_0292877  0.09  0.21 -0.59  0.07 -0.03  0.16 
Ca21Chr1_0292936 -0.54 -0.35 -0.66 -0.31 -0.25  0.25 
Ca21Chr1_0292995 -0.05 -0.16 -0.58 -0.14 -0.24  0.05 
Ca21Chr1_0293054 -0.08  0.16 -0.29  0.21  0.04 -0.09 
Ca21Chr1_0293113  0.16 -0.35 -0.34 -0.01  0.06 -0.14 
Ca21Chr1_0293172  0.02 -0.33 -0.26  0.16 -0.29  0.05 
Ca21Chr1_0293231 -0.13 -0.60 -0.42  0.07  0.21 -0.53 
Ca21Chr1_0293290  0.10 -0.22 -0.68  0.12 -0.43  0.42 
Ca21Chr1_0293349 -0.14 -0.56 -0.49  0.17 -0.23  0.01 
Ca21Chr1_0293408 -0.26 -0.32 -0.67  0.18 -0.27 -0.16 
Ca21Chr1_0293467 -0.50  0.64 -0.54  0.33  0.07  0.07 
Ca21Chr1_0293526 -0.17 -0.53 -0.66  0.02 -0.35 -0.06 
Ca21Chr1_0293585 -0.33 -0.16 -0.45  0.16 -0.51  0.15 
Ca21Chr1_0293644 -0.10 -0.79 -1.02 -0.09 -0.06  0.33 
Ca21Chr1_0293703 -0.43 -0.95 -0.39  0.21 -0.04  0.35 
Ca21Chr1_0293762 -0.44 -0.94 -0.93 -0.24 -0.28  0.76 
Ca21Chr1_0293821 -0.66 -0.87 -0.74 -0.69  0.03  0.74 
Ca21Chr1_0293880 -0.76 -0.56 -1.03  0.01  0.09  0.18 
Ca21Chr1_0293939 -0.63 -0.62 -0.54  0.14  0.01  0.24 
Ca21Chr1_0293998 -0.48 -0.17 -0.66 -0.51 -0.10 -0.32 
Ca21Chr1_0294057 -1.19 -0.46 -0.57 -0.49  0.04  0.08 
Ca21Chr1_0294116 -0.49 -0.47 -0.46 -0.35 -0.32 -0.10 
Ca21Chr1_0294175 -0.28 -0.17 -0.45 -0.41 -0.43 -0.02 
Ca21Chr1_0294234 -0.21 -0.52 -0.19 -0.46 -0.25 -0.06 
Ca21Chr1_0294293 -0.09 -0.30 -0.11 -0.31  0.13 -0.26 
Ca21Chr1_0294352 -0.08  0.18 -0.10 -0.03 -0.08 -0.07 
Ca21Chr1_0294411  0.16  0.12 -0.09 -0.47 -0.17  0.01 
Ca21Chr1_0294470  0.22  0.05 -0.19 -0.18 -0.41  0.67 
Ca21Chr1_0294529  0.33 -0.01  0.20 -0.03 -0.22 -0.63 
Ca21Chr1_0294588  0.12  0.31  0.10 -0.13  0.06  0.37 
Ca21Chr1_0294647  0.16  0.45  0.36  0.06 -0.36 -0.70 
Ca21Chr1_0294706  0.44 -0.01  0.08  0.43 -0.27  0.55 
Ca21Chr1_0294765  0.20 -0.17 -0.09  0.44 -0.16  0.05 
Ca21Chr1_0294824 -0.08 -0.03  0.12  0.01 -0.19  0.08 
Ca21Chr1_0294883 -0.15 -0.01  0.06  0.05 -0.34  0.09 
Ca21Chr1_0294942 -0.12 -0.20  0.18  0.21 -0.05 -0.06 
Ca21Chr1_0295001 -0.20 -0.22  0.60  0.22 -0.20 -0.48 
Ca21Chr1_0295060  0.10  0.06  0.33  0.05 -0.08 -0.46 
Ca21Chr1_0295119 -0.12  0.08  0.38 -0.36 -0.41 -0.34 
Ca21Chr1_0295178  0.12  0.01  0.55 -0.37  0.14 -0.01 
Ca21Chr1_0295237  0.12 -0.25  0.16  0.05 -0.01 -0.05 
Ca21Chr1_0295296  0.12 -0.11  0.52  0.04 -0.16 -0.31 
Ca21Chr1_0295355  1.01  0.31 -0.11 -0.23  0.22 -0.03 
Ca21Chr1_0295414  0.19  0.11 -0.42  0.05 -0.09  0.16 
Ca21Chr1_0295473 -0.28  0.23 -0.21 -0.10  0.03  0.27 
Ca21Chr1_0295532 -0.69  0.31 -0.15  0.50 -0.08 -0.41 
Ca21Chr1_0295591 -0.48 -0.08 -0.82  0.12  0.13  0.51 
Ca21Chr1_0295650 -0.87 -0.08 -1.03  0.47  0.09  0.34 
Ca21Chr1_0295709 -0.85 -0.69 -1.79 -0.28 -0.09  0.62 
Ca21Chr1_0295768 -1.15 -1.23 -1.41 -0.01  0.09 -0.16 
Ca21Chr1_0295827 -1.00 -0.87 -0.92 -0.47  0.22 -0.01 
Ca21Chr1_0295886 -0.95 -0.91 -0.50 -0.01  0.17 -0.59 
Ca21Chr1_0295945 -0.49 -0.30 -0.05 -0.54  0.06 -0.32 
Ca21Chr1_0296004 -0.24 -0.23 -0.25  0.08  0.17  0.05  orf19.3336>
Ca21Chr1_0296063 -0.05 -0.09  0.01  0.08  0.19 -0.44 
Ca21Chr1_0296122 -0.08 -0.03  0.12  0.25  0.17 -0.29 
Ca21Chr1_0296181 -0.07  0.02 -0.03 -0.13 -0.93 -0.74 
Ca21Chr1_0296240  0.22 -0.10  0.10  0.13 -0.14 -0.07 
Ca21Chr1_0296299 -0.73  0.02 -0.05 -0.18  0.04 -0.27 
Ca21Chr1_0296358 -0.35  0.24  0.11  0.11 -0.17  0.00 
Ca21Chr1_0296417 -0.04  0.22  0.43  0.38 -0.35 -0.13 
Ca21Chr1_0296476  0.10  0.17  0.28  0.24 -0.25 -0.22  orf19.3336|
Ca21Chr1_0296535  0.38  0.12  0.04  0.07  0.13 -0.14 
Ca21Chr1_0296594 -0.08  0.05  0.11  0.78  0.23  0.10 
Ca21Chr1_0296653 -0.25 -0.12  0.17 -0.02  0.03 -0.24 
Ca21Chr1_0296712  0.41  0.15 -0.47  0.61 -0.19 -0.20 
Ca21Chr1_0296771  0.01  0.16  0.03  0.06 -0.07 -0.03 
Ca21Chr1_0296830  0.07  0.02  0.02 -0.02  0.17 -0.03 
Ca21Chr1_0296889  0.14 -0.22 -0.11  0.35  1.07  0.11 
Ca21Chr1_0296948 -0.10 -0.21 -0.20  0.34  0.01  0.34 
Ca21Chr1_0297007  0.03  0.21  0.18 -0.16 -0.40  0.06 
Ca21Chr1_0297066 -0.55  0.00 -0.06 -0.10 -0.37 -0.02 
Ca21Chr1_0297125 -0.03  0.07 -0.15  0.24  0.06 -0.09 
Ca21Chr1_0297184 -0.78 -0.00  0.25  0.03  0.17 -0.13 
Ca21Chr1_0297243 -0.04  0.10  0.29 -0.05  0.09  0.22 
Ca21Chr1_0297302  0.16  0.17  0.06  0.13 -0.04 -0.04 
Ca21Chr1_0297361  0.34  0.37 -0.01  0.24 -0.34  0.20 
Ca21Chr1_0297420  0.57  0.15  0.22 -0.11 -0.11 -0.31 
Ca21Chr1_0297479  0.05  0.21  0.25  0.10  0.03 -0.00 
Ca21Chr1_0297538 -0.09  0.03  0.47 -0.13  0.33  0.04 
Ca21Chr1_0297597  0.57 -0.05 -0.35 -0.16 -0.15 -0.01 
Ca21Chr1_0297656 -0.07  0.40  0.74  0.08 -0.23  1.18 
Ca21Chr1_0297715  0.42 -0.06  0.19  0.41  0.14  0.60 
Ca21Chr1_0297774  0.07 -0.15  0.48 -0.25  0.05 -0.79 
Ca21Chr1_0297833 -0.28  0.12 -0.38 -0.20  0.04  0.21 
Ca21Chr1_0297892 -0.15  0.24  0.15  0.20 -0.14  0.00 
Ca21Chr1_0297951 -0.40  0.05  0.84  0.35 -0.04  0.11 
Ca21Chr1_0298010  0.33  0.04  0.36  0.15 -0.61  0.99 
Ca21Chr1_0298069 -0.10 -0.14 -0.07  0.38 -0.26  0.47 
Ca21Chr1_0298128  0.09  0.42  0.44  0.22 -0.02  1.72 
Ca21Chr1_0298187 -0.04  0.46 -0.41  0.12  0.02  1.42 
Ca21Chr1_0298246  0.03  0.28 -0.22  0.12 -0.06  0.97 
Ca21Chr1_0298305  0.63  0.30  0.03  0.04  0.08 -0.27 
Ca21Chr1_0298364  0.10  0.18 -0.21 -0.12  0.09  0.48 
Ca21Chr1_0298423  0.62  0.02  0.10  0.77  0.19  0.35 
Ca21Chr1_0298482 -0.05 -0.00  0.09 -0.09  0.07  0.03 
Ca21Chr1_0298541  0.03  0.28 -0.16 -0.18 -0.14 -0.19 
Ca21Chr1_0298600 -0.09 -0.18 -0.17 -0.34 -0.95 -0.18 
Ca21Chr1_0298659 -0.33 -0.08 -0.10  0.60  0.14  0.68 
Ca21Chr1_0298718 -0.42 -0.14 -0.30 -0.02 -0.23  0.19 
Ca21Chr1_0298777 -0.41 -0.37 -0.46 -0.68  0.18  0.22 
Ca21Chr1_0298836 -0.87 -0.33 -0.17 -0.95 -0.37  0.21 
Ca21Chr1_0298895 -0.53 -0.51  0.13 -0.38  0.09 -0.17 
Ca21Chr1_0298954 -1.23 -0.20 -0.37 -0.66 -0.19  0.19 
Ca21Chr1_0299013 -1.08 -0.16  0.04 -0.60 -0.28  0.61 
Ca21Chr1_0299072 -1.77  0.03  0.14 -0.24 -0.18  0.46 
Ca21Chr1_0299131 -0.55  0.03  0.42 -0.23 -0.07  0.48 
Ca21Chr1_0299190 -0.28 -0.00  0.19 -0.53  0.26  0.33 
Ca21Chr1_0299249  0.40  0.76  0.02 -0.29  0.24  0.33 
Ca21Chr1_0299308    NA    NA    NA    NA    NA    NA
Ca21Chr1_0299367 -0.63 -0.03  0.01  0.17  0.26  0.28 
Ca21Chr1_0299426 -0.33 -0.01  0.09  0.17 -0.05  0.41 
Ca21Chr1_0299485  0.09 -0.20  0.08  0.18 -0.01  0.21 
Ca21Chr1_0299544  0.73 -0.18  0.23  0.89  0.15  0.39 
Ca21Chr1_0299603 -0.01 -0.38  0.35  0.13  0.07  0.10  orf19.3337>
Ca21Chr1_0299662  0.14 -0.20  0.29  0.16 -0.32 -0.22 
Ca21Chr1_0299721 -0.08 -0.11  0.13  0.50  0.08  0.05 
Ca21Chr1_0299780  0.21 -0.01  0.45  0.18 -0.23  0.28 
Ca21Chr1_0299839  0.60  0.18  0.42 -0.01 -0.02 -0.27 
Ca21Chr1_0299898 -0.01  0.16  0.38 -0.07  0.71  0.03 
Ca21Chr1_0299957 -3.42  0.09  0.57  0.04  0.03  0.00 
Ca21Chr1_0300016  0.26  0.41  0.60  0.12  0.26 -0.27 
Ca21Chr1_0300075  0.32  0.07  0.45 -0.50  0.03 -0.02 
Ca21Chr1_0300134  0.80 -0.07  0.42  0.24 -0.09  0.28 
Ca21Chr1_0300193 -0.17  0.36  0.54 -0.11  0.12 -0.21 
Ca21Chr1_0300252  0.12  0.03  0.11 -0.10  0.18 -0.01 
Ca21Chr1_0300311  0.09 -0.18  0.42  0.00 -0.22 -0.00 
Ca21Chr1_0300370  0.13  0.15  0.29  0.00  0.03 -0.11  orf19.3337| orf19.3338>
Ca21Chr1_0300429  0.40  0.14  0.24  0.02  0.15 -0.05 
Ca21Chr1_0300488  0.18  0.14 -0.02 -0.11  0.08  0.25 
Ca21Chr1_0300547  1.17  0.04  0.31 -0.22  0.08 -0.01 
Ca21Chr1_0300606  0.22  0.12  0.15  0.36  0.28  0.24 
Ca21Chr1_0300665  0.30  0.02  0.23  0.08 -0.13 -0.09 
Ca21Chr1_0300724 -0.00 -0.34  0.28 -0.00  0.23 -0.06 
Ca21Chr1_0300783  0.06 -0.26  0.08  0.64  0.08  0.19 
Ca21Chr1_0300842  0.14 -0.31  0.34  0.08  0.02  0.24 
Ca21Chr1_0300901  0.17  0.11  0.18  0.11  0.14  0.35 
Ca21Chr1_0300960  0.02 -0.03  0.61  0.41  0.37  0.01 
Ca21Chr1_0301019  0.13  0.84  0.21  0.95 -0.02  0.03 
Ca21Chr1_0301078  0.21  0.02  0.12 -0.24 -0.15 -0.17 
Ca21Chr1_0301137  0.21  0.14  0.23  0.29 -0.26 -0.41 
Ca21Chr1_0301196  0.47  0.58  0.05  0.06 -0.00  0.09 
Ca21Chr1_0301255 -0.01  0.03  0.01  0.83 -0.13  0.31 
Ca21Chr1_0301314 -0.21 -0.06  0.16 -0.08  0.21 -0.26 
Ca21Chr1_0301373  0.04  0.11  0.19  0.17 -0.04 -0.53 
Ca21Chr1_0301432  0.34  0.37  0.33 -0.16  0.22 -0.34 
Ca21Chr1_0301491 -0.16  0.35  0.19  0.08 -0.13 -0.33 
Ca21Chr1_0301550  0.02  0.22  0.57 -0.18 -0.13 -0.12 
Ca21Chr1_0301609  0.06  0.28  0.36  0.06 -0.19  0.37 
Ca21Chr1_0301668 -0.10  0.12  0.53 -0.03  1.02 -0.12 
Ca21Chr1_0301727  0.51  0.31  0.04 -0.28  0.12  0.35  orf19.3338|
Ca21Chr1_0301786  0.02 -0.04  1.28  0.19  0.59  0.57 
Ca21Chr1_0301845  0.05 -0.02  0.33 -0.55  0.37  0.19 
Ca21Chr1_0301904  1.25 -0.17 -0.39  0.16  0.34 -0.11 
Ca21Chr1_0301963 -0.08 -0.05 -0.23 -0.45 -0.08  0.53 
Ca21Chr1_0302022 -0.63  0.09 -0.42 -0.72  0.05 -0.13 
Ca21Chr1_0302081 -0.02 -0.24 -0.44 -0.10  0.42 -0.16 
Ca21Chr1_0302140 -0.67  0.10 -0.61 -0.11 -0.06 -0.11 
Ca21Chr1_0302199 -0.33 -0.22 -0.86 -0.26  0.18 -0.19 
Ca21Chr1_0302258 -1.06 -0.32 -1.30 -0.37  0.60 -0.34 
Ca21Chr1_0302317 -1.66 -0.49 -1.51 -0.10 -0.07 -0.06 
Ca21Chr1_0302376 -1.59 -1.28 -1.58 -0.18 -0.07 -0.29 
Ca21Chr1_0302435 -2.02 -1.19 -0.99 -0.07 -0.08 -0.16 
Ca21Chr1_0302494 -1.08 -1.06 -1.71 -0.03 -0.23 -0.27 
Ca21Chr1_0302553 -1.76 -1.11 -1.84 -0.06 -0.17 -0.40 
Ca21Chr1_0302612    NA    NA    NA    NA    NA    NA
Ca21Chr1_0302671 -0.96 -0.67 -1.34 -0.60 -0.19 -0.03 
Ca21Chr1_0302730 -0.48 -0.68 -1.08 -0.48  0.03  0.18 
Ca21Chr1_0302789 -0.56 -0.62 -1.09 -0.23 -0.08  0.27 
Ca21Chr1_0302848    NA    NA    NA    NA    NA    NA
Ca21Chr1_0302907 -0.31 -0.17 -0.77 -0.33  0.14 -0.83 
Ca21Chr1_0302966 -0.43 -0.11 -0.68 -0.35 -0.15 -0.52 
Ca21Chr1_0303025  0.92  0.38 -1.08 -0.28  0.18 -0.43 
Ca21Chr1_0303084 -0.28  0.43  0.02  0.08 -0.18  0.49 
Ca21Chr1_0303143  0.74  0.25  0.27  0.21  0.58 -1.08 
Ca21Chr1_0303202 -0.29  0.17 -0.83  0.01  0.62  0.09 
Ca21Chr1_0303261 -0.01  0.20 -0.02 -0.21  0.65  0.01 
Ca21Chr1_0303320  0.91  0.04  0.15  0.37 -0.28  0.08 
Ca21Chr1_0303379  0.11  0.25  0.45  0.25  0.02  0.60 
Ca21Chr1_0303438  0.05  0.06  0.12 -0.35 -0.05  0.46 
Ca21Chr1_0303497 -0.09  0.05  0.65 -0.03 -0.13 -0.23 
Ca21Chr1_0303556 -0.19 -0.10  0.07 -0.29 -0.03 -0.44 
Ca21Chr1_0303615  0.09  0.37  0.25  0.39 -0.05 -0.07 
Ca21Chr1_0303674  0.29  0.22  0.24  0.22 -0.49 -0.11 
Ca21Chr1_0303733  0.32  0.42  0.42 -0.14 -0.13  0.01 
Ca21Chr1_0303792 -0.16  0.19  0.20 -0.13  0.10  0.26 
Ca21Chr1_0303851  0.06  0.10 -0.10 -0.15  0.12  0.26 
Ca21Chr1_0303910  0.26 -0.07  0.53  0.28 -0.06  1.17 
Ca21Chr1_0303969 -0.16  0.16 -0.00 -0.06 -0.02  0.33 
Ca21Chr1_0304028  0.65 -0.21 -0.10 -0.07  0.85  1.52 
Ca21Chr1_0304087 -0.03 -0.13  0.22  0.03 -0.07 -0.18 
Ca21Chr1_0304146  0.31  0.08  0.14 -0.08 -0.16 -0.09 
Ca21Chr1_0304205 -0.02  0.62  0.52  0.27  0.01  0.03 
Ca21Chr1_0304264  0.00 -0.03  0.28  0.14 -0.21  0.39 
Ca21Chr1_0304323 -0.41 -0.01  0.19  0.18 -0.33  0.07 
Ca21Chr1_0304382  0.00  0.28  0.13  0.07 -0.15  0.06 
Ca21Chr1_0304441  0.14  0.21  0.02  0.06 -0.03  0.06 
Ca21Chr1_0304500  0.27  0.17  0.08  0.01  0.12  0.21 
Ca21Chr1_0304559  0.32  0.37  0.25 -0.05  0.08  0.08 
Ca21Chr1_0304618  0.14  0.00  0.35 -0.07 -0.18  0.36 
Ca21Chr1_0304677 -0.07  0.38  0.26 -0.05  0.06  0.08 
Ca21Chr1_0304736 -0.05  0.30  0.21  0.20 -0.01 -0.14 
Ca21Chr1_0304795 -0.02  0.11  0.67 -0.05 -0.14 -0.28 
Ca21Chr1_0304854  0.05  0.43  0.27  0.12 -0.29  0.17 
Ca21Chr1_0304913 -0.23 -0.09  0.25  0.04 -0.12  0.01 
Ca21Chr1_0304972  0.18  0.15  0.16  0.00 -0.21  0.38 
Ca21Chr1_0305031  0.27  0.15 -0.01  0.08 -0.39  0.34 
Ca21Chr1_0305090  0.11 -0.10  0.13  0.06 -0.16  0.31 
Ca21Chr1_0305149  0.69 -0.22  0.09 -0.22 -0.04 -0.03 
Ca21Chr1_0305208  1.02  0.16  0.18 -0.22 -0.08  0.57 
Ca21Chr1_0305267  0.32  0.19 -0.18  0.13  0.13  0.08  |orf19.3340
Ca21Chr1_0305326  0.36  0.06 -0.12 -0.53  0.00  0.13 
Ca21Chr1_0305385  0.19 -0.07  0.58 -0.00 -0.04 -0.04 
Ca21Chr1_0305444  0.59  0.16  0.53  0.35  0.71 -0.06 
Ca21Chr1_0305503  0.61 -0.02  0.11 -0.33 -0.29 -0.44 
Ca21Chr1_0305562  0.56  0.08  0.18  0.06  0.05 -0.04 
Ca21Chr1_0305621  0.45 -0.31  0.11 -0.17 -0.20 -0.07 
Ca21Chr1_0305680  0.78  0.15  0.38  0.47  0.39  0.80 
Ca21Chr1_0305739  0.60  0.20  0.07  0.06  0.03  0.62 
Ca21Chr1_0305798  0.41  0.07  0.17  0.15 -0.03 -0.25 
Ca21Chr1_0305857  0.53  0.08  0.04  0.01  0.05 -0.39 
Ca21Chr1_0305916  0.42  0.15 -0.11 -0.18 -0.27 -0.01 
Ca21Chr1_0305975  0.40  0.24 -0.14  0.16 -0.14 -0.50  <orf19.3340
Ca21Chr1_0306034  0.30 -0.28  0.29 -0.03  0.91 -0.40 
Ca21Chr1_0306093  1.29 -0.13 -0.10  0.27  0.04  0.10 
Ca21Chr1_0306152 -0.52 -0.18  0.15  0.12 -0.28 -0.10 
Ca21Chr1_0306211    NA    NA    NA    NA    NA    NA
Ca21Chr1_0306270 -0.48 -0.23 -0.39  0.14  0.28  0.45 
Ca21Chr1_0306329 -0.10 -0.35 -0.48 -0.62 -0.19  0.02 
Ca21Chr1_0306388 -0.39 -0.43 -0.62 -0.32 -0.02  0.37 
Ca21Chr1_0306447 -0.31 -0.68 -0.33 -0.17 -0.48 -0.08 
Ca21Chr1_0306506  0.25 -0.54 -0.34 -0.12 -0.23  0.42 
Ca21Chr1_0306565  0.16 -0.07 -0.43 -0.15 -0.23  0.11 
Ca21Chr1_0306624  0.93 -0.10 -0.03 -0.06 -0.08 -0.08  |orf19.3341
Ca21Chr1_0306683  0.57 -0.19 -0.29  0.54 -0.66  0.72 
Ca21Chr1_0306742  0.30  0.20  0.18  0.04  0.24  0.02 
Ca21Chr1_0306801  0.81  0.18  0.33 -0.05  0.18  0.08 
Ca21Chr1_0306860  0.12  0.15  0.11 -0.14 -0.26 -0.17 
Ca21Chr1_0306919  0.34 -0.12  0.16  0.00 -0.00 -0.23 
Ca21Chr1_0306978  0.30  0.35  0.21  0.00 -0.44 -0.04 
Ca21Chr1_0307037  0.76  0.07  0.44  0.12  0.08 -0.31 
Ca21Chr1_0307096  0.90  0.58  0.60  0.14 -0.15  0.17 
Ca21Chr1_0307155  0.48 -0.19  0.13 -0.07  0.11 -0.03 
Ca21Chr1_0307214  0.46  0.34  0.19  0.09 -0.73 -0.22 
Ca21Chr1_0307273  0.34  0.08 -0.04  0.04 -0.25  0.06 
Ca21Chr1_0307332  0.35 -0.13 -0.10  0.21 -0.24  0.17 
Ca21Chr1_0307391  0.63  0.01  0.18 -0.25 -0.07  0.22 
Ca21Chr1_0307450  0.42 -0.03  0.19  0.18 -0.42 -0.03 
Ca21Chr1_0307509  0.33 -0.00 -0.13 -0.08 -0.03  0.41 
Ca21Chr1_0307568  0.64  0.04  0.34 -0.15 -0.07 -0.22 
Ca21Chr1_0307627  0.93  0.04  0.33 -0.07  0.31  0.27 
Ca21Chr1_0307686  0.57  0.15  0.17 -0.16  0.11  0.21 
Ca21Chr1_0307745  0.65  0.22  0.42  0.07  0.03  0.06 
Ca21Chr1_0307804  0.40  0.00  0.35  0.09  0.27 -0.06 
Ca21Chr1_0307863  0.19  0.02  0.41  0.35  0.16  0.11 
Ca21Chr1_0307922  0.59  0.04  0.34 -0.07  0.15  0.10 
Ca21Chr1_0307981  0.65 -0.09  0.28 -0.00  0.14  0.25 
Ca21Chr1_0308040  0.52 -0.15  0.03  0.26  0.05  0.29 
Ca21Chr1_0308099 -0.30 -0.20 -0.07  0.31  0.34  0.01 
Ca21Chr1_0308158  0.05  0.17 -0.14  0.17  0.18  0.15 
Ca21Chr1_0308217  1.15 -0.12  0.39 -0.12  0.07 -0.02 
Ca21Chr1_0308276  1.10  0.24 -0.30  0.18  0.10 -0.72 
Ca21Chr1_0308335  0.40  0.31  0.21  0.02 -0.35 -0.09 
Ca21Chr1_0308394  0.11  0.17  0.01 -0.05  0.24  0.30 
Ca21Chr1_0308453  0.44  0.15 -0.08  0.00  0.23 -0.13  <orf19.3341
Ca21Chr1_0308512  0.21 -0.03  0.14  0.16 -0.09 -0.51 
Ca21Chr1_0308571 -0.47 -0.34 -0.44 -0.09 -0.35  0.44 
Ca21Chr1_0308630 -0.23 -0.14 -0.40  0.06 -0.14  0.09 
Ca21Chr1_0308689 -0.04 -0.16 -0.23 -0.44 -0.30 -0.15 
Ca21Chr1_0308748 -0.24 -0.15 -0.10 -0.01 -0.25 -0.03 
Ca21Chr1_0308807 -0.10  0.15 -0.17  0.08 -0.45  0.06 
Ca21Chr1_0308866  0.04 -0.24 -0.21 -0.04 -0.24  0.02 
Ca21Chr1_0308925 -0.13 -0.21 -0.24  0.02 -0.56  0.08 
Ca21Chr1_0308984 -0.53 -0.74 -0.50 -0.40 -0.67 -0.06 
Ca21Chr1_0309043 -0.15 -0.48 -0.26  0.11  0.14 -0.21 
Ca21Chr1_0309102  0.00 -0.36 -0.14  0.27  0.16 -0.22  orf19.3342>
Ca21Chr1_0309161  0.22 -0.14 -0.74 -0.14  0.03 -0.46 
Ca21Chr1_0309220  0.09  0.07  0.51 -0.17 -0.20 -0.17 
Ca21Chr1_0309279  0.32  0.17  0.17  0.12 -0.15  0.00 
Ca21Chr1_0309338  0.08 -0.04 -0.04  0.80 -0.32  0.09 
Ca21Chr1_0309397 -0.18  0.20  0.13 -0.17 -0.30 -0.25 
Ca21Chr1_0309456 -0.00 -0.05  0.49  0.36  0.34  0.03 
Ca21Chr1_0309515  0.05  0.35  0.27  0.03 -0.12 -0.46 
Ca21Chr1_0309574  0.27  0.13  0.30 -0.05  0.07  0.01 
Ca21Chr1_0309633 -0.05 -0.14  0.30  0.33 -0.07  0.18 
Ca21Chr1_0309692  0.22 -0.01  0.12  0.29 -0.19 -0.01 
Ca21Chr1_0309751  0.29 -0.10  0.31 -0.15  0.09  0.04 
Ca21Chr1_0309810  0.21 -0.01  0.31  0.20  0.02 -0.26 
Ca21Chr1_0309869 -0.13  0.21  0.55  0.46  0.02 -0.31 
Ca21Chr1_0309928  0.01  0.21  0.32  0.31 -0.17 -0.39 
Ca21Chr1_0309987  0.34  0.42  0.18 -0.19 -0.07 -0.27 
Ca21Chr1_0310046  0.15  0.31  0.48  0.18  0.11 -0.12 
Ca21Chr1_0310105  0.17  0.45  0.16  0.02 -0.05 -0.01 
Ca21Chr1_0310164  0.45  0.10  0.28  0.07  0.14 -0.17 
Ca21Chr1_0310223 -0.07 -0.30 -0.44 -0.08  0.05  0.09 
Ca21Chr1_0310282  0.20 -0.26  0.27 -0.04  0.06 -0.01 
Ca21Chr1_0310341  0.01 -0.03  0.21  0.19 -0.12 -0.14 
Ca21Chr1_0310400  0.03  0.24 -0.04  0.12  0.27 -0.05 
Ca21Chr1_0310459  0.08 -0.07  0.12  0.16 -0.09 -0.08 
Ca21Chr1_0310518  0.10  0.06 -0.02  0.26 -0.20  0.09 
Ca21Chr1_0310577  0.36  0.11  0.17  0.11 -0.04  0.05 
Ca21Chr1_0310636  0.13  0.26  0.33  0.55 -0.01  0.14 
Ca21Chr1_0310695  0.37  0.23  0.21  0.28  0.04  0.03 
Ca21Chr1_0310754  0.13 -0.10  0.31  0.10 -0.34 -0.34 
Ca21Chr1_0310813 -0.03  0.34  0.21  0.05  0.22  0.26 
Ca21Chr1_0310872 -0.18  0.08 -0.25 -0.02 -0.06  0.03 
Ca21Chr1_0310931  0.05  0.27 -0.08 -0.02 -0.06  0.24  orf19.3342|
Ca21Chr1_0310990 -0.16  0.24 -0.43 -0.01 -0.52  0.11 
Ca21Chr1_0311049 -0.52  0.04 -1.01  0.08 -0.08 -0.13 
Ca21Chr1_0311108 -1.38 -0.54 -1.56  0.15  0.10 -0.14 
Ca21Chr1_0311167 -0.87 -0.69 -0.78  0.19  0.12 -0.12  orf19.3344>
Ca21Chr1_0311226 -1.09 -0.87 -1.33 -0.09 -0.08  0.13 
Ca21Chr1_0311285 -1.71 -1.39 -1.50 -0.38  0.27 -0.29 
Ca21Chr1_0311344 -1.63 -1.08 -1.15  0.11  0.30 -0.12 
Ca21Chr1_0311403 -1.46 -1.39 -1.60 -0.33 -0.12  0.04 
Ca21Chr1_0311462 -1.85 -1.05 -1.46  0.76  0.08 -0.06 
Ca21Chr1_0311521 -0.90 -0.74 -1.33 -0.11  0.17 -0.19 
Ca21Chr1_0311580 -0.47 -0.79 -0.92  0.15 -0.01 -0.16 
Ca21Chr1_0311639 -0.17 -0.86 -0.48  0.28 -0.04  0.06 
Ca21Chr1_0311698 -0.09 -0.23  0.22  0.24  0.05  0.08 
Ca21Chr1_0311757 -0.41 -0.11 -0.17  0.47  0.28  0.06 
Ca21Chr1_0311816  0.11  0.02  0.33  0.16  0.11  0.14 
Ca21Chr1_0311875  0.26  0.13  0.43  0.05 -0.14  0.39 
Ca21Chr1_0311934 -0.06  0.14  0.16 -0.11 -0.28 -0.12 
Ca21Chr1_0311993  0.17  0.14  0.26  0.10 -0.06  0.25 
Ca21Chr1_0312052 -0.02 -0.04  0.30  0.29  0.07  0.07 
Ca21Chr1_0312111  0.11  0.34  0.23  0.04  0.14 -0.15 
Ca21Chr1_0312170  0.17 -0.53  0.30  0.34 -0.18  0.15 
Ca21Chr1_0312229 -0.13 -0.21  0.05 -0.05 -0.02  0.13 
Ca21Chr1_0312288  0.21 -0.08 -0.06  0.10  0.17 -0.21 
Ca21Chr1_0312347  0.18 -0.25  0.34  0.05  0.03 -0.38 
Ca21Chr1_0312406 -0.10 -0.20 -0.04  0.47 -0.46  0.26 
Ca21Chr1_0312465 -0.23  0.07  0.21  0.24 -0.36 -0.23 
Ca21Chr1_0312524  0.25  0.05  0.19  0.28  0.17 -0.03 
Ca21Chr1_0312583 -0.10 -0.11  0.36 -0.50 -0.29 -0.16 
Ca21Chr1_0312642  0.13 -0.22  0.19  0.05 -0.32  0.40 
Ca21Chr1_0312701  0.06  0.40  0.59  0.15  0.03  0.06 
Ca21Chr1_0312760  0.67 -0.01 -0.04  0.38  0.33 -0.95  orf19.3344|
Ca21Chr1_0312819  0.22 -0.07 -0.24  0.17 -0.35 -0.62 
Ca21Chr1_0312878 -0.25  0.01 -0.08 -0.12 -0.13  0.33 
Ca21Chr1_0312937  0.09  0.33 -0.47  1.11 -0.18  0.27 
Ca21Chr1_0312996 -0.28  0.47 -0.65 -0.11  0.04  0.69 
Ca21Chr1_0313055 -0.15 -0.06  0.19  0.29  0.52  0.73 
Ca21Chr1_0313114  0.22 -0.29  0.15 -0.31  0.22 -0.08 
Ca21Chr1_0313173  0.00 -0.02  0.31  0.08 -0.19  0.08  orf19.3345>
Ca21Chr1_0313232  0.19 -0.26  0.18  0.12 -0.24  0.02 
Ca21Chr1_0313291  0.15  0.02  0.22  0.31 -0.17  0.03 
Ca21Chr1_0313350  0.03 -0.10  0.18 -0.27  0.27  0.16 
Ca21Chr1_0313409  0.27 -0.17  0.03  0.26 -0.03 -0.24 
Ca21Chr1_0313468 -0.10 -0.37 -0.17  0.01  0.23 -0.29 
Ca21Chr1_0313527 -0.25  0.65  0.11  0.11 -0.05 -0.05 
Ca21Chr1_0313586 -0.25 -0.13  0.05  0.04  0.07 -0.28 
Ca21Chr1_0313645 -0.12 -0.42  0.03 -0.02  0.70 -0.39 
Ca21Chr1_0313704  0.43 -0.08  0.27  0.20  0.22 -0.25 
Ca21Chr1_0313763  0.27 -0.18  0.24  0.11  0.24 -0.33 
Ca21Chr1_0313822  0.21  0.08  0.17  0.02  0.56  0.05 
Ca21Chr1_0313881  0.16  0.10  0.37  0.41 -0.74 -0.11 
Ca21Chr1_0313940  0.15  0.09  0.12  0.17  0.22  0.41 
Ca21Chr1_0313999 -0.16  0.12  0.12  0.24  0.03  0.17 
Ca21Chr1_0314058  0.13  0.25  0.04  0.17 -0.14 -0.08 
Ca21Chr1_0314117  0.30  0.03  0.18  0.17  0.30 -0.10 
Ca21Chr1_0314176 -0.13  0.25  0.17  0.28 -0.01 -0.05 
Ca21Chr1_0314235 -0.11 -0.16  0.06  0.38 -0.13 -0.67 
Ca21Chr1_0314294 -0.29 -0.16 -0.29 -0.15  0.31 -0.80 
Ca21Chr1_0314353 -0.29  0.45 -0.30  0.07  0.55 -0.60 
Ca21Chr1_0314412 -0.14 -0.29 -0.19 -0.14  0.42 -0.25 
Ca21Chr1_0314471 -0.07 -0.14 -0.25  0.20  0.35 -0.08 
Ca21Chr1_0314530 -0.12  0.54 -0.15  0.02  0.12 -0.17 
Ca21Chr1_0314589  0.33 -0.15  0.16  0.42  0.17 -0.42 
Ca21Chr1_0314648 -0.00 -0.03  0.40  0.35  0.27 -0.25 
Ca21Chr1_0314707  0.11  0.23  0.34  0.25 -0.13 -0.18 
Ca21Chr1_0314766  0.21  0.31  0.25  0.21  0.45 -0.07 
Ca21Chr1_0314825  0.01  0.17  0.22  0.20  0.30 -0.16 
Ca21Chr1_0314884  0.46  0.03  0.38 -0.46  0.23  0.11 
Ca21Chr1_0314943  0.53  0.07  0.20  0.04  0.27  0.12 
Ca21Chr1_0315002  0.02 -0.03  0.37  0.35  0.20 -0.16 
Ca21Chr1_0315061  0.20  0.38  0.47 -0.11  0.45  0.19 
Ca21Chr1_0315120  1.18  0.30  0.42 -0.06  0.13  0.10 
Ca21Chr1_0315179 -0.04  0.26  0.21  0.15  0.44  0.13 
Ca21Chr1_0315238  0.36  0.14  0.36  0.06  0.23  0.02 
Ca21Chr1_0315297 -0.43  0.49  0.57  0.04  0.06  0.18 
Ca21Chr1_0315356 -0.21 -0.34  0.31  0.02 -0.28  0.00 
Ca21Chr1_0315415  0.26  0.15  0.08 -0.15  0.28  0.28 
Ca21Chr1_0315474  0.11  0.12  0.31  0.21  0.20 -0.16 
Ca21Chr1_0315533 -0.38  0.56  0.69  0.51  0.09  0.40 
Ca21Chr1_0315592  0.55  0.22  0.69  0.40  0.41  0.33 
Ca21Chr1_0315651  0.79  0.33  0.65  0.28  0.55 -0.17 
Ca21Chr1_0315710  0.32 -0.06  0.48 -0.27  0.30 -0.74 
Ca21Chr1_0315769  0.21  0.09  0.24 -0.05  0.29 -0.32 
Ca21Chr1_0315828  0.18  0.00  0.44  0.27 -0.27 -0.80 
Ca21Chr1_0315887 -0.19  0.90  0.13 -0.25  0.38  0.45 
Ca21Chr1_0315946 -0.96 -0.26  0.25  0.47 -0.16  0.37 
Ca21Chr1_0316005  0.28 -0.11  0.42  0.14  0.09 -0.18 
Ca21Chr1_0316064  0.40  0.07  0.21 -0.05 -0.44 -0.43 
Ca21Chr1_0316123 -0.71 -0.58 -0.20  0.25  0.16 -0.52 
Ca21Chr1_0316182 -0.13 -0.44 -0.08  0.14 -0.13 -0.64 
Ca21Chr1_0316241 -0.37 -0.44 -0.19 -0.03 -0.18 -0.47 
Ca21Chr1_0316300 -0.30 -0.27  0.29  0.06  0.08 -0.07 
Ca21Chr1_0316359 -0.03 -0.22  0.22  0.12  0.46  0.03 
Ca21Chr1_0316418  0.09 -0.10  0.20  0.06 -0.12 -0.23 
Ca21Chr1_0316477 -0.11 -0.00  0.37  0.01  0.06 -0.18 
Ca21Chr1_0316536  0.29  0.22 -0.17  0.05  0.11 -0.08 
Ca21Chr1_0316595  0.19  0.07  0.61 -0.51 -0.05 -0.49 
Ca21Chr1_0316654 -0.22  0.31  0.32  0.30 -0.36 -0.28 
Ca21Chr1_0316713 -0.11  0.06  0.22 -0.17  0.27 -0.51 
Ca21Chr1_0316772  0.00  0.08  0.31  0.04  0.31 -0.19 
Ca21Chr1_0316831  0.31 -0.07  0.13  0.12 -0.03 -0.32 
Ca21Chr1_0316890  0.04  0.29  0.31 -0.08 -0.01 -0.27 
Ca21Chr1_0316949 -0.01 -0.13  0.18  0.35  0.12  0.04 
Ca21Chr1_0317008 -0.02 -0.14  0.52  0.03  0.09  0.13 
Ca21Chr1_0317067 -0.40  0.06  0.29 -0.04  0.20 -0.11 
Ca21Chr1_0317126 -0.18  0.12  0.03  0.07  0.38 -0.46 
Ca21Chr1_0317185 -0.35  0.02  0.22 -0.07  0.09 -0.64 
Ca21Chr1_0317244  0.65  0.11  0.31  0.24 -0.01 -0.36 
Ca21Chr1_0317303  0.07  0.22  0.26  0.03  0.33 -0.22 
Ca21Chr1_0317362 -0.09  0.10  0.38  0.25  0.31 -0.14 
Ca21Chr1_0317421  0.37  0.08 -0.02  0.41 -0.73  0.00 
Ca21Chr1_0317480  0.38  0.26  0.40  0.25  0.08  0.13 
Ca21Chr1_0317539  0.21  0.16  0.24  0.25  0.01  0.18 
Ca21Chr1_0317598 -0.19  0.20  0.13  0.00 -0.04  0.26 
Ca21Chr1_0317657  0.14  0.32 -0.10  0.46 -0.52 -0.04 
Ca21Chr1_0317716  0.20  0.40  0.08  0.18  0.26 -0.09 
Ca21Chr1_0317775 -0.30  0.02  0.41  0.99  0.05 -0.00  orf19.3345|
Ca21Chr1_0317834 -0.25  0.05  0.12 -0.19 -0.20 -0.85 
Ca21Chr1_0317893  0.09 -0.06 -0.02 -0.81 -0.75  0.05 
Ca21Chr1_0317952  0.26  0.23  0.12 -0.29  0.23  0.30  |orf19.3347
Ca21Chr1_0318011  0.65  0.42 -0.06 -0.10  0.23  0.10 
Ca21Chr1_0318070  0.27  0.38  0.12  0.07  0.20  0.32 
Ca21Chr1_0318129 -0.22  0.26  0.33  0.01 -0.11 -0.02 
Ca21Chr1_0318188  0.28 -0.00  0.42  0.07  0.27  0.14 
Ca21Chr1_0318247  0.12  0.27  0.45  0.28  0.29 -0.01 
Ca21Chr1_0318306  0.14  0.06  0.15 -0.07  0.09  0.07 
Ca21Chr1_0318365  0.21  0.11  0.27  0.10 -0.44 -0.05 
Ca21Chr1_0318424  0.09  0.31  0.43  0.29  0.48  0.17 
Ca21Chr1_0318483  0.87 -0.19  0.01  0.07  0.18  0.44  <orf19.3347
Ca21Chr1_0318542  0.72 -0.15  0.04  0.22  0.10 -0.13 
Ca21Chr1_0318601  0.07  0.05 -0.34  0.10  0.20 -0.39 
Ca21Chr1_0318660  0.19  0.27  0.07  0.33  0.13 -0.06 
Ca21Chr1_0318719 -0.32  0.06 -0.18  0.09 -0.21 -0.15 
Ca21Chr1_0318778 -0.36 -0.21 -0.52 -0.08 -0.29  0.28 
Ca21Chr1_0318837 -0.42 -0.12  0.19  0.13 -0.24 -0.01 
Ca21Chr1_0318896  0.15  0.02  0.23 -0.23  0.05 -0.25  orf19.3348>
Ca21Chr1_0318955  0.13  0.29  0.44  0.20 -0.12 -0.16 
Ca21Chr1_0319014 -0.20  0.12  0.49  0.09 -0.46 -0.15 
Ca21Chr1_0319073 -0.07  0.02  0.48  0.05 -0.21 -0.41 
Ca21Chr1_0319132 -0.03  0.02  0.23  0.31 -0.18 -0.21 
Ca21Chr1_0319191  0.02 -0.23  0.16  0.10 -0.38 -0.05 
Ca21Chr1_0319250 -0.19  0.03  0.27  0.30 -0.21  0.10 
Ca21Chr1_0319309  0.04  0.18  0.14  0.05  0.26  0.26 
Ca21Chr1_0319368 -0.49  0.07  0.06  0.39 -0.06  0.25 
Ca21Chr1_0319427 -0.25 -0.15  0.08  0.46  0.55  0.13  orf19.3348|
Ca21Chr1_0319486  0.07 -0.00 -0.40 -0.35 -0.15 -0.30 
Ca21Chr1_0319545  0.37  0.14 -0.20 -0.10  0.33 -0.97 
Ca21Chr1_0319604  0.55 -0.28  0.29  0.16 -0.02 -0.36 
Ca21Chr1_0319663  0.34 -0.06 -0.20  0.19 -0.10  0.06  |orf19.3349
Ca21Chr1_0319722  0.59 -0.03 -0.51 -0.48 -0.22  0.11 
Ca21Chr1_0319781  0.34 -0.01  0.06 -0.08 -0.11 -0.11 
Ca21Chr1_0319840  1.26  0.07  0.25 -0.10  0.07  0.06 
Ca21Chr1_0319899  0.12  0.34  0.32 -0.06 -0.07  0.11 
Ca21Chr1_0319958  0.41  0.43  0.22  0.14  1.14  0.06 
Ca21Chr1_0320017  0.31 -0.15  0.36 -0.18  0.17  0.01 
Ca21Chr1_0320076  0.64  0.03  0.06  0.07 -0.44 -0.00 
Ca21Chr1_0320135  0.06  0.07  0.34  0.10  0.01  0.15 
Ca21Chr1_0320194  0.51  0.04  0.46 -0.28  0.10  0.04 
Ca21Chr1_0320253  0.01 -0.63  0.50 -0.09 -0.21 -0.14 
Ca21Chr1_0320312  0.41 -0.01  0.33 -0.13  0.14 -0.10 
Ca21Chr1_0320371  0.15  0.24  0.22  0.05 -0.11 -0.22 
Ca21Chr1_0320430  0.57  0.35 -0.06 -0.32 -0.07  0.16 
Ca21Chr1_0320489  0.40  0.48  0.28 -0.62 -0.10 -0.05 
Ca21Chr1_0320548  0.76  0.22  0.33  0.12 -0.45  0.72 
Ca21Chr1_0320607  0.45  0.17  0.12  0.36 -0.11  0.05 
Ca21Chr1_0320666  0.03  0.20  0.44  0.12  0.13  0.12 
Ca21Chr1_0320725  0.32  0.03  0.07 -0.34  0.10  0.01 
Ca21Chr1_0320784  0.48 -0.03  0.19  0.47  0.29 -0.14 
Ca21Chr1_0320843  0.14 -0.08  0.14  0.13  0.04 -0.27 
Ca21Chr1_0320902  0.40 -0.13  0.13 -0.03  0.56 -0.11 
Ca21Chr1_0320961  0.31 -0.22  0.19 -0.32  0.41  0.06 
Ca21Chr1_0321020  0.45  0.50  0.48 -0.08  0.11 -0.01 
Ca21Chr1_0321079  0.63  0.88  0.38  0.20  0.45 -0.33 
Ca21Chr1_0321138  0.60  0.75  0.60 -0.05  0.69 -0.30 
Ca21Chr1_0321197  0.28 -0.05  0.60 -0.04  0.17 -0.17 
Ca21Chr1_0321256  0.99 -0.17  0.49  0.12  0.20 -0.29 
Ca21Chr1_0321315  0.69 -0.04  0.57  0.20  0.30 -0.28 
Ca21Chr1_0321374 -0.06  0.35  0.56 -0.15  0.50 -0.59 
Ca21Chr1_0321433  1.18  0.12  0.51 -0.14  0.21 -0.66 
Ca21Chr1_0321492  0.36  0.32  0.68  0.45  1.25 -0.10 
Ca21Chr1_0321551  0.65  0.14  0.63 -0.02  0.37  0.19 
Ca21Chr1_0321610  0.27  0.31  0.29  0.13 -0.11  0.03 
Ca21Chr1_0321669  0.10 -0.11  0.41 -0.11 -0.02  0.06 
Ca21Chr1_0321728  0.51  0.09  0.64 -0.22 -0.12 -0.54 
Ca21Chr1_0321787  0.14  0.49  0.50 -0.71  0.04  0.10 
Ca21Chr1_0321846  0.39  0.05  0.44 -0.53  0.20 -0.31 
Ca21Chr1_0321905  0.28  0.14  0.73 -0.30  0.32 -0.48 
Ca21Chr1_0321964  0.28  0.17  0.40 -0.25  0.22 -0.13 
Ca21Chr1_0322023  0.41  0.47  0.50 -0.02  0.39 -0.10 
Ca21Chr1_0322082 -0.00  0.30  0.23 -0.11 -0.04  0.60 
Ca21Chr1_0322141 -0.06  0.41  0.37  0.06  0.32  0.52 
Ca21Chr1_0322200  0.82  0.01  0.47 -0.10  0.29  0.45 
Ca21Chr1_0322259  0.29  0.04  0.22 -0.14  0.04  0.39 
Ca21Chr1_0322318  0.06  0.28  0.07 -0.17  0.05  0.14 
Ca21Chr1_0322377  0.41 -0.12  0.68 -0.44 -0.68  0.21 
Ca21Chr1_0322436  0.51  0.44  0.80  0.12  0.16  0.31 
Ca21Chr1_0322495  0.30  0.36  0.50  0.03  0.02  0.18 
Ca21Chr1_0322554  0.37  0.25  0.37  0.00  0.04  0.26 
Ca21Chr1_0322613  0.44 -0.01  0.57  0.15 -0.01  0.44 
Ca21Chr1_0322672  0.65  0.09  0.44  0.37 -0.11  0.27 
Ca21Chr1_0322731  0.50  0.08  0.13  0.14  0.07  0.17 
Ca21Chr1_0322790  0.40  0.09  0.55  0.16  0.06  0.20 
Ca21Chr1_0322849  0.12  0.41  0.18  0.16  0.14 -0.09 
Ca21Chr1_0322908  0.28  0.30  0.52 -0.10  0.29  0.28 
Ca21Chr1_0322967  0.58 -0.16 -0.01 -0.22 -0.48  0.14 
Ca21Chr1_0323026  0.34  0.13  0.27  0.15  0.13  0.04 
Ca21Chr1_0323085  0.14 -0.17  0.37  0.24  0.45 -0.04 
Ca21Chr1_0323144  0.35 -0.15  0.03  0.36 -0.07  0.02 
Ca21Chr1_0323203  0.42  0.01  0.27  0.22 -0.22  0.20 
Ca21Chr1_0323262  0.13  0.04  0.02  0.02  0.02  0.36 
Ca21Chr1_0323321  0.08  0.33  0.20 -0.00 -0.07  0.29 
Ca21Chr1_0323380 -0.14  0.06  0.12  0.07 -0.10  0.72  <orf19.3349
Ca21Chr1_0323439  0.11  0.08  0.34  0.12 -0.15  0.89 
Ca21Chr1_0323498 -0.17  0.14  0.10 -0.06 -0.47  0.73 
Ca21Chr1_0323557  0.21  0.09  0.06  0.30 -0.04  0.26 
Ca21Chr1_0323616  0.91  0.25  0.05 -0.01 -0.57  0.36  orf19.3350>
Ca21Chr1_0323675 -0.35  0.36  0.18 -0.12 -0.02 -0.25 
Ca21Chr1_0323734  0.70  0.13  0.15 -0.01 -0.03  0.06 
Ca21Chr1_0323793  0.13  0.92  0.32 -0.19 -0.32  0.17 
Ca21Chr1_0323852 -0.49  0.11  0.12 -0.22 -0.11  0.40 
Ca21Chr1_0323911 -0.22  0.47  0.37 -0.09 -0.25  0.14 
Ca21Chr1_0323970  0.01  0.32  0.66  0.34 -0.10 -0.20 
Ca21Chr1_0324029 -0.07 -0.22 -0.41  0.43  0.01  0.26 
Ca21Chr1_0324088  0.17 -0.70  0.70  0.20  0.15 -0.07 
Ca21Chr1_0324147  0.08  0.08  0.21 -0.12  0.09 -0.04 
Ca21Chr1_0324206  0.09  0.05  0.23 -0.08  0.46 -0.09 
Ca21Chr1_0324265 -0.12 -0.01  0.48  0.38 -0.07 -0.22 
Ca21Chr1_0324324 -0.01 -0.06  0.20  0.11  0.32  0.25 
Ca21Chr1_0324383  0.10 -0.06  0.21  0.04 -0.05 -0.25 
Ca21Chr1_0324442 -0.06 -0.20  0.25  0.27 -0.35 -0.71 
Ca21Chr1_0324501  0.21  0.12  0.72  0.34 -0.18 -0.08  orf19.3350|
Ca21Chr1_0324560  2.23  0.07 -0.90  0.14  1.23 -1.41 
Ca21Chr1_0324619  0.02 -0.08 -0.02  0.27  1.66 -0.46 
Ca21Chr1_0324678  0.25  0.17  0.00 -0.01  0.08 -0.13 
Ca21Chr1_0324737  1.64 -0.22  0.20 -0.17  0.89 -0.58 
Ca21Chr1_0324796  0.36  0.08 -0.51 -0.07  0.80 -0.21 
Ca21Chr1_0324855 -0.38  0.65  0.10  0.14  0.32  0.12  |orf19.3351
Ca21Chr1_0324914  0.08  0.24  0.42 -0.21 -0.09  2.78 
Ca21Chr1_0324973 -0.24  0.15 -0.09 -0.52 -0.14  0.44 
Ca21Chr1_0325032  0.37 -0.17 -0.02 -0.22 -0.08 -0.02 
Ca21Chr1_0325091 -0.75 -0.05 -0.15 -0.28 -0.19  0.30 
Ca21Chr1_0325150  0.06 -0.23 -0.32  0.10 -0.65  1.04 
Ca21Chr1_0325209 -0.66  0.07  0.30 -0.31  0.26  0.30 
Ca21Chr1_0325268  1.00  0.04 -0.13  0.05 -0.24  0.04 
Ca21Chr1_0325327  1.38  0.29  0.70  0.19  2.36 -0.09 
Ca21Chr1_0325386 -0.31  0.47  0.22 -0.17  0.28  0.00 
Ca21Chr1_0325445  0.05 -0.20 -1.57  0.56  0.17 -0.18 
Ca21Chr1_0325504  0.72 -0.32  0.31 -0.18  0.03  0.31 
Ca21Chr1_0325563  0.25  0.20  0.22 -0.09  0.02  0.46 
Ca21Chr1_0325622  0.10  0.24  0.63  0.23  0.04  0.21 
Ca21Chr1_0325681  0.11  0.35  0.29 -0.11 -0.05 -0.02 
Ca21Chr1_0325740  0.21  0.33  0.36  0.24 -0.26  0.02 
Ca21Chr1_0325799 -0.06 -0.25  0.43  0.35 -0.32 -0.53 
Ca21Chr1_0325858 -0.43  0.06  0.09 -0.43  0.36  0.05 
Ca21Chr1_0325917 -0.36  0.42  0.54 -0.13 -0.26 -0.43  <orf19.3351
Ca21Chr1_0325976 -0.66 -0.08 -0.06 -0.10 -0.23  0.37 
Ca21Chr1_0326035  0.31  0.27  0.26  0.23 -0.28 -0.25 
Ca21Chr1_0326094  0.29 -0.34  0.18  0.20 -0.08  0.68 
Ca21Chr1_0326153  0.45 -0.25 -0.65  0.03 -0.28 -0.11 
Ca21Chr1_0326212  0.69  0.31 -0.64  0.25 -0.30 -0.41 
Ca21Chr1_0326271  0.20  0.03  0.73 -0.11  0.55 -1.24 
Ca21Chr1_0326330  0.05  0.20 -0.15  0.20  0.31 -1.02 
Ca21Chr1_0326389 -0.14 -0.17 -0.57  0.24 -0.22 -0.08  |orf19.3352
Ca21Chr1_0326448  0.30  0.49  0.31 -0.31 -0.03 -0.19 
Ca21Chr1_0326507  0.08  0.11  0.39  0.34 -0.11 -0.01 
Ca21Chr1_0326566  0.48 -0.00  0.55 -0.13 -0.32  0.27 
Ca21Chr1_0326625 -0.05  0.25  0.25 -0.26  0.02  0.11 
Ca21Chr1_0326684 -0.06  0.45  0.22  0.07 -0.12  0.85 
Ca21Chr1_0326743 -0.53  0.29  0.03  0.44 -0.07  1.18 
Ca21Chr1_0326802 -0.08  0.07 -0.02  0.18 -0.46 -0.34 
Ca21Chr1_0326861  0.01 -0.01 -0.20  0.27  0.58 -0.56 
Ca21Chr1_0326920 -0.10  0.06  0.01  0.12 -0.21  0.33 
Ca21Chr1_0326979 -0.59 -0.23 -0.06  0.35 -0.29 -0.14 
Ca21Chr1_0327038  0.09 -0.08  0.11  0.13  0.53  0.43 
Ca21Chr1_0327097 -0.39  0.10  0.15  0.04  0.14  0.10 
Ca21Chr1_0327156  0.46  0.39  0.26  0.08 -0.06  0.74 
Ca21Chr1_0327215  0.52  0.35  5.95 -0.51  0.02  0.33 
Ca21Chr1_0327274  0.33  0.25  0.12  0.21  0.06 -0.23 
Ca21Chr1_0327333 -0.02 -0.43  0.32  0.29 -0.06  0.14 
Ca21Chr1_0327392 -0.01  0.30 -0.20 -0.00  0.07  0.54  <orf19.3352
Ca21Chr1_0327451  1.18 -0.21 -0.06  0.08 -0.20  0.71 
Ca21Chr1_0327510  0.32 -0.15 -0.43  0.36  1.58 -0.03 
Ca21Chr1_0327569  0.16  0.04  1.41 -0.12 -0.04  0.42 
Ca21Chr1_0327628 -0.04 -0.31 -0.35 -0.18  0.18 -0.05 
Ca21Chr1_0327687  0.79  0.14  0.32  0.08 -0.11  0.27 
Ca21Chr1_0327746  0.72  0.45 -0.23  0.26  0.12 -0.11 
Ca21Chr1_0327805 -0.09  0.14  0.06  0.51 -0.01  0.17 
Ca21Chr1_0327864  0.01  0.34 -0.27  0.34  0.10 -0.03 
Ca21Chr1_0327923  0.20 -0.06 -0.19  0.19  0.02  0.39 
Ca21Chr1_0327982 -0.47 -0.38 -0.27  0.16  0.22 -0.24 
Ca21Chr1_0328041  0.09 -0.23 -0.30 -0.38  0.13  0.34 
Ca21Chr1_0328100 -0.59 -0.30 -0.46  0.08  0.21 -0.01 
Ca21Chr1_0328159 -0.06 -0.70 -0.95 -0.11  0.32 -0.41 
Ca21Chr1_0328218 -0.24  0.06 -0.46 -0.08 -0.05 -0.27 
Ca21Chr1_0328277 -0.09  0.02 -0.39  0.40  0.25  0.50 
Ca21Chr1_0328336 -0.75  0.37 -0.77  0.23 -0.17  0.82 
Ca21Chr1_0328395 -0.06 -0.41 -0.48  0.01  0.10  0.46 
Ca21Chr1_0328454 -0.51 -0.38 -0.61  0.22 -0.14  0.18 
Ca21Chr1_0328513  0.52 -0.38  0.50 -0.11 -0.03  0.69 
Ca21Chr1_0328572  0.19  0.01 -0.59 -0.05 -0.28  0.29 
Ca21Chr1_0328631  0.35  0.61 -0.13 -0.06  0.08  0.34 
Ca21Chr1_0328690 -0.03 -0.17 -0.19  0.13 -0.13 -0.05 
Ca21Chr1_0328749 -0.41 -0.37 -0.37  0.39  0.05 -0.27 
Ca21Chr1_0328808 -0.16 -0.20 -0.62  0.09  0.05 -0.27 
Ca21Chr1_0328867 -0.29 -0.33 -0.79  0.32 -0.26 -0.30 
Ca21Chr1_0328926  0.10 -0.05 -0.59 -0.11 -0.14 -0.08 
Ca21Chr1_0328985  0.07 -0.04 -0.57  0.00  0.43  0.03 
Ca21Chr1_0329044 -1.38 -0.15 -1.45  0.54  0.06 -0.12 
Ca21Chr1_0329103  0.39 -0.66 -0.29  0.06  0.24  0.19 
Ca21Chr1_0329162  0.04  0.00 -0.21 -0.10  0.08  0.66 
Ca21Chr1_0329221  0.53  0.03 -0.36  0.30  0.75  0.08 
Ca21Chr1_0329280 -0.03  0.29 -0.40  0.19  0.21 -0.27 
Ca21Chr1_0329339  0.06 -0.24 -0.53  0.82  0.17  0.29 
Ca21Chr1_0329398 -0.52 -0.23 -0.34  0.63  0.28  0.62 
Ca21Chr1_0329457  0.11 -0.08  0.07  0.11  0.07  0.54 
Ca21Chr1_0329516 -0.47 -0.09 -0.13  0.43 -0.22  0.30 
Ca21Chr1_0329575  0.03 -0.23  0.02  0.02  0.41  0.69 
Ca21Chr1_0329634 -0.24 -0.04 -0.11  0.49  0.44  0.56 
Ca21Chr1_0329693  0.04 -0.04 -0.26  0.18  0.16  0.48 
Ca21Chr1_0329752 -0.15 -0.21 -0.48 -0.16  0.18  0.59 
Ca21Chr1_0329811 -0.60 -0.41 -0.91  0.15 -0.18  0.20 
Ca21Chr1_0329870 -0.99 -0.48 -1.26 -0.03 -0.19  0.38 
Ca21Chr1_0329929 -0.44 -0.75 -1.52 -0.23 -0.01  0.04 
Ca21Chr1_0329988 -1.06 -0.74 -1.46  0.32 -0.03  0.25 
Ca21Chr1_0330047 -1.13 -1.12 -1.69 -0.11  0.08  0.33 
Ca21Chr1_0330106  0.43 -1.25 -1.13 -0.14  0.07  0.54 
Ca21Chr1_0330165 -0.00 -0.41 -1.12 -0.16  0.01 -0.37 
Ca21Chr1_0330224 -0.05 -0.31  0.11 -0.10  0.25 -0.00 
Ca21Chr1_0330283  0.21 -0.02 -0.22 -0.04 -0.39  0.50 
Ca21Chr1_0330342 -0.04  0.04 -0.03  0.05  0.10  0.17 
Ca21Chr1_0330401  0.35  0.16  0.26  0.02 -0.05  0.49 
Ca21Chr1_0330460    NA    NA    NA    NA    NA    NA
Ca21Chr1_0330519 -0.15  0.17 -0.08 -0.01  0.13  0.50 
Ca21Chr1_0330578  0.20 -0.05 -0.11 -0.29  0.40  0.00 
Ca21Chr1_0330637 -0.26  0.16 -0.23  0.39  0.19  0.18 
Ca21Chr1_0330696 -0.06  0.29  0.28  0.47  0.46  0.60 
Ca21Chr1_0330755 -0.14  0.12  0.21 -0.04 -0.14 -0.05 
Ca21Chr1_0330814 -0.24 -0.00 -0.10  0.06  0.04  0.41 
Ca21Chr1_0330873 -0.18  0.52 -0.01 -0.09 -0.13  0.89 
Ca21Chr1_0330932 -0.18  0.05 -0.36  0.14  0.02  0.08 
Ca21Chr1_0330991  0.39 -0.02 -0.27 -0.17 -0.14  0.29 
Ca21Chr1_0331050  0.16 -0.06 -0.29 -0.13 -0.14  0.46 
Ca21Chr1_0331109  0.01 -0.26 -0.49 -0.10 -0.06  0.22 
Ca21Chr1_0331168  0.11 -0.13  0.31  0.43  0.11  0.35 
Ca21Chr1_0331227  0.16  0.00  0.22 -0.18  0.27 -0.57 
Ca21Chr1_0331286  0.18  0.03  0.58  0.27  0.34  0.00 
Ca21Chr1_0331345  0.03 -0.16  0.11  0.11 -0.52  0.02  orf19.3353>
Ca21Chr1_0331404  0.30  0.13  0.29 -0.26 -0.07  0.60 
Ca21Chr1_0331463  0.94  0.33  0.54  0.03 -0.04 -0.06 
Ca21Chr1_0331522  0.29 -0.01  0.16 -0.09 -0.41 -0.07 
Ca21Chr1_0331581  0.19  0.10  0.50 -0.10 -0.04  0.48 
Ca21Chr1_0331640  0.57  0.17  0.31 -0.15  0.02  0.09 
Ca21Chr1_0331699  0.07  0.13  0.38  0.44  0.28 -0.41 
Ca21Chr1_0331758 -0.05  0.43  0.26  0.13 -0.14  0.27 
Ca21Chr1_0331817  0.17  0.34  0.25  0.11  0.02  0.08 
Ca21Chr1_0331876  0.35  0.25  0.25 -0.02 -0.34  0.18 
Ca21Chr1_0331935 -0.14  0.10 -0.11 -0.09 -0.01 -0.12 
Ca21Chr1_0331994 -0.08  0.12  0.12 -0.18 -0.00  0.01 
Ca21Chr1_0332053 -0.21  0.36  0.41  0.16 -0.26 -0.04  orf19.3353|
Ca21Chr1_0332112  0.42  0.22  0.33 -0.24  0.03 -0.53 
Ca21Chr1_0332171 -0.18  0.26  0.38 -0.52 -0.16  0.27 
Ca21Chr1_0332230  0.03  0.47  0.20  0.19 -0.01  0.70 
Ca21Chr1_0332289  0.77 -0.06 -0.17 -0.53  0.12  0.08 
Ca21Chr1_0332348 -0.35 -0.06  0.10 -0.24 -0.23  0.11 
Ca21Chr1_0332407  0.75  0.34 -0.22 -0.06 -0.23 -0.68 
Ca21Chr1_0332466 -0.14  0.28  0.16  0.04 -0.06  0.33 
Ca21Chr1_0332525 -0.80  0.09  0.24  0.28  0.03  0.43 
Ca21Chr1_0332584 -0.15  0.01  0.08 -0.32  0.62  0.18 
Ca21Chr1_0332643 -0.24  0.22 -0.04 -0.04 -0.08 -0.29 
Ca21Chr1_0332702 -0.13 -0.15 -0.22 -0.35 -0.25  0.21  orf19.3354>
Ca21Chr1_0332761 -0.72 -0.05  0.16 -0.12  0.05 -0.05 
Ca21Chr1_0332820 -0.49 -0.18 -0.03  0.23 -0.31  0.50 
Ca21Chr1_0332879  0.20  0.00 -0.20 -0.23 -0.01 -0.05 
Ca21Chr1_0332938 -0.34  0.27 -0.16 -0.37 -0.69  0.04 
Ca21Chr1_0332997 -0.62  0.06 -0.39 -0.26  0.19  0.25 
Ca21Chr1_0333056 -0.87  0.25 -0.44  0.08  0.37  0.51 
Ca21Chr1_0333115 -1.27 -0.07 -0.38 -0.38  0.24  0.41 
Ca21Chr1_0333174 -1.10  0.19 -0.29 -0.45  0.18  0.26 
Ca21Chr1_0333233 -0.14 -0.09 -0.16 -0.30  0.18  0.35 
Ca21Chr1_0333292 -0.43 -0.17 -0.32 -0.15  0.11 -0.44 
Ca21Chr1_0333351 -0.13  0.28  0.28 -0.46  0.21 -0.72 
Ca21Chr1_0333410  0.33  0.33  0.10 -0.38  0.32 -0.23 
Ca21Chr1_0333469  0.23  0.56  0.69  0.02  1.06  0.49 
Ca21Chr1_0333528  0.21  0.52  1.41  0.12  0.63 -0.27 
Ca21Chr1_0333587  0.28  0.46  0.90  0.13  0.12 -0.39 
Ca21Chr1_0333646  0.24  0.40  0.87 -0.14 -0.08  0.02 
Ca21Chr1_0333705  0.56  0.69  1.23  0.14  0.24 -0.39 
Ca21Chr1_0333764  0.18  0.89  0.80  0.01  0.13 -0.07 
Ca21Chr1_0333823  0.46  0.48  0.99  0.04 -0.04 -0.04 
Ca21Chr1_0333882 -0.05  0.55  0.67  0.08  0.07 -0.37 
Ca21Chr1_0333941  0.03  0.27  0.42  0.22  0.15 -0.10 
Ca21Chr1_0334000 -0.16  0.31  0.30  0.06 -0.04 -0.23 
Ca21Chr1_0334059  0.02  0.21  0.40  0.13  0.14 -0.44  orf19.3354|
Ca21Chr1_0334118  0.18  0.24 -0.28 -0.13 -0.44 -0.18 
Ca21Chr1_0334177  0.86 -0.30 -0.48 -0.01  0.49 -0.80 
Ca21Chr1_0334236 -0.58 -0.50 -0.26 -0.99  0.40 -0.53 
Ca21Chr1_0334295 -0.06 -0.21 -0.98 -1.43 -0.51 -0.35 
Ca21Chr1_0334354 -0.67 -0.73 -0.90 -1.13 -0.56  0.05 
Ca21Chr1_0334413 -0.29 -0.47 -0.38 -0.05 -0.04  0.12 
Ca21Chr1_0334472 -0.26 -0.50 -0.85 -0.17 -0.30 -0.19 
Ca21Chr1_0334531 -0.30 -0.22 -0.43  0.20  0.05 -0.36 
Ca21Chr1_0334590 -0.07  0.20 -0.27 -0.06  0.21 -0.34 
Ca21Chr1_0334649  0.30  0.53 -0.02 -0.05  0.22  0.03  orf19.3355>
Ca21Chr1_0334708  0.14  0.39 -0.03 -0.15 -0.07 -0.05 
Ca21Chr1_0334767 -0.13  0.15  0.01  0.17 -0.13  0.22 
Ca21Chr1_0334826  0.12  0.23 -0.08 -0.25  0.08  0.23 
Ca21Chr1_0334885 -0.06  0.32  0.24  0.58 -0.21  0.48 
Ca21Chr1_0334944  0.01  0.06  0.11 -0.00  0.28  0.01 
Ca21Chr1_0335003  0.08  0.01  0.04  0.25  0.01 -0.21 
Ca21Chr1_0335062  0.24  0.10 -0.12 -0.44 -0.21 -0.21 
Ca21Chr1_0335121 -1.09  0.30  0.14  0.99 -0.88  0.58 
Ca21Chr1_0335180 -0.08  0.11  0.10 -0.07 -0.02 -0.24 
Ca21Chr1_0335239  0.07  0.18  0.29 -0.25 -0.30 -0.25 
Ca21Chr1_0335298  0.20 -0.06  0.49 -0.40 -0.37 -0.22 
Ca21Chr1_0335357 -0.18  0.24  0.48 -0.25 -0.29 -0.06 
Ca21Chr1_0335416  0.84 -0.06  0.17 -0.38 -0.15 -0.67 
Ca21Chr1_0335475  1.20  0.29  0.07  0.13 -0.06  0.12 
Ca21Chr1_0335534  0.50  0.21  0.23  0.45 -0.35  0.09 
Ca21Chr1_0335593  0.09  0.08  0.37  0.28  0.36  0.17 
Ca21Chr1_0335652  1.15  0.25  0.62  0.21  0.06 -0.36 
Ca21Chr1_0335711  0.16  0.35  0.41  0.03  0.30  0.52 
Ca21Chr1_0335770  0.12  0.17 -0.21  0.03  0.34 -0.15 
Ca21Chr1_0335829 -0.00  0.30  0.26 -0.13  0.13  0.15 
Ca21Chr1_0335888  0.02  0.31  0.35 -0.14 -0.00  0.04 
Ca21Chr1_0335947  0.22  0.16  0.36 -0.36 -0.27  0.46 
Ca21Chr1_0336006 -0.22  0.03  0.19  0.37  0.17 -0.16  orf19.3355|
Ca21Chr1_0336065 -0.28 -0.37 -0.14 -0.64 -0.24  0.37 
Ca21Chr1_0336124  0.06  0.75 -0.76  0.04  0.81 -0.36  |tS(UGA)1
Ca21Chr1_0336183  0.03  0.30  0.41 -0.64 -0.17 -0.40  <tS(UGA)1
Ca21Chr1_0336242 -0.30  0.39 -0.18 -0.72 -0.10 -0.16 
Ca21Chr1_0336301 -0.61  0.03 -0.34 -0.07  0.47  0.26 
Ca21Chr1_0336360 -0.20  0.05 -0.17 -0.25 -0.11  0.37 
Ca21Chr1_0336419 -0.35 -0.53 -0.18 -0.09 -0.02  0.64 
Ca21Chr1_0336478 -0.29 -0.08 -0.14  0.01  0.13  0.80 
Ca21Chr1_0336537 -0.32  0.39  0.01 -0.02  0.26  0.04 
Ca21Chr1_0336596  0.11  0.50 -0.10  0.23 -0.05  1.70 
Ca21Chr1_0336655  0.98  0.05  1.18 -0.03 -0.46 -0.43 
Ca21Chr1_0336714 -0.08  0.64  0.59  0.10  0.07 -0.44  |orf19.3356
Ca21Chr1_0336773  0.25  0.42  0.37  0.29 -0.62 -0.26 
Ca21Chr1_0336832  0.13  0.11  0.27 -0.04 -0.08  0.03 
Ca21Chr1_0336891  0.05  0.30  0.53  0.18 -0.22  0.11 
Ca21Chr1_0336950  0.36  0.18  0.31 -0.32 -0.19  0.25 
Ca21Chr1_0337009  0.56  0.28  0.21  0.14 -0.36 -0.35 
Ca21Chr1_0337068  0.34  0.11 -0.05 -0.05 -0.10  0.06 
Ca21Chr1_0337127 -0.01  0.04 -0.03  0.10 -0.05 -0.04 
Ca21Chr1_0337186  0.10  0.27  0.32  0.18 -0.24  0.29 
Ca21Chr1_0337245 -0.10  0.25  0.76 -0.31 -0.35  1.62 
Ca21Chr1_0337304  0.19  0.02 -0.03 -0.17  0.16 -0.05 
Ca21Chr1_0337363 -0.54  0.06  0.83 -0.07  0.41 -0.38 
Ca21Chr1_0337422  0.13  0.21  0.23 -0.06 -0.11 -0.52 
Ca21Chr1_0337481  0.13  0.55  0.06  0.41 -0.11 -0.17 
Ca21Chr1_0337540 -0.92 -0.02 -0.34 -0.24  0.13 -0.70 
Ca21Chr1_0337599  0.01  0.26  0.99 -0.03 -0.07  0.03 
Ca21Chr1_0337658  0.34  0.13  0.25  0.03  0.21  0.35 
Ca21Chr1_0337717 -0.48  0.20  0.46  0.14 -0.30  0.03 
Ca21Chr1_0337776 -0.04 -0.32  0.36  0.19  0.28  0.37 
Ca21Chr1_0337835 -0.31  0.41  0.47 -0.26  0.03  0.39 
Ca21Chr1_0337894  0.18  0.41 -0.02  0.05  0.09  0.05 
Ca21Chr1_0337953  1.82  0.25  0.39  0.33  0.17  0.34 
Ca21Chr1_0338012  1.15  0.41 -0.08  0.14  0.62  0.31 
Ca21Chr1_0338071  0.01  0.06 -0.03 -0.02  0.02  0.33 
Ca21Chr1_0338130 -0.19 -0.11  0.12  0.02  0.03  0.65 
Ca21Chr1_0338189  0.04 -0.00 -0.05 -0.23  0.20 -0.23 
Ca21Chr1_0338248 -0.11 -0.19  0.07  0.04  0.24  0.12 
Ca21Chr1_0338307  0.12  0.17  0.20  0.12  0.00  0.53 
Ca21Chr1_0338366  0.10  0.22  0.11  0.11  0.20  0.14 
Ca21Chr1_0338425 -0.24 -0.12  0.41  0.47 -0.48  0.45 
Ca21Chr1_0338484 -0.01  0.07  0.33  0.48 -0.39 -0.02 
Ca21Chr1_0338543  0.13  0.10  0.12  0.19 -0.36 -0.23 
Ca21Chr1_0338602  0.04  0.17  0.40  0.09 -0.13  0.22 
Ca21Chr1_0338661  0.24  0.05  0.38  0.21  0.24  0.00 
Ca21Chr1_0338720  0.45  0.14  0.43  0.23  0.28 -0.11 
Ca21Chr1_0338779  0.29  0.24  0.33 -0.37 -0.11 -0.08 
Ca21Chr1_0338838  0.25 -0.04  0.49  0.09 -0.16  0.24 
Ca21Chr1_0338897  0.16  0.23  0.25  0.06  0.04  0.33 
Ca21Chr1_0338956  0.89 -0.29  0.56  0.25  0.10 -0.63 
Ca21Chr1_0339015 -0.54  0.20 -0.76 -0.46 -0.52 -1.06 
Ca21Chr1_0339074  0.48  0.26  0.26  0.15 -0.26  0.24 
Ca21Chr1_0339133  0.19 -0.02 -0.09 -0.21  0.22 -0.25 
Ca21Chr1_0339192 -0.29  0.21  0.36 -0.16  0.70  0.59 
Ca21Chr1_0339251  0.01  0.10  0.12  0.11 -0.29  0.08 
Ca21Chr1_0339310  0.19  0.23  0.82  0.66 -0.42  0.73 
Ca21Chr1_0339369  1.09  0.10  0.08  0.24  0.02 -0.31 
Ca21Chr1_0339428  0.12  0.12 -0.00  0.59 -0.29  1.08 
Ca21Chr1_0339487  0.24  0.23  0.67  0.42 -0.13  0.54 
Ca21Chr1_0339546  0.37  0.26 -0.01  0.07  0.10  0.70 
Ca21Chr1_0339605 -0.09 -0.08  0.22 -0.06  0.05  0.50 
Ca21Chr1_0339664  0.04 -0.19  0.59  0.64 -0.09  0.03 
Ca21Chr1_0339723  0.13  0.31  0.30 -0.40  0.14  0.79 
Ca21Chr1_0339782  0.03  0.47  0.25  0.37  0.21  0.17 
Ca21Chr1_0339841 -0.03  0.49  0.35 -0.36  0.27 -0.08 
Ca21Chr1_0339900 -0.22  0.50  0.42  0.17  0.20  0.22 
Ca21Chr1_0339959  0.41  0.19  0.55  0.09  0.01 -0.12 
Ca21Chr1_0340018  0.54  0.04  0.41  0.36 -0.14  0.40 
Ca21Chr1_0340077  0.09  0.04  0.27  0.36  0.25 -0.28 
Ca21Chr1_0340136 -0.33  0.06  0.44  0.07  0.04  0.04 
Ca21Chr1_0340195  0.08  0.12  0.44  0.44 -0.11 -0.09 
Ca21Chr1_0340254 -0.13 -0.17  0.16  0.06  0.27 -0.01 
Ca21Chr1_0340313  0.13  0.16  0.15  0.40  0.05  0.31 
Ca21Chr1_0340372  0.09  0.30  0.28 -0.25 -0.29  0.28 
Ca21Chr1_0340431  0.52  0.17  0.52  0.13 -0.23  0.19 
Ca21Chr1_0340490  0.09  0.18  0.22 -0.07 -0.02 -0.29 
Ca21Chr1_0340549  0.42  0.33  0.49  0.07  0.03 -0.11 
Ca21Chr1_0340608 -0.12  0.14  0.40  0.15  0.25 -0.74 
Ca21Chr1_0340667 -0.05  0.42  0.42 -0.19  0.02  0.40 
Ca21Chr1_0340726 -0.30  0.22  0.33  0.07 -0.25  0.01 
Ca21Chr1_0340785 -0.05  0.37  0.22 -0.13 -0.04  0.05 
Ca21Chr1_0340844  0.41  0.03 -0.04  0.24 -0.17 -0.67 
Ca21Chr1_0340903 -0.43  0.19  0.11  0.02  0.28  0.24 
Ca21Chr1_0340962 -0.47  0.35  0.25 -0.13 -0.50 -0.40 
Ca21Chr1_0341021  0.10  0.08  0.33  0.05 -0.20 -0.25 
Ca21Chr1_0341080  0.21  0.48  0.34 -0.22 -0.00  0.06 
Ca21Chr1_0341139  0.39 -0.28 -0.04 -0.19  0.21  1.37 
Ca21Chr1_0341198  0.29  0.34  0.23 -0.05  0.38  0.12 
Ca21Chr1_0341257 -0.01  0.44  0.02  0.20  0.02 -0.34 
Ca21Chr1_0341316  0.49  0.29  0.35  0.14  0.20 -0.16 
Ca21Chr1_0341375 -0.15  0.38  0.30  0.06 -0.33 -0.33 
Ca21Chr1_0341434  0.41  0.06  0.38  0.10 -0.04 -0.13 
Ca21Chr1_0341493  0.12 -0.25 -0.03  0.20 -0.17  0.01  <orf19.3356
Ca21Chr1_0341552 -0.04  0.20  0.20  0.25 -0.12 -0.24 
Ca21Chr1_0341611 -0.04  0.27  0.19  0.04  0.15 -0.35 
Ca21Chr1_0341670 -0.25  0.19  0.04 -0.22  0.59 -0.14 
Ca21Chr1_0341729 -0.01 -0.25 -0.52 -0.18 -0.24 -0.24 
Ca21Chr1_0341788  0.29  0.27 -0.07 -0.09  0.06 -0.07 
Ca21Chr1_0341847  0.57  0.12  0.41 -0.50 -0.05  0.23 
Ca21Chr1_0341906  0.46  0.06  0.21  0.01  0.15 -0.08  |orf19.3357
Ca21Chr1_0341965  0.36 -0.10  0.16 -0.27  0.08  0.00 
Ca21Chr1_0342024  0.36  0.16  0.22 -0.05  0.24  0.21 
Ca21Chr1_0342083  0.29 -0.00  0.11  0.37  0.25 -0.33 
Ca21Chr1_0342142  0.37  0.27  0.18 -0.18 -0.30  0.41 
Ca21Chr1_0342201  0.13 -0.02 -0.06 -0.16 -0.32 -0.16 
Ca21Chr1_0342260  0.18  0.13  0.20 -0.38 -0.08  0.09 
Ca21Chr1_0342319  0.38 -0.02  0.27 -0.34  0.01  0.55 
Ca21Chr1_0342378  0.00  0.40  0.41  0.26  0.15  0.05 
Ca21Chr1_0342437  1.02  0.33  0.31  0.18  0.10  0.09  <orf19.3357
Ca21Chr1_0342496 -0.13  0.07  0.15 -0.01  0.04  0.20 
Ca21Chr1_0342555 -0.58  0.04  0.36  0.34  0.20  0.00 
Ca21Chr1_0342614 -0.23  0.16  0.31 -0.08 -0.40 -0.67 
Ca21Chr1_0342673  0.83  0.03 -0.01  0.11 -0.21 -0.35 
Ca21Chr1_0342732  0.51 -0.12  0.17  0.35 -0.06  0.21 
Ca21Chr1_0342791 -0.30  0.25 -0.21 -0.15  0.78 -0.78 
Ca21Chr1_0342850  1.22 -0.20 -0.05  0.16  0.05  0.57 
Ca21Chr1_0342909  1.19  0.28  0.08  0.36  0.05 -0.18  |orf19.3358
Ca21Chr1_0342968  0.69 -0.15  0.17 -0.18  0.07  0.49 
Ca21Chr1_0343027  1.11  0.25  0.11  0.24  0.33 -0.13 
Ca21Chr1_0343086  0.65  0.82  0.30 -0.13  0.19 -0.10 
Ca21Chr1_0343145  0.34 -0.11  0.30 -0.84 -0.03 -0.34 
Ca21Chr1_0343204  0.51  0.21  0.21 -0.10  0.18 -0.17 
Ca21Chr1_0343263  0.97  0.22  0.62 -0.07  0.12  0.09 
Ca21Chr1_0343322  0.25  0.26  0.21  0.02  0.06 -0.12 
Ca21Chr1_0343381  0.44  0.29  0.42 -0.03 -0.08 -0.04 
Ca21Chr1_0343440  0.68  0.12  0.41 -0.26  0.14 -0.16 
Ca21Chr1_0343499  0.78  0.28  0.48 -0.09 -0.01 -0.13 
Ca21Chr1_0343558  1.10  0.36  0.58  0.07  0.28 -0.23 
Ca21Chr1_0343617  0.64  0.45  0.38 -0.24  0.19  0.21 
Ca21Chr1_0343676  1.01  0.32  0.81 -0.13  0.05  0.09 
Ca21Chr1_0343735  0.86  0.44  0.37 -0.08  0.14  0.04 
Ca21Chr1_0343794  0.87 -0.01  0.03 -0.07  0.23 -0.08 
Ca21Chr1_0343853  1.03  0.18 -0.01 -0.27 -0.03  0.10 
Ca21Chr1_0343912  0.80  0.21 -0.01 -0.26 -0.30  0.06  <orf19.3358
Ca21Chr1_0343971 -0.29  0.08 -0.03 -0.61 -0.26 -0.25 
Ca21Chr1_0344030  0.08  0.10 -0.21 -1.02 -0.60  0.18 
Ca21Chr1_0344089  1.16  0.11  0.24 -1.13 -0.16 -0.18 
Ca21Chr1_0344148  0.18 -0.30 -0.34 -1.27 -0.68  0.15 
Ca21Chr1_0344207 -0.09 -0.16  0.68 -1.40  0.01  0.75 
Ca21Chr1_0344266 -0.28 -0.10 -0.34 -1.29 -0.88 -0.10 
Ca21Chr1_0344325 -0.53 -0.18 -0.42 -1.12 -0.95 -0.43 
Ca21Chr1_0344384 -0.43 -0.36 -0.33 -1.27 -1.64 -0.62 
Ca21Chr1_0344443 -0.05 -0.20 -0.22 -1.09 -0.77 -0.40 
Ca21Chr1_0344502 -0.28 -0.05 -0.37 -1.22 -0.48 -0.27 
Ca21Chr1_0344561 -0.19 -0.36  0.05 -0.71 -0.62 -1.05 
Ca21Chr1_0344620 -0.47 -0.00  0.12 -1.59 -0.99 -0.47 
Ca21Chr1_0344679 -0.23 -0.63  0.13 -1.46 -0.72 -0.11 
Ca21Chr1_0344738 -0.05 -0.27 -0.10 -1.54 -0.95 -0.50 
Ca21Chr1_0344797  0.20 -0.30  0.16 -1.71 -0.96 -0.14 
Ca21Chr1_0344856 -0.07  0.02  0.20 -1.03 -1.11 -0.66 
Ca21Chr1_0344915 -0.16 -0.02  0.09 -1.52 -0.43 -0.12 
Ca21Chr1_0344974  0.23  0.22  0.49 -0.86 -0.90 -0.26 
Ca21Chr1_0345033  0.19  0.20  0.15 -1.15 -0.51 -0.15 
Ca21Chr1_0345092  0.10 -0.37  0.19 -0.78 -0.62 -0.01 
Ca21Chr1_0345151 -0.20 -0.09  0.11 -0.96 -0.52 -0.18 
Ca21Chr1_0345210 -0.11  0.11 -0.26 -0.28 -0.57 -0.19 
Ca21Chr1_0345269 -0.06  0.49 -0.14 -0.50 -0.24 -0.19 
Ca21Chr1_0345328 -0.31  0.27 -0.08 -0.50 -0.13  0.10 
Ca21Chr1_0345387 -0.21 -0.36 -0.16 -0.45 -0.25  0.11 
Ca21Chr1_0345446  0.25  0.27 -0.05 -0.35 -0.42 -0.00 
Ca21Chr1_0345505 -0.38 -0.27 -0.23 -0.81  0.20  0.18 
Ca21Chr1_0345564  0.03 -0.29 -0.59 -0.44 -0.25  0.18  orf19.3359>
Ca21Chr1_0345623 -0.48 -0.52 -0.36 -0.05 -0.01  0.21 
Ca21Chr1_0345682 -0.12 -0.24 -0.35 -0.02 -0.03  0.12 
Ca21Chr1_0345741  0.37 -0.05 -0.18 -0.00  0.70  0.05 
Ca21Chr1_0345800 -0.55 -0.34 -0.55  0.01  0.36 -0.14 
Ca21Chr1_0345859 -0.07 -0.01 -0.35 -0.30 -0.33 -0.03 
Ca21Chr1_0345918 -0.36 -0.34 -0.29 -0.23 -0.22  0.13 
Ca21Chr1_0345977 -0.24 -0.58 -0.20 -0.04  0.00  0.01 
Ca21Chr1_0346036  0.05 -0.69 -0.14 -0.23 -0.14 -0.20 
Ca21Chr1_0346095 -0.08 -0.31 -0.20  0.20  0.24  0.11 
Ca21Chr1_0346154  0.32 -0.32 -0.25  0.06  0.30  0.40 
Ca21Chr1_0346213  0.49 -0.30 -0.06  0.44  0.23 -0.92 
Ca21Chr1_0346272 -0.90 -0.13 -0.43  0.04 -0.55  0.50 
Ca21Chr1_0346331 -0.14  0.09  0.48  0.01  0.20 -0.16 
Ca21Chr1_0346390 -0.08 -0.52 -0.00  0.09  0.05 -0.66 
Ca21Chr1_0346449  0.23  0.03  0.06 -0.21  0.19 -0.67 
Ca21Chr1_0346508  0.29 -0.01  0.24  0.17  0.07 -0.14 
Ca21Chr1_0346567  0.42  0.45  0.12  0.06  0.20  0.10 
Ca21Chr1_0346626  0.10 -0.16 -0.07 -0.02 -0.89  0.90 
Ca21Chr1_0346685  0.27 -0.20  0.22 -0.40 -0.10  0.06 
Ca21Chr1_0346744 -0.03 -0.05  0.42 -0.24  0.24 -0.41 
Ca21Chr1_0346803  0.04 -0.06  0.12  0.14 -0.10  0.13 
Ca21Chr1_0346862 -0.17 -0.04  0.40 -0.06  0.10 -0.25 
Ca21Chr1_0346921 -0.18 -0.02  0.54  0.17  0.30 -0.03 
Ca21Chr1_0346980 -0.98  0.26  0.29  0.25 -0.11  0.24 
Ca21Chr1_0347039 -0.11  0.47  0.03  0.20  0.02 -0.11 
Ca21Chr1_0347098 -0.10 -0.02  0.48 -0.06  0.05 -1.07 
Ca21Chr1_0347157 -0.05  0.04  0.56  0.22 -0.15 -0.56 
Ca21Chr1_0347216  0.09 -0.04  0.34 -0.41 -0.04  0.15 
Ca21Chr1_0347275  0.52  0.15  0.32  0.02  0.49 -0.05 
Ca21Chr1_0347334  0.15  0.33 -0.12  0.31  0.06 -0.40 
Ca21Chr1_0347393  0.35  0.28  0.73 -0.02  0.02  0.06 
Ca21Chr1_0347452  0.40  0.43  0.10 -0.07  0.12  0.31 
Ca21Chr1_0347511  0.29  0.12  0.48  0.25  0.14 -0.35 
Ca21Chr1_0347570  0.59  0.26  0.24  0.18 -0.45  0.49 
Ca21Chr1_0347629 -0.52  0.27  0.39 -0.11  0.13  0.27 
Ca21Chr1_0347688  0.09 -0.03 -0.64 -0.03  0.59 -0.17 
Ca21Chr1_0347747 -0.35  0.12  0.21  0.08  0.90  0.80 
Ca21Chr1_0347806  0.12  0.01  0.15  0.17 -0.39 -0.53 
Ca21Chr1_0347865  1.26 -0.07  0.22  0.06  0.12 -0.45 
Ca21Chr1_0347924  0.02 -0.27  0.31 -0.10  0.51 -0.09 
Ca21Chr1_0347983  0.03  0.21 -0.27 -0.08  0.09  0.40 
Ca21Chr1_0348042  1.14  0.28  0.28  0.12 -0.03  0.47 
Ca21Chr1_0348101  0.35  0.09 -0.09 -0.13  0.24 -0.18 
Ca21Chr1_0348160 -0.22  0.14 -0.46  0.01 -0.25 -0.00 
Ca21Chr1_0348219 -0.05  0.28  0.97  0.37  0.21 -0.86 
Ca21Chr1_0348278  0.90 -0.00  0.44  0.03  0.02  0.28 
Ca21Chr1_0348337 -0.09 -0.16 -0.07  0.02  0.26 -0.12 
Ca21Chr1_0348396 -0.01  0.29  0.05  0.39 -0.24  0.01 
Ca21Chr1_0348455 -0.04  0.16  0.18 -0.03 -0.24  0.04 
Ca21Chr1_0348514 -0.15  0.12  0.38  0.10 -0.38 -0.20 
Ca21Chr1_0348573 -0.18 -0.12 -0.07  0.26 -0.32  0.63 
Ca21Chr1_0348632  1.08  0.29 -0.12 -0.32 -0.97  0.42 
Ca21Chr1_0348691 -0.34  0.08 -0.36  0.18 -0.55  1.08 
Ca21Chr1_0348750 -0.68  0.11 -0.48  0.23 -0.17 -0.03  orf19.3359|
Ca21Chr1_0348809 -0.37  0.04 -0.72  0.13 -0.50  0.58 
Ca21Chr1_0348868 -0.05  0.20 -0.13 -0.06 -0.33  1.15 
Ca21Chr1_0348927 -0.24  0.71 -0.19 -0.02 -0.36  0.69 
Ca21Chr1_0348986 -0.14 -0.48 -0.67  0.27 -0.24  1.37 
Ca21Chr1_0349045 -0.21  0.07  0.15  0.12 -0.28  0.78 
Ca21Chr1_0349104  0.12  0.21 -0.79 -0.39 -0.60  0.34 
Ca21Chr1_0349163  0.21 -0.16 -0.88 -0.32  0.40  0.47 
Ca21Chr1_0349222 -0.54 -0.16 -0.81 -0.28  0.12  0.57 
Ca21Chr1_0349281 -1.96 -0.47 -1.80 -0.41 -0.35  0.27 
Ca21Chr1_0349340 -1.90 -0.36 -1.68 -0.34  0.17  0.24 
Ca21Chr1_0349399 -1.89 -1.45 -1.44 -1.27 -0.24  0.34 
Ca21Chr1_0349458 -0.11 -0.73 -0.95  0.25  0.27  0.02 
Ca21Chr1_0349517 -1.75 -0.79 -1.29 -1.28 -0.17 -0.08 
Ca21Chr1_0349576 -1.56 -0.83 -0.52 -1.02  0.09 -0.19 
Ca21Chr1_0349635 -1.35 -0.76 -1.17 -0.70 -0.55 -0.04 
Ca21Chr1_0349694 -1.26 -0.41 -1.15 -0.80  0.04  0.17 
Ca21Chr1_0349753 -0.75 -0.37 -1.17 -0.54 -0.00  0.05 
Ca21Chr1_0349812 -0.28 -0.17 -1.27 -0.53 -0.14  0.12 
Ca21Chr1_0349871 -0.27 -0.33 -0.75  0.02 -0.48  0.19 
Ca21Chr1_0349930 -0.11 -0.23 -0.48 -0.42  0.05  0.69 
Ca21Chr1_0349989 -0.44  0.13 -0.25  0.12 -0.58 -0.03 
Ca21Chr1_0350048 -0.08  0.15  0.53 -0.12  0.20  0.15 
Ca21Chr1_0350107  0.13 -0.32 -0.54 -0.22  0.81  0.43 
Ca21Chr1_0350166  0.01  0.40 -0.48  0.06 -0.84  0.74 
Ca21Chr1_0350225 -0.51 -0.26 -0.19  0.09 -0.29  0.23 
Ca21Chr1_0350284 -1.46  0.08 -1.14  0.09 -0.23 -0.14 
Ca21Chr1_0350343 -0.52  0.12 -0.08  0.01 -0.05  0.52 
Ca21Chr1_0350402 -0.74 -0.17 -0.21  0.21 -0.13  0.91 
Ca21Chr1_0350461 -0.67 -0.11 -0.70  0.08  0.00  0.10 
Ca21Chr1_0350520 -0.57  0.19 -0.26 -0.04 -0.39  0.69 
Ca21Chr1_0350579 -0.30  0.25 -0.43 -0.28 -0.16  1.44 
Ca21Chr1_0350638 -0.12 -0.16 -0.59 -0.08  0.16 -0.61 
Ca21Chr1_0350697  0.21 -0.16 -0.40  0.07  0.24 -0.09 
Ca21Chr1_0350756  0.11  0.24 -0.30  0.11 -0.14  0.25 
Ca21Chr1_0350815  0.07  0.10 -0.10  0.14  0.23 -0.19 
Ca21Chr1_0350874 -0.42 -0.20 -0.96 -1.50 -0.30 -0.71 
Ca21Chr1_0350933  0.26 -0.23 -0.05 -0.19 -0.37 -0.04 
Ca21Chr1_0350992  0.17  0.29  0.34 -0.15  0.09 -0.08 
Ca21Chr1_0351051  0.64 -0.45  0.68  0.11 -0.16  0.09 
Ca21Chr1_0351110  2.02  0.31  0.43 -0.13 -0.06 -0.01  orf19.3360>
Ca21Chr1_0351169 -0.16  0.53  0.54 -0.03 -0.05  0.58 
Ca21Chr1_0351228 -0.13  0.24  0.45  0.12 -0.66  0.53 
Ca21Chr1_0351287 -0.24  0.08  0.28 -0.06 -0.46 -0.08 
Ca21Chr1_0351346 -0.45  0.54  0.55 -0.04 -0.05  0.43 
Ca21Chr1_0351405 -0.11 -0.12  0.18 -0.00  0.25 -0.80 
Ca21Chr1_0351464 -0.08 -0.02  0.24 -0.13 -0.12  0.36 
Ca21Chr1_0351523  0.28 -0.02  0.40  0.01 -0.13  0.51 
Ca21Chr1_0351582  0.03 -0.00  0.12  0.09 -0.21 -0.02 
Ca21Chr1_0351641 -0.12  0.03  0.31  0.13 -0.04  0.05 
Ca21Chr1_0351700  0.15  0.25  0.05 -0.08  0.01  0.27 
Ca21Chr1_0351759 -0.11  0.19  0.17  0.09 -0.32 -0.13 
Ca21Chr1_0351818  0.30 -0.05  0.37  0.09 -0.33 -0.36 
Ca21Chr1_0351877  0.83 -0.04 -0.05 -0.02  0.93 -0.07 
Ca21Chr1_0351936 -0.22  0.21  0.48  0.16 -0.17  0.03 
Ca21Chr1_0351995 -0.13  0.41  0.26 -0.33 -0.10 -0.34 
Ca21Chr1_0352054 -0.13  0.25  0.54  0.05  0.04 -0.65 
Ca21Chr1_0352113  0.16  0.22  0.14 -0.02 -0.04 -0.12 
Ca21Chr1_0352172  0.10  0.02  0.08 -0.05 -0.34 -0.15 
Ca21Chr1_0352231 -0.23 -0.18 -0.34  0.45 -0.18  0.08 
Ca21Chr1_0352290 -0.16  0.02  0.71  0.01 -0.72  0.10 
Ca21Chr1_0352349 -0.17 -0.25  0.41  0.22 -0.21 -0.69 
Ca21Chr1_0352408 -0.17  0.01  0.36 -0.03  0.10  0.10 
Ca21Chr1_0352467 -0.04  0.11  0.21  0.22 -0.46  0.28 
Ca21Chr1_0352526 -0.17 -0.07  0.26 -0.52 -0.46  1.39 
Ca21Chr1_0352585 -0.14  0.35  0.52  0.01  0.09 -0.06 
Ca21Chr1_0352644 -0.09  0.51  0.28 -0.26 -0.19  0.56 
Ca21Chr1_0352703  0.59 -0.10  0.27  0.06 -0.15 -0.19 
Ca21Chr1_0352762 -0.12 -0.63  0.46  0.54  0.05 -0.03 
Ca21Chr1_0352821 -0.09 -0.80 -0.17 -0.14  0.62  0.70 
Ca21Chr1_0352880  0.01  0.94  0.26 -0.23 -0.27 -0.59 
Ca21Chr1_0352939  0.14  0.00  0.15  0.19  0.08 -0.42 
Ca21Chr1_0352998 -0.02  0.45  0.10 -0.12  0.13  0.01 
Ca21Chr1_0353057 -0.28  0.27  0.48  0.20 -0.05 -0.10 
Ca21Chr1_0353116 -0.15  0.18  0.75 -0.11 -0.19 -0.37  orf19.3360|
Ca21Chr1_0353175  0.96  0.64  0.11 -0.22  0.42 -0.63 
Ca21Chr1_0353234 -0.18 -0.01 -0.49 -0.11 -0.40 -0.18 
Ca21Chr1_0353293  0.10 -0.27 -0.38  0.01 -0.06 -0.01 
Ca21Chr1_0353352 -0.17  0.95 -0.06  0.02 -0.13 -0.77 
Ca21Chr1_0353411 -0.66  0.54  1.39 -0.18  0.09 -0.48 
Ca21Chr1_0353470 -0.27  0.55 -0.63 -0.09 -0.00  0.96 
Ca21Chr1_0353529 -0.11  0.18 -0.26  0.17 -0.18  0.02 
Ca21Chr1_0353588 -0.17 -0.32  0.29 -0.22 -0.28 -0.15 
Ca21Chr1_0353647 -0.70 -0.47  0.08 -0.09 -0.28 -0.28 
Ca21Chr1_0353706  0.11 -0.24 -0.15 -0.10 -0.33  0.05 
Ca21Chr1_0353765 -0.13  0.04  0.07 -0.17 -0.34 -0.02 
Ca21Chr1_0353824  0.17 -0.09  0.15 -0.10 -0.39  0.18 
Ca21Chr1_0353883  0.33 -0.16 -0.22  0.10 -0.30 -0.27 
Ca21Chr1_0353942 -0.18 -0.15 -0.24 -0.04  0.03  0.32 
Ca21Chr1_0354001 -0.22 -0.21  0.56 -0.57 -0.38  0.08 
Ca21Chr1_0354060 -0.46 -0.03 -0.43  0.46 -0.20  0.46 
Ca21Chr1_0354119 -0.64 -0.17 -0.75 -0.49 -0.11  0.30 
Ca21Chr1_0354178 -0.10 -0.14 -0.24 -0.51 -0.62  0.20  tA(AGC)3>
Ca21Chr1_0354237 -0.47  0.62 -0.38 -0.08  0.07  0.40  tA(AGC)3|
Ca21Chr1_0354296 -0.47 -0.31 -0.42 -0.53 -0.18 -0.09 
Ca21Chr1_0354355 -0.34 -0.18 -0.33 -0.47 -0.01  0.13 
Ca21Chr1_0354414 -0.21 -0.04 -0.21 -0.29  0.01 -0.00 
Ca21Chr1_0354473 -0.11 -0.12 -0.11  0.06 -0.29  0.07 
Ca21Chr1_0354532  0.13 -0.02 -0.41  0.10 -0.12  0.15 
Ca21Chr1_0354591  0.09  0.41  0.10  0.18 -0.50  0.27 
Ca21Chr1_0354650  0.16  0.03 -0.10 -0.14 -0.41  0.03 
Ca21Chr1_0354709  0.08 -0.19  0.10 -0.10  0.40 -0.45 
Ca21Chr1_0354768  0.10 -0.07 -0.21 -0.07  0.02 -0.16 
Ca21Chr1_0354827 -0.22  0.02  0.05  0.33 -0.04  0.06 
Ca21Chr1_0354886 -0.10  0.78 -0.11  0.37  0.63 -0.22 
Ca21Chr1_0354945  0.09 -0.10 -0.21 -0.45  0.29 -0.13 
Ca21Chr1_0355004 -0.18 -0.14 -0.01  0.10  0.05  0.12 
Ca21Chr1_0355063  0.25  0.30  0.48  0.01  0.19  0.12 
Ca21Chr1_0355122  0.36  0.31  0.37  0.09 -0.03  0.14 
Ca21Chr1_0355181  0.04  0.15 -0.00 -0.01 -0.35 -0.09 
Ca21Chr1_0355240  0.40  0.21 -0.06  0.14  0.54  0.17 
Ca21Chr1_0355299  0.40  0.29  0.27  0.08  0.15  0.00 
Ca21Chr1_0355358  0.06  0.35  0.71  0.18 -0.11  0.02 
Ca21Chr1_0355417 -0.48  0.19  0.33  0.66  0.38  0.06 
Ca21Chr1_0355476 -0.07  0.44 -0.38  0.10 -0.27  0.08 
Ca21Chr1_0355535 -0.18  0.49 -0.08 -0.04  0.36  0.08 
Ca21Chr1_0355594 -0.09 -0.43 -0.16 -0.03 -0.10 -0.79 
Ca21Chr1_0355653 -0.09 -0.33 -0.51 -0.05  0.07 -0.27 
Ca21Chr1_0355712 -0.46 -0.17 -0.16 -0.07 -0.06 -0.43 
Ca21Chr1_0355771 -0.41 -0.26 -0.39  0.17 -0.90 -0.93 
Ca21Chr1_0355830 -0.28  0.04 -0.04  0.31 -0.24 -0.53 
Ca21Chr1_0355889 -0.08  0.18  0.23  0.19 -0.06 -0.42 
Ca21Chr1_0355948  0.33  0.07 -0.41  0.03  0.04  0.81 
Ca21Chr1_0356007  0.35 -0.07  0.11 -0.31 -0.45  0.65 
Ca21Chr1_0356066 -0.24  0.37  0.20  0.26 -0.23  0.11 
Ca21Chr1_0356125 -0.01  0.63  0.07  0.18 -0.04 -0.12 
Ca21Chr1_0356184 -0.08  0.40 -0.16  0.30 -0.25 -0.28 
Ca21Chr1_0356243 -0.09  0.04 -0.03 -0.13  0.02 -0.04 
Ca21Chr1_0356302 -0.34 -0.01 -0.22 -0.10  0.13  0.09 
Ca21Chr1_0356361 -0.19  0.10 -0.32 -0.44 -0.11 -0.22 
Ca21Chr1_0356420 -0.27 -0.15 -0.48 -0.69 -0.34 -0.24 
Ca21Chr1_0356479 -0.22 -0.37 -0.08 -0.34 -0.46  0.05 
Ca21Chr1_0356538 -0.46 -0.02 -0.48 -0.23 -0.29 -0.02 
Ca21Chr1_0356597 -0.35 -0.34 -0.56 -0.22 -0.53  0.12 
Ca21Chr1_0356656  0.42  0.15 -0.48  0.28  0.09 -0.59 
Ca21Chr1_0356715 -0.52 -0.24 -0.03  0.47 -0.15 -0.70 
Ca21Chr1_0356774 -0.33  0.02 -0.76 -0.04 -0.26 -0.54 
Ca21Chr1_0356833 -0.27  0.30 -0.07 -0.17  0.00  0.69 
Ca21Chr1_0356892  0.02  0.44 -0.06  0.12 -0.35  2.68 
Ca21Chr1_0356951 -0.40  0.35 -0.22 -0.11 -0.06  0.13 
Ca21Chr1_0357010  0.15  0.57  0.22 -0.12 -0.12  0.15 
Ca21Chr1_0357069  0.02  0.25 -0.06  0.12 -0.14 -0.01  orf19.4551>
Ca21Chr1_0357128  0.16 -0.04  0.43  0.26  0.09  0.19 
Ca21Chr1_0357187  0.73  0.21  0.37  0.12 -0.50  0.06 
Ca21Chr1_0357246  0.11  0.26  0.03  0.89 -0.63 -0.02 
Ca21Chr1_0357305  0.09  0.18  0.45  0.31  0.11  0.25 
Ca21Chr1_0357364 -0.01 -0.03  0.75 -0.29  0.16  0.33 
Ca21Chr1_0357423  0.06  0.30  0.06 -0.09 -0.04  0.50 
Ca21Chr1_0357482 -0.19  1.19  0.04  0.12  0.23  0.44 
Ca21Chr1_0357541 -0.33  0.40  0.61  0.26 -0.16  0.31 
Ca21Chr1_0357600  0.08  0.20  0.06  0.05 -0.30 -0.39 
Ca21Chr1_0357659  0.24  0.06 -0.03 -0.29 -0.18  0.16 
Ca21Chr1_0357718 -0.05  0.01 -0.04 -0.38 -0.18 -0.05 
Ca21Chr1_0357777  0.35  0.10  0.17  0.06 -0.25  0.07 
Ca21Chr1_0357836  0.19  0.13  0.20  1.16 -0.10 -0.44 
Ca21Chr1_0357895 -0.04 -0.00  0.18 -0.06  0.25 -0.13 
Ca21Chr1_0357954 -0.07  0.08 -0.10  0.29  0.28 -0.05 
Ca21Chr1_0358013 -0.29  0.37  0.32 -0.38 -0.31 -0.35 
Ca21Chr1_0358072 -0.01  0.28  0.33 -0.18 -0.16  0.22 
Ca21Chr1_0358131 -0.21  0.20  0.17 -0.00  0.19  0.15 
Ca21Chr1_0358190 -0.11  0.35  0.18 -0.09 -0.00 -0.14 
Ca21Chr1_0358249  0.01  0.14  0.16 -0.37  0.31  0.25 
Ca21Chr1_0358308  0.21  0.10  0.09 -0.16 -0.05 -0.05 
Ca21Chr1_0358367 -0.29  0.23  0.63  0.49  0.10 -0.63 
Ca21Chr1_0358426  0.33  0.21  0.12  0.11  0.27 -0.32 
Ca21Chr1_0358485  0.12 -0.01  0.38  0.33  0.61  0.30 
Ca21Chr1_0358544 -0.03  0.20  0.63  0.18  0.60  0.10 
Ca21Chr1_0358603 -0.04 -0.53  0.33  0.16  0.18  0.04 
Ca21Chr1_0358662  0.12  0.05  0.14 -0.17  0.44 -0.05 
Ca21Chr1_0358721  0.35  0.13 -0.01  0.11  0.13  0.12 
Ca21Chr1_0358780  0.12  0.63  0.17  0.47  0.15 -0.29 
Ca21Chr1_0358839  1.01  0.28  0.16 -0.37  0.36 -0.05 
Ca21Chr1_0358898  0.46  0.46  0.08  0.27  0.07 -0.36 
Ca21Chr1_0358957  0.21  0.15 -0.11 -0.23 -0.18  0.05 
Ca21Chr1_0359016  0.36  0.28 -0.08  0.22  0.10  0.01 
Ca21Chr1_0359075  0.13  0.24  0.19 -0.11  0.14 -0.12 
Ca21Chr1_0359134 -0.01 -0.07 -0.17 -0.05  0.10  0.23 
Ca21Chr1_0359193  0.11  0.21 -0.29 -0.18  0.05  0.12 
Ca21Chr1_0359252 -0.28 -0.17 -0.22 -0.18 -0.17  0.61 
Ca21Chr1_0359311 -0.17 -0.08 -0.25  0.17  0.19  0.01 
Ca21Chr1_0359370 -0.25  0.18 -0.25  0.56  0.02  0.19 
Ca21Chr1_0359429  0.94 -0.04  0.02  0.06 -0.10 -0.34  orf19.4551|
Ca21Chr1_0359488  1.06 -0.02 -0.75 -0.12  0.26 -0.15 
Ca21Chr1_0359547  0.53 -0.41 -0.04 -0.21  0.95 -0.15 
Ca21Chr1_0359606 -1.15 -0.59 -0.79  0.25  0.03 -0.18 
Ca21Chr1_0359665 -0.78 -0.33 -0.99  0.04 -0.15 -0.18 
Ca21Chr1_0359724 -0.40 -0.50 -0.74 -0.18  0.04  0.14 
Ca21Chr1_0359783 -0.17 -0.28 -0.72  0.14  0.28  0.22 
Ca21Chr1_0359842 -0.54  0.02 -0.46  0.04 -0.16  0.79 
Ca21Chr1_0359901 -0.95 -0.02 -0.66  0.40 -0.26  0.30 
Ca21Chr1_0359960  0.12 -0.79 -0.98 -0.10 -0.13 -0.44 
Ca21Chr1_0360019 -0.27 -0.46 -0.99  0.20 -0.62 -0.14 
Ca21Chr1_0360078 -0.69 -0.58 -1.34 -0.40 -0.16 -0.10 
Ca21Chr1_0360137 -0.98 -0.70 -1.42 -0.51 -0.05 -0.15 
Ca21Chr1_0360196 -0.53 -1.37 -1.59 -0.17 -0.56 -0.27 
Ca21Chr1_0360255 -1.14 -1.31 -1.28 -0.54 -0.19  0.09 
Ca21Chr1_0360314 -1.11 -1.55 -1.61 -0.01 -0.34  0.44 
Ca21Chr1_0360373 -0.36 -1.14 -1.03  0.10 -0.60  0.40 
Ca21Chr1_0360432 -1.04 -0.79 -1.49 -0.34 -0.02  0.15 
Ca21Chr1_0360491 -1.09 -0.84 -1.63 -0.04 -0.48  0.33 
Ca21Chr1_0360550 -1.08  0.10 -1.70  0.15  0.15 -0.97 
Ca21Chr1_0360609 -1.36 -0.45 -1.70 -0.01 -0.33  0.39 
Ca21Chr1_0360668 -2.24 -0.80 -1.87  0.28 -0.34  0.16 
Ca21Chr1_0360727 -1.17 -1.31 -1.83  0.17  0.08 -0.24 
Ca21Chr1_0360786 -0.58 -0.06 -1.29  0.05 -0.27  0.22 
Ca21Chr1_0360845  0.08 -0.48 -0.29 -0.40  0.11  0.08 
Ca21Chr1_0360904  0.91 -0.21  0.17 -0.02 -0.11 -0.46 
Ca21Chr1_0360963 -0.10 -0.43 -0.07 -0.13  0.11 -0.17 
Ca21Chr1_0361022  0.75 -0.16  0.06  0.08 -0.01  0.04  orf19.4550>
Ca21Chr1_0361081  0.21  0.36  0.16 -0.23  0.22 -0.11 
Ca21Chr1_0361140  0.03  0.24  0.09  0.11 -0.06  0.01 
Ca21Chr1_0361199 -0.23  0.02  0.03  0.08 -0.17 -0.08 
Ca21Chr1_0361258  0.04 -0.50  0.02 -0.60 -0.21  0.21 
Ca21Chr1_0361317 -0.30  0.08 -0.06 -0.18 -0.38  0.26 
Ca21Chr1_0361376  0.54  0.11  0.21 -0.18  0.11 -0.00 
Ca21Chr1_0361435 -0.00  0.17  0.29  0.29 -0.13  0.10 
Ca21Chr1_0361494  0.37  0.05  0.08 -0.03  0.12  0.04 
Ca21Chr1_0361553  0.19  0.21  0.28  0.35 -0.06 -0.14 
Ca21Chr1_0361612  0.14  0.37  0.29  0.60 -0.01  0.24 
Ca21Chr1_0361671  0.18  0.23 -0.12  0.05 -0.41  0.81 
Ca21Chr1_0361730  0.14  0.15  0.15 -0.08 -0.28  0.12 
Ca21Chr1_0361789  0.63 -0.42  0.16  0.09  0.17 -0.11 
Ca21Chr1_0361848  0.19  0.05 -0.06  0.20  0.02  0.19 
Ca21Chr1_0361907 -0.10  0.57  0.07  0.15 -0.07 -0.32 
Ca21Chr1_0361966 -0.32  0.26  0.10  0.36 -0.22 -0.30 
Ca21Chr1_0362025 -0.09  0.08  0.17  0.30  0.13 -0.24 
Ca21Chr1_0362084 -0.17  0.24  0.46  0.19 -0.39  0.53 
Ca21Chr1_0362143 -0.05  0.49  0.32  0.03  0.10  0.31 
Ca21Chr1_0362202  0.22  0.18  0.45 -0.18 -0.30 -0.06 
Ca21Chr1_0362261  0.49  0.43  0.48 -0.13  0.02  0.17 
Ca21Chr1_0362320  0.38  0.09  0.19 -0.38 -0.50 -0.10 
Ca21Chr1_0362379  0.43  0.34  0.41  0.01 -0.08  0.32 
Ca21Chr1_0362438  0.24  0.27  0.15  0.10 -0.13  0.23 
Ca21Chr1_0362497  0.36  0.02  0.21  0.38  0.37  1.04 
Ca21Chr1_0362556  0.03  0.22  0.00  0.20 -0.08 -0.32 
Ca21Chr1_0362615  0.21 -0.52 -0.03 -0.13 -0.47  0.20 
Ca21Chr1_0362674  0.60  0.10  0.02 -0.08 -0.20  0.01 
Ca21Chr1_0362733  0.05 -0.07  0.58  0.20 -0.31  0.24 
Ca21Chr1_0362792 -0.69  0.16  0.20  0.12 -0.06  0.06 
Ca21Chr1_0362851  0.10  0.08  0.14  0.12  0.08  0.13 
Ca21Chr1_0362910 -0.05 -0.01 -0.06  0.17 -0.10  0.35  orf19.4550|
Ca21Chr1_0362969 -0.04 -0.23 -0.23 -0.34 -0.41  0.38 
Ca21Chr1_0363028 -0.50  0.07  0.57  0.00 -0.33  1.07 
Ca21Chr1_0363087 -0.41  0.59  0.08  0.05 -0.63  0.55 
Ca21Chr1_0363146  0.34 -0.17  0.29  0.06 -0.03  0.97 
Ca21Chr1_0363205 -0.38  0.42  0.05 -0.11 -0.23  1.17 
Ca21Chr1_0363264 -0.90  0.12 -0.32 -0.04  0.01  1.20 
Ca21Chr1_0363323 -0.25 -0.10  0.31 -0.23 -0.13  0.64 
Ca21Chr1_0363382 -0.25 -0.06  0.21 -0.18 -0.20  1.31 
Ca21Chr1_0363441 -0.57  0.14 -0.07  0.05 -0.13  0.70 
Ca21Chr1_0363500 -0.10  0.36 -0.37 -0.33  0.36 -0.09 
Ca21Chr1_0363559 -0.34 -0.13 -0.73 -0.42 -0.35 -0.36 
Ca21Chr1_0363618 -0.18 -0.41 -0.71 -0.06  0.31  0.40 
Ca21Chr1_0363677 -0.52  0.29 -0.48 -0.03  0.13  0.31 
Ca21Chr1_0363736 -0.19  0.25  0.13  0.05  0.00  0.29 
Ca21Chr1_0363795 -0.20 -0.10 -0.56 -0.11  0.16  0.23 
Ca21Chr1_0363854 -0.20  0.10  0.00 -0.22 -0.07  1.04 
Ca21Chr1_0363913  0.02 -0.22 -0.05 -0.19  0.06  1.27 
Ca21Chr1_0363972  0.05 -0.20  0.01 -0.31 -0.22 -0.80 
Ca21Chr1_0364031  0.31  0.10  0.50 -0.06  0.16 -0.02 
Ca21Chr1_0364090  0.28 -0.03  0.25  0.40  0.42  0.10 
Ca21Chr1_0364149  0.17  0.13  0.27 -0.13 -0.07  0.00  orf19.4549>
Ca21Chr1_0364208 -0.62  0.34  0.36 -0.54 -0.15 -0.46 
Ca21Chr1_0364267  0.19 -0.05  0.40  0.00  0.21 -0.41 
Ca21Chr1_0364326  0.18 -0.10  0.55  0.29 -0.03 -0.14 
Ca21Chr1_0364385  0.28  0.14  0.20  0.12 -0.01 -0.06 
Ca21Chr1_0364444  0.13  0.30  0.31 -0.08  0.60 -0.17 
Ca21Chr1_0364503  0.13  0.30  0.29 -0.37  0.20 -0.03 
Ca21Chr1_0364562  0.16  0.34  0.22 -0.03  0.14 -0.25 
Ca21Chr1_0364621  0.25  0.10  0.26 -0.43  0.24 -0.16 
Ca21Chr1_0364680 -0.07  0.19  0.51 -0.03  0.05 -0.14 
Ca21Chr1_0364739  0.11  0.22  0.27  0.15  0.44 -0.08 
Ca21Chr1_0364798  0.11 -0.07  0.54  0.22 -0.22  0.06 
Ca21Chr1_0364857  0.14  0.01  0.51  0.13  0.00  0.47 
Ca21Chr1_0364916  0.08  0.03  0.02  0.16  0.29  0.18 
Ca21Chr1_0364975 -0.01  0.21  0.50  0.29  0.01  0.25 
Ca21Chr1_0365034  0.48 -0.07  0.14  0.20  0.18 -0.01 
Ca21Chr1_0365093  0.93  0.30  0.29  0.08 -0.44  0.52 
Ca21Chr1_0365152  0.18  0.37  0.17 -0.19  0.07 -0.03 
Ca21Chr1_0365211 -0.06  0.19  0.41  0.10 -0.16 -0.01 
Ca21Chr1_0365270  0.24  0.02  1.05 -0.24  0.15 -0.35 
Ca21Chr1_0365329 -0.17  0.38  0.12  0.27  0.10  0.11 
Ca21Chr1_0365388  0.33  0.39  0.46  0.28 -0.33  0.11 
Ca21Chr1_0365447  0.34  0.29  0.26  0.36 -0.32  0.22 
Ca21Chr1_0365506  0.28  0.06 -0.26  0.06 -0.46  0.17 
Ca21Chr1_0365565  0.08  0.19  0.43  0.12 -0.27  0.41 
Ca21Chr1_0365624  1.17 -0.02  0.31 -0.18  0.33  0.06 
Ca21Chr1_0365683 -0.08  0.05  0.36  0.32 -0.15 -0.15 
Ca21Chr1_0365742  1.00  0.15  0.31 -0.17 -0.09  0.01 
Ca21Chr1_0365801  0.10  0.01  0.20  0.10  0.01 -0.50 
Ca21Chr1_0365860  0.26  0.15  0.32 -0.14 -0.04  0.53 
Ca21Chr1_0365919  0.34  0.20  0.29 -0.33 -0.19 -0.27 
Ca21Chr1_0365978 -0.39  0.14 -0.01  0.01 -0.08 -0.20 
Ca21Chr1_0366037  0.18  0.43  0.21 -0.17 -0.15 -0.24 
Ca21Chr1_0366096  0.03  0.41  0.22 -0.32 -0.20  0.29 
Ca21Chr1_0366155  0.40  0.31  0.40 -0.21  0.04 -0.21 
Ca21Chr1_0366214 -0.14  0.26  0.07  0.21  0.11 -0.14 
Ca21Chr1_0366273 -0.07 -0.15  0.01 -0.00 -0.30 -0.03  orf19.4549|
Ca21Chr1_0366332 -0.31  0.23 -0.25  0.14  0.08  0.07 
Ca21Chr1_0366391 -0.23  0.10  0.03  0.20 -0.42 -0.28 
Ca21Chr1_0366450  0.55 -0.06 -0.06  0.06 -0.63 -0.08 
Ca21Chr1_0366509  0.29  0.08 -0.05  0.23 -0.25 -0.02 
Ca21Chr1_0366568  0.11 -0.04 -0.56 -0.09 -0.27 -0.05 
Ca21Chr1_0366627 -0.02  0.31 -0.09  0.31 -0.40  0.06 
Ca21Chr1_0366686  0.83  0.20  0.48  0.34 -0.06 -0.48 
Ca21Chr1_0366745  0.26 -0.07  0.07  0.01  0.31 -0.21  orf19.4548>
Ca21Chr1_0366804  0.25  0.12  0.28  0.16 -0.05 -0.19 
Ca21Chr1_0366863 -0.11  0.27  0.90  0.07 -0.04 -0.11 
Ca21Chr1_0366922  0.59  0.10 -0.06  0.13  0.04  0.38 
Ca21Chr1_0366981  0.06  0.10  1.04  0.19 -0.42  0.16 
Ca21Chr1_0367040  0.49 -0.03  0.57 -0.24  0.33 -0.03 
Ca21Chr1_0367099 -0.45  0.42 -0.04  0.01  0.21  0.78 
Ca21Chr1_0367158 -0.54  0.13  0.11  0.57  0.67 -0.13 
Ca21Chr1_0367217 -0.06 -0.02 -0.03  0.55  0.06  0.31 
Ca21Chr1_0367276 -0.28 -0.06 -0.27  0.06 -0.27  0.29 
Ca21Chr1_0367335 -0.44  0.01  0.38  0.31 -0.00 -0.36 
Ca21Chr1_0367394 -0.01 -0.30 -0.11 -0.03 -0.20 -0.04 
Ca21Chr1_0367453 -0.02  0.09 -0.09  0.17 -0.27 -0.13 
Ca21Chr1_0367512 -0.11  0.06 -0.09 -0.34 -0.16 -0.25 
Ca21Chr1_0367571 -0.16  0.26  0.18  0.88  0.27  0.81 
Ca21Chr1_0367630  0.18  0.25  0.18  0.49  0.08  0.31 
Ca21Chr1_0367689  0.03  0.30  0.17  0.44 -0.08  0.32 
Ca21Chr1_0367748  0.08 -0.10  0.02  0.10 -0.12  0.33 
Ca21Chr1_0367807  0.26  0.07 -0.26  0.33 -0.07 -0.92 
Ca21Chr1_0367866  0.32  0.02  0.27  0.02 -0.27 -0.07  orf19.4548|
Ca21Chr1_0367925 -0.03  0.15  0.28  0.55  0.12 -0.46  |orf19.4546
Ca21Chr1_0367984  0.18  0.36  0.42  0.08 -0.32  0.15 
Ca21Chr1_0368043  0.17  0.57  0.23  0.28  0.35  0.03 
Ca21Chr1_0368102  0.21  0.05  0.30 -0.03  0.18  0.06 
Ca21Chr1_0368161  0.51  0.26  0.24  0.16 -0.26 -0.03 
Ca21Chr1_0368220  0.48  0.22  0.30  0.22  0.20 -0.33 
Ca21Chr1_0368279  0.13  0.08  0.43  0.04  0.37  0.06 
Ca21Chr1_0368338  0.38  0.42  0.46  0.57  0.15  0.07 
Ca21Chr1_0368397 -0.10 -0.10  0.34 -0.04 -0.04 -0.53 
Ca21Chr1_0368456  0.27 -0.03  0.30  0.38  0.26 -0.21 
Ca21Chr1_0368515  0.05  0.06  0.26  0.39  0.20 -0.25 
Ca21Chr1_0368574  0.08  0.02  0.26  0.09  0.56  0.05 
Ca21Chr1_0368633 -0.24  0.34  0.63  0.03  0.19  0.22 
Ca21Chr1_0368692 -0.20  0.67  0.43 -0.04  0.12  0.17 
Ca21Chr1_0368751  0.13  0.30  0.23 -0.12  0.08  0.00 
Ca21Chr1_0368810  0.49  0.42  0.36  0.08  0.14 -0.11 
Ca21Chr1_0368869  0.28  0.63  0.28 -0.19  0.02 -0.07 
Ca21Chr1_0368928  0.22  0.15  0.43  0.26 -0.03 -0.01 
Ca21Chr1_0368987  0.19  0.39  0.04 -0.10  0.06  0.05 
Ca21Chr1_0369046  0.25  0.26 -0.25  0.03 -0.42 -0.01 
Ca21Chr1_0369105 -0.04  0.25  0.23  0.10  0.06 -0.17 
Ca21Chr1_0369164  0.19  0.23  0.28  0.14  0.38 -0.05 
Ca21Chr1_0369223  0.01  0.13  0.21 -0.21  0.23 -0.29 
Ca21Chr1_0369282 -0.55  0.12  0.26  0.31  0.19 -0.03 
Ca21Chr1_0369341  0.14 -0.24  0.19  0.08  0.35  0.10 
Ca21Chr1_0369400 -0.06 -0.19  0.20  0.28  0.18 -0.03 
Ca21Chr1_0369459 -0.81 -0.31 -0.34  0.14 -0.01  0.21 
Ca21Chr1_0369518 -0.82  0.18  0.62 -0.17  0.16 -0.73 
Ca21Chr1_0369577 -0.15 -0.08  0.28  1.26 -0.63 -0.06 
Ca21Chr1_0369636  0.13  0.48  0.58  0.18  0.05 -0.06 
Ca21Chr1_0369695  0.05 -0.04  0.15  0.16  0.18  0.14 
Ca21Chr1_0369754  0.18  0.13  0.34  0.17  0.00  0.32 
Ca21Chr1_0369813 -0.20 -0.31 -0.16 -0.20 -0.17 -0.16  <orf19.4546
Ca21Chr1_0369872 -0.23 -0.46 -0.29 -0.34  0.13  0.18 
Ca21Chr1_0369931 -0.22 -0.13 -0.17 -0.06 -0.16  0.64 
Ca21Chr1_0369990  0.06 -0.34 -0.26  0.11  0.12  0.40 
Ca21Chr1_0370049 -0.53 -0.48 -0.36  0.44 -0.02  0.49 
Ca21Chr1_0370108 -0.34 -0.07 -0.89  0.18 -0.19  0.22 
Ca21Chr1_0370167 -0.45 -0.12 -1.13 -0.25 -0.39  0.08 
Ca21Chr1_0370226 -0.62 -0.20 -1.48 -0.05  0.16  1.11 
Ca21Chr1_0370285 -0.57 -1.05 -2.00 -0.39  0.32  1.06 
Ca21Chr1_0370344 -1.02 -1.51 -2.37 -0.40  0.02  0.90 
Ca21Chr1_0370403 -1.10 -1.62 -1.86 -0.39  0.24  0.39 
Ca21Chr1_0370462 -1.34 -0.50 -1.76 -0.07  0.00  0.42 
Ca21Chr1_0370521 -0.40 -1.39 -2.09  0.09 -0.19  0.16 
Ca21Chr1_0370580 -1.14 -1.20 -1.99  0.56 -0.25  0.32 
Ca21Chr1_0370639 -1.51 -1.30 -1.73  0.01 -0.21  0.72 
Ca21Chr1_0370698 -1.47 -1.78 -1.58 -0.12  0.11  0.29 
Ca21Chr1_0370757 -0.44 -1.46 -1.01 -0.05 -0.18 -0.04 
Ca21Chr1_0370816 -0.30 -0.61 -0.91  0.13  0.07 -0.22 
Ca21Chr1_0370875 -0.07 -0.75 -0.57  0.08 -0.18 -0.30 
Ca21Chr1_0370934 -0.14 -0.42 -0.49  0.23 -0.19  0.67 
Ca21Chr1_0370993 -0.09 -0.22 -0.44  0.25  0.07 -0.32 
Ca21Chr1_0371052 -0.18 -0.02 -0.36  0.05 -0.79  0.33 
Ca21Chr1_0371111 -0.40 -0.20 -0.24 -0.45 -0.56  0.37 
Ca21Chr1_0371170  0.05  0.03 -0.13  0.12  0.17  0.72 
Ca21Chr1_0371229 -0.73  0.05  0.07 -0.35  0.15  0.69 
Ca21Chr1_0371288 -0.04 -0.25  0.08  0.02 -0.33  0.86 
Ca21Chr1_0371347 -0.49 -0.03  0.09 -0.58  0.10  0.77 
Ca21Chr1_0371406 -0.24 -0.13 -0.15  0.28 -0.53  0.34 
Ca21Chr1_0371465 -0.31 -0.08 -0.18 -0.07 -0.01  0.03 
Ca21Chr1_0371524 -0.38 -0.00  0.06 -0.03 -0.14 -0.14 
Ca21Chr1_0371583 -0.85  0.22 -0.27 -0.26  0.12  0.27 
Ca21Chr1_0371642  0.08  0.00  0.23 -0.00  0.19 -0.13 
Ca21Chr1_0371701 -0.15  0.27  0.11 -0.04 -0.18  0.17 
Ca21Chr1_0371760 -0.08  0.24  0.07 -0.06 -0.09  0.32 
Ca21Chr1_0371819  0.16  0.29  0.07  0.01 -0.24  0.15 
Ca21Chr1_0371878 -0.24  0.16 -0.25 -0.42 -0.08  0.07 
Ca21Chr1_0371937  0.06 -0.05 -0.25 -0.33  0.12  0.88 
Ca21Chr1_0371996    NA    NA    NA    NA    NA    NA
Ca21Chr1_0372055  0.04 -0.06 -0.22  0.23  0.36 -0.04 
Ca21Chr1_0372114 -0.05  0.12 -0.74 -0.19 -0.38  0.21 
Ca21Chr1_0372173  0.76  0.05 -0.33 -0.06 -0.07  0.39 
Ca21Chr1_0372232 -0.27 -0.07 -0.43  0.28 -0.26  0.47  orf19.4545>
Ca21Chr1_0372291 -0.12 -0.46 -0.85 -0.26 -0.04  0.49 
Ca21Chr1_0372350 -0.06 -0.16 -0.26  0.02 -0.09 -0.23 
Ca21Chr1_0372409 -0.29 -0.24  0.13  0.40  0.08 -0.07 
Ca21Chr1_0372468  0.30 -0.38  0.16  0.06  0.14 -0.32 
Ca21Chr1_0372527 -0.09 -0.11  0.06 -0.16 -0.70  0.13 
Ca21Chr1_0372586  0.15  0.10  0.27  0.18  0.11 -0.21 
Ca21Chr1_0372645  0.12  0.12  0.10 -0.20  0.21 -0.20 
Ca21Chr1_0372704  0.33 -0.12  0.18 -0.09  0.19 -0.00 
Ca21Chr1_0372763  0.22 -0.01  1.21  0.09  0.05 -0.17 
Ca21Chr1_0372822  0.10  0.11  0.65 -0.15 -0.03 -0.70 
Ca21Chr1_0372881  0.18  0.24  0.35 -0.32  0.35 -0.59 
Ca21Chr1_0372940 -0.21 -0.05  0.23 -0.02  0.20  0.06 
Ca21Chr1_0372999 -0.03  0.02 -0.08 -0.15  0.19 -0.52 
Ca21Chr1_0373058  0.37  0.29  0.21 -0.46  0.09 -0.49 
Ca21Chr1_0373117  0.14  0.27  0.21  0.12  0.11 -0.20 
Ca21Chr1_0373176  0.53  0.40  0.27  0.09  0.08  0.17 
Ca21Chr1_0373235  0.05  0.22  0.34 -0.09  0.11 -0.03 
Ca21Chr1_0373294  0.48  0.46  0.42 -0.23  0.48  0.11 
Ca21Chr1_0373353  0.05 -0.01  0.30  0.10  0.26  0.14 
Ca21Chr1_0373412  0.15 -0.07  0.47 -0.14  0.36 -0.04 
Ca21Chr1_0373471  0.19 -0.04  0.21 -0.08  0.09 -0.21 
Ca21Chr1_0373530  0.30  0.14  0.24  0.18  0.57 -0.28 
Ca21Chr1_0373589  0.11  0.07  0.38  0.23  0.09 -0.47 
Ca21Chr1_0373648  0.33  0.41  0.36  0.07  0.01 -0.20 
Ca21Chr1_0373707  0.09  0.28 -0.22 -0.38  0.51  0.09 
Ca21Chr1_0373766 -0.21  0.18  0.33  0.34 -0.06 -0.03 
Ca21Chr1_0373825  0.31  0.29  0.24  0.28 -0.18  0.01 
Ca21Chr1_0373884  0.29 -0.03  0.59  0.18 -0.00 -0.09 
Ca21Chr1_0373943  0.17 -0.09  0.18  0.28 -0.19 -0.46 
Ca21Chr1_0374002  0.09  0.45  0.33 -0.07  0.08 -0.60 
Ca21Chr1_0374061  0.33  0.29  0.32  0.09  0.11 -0.25 
Ca21Chr1_0374120  0.43  0.11  1.20  0.31 -0.11  0.05 
Ca21Chr1_0374179 -0.08  0.46  0.30  0.39  0.17 -0.05 
Ca21Chr1_0374238 -0.01  0.31  0.57  0.08 -0.04  0.05 
Ca21Chr1_0374297  0.20  0.62  0.29  0.13  0.25 -0.23 
Ca21Chr1_0374356 -0.09  0.38 -0.06  0.14 -0.17 -0.31 
Ca21Chr1_0374415  0.15  0.11  0.48  0.12 -0.21 -0.12 
Ca21Chr1_0374474  0.20  0.21  0.31  0.11 -0.02 -0.11 
Ca21Chr1_0374533  0.18  0.27  0.20  0.19  0.23 -0.52 
Ca21Chr1_0374592  0.01 -0.20  0.30  0.27  0.38 -0.16 
Ca21Chr1_0374651  0.23  0.21  0.51  0.22  0.35 -0.12 
Ca21Chr1_0374710  0.44  0.89  0.32  0.57  0.44 -0.21 
Ca21Chr1_0374769  0.29  0.25  0.38  0.32  0.21 -0.33 
Ca21Chr1_0374828  0.16  0.22  0.16  0.23  0.06  0.01 
Ca21Chr1_0374887 -0.20  0.03  0.08  0.12  0.01  0.17 
Ca21Chr1_0374946 -0.37 -0.02 -0.05  0.59  0.12  0.09 
Ca21Chr1_0375005  0.08  0.08 -0.25  0.04 -0.66  0.80 
Ca21Chr1_0375064 -0.11  0.20 -0.09  0.03 -0.15 -0.11 
Ca21Chr1_0375123 -0.13  0.06 -0.22 -0.10 -0.37  0.27 
Ca21Chr1_0375182  0.11  0.27 -0.13  0.37  0.31 -0.00 
Ca21Chr1_0375241  0.59  0.22 -0.26  0.08 -0.66  0.36 
Ca21Chr1_0375300 -0.18  0.16 -0.09 -0.12 -0.19  0.27 
Ca21Chr1_0375359 -0.29 -0.02 -0.48 -0.04 -0.04  0.07 
Ca21Chr1_0375418 -0.41 -0.61 -0.35 -0.03 -0.12  0.16  orf19.4545|
Ca21Chr1_0375477 -0.03 -0.20 -0.49  0.11  0.12  0.23 
Ca21Chr1_0375536  0.40 -0.17 -0.17  0.07 -0.11  0.64 
Ca21Chr1_0375595 -0.04  0.10 -0.05  0.05 -0.26  0.22 
Ca21Chr1_0375654 -0.28 -0.69 -0.01 -0.00 -2.84  0.27 
Ca21Chr1_0375713 -0.00 -0.10 -0.26 -0.30 -0.13  0.04 
Ca21Chr1_0375772  0.17 -0.52  0.47  0.46 -0.08 -0.04 
Ca21Chr1_0375831 -0.37 -0.30 -0.17 -0.01 -0.04 -0.25  orf19.4544>
Ca21Chr1_0375890  0.11  0.00 -0.05  0.01  0.10 -0.05 
Ca21Chr1_0375949  0.00  0.00  2.31 -0.09  0.04  0.07 
Ca21Chr1_0376008 -0.02  0.15  0.12 -0.44  0.19 -0.26 
Ca21Chr1_0376067 -0.14 -0.13 -0.03 -0.25 -0.22  0.14 
Ca21Chr1_0376126  0.01  0.00  0.29 -0.01 -0.02  0.50 
Ca21Chr1_0376185  0.19 -0.03  0.25  0.20 -0.24  0.18 
Ca21Chr1_0376244 -0.13  0.19  0.15  0.07 -0.56  0.64 
Ca21Chr1_0376303 -0.11  0.43  0.06  0.33  0.07  0.32 
Ca21Chr1_0376362 -0.03 -0.01 -0.11  0.02  0.01  0.46 
Ca21Chr1_0376421  0.27  0.30  0.00  0.24  0.09  0.17 
Ca21Chr1_0376480  0.53  0.23 -0.03  0.22 -0.34 -0.09 
Ca21Chr1_0376539  0.88  0.50  0.05  0.19  0.26  0.44  orf19.4544|
Ca21Chr1_0376598  0.48  0.25  0.17 -0.28  0.33 -0.35 
Ca21Chr1_0376657  0.18  0.16  0.26 -0.07 -0.16 -0.34  |orf19.4543
Ca21Chr1_0376716 -0.25  0.12  0.31  0.78 -0.57 -0.18 
Ca21Chr1_0376775  0.44  0.27  0.52  0.18  0.01 -0.34 
Ca21Chr1_0376834  0.24  0.24  0.13  0.10  0.22 -0.59 
Ca21Chr1_0376893  0.29  0.33  0.46  0.09  0.27 -0.19 
Ca21Chr1_0376952  0.15 -0.02  0.04  0.01  0.30  0.02 
Ca21Chr1_0377011  0.08 -0.03  0.48  0.03  0.12  0.03 
Ca21Chr1_0377070  0.06  0.46  0.24  0.06  0.28 -0.20 
Ca21Chr1_0377129 -0.10  0.23  0.41  0.15  0.18  0.44 
Ca21Chr1_0377188 -0.09  0.05  0.16  0.01  0.10 -0.29 
Ca21Chr1_0377247  0.15  0.24 -0.16 -0.14 -0.33  0.10 
Ca21Chr1_0377306 -0.34 -0.13 -0.34  0.01 -0.15 -0.03 
Ca21Chr1_0377365 -0.62 -0.13 -0.38  0.19 -0.16  0.08 
Ca21Chr1_0377424 -0.75 -0.39 -0.34  0.08  0.38  0.07 
Ca21Chr1_0377483 -0.13 -0.06  0.25 -0.03  0.20 -0.52 
Ca21Chr1_0377542  0.19 -0.11  0.43  0.22  0.23 -0.02 
Ca21Chr1_0377601  0.00  0.13  0.29 -0.13  0.02  0.01 
Ca21Chr1_0377660  0.20  0.14  0.44  0.70 -0.34  0.07 
Ca21Chr1_0377719  0.07  0.09  0.26  0.35  0.05 -0.10 
Ca21Chr1_0377778  0.16  0.17  1.35  0.50 -0.19  0.58 
Ca21Chr1_0377837  0.52  0.11  0.35  0.54  0.22 -0.05 
Ca21Chr1_0377896  0.17  0.20  0.31  0.20  0.25  0.19 
Ca21Chr1_0377955  0.13  0.04  0.18  0.17 -0.05 -0.13 
Ca21Chr1_0378014  0.31  0.31  0.22 -0.14 -0.17  0.07 
Ca21Chr1_0378073  0.15  0.39  0.10  0.29 -0.50  0.11 
Ca21Chr1_0378132  1.34  0.45  0.23  0.46  0.25  0.18  <orf19.4543
Ca21Chr1_0378191  0.04  0.35  0.27 -0.02  0.17 -0.34 
Ca21Chr1_0378250  0.02  0.13 -0.07  0.41 -0.12 -0.14 
Ca21Chr1_0378309  0.13  0.05 -0.24  0.10  0.31  0.32 
Ca21Chr1_0378368 -0.42  0.10 -0.22  0.18  0.07  0.52 
Ca21Chr1_0378427 -0.32  0.20 -0.28 -0.23 -0.56  0.34  |orf19.4542
Ca21Chr1_0378486 -0.21 -0.23 -0.29  0.06  0.02  0.26 
Ca21Chr1_0378545  0.13 -0.14  0.05 -0.02  0.03  0.06 
Ca21Chr1_0378604  0.56  0.21  0.18 -0.16 -0.03  0.22 
Ca21Chr1_0378663 -0.23  0.06  0.03  0.04 -0.02 -0.07 
Ca21Chr1_0378722  0.20  0.20  0.15  0.12 -0.14 -0.10 
Ca21Chr1_0378781  0.30  0.07  0.00  0.06  0.05 -0.12 
Ca21Chr1_0378840  0.13  0.11 -0.20  0.09 -0.06 -0.32 
Ca21Chr1_0378899  0.38  0.22  0.54  0.36  0.22  0.24 
Ca21Chr1_0378958  0.29  0.19 -0.07 -0.48 -0.16 -0.06 
Ca21Chr1_0379017 -0.00  0.29  0.17  0.21 -0.26  0.14 
Ca21Chr1_0379076  0.12  0.58  0.35  0.13  0.13  0.18 
Ca21Chr1_0379135  0.54  0.16 -0.17  0.18 -0.26  0.10 
Ca21Chr1_0379194  0.62  0.22  0.25 -0.05 -0.25 -0.64 
Ca21Chr1_0379253  0.13  0.47  0.01 -0.07 -0.10 -0.04 
Ca21Chr1_0379312  0.16  0.05  0.46  0.10 -0.12 -0.10 
Ca21Chr1_0379371 -0.14  0.28 -0.09  0.03  0.49  0.06 
Ca21Chr1_0379430  0.47  0.36  0.24 -0.13 -0.03 -0.11 
Ca21Chr1_0379489  0.38  0.34  0.43  0.39  0.02  0.09 
Ca21Chr1_0379548  0.81  0.31  0.19  0.38  0.59 -0.14 
Ca21Chr1_0379607  0.47  0.43  0.39  0.30  0.30  0.03 
Ca21Chr1_0379666 -0.08 -0.08 -0.05 -0.12 -0.52 -0.50  <orf19.4542
Ca21Chr1_0379725 -0.02  0.26  0.02  0.30 -0.25 -0.48 
Ca21Chr1_0379784  0.18  0.07  0.74  0.20 -0.40 -0.30 
Ca21Chr1_0379843  0.40  0.30 -0.04  0.25 -0.04  0.19 
Ca21Chr1_0379902  1.49 -0.09 -0.46  0.27  0.02  0.24 
Ca21Chr1_0379961  1.51  0.19 -0.16 -0.36  0.13 -0.54 
Ca21Chr1_0380020  0.13 -0.27  0.11 -0.40  0.16 -0.29 
Ca21Chr1_0380079  0.02  0.10  0.20  0.32 -0.16  0.34 
Ca21Chr1_0380138  0.42  0.13  0.29  0.30 -0.06  0.53 
Ca21Chr1_0380197  0.30  0.49  0.45  0.32  0.18 -0.04  |orf19.4540
Ca21Chr1_0380256  0.72  0.08  0.98  0.22 -0.04  0.12 
Ca21Chr1_0380315 -0.75 -0.08  0.34  0.39  0.02 -0.18 
Ca21Chr1_0380374  0.18 -0.07  0.14  0.05  0.32 -0.07 
Ca21Chr1_0380433  0.01 -0.11  0.22  0.12  0.05 -0.00 
Ca21Chr1_0380492 -0.22 -0.20 -0.33  0.27 -0.18 -0.19 
Ca21Chr1_0380551  0.26 -0.04 -0.23  0.72  0.10  0.28 
Ca21Chr1_0380610 -0.15 -0.39 -0.45 -0.04  0.67 -0.00 
Ca21Chr1_0380669  0.08 -0.22 -0.76  0.33 -0.09 -0.24 
Ca21Chr1_0380728 -0.02 -0.40 -0.85  0.32 -0.09 -0.09 
Ca21Chr1_0380787  0.39 -0.50 -0.62  0.38  0.11  0.09  <orf19.4540
Ca21Chr1_0380846 -0.00 -0.73 -0.32  0.08 -0.05  0.28 
Ca21Chr1_0380905  0.26  0.25 -0.52 -0.04 -0.18 -0.07 
Ca21Chr1_0380964  0.10 -0.22 -0.96  0.05 -0.15  0.34 
Ca21Chr1_0381023  0.49  0.05 -1.63 -0.14 -0.14  1.22 
Ca21Chr1_0381082 -0.09 -0.06 -1.62  0.42 -0.52  0.12 
Ca21Chr1_0381141 -2.10 -1.04 -2.37 -0.40 -0.58 -0.06 
Ca21Chr1_0381200 -1.21 -0.92 -2.19 -0.41 -0.12  0.10 
Ca21Chr1_0381259 -1.58 -1.88 -1.72 -0.20 -0.25  0.42 
Ca21Chr1_0381318 -1.76 -1.93 -1.72 -0.05 -0.40  0.37 
Ca21Chr1_0381377 -0.86 -1.97 -1.81 -0.53 -0.22  0.27 
Ca21Chr1_0381436 -0.61 -1.06 -1.16 -0.11 -0.29  0.39 
Ca21Chr1_0381495  0.00 -0.51 -0.44  0.12 -0.10  0.30  |orf19.4539
Ca21Chr1_0381554  0.21 -0.11  0.01 -0.24 -0.09  0.38 
Ca21Chr1_0381613  0.21 -0.20  0.05  0.07  0.27  0.30 
Ca21Chr1_0381672  0.17  0.09  0.15 -0.37  0.06  0.15 
Ca21Chr1_0381731  0.09 -0.10  0.28 -0.14  0.03  0.18 
Ca21Chr1_0381790  0.13 -0.02  0.21  0.06 -0.19  0.07 
Ca21Chr1_0381849  0.06 -0.31  0.07  0.07 -0.02  0.02 
Ca21Chr1_0381908  0.05  0.13  0.28  0.07 -0.01 -0.07 
Ca21Chr1_0381967  0.57  0.08  0.87 -0.16 -0.04 -0.13 
Ca21Chr1_0382026  0.34 -0.31 -0.24 -0.23  0.03 -0.14 
Ca21Chr1_0382085  0.27  0.02 -0.16  0.05 -0.13 -0.15  <orf19.4539
Ca21Chr1_0382144 -0.03 -0.10 -0.44 -0.29  0.38  0.10 
Ca21Chr1_0382203  0.01 -0.21 -0.70 -0.02  0.61  0.47 
Ca21Chr1_0382262 -0.26  0.00 -0.89  0.16  0.41 -0.18 
Ca21Chr1_0382321 -0.71 -0.50 -1.18  0.28  0.14  0.62 
Ca21Chr1_0382380 -1.23 -0.94 -1.51  0.15  0.31  0.53 
Ca21Chr1_0382439 -0.50 -1.04 -1.47  0.07 -0.30  0.09 
Ca21Chr1_0382498 -0.27 -0.65 -0.62  0.12  0.06 -0.07 
Ca21Chr1_0382557 -0.19 -0.44 -0.83  0.21 -0.07 -0.03 
Ca21Chr1_0382616 -0.49 -0.12 -0.65 -0.01 -0.52  0.29 
Ca21Chr1_0382675 -0.45 -0.24 -0.40  0.44  0.32  0.23 
Ca21Chr1_0382734 -0.13 -0.14 -0.63 -0.61 -0.25  0.50  |orf19.4538
Ca21Chr1_0382793 -0.18  0.08 -0.10  0.16 -0.11 -0.14 
Ca21Chr1_0382852 -0.62 -0.20 -0.58 -0.02 -0.69 -0.64 
Ca21Chr1_0382911 -0.02 -0.31 -0.18  0.22 -0.04  0.64 
Ca21Chr1_0382970 -0.21 -0.25 -0.57  0.19 -0.05  0.06 
Ca21Chr1_0383029 -0.33  0.06 -0.15 -0.02  0.23  0.76 
Ca21Chr1_0383088 -0.03 -0.06 -0.18 -0.05 -0.38  0.01 
Ca21Chr1_0383147 -0.01 -0.09 -0.24  0.05 -0.00  0.36 
Ca21Chr1_0383206  0.25 -0.01 -0.01 -0.30  0.11  0.05 
Ca21Chr1_0383265 -0.01 -0.14 -0.01  0.05 -0.17  0.11 
Ca21Chr1_0383324  0.42  0.07 -0.12 -0.62 -0.19 -0.25 
Ca21Chr1_0383383  0.94  0.33  0.09 -0.06 -0.13 -0.05 
Ca21Chr1_0383442  0.31  0.06  0.03 -0.04  0.03 -0.19 
Ca21Chr1_0383501  0.01 -0.17 -0.19  0.01 -0.06 -0.17 
Ca21Chr1_0383560 -0.42  0.27  0.32 -0.13 -0.15  0.33  <orf19.4538
Ca21Chr1_0383619  0.39 -0.22 -0.03 -0.22  0.26 -0.24 
Ca21Chr1_0383678  0.19  0.37  0.18  0.11  0.05  0.05 
Ca21Chr1_0383737  0.18  0.04  0.07 -0.18 -0.36 -0.50  orf19.4537>
Ca21Chr1_0383796  0.30  0.15  0.19  0.02 -0.27 -0.09 
Ca21Chr1_0383855  0.08  0.14  0.09 -0.04  0.31 -0.14 
Ca21Chr1_0383914  0.12 -0.09 -0.01 -0.32  0.08 -0.05 
Ca21Chr1_0383973  0.04 -0.12 -0.04 -0.07 -0.04  0.09 
Ca21Chr1_0384032  0.68  0.14 -0.08 -0.01  0.10 -0.06 
Ca21Chr1_0384091  0.15 -0.21  0.02  0.16 -0.03 -0.01 
Ca21Chr1_0384150 -0.38 -0.07  0.04 -0.20 -0.06 -0.01 
Ca21Chr1_0384209 -0.14  0.12 -0.28 -0.18  0.36 -0.03 
Ca21Chr1_0384268  0.09 -0.35 -0.30  0.01  0.05  0.09 
Ca21Chr1_0384327  0.54 -0.34 -0.11 -0.07  0.44 -0.26 
Ca21Chr1_0384386  0.17 -0.37 -0.16  0.34  0.26 -0.27 
Ca21Chr1_0384445 -0.00 -0.28 -0.45  0.20  0.07 -0.25 
Ca21Chr1_0384504 -0.16  0.13 -0.27  0.02 -0.02  0.00 
Ca21Chr1_0384563  0.10  0.33  0.09  0.12  0.36  0.06 
Ca21Chr1_0384622  0.17 -0.00 -0.57  0.04  0.06  0.42 
Ca21Chr1_0384681  0.45  0.01 -0.06 -0.09  0.07  0.56  orf19.4537|
Ca21Chr1_0384740  0.58  0.00  0.21  0.02  0.13  0.22 
Ca21Chr1_0384799  0.78  0.32  0.08  0.18  0.09 -0.78  |orf19.4536
Ca21Chr1_0384858  0.04  0.11  0.41 -0.22  0.03  0.05 
Ca21Chr1_0384917  0.40  0.10  0.34  0.26  0.12 -0.08 
Ca21Chr1_0384976  0.30  0.08  0.20 -0.06  0.13  0.29 
Ca21Chr1_0385035  0.23  0.17  0.20  0.25 -0.05  0.02 
Ca21Chr1_0385094  0.35  0.04  0.11  0.16  0.01 -0.05 
Ca21Chr1_0385153  0.62  0.15  0.33  0.24 -0.20 -0.12 
Ca21Chr1_0385212  1.05 -0.05  0.32 -0.06  0.28 -0.18 
Ca21Chr1_0385271  1.71  0.36  0.41 -0.27  0.28  0.61 
Ca21Chr1_0385330  0.10 -0.04  0.37  0.77 -0.33 -0.07 
Ca21Chr1_0385389  0.47  0.14  0.22  0.02  0.22  0.04 
Ca21Chr1_0385448  0.36  0.06  0.32  0.15  0.38  0.77 
Ca21Chr1_0385507  0.43  0.19  0.13 -0.14  0.23 -0.03 
Ca21Chr1_0385566  0.33  0.06  0.06  0.09  0.02  0.76 
Ca21Chr1_0385625  0.82  0.01  0.37  0.18  0.59 -0.05 
Ca21Chr1_0385684  0.36  0.25  0.50  0.42  0.21  0.27 
Ca21Chr1_0385743  0.83  0.17  0.20  0.19 -0.14 -0.75 
Ca21Chr1_0385802  0.77  0.22  0.46  0.06  0.16 -0.12 
Ca21Chr1_0385861  0.56  0.12  0.26  0.25 -0.17  0.08 
Ca21Chr1_0385920 -0.33  0.07  0.29 -0.03 -0.45  0.54 
Ca21Chr1_0385979  0.27  0.10  0.21  0.19  0.07 -0.16 
Ca21Chr1_0386038  0.10 -0.01  0.27  0.45 -0.32 -0.15 
Ca21Chr1_0386097  0.48  0.26  0.10 -0.09  0.06 -0.05 
Ca21Chr1_0386156  0.44  0.02 -0.03  0.04  0.04 -0.22 
Ca21Chr1_0386215  0.37  0.10  0.10  0.14  0.20 -0.02 
Ca21Chr1_0386274  0.67  0.09 -0.37  0.42 -0.02 -0.04  <orf19.4536
Ca21Chr1_0386333 -0.05 -0.40 -0.29  0.19  0.09 -0.27 
Ca21Chr1_0386392 -0.20  0.01 -1.04 -0.07 -0.20 -0.09 
Ca21Chr1_0386451 -0.47  0.01 -0.92  0.19 -0.25 -0.26 
Ca21Chr1_0386510  0.09 -0.59 -0.83 -0.08 -0.15  0.12 
Ca21Chr1_0386569  0.04 -0.22 -0.52 -0.03 -0.05  0.06  |orf19.4535
Ca21Chr1_0386628 -0.21 -0.30 -0.54  0.07  0.13  0.08 
Ca21Chr1_0386687  0.04 -0.01  0.20  0.15  0.01 -0.36 
Ca21Chr1_0386746  0.08  0.04  0.11  0.42 -0.07 -0.06 
Ca21Chr1_0386805  0.05 -0.04 -0.17 -0.15 -0.04 -0.08 
Ca21Chr1_0386864  0.10 -0.04  0.06 -0.08  0.85 -0.25 
Ca21Chr1_0386923  0.80 -0.02  0.17  0.12 -0.43 -0.39 
Ca21Chr1_0386982  0.16  0.05  0.11 -0.09  0.08 -0.28 
Ca21Chr1_0387041  0.31 -0.08  0.12  0.12  0.17  0.18 
Ca21Chr1_0387100  0.20  0.26  0.36  0.36  0.28 -0.00 
Ca21Chr1_0387159  0.21 -0.06  0.32 -0.31 -0.17 -0.12 
Ca21Chr1_0387218  0.78  0.26  0.29  0.25  0.04 -0.00 
Ca21Chr1_0387277  0.28  0.25  0.52  0.10 -0.02 -0.09 
Ca21Chr1_0387336 -0.04  0.43  0.04 -0.10 -0.07 -0.36 
Ca21Chr1_0387395 -0.16  0.54  0.19 -0.20 -0.12 -0.22 
Ca21Chr1_0387454 -0.00  0.00  0.44  0.29 -0.04 -0.41 
Ca21Chr1_0387513 -0.47  0.19  0.47  0.05  0.03 -0.26 
Ca21Chr1_0387572 -0.08  0.14  1.15  0.10  0.01  0.00 
Ca21Chr1_0387631  0.02  0.19  0.42 -0.34  0.22 -0.14 
Ca21Chr1_0387690 -0.12  0.07  0.66 -0.29  0.27 -0.06 
Ca21Chr1_0387749  0.11 -0.01  0.25 -0.14 -0.05 -0.10 
Ca21Chr1_0387808  0.32  0.23  0.53  0.19 -0.06  0.16 
Ca21Chr1_0387867  0.33  0.07  0.15  0.03  0.77 -0.25 
Ca21Chr1_0387926  0.28  0.25  0.13  0.15  0.20 -0.12 
Ca21Chr1_0387985  0.32 -0.01  0.17  0.12 -0.01  0.04 
Ca21Chr1_0388044  0.23  0.10  0.33 -0.16 -0.18 -0.09 
Ca21Chr1_0388103  0.21  0.33 -0.07 -0.04  0.32  0.42 
Ca21Chr1_0388162 -0.84  0.17 -0.00 -0.12 -0.25 -0.36 
Ca21Chr1_0388221  0.25  0.38  0.72 -0.09 -0.02 -0.76 
Ca21Chr1_0388280 -0.01  0.25  0.38  0.56 -0.31  0.27 
Ca21Chr1_0388339 -0.12  0.61  0.28  0.16  0.32  0.15 
Ca21Chr1_0388398 -0.19  0.37  0.06 -0.25  0.27  0.07 
Ca21Chr1_0388457  0.41  0.28  0.43 -0.06  0.04  0.91 
Ca21Chr1_0388516  0.37  0.28  0.14 -0.13  0.30  0.08 
Ca21Chr1_0388575  0.16  0.04  0.17  0.09 -0.06  0.24 
Ca21Chr1_0388634  0.03 -0.01 -0.02  0.12 -0.22  0.24 
Ca21Chr1_0388693  0.57  0.23 -0.26 -0.41  0.33  0.08 
Ca21Chr1_0388752  0.15 -0.02 -0.24  0.18 -0.07  0.05 
Ca21Chr1_0388811 -0.21 -0.04 -0.25  0.45 -0.31  0.10 
Ca21Chr1_0388870  0.33 -0.26 -0.47  0.36  0.06  0.24 
Ca21Chr1_0388929 -0.11  0.12 -0.44  0.03 -0.03 -0.11  <orf19.4535
Ca21Chr1_0388988  0.08 -0.06 -0.95  0.06  0.25  0.30 
Ca21Chr1_0389047  0.43 -0.55  0.07 -0.15  0.04  0.11 
Ca21Chr1_0389106 -0.00  0.03  0.39  0.32 -0.25  0.57 
Ca21Chr1_0389165 -0.02 -0.03  0.14  0.10 -0.10  0.37 
Ca21Chr1_0389224  0.96  0.00  0.62 -0.09 -0.17  0.38 
Ca21Chr1_0389283 -0.48  0.38  0.30  0.04  0.70 -0.19  |orf19.4534
Ca21Chr1_0389342  0.08 -0.01  0.31 -0.14 -0.01  0.06 
Ca21Chr1_0389401 -0.01  0.24  0.41 -0.36  0.04 -0.07 
Ca21Chr1_0389460  0.24  0.12  0.39 -0.09  0.05 -0.28 
Ca21Chr1_0389519 -0.43  0.43  0.44 -0.29  0.08  0.12 
Ca21Chr1_0389578  0.03  0.44  0.33  0.26  0.02 -0.30 
Ca21Chr1_0389637 -0.24  0.18  0.22 -0.04  0.01 -0.54 
Ca21Chr1_0389696  0.21  0.20  0.29  0.13 -0.05 -0.38 
Ca21Chr1_0389755 -0.76  0.05  0.31 -0.29 -0.02  0.04 
Ca21Chr1_0389814  0.30  0.03  0.23  0.08  0.23 -0.22 
Ca21Chr1_0389873  0.01  0.14  0.09  0.00  0.22 -0.52 
Ca21Chr1_0389932  0.17  0.37 -0.13 -0.10  0.11 -0.31 
Ca21Chr1_0389991 -0.01  0.03  0.43 -0.36  0.34 -0.43 
Ca21Chr1_0390050 -0.28  0.19  0.54  0.03  0.10 -0.38 
Ca21Chr1_0390109  0.04  0.31  0.42 -0.22 -0.34 -0.35 
Ca21Chr1_0390168 -0.03  0.18  0.23  0.06  0.24  0.60 
Ca21Chr1_0390227  0.42  0.24  0.19 -0.36  0.03  0.06 
Ca21Chr1_0390286  0.08  0.05  0.10 -0.27  0.15 -0.03 
Ca21Chr1_0390345  0.15 -0.03  0.26  0.00 -0.04  0.27 
Ca21Chr1_0390404 -0.01 -0.20  0.01 -0.24  0.33  0.23 
Ca21Chr1_0390463  0.17  0.12 -0.01 -0.01  0.13  0.31 
Ca21Chr1_0390522 -0.02 -0.11 -0.18 -0.11  0.46  0.08 
Ca21Chr1_0390581  0.12 -0.04  0.19  0.21 -0.04  0.06 
Ca21Chr1_0390640  0.08  0.19 -0.05 -0.45  0.08  0.64 
Ca21Chr1_0390699  0.02  0.05 -0.01 -0.00 -0.01  0.26 
Ca21Chr1_0390758  0.27 -0.02 -0.16 -0.02 -0.26  0.45  <orf19.4534
Ca21Chr1_0390817 -0.04 -0.03 -0.58 -0.04 -0.07 -0.26 
Ca21Chr1_0390876 -0.20 -0.14 -1.05 -0.04  0.20 -0.23 
Ca21Chr1_0390935 -0.63 -0.50 -1.04 -0.15  0.08 -0.25 
Ca21Chr1_0390994  0.04 -0.98 -0.82 -0.40  0.28 -0.08 
Ca21Chr1_0391053  0.07 -0.12 -0.36 -0.36  0.23  0.14 
Ca21Chr1_0391112 -0.24 -0.32 -0.25 -0.01  0.13  0.00 
Ca21Chr1_0391171  0.01  0.08 -0.36 -0.20 -0.69 -0.00 
Ca21Chr1_0391230  0.33 -0.14 -0.25 -0.02 -0.18  1.28  |orf19.4533
Ca21Chr1_0391289 -0.11  0.05 -0.23 -0.21  0.04 -0.07 
Ca21Chr1_0391348 -0.26 -0.13 -0.53  0.12  0.22  0.44 
Ca21Chr1_0391407 -0.07  0.27 -0.03  0.21 -0.01 -0.09 
Ca21Chr1_0391466  0.18  0.25 -0.17  0.09 -0.01  0.38 
Ca21Chr1_0391525 -0.01  0.16 -0.30  0.11  0.18 -0.07 
Ca21Chr1_0391584  0.14  0.02 -0.26 -0.03  0.11 -0.08 
Ca21Chr1_0391643  0.86  0.10 -0.02  0.15  0.07 -0.17 
Ca21Chr1_0391702  0.11 -0.18  0.12  0.15  0.02 -0.03 
Ca21Chr1_0391761  0.18  0.00  0.30 -0.10  0.37  0.12 
Ca21Chr1_0391820  0.47  0.20  0.56 -0.03  0.20  0.38 
Ca21Chr1_0391879  0.44  0.18  0.45  0.09  0.00 -0.19 
Ca21Chr1_0391938 -0.05  0.15 -0.01 -0.42 -0.15  0.32 
Ca21Chr1_0391997 -0.05 -0.00 -0.18 -0.08 -0.37 -0.13 
Ca21Chr1_0392056  0.02 -0.09 -0.38  0.05  0.29 -0.71 
Ca21Chr1_0392115 -0.24 -0.14  0.18 -0.01  0.08  0.40 
Ca21Chr1_0392174 -0.01 -0.24 -0.35 -0.31 -0.12  0.05 
Ca21Chr1_0392233 -0.10 -0.27 -0.19  0.02  0.01 -0.21 
Ca21Chr1_0392292 -0.54 -0.28 -0.32  0.06  0.18  0.21 
Ca21Chr1_0392351  0.10 -0.13  0.10 -0.08  0.27 -0.02 
Ca21Chr1_0392410 -0.71  0.01 -0.03 -0.25  0.12  0.60 
Ca21Chr1_0392469  0.29 -0.24  0.31 -0.25  0.25 -0.22 
Ca21Chr1_0392528 -0.03 -0.16 -0.24  0.03 -0.03  0.22 
Ca21Chr1_0392587 -0.03 -0.05  0.10  0.08  0.02  0.11 
Ca21Chr1_0392646  0.25  0.01  0.01 -0.14  0.16  0.10 
Ca21Chr1_0392705 -0.26  0.00 -0.17  0.20 -0.07 -0.02 
Ca21Chr1_0392764 -0.54  0.07 -0.05  0.17  0.17 -0.27  <orf19.4533
Ca21Chr1_0392823 -0.50  0.04 -0.18 -0.09 -0.08 -0.07 
Ca21Chr1_0392882 -0.78  0.13 -0.46 -0.58  0.09  0.00 
Ca21Chr1_0392941 -0.52  0.01 -0.73 -0.26 -0.14  0.06 
Ca21Chr1_0393000 -1.33  0.01 -0.50  0.24 -0.32  0.28 
Ca21Chr1_0393059 -0.78 -0.18 -0.42 -0.15 -0.13  0.29 
Ca21Chr1_0393118 -0.69 -0.23  0.11  0.06 -0.25  0.14 
Ca21Chr1_0393177 -0.30  0.11  0.07 -0.17 -0.08  0.33 
Ca21Chr1_0393236 -0.04  0.28  0.23  0.16 -0.09  0.40 
Ca21Chr1_0393295  0.29  0.10 -0.43 -0.04  0.90  0.27  orf19.4532>
Ca21Chr1_0393354 -0.38 -0.01  0.08 -0.09  0.07 -0.17 
Ca21Chr1_0393413  0.21  0.34  1.12 -0.35 -0.21 -0.05 
Ca21Chr1_0393472  0.25  0.04 -0.05  0.05 -0.23  0.13 
Ca21Chr1_0393531  0.03 -0.20  0.04  0.21 -0.04  0.19 
Ca21Chr1_0393590  0.07 -0.06 -0.07  0.30 -0.13  0.16 
Ca21Chr1_0393649  0.06 -0.04 -0.07 -0.26  0.06  0.17 
Ca21Chr1_0393708 -0.30  0.06  0.84  0.19  0.28 -0.30 
Ca21Chr1_0393767 -0.20  0.01 -0.04 -0.35  0.49 -0.10 
Ca21Chr1_0393826 -0.18  0.14  0.25  0.01  0.09 -0.10 
Ca21Chr1_0393885 -0.23 -0.05 -0.00 -0.44  0.12 -0.11 
Ca21Chr1_0393944 -0.21 -0.17 -0.10 -0.36 -0.20 -0.39 
Ca21Chr1_0394003 -0.58 -0.07 -0.53  0.00 -0.61 -0.47 
Ca21Chr1_0394062 -0.93  0.22 -0.68  0.11 -0.46 -0.40 
Ca21Chr1_0394121 -0.92 -0.17 -0.73 -0.09  0.01  0.00 
Ca21Chr1_0394180 -1.24 -0.45 -1.05  0.04 -0.84  0.49 
Ca21Chr1_0394239 -0.18 -0.31 -1.53  0.50 -0.29  0.04 
Ca21Chr1_0394298 -2.04 -0.46 -1.91 -0.39 -0.11  0.01 
Ca21Chr1_0394357 -2.01 -1.15 -2.33  0.09 -0.15  0.09  orf19.4532|
Ca21Chr1_0394416 -2.42 -1.06 -2.41 -0.41 -0.75  0.17 
Ca21Chr1_0394475 -2.86 -1.12 -1.99 -0.56 -0.05 -0.04 
Ca21Chr1_0394534 -2.33 -0.80 -1.87 -0.94 -0.16  0.19 
Ca21Chr1_0394593 -1.78 -1.13 -1.23 -0.38  0.02  0.20 
Ca21Chr1_0394652 -0.93 -0.44 -1.33 -0.21 -0.20 -0.45 
Ca21Chr1_0394711 -0.51 -0.16 -0.67 -0.11 -0.03  0.03 
Ca21Chr1_0394770 -0.68  0.20 -0.35 -0.86 -0.36  0.29 
Ca21Chr1_0394829 -1.26 -0.00 -0.19 -0.54  0.54  0.05 
Ca21Chr1_0394888 -0.02  0.10 -0.12 -0.58 -0.14  0.39 
Ca21Chr1_0394947 -0.03  0.14 -0.18 -0.25 -0.25 -0.05 
Ca21Chr1_0395006 -0.51 -0.12 -0.01 -0.08 -0.16 -0.03 
Ca21Chr1_0395065 -0.24  0.07  0.03 -0.09 -0.13 -0.06 
Ca21Chr1_0395124 -0.51 -0.03 -0.64 -0.22  0.14 -0.15 
Ca21Chr1_0395183 -0.42 -0.18 -0.04  0.06  0.31  0.06 
Ca21Chr1_0395242 -0.54  0.04 -0.37 -0.10  0.06  0.69 
Ca21Chr1_0395301 -0.58 -0.01 -0.16 -0.41 -0.01  0.52 
Ca21Chr1_0395360 -0.26 -0.27 -0.44  0.03 -0.07  1.71 
Ca21Chr1_0395419  0.07 -0.29  0.18 -0.13 -0.04  0.44 
Ca21Chr1_0395478  0.63  0.23  0.11  0.13 -0.23  0.51 
Ca21Chr1_0395537 -0.25  0.10  0.18  0.23  0.16  0.08 
Ca21Chr1_0395596 -0.12 -0.19  0.22  0.17 -0.11  0.33  orf19.4531>
Ca21Chr1_0395655 -0.01 -0.07  0.72  0.05  0.07 -0.70 
Ca21Chr1_0395714 -0.05  0.26  0.20 -0.07  0.13 -0.32 
Ca21Chr1_0395773  0.13 -0.68  0.17 -0.18  0.27 -0.23 
Ca21Chr1_0395832 -0.19 -0.04  0.04 -0.20 -0.18 -0.15 
Ca21Chr1_0395891  0.54  0.11  0.27  0.28  0.12 -0.16 
Ca21Chr1_0395950  0.26 -0.00  0.13  0.03  0.28 -0.18 
Ca21Chr1_0396009  0.51 -0.06 -0.08 -0.10  0.30  0.14 
Ca21Chr1_0396068  0.24 -0.21  0.18 -0.18 -0.04  0.19 
Ca21Chr1_0396127  0.21  0.23  0.42  0.20 -0.10 -0.04 
Ca21Chr1_0396186  0.31 -0.07  0.39 -0.17  0.42  0.13 
Ca21Chr1_0396245  0.25 -0.05  0.15 -0.41 -0.08  0.51 
Ca21Chr1_0396304  0.24  0.46  0.05  0.02  0.45  0.15 
Ca21Chr1_0396363 -0.06  0.42  0.38  0.14  0.34 -0.11 
Ca21Chr1_0396422  0.10  0.04  0.19  0.17  0.41  0.12 
Ca21Chr1_0396481  3.77 -0.13  0.22  0.04  0.25 -0.20 
Ca21Chr1_0396540 -0.08  0.14  0.45  0.51  0.20  0.09 
Ca21Chr1_0396599  0.17 -0.23  0.38  0.49  0.62  0.09 
Ca21Chr1_0396658 -0.33  0.33  0.44  0.17 -0.00 -0.04 
Ca21Chr1_0396717  0.12  0.20  0.31  0.21  0.17 -0.31 
Ca21Chr1_0396776  0.12 -0.03  0.63 -0.05  0.05 -0.16 
Ca21Chr1_0396835  0.12  0.03  0.56  0.08  0.03  0.11 
Ca21Chr1_0396894  0.23  0.18  0.34 -0.43  0.08  0.01 
Ca21Chr1_0396953  0.87  0.18  1.29  0.13  0.01  0.61 
Ca21Chr1_0397012 -0.23  0.15  0.27 -0.24  0.25  0.01 
Ca21Chr1_0397071  0.32 -0.14  0.18 -0.29  0.63  0.01 
Ca21Chr1_0397130  0.17  0.34  0.11  0.23 -0.21  0.44 
Ca21Chr1_0397189  0.00  0.29  0.59 -0.07 -0.68 -0.06 
Ca21Chr1_0397248  0.04 -0.19  0.31 -0.07  0.02  0.06 
Ca21Chr1_0397307  0.12 -0.15  0.33  0.00  0.13 -0.19 
Ca21Chr1_0397366  0.21  0.07  0.41 -0.22 -0.03 -0.15 
Ca21Chr1_0397425  0.44  0.14  0.56  0.03  0.24  0.04 
Ca21Chr1_0397484  0.44 -0.21  0.58 -0.23  0.33 -0.08 
Ca21Chr1_0397543  0.13  0.12  0.36 -0.18 -0.05  0.77 
Ca21Chr1_0397602 -0.40 -0.15  0.59  0.34  0.56  0.05 
Ca21Chr1_0397661  0.13  0.07  0.10 -0.24 -1.04  1.06 
Ca21Chr1_0397720  0.11 -0.27  0.05 -0.24  0.18 -0.17 
Ca21Chr1_0397779  0.05  0.13  0.03 -0.38  0.18 -0.09 
Ca21Chr1_0397838 -0.21  0.01  0.02 -0.11 -0.16 -0.35 
Ca21Chr1_0397897  0.21  0.14  0.00 -0.64 -0.23 -0.32 
Ca21Chr1_0397956  0.29  0.00  0.06 -0.47 -0.64  0.03 
Ca21Chr1_0398015 -0.13 -0.24  0.11 -1.06 -0.53 -0.53 
Ca21Chr1_0398074  0.10  0.65  0.29 -1.38 -0.51 -0.31 
Ca21Chr1_0398133  0.38  0.10  0.87 -0.88  0.13 -0.34 
Ca21Chr1_0398192 -0.49 -0.01  0.03 -1.34 -0.61 -0.88 
Ca21Chr1_0398251  0.32 -0.02  0.25 -0.84 -0.19 -0.71 
Ca21Chr1_0398310  0.22 -0.05  0.13 -0.84 -0.77 -0.10 
Ca21Chr1_0398369 -0.10 -0.05  0.31 -1.09 -0.36 -0.25 
Ca21Chr1_0398428  0.08  0.11  0.29 -0.45  0.09  0.11 
Ca21Chr1_0398487 -0.02  0.11  0.09 -0.37  0.05 -0.23 
Ca21Chr1_0398546  0.10  0.28  0.46 -0.24  0.25  0.02 
Ca21Chr1_0398605  0.34  0.11  0.56 -0.18 -0.14  0.25 
Ca21Chr1_0398664 -0.06  0.10 -0.23 -0.34  0.01 -0.22 
Ca21Chr1_0398723  0.44  0.15  0.38  0.13  0.15 -0.29 
Ca21Chr1_0398782  0.01  0.05  0.17  0.02  0.29  0.02 
Ca21Chr1_0398841 -0.12  0.17 -0.21 -0.32 -0.15  0.14 
Ca21Chr1_0398900 -0.65  0.10  0.55  0.12 -0.82 -0.31 
Ca21Chr1_0398959  0.28  0.03  0.37 -0.63  0.20  0.08 
Ca21Chr1_0399018  0.18  0.38  0.33 -0.15  0.07 -0.02 
Ca21Chr1_0399077  0.24 -0.00  0.30  0.04  0.20  0.11 
Ca21Chr1_0399136  0.21  0.12  0.27 -0.11  0.31 -0.27 
Ca21Chr1_0399195  0.37 -0.05  0.07 -0.12 -0.04  0.00 
Ca21Chr1_0399254  0.09 -0.16  0.28  0.23 -0.09  0.02 
Ca21Chr1_0399313 -0.07  0.09  0.15 -0.07 -0.13  0.30 
Ca21Chr1_0399372  0.10  0.15 -0.01  0.03  0.02  0.18  orf19.4531|
Ca21Chr1_0399431  0.08 -0.21 -0.18  0.15 -0.21  0.04 
Ca21Chr1_0399490  0.16  0.10 -0.36  0.14  0.37 -0.35 
Ca21Chr1_0399549 -0.05  0.03 -0.46  0.27  0.11 -0.26 
Ca21Chr1_0399608  0.71  0.19 -0.65  0.26 -0.22 -0.15 
Ca21Chr1_0399667 -0.12 -0.00 -0.73 -0.12  0.09  0.08 
Ca21Chr1_0399726  0.01 -0.05 -0.51  0.38  0.19  0.53 
Ca21Chr1_0399785 -0.22  0.37 -0.35 -0.21  0.54 -0.23 
Ca21Chr1_0399844 -0.07  0.19  1.35  0.08  0.14 -0.54 
Ca21Chr1_0399903  0.01  0.32  0.18  0.12  0.01  0.03 
Ca21Chr1_0399962 -0.55 -0.21 -0.09 -0.13  0.25  0.32 
Ca21Chr1_0400021  0.40 -0.11 -0.15 -0.30 -0.15  0.17 
Ca21Chr1_0400080 -0.07 -0.15 -0.30 -0.54 -0.46 -0.15 
Ca21Chr1_0400139 -0.22  0.10 -0.13  0.03  0.43  0.53 
Ca21Chr1_0400198 -0.42  0.15 -0.32  0.07  0.25  0.32 
Ca21Chr1_0400257 -0.19  0.06 -0.27  0.02 -0.08 -2.07 
Ca21Chr1_0400316 -0.00  0.40 -0.01 -0.16 -0.33  0.03 
Ca21Chr1_0400375  0.22  0.29  0.44  0.02  0.32  0.06 
Ca21Chr1_0400434 -0.15 -0.03 -0.11  0.43 -0.28 -0.01 
Ca21Chr1_0400493    NA    NA    NA    NA    NA    NA
Ca21Chr1_0400552 -0.49 -0.41 -0.21 -0.54 -0.13 -0.86  orf19.4530.1>
Ca21Chr1_0400611 -0.55  0.21  0.33 -0.01  0.06 -0.46 
Ca21Chr1_0400670 -0.58  0.18  0.14 -0.45  0.02  0.06 
Ca21Chr1_0400729  0.24  0.17  0.07 -0.38  0.17 -0.03 
Ca21Chr1_0400788  0.14 -0.12 -0.34  0.13 -0.21  0.57 
Ca21Chr1_0400847 -0.08  0.05  0.20 -0.12 -0.09  0.14 
Ca21Chr1_0400906 -0.01 -0.07  0.19 -0.05 -0.27  0.15 
Ca21Chr1_0400965  0.13 -0.84  0.36 -0.28 -0.01  0.10 
Ca21Chr1_0401024 -0.97  0.19  0.10 -0.01 -0.70  0.22 
Ca21Chr1_0401083 -0.00 -0.03  0.07  0.29 -0.08 -0.32 
Ca21Chr1_0401142  0.42 -0.20  0.30 -0.30  0.25 -0.07 
Ca21Chr1_0401201  0.04  0.15  0.15  0.36  0.09 -0.04 
Ca21Chr1_0401260  0.12  0.11  0.61  0.18  0.11  0.00 
Ca21Chr1_0401319  0.14  0.38 -0.18  0.39 -0.39 -0.30 
Ca21Chr1_0401378  0.20 -0.43  0.75  0.18  0.29 -0.10 
Ca21Chr1_0401437  1.13  0.09  0.20  0.07  0.08 -0.03 
Ca21Chr1_0401496 -0.27  0.22  0.31 -0.11  0.34  0.36 
Ca21Chr1_0401555 -0.01 -0.04  0.28  0.18 -0.19  0.09 
Ca21Chr1_0401614 -0.12 -0.06  0.25 -0.34  0.37  0.02 
Ca21Chr1_0401673  0.28 -0.01  0.45  0.32  0.13  0.12 
Ca21Chr1_0401732  0.40  0.16  0.31  0.11 -0.12  0.34 
Ca21Chr1_0401791 -0.06  0.22  0.08 -0.45 -0.02  0.10 
Ca21Chr1_0401850  0.31  0.04  0.42  0.03 -0.09  0.03 
Ca21Chr1_0401909  0.07  0.42 -0.08 -0.50  0.26 -0.21 
Ca21Chr1_0401968 -0.12  0.19  0.96 -0.24 -0.06 -0.14  orf19.4530.1|
Ca21Chr1_0402027  0.02  0.32 -0.05 -0.19  0.26 -0.10 
Ca21Chr1_0402086 -0.41  0.09  0.07 -0.36 -0.02  0.08 
Ca21Chr1_0402145  0.01  0.35 -0.14 -0.33 -0.00  0.13 
Ca21Chr1_0402204 -0.19  0.35  0.30  0.07 -0.17 -0.27 
Ca21Chr1_0402263  0.24 -0.29  0.16 -0.05 -0.33  0.70  |orf19.4529
Ca21Chr1_0402322  0.71 -0.10 -0.36  0.04  0.45  0.36 
Ca21Chr1_0402381  0.18 -0.14 -0.15  0.11 -0.16  0.82 
Ca21Chr1_0402440  0.02  0.10  0.16 -0.01 -0.16  0.40 
Ca21Chr1_0402499  0.11 -0.11 -0.08 -0.12 -0.18  0.50 
Ca21Chr1_0402558 -0.16  0.07 -0.27 -0.17 -0.13 -0.08 
Ca21Chr1_0402617  0.12 -0.71 -0.04  0.60 -0.27  0.61 
Ca21Chr1_0402676  0.27  0.02 -0.50 -0.11 -0.11 -0.81 
Ca21Chr1_0402735 -0.00 -0.04 -0.21 -0.10 -0.63  0.04  <orf19.4529
Ca21Chr1_0402794  1.26 -0.05  0.05 -0.06 -0.15 -0.03 
Ca21Chr1_0402853  0.10 -0.11 -0.18 -0.22 -0.10  0.01 
Ca21Chr1_0402912  0.72 -0.10 -0.18  0.11  0.09  0.24 
Ca21Chr1_0402971 -0.02  0.25 -0.03  0.36 -0.10 -0.12 
Ca21Chr1_0403030  0.46 -0.04 -0.12 -0.18 -0.14  0.31 
Ca21Chr1_0403089 -0.01  0.00 -0.08 -0.08 -0.11  0.08 
Ca21Chr1_0403148  0.30  0.15  0.17  0.15 -0.07  0.16  |orf19.4528
Ca21Chr1_0403207  0.35  0.31 -0.24  0.01 -0.27  0.24 
Ca21Chr1_0403266 -0.32  0.05 -0.05  0.23 -0.34 -0.23 
Ca21Chr1_0403325  0.10  0.06 -0.65  0.07 -0.09  0.50 
Ca21Chr1_0403384  0.26 -0.19 -0.57  0.02  0.01 -0.07 
Ca21Chr1_0403443 -0.01 -0.08 -0.94 -0.10 -0.32  0.32 
Ca21Chr1_0403502 -0.35 -0.36 -1.22 -0.08 -0.21 -0.12 
Ca21Chr1_0403561  0.25 -0.63 -1.41 -0.51 -0.47  0.15 
Ca21Chr1_0403620 -0.51 -0.92 -2.13 -0.37 -0.35 -0.35 
Ca21Chr1_0403679 -0.82 -0.91 -2.16 -0.04  0.02  0.33  <orf19.4528
Ca21Chr1_0403738 -1.28 -0.96 -2.09 -0.37  0.02 -0.31 
Ca21Chr1_0403797 -1.36 -1.46 -1.49 -0.22  0.49 -0.08 
Ca21Chr1_0403856 -0.65 -0.78 -0.89 -0.43  0.10  0.08 
Ca21Chr1_0403915 -0.33 -0.67 -0.09 -0.05  0.27  0.12 
Ca21Chr1_0403974 -0.04 -0.45 -0.39 -0.39 -0.12  0.20 
Ca21Chr1_0404033 -0.24 -0.38  0.13 -0.22 -0.49  0.25 
Ca21Chr1_0404092 -0.30 -0.11 -0.65 -0.45  0.07 -0.03 
Ca21Chr1_0404151 -0.02 -0.07 -0.07 -0.22  0.09 -0.33 
Ca21Chr1_0404210  0.03  0.28 -0.09  0.14 -0.28  0.55 
Ca21Chr1_0404269  0.00 -0.74 -1.13  0.11  0.24 -0.30 
Ca21Chr1_0404328 -0.02 -0.53 -0.74  0.26  0.05 -0.40 
Ca21Chr1_0404387 -0.29 -0.42 -0.56 -0.06  0.08 -0.30 
Ca21Chr1_0404446 -0.23 -0.23 -0.83 -0.01 -0.02 -0.60 
Ca21Chr1_0404505 -0.21 -0.26 -0.13  0.17  0.27 -0.29 
Ca21Chr1_0404564  0.24 -0.15  0.51  0.12  0.37  0.25 
Ca21Chr1_0404623  0.43  0.01  0.29 -0.13 -0.26  0.10 
Ca21Chr1_0404682  0.04 -0.00  0.18  0.24  0.33 -0.10 
Ca21Chr1_0404741 -0.06  0.67  0.46  0.16  0.60  0.49 
Ca21Chr1_0404800 -0.07  0.57 -0.14 -0.01  0.26  0.08 
Ca21Chr1_0404859 -0.05 -0.05 -0.04  0.02 -0.00 -0.28 
Ca21Chr1_0404918  0.14 -0.17 -0.42 -0.10 -0.09 -0.13 
Ca21Chr1_0404977  0.10 -0.18 -0.67  0.00  0.24  0.31 
Ca21Chr1_0405036 -0.21 -0.04 -0.73 -0.20  0.20  0.67 
Ca21Chr1_0405095 -0.04 -0.70 -1.03  0.09  0.18  0.26 
Ca21Chr1_0405154  0.02 -0.44  1.98 -0.52 -0.35  0.43 
Ca21Chr1_0405213 -0.59 -0.51 -0.82 -0.32  0.09  0.47 
Ca21Chr1_0405272 -0.78 -1.36 -1.92 -0.51 -0.03  0.52 
Ca21Chr1_0405331 -0.57 -0.78 -0.84 -0.54 -0.16  0.34 
Ca21Chr1_0405390  0.27 -0.57 -0.73 -0.51 -0.18  0.35 
Ca21Chr1_0405449 -0.13 -0.34 -0.72 -0.22 -0.17  0.01 
Ca21Chr1_0405508  0.64 -0.66 -0.19 -0.79 -0.16 -0.55 
Ca21Chr1_0405567 -0.20 -0.46 -0.10 -0.46 -0.19  0.11 
Ca21Chr1_0405626 -0.25 -0.40 -0.33 -0.36 -0.20 -0.14 
Ca21Chr1_0405685 -0.36 -0.14 -0.14 -0.48 -0.26 -0.12 
Ca21Chr1_0405744 -0.02 -0.37 -0.40 -0.40 -0.55 -0.32 
Ca21Chr1_0405803 -0.43 -0.25 -0.96 -0.20 -0.48  0.07 
Ca21Chr1_0405862 -0.28 -0.34 -0.85 -0.39 -0.04  0.14 
Ca21Chr1_0405921 -0.38 -0.61 -0.86  0.09 -0.06  0.20 
Ca21Chr1_0405980  0.12 -0.57 -0.87  0.18  0.06  0.43 
Ca21Chr1_0406039 -0.54 -0.65 -0.95  0.34  0.14  0.42 
Ca21Chr1_0406098 -0.50 -0.34 -0.63  0.05  0.15  0.22 
Ca21Chr1_0406157  0.02 -0.44 -0.88  0.02 -0.20  0.21 
Ca21Chr1_0406216  0.69 -0.55 -0.16  0.39 -0.26  0.45 
Ca21Chr1_0406275 -0.08 -0.32 -0.31  0.13 -0.06  0.44 
Ca21Chr1_0406334 -0.30  0.19 -0.49  0.26 -0.02  0.41 
Ca21Chr1_0406393 -0.12 -0.16 -0.36 -0.33 -0.30  0.20 
Ca21Chr1_0406452  0.09  0.18 -0.07  0.01 -0.01 -0.70 
Ca21Chr1_0406511  0.15 -0.21  0.06  0.32  0.08  0.23 
Ca21Chr1_0406570 -0.04  0.01  0.01  0.08 -0.28  0.20 
Ca21Chr1_0406629  0.18 -0.07  0.02 -0.16  0.12 -0.08 
Ca21Chr1_0406688  0.14  0.35  0.43 -0.02  0.02 -0.10  orf19.4527>
Ca21Chr1_0406747  0.41  0.65  0.85  0.26 -0.21  0.01 
Ca21Chr1_0406806  0.34  0.98  0.34 -0.20  0.03 -0.14 
Ca21Chr1_0406865 -0.14  0.37  0.32 -0.03  0.35 -0.45 
Ca21Chr1_0406924  0.20  0.35 -0.08 -0.32  0.14 -0.36 
Ca21Chr1_0406983  0.24  0.68  0.20  0.01 -0.20 -0.39 
Ca21Chr1_0407042  0.47  0.23  0.60  0.12  0.23 -0.20 
Ca21Chr1_0407101  0.68  0.67  0.84  0.21  0.38  0.00 
Ca21Chr1_0407160  0.46  0.89  0.16 -0.10  0.17 -0.12 
Ca21Chr1_0407219  0.45  0.49  0.85  0.02  0.18 -0.01 
Ca21Chr1_0407278  0.52  1.55  0.83 -0.17  0.35 -0.15 
Ca21Chr1_0407337  0.25  0.76  0.30  0.05  0.19 -0.27 
Ca21Chr1_0407396  0.13  0.48  0.16 -0.20  0.19 -0.53 
Ca21Chr1_0407455 -0.07  0.28  0.42 -0.10  0.82 -0.32 
Ca21Chr1_0407514  0.20  0.59  0.64  0.14  0.18 -0.66 
Ca21Chr1_0407573  0.10  0.77  0.56  0.07  0.14 -0.03 
Ca21Chr1_0407632 -0.02  0.97  0.29 -0.08  0.24 -0.26 
Ca21Chr1_0407691  0.39  0.65  0.38 -0.22  0.31 -0.29 
Ca21Chr1_0407750  0.29  1.23  0.35 -0.26 -0.39 -0.16 
Ca21Chr1_0407809  0.02  0.66  0.40  0.03  0.33 -0.47 
Ca21Chr1_0407868  0.40  1.16  1.37 -0.02  0.41  0.06 
Ca21Chr1_0407927  0.96  1.29  1.08 -0.26  0.46 -0.06 
Ca21Chr1_0407986  0.59  0.84  0.87 -0.31  0.83  0.11 
Ca21Chr1_0408045  0.15  0.51  0.26 -0.01  0.07 -0.02 
Ca21Chr1_0408104  0.25  0.72  0.58 -0.02  0.16  0.01 
Ca21Chr1_0408163  0.17  1.11  0.77  0.48  0.90 -0.21 
Ca21Chr1_0408222  0.24  0.95  0.37  0.23  0.26 -0.31 
Ca21Chr1_0408281  0.31  0.62  0.31  0.39  0.39  0.08  orf19.4527|
Ca21Chr1_0408340  0.34  0.45  0.31  0.35  0.13 -0.39 
Ca21Chr1_0408399 -0.12  0.02  0.53  0.09 -0.35  0.25 
Ca21Chr1_0408458  0.34  0.10  0.26  0.39  0.35  0.32 
Ca21Chr1_0408517  0.37  0.08 -0.05  0.12  0.31  0.01 
Ca21Chr1_0408576  0.43  0.57  0.29  0.30  0.12 -0.14 
Ca21Chr1_0408635  0.19  0.27  0.10  0.03  0.35 -0.47 
Ca21Chr1_0408694  0.23  0.27  0.23  0.33  0.01 -0.33 
Ca21Chr1_0408753  0.12  0.17  0.09  0.39 -0.12  0.16 
Ca21Chr1_0408812  0.13  0.01  0.18  0.39 -0.11  0.14 
Ca21Chr1_0408871  0.04  0.00  0.33  0.43  0.13 -0.09 
Ca21Chr1_0408930  0.32  0.17  0.05  0.19 -0.29 -0.23 
Ca21Chr1_0408989  0.22  0.01  0.18  0.19  0.14  0.23 
Ca21Chr1_0409048 -0.04  0.40  0.08  0.20 -0.07 -0.08 
Ca21Chr1_0409107  0.25 -0.01  0.08 -0.14  0.12  0.23 
Ca21Chr1_0409166  0.13 -0.09  0.19 -0.13  0.16  0.17 
Ca21Chr1_0409225 -0.44 -0.21  0.43  0.34 -0.77  0.35 
Ca21Chr1_0409284 -0.29  0.09  0.01 -0.19 -0.04  0.31 
Ca21Chr1_0409343  0.05 -0.27  0.10 -0.39  0.14  0.53 
Ca21Chr1_0409402 -0.27  0.46 -0.21  0.01 -0.38  0.84 
Ca21Chr1_0409461  0.26  0.84  0.02 -0.20 -0.76  1.12 
Ca21Chr1_0409520 -0.29  0.11  0.15  0.09 -0.67  0.38 
Ca21Chr1_0409579  0.01  0.25  0.01 -0.15 -0.18  1.03 
Ca21Chr1_0409638  0.73  0.26  0.39 -0.10  0.25 -0.14 
Ca21Chr1_0409697  0.26 -0.21 -0.03  0.02 -0.00  0.40 
Ca21Chr1_0409756  0.27 -0.27 -0.01  0.18  0.12 -0.12 
Ca21Chr1_0409815  0.27  0.23 -0.09  0.16  0.03 -0.03 
Ca21Chr1_0409874  0.27 -0.08  0.15 -0.25 -0.12 -0.30 
Ca21Chr1_0409933  0.18 -0.04  0.02  0.00  0.18 -0.16 
Ca21Chr1_0409992  0.08 -0.09  0.28  0.16 -0.08 -0.05 
Ca21Chr1_0410051  0.10  0.26  0.44  0.17  0.30  0.53 
Ca21Chr1_0410110  0.05  0.38  0.25 -0.31  0.05  0.18 
Ca21Chr1_0410169  0.42  0.23  0.11  0.04 -0.35  0.18 
Ca21Chr1_0410228  0.39  0.10  0.14 -0.02  0.20  0.07 
Ca21Chr1_0410287 -0.50 -0.03  0.49  0.30 -0.09  0.10 
Ca21Chr1_0410346  0.16 -0.38  0.22  0.20  0.05  0.13 
Ca21Chr1_0410405  0.12 -0.15 -0.09  0.32 -0.04 -0.34 
Ca21Chr1_0410464  0.57  0.28  0.07  0.42  0.19  0.23 
Ca21Chr1_0410523  0.27  0.22  1.30 -0.07 -0.16  0.26 
Ca21Chr1_0410582  0.33  0.31  0.41  0.11 -0.05 -0.30 
Ca21Chr1_0410641  0.09  0.16  0.41 -0.20 -0.56  0.90 
Ca21Chr1_0410700  0.14 -0.11  0.18 -0.05 -0.13  0.33 
Ca21Chr1_0410759  0.22  0.11  0.04  0.13  0.35 -0.03 
Ca21Chr1_0410818  0.08  0.16  0.11  0.11  0.38  0.18 
Ca21Chr1_0410877  0.04  0.22  0.21 -0.27  0.17  0.24 
Ca21Chr1_0410936 -0.47  0.10 -0.01  1.13 -0.60  0.73 
Ca21Chr1_0410995  1.22  0.10  0.50  0.50  0.04 -0.43 
Ca21Chr1_0411054 -0.06  0.03 -0.48  0.25 -0.15  0.89 
Ca21Chr1_0411113  0.25  0.02 -0.37  0.19  0.82 -0.03 
Ca21Chr1_0411172  0.02  0.08 -0.03 -0.29 -0.46 -0.62 
Ca21Chr1_0411231  0.48  0.14 -0.39 -0.14 -0.04  0.47 
Ca21Chr1_0411290  0.13  0.08 -0.35 -0.12 -0.39 -0.81 
Ca21Chr1_0411349  0.35  0.09  0.24 -0.06  0.48 -0.43  orf19.4526>
Ca21Chr1_0411408  0.23  0.23  0.51  0.23 -0.08 -0.07 
Ca21Chr1_0411467  0.28  0.35  0.53 -0.06  0.30 -0.32 
Ca21Chr1_0411526  0.15  0.47  0.30  0.26  0.36 -0.08 
Ca21Chr1_0411585 -0.13  0.16  0.08 -0.29 -0.01 -0.50 
Ca21Chr1_0411644  0.07  0.32  0.34  0.03  0.20  0.04 
Ca21Chr1_0411703  0.36  0.48  0.31 -0.08  0.41  0.16 
Ca21Chr1_0411762 -0.04  0.23  0.39 -0.34 -0.01  0.08 
Ca21Chr1_0411821  0.21  0.29  0.22  0.03 -0.31  0.01 
Ca21Chr1_0411880  0.19  0.28  0.43  0.33  0.12  0.05 
Ca21Chr1_0411939  1.01  0.28  0.34  0.17  0.15  0.24 
Ca21Chr1_0411998 -0.69  0.13  0.15 -0.07  0.12  0.67 
Ca21Chr1_0412057  0.62  0.43  0.54  0.06  0.12 -0.08 
Ca21Chr1_0412116 -0.02  0.00 -0.08 -0.20  0.42  0.07 
Ca21Chr1_0412175  0.11  0.04 -0.04  0.41  0.34 -0.15 
Ca21Chr1_0412234 -0.18  0.01 -0.13 -0.11  0.00  0.04 
Ca21Chr1_0412293 -0.03  0.10  0.24 -0.28  0.02  0.03 
Ca21Chr1_0412352 -0.38  0.24  0.10  0.19  0.11  0.20  orf19.4526|
Ca21Chr1_0412411  0.03  0.20  0.07 -0.03 -0.11 -0.06 
Ca21Chr1_0412470 -0.20 -0.05  0.15 -0.11  0.44  0.03 
Ca21Chr1_0412529  0.04 -0.12 -0.13  0.03 -0.05  0.14 
Ca21Chr1_0412588  0.20  0.13 -0.24  0.06 -0.04  0.35 
Ca21Chr1_0412647  1.60 -0.51  0.06 -0.08  1.06 -1.27 
Ca21Chr1_0412706 -0.03  0.00 -0.27  0.19 -0.05  0.15 
Ca21Chr1_0412765 -0.01  0.19  0.01  0.16  0.57 -0.54 
Ca21Chr1_0412824 -0.16 -0.40 -0.02 -0.03  0.10 -0.37  orf19.4525>
Ca21Chr1_0412883 -0.03  0.20 -0.04  0.06  0.23  0.08 
Ca21Chr1_0412942  0.04 -0.20  0.03 -0.63  0.05  0.15 
Ca21Chr1_0413001 -0.05  0.45  0.13 -0.12 -0.08 -0.09 
Ca21Chr1_0413060  0.17  0.17  0.25 -0.22  0.04  0.01 
Ca21Chr1_0413119  0.05 -0.08 -0.47  0.10  0.34  0.10 
Ca21Chr1_0413178 -0.13 -0.28 -0.16  0.21  0.09 -0.04 
Ca21Chr1_0413237 -0.22 -0.17 -0.46 -0.46 -0.01  1.18 
Ca21Chr1_0413296 -0.17 -0.04 -0.24 -0.23 -0.02  0.21 
Ca21Chr1_0413355  0.12  0.22 -0.04  0.01  0.23  0.21 
Ca21Chr1_0413414  0.35  0.14  1.16  0.12  0.22 -0.19 
Ca21Chr1_0413473  0.56  0.23  0.06 -0.12  0.05 -0.10 
Ca21Chr1_0413532  0.14  0.38  0.57  0.01 -0.13 -0.26 
Ca21Chr1_0413591  0.01  0.33  0.42  0.18  0.09 -0.32 
Ca21Chr1_0413650  0.17  0.40  0.05  0.03  0.18 -0.05 
Ca21Chr1_0413709  0.06  0.35  0.37  0.05 -0.02 -0.03 
Ca21Chr1_0413768  0.20  0.53  0.33  0.01 -0.14 -0.07 
Ca21Chr1_0413827 -0.07  0.33  0.34  0.10 -0.03  0.44  orf19.4525|
Ca21Chr1_0413886  0.05 -0.05  0.33 -0.49 -0.04  0.20 
Ca21Chr1_0413945 -0.13  0.12  0.16 -0.03  0.10  0.02 
Ca21Chr1_0414004 -0.08  0.22 -0.09 -0.18  0.10  0.15 
Ca21Chr1_0414063  0.29  0.41  0.23  0.28  0.08  0.23  |orf19.4524
Ca21Chr1_0414122  0.13  0.16  0.20 -0.03  0.45  0.50 
Ca21Chr1_0414181  0.06 -0.07 -0.29 -0.03 -0.08  0.05 
Ca21Chr1_0414240 -0.07  0.21  0.23 -0.12  0.02  0.48 
Ca21Chr1_0414299 -0.00  0.09  0.09  0.10  0.00 -0.02 
Ca21Chr1_0414358  0.17  0.13  0.30  0.13 -0.17 -0.07 
Ca21Chr1_0414417  0.07 -0.03  0.16  0.73 -0.06 -0.30 
Ca21Chr1_0414476  0.03  0.60 -0.84 -0.16 -0.15  0.04 
Ca21Chr1_0414535 -0.18 -0.25  0.16 -0.09  0.16 -0.47 
Ca21Chr1_0414594  0.17 -0.04  0.50  0.36 -0.05  0.08 
Ca21Chr1_0414653 -0.10  0.02  0.26  0.09  0.05 -0.41 
Ca21Chr1_0414712 -0.17 -0.00  0.22  0.00 -0.18 -0.70 
Ca21Chr1_0414771 -0.19 -0.06  0.14  0.10  0.28  0.34 
Ca21Chr1_0414830  0.02  0.12 -0.20 -0.21  0.57 -0.39 
Ca21Chr1_0414889  0.74 -0.07  0.42  0.17  0.43  0.18 
Ca21Chr1_0414948  0.36 -0.16  0.47  0.32  0.09 -0.16 
Ca21Chr1_0415007  0.07  0.39  0.26  0.31  0.03 -0.04 
Ca21Chr1_0415066  0.33  0.31  0.25  0.08  0.19  0.11 
Ca21Chr1_0415125  0.36  0.44  0.50  0.21  0.11  0.06 
Ca21Chr1_0415184  0.03  0.35  0.14  0.46 -0.06 -0.01 
Ca21Chr1_0415243  0.01  0.08  0.19  0.52 -0.39 -0.38 
Ca21Chr1_0415302 -0.86  0.09 -0.49  0.15 -0.68 -0.43 
Ca21Chr1_0415361  0.29  0.52  0.37 -0.20 -0.00 -0.57 
Ca21Chr1_0415420 -0.19  0.56  0.14 -0.12 -0.33 -0.40 
Ca21Chr1_0415479 -0.14  0.28  0.23 -0.11  0.19  0.15 
Ca21Chr1_0415538  0.12 -0.30  0.46 -0.15 -0.04 -0.55 
Ca21Chr1_0415597  1.74  0.15 -0.10  0.11 -0.03  0.18 
Ca21Chr1_0415656 -0.11  0.17  0.10 -0.46 -0.01 -0.69 
Ca21Chr1_0415715  0.22 -0.02  0.30  0.32 -0.04 -0.30 
Ca21Chr1_0415774 -0.14  0.09 -0.03  0.14 -0.14 -0.31 
Ca21Chr1_0415833  0.10  0.15  0.27  0.07  0.22 -0.15 
Ca21Chr1_0415892 -0.34  0.24 -0.17 -0.27 -0.08 -0.40 
Ca21Chr1_0415951 -0.41  0.04 -0.39 -0.04  0.04 -0.14 
Ca21Chr1_0416010 -0.75 -0.44 -0.60 -0.13  0.31 -0.43 
Ca21Chr1_0416069 -0.56 -0.02 -0.50 -0.47  0.04 -0.40 
Ca21Chr1_0416128 -1.18 -0.23 -1.29 -0.00 -0.16  0.04 
Ca21Chr1_0416187 -1.24 -0.60 -1.07  0.15  0.06  0.14 
Ca21Chr1_0416246 -1.13 -0.70 -1.27 -0.15  0.36 -0.06  <orf19.4524
Ca21Chr1_0416305 -1.43 -0.99 -1.07  0.28  0.16  0.12 
Ca21Chr1_0416364 -1.13 -1.31 -1.08 -0.25 -0.27  0.02 
Ca21Chr1_0416423 -0.83 -0.59 -0.69  0.07 -0.12  0.26  orf19.4523>
Ca21Chr1_0416482 -0.25 -0.32 -0.21  0.30 -0.06  0.22 
Ca21Chr1_0416541  0.19 -0.11 -0.15  0.32 -0.22  0.40 
Ca21Chr1_0416600  0.77  0.22  0.08  0.11  0.06  0.18 
Ca21Chr1_0416659  0.31 -0.27 -0.01  0.01  0.30  0.11 
Ca21Chr1_0416718  0.01 -0.04  0.24 -0.15  0.40  0.07 
Ca21Chr1_0416777 -0.01  0.10  0.21  0.05  0.07  0.22 
Ca21Chr1_0416836  0.18  0.13  0.24  0.31 -0.09 -0.33 
Ca21Chr1_0416895  0.09  0.04  0.13  0.15 -0.04 -0.38 
Ca21Chr1_0416954  0.09  0.07 -0.01  0.05 -0.02 -0.34 
Ca21Chr1_0417013  0.01  0.04  0.00 -0.22  0.12  0.13 
Ca21Chr1_0417072  0.04  0.47 -0.14  0.14 -0.04  0.20  orf19.4523| |orf19.4522
Ca21Chr1_0417131  0.15  0.24 -0.03  0.00  0.10  0.11 
Ca21Chr1_0417190  0.41  0.03  0.15 -0.06  0.04 -0.08 
Ca21Chr1_0417249  0.62  0.05 -0.35 -0.24 -0.01  0.08 
Ca21Chr1_0417308  0.06  0.06 -0.04 -0.58 -0.21 -0.60 
Ca21Chr1_0417367  0.19 -0.08  0.38 -0.05  0.11 -0.21 
Ca21Chr1_0417426 -0.05  0.17  0.28  0.65  0.15  0.64 
Ca21Chr1_0417485  0.40  0.06  0.20  0.24  0.02 -0.03  <orf19.4522
Ca21Chr1_0417544  0.56 -0.42  0.13  0.02 -0.62  0.04 
Ca21Chr1_0417603  0.11  0.72  0.02  0.44  0.24  0.03 
Ca21Chr1_0417662  0.39 -0.25  0.35  0.10  0.54  0.15 
Ca21Chr1_0417721 -0.05  0.23 -0.20  0.60  0.30  0.42 
Ca21Chr1_0417780    NA    NA    NA    NA    NA    NA
Ca21Chr1_0417839 -0.28 -0.59 -0.53  0.20  0.11  0.24 
Ca21Chr1_0417898  0.09 -0.51 -0.29  0.42  0.01 -0.12 
Ca21Chr1_0417957 -0.02 -0.19 -0.06  0.07 -0.05 -0.14 
Ca21Chr1_0418016  0.04  0.17  0.09 -0.05  0.04 -0.12  |orf19.3661
Ca21Chr1_0418075  0.06  0.04  0.21  0.23 -0.10 -0.40 
Ca21Chr1_0418134  0.04  0.22  0.33 -0.09  0.06 -0.22 
Ca21Chr1_0418193  0.69  0.05  0.44 -0.21  0.29  0.12 
Ca21Chr1_0418252  0.26  0.26  0.41  0.05  0.03  0.29 
Ca21Chr1_0418311  0.41  0.05  0.49  0.06 -0.18  0.26 
Ca21Chr1_0418370  0.42  0.27  0.32 -0.06  0.30 -0.27 
Ca21Chr1_0418429  0.61  0.37  0.79  0.10 -0.01 -0.21 
Ca21Chr1_0418488  0.22  0.51  0.31 -0.20 -0.25 -0.36 
Ca21Chr1_0418547 -0.29  0.15  0.39  0.06  0.04 -0.10 
Ca21Chr1_0418606 -0.21  0.36  0.19  0.06  0.12 -0.03 
Ca21Chr1_0418665  0.31  0.20  0.34 -0.11  0.52  0.04 
Ca21Chr1_0418724  0.36  0.38  0.56 -0.28 -0.18  0.04 
Ca21Chr1_0418783  0.06  0.49  0.68  0.02 -0.08  0.44 
Ca21Chr1_0418842  0.15  0.28  0.16 -0.01  0.16 -0.02 
Ca21Chr1_0418901  0.57  0.46  0.56 -0.22  0.12  0.41 
Ca21Chr1_0418960  0.97  0.32  0.90  0.45  0.30  0.05 
Ca21Chr1_0419019 -0.12  0.17  0.28 -0.20  0.21 -0.17 
Ca21Chr1_0419078  0.15  0.19  0.04  0.03  0.04 -0.12 
Ca21Chr1_0419137  0.29  0.17  0.52 -0.24  0.17  0.03 
Ca21Chr1_0419196  0.85  0.32  0.29 -0.04  0.16 -0.02 
Ca21Chr1_0419255  0.23  0.90  0.25  0.10  0.00 -0.00 
Ca21Chr1_0419314  0.24 -0.00  0.32  0.20 -0.03  0.09 
Ca21Chr1_0419373  0.55  0.27  0.26  0.11 -0.03  0.08 
Ca21Chr1_0419432 -0.12  0.75  1.06 -0.12  0.11  0.51 
Ca21Chr1_0419491  0.22  0.05  0.37 -0.38  0.10 -0.43 
Ca21Chr1_0419550  0.15  0.06  0.48 -0.07  0.11  0.22 
Ca21Chr1_0419609  0.05  0.08  0.08 -0.11  0.19 -0.32 
Ca21Chr1_0419668  0.12  0.51  0.36  0.07  0.09 -0.09 
Ca21Chr1_0419727  0.32 -0.12  0.41  0.33  0.66 -0.27 
Ca21Chr1_0419786 -0.14 -0.17  0.71 -0.07  0.46  0.39 
Ca21Chr1_0419845 -0.09  0.29  0.42  0.03  0.48  0.04 
Ca21Chr1_0419904  0.39  0.06  0.34  0.24  0.12  0.03 
Ca21Chr1_0419963 -0.13  0.10  0.43  0.47 -0.01 -0.06 
Ca21Chr1_0420022  0.09  0.22  0.35  0.11 -0.29 -0.19 
Ca21Chr1_0420081  0.30  0.17 -0.04 -0.07 -0.14  0.03 
Ca21Chr1_0420140  0.36  0.45  0.09  0.63  0.06  0.25 
Ca21Chr1_0420199  0.36  0.01  0.10  0.11 -0.08  0.22 
Ca21Chr1_0420258 -0.12 -0.12  0.43  0.60  0.29 -1.10 
Ca21Chr1_0420317  0.20  0.18  0.87  0.49  0.47  0.75 
Ca21Chr1_0420376 -0.37  0.20  0.45  0.15 -0.06  0.05 
Ca21Chr1_0420435 -0.49  0.11 -0.17  0.08  0.26  0.45 
Ca21Chr1_0420494  0.25  0.07  0.29  0.23 -0.06  0.38  <orf19.3661
Ca21Chr1_0420553    NA    NA    NA    NA    NA    NA
Ca21Chr1_0420612 -0.81 -0.12  0.41  0.24  0.18 -0.69 
Ca21Chr1_0420671 -0.19  0.14  0.69  0.07  0.65 -0.09 
Ca21Chr1_0420730 -0.27 -0.43 -0.55  0.31 -0.72  0.19 
Ca21Chr1_0420789  0.25 -0.31 -0.52 -0.16 -0.11 -0.51 
Ca21Chr1_0420848  0.08 -0.39  0.01 -0.13  0.15  0.05 
Ca21Chr1_0420907 -0.15 -0.38  0.19  0.17 -0.18  0.00 
Ca21Chr1_0420966 -0.15 -0.11  0.18  0.04 -0.24  0.08 
Ca21Chr1_0421025 -0.34  0.05  0.23 -0.12  0.18  0.10 
Ca21Chr1_0421084 -0.64  0.25  0.13  0.33  0.03  0.16 
Ca21Chr1_0421143  0.06  0.28  0.30  0.33  0.03 -0.52 
Ca21Chr1_0421202  0.33  0.14  0.10  0.10 -0.12 -0.35 
Ca21Chr1_0421261  0.25  0.10  0.01 -0.27 -0.24 -0.30  |orf19.3663
Ca21Chr1_0421320  0.07  0.33  0.38  0.13  0.35 -0.18 
Ca21Chr1_0421379  0.00  0.33  0.40  0.51 -0.17 -0.31 
Ca21Chr1_0421438  0.08  0.35  0.61  0.47  0.04 -0.39 
Ca21Chr1_0421497  0.31  0.40  0.35 -0.05  0.22 -0.51 
Ca21Chr1_0421556  0.10  0.16  0.25  0.10  0.11 -0.15 
Ca21Chr1_0421615  0.60  0.01  0.14  0.08  0.22 -0.12 
Ca21Chr1_0421674  0.14  0.22  0.46  0.39  0.05  0.17 
Ca21Chr1_0421733 -0.22 -0.16  0.47  0.16  0.20 -0.35 
Ca21Chr1_0421792  0.16  0.46  0.42  0.26 -0.10  0.20 
Ca21Chr1_0421851 -0.05  0.12  0.46  0.43  0.04  0.16 
Ca21Chr1_0421910  0.28  0.35  0.56  0.04  0.01  0.10 
Ca21Chr1_0421969  0.01 -0.01  0.38  0.18 -0.23 -0.16 
Ca21Chr1_0422028 -0.20  0.15  0.35  0.56  0.38 -0.23 
Ca21Chr1_0422087  0.47  0.33  0.29  0.25 -0.10 -0.25 
Ca21Chr1_0422146  0.42  0.40  0.34  0.46  0.31 -0.18 
Ca21Chr1_0422205  0.27  0.22  0.41  0.17  0.01  0.10 
Ca21Chr1_0422264 -0.07  1.11  0.50  0.33 -0.03  0.03 
Ca21Chr1_0422323 -0.02  0.16  0.32  0.50  0.11 -0.09 
Ca21Chr1_0422382  0.16  0.19  0.37  0.60 -0.32  0.04 
Ca21Chr1_0422441  0.37  0.04  0.16  0.08  0.01  0.83 
Ca21Chr1_0422500  0.26 -0.00 -0.14  0.13 -0.10  0.02 
Ca21Chr1_0422559  0.11 -0.19  0.18  0.44 -0.35  0.14 
Ca21Chr1_0422618 -0.13  0.16  0.33  0.19  0.16  0.04 
Ca21Chr1_0422677 -0.07  0.06  0.19  0.34  0.37 -0.28 
Ca21Chr1_0422736  0.32 -0.21  0.03  0.41  0.11 -0.06 
Ca21Chr1_0422795  0.26 -0.08  0.28  0.25 -0.00  0.09 
Ca21Chr1_0422854  0.17 -0.09 -0.07  0.12 -0.11 -0.03 
Ca21Chr1_0422913 -0.14  0.03 -0.26 -0.21  0.23  0.04 
Ca21Chr1_0422972 -0.06 -0.08  0.36  0.23  0.09  0.51 
Ca21Chr1_0423031  0.36  0.24  0.17  0.23 -0.05  0.35 
Ca21Chr1_0423090  0.11  0.13 -0.13  0.15 -0.22  0.28 
Ca21Chr1_0423149 -0.27  0.25  0.14 -0.01 -0.60 -0.01 
Ca21Chr1_0423208 -0.04  0.30  0.12 -0.30 -0.05  0.07 
Ca21Chr1_0423267  0.10  0.27  0.50 -0.37 -0.02 -0.80 
Ca21Chr1_0423326  0.06  0.13  0.94  0.02  0.23  0.05 
Ca21Chr1_0423385  0.24  0.34  0.36 -0.36  0.23 -0.17 
Ca21Chr1_0423444  0.30  0.26  0.34 -0.16  0.60 -0.11 
Ca21Chr1_0423503  0.12  0.13  0.48  0.03  0.37  0.02 
Ca21Chr1_0423562  0.12 -0.21  0.33  0.12 -0.15  0.12 
Ca21Chr1_0423621 -0.17  0.52  0.28  0.16  0.25 -0.34 
Ca21Chr1_0423680  0.09  0.28  0.41 -0.01 -0.27 -0.42 
Ca21Chr1_0423739  0.02  0.06  0.66  0.12 -0.14 -0.21 
Ca21Chr1_0423798 -0.14  0.24  0.06  0.07  0.31  0.02 
Ca21Chr1_0423857  0.09  0.35 -0.14 -0.12  0.03 -0.29  <orf19.3663
Ca21Chr1_0423916  0.07  0.45  0.09 -0.20  0.01 -0.26 
Ca21Chr1_0423975  0.02  0.47 -0.33 -0.20 -0.22 -0.21 
Ca21Chr1_0424034 -0.05  0.14 -0.24  0.05 -0.09  0.29 
Ca21Chr1_0424093 -0.15 -0.02 -0.49  0.36  0.07 -0.01 
Ca21Chr1_0424152 -0.70 -0.31 -0.32  0.38 -0.17  0.83 
Ca21Chr1_0424211 -0.41 -0.17 -0.71 -0.05  0.25  0.10 
Ca21Chr1_0424270 -0.09  0.25 -0.48  0.18  0.15  0.41 
Ca21Chr1_0424329  0.23  0.13 -0.46 -0.09 -0.09  0.39 
Ca21Chr1_0424388  0.09 -0.09 -0.29  0.29 -0.01  0.78 
Ca21Chr1_0424447 -0.00 -0.25 -0.38 -0.28 -0.27  0.24 
Ca21Chr1_0424506  0.04  0.00 -0.67  0.38 -0.46 -0.18 
Ca21Chr1_0424565 -0.39  0.39 -0.36  0.23 -0.44  0.36 
Ca21Chr1_0424624 -0.44 -0.31 -0.88 -0.05 -0.22  0.82 
Ca21Chr1_0424683  0.10  0.01 -0.80  0.11  0.27 -0.11 
Ca21Chr1_0424742  0.13 -0.28 -0.72 -0.11  0.10  0.25 
Ca21Chr1_0424801 -0.18  0.08 -0.28  0.25  0.13  0.24 
Ca21Chr1_0424860  0.30 -0.16 -0.61 -0.31  0.20 -0.24 
Ca21Chr1_0424919 -0.15 -0.66  0.31  0.13  0.42  0.02 
Ca21Chr1_0424978 -0.28  0.03 -0.29 -0.44  0.21  0.44 
Ca21Chr1_0425037 -0.03  0.08 -0.07  0.01  0.48  0.09 
Ca21Chr1_0425096 -0.19  0.11 -0.23 -0.37  0.22  0.51 
Ca21Chr1_0425155  0.25  0.21 -0.32 -0.26  0.06  0.04 
Ca21Chr1_0425214 -0.00  0.13 -0.28 -0.27 -0.01 -0.06 
Ca21Chr1_0425273 -0.12 -0.02 -0.17 -0.06 -0.43  0.22 
Ca21Chr1_0425332  0.06 -0.12 -0.08 -0.37 -0.31  0.29 
Ca21Chr1_0425391  0.06  0.13 -0.18 -0.29 -0.19  0.61 
Ca21Chr1_0425450  0.16 -0.20  0.02  0.21 -0.09 -0.02 
Ca21Chr1_0425509  0.30 -0.03  0.43  0.28 -0.03  0.19 
Ca21Chr1_0425568 -0.25  0.24  0.22  0.43  0.15 -0.04 
Ca21Chr1_0425627  0.19 -0.04  0.08  0.18  0.14  0.06 
Ca21Chr1_0425686  0.08 -0.05  0.04  0.02  0.28  0.29 
Ca21Chr1_0425745  0.01  0.10 -0.01  0.25  0.23  0.66 
Ca21Chr1_0425804 -0.11 -0.02 -0.07  0.16  0.26  0.45 
Ca21Chr1_0425863  0.11 -0.00  0.03 -0.13 -0.58  0.60 
Ca21Chr1_0425922  0.71  0.32 -0.22 -0.01 -0.16 -0.23 
Ca21Chr1_0425981  0.17  0.27 -0.13 -0.11  0.40  1.49  orf19.3663.1>
Ca21Chr1_0426040  0.10  0.03  0.03  0.01 -0.21  0.54 
Ca21Chr1_0426099  0.10  0.14 -0.48 -0.01 -0.01  0.36 
Ca21Chr1_0426158  0.24 -0.33 -0.30 -0.01 -0.30 -0.19 
Ca21Chr1_0426217  0.36  0.13 -0.43 -0.20 -0.22 -0.02 
Ca21Chr1_0426276  0.43  0.20 -0.13  0.06  0.18 -0.03 
Ca21Chr1_0426335  0.33  0.15 -0.03 -0.14 -0.25  0.10 
Ca21Chr1_0426394  0.17  0.20 -0.30  0.09 -0.12 -0.08 
Ca21Chr1_0426453  0.22  0.80 -0.06  0.13  0.13 -0.12 
Ca21Chr1_0426512  0.18  0.12 -0.44  0.18 -0.42  0.07 
Ca21Chr1_0426571  0.23  0.11 -0.13  0.27 -0.01  0.09 
Ca21Chr1_0426630  0.21  0.16 -0.28  0.17 -0.12 -0.19  orf19.3663.1|
Ca21Chr1_0426689 -0.20  0.30 -0.34  0.16  0.07 -0.54 
Ca21Chr1_0426748 -0.14  0.25  0.16  0.12 -0.11 -0.12 
Ca21Chr1_0426807  0.14 -0.11 -0.22 -0.02 -0.07 -0.13 
Ca21Chr1_0426866  0.04  0.29 -0.32 -0.12 -0.03 -0.45 
Ca21Chr1_0426925  0.31 -0.03 -0.29 -0.17  0.05 -0.14 
Ca21Chr1_0426984  0.04 -0.05 -0.60 -0.01 -0.03 -0.15 
Ca21Chr1_0427043 -0.52  0.17 -0.35 -0.21 -0.32  0.30 
Ca21Chr1_0427102  0.19  0.13 -0.32  0.37 -0.09  0.46 
Ca21Chr1_0427161 -0.09  0.23  0.21 -0.05 -0.60  0.22 
Ca21Chr1_0427220 -0.07  0.24 -0.30  0.00 -0.37  0.22 
Ca21Chr1_0427279 -0.34  0.14 -0.66  0.10 -0.01  0.56 
Ca21Chr1_0427338  0.12  0.13 -0.54  0.30 -0.14  0.72 
Ca21Chr1_0427397  0.05 -0.14 -0.58  0.04 -0.24  0.88 
Ca21Chr1_0427456  0.28 -0.13 -0.56 -0.15  0.02  0.81 
Ca21Chr1_0427515  0.46 -0.27 -0.18 -0.17 -0.34  0.69 
Ca21Chr1_0427574  0.56 -0.11 -0.20 -0.04 -0.44  0.53 
Ca21Chr1_0427633  0.41 -0.57 -0.08 -0.10  0.22  0.06 
Ca21Chr1_0427692  0.26 -0.06 -0.19 -0.05 -0.05  0.64 
Ca21Chr1_0427751 -0.05  0.20  1.32  0.03  0.11 -0.05 
Ca21Chr1_0427810  0.35 -0.20  0.33 -0.19  0.05  0.18  orf19.3664>
Ca21Chr1_0427869  0.36  0.27  0.58 -0.07  0.06 -0.16 
Ca21Chr1_0427928  0.05  0.31  0.06  0.22  0.61 -0.00 
Ca21Chr1_0427987  0.26  0.38  0.57 -0.07  0.01  0.21 
Ca21Chr1_0428046  0.27  0.24  0.53 -0.01  0.11  0.29 
Ca21Chr1_0428105  0.40  0.20  0.39  0.10  0.08 -0.04 
Ca21Chr1_0428164  0.09 -0.13  0.64 -0.16  0.95  0.33 
Ca21Chr1_0428223  0.49  0.87  0.48  0.23  0.14  0.23 
Ca21Chr1_0428282  0.28 -0.00  0.35  0.06  0.26  0.08 
Ca21Chr1_0428341  0.20  0.26  0.05  0.13  0.08  0.34 
Ca21Chr1_0428400  0.56  0.34  0.07  0.24  0.02  0.27 
Ca21Chr1_0428459  0.22  0.35  0.04 -0.65  0.10  0.49 
Ca21Chr1_0428518  0.21  0.52  0.35 -0.13  0.32  0.32 
Ca21Chr1_0428577  0.32  0.23  0.78  0.50  0.85  0.46 
Ca21Chr1_0428636  0.25  0.07  0.52 -0.14  0.03  0.15 
Ca21Chr1_0428695 -0.01 -0.05  0.33  0.09  0.05 -0.16 
Ca21Chr1_0428754  0.62  0.32  0.58  0.11 -0.16  0.23 
Ca21Chr1_0428813  0.30  0.23  0.07 -0.70  0.23  0.01  orf19.3664|
Ca21Chr1_0428872  0.25  0.28  0.09  0.03  0.29  0.27 
Ca21Chr1_0428931    NA    NA    NA    NA    NA    NA
Ca21Chr1_0428990 -0.25 -0.04 -0.82  0.11  0.68  0.34 
Ca21Chr1_0429049  0.05 -0.40 -0.45 -0.07  0.28  0.51 
Ca21Chr1_0429108 -0.13 -0.21 -0.62 -0.04  0.07  0.11 
Ca21Chr1_0429167 -0.61 -0.03 -0.62 -0.16 -0.28 -0.19 
Ca21Chr1_0429226    NA    NA    NA    NA    NA    NA
Ca21Chr1_0429285  1.31 -0.29  0.11 -0.00 -0.27  0.66 
Ca21Chr1_0429344 -0.14  0.00 -0.16 -0.41 -0.19 -0.40  orf19.3665>
Ca21Chr1_0429403  0.34  0.15 -0.21 -0.13  0.26  0.27 
Ca21Chr1_0429462  0.28  0.23  0.09  0.12  0.15  0.12 
Ca21Chr1_0429521  0.45  0.20  0.62 -0.12  0.11  0.19 
Ca21Chr1_0429580 -0.04 -0.64  0.96 -0.24 -0.01  0.49 
Ca21Chr1_0429639  0.37  0.17  0.03 -0.03  0.29  0.17 
Ca21Chr1_0429698 -0.04  0.15  0.18  0.11  0.27  0.59 
Ca21Chr1_0429757  0.69 -0.03  0.21  0.35  0.50  0.26 
Ca21Chr1_0429816  0.00  0.17  0.19 -0.07  0.12  0.01 
Ca21Chr1_0429875 -0.03 -0.12  0.15 -0.00 -0.33 -0.23 
Ca21Chr1_0429934  0.07  0.27  0.18  0.11  0.02 -0.02 
Ca21Chr1_0429993 -0.24  0.04  0.32 -0.07  0.06  0.10 
Ca21Chr1_0430052 -0.04  0.05  0.37  0.66 -0.11 -0.78 
Ca21Chr1_0430111 -0.03  0.20  0.31  0.04  0.16 -0.29 
Ca21Chr1_0430170  0.18  0.10  0.46  0.02  0.17  0.41 
Ca21Chr1_0430229  0.56  0.19  0.20 -0.24 -0.03 -0.20 
Ca21Chr1_0430288  0.51  0.29  0.54  0.20 -0.00 -0.34 
Ca21Chr1_0430347  0.31  0.28  0.36  0.16  0.11 -0.32 
Ca21Chr1_0430406  0.15  0.31  0.23  0.14  0.08  0.15 
Ca21Chr1_0430465 -0.07 -0.18  0.27  0.30 -0.36 -0.21 
Ca21Chr1_0430524  0.02  0.12  0.06 -0.05  0.18  0.25  |orf19.3666 orf19.3665|
Ca21Chr1_0430583  0.46  0.11  0.26 -0.19 -0.17  0.11 
Ca21Chr1_0430642  0.15  0.28  0.13  0.21 -0.33  0.56 
Ca21Chr1_0430701  0.17  0.11  0.29 -0.12 -0.19 -0.06 
Ca21Chr1_0430760 -0.08 -0.06  0.19  0.03  0.02  0.00 
Ca21Chr1_0430819  0.12  0.08  0.55 -0.29  0.08  0.56 
Ca21Chr1_0430878  0.12  0.26 -0.10  0.14 -0.48  0.41 
Ca21Chr1_0430937  0.41  0.27  0.34  0.02  0.05  0.71 
Ca21Chr1_0430996  0.19  0.01  0.01  0.21  0.19  0.55 
Ca21Chr1_0431055  0.22  0.32 -0.09 -0.05  0.15 -0.03 
Ca21Chr1_0431114 -0.00  0.03 -0.04 -0.41 -0.02 -0.42 
Ca21Chr1_0431173  0.24  0.06  0.46  0.05 -0.30  0.17 
Ca21Chr1_0431232  0.18  0.10  0.21 -0.16  0.17 -0.12 
Ca21Chr1_0431291  0.05  0.15  0.10  0.10  0.04 -0.13 
Ca21Chr1_0431350  0.10 -0.00  0.09  0.01  0.05 -0.02 
Ca21Chr1_0431409  0.08 -0.19 -0.14 -0.13 -0.12 -0.05 
Ca21Chr1_0431468  0.18  0.03  0.12 -0.08 -0.23 -0.09 
Ca21Chr1_0431527 -0.05  0.23  0.16 -0.03 -0.26  0.24 
Ca21Chr1_0431586 -0.21  0.14  0.03 -0.37 -0.84 -0.02 
Ca21Chr1_0431645  0.06  0.05  0.13 -0.07 -0.16 -0.23 
Ca21Chr1_0431704 -0.45 -0.01  0.11 -0.01 -0.03 -0.15 
Ca21Chr1_0431763 -0.61 -0.15  0.04  0.01 -0.15 -0.34
[truncated: 12,832,318 more chars]
